# Regioselectivity Study of Contrasteric Aromatic Claisen Rearrangements and Their Utilization in the Total Syntheses of 5‑Hydroxymellein and Botyroisocoumarin A

**DOI:** 10.1021/acs.joc.5c01268

**Published:** 2025-09-04

**Authors:** David K. Tanas, Runzi Li, Harris H. Khan, Tangela C. Johnson, Logan E. Brown, Philip M. West, Emily J. Guin, Sydney E. Jones, Nolan K. Garci, Drew M. Canning, Emily J. Ramirez, Gurjant S. Sekhon, Tanner H. Pierce, Abraham Ustoyev, Mitchell P. Croatt

**Affiliations:** Department of Chemistry and Biochemistry, 14616 University of North Carolina at Greensboro, Greensboro, North Carolina 27402, United States

## Abstract

In this study, we present data on the regioselectivity of aromatic Claisen rearrangements with *meta*-substituted benzenes. A variety of gentisic acid, tetralin, and *m*-salicylamide derivatives were synthesized to test the potential of an internal base to direct the regioselectivity of *ortho*-alkylation. A key mechanistic insight hinges on a reversible [3,3]-sigmatropic rearrangement step, supported by ^1^H NMR studies of the isomerization of a *Z* to *E* crotyl group. This evidence supports the potential for the keto–enol tautomerization step of the aromatic Claisen rearrangement to be the rate-determining step. A ^13^C NMR study shows that this kinetic insight can be used to direct regioselectivity by correlating the carbonyl carbon shifts of salicylamides to the regioselectivity of the associated aromatic Claisen rearrangement. Systems with rigid, in-plane bases, especially *meta*-lactone carbonyls, are highly regioselective for the contrasteric position. The utility of this regioselectivity was used in 4-step syntheses of 5-hydroxymellein and botyroisocoumarin A.

## Graphical abstract

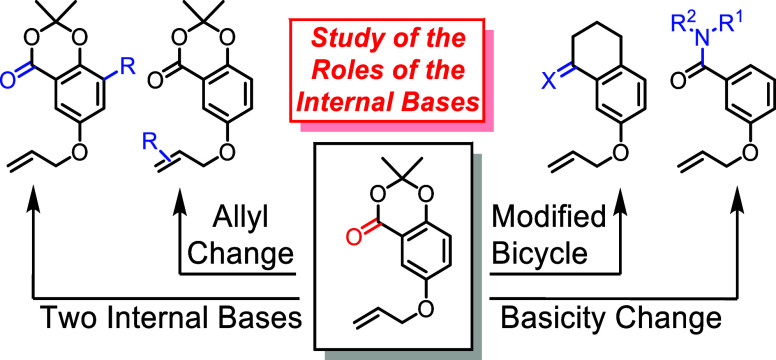

## Introduction

Reactions that form carbon–carbon bonds are crucial to the synthesis of complex molecules. One such reaction is the aromatic Claisen rearrangement, a [3,3]-sigmatropic rearrangement discovered by Rainer Ludwig Claisen that utilizes aryl allyl ethers to generate *ortho*-alkylated phenols.^1^ This classic reaction has been used in several total syntheses of biologically important molecules.^2–9^ Despite the prevalence of the aromatic Claisen rearrangement, most of the cases utilize a monosubstituted benzene, a *para*-substituted benzene, or an *ortho*-substituted benzene to obviate regioselectivity issues (Figure 1).^9–11^ There are also regioselective aromatic Claisen rearrangements of naphthalene systems, and the regioselectivity of this system is due to retaining aromaticity in the transition state for one regioisomer.^12–16^ The dearth of examples of *meta*-substituted benzenes reacting in an aromatic Claisen rearrangement is likely due to the lack of regioisomeric control of the reaction. Herein, we present examples of highly regioselective aromatic Claisen rearrangements with an analysis to support a regioselectivity-directing internal base. It is anticipated that this study will enable step-economical syntheses of polysubstituted aromatic cores of complex molecules.

**Figure 1:**
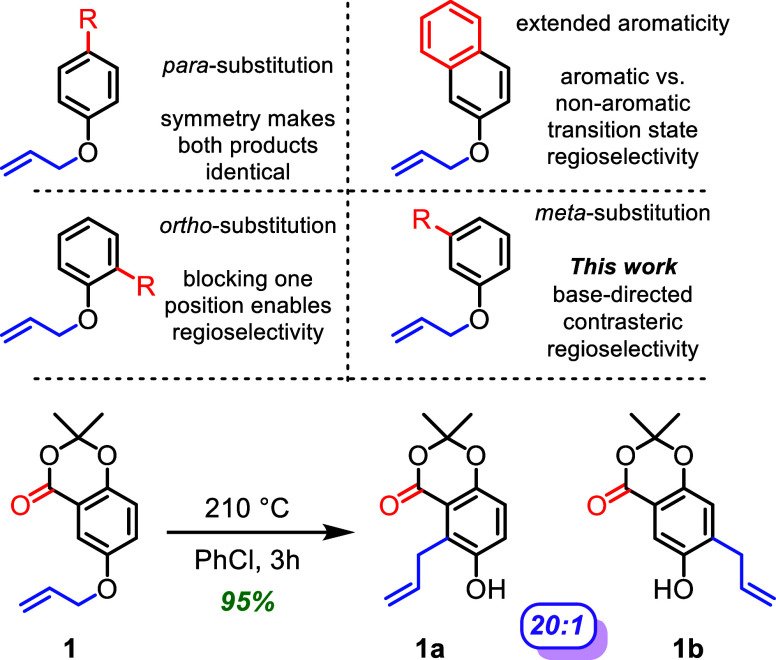
Discovery of a highly regioselective contrasteric aromatic Claisen rearrangement.

During our efforts toward the total synthesis of ambuic acid, an epoxyquinol natural product with promising biological activity against methicillin-resistant *Staphylococcus aureus* (MRSA),^17^ it was discovered that the aromatic Claisen rearrangement was highly regioselective with a 20:1 ratio of products in favor of the more sterically hindered position (Figure 1). It was anticipated that the aromatic Claisen rearrangement would have been nonselective and that we would require a subsequent chemoselective functionalization. The unexpected, contrasteric regioselectivity raised questions about the potential of the lactone carbonyl to direct the reaction by acting as an internal base. In this paper, various aromatic substitution patterns and internal bases are tested to understand the source of the observed contrasteric regioselectivity and demonstrate its utility in synthesis.

## Results and Discussion

### Proposed Mechanism

The aromatic Claisen rearrangement involves a [3,3] sigmatropic rearrangement followed by tautomerization to convert an allyloxybenzene into an *ortho*-substituted allyl phenol (Scheme 1). This reaction has been studied extensively, however, the mechanistic analysis typically examines the sigmatropic rearrangement and does not consider the activation barrier for tautomerization.^18–20^ The p*K*_a_ of the proton that is removed in the tautomerization process has been estimated in some cases to be approximately −1, which rationalizes the assumption that the sigmatropic rearrangement is the rate-determining step and that the tautomerization is rapid and irreversible.^21^ This assumption implies that the regioselectivity relies solely on the sigmatropic rearrangement, which is likely true when there are solvent molecules capable of shuttling protons. However, the kinetics of the process when run in a solvent without basic or acidic sites have shown that tautomerization has the potential to be the rate-determining step.^22^ Thus, if the starting material has an internal base and the reaction is run in the proper solvent, it is hypothesized that a base-directed, contrasteric aromatic Claisen rearrangement can result (Scheme 1).

**Scheme 1:**
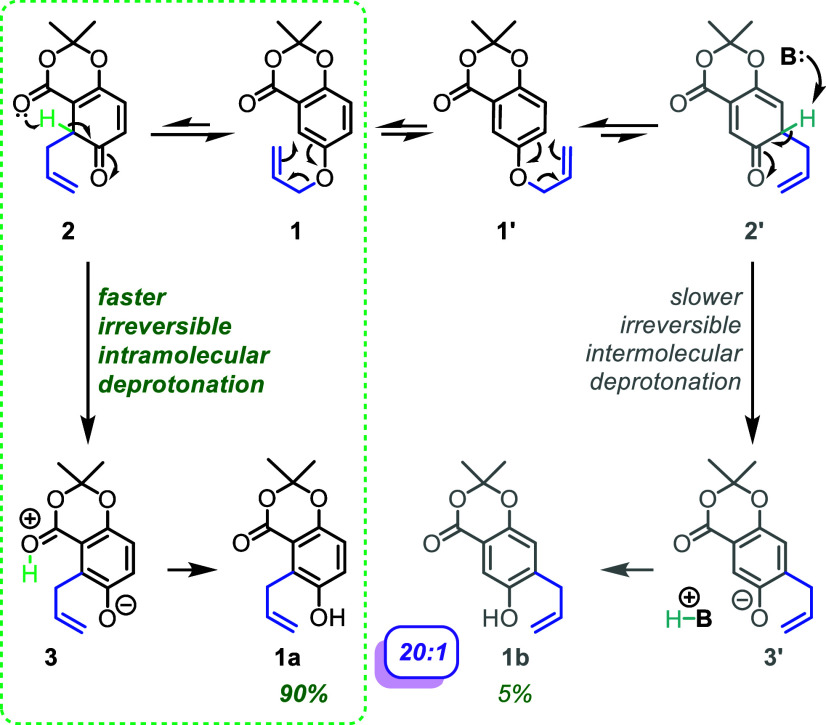
Proposed Mechanism of the Regioselective Aromatic Claisen Rearrangement

The starting allylic ether can undergo bond rotation to convert between rotamers (**1** and **1′**). If the reaction proceeds through a [3,3] sigmatropic rearrangement to allylate the contrasteric position (**2**), the lactone carbonyl is positioned to deprotonate the acidic proton. This aromatizes the ring to irreversibly form zwitterionic intermediate **3**. The final *ortho*-alkylated phenol (**1a**) is then generated by intermolecular proton transfers, and this would be after the rate-determining step. The analogous process can take place with the other rotamer (**1′**), however, the deprotonation of **2′** is predicted to be slower since it would necessarily be an intermolecular process.

The potential for a fast, intramolecular deprotonation step to impact the regioselectivity of the aromatic Claisen rearrangement is explored by studying various types of aromatic groups and internal bases. These different aromatic systems include various allyl groups, competing internal bases, modified bicyclic rings, and a variety of amides. Herein, we present the impact on regioselectivity of allyl migration to decipher the potential role of an internal base on the aromatic Claisen rearrangement.

### Lactone Allyl-Derivatives

A variety of allyl and propargyl groups were installed onto the starting phenol using standard alkylation techniques to generate a focused library of structures (**1, 4–12**, Figure 2).^23–25^ All substrates were dissolved individually in dry chlorobenzene and reacted between 3 and 17 h at 210 °C for the aromatic Claisen rearrangement, depending on the time required for reaction completion. Yields and regioselectivities are shown in Figure 2, with respect to varying the allyl and propargyl substrates.

**Figure 2:**
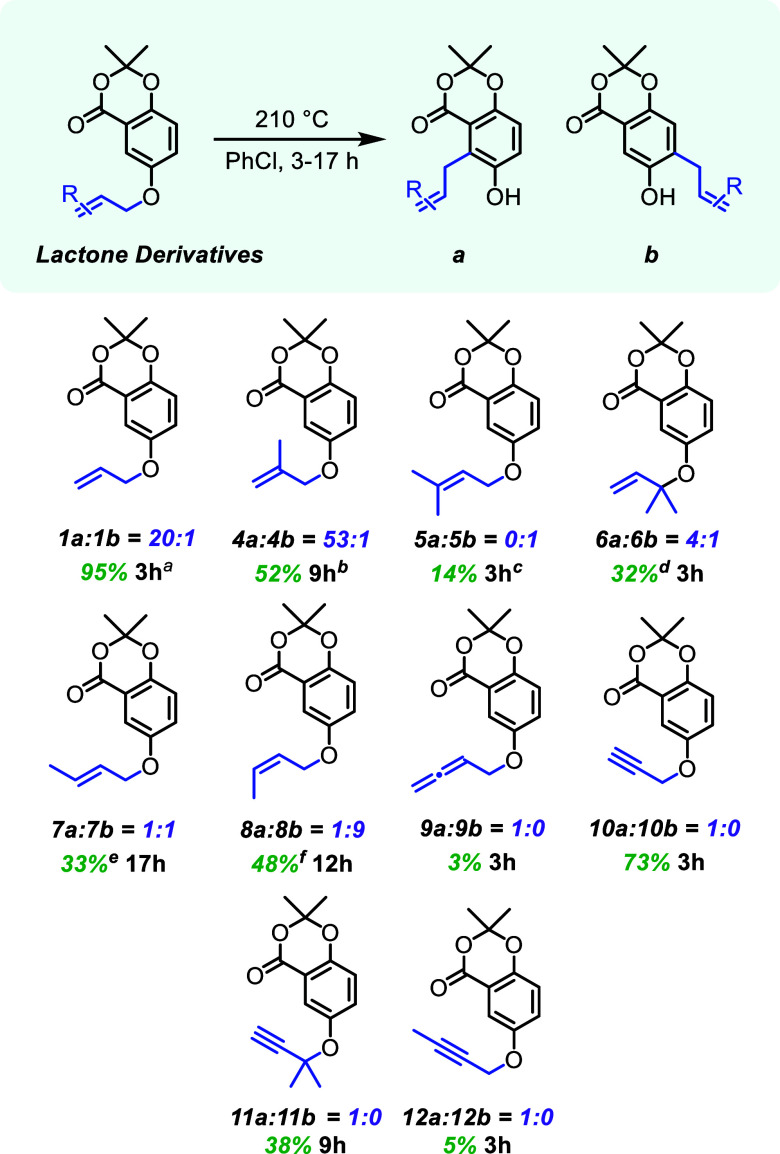
Lactone allyl-derivatives with aromatic Claisen rearrangement yields and regioselectivities. ^1^H NMR analysis was used to determine the regioselectivity before chromatographic isolation of products. Combined isolated yields of **a** and **b** are reported for the aromatic Claisen rearrangement. ^*a*^Additionally tested on a 14.1 mmol scale with a yield of 64% and an **a**/**b** ratio of 18:1. ^*b*^Cyclized to form a dihydrofuran ring as the major product. ^*c*^DMF as the solvent due to stability issues in PhCl. ^*d*1^H NMR yield. The crude mixture was not purified due to decomposition of the product on silica gel chromatography. ^*e*^ *E*/*Z* = 3:1 for **7**. ^*f*^ *E*/*Z* = 1:22 for **8**.

The allyl derivatives were analyzed to ascertain how steric bulk of the migratory group can affect the regioselectivity in the rearrangement. Model substrate **1** (Figure 1) was highly regioselective (20:1) for the contrasteric site (Figure 2). Methylation at the internal methine (**4**) exhibits even higher selectivity for the contrasteric site. Both *E* (**7**) and *Z* (**8**) internal olefins have decreased regioselectivity with 1:1 and 1:9 ratios, respectively. This is likely due to differing steric interactions between the methyl and carbonyl groups in the reactive geometry for sigmatropic rearrangement. The prenyl analogue (**5**) displayed complete regioselectivity toward the less sterically hindered position, producing **5b**. This is presumably due to the steric interactions between the geminal dimethyl groups and the carbonyl. The allylic dimethyl substrate (**6**) had slightly less regioselectivity toward the contrasteric site, as compared to the model system (**1**). When an allene was used in place of the alkene (**9**), the substrate exhibited complete regioselectivity to form **9a**, although the yield suffered due to the instability of the starting material under the high temperatures required for the rearrangement.

The propargylic systems differ from the allylic systems with respect to hybridization/geometry and oxidation states, resulting in benzopyran formation instead of *ortho*-alkylation.^26^ As compared to the allylic systems, the propargylic group can only be functionalized at the methylene and terminal positions. The initial propargyl ether (**10**) showed similar results to the model substrate (**1**), where the only product observed resulted from the reaction at the more sterically hindered site. The addition of two methyl groups at the propargylic position (**11**) had no effect on the regioselectivity with only the contrasteric product observed despite diminished efficiency. Similarly, functionalizing the terminal end of the propargyl group (**12**) did not affect the contrasteric regioselectivity. From the data of Figure 2, there is a strong preference for producing the contrasteric product in the presence of the fixed lactone carbonyl base, with the only exceptions being systems with significant steric hindrance affecting the reactive conformations.

### Double *meta*-Substitution of Gentisic Acid Derivatives

A second suite of substrates, differentiated by additional *meta*-substitution, were synthesized to interrogate regioselectivity differences that may arise from a competing basic moiety or electronic perturbation of the arene (Figure 3). The synthesis for these substrates, as with the previous allyl substrates, began with gentisic acid (see Supporting Information).^27–32^ Each individual substrate was dissolved in dry chlorobenzene and reacted at 180 or 210 °C for 3–18 h for the aromatic Claisen rearrangement

**Figure 3:**
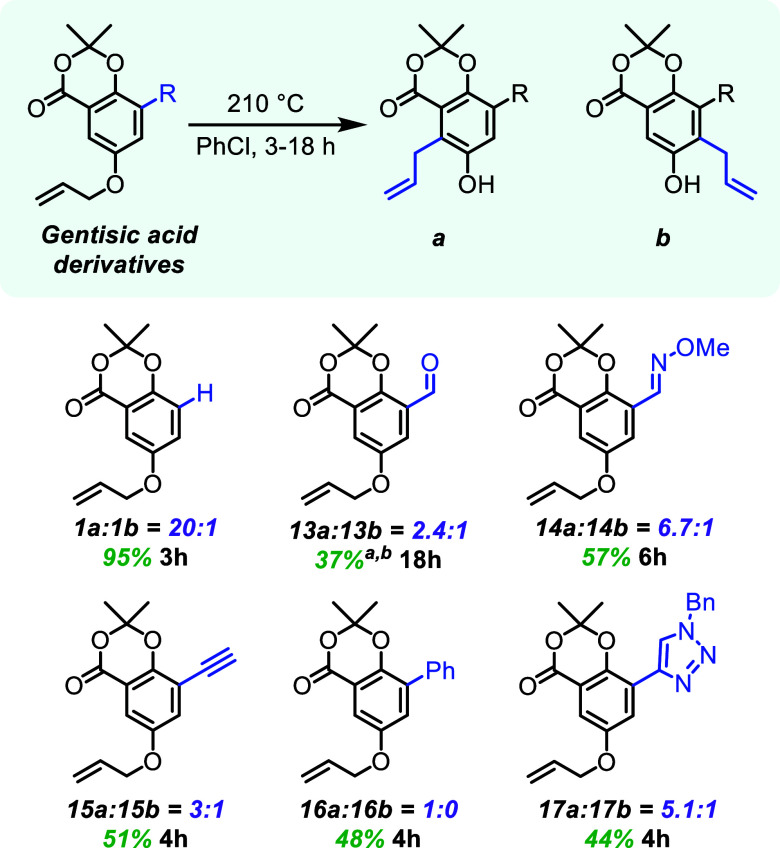
Gentisic acid derivatives with aromatic Claisen rearrangement yields and regioselectivities. Combined isolated yields of **a** and **b** reported for the aromatic Claisen rearrangement. ^*a*^ **13b** regioisomer isomerized the alkene into conjugation with the benzene ring, with an *E*/*Z* ratio of 94:6. ^*b*^180 °C.

The focus on the gentisic acid derivatives was to investigate the potential competitive effect of internal bases that may influence the regioselectivity. By having two different *meta*-substituents in the same molecule, the substrates synthesized could probe the impacts of electronic and steric differences adjacent to the migration positions on the aromatic ring. In all cases, the lactone carbonyl is rotationally locked into position to act as a base. This enables direct comparisons between the gentisic acid derivatives (**13–17**) to see which groups are more or less impactful on the regioselectivity of the reaction.

With a *meta*-aldehyde (**13**), which can act as a base, the regioselectivity still favors the position toward the lactone, but with greatly decreased selectivity (2.4:1). Unexpectedly, the minor product (**13b**) undergoes a subsequent isomerization to conjugate the olefin (*E*/*Z* 94:6) with the aromatic ring, a phenomenon unobserved for any alkylation adjacent to the lactone (products relating to structures **a**). Since there is rotation along the aryl-aldehyde bond, it is hypothesized that the aldehydic oxygen atom can rotate out of plane to isomerize the allylic group, whereas the lactone carbonyl oxygen cannot easily rotate out of planarity to deprotonate the benzylic proton and isomerize the alkene. With aldehyde **13** in hand, oxime **14** was made and resulted in a 6.7:1 ratio favoring the *ortho*-position toward the lactone carbonyl (**14a**). The oxime nitrogen is a stronger base than the lactone oxygen, however, the rotational nature of the oxime could be favored away from the acidic hydrogen for deprotonation. In contrast, the lactone oxygen is structurally locked in position, allowing for faster intramolecularlar deprotonation. The significant erosion of regioselectivity in the presence of competing bases, as well as the isomerization of **13b**, are strong evidence for the general hypothesis that intramolecular proton transfers can affect the product distributions of aromatic Claisen rearrangements.

The remaining three substrates (**15–17**) investigate the potential effects of exocyclic conjugation at the *meta*-position, which should stabilize the core arene. These systems were accessed through Suzuki–Miyaura coupling, Sonogashira coupling, and CuAAC click reactions. The alkyne substrate (**15**) was less regioselective compared to the parent system (**1**), with a ratio of 3:1. The biphenyl substrate (**16**) had complete regioselectivity, with the only product observed having reacted adjacent to the lactone (**16a**). By incorporating a triazole (**17**), the regioselectivity still favored the contrasteric product (**17a**) in a 5.1:1 ratio. It Is hypothesized again that the triazole’s nitrogen atoms are rotated away from the aromatic proton, as compared to the rotationally locked carbonyl in the lactone ring.

### Tetralin Bicycle Derivatives

Using the simplified 7-allyloxy tetralin scaffold, three subsets of substrates were investigated to further interogate the regioselectivity of aromatic Claisen rearrangements (Figure 4). The first subset (**18–20**) lacks an internal heteroatom base and provides the inherent regioselectivity for the tetralin system. The second and third groups feature oxygen (**21–23**) and nitrogen (**24–27**) derived bases, respectively. All of these classes have structural rigidity provided by the tetralin ring system to elucidate the potential role of rotationally rigid basic sites.^33–38^

**Figure 4:**
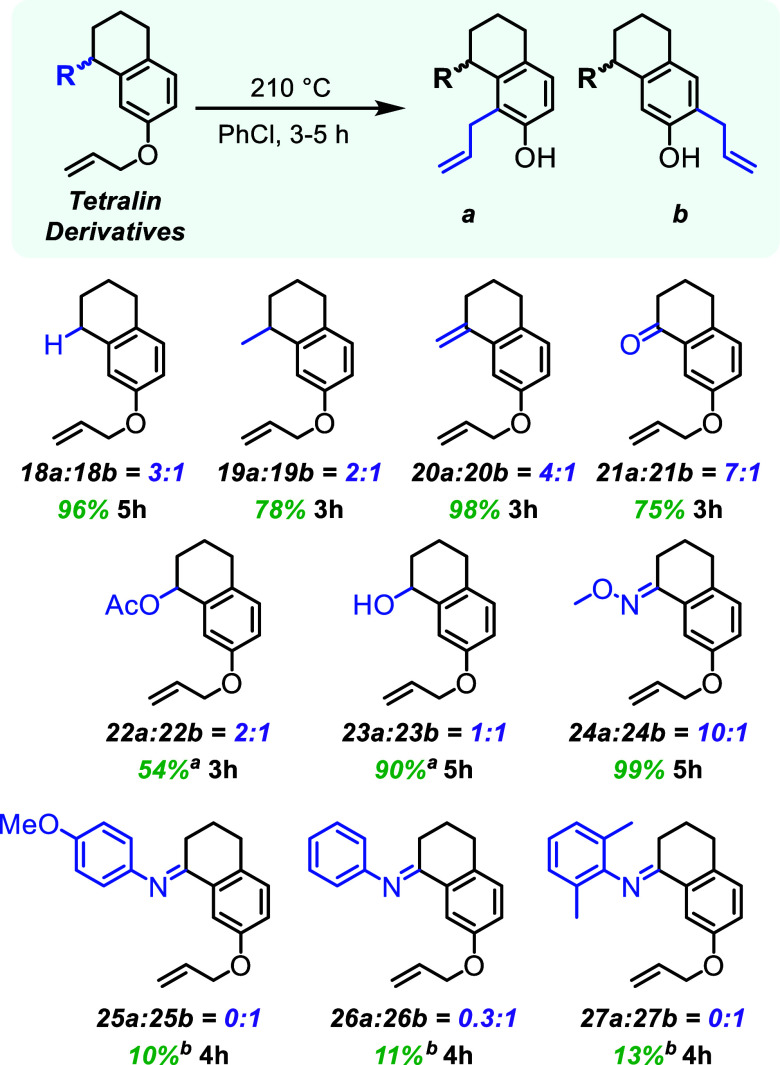
Tetralin derivatives with aromatic Claisen rearrangement yields and regioselectivities. ^1^H NMR was used to determine regioselectivities before structural elucidation. Combined isolated yields of **a** and **b** reported for the aromatic Claisen rearrangement. ^*a*^ **22** and **23** generated an alkene in conjugation with the aromatic ring after the rearrangement via an elimination of acetic acid and water, respectively. ^*b*^Rearranged products were hydrolyzed to the ketones.

The substrates without internal bases (**18–20**) still produced the contrasteric regioisomer as the major product, although with modest regioselectivity. Tetralin **18**, the methyl analogue (**19**), and the vinyl analogue (**20**) reacted efficiently in the aromatic Claisen rearrangement with 3:1, 2:1, and 4:1 regioselectivity ratios, respectively. Collectively, these regioselectivities show a slight preference with the tetralin system to react at the position adjacent to the second ring. It is hypothesized that this slight intrinsic preference could be due to conformational preference in forming the two different hexalins.

Ketone **21** contains a fixed internal base and its regioselectivity is expectedly higher (7:1) than substrates without a base. For substrates where the ketone was reduced to an alcohol (**23**) or functionalized as an acetoxy group (**22**), the regioselectivity is negligible to minor. This is potentially due to the basic oxygen atom being spatially out of the plane, compared to ketone **21**. Unexpectedly, the alcohol and acetoxy group eliminated under the conditions of the reaction, but it was determined that the rearrangement was faster than the elimination process. The oxime (**24**) resulted in a highly selective contrasteric process with a 10:1 regioselectivity ratio favoring **24a**. The heightened basicity of the oxime, in comparison to the others (**21–23**) due to the α-heteroatom effect, likely makes the group a more efficient directing group.

The last group of tetralin derivatives are the N-substituted imines (**25–27**). The regioselectivity of each imine substrate was determined from the hydrolyzed products (**21a** and **21b**), as the imine products converted to the ketones on workup and purification. In each case, the imines favored rearrangement toward the less sterically encumbered position. The N-*para*-anisyl imine (**25**) presented total regioselectivity toward the less sterically hindered position. Removing the methoxy group, imine **26** produced a 0.3:1 ratio of regioisomers. The *N*-phenyl imine with two methyl groups at the *ortho*-positions (**27**) was fully regioselective for the less sterically hindered isomer.

### *m*-Salicylamide Derivatives

The final class of allyloxy compounds investigated mild changes in basicity by testing a series of amides at the *meta*-position (Figure 5).^39–42^ These amides have differing levels of electron withdrawing and donating ability, that could affect the basicity of the carbonyl in directing the regioselectivity.

**Figure 5:**
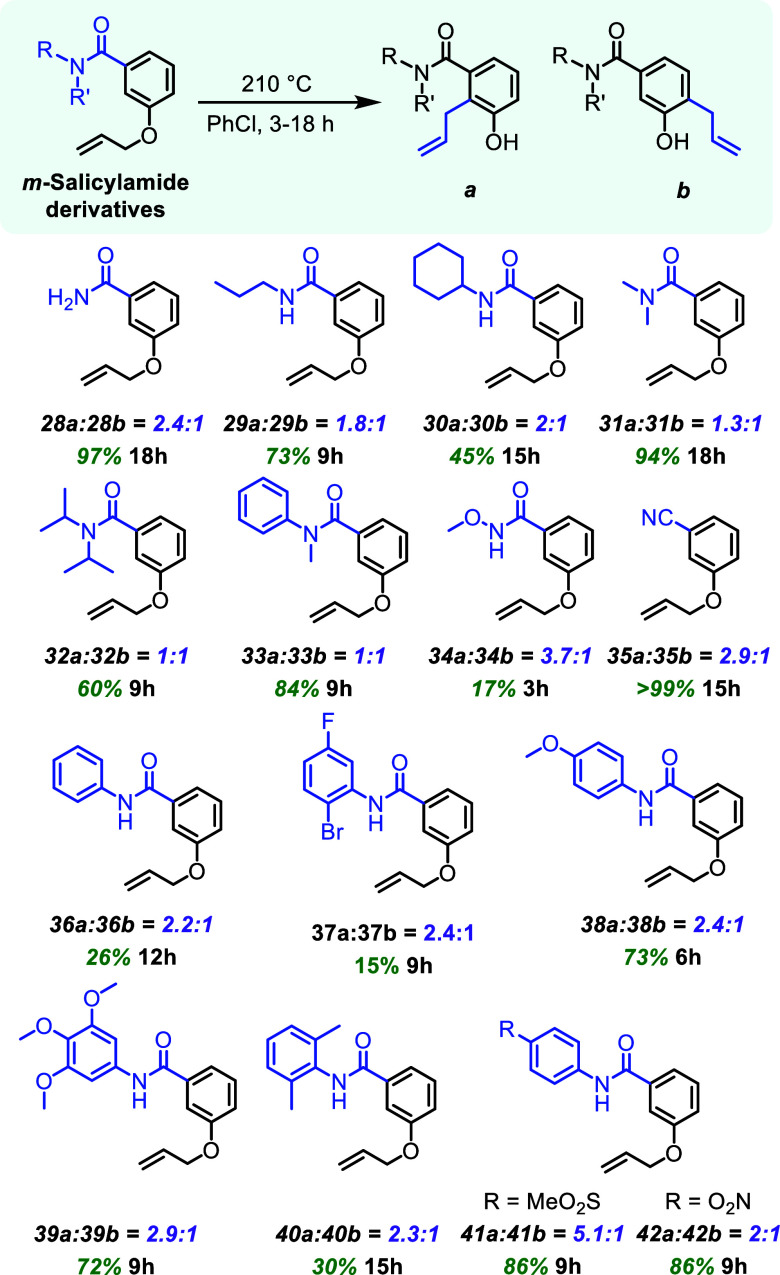
*m*-Salicylamide derivatives with aromatic Claisen rearrangement yields and regioselectivities. ^1^H NMR was used to determine regioselectivities before chromatographic isolation of products. Combined isolated yields of **a** and **b** reported for the aromatic Claisen rearrangement.

To begin, the primary amide (**28**) was found to have moderate selectivity (2.4:1) in favor of the contrasteric position. The aliphatic amides (**29** and **30**) had slight regioselectivity decreases to 1.8:1 and 2:1, respectively. Tertiary amides (**31**, **32**, and **33**) had limited to no regioselectivity at all with 1.3:1, 1:1 and 1:1 ratios, respectively. The Weinreb amide (**34**), which has increased basicity at the carbonyl, had a 3.7:1 ratio favoring the more sterically hindered isomer (**34a**). Benzonitrile **35** had a regioselectivity ratio of 2.9:1 toward the more sterically hindered position. This was unexpected since the nitrile is not positioned to deprotonate the requisite position. We hypothesize that the nitrile makes the contrasteric *ortho*-position much more acidic, potentially lowering the barrier of tautomerization to a degree where the [3,3] sigmatropic rearrangement step becomes the rate-determining step.

The arene electronics of N-arylated secondary salicylamides (**36–42**) were adjusted with electron withdrawing and donating groups. All these amides show that the major product is the contrasteric regioisomer with only minor differences in regioselectivity. The first aromatic amide (**36**), featuring an undecorated phenyl ring, produced a 2.2:1 ratio of regioisomers. The presence of electron donating groups, such as methoxy **38**, trimethoxy **39**, and dimethyl **40**, led to regioselectivity ratios of 2.1:1, 2.9:1, and 2.3:1, respectively. For substrates with electron withdrawing groups, amides **37**, **41**, and **42** still favored the more sterically hindered position, with regioselectivity ratios of 2.4:1, 5.1:1 and 2:1, respectively. Generally, the primary and secondary amides favor the more sterically hindered position and no regioisomeric preference was observed for tertiary amides. All of the amides studied were less regioselective than the parent compound (**1**), which is likely due to the rotational flexibility of the amides.

### Mechanistic Probe

To further understand and study the mechanism of the aromatic Claisen rearrangement, we investigated the reversibility of the [3,3]-sigmatropic rearrangement. Importantly, the initial step of the mechanism was determined to be reversible (Figure 6). Using ^1^H NMR experiments, it was shown over time that the *Z*-alkene of compound **8** isomerizes to the *E*-alkene (**7**) at a rate faster than its consumption. The best explanation for this observation is that the [3,3]-sigmatropic rearrangement is occurring and is reversible between the boat and chair transition states to change the alkene configuration from *Z* to the more stable *E* configuration. This evidence for reversibility led us to focus more on the tautomerization step.

**Figure 6:**
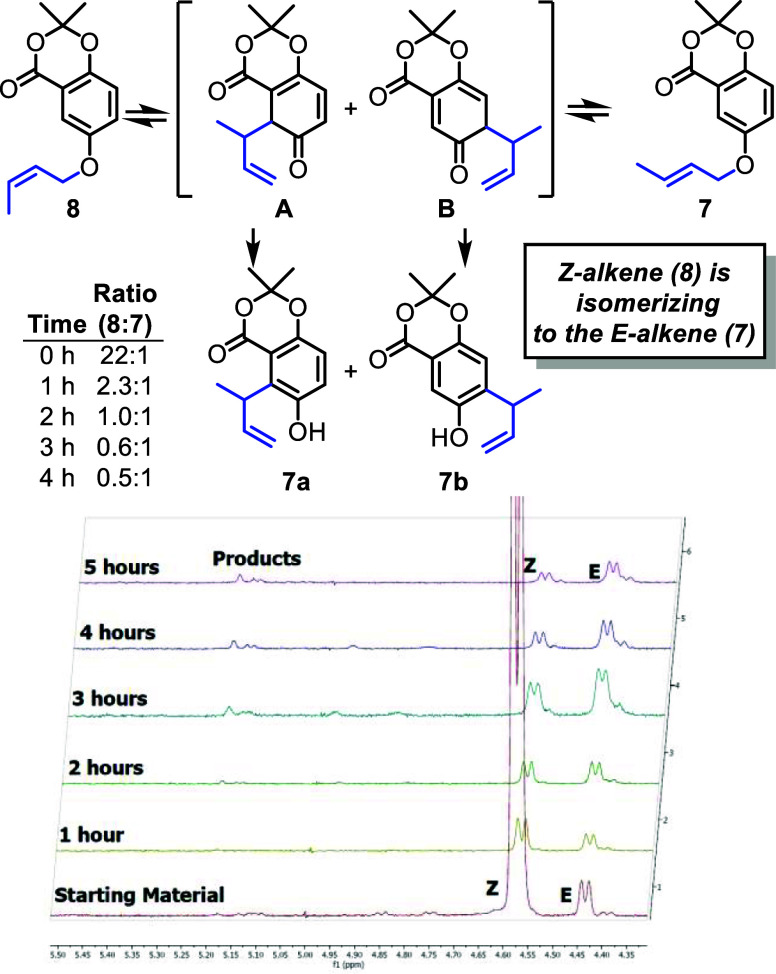
Evidence for reversible migration: alkene isomerization. The reaction was at 210 °C for the time shown. Aliquots of the reaction mixture were removed every hour, concentrated and ^1^H NMR was taken. The *Z*-alkene (**8**) has a distinct peak at 4.57 ppm, the *E*-alkene (**7**) has a peak at 4.44 ppm, and the products have peaks between 5.20 and 5.14 ppm. The table shows the quantified ratio of **8**:**7** based on integrations of the respective ^1^H NMRs.

To compare the basicity and regioselectivities of the *meta*-salicylamide derivatives, the percent selectivity for the more sterically hindered isomer was graphed versus the amide carbonyl carbon shifts from the ^13^C NMR (Chart 1). It was hypothesized that the more shielded amide carbonyl carbons would have more basic oxygen atoms, due to increased electron donation from the nitrogen atoms, creating more selectivity for the contrasteric position. This reasoning would be the same for the less shielded amide carbons having less basic oxygen atoms for decreased selectivity. These data show a correlation with the carbon shifts and regioselectivity in Chart 1, which aligns with the expected selectivities based on the proposed impact of a tethered base.

**Chart 1:**
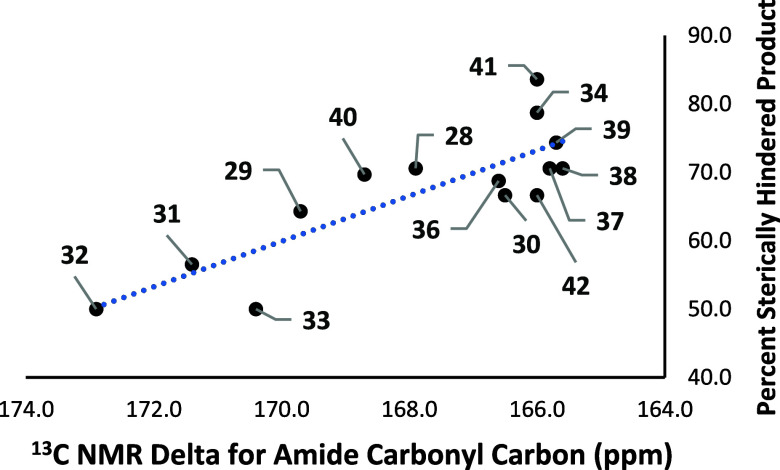
Comparison of ^13^C NMR with Regioselectivity of Benzamides

To validate the internal base-directed aromatic Claisen rearrangement in the gentisic acid system, a “push–pull” system was tested to determine whether electronics are the primary factor to drive the regioselectivity of the reaction. By having a nitrile group as an electron “pull”, and a methoxy group as the “push”, nitrile **43** was subjected to the aromatic Claisen rearrangement in dry chlorobenzene at 210 °C for 18 h (Figure 7). The bias of an internal base is not present, as the nitrile would not be able to reach the proton for deprotonation due to its hybridization. The result showed a 2.5:1 ratio of **43a** to **43b**. The regioselectivity of the “push–pull” system of **43** is very similar to that of nitrile **35** (Figure 5) and much less selective than the parent system (**1**; Figure 3). This supports the hypothesis that the regioselectivity of the rearrangement is minorly affected by the “push–pull” electronics and is aided primarily with a structurally locked internal base.

**Figure 7:**
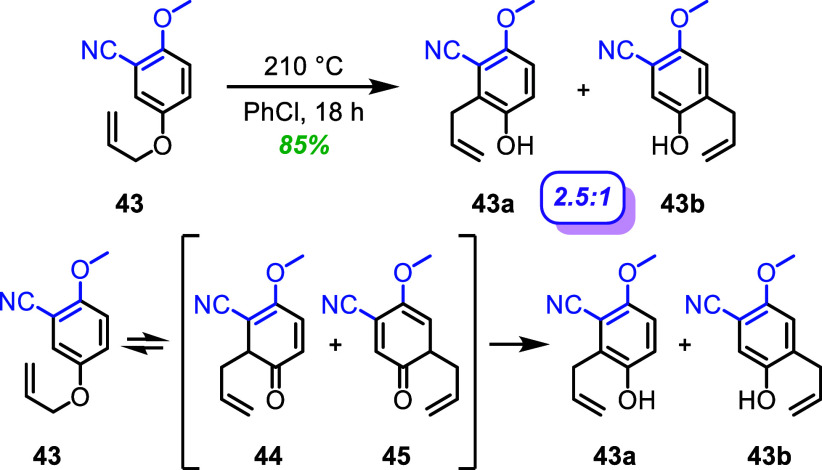
Aromatic-Claisen rearrangement of the push–pull system of nitrile **43**. ^1^H NMR was used to determine the regioselectivity before chromatographic isolation of **43a** and **43b**. Combined isolated yields of **43a** and **43b** reported for the aromatic Claisen rearrangement.

To study the effect of concentration on the regioselectivity of the aromatic Claisen rearrangement, multiple substrates were tested at varying concentrations between 1 mM and 1.0 M. No observable correlation was observed between regioselectivity and concentration, implying that the regioselectivity is not concentration dependent (see Supporting Information). Additionally, the regioselectivity of the reaction was monitored over the course of multiple reactions and there were only negligible changes in regioselectivity observed.

The sum of these mechanistic experiments indicated that the tautomerization step can be the rate-determining step, and that the inclusion of an intramolecular base can accordingly influence the regioselectivity of the aromatic Claisen rearrangement. Furthermore, a correlation was observed between the basicity of groups positioned near the aromatic hydrogen atom to be deprotonated and the regioisomeric outcome of the reaction. It was found that a structurally rigid system placing a basic atom in the plane of the arene is highly beneficial to selectively achieving the contrasteric product. To this end, the optimal structures examined were the gentisic acid derived lactones (based on **1**).

### Synthetic Application

The contrasteric aromatic Claisen rearrangement has utility toward the synthesis of biologically relevant molecules, due to its high regioselectivity in forming polysubstituted aromatic rings. Furthermore, the products of the most efficient and highly selective system (Figure 1) generated a densely functionalized 1,2,3,4-tetrasubstituted benzene ring with oxygen substitution to produce quinones and dihydroquinones.

Based on the clear value to this contrasteric and regioselective aromatic Claisen rearrangement, parent lactone **1a** was prepared on a gram-scale in three steps from gentisic acid (see Supporting Information). Phenol **1a** was treated with aqueous sulfuric acid in THF, to generate 5-hydroxymellein (**46**) in a single step (Scheme 2). Dihydrocourmarin **46** has interesting biological activities, including antiatherosclerotic, antibacterial, antifungal, and algicidal properties and is a potential multifunctional skin UV protectant.^43–46^ Using the same lactone (**1a**), Upjohn dihydroxylation and a base catalyzed lactonization in one flask forms botryoisocoumarin A (**47**), providing the first total synthesis of this natural product. Botryoisocoumarin A (**47**) has shown promising biological activity in the inhibition of the COX-2 enzyme, a therapeutic target which has shown promising activity for anti-inflammatory and anticancer properties.^47^

**Scheme 2:**
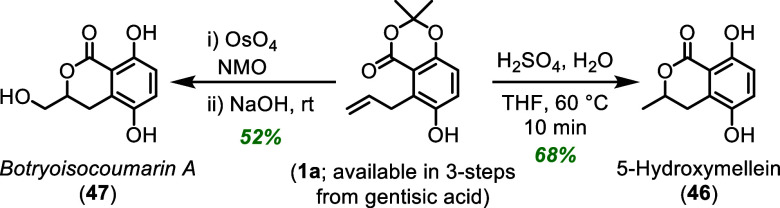
Total Syntheses of Dihydroisocoumarins Utilizing the Contrasteric Aromatic Claisen Rearrangement

## Conclusions

In summary, the aromatic Claisen rearrangement has been studied to determine how regioselectivity can be affected by different internal bases and allyl or propargyl groups of various aromatic systems. Unlike prior reports, the approach reported does not rely on a blocking group or aromatic stabilization to direct the regioselectivity. Mechanistic studies show that the migratory step is reversible and that there is a correlation between the positioning of the internal bases and regioselectivities observed, although the observed regioselectivities were modest at times and reliant on conformational rigidity. The analysis also showed that the regioselectivity is not dictated strongly by the push–pull system and instead correlates more strongly with a tethered base. By determining the various regioselectivities and yields, this work was useful for the efficient total synthesis of biologically relevant molecules and expands the utility of the aromatic Claisen rearrangement.

## Supplementary Material



## Data Availability

The data underlying this study are available in the published article and its Supporting Information.

## Notes

Experimental details, and ^1^H and ^13^C NMR spectra for all synthesized compounds (PDF)
