# Supplementary material for: Regioselectivity Study of Contrasteric Aromatic Claisen Rearrangements and Their Utilization in the Total Syntheses of 5‑Hydroxymellein and Botyroisocoumarin A
Source: J Org Chem. 2025 Sep 4;90(37):12956–63. doi: 10.1021/acs.joc.5c01268 (PMC12455646; doi:10.1021/acs.joc.5c01268)
Supplement: Supplementary file 1 [file jo5c01268_si_001.pdf]

Supporting Information for

Regioselectivity Study of Contrasteric Aromatic Claisen Rearrangements and Their Utilization in the Total Syntheses of 5-Hydroxymellein and Botyroisocoumarin A

David K. Tanas, Runzi Li, Harris H. Khan, Tangela C. Johnson, Logan E. Brown, Philip M. West, Emily J. Guin, Sydney E. Jones, Nolan K. Garci, Drew M. Canning, Emily J. Ramirez, Gurjant S. Sekhon, Tanner H. Pierce, Abraham Ustoyev, Mitchell P. Croatt*

Corresponding Author Email: mpcroatt@uncg.edu

Department of Chemistry and Biochemistry, University of North Carolina at Greensboro, Greensboro, NC, 27402, USA

Table of Contents Page

Experimental Section S2
General Information S2
Allylation of Phenols: General Procedure S3
Acetonide Formation: General Procedure S8
Synthesis of Imines: General Procedure S9
Amidations: General Procedure S11
Synthesis of Oximes: General Procedure S15
Lindlar's Reduction: General Procedure S16
Aromatic-Claisen Rearrangement: General Procedure S17
Synthesis of 2,2-Dimethyl-6-((2-methylbut-3-yn-2-yl)oxy)-4H-benzo[d][1,3] dioxin-4-one (**11**) S35
Synthesis of 6-(Buta-2,3-dien-1-yloxy)-2,2-dimethyl-4H-benzo[d][1,3]dioxin-4-one (**9**) S36
Synthesis of Synthesis of Buta-2,3-dien-1-ol (**S5**) S36
Synthesis of 3-Bromo-2,5-dihydroxybenzoic acid (**S6**) S36
Synthesis of 6-Hydroxy-2,2-dimethyl-8-phenyl-4H-benzo[d][1,3]dioxin-4-one (**S7**) S37
Synthesis of 5-(Allyloxy)-2-hydroxybenzoic acid (**S8**) S37
Synthesis of 5-(Allyloxy)-3-formyl-2-hydroxybenzoic acid (**S9**) S38
Synthesis of 6-hydroxy-2,2-dimethyl-8-((trimethylsilyl)ethynyl)-4H-benzo[d][1,3] dioxin-4-one (**S10**) S38
Synthesis of 8-Ethynyl-6-hydroxy-2,2-dimethyl-4H-benzo[d][1,3]dioxin-4-one (**S11**) S39
Synthesis of 8-(1-benzyl-1H-1,2,3-triazol-4-yl)-6-hydroxy-2,2-dimethyl-4H-benzo [d][1,3]dioxin-4-one (**S12**) S39
Synthesis of 7-Hydroxy-1-tetralone (**S13**) S39

Synthesis of 7-(Allyloxy)-1-methylene-1,2,3,4-tetrahydronaphthalene (**20**) S40
Synthesis of 7-(Benzyloxy)-3,4-dihydronaphthalen-1(2H)-one (**S14**) S40
Synthesis of 7-(Benzyloxy)-1-methylene-1,2,3,4-tetrahydronaphthalene (**S15**) S41
Synthesis of 8-Methyl-5,6,7,8-tetrahydronaphthalen-2-ol (**S16**) S41
Synthesis of 7-(Allyloxy)-1,2,3,4-tetrahydronaphthalen-1-ol (**23**) S41
Synthesis of 7-(Allyloxy)-1,2,3,4-tetrahydronaphthalen-1-yl acetate (**22**) S42
Synthesis of 3-(Allyloxy)benzoic acid (**S17**) S42
Synthesis of 3-(Allyloxy)benzamide (**28**) S43
Synthesis of 5-(2,3-dihydroxypropyl)-6-hydroxy-2,2-dimethyl-4H-benzo[d][1,3] dioxin-4-one (**S18**) S43
Synthesis of Botyroisocoumarin A (**47**) from diol **S18** S44
Synthesis of Botyroisocoumarin A (**47**) from lactone **1a** S44
Synthesis of 5-Hydroxymellein (**46**) S44
Synthesis of 5-Hydroxy-2-methoxybenzonitrile (**S19**) S45
^{1}H and ^{13}C NMR Spectra of Compounds S46
Concentration Studies of Lactone **1** and Salicylamide **38** S272

Experimental Section

General Information

All chemicals were of commercially available grade and used without further purification, unless noted otherwise. Commercial ACS grade solvents were used for extractions and chromatography. Air- and moisture-sensitive compounds were prepared and handled under nitrogen atmosphere in a Vacuum Atmospheres OMNI-Lab inert atmosphere glovebox. Unless noted otherwise, all reactions were carried out under N_2 atmosphere in oven-dried glassware at room temperature (25 °C). Reactions were monitored using thin-layer chromatography (TLC) on silica gel-coated plates (Silica gel 60 F_{254}). TLC plates were visualized with 254 nm UV light irradiation, or by $KMnO_4$ staining. Chromatographic purification was performed using silica gel (60 Å, 32-63 µm).

Nuclear Magnetic Resonance (NMR) spectra were recorded in $CDCl_3$, acetone-d_6, methanol-d_4, or DMSO-d_6 using a JEOL ECA 400 spectrometer (400 MHz for ^{1}H, 101 MHz for ^{13}C, and 376.5 MHz for ^{19}F), and JEOL ECA spectrometer (500 MHz for ^{1}H, 125 MHz for ^{13}C, and 470 MHz for ^{19}F). Coupling constants, J, are reported in hertz (Hz) and multiplicities are listed as singlet (s), doublet (d), triplet (t), quartet (q), quintet (quint), sextet (sext), septet (sept), doublet of doublets (dd), triplet of triplets (tt), triplet of septets (tsept), multiplet (m), etc. Chemical shifts (δ) are given in parts per million (ppm) and referenced against ^{1}H NMR solvent residual shifts (e.g., chloroform-d at δ = 7.26 ppm) and/or tetramethylsilane (TMS at δ = 0.00 ppm) and using the solvent for ^{13}C NMR (chloroform = 77.2 ppm, acetone = 29.1 ppm, methanol = 49.0 ppm, DMSO = 39.5 ppm). High-resolution mass spectrometry (HRMS) spectra were performed on a Thermo Fisher Scientific UPLC/LTQ Orbitrap XL system with an electrospray ionization source. The UPLC separations were achieved using an Acquity BEH C_{18} column (50 mm × 2.1 mm i.d., 1.7 µm) equilibrated at 40 °C and a flow rate of 0.3 mL/min.

The mobile phase comprised a linear gradient of CH_3CN/H_2O with 0.1% HCOOH starting at 15% CH_3CN to 100% over 8.5 min. Electrospray ionization mass spectra (ESI-MS) of the compounds were collected in positive ion mode by a Waters Synapt G2 HDMS q-TOF system.

Allylation of Phenols: General Procedure

Anhydrous potassium carbonate (2.32 mmol, 3.0 eq) was added to a flask with a magnetic stir bar and phenol (0.77 mmol, 1.0 eq) followed by the addition of sodium iodide (10-40 mol%*). The resulting mixture was dissolved in dry acetone (9 mL, 0.09 M) before addition of the allylic or propargylic bromide (3.86 mmol, 5 eq). The reaction mixture was heated to 50 °C with either an oil bath or heating mantle and left to stir until completion, as monitored by thin layer chromatography. Upon reaction completion, the crude mixture was cooled to ambient temperature, filtered via vacuum filtration, and the solids washed with ethyl acetate. The filtrate was collected and washed twice with DI water, before subsequent drying with brine and anhydrous sodium sulfate. The mixture was filtered, concentrated via reduced pressure, and purified via silica gel column chromatography to yield the aryl allyl and propargyl ethers.

*= Increase in catalyst amount shortened reaction time to completion without a detriment to yields.

6-(Allyloxy)-2,2-dimethyl-4H-benzo[d][1,3]dioxin-4-one (1)

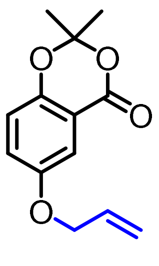

1

Following the general procedure, **1** was observed in quantitative yield as an orange oil without any purification (180 mg, quantitative).

^{1}H NMR (400 MHz, $CDCl_3$) δ_H 7.36 (1H, d, J = 2.7 Hz), 7.11 (1H, dd, J = 9.0, 3.2 Hz), 6.85 (1H, d, J = 9.1 Hz), 5.99 (1H, ddt, J_d = 16.0, 10.5 Hz, J_t = 5.4 Hz), 5.37 (1H, d, J = 17.2 Hz), 5.25 (1H, d, J = 10.3 Hz), 4.49 (2H, d, J = 5.3 Hz), 1.67 (6H, s) ppm.

^{13}C{^{1}H} NMR (101 MHz, $CDCl_3$) δ 161.4, 153.9, 150.4, 132.8, 125.5, 118.5, 118.3, 113.7, 112.2, 106.6, 69.6, 25.8 (2C) ppm.

R_f = 0.53 (20% ethyl acetate in hexane, visualized by UV lamp).

HRMS = (ESI) *m/z*: $[M+H]^+$ calculated for $C_{14}H_{15}O_4$ 235.0965; Found 235.0972.

2,2-Dimethyl-6-((2-methylallyl)oxy)-4H-benzo[d][1,3]dioxin-4-one (4)

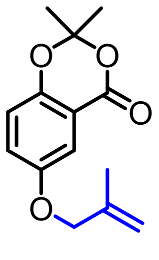

4

Following the general procedure, **4** was purified through isocratic column chromatography at 15% ethyl acetate in hexane to yield a light-yellow oil (213 mg, 92%).

^{1}H NMR (400 MHz, $CDCl_3$) δ_H 7.41 (1H, d, J = 3.0 Hz), 7.17 (1H, dd, J = 9.1, 3.1 Hz), 6.89 (1H, d, J = 9.1 Hz), 5.09 (1H, s), 5.00 (1H, s), 4.43 (2H, s), 1.83 (3H, s), 1.72 (6H, s) ppm.

^{13}C{^{1}H} NMR (101 MHz, $CDCl_3$) δ 161.5, 154.0, 150.3, 140.4, 125.4, 118.4, 113.8, 113.2, 112.4, 106.5, 72.5, 25.8 (2C), 19.5 ppm.

R_f = 0.57 (25% ethyl acetate in hexane, visualized by UV lamp).

HRMS = (ESI) *m/z*: $[M+H]^+$ calculated for $C_{14}H_{17}O_4$ 249.1121; Found 249.1118.

2,2-Dimethyl-6-((3-methylbut-2-en-1-yl)oxy)-4H-benzo[d][1,3]dioxin-4-one (5)

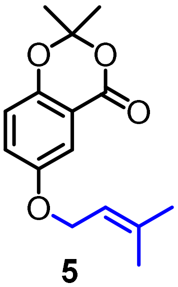

5

Following the general procedure, **5** was purified through gradient column chromatography from 10% to 50 ethyl acetate in hexane to yield a light-yellow oil (541.1 mg, 87%).

^{1}H NMR (500 MHz, $CDCl_3$) δ_H 7.14 (1H, d, J = 3.1 Hz), 7.14 (1H, dd, J = 8.8, 3.2 Hz), 6.88 (1H, d, J = 8.8 Hz), 5.48 (1H, t-sep, J_t = 6.7 Hz, J_{sept} = 1.6 Hz), 4.50 (2H, d, J = 6.8 Hz), 1.80 (3H, d, J = 1.2 Hz), 1.75 (3H, d, J = 1.3 Hz), 1.72 (6H, s) ppm.

^{13}C{^{1}H} NMR (125 MHz, $CDCl_3$) δ 161.5, 154.1, 150.2, 138.9, 125.6, 119.1, 118.4, 113.6, 111.8, 106.5, 65.6, 25.9, 25.8 (2C), 18.3 ppm.

R_f = 0.80 (30% ethyl acetate in hexane, visualized by UV lamp).

HRMS = (ESI) *m/z*: $[M+H]^+$ calculated for $C_{15}H_{19}O_4$ 263.1278; Found 263.1274.

(*E*)-6-(But-2-en-1-yloxy)-2,2-dimethyl-4H-benzo[d][1,3]dioxin-4-one (7)

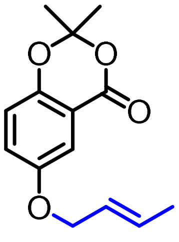

7

Following the general procedure, **7** was observed as a red oil in a 3:1 ratio of *Z* to *E* alkene isomers (541.1 mg, 87%).

^{1}H NMR (400 MHz, $CDCl_3$) δ_H 7.40 (1H, d, J = 3.2 Hz), 7.14 (1H, dd, J = 8.8, 3.3 Hz), 6.88 (1H, d, J = 9.0 Hz), 5.92-5.82 (1H, m), 5.75-5.66 (1H, m), 4.45 (2H, dt, J_d = 6.2 Hz, J_t = 1.1 Hz), 1.76 (3H, dq, J_d = 6.5 Hz, J_q = 1.1 Hz), 1.72 (6H, s) ppm.

^{13}C{^{1}H} NMR (101 MHz, $CDCl_3$) δ 161.4, 154.0, 150.2, 131.2, 126.6, 125.5, 118.4, 113.7, 112.0, 160.5, 69.5, 25.8 (2C), 18.0 ppm.

R_f = 0.71 (30% ethyl acetate in hexane, visualized by UV lamp).

HRMS = (ESI) *m/z*: $[M+H]^+$ calculated for $C_{14}H_{17}O_4$ 249.1121; Found 249.1117.

2,2-Dimethyl-6-(prop-2-yn-1-yloxy)-4H-benzo[d][1,3]dioxin-4-one (10)

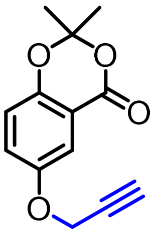

10

Following the general procedure, **10** was purified through gradient column chromatography from 10% to 50 ethyl acetate in hexane to yield product as a yellow solid (192.4 mg, 64%).

^{1}H NMR (500 MHz, $CDCl_3$) δ_H 7.50 (1H, d, J = 3.1 Hz), 7.21 (1H, dd, J = 9.1, 3.2 Hz), 6.92 (1H, d, J = 9.1 Hz), 4.70 (2H, d, J = 2.5 Hz), 2.55 (1H, t, J = 2.4 Hz), 1.72 (6H, s) ppm.

^{13}C{^{1}H} NMR (125 MHz, ($CDCl_3$) δ 161.3, 152.8, 150.9, 125.6, 118.6, 113.8, 112.9, 106.6, 78.0, 76.3, 56.6, 25.8 (2C) ppm.

R_f = 0.50 (30% ethyl acetate in hexane, visualized by UV lamp).

HRMS = (ESI) *m/z*: $[M+H]^+$ calculated for $C_{13}H_{13}O_4$ 233.0808; Found 233.0805.

6-(But-2-yn-1-yloxy)-2,2-dimethyl-4H-benzo[d][1,3]dioxin-4-one (12)

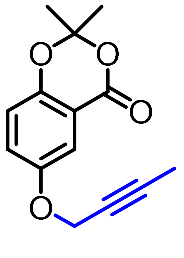

12

Following the general procedure, **12** was purified through gradient column chromatography from 5% to 20 ethyl acetate in hexane to yield product as a clear oil (444 mg, 88%).

^{1}H NMR (400 MHz, $CDCl_3$) δ_H 7.48 (1H, d, J = 3.2 Hz), 7.19 (1H, dd, J = 9.0, 3.1 Hz), 6.90 (1H, d, J = 9.0 Hz), 4.65 (2H, q, J = 2.3 Hz), 1.87 (3H, t, J = 2.3 Hz), 1.72 (6H, s) ppm.

^{13}C{^{1}H} NMR (101 MHz, ($CDCl_3$) δ 161.3, 153.1, 150.6, 125.5, 118.5, 113.7, 112.7, 106.5, 84.5, 73.5, 57.2, 25.8 (2C), 3.8 ppm.

R_f = 0.48 (20% ethyl acetate in hexane, visualized by UV lamp).

HRMS = (ESI) *m/z*: $[M+H]^+$ calculated for $C_{14}H_{15}O_4$ 247.0965; Found 247.0962.

6-(Allyloxy)-8-bromo-2,2-dimethyl-4H-benzo[d][1,3]dioxin-4-one (S1)

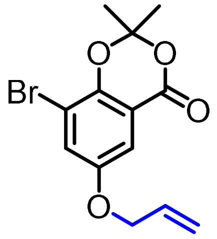

S1

Following the general procedure, **S1** was purified through gradient column chromatography from 0% to 15% ethyl acetate in hexane to yield a clear oil (228 mg, 99%).

^{1}H NMR (500 MHz, $CDCl_3$) δ_H 7.40 (1H, s), 7.40 (1H, s), 6.01 (1H, ddt, J_d = 16.5, 10.3 Hz, J_t = 5.3 Hz), 5.42 (1H, dd, J = 17.4, 1.4 Hz), 5.32 (1H, dd, J = 10.9, 1.2 Hz), 4.53 (2H, dt, J_d = 5.3 Hz, J_t = 1.9 Hz), 1.76 (6H, s).

^{13}C{^{1}H} NMR (101 MHz, $CDCl_3$) δ 160.6, 153.8, 1475, 132.4, 128.1, 118.5, 114.8, 112.4, 111.4, 107.3, 69.8, 25.9 (2C) ppm.

R_f = 0.69 (25% ethyl acetate in hexane, visualized by UV lamp).

HRMS = (ESI) *m/z*: $[M+H]^+$ calculated for $C_{13}H_{14}BrO_4$ 313.0070; Found 313.0080.

6-(Allyloxy)-8-ethynyl-2,2-dimethyl-4H-benzo[d][1,3]dioxin-4-one (15)

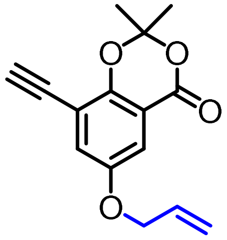

15

Following the general procedure, **15** was purified through gradient column chromatography from 0% to 10% ethyl acetate in hexane to yield a clear oil (9.8 mg, 79%).

^{1}H NMR (400 MHz, $CDCl_3$) δ_H 7.45 (1H, d, J = 3.0 Hz), 7.29 (1H, d, J = 3.3 Hz), 6.02 (1H, ddt, J_d = 16.3, 10.5 Hz, J_t = 5.3 Hz), 5.41 (1H, d, J = 17.2 Hz), 5.31 (1H, d, J = 10.5 Hz), 4.53 (2H, d, J = 5.3 Hz), 3.30 (1H, s), 1.76 (6H, s) ppm.

^{13}C{^{1}H} NMR (125 MHz, $CDCl_3$) δ 160.7, 153.1, 151.3, 132.5, 128.2, 118.4, 114.2 (2C), 113.0, 107.1, 83.0, 77.2, 69.7, 25.9 (2C), ppm.

R_f = 0.33 (15% ethyl acetate in hexane, visualized by UV lamp).

HRMS = (ESI) *m/z*: $[M+H]^+$ calculated for $C_{15}H_{15}O_4$ 259.0965; Found 259.0969.

6-(Allyloxy)-2,2-dimethyl-8-phenyl-4H-benzo[d][1,3]dioxin-4-one (16)

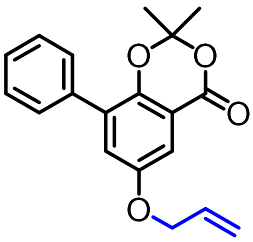

16

Following the general procedure, **16** was purified through gradient column chromatography from 5% to 10% ethyl acetate in hexane to yield a clear oil (76.5 mg, 77%).

^{1}H NMR (500 MHz, $CDCl_3$) δ_H 7.51-7.36 (6H, m), 7.25 (1H, d, J = 3.1 Hz), 6.06 (1H, ddt, J_d = 17.2, 10.5 Hz, J_t = 5.3 Hz), 5.44 (1H, dq, J_d = 17.3 Hz, J_q = 1.6 Hz), 5.32 (1H, dq, J_d = 10.5 Hz, J_q = 1.4 Hz), 4.58 (2H, dt, J_d = 5.3 Hz, J_t = 1.5 Hz), 1.72 (6H, s) ppm.

^{13}C{^{1}H} NMR (125 MHz, $CDCl_3$) δ 161.6, 153.6, 147.4, 135.7, 132.8, 132.2, 129.2 (2C), 128.5 (2C), 128.1, 125.9, 118.3, 114.6, 111.7, 106.4, 69.6, 25.9 (2C) ppm.

R_f = 0.56 (20% ethyl acetate in hexane, visualized by UV lamp).

HRMS = (ESI) *m/z*: $[M+H]^+$ calculated for $C_{19}H_{19}O_4$ 311.1278; Found 311.1287.

6-(Allyloxy)-8-(1-benzyl-1H-1,2,3-triazol-4-yl)-2,2-dimethyl-4H-benzo[d][1,3]dioxin-4-one (17)

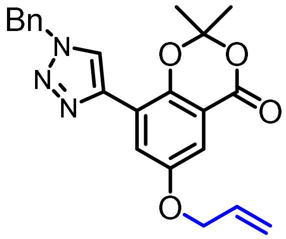

17

Following the general procedure, **17** was purified through gradient column chromatography from 20% to 60% ethyl acetate in hexane to yield **17** as a white solid (26.8 mg, 44%).

^{1}H NMR (400 MHz, Acetone-d_6) δ_H 8.51 (1H, s), 8.13 (1H, d, J = 3.1 Hz), 7.43-7.31 (6H, m), 6.12 (1H, ddt, J_d = 17.1, 10.6 Hz, J_t = 5.6 Hz), 5.71 (2H, s), 5.48 (1H, dq, J_d = 17.2 Hz, J_q = 1.7 Hz), 5.29 (1H, dq, J_d = 10.8 Hz, J_q = 1.5 Hz), 4.70 (2H, dt, J_d = 5.1 Hz, J_t = 1.4 Hz), 1.75 (6H, s) ppm.

^{13}C{^{1}H} NMR (101 MHz, Acetone-d_6) δ 160.7, 154.7, 146.9, 141.3, 137.0, 134.3, 129.7 (2C), 129.1, 128.7 (2C), 124.9, 122.6, 121.1, 117.7, 115.7, 113.3, 107.3, 70.0, 54.2, 25.8 (2C) ppm.

R_f = 0.69 (50 % ethyl acetate in hexane, visualized by UV lamp).

HRMS = (ESI) *m/z*: $[M+H]^+$ calculated for $C_{22}H_{22}N_3O_4$ 392.1605; Found 392.1599.

6-(Allyloxy)-1,2,3,4-tetrahydronaphthalene (18)

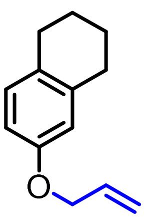

18

Following the general procedure, **18** was obtained as a yellow oil without any purification (119 mg, 95%).

^{1}H NMR (400 MHz, $CDCl_3$) δ_H 6.95 (1H, d, J = 8.4 Hz), 6.67 (1H, dd, J = 8.3, 2.5 Hz), 6.61 (1H, d, J = 2.3 Hz), 6.04 (1H, ddt, J_d = 17.2, 10.6 Hz, J_t= 5.4 Hz), 5.39 (1H, ddt, J_d = 17.1, 4.5 Hz, J_t = 1.6 Hz), 5.25 (1H, ddt, J_d = 10.4, 4.3 Hz, J_t = 1.3 Hz), 4.49 (2H, dt, J_d = 5.3 Hz, J_t = 1.4 Hz), 2.70 (4H, dt, J_d = 15.9 Hz, J_t= 5.4 Hz), 1.76 (4H, m) ppm.

^{13}C{^{1}H} NMR (101 MHz, $CDCl_3$) δ 156.4, 138.3, 133.7, 130.0, 129.6, 117.5, 114.7, 112.6, 68.9, 29.8, 28.7, 23.5, 23.2 ppm.

R_f = 0.88 (20% ethyl acetate in hexane, visualized by UV lamp).

HRMS = (ESI) *m/z*: $[M+H]^+$ calculated for $C_{13}H_{17}O$ 189.1274; Found 189.1269.

7-(Allyloxy)-1-methyl-1,2,3,4-tetrahydronaphthalene (19)

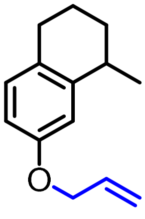

19

Following the general procedure, **19** was purified through isocratic column chromatography at 10% ethyl acetate in hexane to yield a yellow oil (361 mg, 89%).

^{1}H NMR (400 MHz, $CDCl_3$) δ_H 6.94 (1H, d, J = 8.5 Hz), 6.76 (1H, d, J = 2.8 Hz), 6.67 (1H, dd, J = 8.2, 2.7 Hz), 6.05 (1H, ddt, J_d = 17.2, 10.3 Hz, J_t = 5.3 Hz), 5.40 (1H, ddt, J_d = 16.3, 4.8 Hz, J_t = 1.7 Hz), 5.26 (1H, ddt, J_d = 10.5, 4.1 Hz, J_t = 1.4 Hz), 4.40 (2H, dt, J_d = 5.4, J_t =1.5 Hz), 2.86 (1H, dt, J_d = 14.1 Hz, J_t = 6.5 Hz), 2.76-2.60 (2H, m), 1.95-1.78 (2H, m), 1.74-1.63 (1H, m), 1.54-1.45 (1H, m), 1.26 (3H, d, J = 7.4 Hz) ppm.

^{13}C{^{1}H} NMR (101 MHz, $CDCl_3$) δ 156.7, 143.5, 133.8, 129.9, 129.4, 117.6, 114.3, 112.3, 69.0, 32.9, 31.6, 29.3, 22.9, 20.8 ppm.

R_f = 0.83 (20% ethyl acetate in hexane, visualized by UV lamp).

HRMS = (ESI) *m/z*: $[M+H]^+$ calculated for $C_{14}H_{19}O$ 203.1430; Found 203.1439.

7-(Allyloxy)-3,4-dihydronaphthalen-1(2H)-one (21)

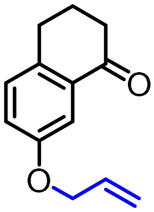

21

Following the general procedure, **21** was obtained as a yellow oil without any purification (519 mg, 99%).

^{1}H NMR (400 MHz, $CDCl_3$) δ_H 7.52 (1H, d, J = 2.7 Hz), 7.17 (1H, d, J = 8.4 Hz), 7.08 (1H, dd, J = 8.6, 2.7 Hz), 6.05 (1H, ddt, J_d = 16.4, 10.6 Hz, J_t = 5.4 Hz), 5.42 (1H, d, J = 17.6 Hz), 5.29 (1H, d, J = 11.2 Hz), 4.57 (2H, d, J = 5.5 Hz), 2.90 (2H, t, J = 6.0 Hz), 2.63 (2H, t, J = 6.0 Hz), 2.12 (2H, quint, J = 6.6 Hz) ppm.

^{13}C{^{1}H} NMR (101 MHz, $CDCl_3$) δ 198.5, 157.4, 137.4, 133.5, 133.1, 130.2, 122.4, 118.0, 110.2, 69.1, 39.1, 29.0, 23.6 ppm.

R_f = 0.49 (20% ethyl acetate in hexanes, visualized by UV lamp).

HRMS = (ESI) *m/z*: $[M+H]^+$ calculated for $C_{13}H_{15}O_2$ 203.1067; Found 203.1062.

Methyl 3-(Allyloxy)benzoate (S2)

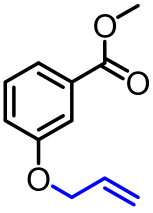

S2

Following the general procedure, methyl ester (**S2**) was observed as a clear oil without any purification (1.2 g, 99%).

^{1}H NMR (500 MHz, $CDCl_3$) δ_H 7.63 (1H, dt, J_d = 7.8 Hz, J_t = 1.1 Hz), 7.56 (1H, dd, J = 2.7 Hz, J = 1.4 Hz), 7.33 (1H, t, J = 7.9 Hz), 7.11 (1H, dd, J = 8.2, 2.7 Hz), 6.05 (1H, ddt, J_d = 17.1, 10.5 Hz, J_t = 5.3 Hz), 5.42 (1H, dq, J_d = 17.3 Hz, J_q = 1.6 Hz), 5.30 (1H, dq, J_d = 10.5 Hz, J_q = 1.3 Hz), 4.57 (2H, dt, J_d = 5.3 Hz, J_t = 1.5 Hz), 3.90 (3H, s) ppm, confirmed with the literature source *Eur. J. Org. Chem.* **2019**, *46,* 7568-7577.

3-(Allyloxy)benzonitrile (35)

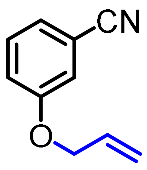

35

Following the general procedure, **35** was obtained as an off-white liquid without any purification (668 mg, quant.).

^{1}H NMR (500 MHz, $CDCl_3$) δ_H 7.38-7.33 (1H, m), 7.23 (1H, dt, J_d = 7.4 Hz, J_t = 1.2 Hz), 7.15-7.12 (2H, m), 6.02 (1H, ddt, J_d = 17.3, 10.5 Hz, J_t = 5.2 Hz), 5.41 (1H, dq, J_d = 17.4 Hz, J_q = 1.5 Hz), 5.32 (1H, dq, J_d = 10.6 Hz, J_q = 1.3 Hz), 4.55 (2H, dt, J_d = 5.2 Hz, J_t = 1.6 Hz) ppm, matches with literature source *Heterocycles* **2003**, *59*, 237-243.

5-(allyloxy)-2-methoxybenzonitrile (43)

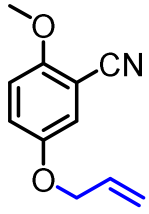

43

Following the general procedure, **43** was purified through gradient column chromatography from 0% to 20% ethyl acetate in hexane to yield **43** as an off-white liquid (723 mg, 96%).

^{1}H NMR (400 MHz, $CDCl_3$) δ_H 7.11 (1H, dd, J = 9.7, 3.0 Hz), 7.08-7.04 (1H, m), 6.89 (1H, d, J = 9.2 Hz), 6.01 (1H, ddt, J_d = 16.2, 10.5 Hz, J_t = 5.2 Hz), 5.40 (1H, d, J = 18.0 Hz). 5.31 (1H, d, J = 9.6 Hz), 4.49 (2H, d, J = 5.0 Hz), 3.88 (3H, s) ppm.

^{13}C{^{1}H} NMR (101 MHz, $CDCl_3$) δ 156.0, 152.2, 132.7, 121.8, 118.8, 118.3, 116.5, 112.6, 101.9, 69.8, 56.5 ppm.

R_f = 0.44 (25% ethyl acetate in hexane, visualized by $KMnO_4$ stain).

HRMS = (ESI) *m/z*: $[M+H]^+$ calculated for $C_{11}H_{12}NO_2$ 190.0863; Found 190.0863.

Acetonide Formation: General Procedure

To a stirred solution of gentisic acid (32.4 mmol, 1 eq) in trifluoroacetic acid (TFA, 0.79 M) at 0 °C, trifluoroacetic anhydride (TFAA, 227 mmol), and dry acetone (227 mmol) were added slowly into an oven dried round bottom flask, equipped with a magnetic stir bar. The solution was warmed up to room temperature and immediately fitted with a condenser and allowed to react for 24 hours at 90 °C using an oil bath or heating mantle. Once completed, the reaction mixture was concentrated under vacuum to remove excess TFA. The resultant dark colored solid was dissolved in ethyl acetate and washed with water four times, $NaHCO_3$ once, and brine once. The organic layer was then dried with Na_2SO_4, and further concentrated down under vacuum. The resulting impure lactone was purified using silica gel column chromatography. **Note:** TFA and TFAA are strongly acidic and should be handled with care. After removal of the residual TFA, all glassware of the system removing the TFA (i.e., rotary evaporator) should be rinsed to eliminate dangers to researchers using the system afterwards.

6-Hydroxy-2,2-dimethyl-4H-benzo[d][1,3]dioxin-4-one (S3)

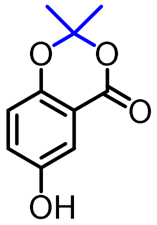

S3

Following the general procedure, lactone (**S3**) was purified through gradient column chromatography from 0% to 35% ethyl acetate in hexane to yield a light yellow crystalline solid (3.2 g, 51%).

^{1}H NMR (400 MHz, $CDCl_3$) δ_H 7.38 (1H, d, *J* = 3.1 Hz), 7.08 (1H, dd, *J*= 8.7, 3.2 Hz), 6.88 (1H, d, *J* = 8.8 Hz), 4.86 (1H, s), 1.72 (6H, s) ppm, confirmed with the literature source of *Int. J. Mol. Sci.* **2022**, *23*, 3927-3944.

8-Bromo-6-hydroxy-2,2-dimethyl-4H-benzo[d][1,3]dioxin-4-one (S4)

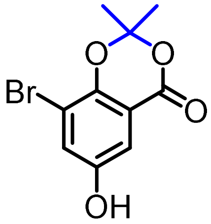

S4

Following the general procedure, lactone (**S4**) was purified through gradient column chromatography from 0% to 35% ethyl acetate in hexane to yield an off-white solid (1.1 g, 48%).

^{1}H NMR (400 MHz, $CDCl_3$) δ_H 7.37 (1H, d, *J* = 2.8 Hz), 7.34 (1H, d, *J* = 2.8 Hz), 1.75 (6H, s) ppm, O-H signal not observed.

^{13}C{^{1}H} NMR (101 MHz, $CDCl_3$) δ 161.2, 151.5, 147.2, 127.8, 114.8, 114.4, 111.4, 107.5, 25.8 (2C) ppm.

R_f = 0.34 (20% ethyl acetate in hexane, visualized by UV lamp).

HRMS = (ESI) *m/z*: $[M+H]^+$ calculated for $C_{10}H_{10}BrO_4$ 272.9757; Found 272.9753.

6-(Allyloxy)-2,2-dimethyl-4-oxo-4H-benzo[d][1,3]dioxine-8-carbaldehyde (13)

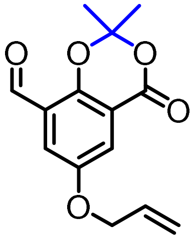

13

Following the general procedure, **13** was purified through gradient column chromatography from 0% to 25% ethyl acetate in hexane to yield a red solid (87 mg, 32%).

^{1}H NMR (400 MHz, $CDCl_3$) δ_H 10.30 (1H, s), 7.72 (1H, d, *J* = 3.2 Hz), 7.61 (1H, d, *J* = 3.2 Hz), 6.00 (1H, ddt, J_d = 16.0, 10.5 Hz, J_t = 5.1 Hz), 5.41 (1H, d, *J* = 17.3 Hz), 5.31 (1H, d, *J* = 10.5 Hz), 4.56 (2H, d, *J* = 5.0 Hz), 1.79 (6H, s) ppm.

^{13}C{^{1}H} NMR (125 MHz, $CDCl_3$) δ 186.8, 159.9, 153.6, 152.0, 132.2, 125.6, 121.3, 120.7, 118.5, 115.5, 107.4, 69.70, 26.0 (2C) ppm.

R_f = 0.40 (25% ethyl acetate in hexane, visualized by UV lamp).

HRMS = (ESI) *m/z*: $[M+H]^+$ calculated for $C_{14}H_{15}O_5$ 263.0914; Found 263.0922.

Synthesis of Imines: General Procedure

In an oven-dried round-bottomed flask equipped with a magnetic stir bar, 0.4 to 2.0 mmol of ketone (1 equiv.) and the appropriate amine starting material (5 equiv.) were dissolved in 2 to 10 mL of dry toluene. Activated 5 Å molecular sieves (60% w/v with respect to the toluene solvent) were then added, and the flask was sealed with a rubber septum and heated at 105 °C in an oil bath for approximately 16 h under positive N_2 atmosphere. Reaction progression was monitored using basic alumina gel TLC. The reaction mixture was quantitatively transferred using 1% v/v triethylamine:EtOAc and filtered through a thick pad of celite and sand

over a coarse glass frit, then evaporated under reduced pressure. Purification was performed using a silica gel column chromatography pretreated using 1%-5% v/v triethylamine solution (eluent: 1%-5% v/v triethylamine in 0%-10% EtOAc:hexanes, isocratic or gradient) to yield the imine as a red, orange, or yellow oil.

(*E*)-7-(Allyloxy)-N-(4-methoxyphenyl)-3,4-dihydronaphthalen-1(2H)-imine (25)

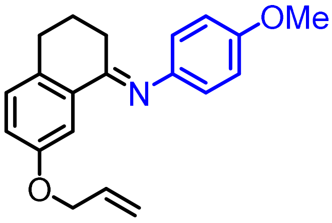

25

Following the general procedure, **25** was obtained as a red oil (114 mg, 62%).

^{1}H NMR (400 MHz, $CDCl_3$) δ_H 7.85 (1H, d, J = 2.6 Hz), 7.10 (1H, d, J = 8.4 Hz), 6.98 (1H, dd, J = 8.4, 2.7 Hz), 6.90 (2H, d, J = 8.8 Hz), 6.75 (2H, d, J = 8.7 Hz), 6.07 (1H, ddt, J_d = 16.2, 10.5 Hz, J_t = 5.3 Hz), 5.43 (1H, dd, J = 17.4, 1.8 Hz), 5.34 – 5.23 (1H, m), 4.60 (2H, d, J = 5.3 Hz), 3.82 (3H, s), 2.83 (2H, t, J = 6.1 Hz), 2.53 (2H, t, J = 6.3 Hz), 1.88 (2H, quint, J = 6.3 Hz) ppm.

^{13}C{^{1}H} NMR (101 MHz, $CDCl_3$) δ 166.0, 157.3, 155.9, 144.8, 135.0, 134.1, 133.5, 130.0, 121.0 (2C), 119.5, 117.7, 114.4 (2C), 109.6, 69.1, 55.6, 29.9, 29.3, 23.4 ppm.

R_f = 0.34 (silica gel TLC, 1% triethyl amine in 10% ethyl acetate in hexane, visualized by UV lamp), 0.60 (basic alumina TLC, 10% ethyl acetate in hexane).

HRMS = (ESI) *m/z*: $[M+H]^+$ calculated for $C_{20}H_{22}NO_2$ 308.1645; Found 308.1647.

(*E*)-7-(Allyloxy)-N-phenyl-3,4-dihydronaphthalen-1(2H)-imine (26)

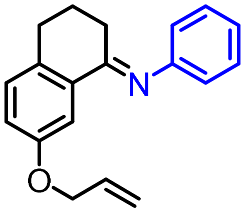

26

Following the general procedure, **26** was obtained as an orange-red oil (128 mg, 80%).

^{1}H NMR (400 MHz, $CDCl_3$) δ_H 7.85 (1H, d, J = 2.7 Hz), 7.34 (2H, t, J = 7.7 Hz), 7.11 (1H, d, J = 8.5 Hz), 7.07 (1H, t, J = 7.5 Hz), 7.00 (1H, dd, J = 8.4, 2.8 Hz), 6.80 (2H, d, J = 7.7 Hz), 6.08 (1H, ddt, J_d = 16.0, 10.6 Hz, J_t = 5.4 Hz), 5.43 (1H, d, J = 17.4 Hz), 5.28 (1H, d, J = 10.6 Hz), 4.60 (2H, d, J = 5.4 Hz), 2.83 (2H, t, J = 6.0 Hz), 2.49 (2H, t, J = 6.3 Hz), 1.89 (2H, quint, J = 6.2 Hz,) ppm.

^{13}C{^{1}H} NMR (101 MHz, $CDCl_3$) δ 165.7, 157.3, 151.8, 134.8, 134.3, 133.4, 130.0, 129.1 (2C), 123.2, 119.7, 119.6 (2C), 117.8, 109.7, 69.1, 29.9, 29.3, 23.3 ppm.

R_f = 0.62 (silica gel TLC, 10% ethyl acetate in hexane, visualized by UV lamp).

HRMS = (ESI) *m/z*: $[M+H]^+$ calculated for $C_{19}H_{20}NO$ 278.1545; Found 278.1543.

(*E*)-7-(Allyloxy)-N-(2,6-dimethylphenyl)-3,4-dihydronaphthalen-1(2H)-imine (27)

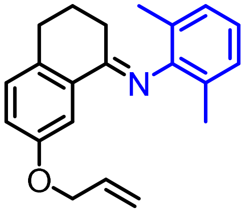

27

Following the general procedure, **27** was obtained as a yellow-orange oil (37 mg, 20%).

^{1}H NMR (400 MHz, $CDCl_3$) δ_H 8.00 (1H, d, J = 2.7 Hz), 7.15 (1H, d, J = 8.5 Hz), 7.09 – 6.99 (3H, m), 6.96 – 6.89 (1H, m), 6.09 (1H, ddt, J_d = 16.3, 10.6 Hz, J_t = 5.3 Hz), 5.46 (1H, d, J = 17.4 Hz), 5.30 (1H, d, J = 10.4 Hz), 4.63 (2H, d, J = 5.3 Hz), 2.86 (2H, t, J = 6.1 Hz), 2.25 (2H, t, J = 6.4 Hz), 2.04 (6H, s), 1.90 (2H, quint, J = 6.2 Hz) ppm.

$^{13}C\{^1H\}$ **NMR** (101 MHz, $CDCl_3$) δ 165.0, 157.2, 149.1, 134.5, 134.0, 133.4, 130.0, 127.9 (2C), 125.8, 122.7, 119.4, 117.8, 110.0, 69.1, 30.0, 29.3, 23.1, 18.2 (2C) ppm. One carbon signal listed represents two carbons.

$\mathbf{R_f}$ = 0.54 (10% ethyl acetate in hexane, visualized by UV lamp), 0.92 (basic alumina TLC, 10% ethyl acetate in hexane).

HRMS = (ESI) *m/z*: $[M+H]^+$ calculated for $C_{21}H_{24}NO$ 306.1858; Found 306.1853.

Amidations: General Procedure

To a solution of **S17** (1.12 mmol, 1 eq) in dry dichloromethane (4 mL, 0.28 M) was added oxalyl chloride (1.35 mmol, 1.2 eq) at 0 °C under nitrogen atmosphere followed by the addition of dry DMF (2 drops). The reaction mixture stirred for four hours at room temperature. The solvent was evaporated off and the crude acid chloride was used in the next step. Dimethylamine hydrochloride (1.35 mmol, 1.2 eq) was added to a biphasic mixture of K_2CO_3 (2.25 mmol, 2 eq) in ethyl acetate and water. The mixture cooled to 0 °C, whereupon crude acid chloride was added dropwise as a solution in ethyl acetate (5 mL). The reaction mixture allowed to warm to room temperature overnight. After separation of the organic layer, the aqueous layer extracted twice more with ethyl acetate. The combined organic fractions dried with sodium sulfate, then concentrated under vacuum. The crude material was purified using silica gel column chromatography to yield the amides.

3-(Allyloxy)-N-methoxybenzamide (34)

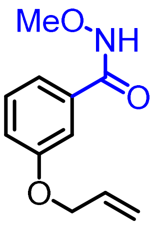

34

Following the general procedure, purification with a gradient of 15% to 100% ethyl acetate in hexane yielded **34** as a light-yellow oil (213 mg, 92%).

^{1}**H NMR** (400 MHz, $CDCl_3$) δ_H 7.35 – 7.29 (2H, m), 7.24 (1H, t, J = 1.2 Hz), 7.07 (1H, dt, J_d = 8.2 Hz, J_t = 1.1 Hz), 6.04 (1H, ddt, J_d = 17.2, 10.5 Hz, J_t = 5.3 Hz), 5.41 (1H, dd, J = 17.3, 1.5 Hz), 5.30 (1H, dd, J = 10.6, 1.4 Hz), 4.57 (2H, dt, J_d = 5.3 Hz, J_t = 1.5 Hz), 3.88 (3H, s) ppm, N-H signal not observed.

$^{13}C\{^1H\}$ **NMR** (101 MHz, $CDCl_3$) δ 166.0, 158.5, 132.9, 132.7, 129.5, 119.5, 118.8, 117.7, 113.0, 68.7, 63.9 ppm.

$\mathbf{R_f}$ = 0.4 (66% ethyl acetate in hexane, visualized by UV lamp).

HRMS = (ESI) *m/z*: $[M+H]^+$ calculated for $C_{11}H_{14}NO_3$ 208.0974; Found 208.0968.

3-(Allyloxy)-N-propylbenzamide (29)

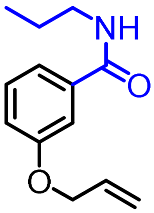

29

Following the general procedure, **29** was obtained as a yellow oil without any purification (228 mg, 93%).

^{1}**H NMR** (400 MHz, MeOD) δ_H 7.41 – 7.33 (2H, m), 7.27 (1H, t, J = 8.1 Hz), 7.04 – 6.97 (1H, m), 5.99 (1H, ddt, J_d = 17.2, 10.2 Hz, J_t = 4.9 Hz), 5.35 (1H, br dd, J = 17.3, 1.4 Hz), 5.19 (1H, br dd, J = 10.6, 1.3 Hz), 4.50 (2H, br d, J = 5.1 Hz), 3.28 (2H, m), 1.58 (2H, sext, J = 7.4 Hz), 0.91 (3H, t, J = 7.4 Hz) ppm, N-H signal not observed.

^{13}C{^{1}H} NMR (101 MHz, MeOD) δ 169.7, 160.0, 137.1, 134.5, 130.5, 120.4, 118.9, 117.6, 114.4, 69.7, 42.7, 23.6, 11.8 ppm.

R$_f$ = 0.5 (33% ethyl acetate in hexane, visualized by UV lamp).

HRMS = (ESI) *m/z*: $[M+H]^+$ calculated for $C_{13}H_{18}NO_2$ 220.1338; Found 220.1331.

3-(Allyloxy)-N-cyclohexylbenzamide (30)

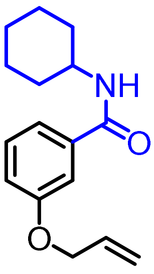

30

Following the general procedure, purification with a gradient of 15% to 100% ethyl acetate in hexane yielded **30** as a white solid (137 mg, 47%).

^{1}H NMR (400 MHz, $CDCl_3$) δ_H 7.35 (1H, t, J = 2.1 Hz), 7.31 (1H, d, J = 7.8 Hz), 7.27 – 7.24 (1H, m), 7.03 (1H, dd, J = 8.1, 2.6 Hz), 6.06 (1H, ddt, J_d = 17.2, 10.6 Hz, J_t = 5.3 Hz), 5.93 (1H, br d, J = 7.7 Hz), 5.43 (1H, dq, J_d = 17.3 Hz, J_q = 1.6 Hz), 5.31 (1H, dq, J_d = 10.5 Hz, J_q = 1.4 Hz), 4.58 (2H, dt, J_d = 5.3 Hz, J_t = 1.4 Hz), 4.02 – 3.91 (1H, m), 2.07 – 1.98 (2H, m), 1.75 (2H, dt, J_d = 13.2 Hz, J_t = 3.7 Hz), 1.65 (1H, dt, J_d = 12.9 Hz, J_t = 3.5 Hz), 1.49 – 1.36 (2H, m), 1.28 – 1.14 (3H, m) ppm.

^{13}C{^{1}H} NMR (101 MHz, $CDCl_3$) δ 166.5, 158.7, 136.6, 133.0, 129.4, 119.1, 118.0, 117.8, 113.3, 68.8, 48.9, 33.1 (2C), 25.6, 25.1 (2C) ppm

R$_f$ = 0.6 (50% ethyl acetate in hexane, visualized by UV lamp).

HRMS = (ESI) *m/z*: $[M+H]^+$ calculated for $C_{16}H_{22}NO_2$ 260.1651; Found 260.1647.

3-(Allyloxy)-N,N-dimethylbenzamide (31)

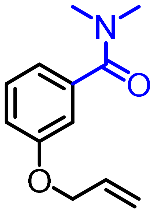

31

Following the general procedure, purification with a gradient of 0% to 40% ethyl acetate in hexane yielded **31** as an off-white oil (158 mg, 69%).

^{1}H NMR (500 MHz, $CDCl_3$) δ_H 7.23 (1H, td, J_t = 7.3, J_d = 1.4 Hz), 6.91 (3H, m), 5.98 (1H, ddt, J_d = 17.3, 10.6 Hz, J_t = 5.3 Hz), 5.35 (1H, dq, J_d = 17.2 Hz, J_q = 1.6 Hz), 5.23 (1H, dq, J_d = 10.5 Hz, J_q = 1.4 Hz), 4.49 (2H, dt, J_d = 5.3 Hz, J_t = 1.6 Hz), 3.04 (3H, br s), 2.91 (3H, br s) ppm.

^{13}C{^{1}H} NMR (125 MHz, $CDCl_3$) δ 171.4, 158.5, 137.6, 133.0, 129.5, 119.4, 117.9, 116.2, 113.2, 68.9, 39.6, 35.3 ppm.

R$_f$ = 0.22 (33% ethyl acetate in hexane, visualized by UV lamp).

HRMS = (ESI) *m/z*: $[M+H]^+$ calculated for $C_{12}H_{16}NO_2$ 206.1176; Found 206.1177.

3-(Allyloxy)-N,N-diisopropylbenzamide (32)

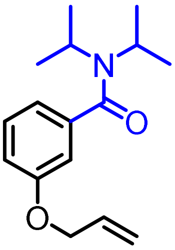

32

Following the general procedure, purification of 10% ethyl acetate in hexane yielded **32** as a clear oil (199 mg, 68%).

^{1}H NMR (400 MHz, MeOD) δ_H δ 7.35 (1H, t, J = 7.9 Hz), 7.00 (1H, dd, J = 8.3, 2.2 Hz), 6.89 – 6.82 (2H, m), 6.06 (1H, ddt, J_d = 17.3, 10.4 Hz, J_t = 5.1 Hz), 5.40 (1H, br dd, J = 17.4, 1.3 Hz), 5.26 (1H, br dd, J = 10.6, 1.3 Hz), 4.58 (2H, br d, J = 5.1 Hz), 3.73 (2H, br d, J = 87.1 Hz), 1.52 (6H, br s), 1.17 (6H, br s) ppm.

^{13}C{^{1}H} NMR (101 MHz, MeOD) δ 172.9, 160.1, 140.8, 134.5, 131.0, 118.6, 117.7, 116.4, 112.8, 69.7, 52.6, 47.1, 20.8 (4C) ppm.

R$_f$ = 0.6 (33% ethyl acetate in hexane, visualized by UV lamp).

HRMS = (ESI) *m/z*: $[M+H]^+$ calculated for $C_{16}H_{24}NO_2$ 262.1808; Found 262.1799.

3-(Allyloxy)-N-methyl-N-phenylbenzamide (33)

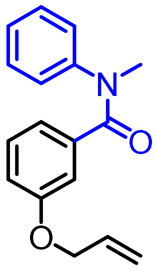

33

Following the general procedure, purification of 15% ethyl acetate in hexane yielded **33** as a yellow oil (230 mg, 77%).

^{1}H NMR (400 MHz, $CDCl_3$) δ_H 7.21 (2H, t, J = 7.6 Hz), 7.12 (1H, t, J = 7.4 Hz), 7.06 – 6.99 (3H, m), 6.87 – 6.81 (2H, m), 6.77 (1H, dd, J = 8.2, 1.8 Hz), 5.92 (1H, ddt, J_d = 17.3, 10.5 Hz, J_t = 5.2 Hz), 5.30 (1H, br dd, J = 17.4, 1.4 Hz), 5.21 (1H, br dd, J = 10.5, 1.3 Hz), 4.33 (2H, br d, J = 5.2 Hz), 3.47 (3H, s) ppm.

^{13}C{^{1}H} NMR (101 MHz, $CDCl_3$) δ 170.5, 158.0, 145.0, 137.2, 133.0, 129.3 (2C), 128.9, 126.9 (2C), 126.6, 121.5, 117.8, 117.0, 114.6, 68.7, 38.4 ppm.

R$_f$ = 0.5 (66% ethyl acetate in hexane, visualized by UV lamp).

HRMS = (ESI) *m/z*: $[M+H]^+$ calculated for $C_{17}H_{18}NO_2$ 268.1338; Found 268.1333.

3-(Allyloxy)-N-(2-bromo-5-fluorophenyl)benzamide (37)

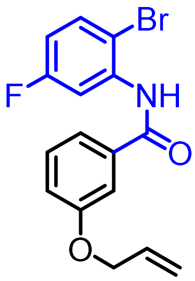

37

Following the general procedure, purification of 10% ethyl acetate in hexane yielded **37** as a yellow oil (121 mg, 31%).

^{1}H NMR (400 MHz, Acetone-d_6) δ_H 8.94 (1H, s), 8.20 – 8.08 (1H, m), 7.70 (1H, dd, J = 9.0, 5.9 Hz), 7.63 – 7.53 (2H, m), 7.48 (1H, t, J = 8.0 Hz), 7.22 (1H, dd, J = 8.2, 2.5 Hz), 6.96 (1H, td, J_t = 8.3 Hz, J_d = 3.1 Hz), 6.10 (1H, ddt, J_d = 15.9, 10.4 Hz, J_t = 5.1 Hz), 5.46 (1H, br dd, J = 17.4, 1.4 Hz), 5.28 (1H, br dd, J = 10.6, 1.3 Hz), 4.68 (2H, br d, J = 4.9 Hz) ppm.

^{13}C{^{1}H} NMR (101 MHz, Acetone-d_6) δ 165.8, 165.7, 162.8 (d, J_{C-F} = 245.2 Hz), 160.0, 138.6 (d, J_{C-F} = 10.9 Hz), 138.5 (d, J_{C-F} = 10.9 Hz), 136.6 (d, J_{C-F} = 3.4 Hz), 134.4 (d, J_{C-F} = 9.6 Hz), 130.9, 120.3, 119.7, 117.9, 114.4, 113.8 (d, J_{C-F} = 3.5 Hz), 113.6 (d, J_{C-F} = 3.4 Hz), 111.6 (d, J_{C-F} = 28.5 Hz), 111.5 (d, J_{C-F} = 28.5 Hz), 110.6 (d, J_{C-F} = 3.6 Hz), 110.4 (d, J_{C-F} = 3.5 Hz), 69.55 ppm. Extra carbon peaks were observed due to rotamers.

^{19}F NMR (376.5 MHz, $CDCl_3$) δ -113.8 ppm.

R$_f$ = 0.7 (20% ethyl acetate in hexane, visualized by UV lamp).

HRMS = (ESI) *m/z*: $[M+H]^+$ calculated for $C_{16}H_{14}BrFNO_2$ 350.0187; Found 350.0188.

3-(Allyloxy)-N-phenylbenzamide (36)

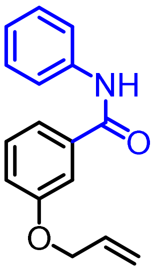

36

Following the general procedure, purification of 15% ethyl acetate in hexane yielded **36** as an orange solid (236 mg, 83%).

^{1}H NMR (500 MHz, $CDCl_3$) δ_H 7.77 (1H, s), 7.64 (2H, dd, J = 8.6, 1.3 Hz), 7.45 (1H, m), 7.40 – 7.39 (2H, m), 7.38 (1H, d, J = 1.1 Hz), 7.37 (1H, s), 7.16 (1H, tt, J = 7.7, 1.1 Hz), 7.12 – 7.08 (1H, m), 6.07 (1H, ddt, J_d = 17.3, 10.6 Hz, J_t = 5.3 Hz), 5.45 (1H, dq, J_d = 17.2 Hz, J_q = 1.6 Hz), 5.32 (1H, dq, J_d = 10.6 Hz, J_q = 1.4 Hz), 4.61 (2H, dt, J_d = 5.3 Hz, J_t = 1.5 Hz) ppm.

^{13}C{^{1}H} NMR (101 MHz, $CDCl_3$) δ 166.3, 158.5, 138.0, 136.1, 132.7, 129.4, 128.7 (2C), 124.4, 120.7 (2C), 119.3, 118.5, 117.6, 113.0, 68.5 ppm.

R_f = 0.7 (50% ethyl acetate in hexane, visualized by UV lamp).

HRMS = (ESI) *m/z*: $[M+H]^+$ calculated for $C_{16}H_{16}NO_2$ 254.1181; Found 254.1176.

3-(Allyloxy)-N-(4-methoxyphenyl)benzamide (3)

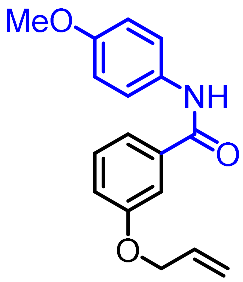

38

Following the general procedure, purification with 20% ethyl acetate in hexane yielded **38** as a light brown solid (302 mg, 95%).

^{1}H NMR (400 MHz, $CDCl_3$) δ_H 7.91 (1H, s), 7.53 (2H, d, J = 8.8 Hz), 7.42 (1H, s), 7.39 – 7.30 (2H, m), 7.09 – 7.04 (1H, m), 6.88 (2H, d, J = 8.9 Hz), 6.04 (1H, ddt, J_d = 15.9, 10.5 Hz, J_t = 5.3 Hz), 5.42 (1H, dq, J_d = 17.2 Hz, J_q = 1.3 Hz), 5.30 (1H, dq, J_d = 10.5 Hz, J_q = 1.3 Hz), 4.56 (2H, br d, J = 5.2 Hz), 3.80 (3H, s) ppm.

^{13}C{^{1}H} NMR (101 MHz, $CDCl_3$) δ 165.6, 159.0, 156.7, 136.6, 132.9, 131.1, 129.8, 122.2 (2C), 119.0, 118.6, 118.1, 114.3 (2C), 113.4, 69.0, 55.6 ppm.

R_f = 0.8 (66% ethyl acetate in hexane, visualized by UV lamp).

HRMS = (ESI) *m/z*: $[M+H]^+$ calculated for $C_{17}H_{18}NO_3$ 284.1287; Found 284.1282.

3-(Allyloxy)-N-(3,4,5-trimethoxyphenyl)benzamide (39)

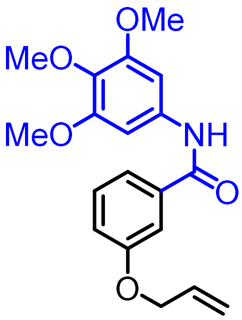

39

Following the general procedure, purification with 25% ethyl acetate in hexane yielded **39** as a white solid (301 mg, 78%).

^{1}H NMR (400 MHz, $CDCl_3$) δ_H 8.00 (1H, s), 7.44 (1H, br s), 7.41 – 7.31 (2H, m), 7.10 – 7.04 (1H, m), 6.96 (2H, s), 6.04 (1H, ddt, J_d = 15.8, 10.5 Hz, J_t = 5.3 Hz), 5.41 (1H, br dd, J = 17.2, 1.3 Hz), 5.29 (1H, br dd, J = 10.6, 1.2 Hz), 4.56 (2H, br d, J = 5.2 Hz), 3.82 (9H, br s) ppm.

^{13}C{^{1}H} NMR (101 MHz, $CDCl_3$) δ 165.7, 159.0, 153.4 (2C), 136.3, 134.8, 134.2, 132.8, 129.9, 118.9, 118.8, 118.1, 113.4, 97.9 (2C), 69.0, 61.1, 56.1 (2C) ppm.

R_f = 0.6 (33% ethyl acetate in hexane, visualized by UV lamp).

HRMS = (ESI) *m/z*: $[M+H]^+$ calculated for $C_{19}H_{22}NO_5$ 344.1499; Found 344.1486.

3-(Allyloxy)-N-(2,6-dimethylphenyl)benzamide (40)

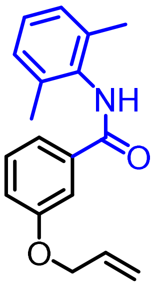

40

Following the general procedure, purification with 5% ethyl acetate in hexane yielded **40** as a tan oil (256 mg, 81%).

^{1}H NMR (400 MHz, MeOD) δ_H 7.58 – 7.51 (2H, m), 7.37 (1H, t, J = 7.9 Hz), 7.15 – 7.02 (4H, m), 6.03 (1H, ddt, J_d = 17.8, 10.4 Hz, J_t = 5.1 Hz), 5.39 (1H, br dd, J = 17.5, 1.4 Hz), 5.23 (1H, br dd, J = 10.6, 1.3 Hz), 4.55 (2H, br d, J = 5.1 Hz), 2.20 (6H, s) ppm, N-H signal not observed.

^{13}C{^{1}H} NMR (101 MHz, MeOD) δ 168.7, 160.2, 137.1, 136.7, 135.7, 134.5, 130.8, 129.1, 128.4, 120.8, 119.5, 117.7, 114.8, 69.8, 18.4 (2C) ppm. One carbon signal listed represents two carbons.

R$_f$ = 0.3 (20% ethyl acetate in hexane, visualized by UV lamp).

HRMS = (ESI) *m/z*: $[M+H]^+$ calculated for $C_{18}H_{20}NO_2$ 282.1495; Found 282.1482.

3-(Allyloxy)-N-(4-(methylsulfonyl)phenyl)benzamide (41)

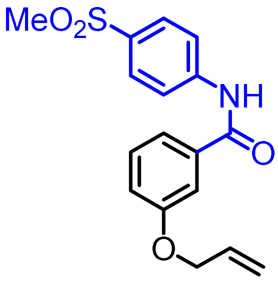

41

Following the general procedure, purification with 50% ethyl acetate in hexane yielded **41** as a white solid (90 mg, 24%).

^{1}H NMR (400 MHz, $CDCl_3$) δ_H 8.36 (1H, s), 7.92 – 7.80 (4H, m), 7.48 – 7.34 (3H, m), 7.11 (1H, br d, J = 7.8 Hz), 6.04 (1H, ddt, J_d = 16.0, 10.5 Hz, J_t = 5.2 Hz), 5.43 (1H, br d, J = 17.3 Hz), 5.31 (1H, br d, J = 10.5 Hz), 4.59 (3H, br d, J = 5.1 Hz), 3.04 (3H, s) ppm.

^{13}C{^{1}H} NMR (101 MHz, $CDCl_3$) δ 166.0, 159.1, 143.1, 135.6, 135.4, 132.7, 130.1, 128.8 (2C), 120.2 (2C), 119.3 (2C), 118.3, 113.7, 69.1, 44.8 ppm.

R$_f$ = 0.6 (66% ethyl acetate in hexane, visualized by UV lamp).

HRMS = (ESI) *m/z*: $[M+H]^+$ calculated for $C_{17}H_{18}NO_4S$ 332.0957; Found 332.0951.

3-(Allyloxy)-N-(4-nitrophenyl)benzamide (42)

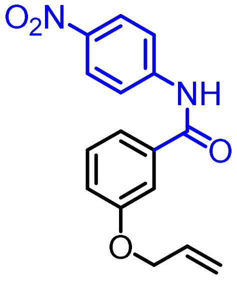

42

Following the general procedure, purification with 25% ethyl acetate in hexane yielded **42** as a white-yellow solid (258 mg, 77%).

^{1}H NMR (400 MHz, $CDCl_3$) δ_H 8.33 (1H, s), 8.22 (2H, d, J = 9.1 Hz), 7.84 (2H, d, J = 9.1 Hz), 7.43 – 7.34 (3H, m), 7.11 (1H, dt, J_d = 7.3 Hz, Jt = 2.0 Hz), 6.04 (1H, ddt, J_d = 17.4, 10.5 Hz, J_t = 5.3 Hz), 5.42 (1H, br dd, J = 17.3, 1.3 Hz), 5.31 (1H, br dd, J = 10.5, 1.3 Hz), 4.57 (2H, br d, J = 5.3 Hz) ppm.

^{13}C{^{1}H} NMR (101 MHz, $CDCl_3$) δ 166.0, 159.1, 143.9, 143.7, 135.5, 132.7, 130.1, 125.2 (2C), 119.6 (2C), 119.3, 119.1, 118.3, 113.7, 69.1 ppm.

R$_f$ = 0.8 (66% ethyl acetate in hexane, visualized by UV lamp).

HRMS = (ESI) *m/z*: $[M+H]^+$ calculated for $C_{16}H_{15}N_2O_4$ 299.1032; Found 299.1026.

Synthesis of Oximes: General Procedure

In a round bottom flask, ketone or aldehyde (0.381 mmol, 1 eq) and methoxylamine hydrochloride (0.572 mmol, 1.5 eq), were dissolved in ethanol (2.4 mL, 0.16 M). Once the starting materials were dissolved in the ethanol,

pyridine (0.381 mmol, 1 eq) was added to the reaction mixture. This reaction mixture was then stirred between 10 min and 3 hours at room temperature or 75 ˚C with an oil bath. The solvent was then evaporated from the reaction mixture and the residue was extracted with dichloromethane and washed with 1M HCl. Purification on silica gel column chromatography resulted in oxime being observed as yellow liquids.

(*E*)-7-(Allyloxy)-3,4-dihydronaphthalen-1(2H)-one O-methyl oxime (14)

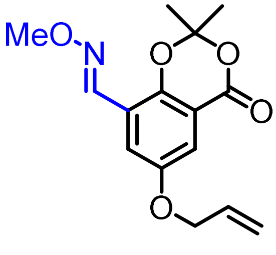

14

Following the general procedure at room temperature for 10 minutes, purification with 10% to 15% ethyl acetate in hexane yielded **14** as a yellow liquid (61.3 mg, 55%).

^{1}H NMR (400 MHz, $CDCl_3$) δ_H 8.26 (1H, s), 7.65 (1H, d, *J* = 3.2 Hz), 7.49 (1H, d, *J* = 3.2 Hz), 6.03 (1H, ddt, J_d = 16.9, 10.3 Hz, J_t = 5.4 Hz), 5.43 (1H, dq, J_d = 17.4 Hz, J_q = 1.6 Hz), 5.31 (1H, dq, J_d = 10.6 Hz, J_q = 1.6 Hz), 4.56 (2H, d, *J* = 5.5 Hz), 3.99 (3H, s), 1.72 (6H, s) ppm.

^{13}C{^{1}H} NMR (101 MHz, $CDCl_3$) δ 160.8, 153.6, 148.1, 141.9, 132.7, 122.4, 119.8, 118.3, 115.6, 114.7, 106.9, 69.6, 25.9 (2C) ppm. Missing carbon due to possible rotamers.

R_f = 0.67 (25% ethyl acetate in hexane, visualized by UV lamp).

HRMS = (ESI) *m/z*: $[M+H]^+$ calculated for for $C_{15}H_{18}NO_5$ 292.1179; Found 292.1184.

(*E*)-7-(Allyloxy)-3,4-dihydronaphthalen-1(2H)-one O-methyl oxime (24)

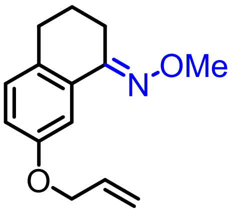

24

Following the general procedure at 75 ˚C and for 3 hours, purification with 10% ethyl acetate in hexane yielded **24** as a yellow liquid (91 mg, 91%).

^{1}H NMR (400 MHz, $CDCl_3$) δ_H 7.51 (1H, d, *J* = 2.7 Hz), 7.04 (1H, d, *J* = 8.4 Hz), 6.86 (1H, dd, *J* = 8.4, 2.7 Hz), 6.07 (1H, ddt, J_d = 16.9, J_d =10.6, J_t= 5.3 Hz), 5.43 (1H, dq, J_d = 17.3, $J_{q\,=}$ 1.5 Hz), 5.29 (1H, dq, J_d = 10.5, J_q = 1.3 Hz), 4.56 (2H, dt, J_d = 5.3, J_t = 1.26 Hz), 3.99 (3H, s), 2.73-2.64 (4H, m), 1.81 (2H, quint, *J* = 6.7 Hz) ppm.

^{13}C{^{1}H} NMR (101 MHz, $CDCl_3$) δ 157.1, 154.0, 133.5, 132.4, 131.5, 129.7, 117.7, 117.2, 108.6, 69.0, 62.1, 29.0, 24.2, 21.7 ppm.

R_f = 0.78 (20% ethyl acetate in hexane, visualized by UV lamp).

HRMS = (ESI) *m/z*: $[M+H]^+$ calculated for $C_{14}H_{18}NO_2$ 232.1332; Found 232.1333.

Lindlar's Reduction: General Procedure

Quinoline (0.025 mmol, 0.08 eq) was added to a round bottom flask containing a suspension of palladium on calcium carbonate poisoned with lead (5% weight, 0.016 mmol, 0.05 eq) in ethyl acetate (10.8 mL, 0.03 M). The flask was evacuated and back filled with H_2 gas three times before addition of a solution of alkyne (0.32 mmol, 1 eq) in ethyl acetate (5 mL) via syringe. The reaction mixture was stirred at 0 °C under an H_2 atmosphere (balloon

pressure) until full consumption of starting material was observed via thin layer chromatography. The mixture was then filtered through a pad of celite and washed with ethyl acetate. The resulting filtrate was concentrated under reduced pressure and purified via column chromatography to afford respective oil.

2,2-Dimethyl-6-((2-methylbut-3-en-2-yl)oxy)-4H-benzo[d][1,3]dioxin-4-one (6)

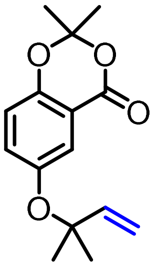

6

Following the general procedure, purification through gradient column chromatography with an eluent of 10% to 30% ethyl acetate in hexane yielded **6** as (25.3 mg, 78%) as clear oil.

^{1}H NMR (500 MHz, $CDCl_3$) δ_H 7.54 (1H, d, J = 2.9 Hz), 7.17 (1H, dd, J = 8.9, 3.0 Hz), 6.83 (1H, d, J = 8.7 Hz), 6.12-6.05 (1H, m), 5.18 (1H, s), 5.15 (1H, dd, J = 6.7, 0.9 Hz), 1.71 (6H, s), 1.43 (6H, s) ppm.

^{13}C{^{1}H} NMR (125 MHz, $CDCl_3$) δ 161.3, 151.5, 150.8, 143.5, 131.5, 121.9, 117.6, 114.6, 113.5, 106.5, 80.4, 26.9 (2C), 25.9 (2C) ppm.

R_f = 0.69 (25 % ethyl acetate in hexane, visualized by UV lamp).

HRMS = (ESI) *m/z*: $[M+H]^+$ calculated for $C_{15}H_{19}O_4$ 263.1278; Found 263.1270.

(*Z*)-6-(But-2-en-1-yloxy)-2,2-dimethyl-4H-benzo[d][1,3]dioxin-4-one (8)

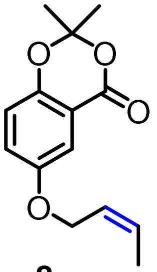

8

Following the general procedure, purification through gradient column chromatography with an eluent of 2% to 16% ethyl acetate in hexane yielded **8** as a mixture of *Z* and *E* isomers of alkenes in a 22 to 1 ratio (76.3 mg, 80%), respectively, as yellow oil.

^{1}H NMR (400 MHz, $CDCl_3$) δ_H 7.40 (1H, d, J = 3.1 Hz), 7.13 (1H, dd, J = 8.9, 3.0 Hz), 6.87 (1H, d, J = 9.1 Hz), 5.75 (1H, dqt, J_d= 10.8 Hz, J_q = 6.9 Hz, J_t =1.4 Hz), 5.67 (1H, dtq, J_d= 11.0 Hz, J_t = 6.3 Hz, J_q =1.6 Hz), 4.57 (2H, d, J = 6.1 Hz), 1.73 (3H, d, J = 7.0 Hz), 1.70 (6H, s) ppm.

^{13}C{^{1}H} NMR (101 MHz, $CDCl_3$) δ 161.4, 154.0, 150.3, 129.3, 125.6, 125.0, 118.5, 113.7, 111.9, 106.5, 64.5, 25.8 (2C), 13.5 ppm.

R_f = 0.46 (25% ethyl acetate in hexane, visualized by UV lamp).

HRMS = (ESI) *m/z*: $[M+H]^+$ calculated for $C_{14}H_{16}O_4$ 249.1121; Found 249.1117.

Aromatic-Claisen Rearrangement: General Procedure

A solution of aryl allyl (or propargyl) ether dissolved in dry chlorobenzene (3 to 5 mL) was heated to 180-210 °C in a microwave reactor (300 W) until complete conversion of starting material was observed via thin layer chromatography (TLC). The resulting mixture was dried under N_2 and further dried under reduced pressure to ensure complete removal of chlorobenzene, before determining the ratio of regioisomers by ^{1}H NMR analysis. Purification was performed on a silica gel column chromatography with isocratic or gradient eluents to yield the *ortho*-alkylated phenol or benzopyran products.

*Imines **25-27** hydrolyzed to the ketone regioisomers (**21a** and **21b**) after the aromatic Claisen Rearrangement, and the initial mixture was used for regioselectivity ratio determination.

5-Allyl-6-hydroxy-2,2-dimethyl-4H-benzo[d][1,3]dioxin-4-one (1a) and 7-allyl-6-hydroxy-2,2-dimethyl-4H-benzo[d][1,3]dioxin-4-one (1b)

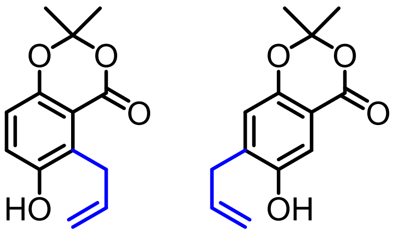

1a **1b**

Following the general procedure at 210 °C for 3 hours, **1a** and **1b** were observed as a 20:1 ratio before purification through gradient column chromatography using 0% to 20% ethyl acetate in hexane to yield regioisomers **1a** and **1b** (95 mg, 95%) as an off-white solid.

(1a) ^{1}**H NMR** (500 MHz, $CDCl_3$) δ_H 7.07 (1H, d, J = 8.9 Hz), 6.79 (1H, d, J = 8.9 Hz), 6.04 (1H, ddt, J_d = 17.2, 10.3 Hz, J_t = 6.0 Hz), 5.15-5.09 (2H, m), 5.01 (1H, br s), 3.99 (2H, dt, J_d = 6.0 Hz, J_t = 1.7 Hz), 1.69 (6H, s) ppm.

(1b) ^{1}**H NMR** (500 MHz, $CDCl_3$) δ_H 7.33 (1H, s), 6.76 (1H, s), 5.98-5.94 (1H, m), 5.21-5.18 (2H, m), 3.42 (2H, d, J = 6.4 Hz), 1.71 (6H, s) ppm.

(1a) ^{13}**C{**^{1}**H} NMR** (125 MHz, $CDCl_3$) δ 160.6, 151.2, 150.3, 135.7, 128.4, 124.0, 116.5, 116.2, 113.0, 105.4, 31.0, 25.6 (2C) ppm. **1b** not observed due to low amount of material.

(1a) R$_f$ = 0.31 (20 % ethyl acetate in hexane, visualized by UV lamp). **(1b) R$_f$** = 0.31 (20 % ethyl acetate in hexane, visualized by UV lamp).

(1a) HRMS = (ESI) *m/z*: $[M+H]^+$ calculated for $C_{14}H_{15}O_4$ 235.0965; Found 235.0973. **(1b) HRMS** = (ESI) *m/z*: $[M+H]^+$ calculated for $C_{14}H_{15}O_4$ 235.0965; Found 235.0973.

3,3,8,8-Tetramethyl-8,9-dihydro-1H-[1,3]dioxino[5,4-e]benzofuran-1-one (4a) 6-Hydroxy-2,2-dimethyl-5-(2-methylallyl)-4H-benzo[d][1,3]dioxin-4-one (4a′) and 6-Hydroxy-2,2-dimethyl-7-(2-methylallyl)-4H-benzo[d][1,3]dioxin-4-one (4b)

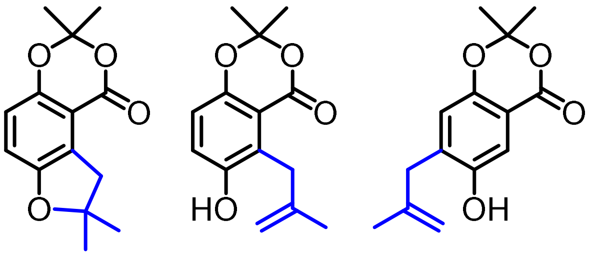

4a **4a′** **4b**

Following the general procedure at 210 °C for nine hours, **4a**, **4a′** and **4b** were observed as a 49.5:3:1 ratio, which equates to a regioselectivity ratio of **4a** and **4a′** to **4b** as a 53:1 ratio before purification through gradient column chromatography from 5% to 15% ethyl acetate in hexane to yield regioisomers **4a** (37.1 mg, 48%) and **4a′**and **4b** (3 mg, 4%) as yellow/brown solids.

(4a) ^{1}**H NMR** (400 MHz, $CDCl_3$) δ_H 6.86 (1H, d, J = 8.7 Hz), 6.70 (1H, d, J = 8.5 Hz), 3.35 (2H, s), 1.69 (6H, s), 1.46 (6H, s) ppm. **(4a′)** ^{1}**H NMR** (400 MHz, $CDCl_3$) δ_H 7.08 (1H, d, J = 8.7 Hz), 6.81 (1H, d, J = 8.8 Hz), 5.27 (1H, br s), 4.90 (1H, s), 4.70 (1H, s), 4.03 (2H, s), 1.81 (3H, s), 1.96 (6H, s) ppm. **(4b)** ^{1}**H NMR** (400 MHz, $CDCl_3$) δ_H 7.37 (1H, s), 6.75 (1H, s), 4.96 (1H, s), 4.83 (1H, s), 3.38 (2H, s), 1.75 (3H, s), 1.71 (6H, s) ppm.

(4a) ^{13}**C NMR** (125 MHz, $CDCl_3$) δ 161.1, 154.7, 149.7, 129.7, 116.5, 116.2, 110.9, 106.5, 88.3, 43.8, 28.4 (2C), 25.8 (2C) ppm. **(4a′)** ^{13}**C NMR** (125 MHz, $CDCl_3$) δ 160.3, 150.4, 148.2, 140.0, 125.5, 122.6, 118.3, 117.1, 112.2, 105.7, 25.7, 25.6 (2C), 19.6 ppm. ^{13}C NMR data not observed of **4b** due to low amount of material of regioisomers **4a′**and **4b**.

(4a) R_f = 0.67 (25 % ethyl acetate in hexane, visualized by UV lamp). **(4a′and 4b) R_f** = 0.37 (25 % ethyl acetate in hexane, visualized by UV lamp).

(4a) HRMS = (ESI) *m/z*: $[M+H]^+$ calculated for $C_{14}H_{17}O_4$ 249.1121; Found 249.1119 **(4a′) HRMS** = (ESI) *m/z*: $[M+H]^+$ calculated for $C_{14}H_{17}O_4$ 249.1121; Found 249.1119. **(4b) HRMS** = (ESI) *m/z*: $[M+H]^+$ calculated for $C_{14}H_{17}O_4$ 249.1121; Found 249.1119.

6-Hydroxy-2,2-dimethyl-7-(2-methylbut-3-en-2-yl)-4H-benzo[d][1,3]dioxin-4-one (5b)

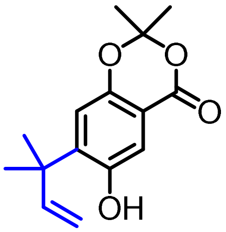

5b

Following the general procedure using dry DMF as the solvent at 210 °C for three hours, **5b** was only observed before purification through isocratic column chromatography at 15% ethyl acetate in hexane to yield the regioisomer as a white solid (8.8 mg, 14%).

^{1}H NMR (500 MHz, $CDCl_3$) δ_H 7.38 (1H, s), 6.89 (1H, s), 6.16 (1H, dd, *J* = 17.8, 10.4 Hz), 5.72 (1H, s), 5.34 (1H, dd, *J* = 17.8, 3.6 Hz) 5.34 (1H, dd, *J* = 10.4, 3.6 Hz), 1.72 (6H, s), 1.45 (6H, s) ppm.

^{13}C NMR (125 MHz, $CDCl_3$) δ 161.2, 150.1, 150.0, 146.2, 142.85, 117.1, 115.3, 114.7, 112.8, 106.5, 41.2, 26.8 (2C), 25.9 (2C) ppm.

R_f = 0.37 (25 % ethyl acetate in hexane, visualized by UV lamp).

HRMS = (ESI) *m/z*: $[M+H]^+$ calculated for $C_{15}H_{19}O_4$ 263.1278; Found 263.1275.

6-Hydroxy-2,2-dimethyl-5-(3-methylbut-2-en-1-yl)-4H-benzo[d][1,3]dioxin-4-one (6a) and 6-Hydroxy-2,2-dimethyl-7-(3-methylbut-2-en-1-yl)-4H-benzo[d][1,3]dioxin-4-one (6b)

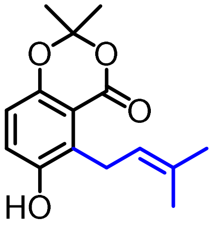

6a

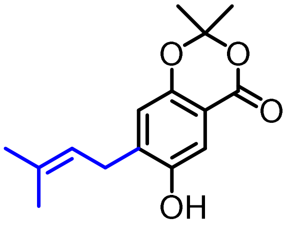

6b

Following the general procedure at 210 °C for three hours, **6a** and **6b** were observed as a 4:1 ratio of **6a** and **6b** as an orange/brown solid (20.2 mg, 32%). Trimethoxybenzene was used as an internal standard to determine the yield. Impure material could not be purified due to product degradation on the silica gel column.

(6a) ^{1}H NMR (400 MHz, Acetone-*d_6*) δ_H 8.41 (1H, br s), 7.12 (1H, d, *J* = 8.8 Hz), 6.72 (1H, d, *J* = 9.2 Hz), 5.19 (1H, tt, *J* = 7.0, 1.3 Hz), 3.86 (2H, d, *J* = 7.4 Hz), 1.73 (3H, s), 1.60 (6H, s), 1.60 (3H, s) ppm. **(6b) ^{1}H NMR** (400 MHz, Acetone-*d_6*) δ_H 8.59 (1H, br s), 7.28 (1H, s), 6.74 (1H, s), 5.31 (1H, tt, *J* = 7.4, 1.4 Hz), 3.34 (2H, d, *J* = 7.8 Hz) ppm. Missing geminal methyl protons in **6b**.

HRMS = (ESI) *m/z*: $[M+H]^+$ calculated for $C_{15}H_{19}O_4$ 263.1278; Found 263.1270.

5-(But-3-en-2-yl)-6-hydroxy-2,2-dimethyl-4H-benzo[d][1,3]dioxin-4-one (7a) and 7-(But-3-en-2-yl)-6-hydroxy-2,2-dimethyl-4H-benzo[d][1,3]dioxin-4-one (7b)

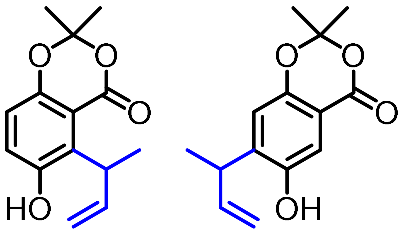

7a **7b**

Following the general procedure at 210 °C for 17 hours, **7a** and **7b** were observed as a 1:1 ratio with the *E*-alkene allyl ether before purification through isocratic column chromatography at 15% ethyl acetate in hexane to yield the regioisomers as a yellow solid (26.8 mg, 33%). After 12 hours, **7a** and **7b** were observed as a 1:9 ratio with the *Z*-alkene allyl ether before purification through isocratic column chromatography at 15% ethyl acetate in hexane to yield the regioisomers as a yellow solid (11.6 mg, 48%).

(7a) ^{1}**H NMR** (400 MHz, $CDCl_3$) δ_H 7.02 (1H, d, J = 9.1 Hz), 6.80 (1H, d, J = 9.2 Hz), 6.30 (1H, ddd, J = 18.0, 10.4, 3.6 Hz), 5.83 (1H, s), 5.46-5.43 (1H, m), 5.43-5.40 (1H, m), 5.25-5.16 (1H, m), 1.71 (3H, s), 1.68 (3H, s), 1.44 (3H, d, J = 6.3 Hz) ppm. **(7b)** ^{1}**H NMR** (400 MHz, $CDCl_3$) δ_H 7.37 (1H, s), 6.77 (1H, s), 6.06-5.95 (1H, m), 5.35 (1H, s), 5.21-5.17 (1H, m), 5.17-5.14 (1H, m), 3.80 (1H, quint, J = 6.8 Hz), 1.71 (6H, s), 1.37 (3H, d, J = 7.0 Hz) ppm.

(7a) ^{13}**C{**^{1}**H} NMR** (101 MHz, $CDCl_3$) δ 160.9, 151.5, 151.0, 141.9, 131.8, 125.5, 117.1, 116.2, 112.5, 105.1, 34.8, 26.1, 25.1, 15.7 ppm. **(7b)** ^{13}**C{**^{1}**H} NMR** (101 MHz, $CDCl_3$) δ 161.5, 150.4, 149.0, 142.0, 141.0, 116.4, 115.2, 114.9, 111.9, 106.6, 37.4, 25.9 (2C), 18.8 ppm.

(7a) $\mathbf{R_f}$ = 0.48 (30 % ethyl acetate in hexane, visualized by UV lamp). **(7b)** $\mathbf{R_f}$ = 0.43 (30 % ethyl acetate in hexane, visualized by UV lamp).

(7a) HRMS = (ESI) *m/z*: $[M+H]^+$ calculated for $C_{14}H_{17}O_4$ 249.1121; Found 249.1117. **(7b) HRMS** = (ESI) *m/z*: $[M+H]^+$ calculated for $C_{14}H_{17}O_4$ 249.1121; Found 249.1118.

5-(But-3-en-2-yl)-6-hydroxy-2,2-dimethyl-4H-benzo[d][1,3]dioxin-4-one (9a)

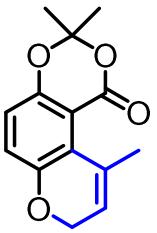

9a

Following the general procedure with allyl ether **9** at 210 °C for three hours, **9a** was only observed before purification through gradient column chromatography from 0% to 15% ethyl acetate in hexane to yield the regioisomer **9a** as an off-white/yellow solid (3 mg, 3%). After three hours, **9a** was only observed with aryl alkyl ether **12** before purification through gradient column chromatography from 1% to 5% ethyl acetate in hexane to yield the regioisomer **9a** as an off-white/yellow solid (3.8 mg, 5%).

^{1}**H NMR** (400 MHz, $CDCl_3$) δ_H 7.11 (1H, d, J = 8.8 Hz), 6.80 (1H, d, J = 8.8 Hz), 5.86 (1H, t, J = 4.8 Hz), 4.50 (2H, dd, J = 4.8, 0.9 Hz), 2.10 (3H, s), 1.76 (6H, s) ppm.

^{13}**C{**^{1}**H} NMR** (125 MHz, $CDCl_3$) δ 159.8, 151.7, 151.5, 132.4, 126.6, 142.0, 121.5, 117.2, 111.1, 105.1, 64.73, 25.2 (2C), 20.4 ppm.

$\mathbf{R_f}$ = 0.57 (25% ethyl acetate in hexane, visualized by UV lamp).

HRMS = (ESI) *m/z*: $[M+H]^+$ calculated for $C_{14}H_{15}O_4$ 247.0965; Found 247.0961.

3,3-Dimethyl-[1,3]dioxino[5,4-f]chromen-1(8H)-one (10a)

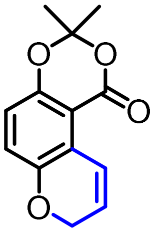

10a

Following the general procedure at 210 °C for three hours, **10a** was only observed before purification through isocratic column chromatography at 15% ethyl acetate in hexane to yield the regioisomer **10a** as a pale yellow oil (73 mg, 73%).

^{1}H NMR (400 MHz, $CDCl_3$) δ_H 7.60 (1H, dt, J_d = 10.1 Hz, J_t = 2.0 Hz), 7.00 (1H, d, J = 8.7 Hz), 6.73 (1H, d, J = 9.1 Hz), 6.01 (1H, dt, J_d = 10.3 Hz, J_t = 3.8 Hz), 4.76 (2H, dd, J = 3.9, 1.9 Hz), 1.70 (6H, s) ppm.

^{13}C{^{1}H} NMR (101 MHz, $CDCl_3$) δ 160.8, 151.1, 149.7, 125.4, 125.2, 123.8, 122.8, 117.1, 108.8, 105.7, 64.9, 25.7 (2C) ppm.

R$_f$ = 0.66 (25% ethyl acetate in hexane, visualized by UV lamp).

HRMS = (ESI) *m/z*: $[M+H]^+$ calculated for $C_{13}H_{13}O_4$ 233.0814; Found 233.0804.

3,3,8,8-Tetramethyl-[1,3]dioxino[5,4-f]chromen-1(8H)-one (11a)

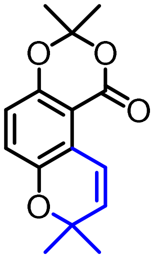

11a

Following the general procedure at 210 °C for nine hours, **11a** was only observed before purification through gradient column chromatography from 0% to 15% ethyl acetate in hexane to yield the regioisomer **11a** as a white solid (3.6 mg, 38%).

^{1}H NMR (400 MHz, $CDCl_3$) δ_H 7.50 (1H, d, J = 10.5 Hz), 6.98 (1H, d, J = 8.6 Hz), 6.73 (1H, d, J = 8.8 Hz), 5.82 (1H, d, J = 9.8 Hz), 1.70 (6H, s), 1.43 (6H, s) ppm.

^{13}C{^{1}H} NMR (101 MHz, $CDCl_3$) δ 160.9, 150.8, 148.6, 134.2, 124.5, 122.5, 120.2, 108.7, 105.6, 75.8, 27.5 (2C), 25.7 (2C) ppm.

R$_f$ = 0.43 (25% ethyl acetate in hexane, visualized by UV lamp).

HRMS = (ESI) *m/z*: $[M+H]^+$ calculated for $C_{15}H_{17}O_4$ 261.1121; Found 261.1117.

5-Allyl-6-hydroxy-2,2-dimethyl-4-oxo-4H-benzo[d][1,3]dioxine-8-carbaldehyde (13a) and (*E*)-6-Hydroxy-2,2-Dimethyl-4-oxo-7-(prop-1-en-1-yl)-4H-benzo[d][1,3]dioxine-8-carbaldehyde (13b)

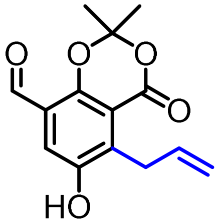

13a

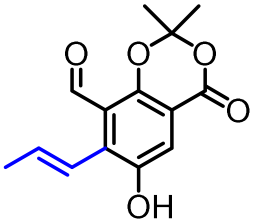

13b

Following the general procedure at 180 °C for 18 hours, **13a** and **13b** were observed as a 2.4:1 ratio utilizing NOESY and HMBC before purification through gradient column chromatography from 10% to 40% ethyl acetate in hexane to yield regioisomers **13a** and **13b** as an orange and yellow solid, respectively (45.5 mg, 37%).

(13a) ^{1}H NMR (400 MHz, $CDCl_3$) δ_H 10.29 (1H, s), 7.67 (1H, s), 6.61-6.51 (1H, m), 6.02 (1H, ddt, J_d = 16.3, 9.9 Hz, J_t = 6.1 Hz), 5.15-5.04 (2H, m), 4.04 (2H, d, J = 6.3 Hz), 1.77 (6H, s) ppm. **(13b) ^{1}H NMR** (400 MHz, $CDCl_3$) δ_H 10.34 (1H, s), 7.73 (1H, s), 6.77 (1H, dq, J_d = 16.4 Hz, J_q = 1.7 Hz), 6.13 (1H, dq, J_d = 16.4 Hz, J_q = 6.7 Hz), 6.00 (1H, s), 2.02 (3H, dd, J = 6.5, 1.7 Hz), 1.78 (6H, s).

(13a) ^{13}C{^{1}H} NMR (101 MHz, $CDCl_3$) δ 187.9, 1596, 153.2, 150.6, 139.0, 134.7, 123.4, 119.6, 116.6, 114.2, 106.3, 31.3, 25.8 (2C) ppm. **(13b) ^{13}C{^{1}H} NMR** (101 MHz, $CDCl_3$) δ 189.2, 160.1, 152.1, 148.5, 135.8, 133.5, 24.6, 122.8, 120.5, 113.3, 107.3, 26.0 (2C), 19.4 ppm.

(13a) R$_f$ = 0.42 (25% ethyl acetate in hexane, visualized by UV lamp). **(13b) R$_f$** = 0.33 (25% ethyl acetate in hexane, visualized by UV lamp).

(13a) HRMS = (ESI) *m/z*: $[M+H]^+$ calculated for $C_{14}H_{15}O_5$ $[M+H]^+$ 263.0914; Found 263.0912. **(13b) HRMS** = (ESI) *m/z*: $[M+H]^+$ calculated for $C_{14}H_{15}O_5$ 263.0914; Found 263.0912.

(*E*)-5-Allyl-6-hydroxy-2,2-dimethyl-4-oxo-4H-benzo[d][1,3]dioxine-8-carbaldehyde O-methyl oxime (14a) and (*E*)-7-allyl-6-hydroxy-2,2-dimethyl-4-oxo-4H-benzo[d][1,3]dioxine-8-carbaldehyde O-methyl oxime (14b)

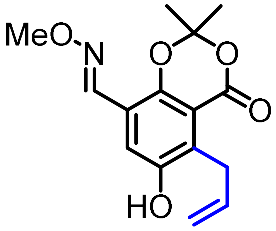

14a

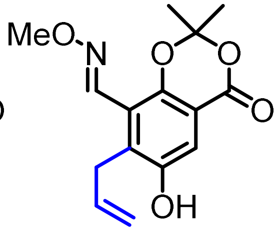

14b

Following the general procedure at 210 °C for 6 hours, **14a** and **14b** were observed as a 6.7 to 1 ratio utilizing NOESY and HMBC before purification through gradient column chromatography from 0% to 25% ethyl acetate in hexane to yield regioisomers **14a** and **14b** as light yellow solids (8.4 mg, 57%).

(14a) ^{1}H NMR (400 MHz, $CDCl_3$) δ_H 8.25 (1H, s), 7.55 (1H, s), 6.01 (1H, ddt, J_d = 16.8, 11.8 Hz, J_t = 6.0 Hz), 5.15-5.07 (2H, m), 5.03 (1H, br s), 4.01-3.96 (5H, m), 1.69 (6H, s) ppm.

(14b) ^{1}H NMR (400 MHz, $CDCl_3$) δ_H 8.13 (1H, s), 7.56 (1H, s), 6.02 (1H, ddt, J_d = 16.9, 11.8 Hz, J_t = 6.0 Hz), 5.13-5.07 (2H, m), 5.06 (1H, br s), 4.02 (3H, s), 4.00-3.96 (2H, m), 1.71 (6H, s) ppm.

(14a) ^{13}C{^{1}H} NMR (101 MHz, $CDCl_3$) δ 150.1, 142.1, 135.4, 131.6, 120.4, 118.8, 116.4, 105.7, 62.5, 31.1, 25.8 (2C) ppm.

(14b) ^{13}C{^{1}H} NMR (101 MHz, $CDCl_3$) δ 160.0, 149.6, 149.3, 138.2, 135.3, 131.4, 125.3, 118.5, 116.5, 113.3, 105.7, 62.9, 31.2, 25.7 (2C) ppm.

(14a) R$_f$ = 0.50 (25% ethyl acetate in hexane, visualized by UV lamp). **(14b) R$_f$** = 0.28 (25% ethyl acetate in hexane, visualized by UV lamp).

(14a) HRMS = (ESI) *m/z*: $[M+H]^+$ calculated for $C_{15}H_{18}NO_5$ 292.1179; Found 292.1175. **(14b) HRMS** = (ESI) *m/z*: $[M+H]^+$ calculated for $C_{15}H_{18}NO_5$ 292.1179; Found 292.1177.

5-Allyl-8-ethynyl-6-hydroxy-2,2-dimethyl-4H-benzo[d][1,3]dioxin-4-one (15a) and7-allyl-8-ethynyl-6-hydroxy-2,2-dimethyl-4H-benzo[d][1,3]dioxin-4-one (15b)

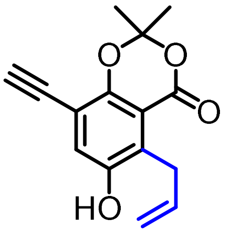

15a

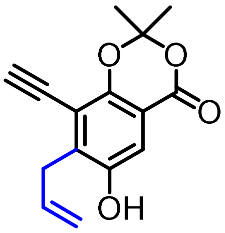

15b

Following the general procedure at 210 °C for four hours, **15a** and **15b** were observed as a 3:1 ratio utilizing HMBC before purification through gradient column chromatography from 10% to 30% ethyl acetate in hexane to yield regioisomers **15a** and **15b** as white solids (7.6 mg, 51%).

(15a) ^{1}H NMR (400 MHz, $CDCl_3$) δ_H 7.20 (1H, s), 6.02 (1H, ddt, J_d = 16.5, 10.1 Hz, J_t = 6.0 Hz), 5.16-5.08 (2H, m), 4.99 (1H, br s), 3.99 (2H, dt, J_d = 5.9 Hz, J_t = 1.7 Hz), 3.27 (1H, s), 1.74 (6H, s) ppm.

(15b) ^{1}H NMR (400 MHz, $CDCl_3$) δ_H 7.38 (1H, s), 6.08-5.90 (1H, m), 5.20-5.07 (2H, m), 4.98 (1H, br s), 3.69 (2H, dt, J_d = 6.4 Hz, J_t = 1.7 Hz), 3.50 (1H, s), 1.76 (6H, s) ppm.

(15a and 15b) ^{13}C{^{1}H} NMR (101 MHz, $CDCl_3$) δ 161.0, 160.2, 152.3, 151.8, 149.7, 149.3, 138.6, 135.3, 134.0, 130.6, 126.8, 117.2, 116.5, 115.5, 113.5, 112.9, 112.2, 111.0, 107.2, 106.0, 86.7, 82.7, 76.0, 33.5, 31.1, 29.8, 25.9, 25.7 ppm.

(15a) R$_f$ = 0.50 (25 % ethyl acetate in hexane, visualized by UV lamp). **(15a) R$_f$** = 0.47 (25 % ethyl acetate in hexane, visualized by UV lamp).

(15a HRMS = (ESI) *m/z*: $[M+H]^+$ calculated for $C_{15}H_{15}O_4$ 259.0965; Found 259.0964. **(15b) HRMS** = (ESI) *m/z*: $[M+H]^+$ calculated for $C_{15}H_{15}O_4$ 259.0965; Found 259.0963.

5-Allyl-6-hydroxy-2,2-dimethyl-8-phenyl-4H-benzo[d][1,3]dioxin-4-one (16a)

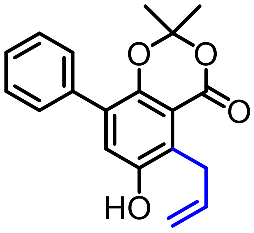

16a

Following the general procedure at 210 °C for four hours, **16a** was only observed utilizing NOESY before purification through gradient column chromatography from 0% to 20% ethyl acetate in hexane to yield the regioisomer **16a** as a tan solid (34.0 mg, 48%).

^{1}H NMR (500 MHz, $CDCl_3$) δ_H 7.47-7.34 (5H, m), 7.18 (1H, s), 6.08 (1H, ddt, J_d = 17.2, 10.0 Hz, J_t = 6.0 Hz), 5.15 (1H, dq, J_d = 17.3 Hz, J_q = 1.6 Hz), 5.11 (1H, dq, J_d = 10.1 Hz, J_q = 1.5 Hz), 4.01 (2H, dt, J_d = 6.1 Hz, J_t = 1.6 Hz), 1.66 (6H, s) ppm, O-H peak not observed.

^{13}C{^{1}H} NMR (125 MHz, $CDCl_3$) δ 161.2, 150.2, 147.8, 135.9 (2C), 130.0, 129.2 (2C), 128.4 (2C), 128.0, 127.9, 124.4, 116.1, 113.8, 105.4, 31.0, 25.6 (2C) ppm.

R$_f$ = 0.39 (40% ether in hexane, visualized by UV lamp).

HRMS = (ESI) *m/z*: $[M+H]^+$ calculated for $C_{19}H_{19}O_4$ 311.1283; Found 311.1288.

5-Allyl-8-(1-benzyl-1H-1,2,3-triazol-4-yl)-6-hydroxy-2,2-dimethyl-4H-benzo[d][1,3]dioxin-4-one (17a) and 7-allyl-8-(1-benzyl-1H-1,2,3-triazol-4-yl)-6-hydroxy-2,2-dimethyl-4H-benzo[d][1,3]dioxin-4-one (17b)

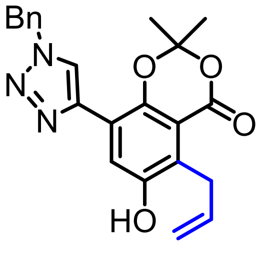

17a

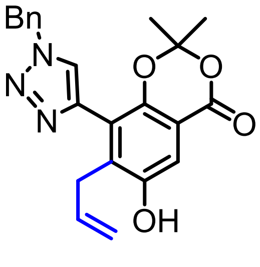

17b

Following the general procedure at 210 °C for 4 hours, **17a** and **17b** were observed as a 5.1:1 ratio utilizing NOESY and HMBC before purification through gradient column chromatography from 0% to 35% ethyl acetate in hexane to yield regioisomers **17a** and **17b** as white solids (10.5 mg, 44%).

(17a) ^{1}H NMR (500 MHz, Acetone-d_6) δ_H 8.70 (1H, s), 8.42 (1H, s), 8.14 (1H, s), 7.42-7.31 (5H, m), 6.00 (1H, ddt, J_d = 17.2, 10.0 Hz, J_t = 6.2 Hz), 5.70 (2H, s), 5.03 (1H, dq, J_d = 17.5 Hz, J_q = 1.8 Hz), 4.91 (1H, dq, J_d = 10.3 Hz, J_q = 1.8 Hz), 3.95 (2H, dt, J_d = 6.3 Hz, J_t = 1.6 Hz), 1.69 (6H, s) ppm.

(17b) ^{1}H NMR (500 MHz, Acetone-d_6) δ_H 8.80 (1H, br s), 8.15 (1H, s), 7.42-7.31 (6H, m), 5.89 (1H, ddt, J_d = 18.0, 11.6 Hz, J_t = 6.7 Hz), 5.73 (2H, s), 4.83 (1H, t, J = 1.5 Hz), 4.80 (1H, dq, J_d = 6.2 Hz, J_q = 1.8 Hz), 3.63 (2H, dt, J_d = 6.5 Hz, J_t = 1.4 Hz), 1.61 (6H, s) ppm.

(17a and 17b) ^{13}C{^{1}H} NMR (101 MHz, Acetone-d_6) δ 161.0, 160.4, 151.8, 151.6, 146.7, 141.7, 137.4, 137.1, 136.8, 130.3, 129.7, 129.1, 128.7, 124.6, 119.8, 119.6, 115.2, 114.7, 113.1, 106.8, 106.0, 54.1, 30.7, 25.7 (2C) ppm. Some carbon peaks are missing due to overlapping peaks.

(17a and 17b) R$_f$ = 0.40 (50% ethyl acetate in hexane, visualized by UV lamp).

(17a) HRMS = (ESI) *m/z*: [M+H]$^+$ calculated for $C_{22}H_{22}N_3O_4$ 392.1605; Found 392.1599. **(17b) HRMS** = (ESI) *m/z*: [M+H]$^+$ calculated for $C_{22}H_{22}N_3O_4$ 392.1605; Found 392.1597.

1-Allyl-5,6,7,8-tetrahydronaphthalen-2-ol (18a) and 3-Allyl-5,6,7,8-tetrahydronaphthalen-2-ol (18b)

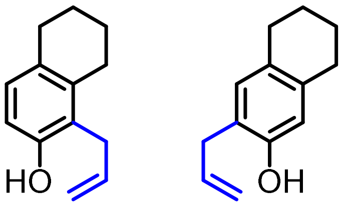

18a **18b**

Following the general procedure at 210 °C for five hours, **18a** and **18b** were observed as a 3:1 ratio with no purification to yield the regioisomers **18a** and **18b** as a tan solid (96 mg, 96%).

(18a) ^{1}H NMR (400 MHz, CDCl$_3$) δ_H 6.86 (1H, d, J = 9.1 Hz), 6.62 (1H, d, J = 9.0 Hz), 5.95 (1H, ddt, J_d = 16.4 Hz, 11.4 Hz, J_t = 5.8 Hz), 5.09-4.97 (2H, m), 3.40 (2H, dt, J_d = 5.8 Hz, J_t = 1.9 Hz), 2.75-2.63 (4H, m), 1.84-1.67 (4H, m) ppm, O-H peak not observed. **(18b) ^{1}H NMR** (400 MHz, CDCl$_3$) δ_H 6.80 (1H, s), 6.53 (1H, s), 6.08-5.99 (1H, m), 5.20-5.10 (2H, m), 3.35 (2H, d, J = 6.4 Hz), 2.75-2.63 (4H, m), 1.84-1.67 (4H, m) ppm, O-H peak not observed.

(18a and 18b) ^{13}C{^{1}H} NMR (125 MHz, CDCl$_3$) δ 151.6, 151.6, 136.9, 136.6, 136.6, 135.8, 130.8, 129.8, 129.4, 128.1, 123.4, 123.0, 116.1, 115.9, 115.2, 113.2, 34.8, 30.0, 30.0, 29.1, 28.5, 26.5, 23.5, 23.4, 23.2, 22.9 ppm.

(18a and 18b) R$_f$ = 0.81 (20% ethyl acetate in hexane, visualized by UV lamp).

(18a) HRMS = (ESI) *m/z*: [M+H]$^+$ calculated for $C_{13}H_{17}O$ 189.1274; Found 189.1280. **(18b HRMS** = (ESI) *m/z*: [M+H]$^+$ calculated for $C_{13}H_{17}O$ 189.1274; Found 189.1281.

1-Allyl-8-methyl-5,6,7,8-tetrahydronaphthalen-2-ol (19a) and 3-Allyl-8-methyl-5,6,7,8-tetrahydronaphthalen-2-ol (19b)

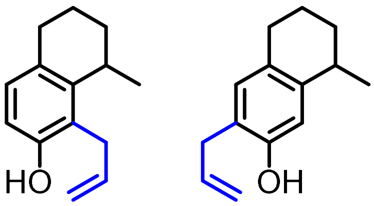

19a **19b**

Following the general procedure at 210 °C for three hours, **19a** and **19b** were observed as a 2:1 ratio with no purification to yield the regioisomers **19a** and **19b** as a yellow oil (78 mg, 78%).

(19a) ^{1}H NMR (400 MHz, CDCl$_3$) δ_H 6.87 (1H, d, J = 8.7 Hz), 6.65 (1H, d, J = 8.7 Hz), 6.07-5.95 (1H, m), 5.21-5.06 (2H, m), 4.81 (1H, s), 3.51 (1H, ddt, J_d = 16.6, 5.5 Hz, J_t = 2.0 Hz), 3.42 (1H, ddt, J_d = 16.9, 5.7 Hz, J_t = 2.0 Hz), 3.14-3.06 (1H, m), 2.86-2.59 (2H, m), 1.92-1.62 (4H, m), 1.17 (3H, d, J = 7.4 Hz) ppm. **(19b) ^{1}H NMR** (400 MHz, CDCl$_3$) δ_H 6.79 (1H, s), 6.67 (1H, s), 6.07-5.95 (1H, m), 5.21-5.06 (2H, m), 4.75 (1H, s), 3.35 (2H, d, J = 6.7 Hz), 2.86-2.5g9 (3H, m), 1.92-1.62 (4H, m), 1.26 (3H, d, J = 6.9 Hz) ppm.

(19a and 19b) ^{13}C{^{1}H} NMR (125 MHz, $CDCl_3$) δ 156.7, 143.5, 133.8, 129.9, 129.6, 129.4, 117.7, 114.3, 114.0, 112.3, 69.0, 39.0, 32.9, 31.6, 29.8, 29.3, 23.9, 22.9, 20.7, 14.2, 11.2 ppm. Several signals are overlapping each other due to very similar chemical shifts.

(19a and 19b) R_f = 0.83 (20% ethyl acetate in hexane, visualized by UV lamp).

(19a) HRMS = (ESI) *m/z*: $[M+H]^+$ calculated for $C_{14}H_{19}O$ 203.1430; Found 203.1427. **(19b) HRMS** = (ESI) *m/z*: $[M+H]^+$ calculated for $C_{14}H_{19}O$ 203.1430; Found 203.1427.

1-Allyl-8-methylene-5,6,7,8-tetrahydronaphthalen-2-ol (20a) and 3-Allyl-8-methylene-5,6,7,8-tetrahydronaphthalen-2-ol (20b)

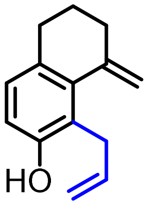

20a

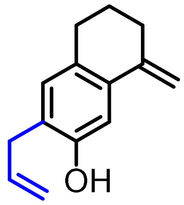

20b

Following the general procedure at 210 °C for three hours, **20a** and **20b** were observed as a 4:1 ratio with purification through isocratic column chromatography at 15% ethyl acetate in hexane to yield the regioisomers **20a** and **20b** (320 mg, 98%) as a red liquid.

(20a) ^{1}H NMR (400 MHz, $CDCl_3$) δ_H 6.93 (1H, d, J = 8.5 Hz), 6.73 (1H, d, J = 8.1 Hz), 6.12 (1H, ddt, J_d = 17.3, 10.3 Hz, J_t = 4.9 Hz), 5.20 (1H, dq, J_d = 10.3 Hz, J_q = 1.9 Hz), 5.17 (1H, q, J = 1.5 Hz), 5.10 (1H, dq, J_d = 17.3 Hz, J_q = 1.9 Hz), 5.04 (1H, s), 4.93 (1H, br s), 3.62 (2H, dt, J_d = 5.2 Hz, J_t = 1.8 Hz), 2.65 (2H, t, J = 6.4 Hz), 2.47 (2H, t, J = 7.0 Hz), 1.84 (2H, quint, J = 6.7 Hz) ppm. **(20b) ^{1}H NMR** (101 MHz, $CDCl_3$) δ_H 7.09 (1H, s), 6.86 (1H, s), 6.01 (1H, ddt, J_d = 16.9, 10.5 Hz, J_t = 5.3 Hz), 5.39 (1H, s), 5.20 (1H, dq, J_d = 10.3 Hz, J_q = 1.9 Hz), 5.15-5.13 (1H, m), 4.78 (1H, br s), 3.40-3.34 (2H, m), 2.75 (2H, quint, J = 6.2 Hz), 2.52-2.44 (2H, m), 1.84 (2H, quint, J = 6.7 Hz) ppm.

(20a and 20b) ^{13}C{^{1}H} NMR (125 MHz, $CDCl_3$) δ 153.5, 152.6, 143.6, 139.0, 137.1, 136.7, 135.7, 132.2, 131.9, 129.3, 128.8, 127.0, 125.6, 123.1, 121.3, 116.5, 116.4, 114.7, 113.8, 111.0, 35.1, 33.0, 32.3, 29.5, 27.4, 23.6, 23.6, 19.4 ppm.

(20a and 20b) R_f = 0.83 (20% ethyl acetate in hexane, visualized by UV lamp).

(20a) HRMS = (ESI) *m/z*: $[M+H]^+$ calculated for $C_{14}H_{17}O$ 201.1274; Found 201.1282. **(20b) HRMS** = (ESI) *m/z*: $[M+H]^+$ calculated for $C_{14}H_{17}O$ 201.1274; Found 201.1278.

8-Allyl-7-hydroxy-3,4-dihydronaphthalen-1(2H)-one (21a) and 6-Allyl-7-hydroxy-3,4-dihydronaphthalen-1(2H)-one (21b)

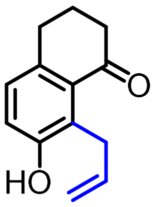

21a

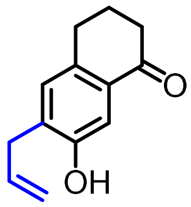

21b

Following the general procedure at 210 °C for three hours, **21a** and **21b** were observed as a 6.5:1 ratio before purification through gradient column chromatography from 0% to 10% ethyl acetate in hexane to yield regioisomers **21a** and **21b** (28.2 mg, 75%) as a light red liquid.

(21a) ^{1}H NMR (400 MHz, $CDCl_3$) δ_H 7.04 (1H, d, J = 8.6 Hz), 7.00 (1H, d, J = 8.3 Hz), 6.07 (1H, ddt, J_d = 16.5, 10.3 Hz, J_t = 6.1 Hz), 5.68 (1H, br s), 5.13-5.04 (2H, m), 3.90 (2H, d, J = 5.6 Hz), 2.88 (2H, t, J = 6.5 Hz), 2.64 (2H, t, J = 7.0 Hz), 2.05 (2H, quint, J = 6.4 Hz) ppm. **(21b) ^{1}H NMR** (400 MHz, $CDCl_3$) δ_H 7.59 (1H, s), 7.02 (1H, s), 6.07-5.93 (2H, m), 5.14 (2H, d, J = 13.7 Hz), 3.44 (2H, d, J = 6.5 Hz), 2.87 (2H, t, J = 6.0 Hz), 2.62 (2H, t, J = 6.0 Hz), 2.10 (2H, quint, J = 6.3 Hz) ppm.

(21a) $^{13}C\{^1H\}$ NMR (101 MHz, $CDCl_3$) δ 201.2, 153.9, 138.4, 136.8, 132.1, 128.0, 126.9, 120.9, 115.4, 41.0, 31.1, 30.5, 23.3 ppm. **(21b) $^{13}C\{^1H\}$ NMR** (101 MHz, $CDCl_3$) δ 199.1, 153.0, 137.4, 135.8, 133.5, 132.0, 130.6, 116.8, 112.9, 39.1, 34.9, 28.9, 23.7 ppm.

(21a) R_f = 0.34 (20% ethyl acetate in hexane, visualized by UV lamp. **(21b) R_f** = 0.24 (20% ethyl acetate in hexane, visualized by UV lamp).

(21a) HRMS = (ESI) *m/z*: $[M+H]^+$ calculated for $C_{13}H_{15}O_2$ 203.1067; Found 203.1062. **(21b) HRMS** = (ESI) *m/z*: $[M+H]^+$ calculated for $C_{13}H_{15}O_2$ 203.1067; Found 203.1063.

1-Allyl-5,6-dihydronaphthalen-2-ol (22a) and 3-Allyl-5,6-dihydronaphthalen-2-ol (22b)

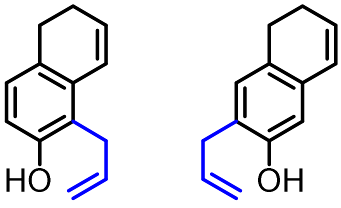

22a **22b**

Following the general procedure with acetate **22** at 210 °C for three hours, **22a** and **22b** were initially observed as a 2:1 ratio before purification through gradient column chromatography from 10% to 20% ether in hexane to yield regioisomers **22a** and **22b** (56.6 mg, 54%) as a light red liquid.

Following the general procedure with alcohol **23** at 210 °C for five hours, **22a** and **22b** were initially observed as a 1:1.3 ratio before purification through isocratic column chromatography at 15% ethyl acetate in hexane to yield regioisomers **22a** and **22b** (120 mg, 90%) as a light red liquid.

(22a) ^1H NMR (500 MHz, $CDCl_3$) δ_H 6.90 (1H, d, J = 8.1 Hz), 6.69 (1H, dt, J_d = 9.6 Hz, J_t = 1.8 Hz), 6.63 (1H, d, J = 8.1 Hz), 6.15 (1H, dt, J_d = 10.1 Hz, J_t = 4.1 Hz), 6.08-5.96 (1H, m), 5.11-5.02 (2H, m), 4.91 (1H, br s), 3.50 (2H, dt, J_d = 5.8 Hz, J_t = 1.8 Hz), 2.73 (2H, t, J = 8.1 Hz), 2.33-2.23 (2H, m) ppm. **(22b) ^1H NMR** (500 MHz, $CDCl_3$) δ_H 6.87 (1H, s), 6.53 (1H, s), 6.39 (1H, dt, J_d = 9.5 Hz, J_t = 1.8 Hz), 6.07-5.96 (2H, m), 5.22-5.15 (2H, m), 4.97 (1H, br s), 3.40 (2H, dt, J_d = 6.6 Hz, J_t = 1.3 Hz), 2.72 (2H, t, J = 8.2 Hz), 2.33-2.23 (2H, m) ppm.

(22a and 22b) $^{13}C\{^1H\}$ NMR (125 MHz, $CDCl_3$) δ 152.5, 152.5, 136.8, 136.3, 133.8, 133.4, 130.1, 129.5, 128.9, 128.7, 128.0, 127.4, 126.5, 124.5, 123.5, 121.1, 116.5, 115.7, 113.7, 113.6, 35.1, 29.7, 27.9, 26.7, 23.7, 23.2 ppm.

(22a and 22b) R_f = 0.36 (22% ether in hexane, visualized by UV lamp).

(22a) HRMS = (ESI) *m/z*: $[M+H]^+$ calculated for $C_{13}H_{15}O$ 187.1117; Found 187.1119. **(22b) HRMS** = (ESI) *m/z*: $[M+H]^+$ calculated for $C_{13}H_{15}O$ 187.1117; Found 187.1119.

(*E*)-8-Allyl-7-hydroxy-3,4-dihydronaphthalen-1(2H)-one O-methyl oxime (24a) and (*E*)-6-Allyl-7-hydroxy-3,4-dihydronaphthalen-1(2H)-one O-methyl oxime (24b)

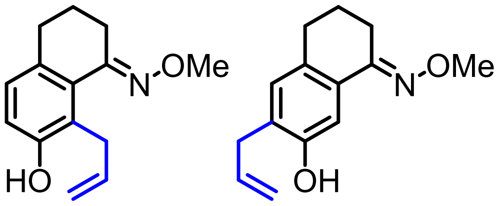

24a **24b**

Following the general procedure at 210 °C for five hours, **24a** and **24b** were observed as a 10:1 ratio with no purification to yield regioisomers **24a** and **24b** (90 mg, 99%) as a dark brown oil.

(24a) ^1H NMR (400 MHz, $CDCl_3$) δ_H 6.93 (1H, d, J = 8.0 Hz), 6.76 (1H, d, J = 8.0 Hz), 6.11 (1H, ddt, J_d = 16.7, 10.0 Hz, J_t = 6.2 Hz), 5.21 (1H, dq, J_d = 17.6 Hz, J_q = 1.8 Hz), 5.13 (1H, dq, J_d = 10.4 Hz, J_q = 1.6 Hz), 5.07 (1H, br s), 3.95 (3H, s), 3.79 (2H, dt, J_d = 6.3 Hz, J_t = 1.7 Hz), 2.71 (2H, t, J = 7.2 Hz), 2.56 (2H, t, J = 5.9 Hz), 1.72 (2H, quint, J = 6.1 Hz) ppm. **(24b) ^1H NMR** (400 MHz, $CDCl_3$) δ_H 7.38 (1H, s), 6.87 (1H, s), 6.03-5.94

(1H, m), 5.21 (1H, dq, J_d = 17.6 Hz, J_q = 1.8 Hz), 5.13 (1H, dq, J_d = 10.4 Hz, J_q = 1.6 Hz), 3.97 (3H, s), 3.37 (2H, d, J = 6.4 Hz), 2.68-2.60 (2H, m), 2.56 (2H, t, J = 5.9 Hz), 1.80 (2H, quint, J = 6.3 Hz) ppm.

(24a) ^{13}C{^{1}H} NMR (101 MHz, $CDCl_3$) δ 155.4, 154.3, 138.0, 134.8, 131.0, 127.1, 123.7, 116.5, 115.6, 62.1, 33.1, 30.6, 25.5, 21.6 ppm. **(24b) ^{13}C{^{1}H} NMR** (101 MHz, $CDCl_3$) δ 154.1, 152.4, 136.4, 132.4, 130.4, 129.9, 127.7, 110.3, 62.0, 34.8, 29.8, 28.9, 24.2, 21.8 ppm. **24b** is missing a carbon peak and is overlapping with one of the peaks in **24a**.

(24a and 24b) R_f = 0.46 (25% ethyl acetate in hexane, visualized by UV lamp).

(24a) HRMS = (ESI) *m/z*: $[M+H]^+$ calculated for $C_{14}H_{18}NO_2$ 232.1332; Found 232.1328. **(24b) HRMS** = (ESI) *m/z*: $[M+H]^+$ calculated for $C_{14}H_{18}NO_2$ 232.1332; Found 232.1327.

2-Allyl-3-hydroxy-N-methoxybenzamide (34a) and 4-Allyl-3-hydroxy-N-methoxybenzamide (34b)

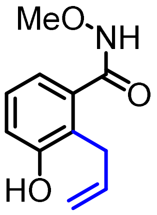

34a

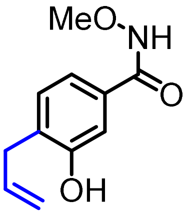

34b

Following the general procedure at 210 °C for three hours, **34a** and **34b** were observed as a 3.7:1 ratio before purification through isocratic column chromatography of 50% ethyl acetate in hexane to yield regioisomers **34a** and **34b** (17 mg, 17%) as a light yellow solid.

(34a) ^{1}H NMR (400 MHz, $CDCl_3$) δ_H 7.45 – 7.32 (1H, m), 7.10 (1H, t, J = 8.1 Hz), 6.60 (1H, d, J = 8.1 Hz), 5.96 (1H, ddt, J_d = 17.1, 10.2 Hz, J_t = 5.7 Hz), 5.13 (1H, br dd, J = 10.2, 1.5 Hz), 5.06 (1H, br dd, J = 17.2, 1.6 Hz), 3.76 (3H, s), 3.43 (2H, dt, J_d = 5.4 Hz, J_t = 1.5 Hz) ppm, N-H and O-H peaks not observed. **(34b) ^{1}H NMR** (400 MHz, MeOD) δ_H 7.02 (1H, br s), 6.88 (1H, d, J = 8.2 Hz), 6.67 (1H, dd, J = 8.2, 1.5 Hz), 5.92 (1H, ddt, J_d = 16.7, 10.0 Hz, J_t = 6.5 Hz), 5.00 – 4.90 (2H, m), 3.67 (3H, s), 3.24 (2H, br d, J = 6.5 Hz) ppm, N-H and O-H peaks not observed.

(34a) ^{13}C{^{1}H} NMR (125 MHz, MeOD) δ 168.1, 156.6, 137.6, 132.6, 132.1, 131.1, 118.8, 116.0, 114.7, 64.4, 35.2 ppm. **(34b) ^{13}C{^{1}H} NMR** (101 MHz, MeOD) δ 156.5, 156.3, 139.2, 138.7, 130.9, 122.5, 115.1, 111.1, 106.9, 52.4, 34.7 ppm.

(34a and 34b) R_f = 0.50 (70% ethyl acetate in hexane, visualized by UV lamp).

(34a) HRMS = (ESI) *m/z*: $[M+H]^+$ calculated for $C_{11}H_{14}NO_3$ 208.0974; Found 208.0966. **(34b HRMS** = (ESI) *m/z*: $[M+H]^+$ calculated for $C_{11}H_{14}NO_3$ 208.0974; Found 208.0965.

2-Allyl-3-hydroxy-N-propylbenzamide (29a) and 4-Allyl-3-hydroxy-N-propylbenzamide (29b)

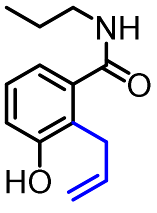

29a

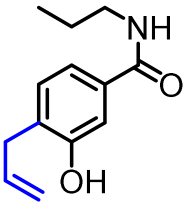

29b

Following the general procedure at 210 °C for nine hours, **29a** and **29b** were observed as a 1.8:1 ratio before purification through gradient column chromatography from 10% to 100% ethyl acetate in hexane to yield regioisomers **29a** and **29b** (73 mg, 73%) as a white solid.

(29a) ^{1}H NMR (400 MHz, MeOD) δ_H 7.03 (1H, t, J = 7.8 Hz), 6.80 (1H, d, J = 8.2 Hz), 6.75 (1H, d, J = 7.7 Hz), 5.90 (1H, ddt, J_d = 16.7, 10.2 Hz, J_t = 6.3 Hz), 4.92 (1H, br d, J = 17.3 Hz), 4.83 – 4.88 (1H, m), 3.46 (2H, br d, J = 6.3 Hz), 3.23 (2H, t, J = 7.1 Hz), 1.57 (2H, h, J = 7.3 Hz), 0.94 (3H, t, J = 7.4 Hz) ppm, N-H and O-H peaks not observed. **(29b) ^{1}H NMR** (400 MHz, MeOD) δ_H 7.18 (1H, s), 7.15 (1H, d, J = 7.9 Hz), 7.08 (1H, d, J = 7.8 Hz), 5.95 (1H, ddt, J_d = 16.7, 10.1 Hz, J_t = 6.6 Hz), 5.00 (1H, br d, J = 16.2

Hz), 4.97 (1H, br d, J = 8.5 Hz), 3.34 (2H, br d, J = 6.6 Hz), 3.27 – 3.20 (3H, m), 1.58 (2H, h, J = 7.4 Hz), 0.92 (3H, t, J = 7.4 Hz) ppm, O-H peak not observed.

(29a) ^{13}C{^{1}H} NMR (101 MHz, MeOD) δ 173.3, 156.9, 139.8, 138.1, 128.1, 125.1, 119.2, 117.3, 115.1, 42.5, 31.8, 23.6, 11.9 ppm. **(29b) ^{13}C{^{1}H} NMR** (101 MHz, MeOD) δ 170.4, 156.4, 137.7, 135.0, 131.8, 130.9, 118.9, 115.9, 114.8, 42.7, 35.1, 23.8, 11.7 ppm.

(29a) R_f = 0.30 (33% ethyl acetate in hexane, visualized by UV lamp). **(29b) R_f** = 0.20 (33% ethyl acetate in hexane, visualized by UV lamp).

(29a) HRMS = (ESI) *m/z*: [M+H]$^+$ calculated for $C_{13}H_{18}NO_2$ 220.1338; Found 220.1330. **(29b) HRMS** = (ESI) *m/z*: [M+H]$^+$ calculated for $C_{13}H_{18}NO_2$ 220.1338; Found 220.1329.

2-Allyl-N-cyclohexyl-3-hydroxybenzamide (30a) and 4-Allyl-N-cyclohexyl-3-hydroxybenzamide (30b)

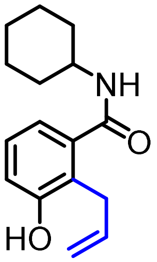

30a

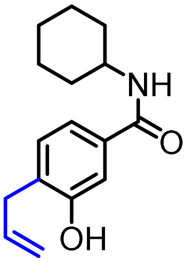

30b

Following the general procedure at 210 °C for 15 hours, **30a** and **30b** were observed as a 2:1 ratio before purification through gradient column chromatography from 5% to 20% ethyl acetate in hexane to yield regioisomers **30a** and **30b** (46 mg, 45%) as an off-white solid.

(30a) ^{1}H NMR (500 MHz, $CDCl_3$) δ_H 7.04 (1H, t, J = 7.8 Hz), 7.07-7.02 (1H, m), 6.93 (1H, d, J = 7.5 Hz), 6.86 (1H, d, J = 8.0 Hz), 6.10 – 5.93 (1H, ddt, J_d = 17.6, 10.4 Hz, J_t = 5.47 Hz), 5.88 (1H, d, J = 8.1 Hz), 5.10 – 5.04 (1H, m), 4.98 (1H, br d, J =17.2 Hz), 3.97 – 3.88 (1H, m), 3.50 (2H, d, J = 5.5 Hz), 2.05 – 1.94 (2H, m), 1.75 – 1.67 (2H, m), 1.65 – 1.58 (1H, m), 1.43 – 1.33 (2H, m), 1.27 – 1.12 (3H, m). **(30b) ^{1}H NMR** (500 MHz, $CDCl_3$) δ_H 7.81 (1H, br s), 7.47 (1H, s), 7.11-7.07 (2H, m), 6.11 (1H ,d, J = 8.1 Hz), 6.10 – 5.93 (1H, ddt, J_d = 18.1, 9.7 Hz, J_t = 6.5 Hz), 5.10 – 5.04 (2H, m), 3.97 – 3.88 (1H, m), 3.41 (2H, d, J = 6.5 Hz), 2.05 – 1.94 (2H, m), 1.75 – 1.67 (2H, m), 1.65 – 1.58 (1H, m), 1.43 – 1.33 (2H, m), 1.27 – 1.12 (3H, m).

(30a and 30b) ^{13}C{^{1}H} NMR (125 MHz, $CDCl_3$) δ 169.4, 167.4, 155.2, 155.1, 138.4, 137.3, 136.3, 133.9, 130.5, 130.1, 127.7, 122.9, 119.3, 117.7, 117.4, 116.2, 115.7, 114.9, 49.0, 48.9, 34.4, 33.2 (2C), 33.1 (2C), 31.5, 25.6, 25.6, 24.9 (2C), 24.9 (2C) ppm.

(30a and 30b) R_f = 0.40 (40% ethyl acetate in hexane, visualized by UV lamp).

(30a) HRMS = (ESI) *m/z*: [M+H]$^+$ calculated for $C_{16}H_{22}NO_2$ 260.1651; Found 260.1647. **(30b) HRMS** = (ESI) *m/z*: [M+H]$^+$ calculated for $C_{16}H_{22}NO_2$ 260.1651; Found 260.1647.

2-Allyl-3-hydroxy-N,N-dimethylbenzamide (31a) and 4-Allyl-3-hydroxy-N,N-dimethylbenzamide (31b)

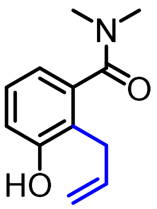

31a

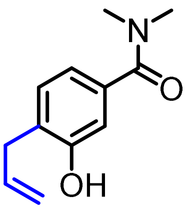

31b

Following the general procedure at 210 °C for 18 hours, **31a** and **31b** were observed as a 1.3:1 ratio before purification through isocratic column chromatography at 50% ethyl acetate in hexane to yield regioisomers **31a** and **31b** (138 mg, 94%) as a clear oil.

(31a) ^{1}H NMR (400 MHz, MeOD) δ_H 7.06 (1H, t, J = 7.6 Hz), 6.79 (1H, d, J = 8.0 Hz), 6.59 (1H, d, J = 8.0 Hz), 5.87 (1H, ddt, J_d = 16.8, 10.1 Hz, J_t = 6.6 Hz), 4.98-4.83 (3H, m), 3.39-3.22 (2H, m), 3.03 (3H, s), 2.75 (3H, s) ppm. **(31b) ^{1}H NMR** (400 MHz, MeOD) δ_H 7.09 (1H, d, J = 7.1 Hz), 6.76

(2H, t, J = 3.0 Hz), 5.95 (1H, ddt, J_d = 16.8 Hz, 10.1 Hz, J_t = 6.7 Hz), 5.05-4.95 (2H, m), 3.33 (2H, d, J = 7.5 Hz), 3.03 (3H, s), 2.96 (3H, s) ppm.

(31a) ^{13}C{^{1}H} NMR (101 MHz, MeOD) δ 173.7, 157.1, 138.7, 137.4, 128.6, 124.2, 117.9, 116.6, 115.3, 39.6, 34.9, 32.4 ppm. **(31b) ^{13}C{^{1}H} NMR** (101 MHz, MeOD) δ 173.9, 156.4, 137.8, 136.0, 131.0, 130.1, 118.9, 115.8, 114.3, 40.1, 35.6, 35.1 ppm.

(31a) R$_f$ = 0.64 (100% ethyl acetate in hexane, visualized by UV lamp). **(31b) R$_f$** = 0.53 (100% ethyl acetate in hexane, visualized by UV lamp).

(31a) HRMS = (ESI) *m/z*: $[M+H]^+$ calculated for $C_{12}H_{16}NO_2$ 206.1175; Found 206.1168. **(31b) HRMS** = (ESI) *m/z*: $[M+H]^+$ calculated for $C_{12}H_{16}NO_2$ 206.1175; Found 206.1167.

2-Allyl-3-hydroxy-N,N-diisopropylbenzamide (32a) and 4-Allyl-3-hydroxy-N,N-diisopropylbenzamide (32b)

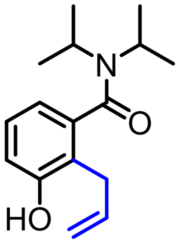

32a

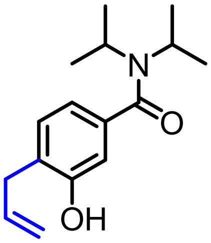

32b

Following the general procedure at 210 °C for nine hours, **32a** and **32b** were observed as a 1:1 ratio before purification through gradient column chromatography from 10% to 100% ethyl acetate in hexane to yield regioisomers **32a** and **32b** (66 mg, 60%) as a clear oil.

(32a) ^{1}H NMR (400 MHz, Acetone-d_6) δ_H 8.56 (1H, s), 7.05 (1H, t, J = 7.8 Hz), 6.83 (1H, d, J = 7.6 Hz), 6.63 (1H, d, J = 7.5 Hz), 6.05 – 5.92 (1H, m), 5.11 – 4.97 (1H, m), 4.90 (1H, d, J = 10.0 Hz), 3.83 – 3.50 (2H, m), 3.49 – 3.31 (2H, m), 1.40 – 1.20 (6H, m), 1.11 (6H, d, J = 6.7 Hz) ppm. **(32b) ^{1}H NMR** (400 MHz, Acetone-d_6) δ_H 8.65 (1H, s), 7.09 (1H, d, J = 7.6 Hz), 6.82 (1H, s), 6.72 (1H, d, J = 7.7 Hz), 6.05 – 5.92 (1H, m), 5.11 – 4.97 (2H, m), 3.83 – 3.50 (2H, m), 3.49 – 3.31 (2H, m), 1.54 (3H, d, J = 6.8 Hz), 1.52 (3H, d, J = 6.8 Hz), 1.40 – 1.20 (6H, m) ppm.

(32a and 32b) ^{13}C{^{1}H} NMR (101 MHz, Acetone-d_6) δ 171.1, 170.5, 156.6, 155.7, 141.0, 139.2, 137.7, 130.6, 127.9, 117.4, 117.0, 115.7, 115.5, 115.2, 113.2, 51.3, 46.0, 34.7, 32.3, 20.9 (4C), 20.8 (4C) ppm.

(32a) R$_f$ = 0.40 (33% ethyl acetate in hexane, visualized by UV lamp). **(32b) R$_f$** = 0.30 (33% ethyl acetate in hexane, visualized by UV lamp).

(32a) HRMS = (ESI) *m/z*: $[M+H]^+$ calculated for $C_{16}H_{24}NO_2$ 262.1808; Found 262.1799. **(32b HRMS** = (ESI) *m/z*: $[M+H]^+$ calculated for $C_{16}H_{24}NO_2$ 262.1808; Found 262.1799.

2-Allyl-3-hydroxy-N-methyl-N-phenylbenzamide (33a) and 4-Allyl-3-hydroxy-N-methyl-N-phenylbenzamide (33b)

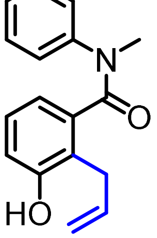

33a

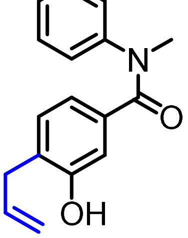

33b

Following the general procedure at 210 °C for nine hours, **33a** and **33b** were observed as a 1:1 ratio before purification through gradient column chromatography from 10% to 20% ethyl acetate in hexane to yield regioisomers **33a** and **33b** (101 mg, 84%) as a light yellow solid.

(33a) ^{1}H NMR (400 MHz, MeOD) δ_H 7.53 – 7.25 (1H, m), 7.15 (2H, d, J = 7.0 Hz), 7.11 (2H, d, J = 7.1 Hz), 6.75 (1H, t, J = 7.9 Hz), 6.96-6.71 (1H, m), 6.60 (1H, d, J = 8.2 Hz), 6.48 (1H, d, J = 7.6 Hz), 5.96 (1H, ddt, J_d = 16.7, 10.7 Hz,

J_t = 6.6 Hz), 5.08 (1H, br d, J = 17.1 Hz), 4.98 (1H, br d, J = 10.1 Hz), 3.46 (3H, s), 3.41 (2H, br d, J = 6.9 Hz) ppm. **(33b) ^{1}H NMR** (400 MHz, MeOD) δ_H 7.23 (2H, t, J = 7.6 Hz), 7.14 (1H, t, J = 7.4 Hz), 7.08 (2H, d, J = 7.7 Hz), 6.81 (1H, d, J = 7.7 Hz), 6.72 (1H, s), 6.59 (1H, d, J = 7.7 Hz), 5.91 – 5.79 (1H, m), 4.94 – 4.85 (2H, m), 3.40 (3H, s), 3.20 (2H, br d, J = 6.5 Hz) ppm.

(33a) ^{13}C{^{1}H} NMR (101 MHz, MeOD) δ 173.1, 156.8, 145.1, 138.5, 137.7, 129.9 (2C), 128.0 (2C), 127.5, 125.0, 120.2, 116.4, 115.6 (2C), 37.9, 33.0 ppm. **(33b) ^{13}C{^{1}H} NMR** (101 MHz, MeOD) δ 173.1, 155.8, 146.0, 137.6, 136.0, 130.3 (2C), 130.1, 128.1 (2C), 127.9, 120.8, 115.9, 115.8, 49.6, 38.9, 34.9 ppm.

(33a) R$_f$ = 0.40 (33% ethyl acetate in hexane, visualized by UV lamp). **(33b) R$_f$** = 0.30 (33% ethyl acetate in hexane, visualized by UV lamp).

(33a) HRMS = (ESI) *m/z*: $[M+H]^+$ calculated for $C_{17}H_{18}NO_2$ 268.1338; Found 268.1331. **(33b) HRMS** = (ESI) *m/z*: $[M+H]^+$ calculated for $C_{17}H_{18}NO_2$ 268.1338; Found 268.1331.

2-Allyl-3-hydroxybenzamide (28a) and 4-Allyl-3-hydroxybenzamide (28b)

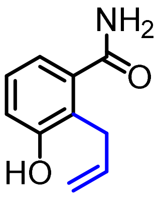

28a

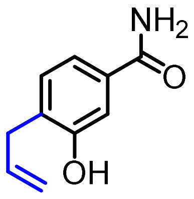

28b

Following the general procedure at 210 °C for 18 hours, **28a** and **28b** were observed as a 2.4:1 ratio before purification through gradient column chromatography from 0% to 5% MeOH in dichloromethane to yield regioisomers **28a** and **28b** (89 mg, 97%) as a light yellow solid.

(28a) ^{1}H NMR (400 MHz, MeOD) δ_H 7.04 (1H, t, J = 8.0 Hz), 6.84 (1H, d, J = 9.1 Hz), 6.82 (1H, d, J = 9.4 Hz), 5.94 (1H, ddt, J_d = 16.6, 10.4 Hz, J_t = 6.3 Hz), 5.00-4.82 (2H, m), 3.51 (2H, d, J = 6.4 Hz) ppm, O-H and N-H peaks not observed. **(28b) ^{1}H NMR** (400 MHz, MeOD) δ_H 7.30-7.18 (2H, m), 7.08 (1H, d, J = 7.8 Hz), 6.01-5.86 (1H, m), 5.04-4.87 (2H, m), 3.34 (2H, d, J = 6.9 Hz) ppm, O-H and N-H peaks not observed.

(28a and 28b) ^{13}C{^{1}H} NMR (101 MHz, MeOD) δ 175.9, 172.6, 156.9, 156.3, 138.9, 138.0, 137.6, 133.9, 132.3, 130.9, 128.1, 125.1, 119.5, 119.3, 117.5, 116.0, 115.2, 115.1, 35.1, 31.8 ppm.

(28a) R$_f$ = 0.30 (10% methanol in dichloromethane, visualized by UV lamp). **(28b) R$_f$** = 0.25 (10% methanol in dichloromethane, visualized by UV lamp).

(28a) HRMS = (ESI) *m/z*: $[M+H]^+$ calculated for $C_{10}H_{12}NO_2$ 178.0863; Found 178.0859. **(28b) HRMS** = (ESI) *m/z*: $[M+H]^+$ calculated for $C_{10}H_{12}NO_2$ 178.0863; Found 178.0863.

2-Allyl-3-hydroxybenzonitrile (35a) and 4-allyl-3-hydroxybenzonitrile (35b)

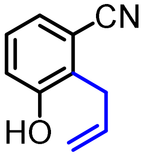

35a

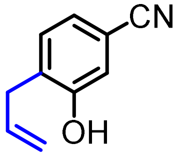

35b

Following the general procedure at 210 °C for 15 hours, **35a** and **35b** were observed as a 2.9:1 ratio with no purification to yield regioisomers **35a** and **35b** (100 mg, quantitative) as a light yellow solid.

(35a) ^{1}H NMR (400 MHz, $CDCl_3$) δ_H 7.26-7.16 (2H, m), 7.08 (1H, dd, J = 7.6, 1.9 Hz), 6.03 (1H, br s), 5.98 (1H, ddt, J_d = 17.6, 11.7, J_t = 5.9 Hz), 5.16 (1H, d, J = 1.5 Hz), 5.15-5.10 (1H, m), 3.64 (2H, dt, J_d = 6.0 Hz, J_t = 1.9 Hz) ppm. **(35b) ^{1}H NMR** (400 MHz, $CDCl_3$) δ_H 7.20 (1H, d, J = 7.6 Hz), 7.18 (1H, d, J = 7.9 Hz), 7.13 (1H, d, J = 1.6 Hz), 6.22 (1H, br s), 5.98 (1H, ddt, J_d = 16.8, 10.3 Hz, J_t = 6.5 Hz), 5.21-5.11 (2H, m), 3.45 (2H, dt, J_d = 6.6 Hz, J_t = 1.5 Hz) ppm.

(35a) ^{13}C{^{1}H} NMR (101 MHz, $CDCl_3$) δ 154.8, 134.3, 129.6, 128.4, 125.4, 120.7, 118.0, 117.1, 113.6, 33.2 ppm. **(35b) ^{13}C{^{1}H} NMR** (101 MHz, $CDCl_3$) δ 154.6, 135.0, 132.5, 131.2, 124.8, 118.9, 118.8, 118.7 (extra C peak due to rotamer issues), 117.5, 110.5, 34.8 ppm.

(35a) R_f = 0.42 (25% ethyl acetate in hexane, visualized by $KMnO_4$). **(35b) R_f** = 0.53 (25% ethyl acetate in hexane, visualized by $KMnO_4$).

(35a) HRMS = (ESI) *m/z*: $[M+H]^+$ calculated for $C_{10}H_{10}NO$ 160.0757; Found 160.0760. **(35b) HRMS** = (ESI) *m/z*: $[M+H]^+$ calculated for $C_{10}H_{10}NO$ 160.0757; Found 160.0759.

2-Allyl-3-hydroxy-N-phenylbenzamide (36a) and 4-Allyl-3-hydroxy-N-phenylbenzamide (36b)

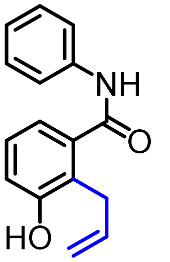

36a

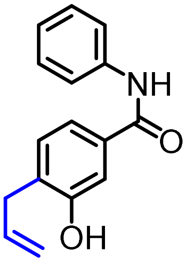

36b

Following the general procedure at 210 °C for 12 hours, **36a** and **36b** were observed as a 2.2:1 ratio before purification through gradient column chromatography from 15% to 20% ethyl acetate in hexane to yield regioisomers **36a** and **36b** (25.6 mg, 26%) as a yellow/orange solid.

(36a) ^{1}H NMR (500 MHz, $CDCl_3$) δ_H 7.68 (1H, br s), 7.59 (2H, d, J = 7.8 Hz), 7.36 (2H, t, J = 7.9 Hz), 7.20-7.10 (3H, m), 6.93 (1H, dd, J = 7.9, 1.2 Hz), 6.13 (1H, ddt, J_d = 17.1, 10.8 Hz, J_t = 5.5 Hz), 5.82 (1H, s), 5.18 (1H, dq, J_d = 10.3 Hz, J_q = 1.7 Hz), 5.08 (1H, dq, J_d = 17.4 Hz, J_q = 1.9 Hz), 3.59 (2H, dt, J_d = 5.7 Hz, J_t = 1.7 Hz) ppm. **(36b) ^{1}H NMR** (400 MHz, $CDCl_3$) δ_H 7.87 (1H, br s), 7.61 (2H, d, J = 7.9 Hz) 7.58 (1H, d, J = 1.7 Hz), 7.37 (2H, t, J = 7.9 Hz), 7.27 (1H, dd, J = 7.8, 1.9 Hz), 7.19 (1H, d, J = 7.9 Hz), 7.15 (1H, t, J = 7.4 Hz), 6.66 (1H, br s), 6.01 (1H, ddt, J_d = 16.8, 10.5 Hz, J_t = 2.0 Hz), 5.15 (1H, dt, J_d = 3.3 Hz, J_t = 1.7 Hz), 5.14-5.09 (1H, m), 3.45 (2H, d, J = 6.5 Hz) ppm.

(36a) ^{13}C{^{1}H} NMR (125 MHz, $CDCl_3$) δ 168.0, 155.1, 138.4, 137.8, 136.9, 129.3 (2C), 128.1, 124.8, 123.0, 120.0 (3C), 118.1, 116.3, 31.8 ppm. **(36b) ^{13}C{^{1}H} NMR** (101 MHz, $CDCl_3$) δ 166.1, 155.0, 137.7, 135.9, 134.2, 130.9, 130.6, 129.3 (2C), 124.9, 120.5 (2C), 118.2, 116.8, 115.2, 34.7 ppm.

(36a and 36b) R_f = 0.50 (40% ethyl acetate in hexane, visualized by UV lamp).

(36a) HRMS = (ESI) *m/z*: $[M+H]^+$ calculated for $C_{16}H_{16}NO_2$ 254.1181; Found 254.1177. **(36b) HRMS** = (ESI) *m/z*: $[M+H]^+$ calculated for $C_{16}H_{16}NO_2$ 254.1181; Found 254.1176.

2-Allyl-N-(2-bromo-5-fluorophenyl)-3-hydroxybenzamide (37a) and 4-Allyl-N-(2-bromo-5-fluorophenyl)-3-hydroxybenzamide (37b)

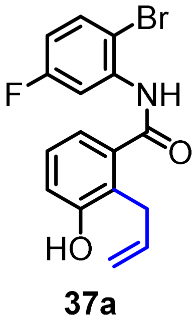

37a

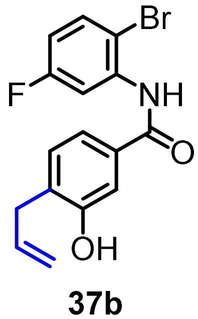

37b

Following the general procedure at 210 °C for nine hours, **37a** and **37b** were observed as a 2.4:1 ratio before purification through gradient column chromatography from 5% to 10% ethyl acetate in hexane to yield regioisomers **37a** and **37b** (18 mg, 15%) as a white solid.

(37a) ^{1}H NMR (400 MHz, MeOD) δ_H 7.70 (1H, d, J = 9.1 Hz), 7.64 (1H, dd, J = 8.9, 5.8 Hz), 7.15 (1H, t, J = 7.8 Hz), 7.05 (1H, d, J = 7.4 Hz), 6.97 – 6.89 (2H, m), 5.97 (1H, ddt, J_d = 16.6, 10.2 Hz, J_t = 6.3 Hz), 4.97 (1H, br dd, J = 17.2, 1.4 Hz), 4.91 (1H, br dd, J = 10.1, 1.4 Hz), 3.60 (2H, br d, J = 6.2 Hz) ppm, O-H and N-H peaks

not observed. **(37b) ^{1}H NMR** (400 MHz, MeOD) δ_H 7.79 (1H, dd, J = 10.4, 3.0 Hz), 7.64 (1H, dd, J = 8.9, 5.8 Hz), 7.38 – 7.32 (2H, m), 7.19 (1H, d, J = 8.2 Hz), 6.92 (1H, td, J_t = 8.4 Hz, J_d = 3.0 Hz), 5.98 (1H, ddt, J_d = 16.9, 10.1 Hz, J_t = 6.7 Hz), 5.09 – 4.97 (2H, m), 3.39 (2H, br d, J = 6.6 Hz) ppm, O-H and N-H peaks not observed.

(37a) ^{13}C{^{1}H} NMR (101 MHz, MeOD) δ171.1, 163.3 (d, $J_{C\text{-}F}$ = 245.1 Hz), 157.2, 138.5 (d, $J_{C\text{-}F}$ = 11.7 Hz), 138.0 (d, $J_{C\text{-}F}$ = 11.7 Hz), 135.0 (d, $J_{C\text{-}F}$ = 8.7 Hz), 134.9 (d, $J_{C\text{-}F}$ = 8.7 Hz), 128.4, 128.3, 125.9, 119.4, 118.1 (d, $J_{C\text{-}F}$ = 8.9 Hz), 115.1 (d, $J_{C\text{-}F}$ = 23.9 Hz), 115.0 (d, $J_{C\text{-}F}$ = 23.9 Hz), 114.2, 113.9, 112.9 (d, $J_{C\text{-}F}$ = 2.9 Hz), 31.5 ppm. Extra carbon peaks were observed due to rotamers. **(37b) ^{13}C{^{1}H} NMR** (101 MHz, MeOD) δ 168.2, 163.3 (d, $J_{C\text{-}F}$ = 245.2 Hz), 156.8, 138.8, 138.7, 137.5 (d, $J_{C\text{-}F}$ = 19.6 Hz), 134.8 (d, $J_{C\text{-}F}$ = 8.3 Hz), 134.8 (d, $J_{C\text{-}F}$ = 8.6 Hz), 134.2, 133.3 (d, $J_{C\text{-}F}$ = 19.1 Hz), 119.3 (d, $J_{C\text{-}F}$ = 13.2 Hz), 114.9 (d, $J_{C\text{-}F}$ = 29.8 Hz), 114.7 (d, $J_{C\text{-}F}$ = 29.8 Hz), 113.7, 113.4, 112.8 (d, $J_{C\text{-}F}$ = 3.1 Hz), 35.2 ppm. Extra carbon peaks were observed due to rotamers.

(37a) ^{19}F NMR (376.5 MHz, MeOD) δ -114.8 ppm. **(37b) ^{19}F NMR** (376.5 MHz, MeOD) δ -114.7 ppm.

(37a) R$_f$ = 0.50 (20% ethyl acetate in hexane, visualized by UV lamp). **(37b) R$_f$** = 0.40 (20% ethyl acetate in hexane, visualized by UV lamp).

(37a) HRMS = (ESI) *m/z*: $[M+H]^+$ calculated for $C_{16}H_{14}BrFNO_2$ 350.0193; Found 350.0188. **(37b) HRMS** = (ESI) *m/z*: $[M+H]^+$ calculated for $C_{16}H_{14}BrFNO_2$ 350.0193; Found 350.0187.

2-Allyl-3-hydroxy-N-(4-methoxyphenyl)benzamide (38a) and 4-Allyl-3-hydroxy-N-(4-methoxyphenyl)benzamide (38b)

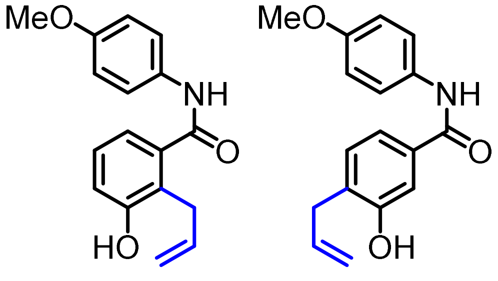

38a **38b**

Following the general procedure at 210 °C for six hours, **38a** and **38b** were observed as a 2.4:1 ratio with no purification to yield regioisomers **38a** and **38b** (88 mg, 73%) as an orange/yellow solid.

(38a) ^{1}H NMR (400 MHz, MeOD) δ_H 7.48 (2H, d, J = 9.0 Hz), 7.08 (1H, t, J = 7.8 Hz), 6.90 – 6.82 (4H, m), 6.04 – 5.85 (1H, m), 4.96 – 4.83 (2H, m), 3.73 (3H, s), 3.51 (2H, br d, J = 6.4 Hz) ppm, O-H and N-H peaks not observed. **(38b) ^{1}H NMR** (400 MHz, MeOD) δ_H 7.49 (2H, d, J = 9.0 Hz), 7.28 (2H, m), 7.13 (1H, d, J = 8.3 Hz), 6.90 – 6.82 (2H, m), 6.04 – 5.85 (1H, m), 5.02 (1H, br d, J = 16.5 Hz), 4.99 (1H, br d, J = 9.2 Hz), 3.73 (3H, s), 3.37 (2H, br d, J = 6.5 Hz) ppm, O-H and N-H peaks not observed.

(38a and 38b) ^{13}C{^{1}H} NMR (101 MHz, MeOD) δ 171.2, 168.8, 158.1, 158.1, 156.8, 156.4, 140.0, 137.9, 137.6, 135.3, 132.7, 132.2, 130.9, 128.2 (2C), 125.2, 124.1, 123.4 (2C), 119.3, 119.2 (2C), 117.4, 116.0, 115.3, 115.0, 114.9 (2C), 114.87, 55.8, 35.2, 31.9 ppm. Some peaks are missing due to overlapping signals between the regioisomers.

(38a and 38b) R$_f$ = 0.40 (33% ethyl acetate in hexane, visualized by UV lamp).

(38a) HRMS = (ESI) *m/z*: $[M+H]^+$ calculated for $C_{17}H_{18}NO_3$ 284.1287; Found 284.1283. **(38b) HRMS** = (ESI) *m/z*: $[M+H]^+$ calculated for $C_{17}H_{18}NO_3$ 284.1287; Found 284.1283.

2-Allyl-3-hydroxy-N-(3,4,5-trimethoxyphenyl)benzamide (39a) and 4-Allyl-3-hydroxy-N-(3,4,5-trimethoxyphenyl)benzamide (39b)

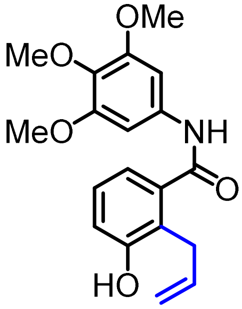

39a

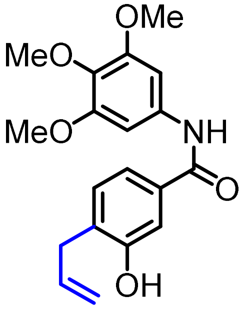

39b

Following the general procedure at 210 °C for nine hours, **39a** and **39b** were observed as a 2.9:1 ratio with no purification to yield regioisomers **39a** and **39b** (108 mg, 72%) as orange/tan crystals.

(39a) ^{1}H NMR (400 MHz, MeOD) δ_H 7.10 (1H, t, J = 7.8 Hz), 7.01 (2H, s), 6.88 (1H, d, J = 8.2 Hz), 6.86 (1H, d, J = 8.6 Hz), 5.96 (1H, ddt, J_d = 16.7, 10.1 Hz, J_t = 6.5 Hz), 4.97 – 4.83 (2H, m), 3.80 (6H, br s), 3.72 (3H, s), 3.51 (2H, br d, J = 6.4 Hz) ppm, O-H and N-H peaks not observed. **(39b) ^{1}H NMR** (400 MHz, MeOD) δ_H 7.29 (1H, d, J = 7.0 Hz), 7.28 (1H, s), 7.15 (1H, d, J = 7.6 Hz), 7.06 (2H, s), 5.96 (1H, ddt, J_d = 16.7, 10.1 Hz, J_t = 6.5 Hz), 5.03 (1H, br d, J = 16.2 Hz), 5.00 (1H, br d, J = 9.1 Hz), 3.80 (6H, br s), 3.72 (3H, s), 3.38 (2H, d, J = 6.6 Hz) ppm, O-H and N-H peaks not observed.

(39a and 39b) ^{13}C{^{1}H} NMR (101 MHz, MeOD) δ 171.3, 168.9, 156.9, 156.5, 154.5, 154.4, 140.0, 137.9, 137.6, 136.3, 135.7, 135.4, 132.4, 131.0, 128.2, 125.3, 119.3, 119.2, 117.5, 116.0, 115.3, 115.1, 99.8 (3C), 99.1 (3C), 61.2, 56.5, 35.2, 31.9 ppm. Some peaks are missing due to overlapping signals between the regioisomers.

(39a and 39b) R$_f$ = 0.60 (66% ethyl acetate in hexane, visualized by UV lamp).

(39a) HRMS = (ESI) *m/z*: $[M+H]^+$ calculated for $C_{19}H_{22}NO_5$ 344.1499; Found 344.1488. **(39b) HRMS** = (ESI) *m/z*: $[M+H]^+$ calculated for $C_{19}H_{22}NO_5$ 344.1499; Found 344.1488.

2-Allyl-N-(2,6-dimethylphenyl)-3-hydroxybenzamide (40a) and 4-Allyl-N-(2,6-dimethylphenyl)-3-hydroxybenzamide (40b)

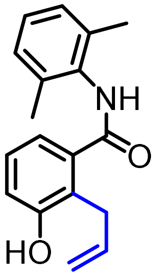

40a

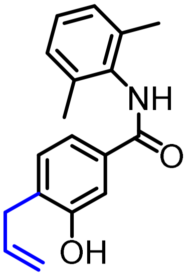

40b

Following the general procedure at 210 °C for 15 hours, **40a** and **40b** were observed as a 2.3:1 ratio before purification through gradient column chromatography from 10% to 25% ethyl acetate in hexane to yield regioisomers **40a** and **40b** (37 mg, 30%) as orange/tan crystals.

(40a) ^{1}H NMR (400 MHz, MeOD) δ_H 7.14 (1H, t, J = 7.7 Hz), 7.11 – 7.06 (4H, m), 6.90 (1H, d, J = 7.8 Hz), 6.01 (1H, ddt, J_d = 16.3, 10.3 Hz, J_t = 6.0 Hz), 5.00 – 4.87 (2H, m), 3.61 (2H, br d, J = 6.0 Hz), 2.30 (6H, s) ppm, O-H and N-H peaks not observed. **(40b) ^{1}H NMR** (400 MHz, MeOD) δ_H 7.37 (1H, dd, J = 7.8, 1.4 Hz), 7.33 (1H, d, J = 1.2 Hz), 7.17 (1H, d, J = 7.8 Hz), 7.09 (3H, s), 5.99 (1H, ddt, J_d = 16.8, 10.1 Hz, J_t = 6.6 Hz), 5.08 – 4.97 (2H, m), 3.39 (2H, br d, J = 6.4 Hz), 2.22 (6H, s) ppm, O-H and N-H peaks not observed.

(40a) ^{13}C{^{1}H} NMR (101 MHz, MeOD) δ 172.0, 157.3, 139.2, 138.2, 137.1 (2C), 135.4, 129.2 (2C), 128.5, 128.2, 125.8, 119.6, 117.7, 115.2, 31.7, 18.8 (2C) ppm. **(40b) ^{13}C NMR** (101 MHz, MeOD) δ 169.3, 156.6, 137.7, 137.2 (2C), 135.9, 134.7, 132.3, 131.0, 129.1 (2C), 128.5, 119.3, 116.0, 115.1, 35.2, 18.4 (2C) ppm.

(40a) R$_f$ = 0.30 (20% ethyl acetate in hexane, visualized by UV lamp). **(40b) R$_f$** = 0.20 (20% ethyl acetate in hexane, visualized by UV lamp).

(40a) HRMS = (ESI) *m/z*: $[M+H]^+$ calculated for $C_{18}H_{20}NO_2$ 282.1482; Found 282.1482. **(40b) HRMS** = (ESI) *m/z*: $[M+H]^+$ calculated for $C_{18}H_{20}NO_2$ 282.1482; Found 282.1481.

2-Allyl-3-hydroxy-N-(4-(methylsulfonyl)phenyl)benzamide (41a) and 4-Allyl-3-hydroxy-N-(4-(methylsulfonyl)phenyl)benzamide (41a)

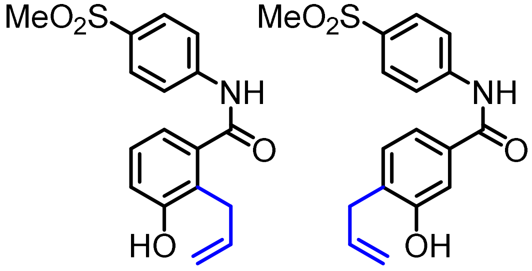

41a **41b**

Following the general procedure at 210 °C for nine hours, **41a** and **41b** were observed as a 5.1:1 ratio with no purification to yield regioisomers **41a** and **41b** (78 mg, 86%) as an orange oil.

(41a) ^{1}**H NMR** (400 MHz, MeOD) δ_H 8.01 – 7.83 (4H, m), 7.12 (1H, t, *J* = 7.8 Hz), 6.92 (1H, d, *J* = 8.0 Hz), 6.90 (1H, d, *J* = 8.5 Hz), 6.04 – 5.86 (1H, m), 4.94 – 4.80 (2H, m), 3.52 (2H, br d, *J* = 6.3 Hz), 3.09 (3H, s) ppm, O-H and N-H peaks not observed. **(41b)** ^{1}**H NMR** (400 MHz, MeOD) δ_H 8.01 – 7.83 (4H, m), 7.38 – 7.21 (2H, m), 7.17 (1H, d, *J* = 7.7 Hz), 6.04 – 5.86 (1H, m), 5.04 (1H, br d, *J* = 15.6 Hz), 5.01 (1H, br d, *J* = 8.9 Hz), 3.39 (2H, d, *J* = 6.5 Hz), 3.09 (3H, s) ppm, O-H and N-H peaks not observed.

(41a and 41b) ^{13}**C{**^{1}**H} NMR** (101 MHz, MeOD) δ 171.6, 169.1, 156.9, 156.5, 145.3, 145.1, 139.4, 137.8, 137.5, 136.5, 136.5, 134.8, 132.9, 131.0, 131.0, 129.6 (2C), 129.5, 129.4 (2C), 128.3, 127.7, 125.4, 121.5, 121.0 (2C), 119.5, 119.2, 117.8, 116.1, 115.3, 115.2, 44.5, 35.2, 31.7 ppm.

(41a and 41b) $\mathbf{R_f}$ = 0.40 (50% ethyl acetate in hexane, visualized by UV lamp).

(41a) HRMS = (ESI) *m/z*: $[M+H]^+$ calculated for $C_{17}H_{18}NO_4S$ 332.0957; Found 332.0949. **(41b) HRMS** = (ESI) *m/z*: $[M+H]^+$ calculated for $C_{17}H_{18}NO_4S$ 332.0957; Found 332.0949.

2-Allyl-3-hydroxy-N-(4-nitrophenyl)benzamide (42a) and 4-allyl-3-hydroxy-N-(4-nitrophenyl)benzamide (42b)

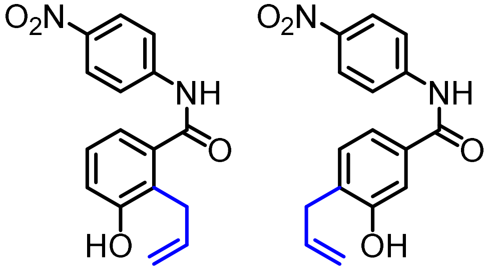

42a **42b**

Following the general procedure at 210 °C for nine hours, **42a** and **42b** were observed as a 2:1 ratio with no purification to yield regioisomers **42a** and **42b** (78 mg, 86%) as copper-like crystals.

(42a) ^{1}**H NMR** (400 MHz, MeOD) δ_H 8.15 (2H, d, *J* = 9.1 Hz), 7.83 (2H, d, *J* = 9.1 Hz), 7.09 (1H ,t, *J* = 7.8 Hz), 6.89 (1H, d, *J* = 7.2 Hz), 6.88 (1H, d, *J* = 7.5 Hz), 6.03 – 5.81 (1H, m), 4.92 – 4.78 (2H, m), 3.49 (2H, br d, *J* = 6.3 Hz) ppm, O-H and N-H peaks not observed. **(42b)** ^{1}**H NMR** (400 MHz, MeOD) δ_H 8.14 (2H, d, *J* = 9.1 Hz), 7.88 (2H, d, *J* = 9.1 Hz), 7.35 – 7.21 (2H, m), 7.12 (1H, d, *J* = 7.7 Hz), 6.03 – 5.81 (1H, m), 5.01 (1H, br d, *J* = 15.9 Hz), 4.98 (1H, br d, *J* = 9.1 Hz), 3.35 (2H, d, *J* = 6.6 Hz) ppm, O-H and N-H peaks not observed.

(42a and 42b) ^{13}**C{**^{1}**H} NMR** (101 MHz, MeOD) δ 171.6, 169.0, 156.9, 156.5, 146.3, 146.1, 144.6, 144.6, 139.2, 137.8, 137.4, 134.7, 132.9, 131.0, 128.3, 125.7 (2C), 125.6, 125.4, 121.1, 120.6 (2C), 119.6, 119.2, 117.9, 116.1, 115.3, 115.2, 35.2, 31.7 ppm. Some peaks are missing due to overlapping signals between the regioisomers.

(42a and 42b) $\mathbf{R_f}$ = 0.50 (33% ethyl acetate in hexane, visualized by UV lamp).

(42a) HRMS = (ESI) *m/z*: $[M+H]^+$ calculated for $C_{16}H_{15}N_2O_4$ 299.1032; Found 299.1025. **(42b) HRMS** = (ESI) *m/z*: $[M+H]^+$ calculated for $C_{16}H_{15}N_2O_4$ 299.1032; Found 299.1025.

2-allyl-3-hydroxy-6-methoxybenzonitrile (43a) and 4-allyl-5-hydroxy-2-methoxybenzonitrile (43b)

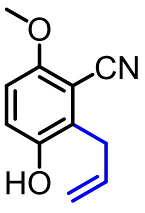

43a

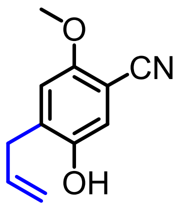

43b

Following the general procedure at 210 °C for 18 hours, **43a** and **43b** were observed as a 2.5:1 ratio before through gradient column chromatography from 10% to 40% ethyl acetate in hexane to yield regioisomers **43a** and **43b** (102 mg, 85%) as off-white and white solids, respectively.

(43a) ^{1}**H NMR** (400 MHz, $CDCl_3$) δ_H 7.02 (1H, d, J = 9.0 Hz), 6.74 (1H, d, J = 9.0 Hz), 5.97 (1H, ddt, J_d = 18.0, 9.7 Hz, J_t = 6.3 Hz), 5.19 (1H, s), 5.15 (1H, d, J = 5.3 Hz), 5.02-4.94 (1H, m), 3.87 (3H, s), 3.62 (2H, d, J = 6.2 Hz) ppm. **(43b)** ^{1}**H NMR** (400 MHz, $CDCl_3$) δ_H 7.03 (1H, s), 6.74 (1H, s), 5.97 (1H, ddt, J_d = 16.6, 10.2 Hz, J_t = 6.4 Hz), 5.54 (1H, s), 5.21-5.11 (2H, m), 3.87 (3H, s), 3.43 (2H, d, J = 6.6 Hz) ppm.

(43a) ^{13}**C{**^{1}**H} NMR** (101 MHz, $CDCl_3$) δ 156.4, 148.0, 134.1, 130.4, 121.7, 117.5, 115.5, 110.3, 102.9, 56.4, 33.6 ppm. **(43b)** ^{13}**C{**^{1}**H} NMR** (101 MHz, $CDCl_3$) δ 155.9, 147.8, 134.9, 134.0, 119.7, 117.6, 116.7, 113.5, 99.3, 56.5, 35.5 ppm.

(43a) $\mathbf{R_f}$ = 0.14 (20% ethyl acetate in hexane, visualized by $KMnO_4$). **(43b)** $\mathbf{R_f}$ = 0.27 (20% ethyl acetate in hexane, visualized by $KMnO_4$).

(42a) HRMS = (ESI) *m/z*: $[M+H]^+$ calculated for $C_{11}H_{12}NO_2$ 190.0863; Found 190.0862. **(42b) HRMS** = (ESI) *m/z*: $[M+H]^+$ calculated for $C_{11}H_{12}NO_2$ 190.0863; Found 190.0862.

Synthesis of 2,2-Dimethyl-6-((2-methylbut-3-yn-2-yl)oxy)-4H-benzo[d][1,3]dioxin-4-one (11)

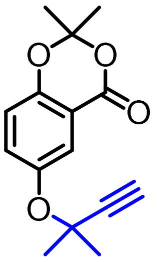

11

To a solution of **S3** (500 mg, 2.71 mmol) and copper(II) chloride (8.52 mg, 0.05 mmol) in anhydrous acetonitrile (5.2 mL, 0.52 M) under an argon atmosphere at 0 °C, 1,8-diazabicyclo[5.4.0]undec-7-ene (537 µL, 3.53 mmol) was added dropwise followed by dropwise addition of propargyl chloride (3-chloro-3-methylbut-1-yne, 250 µL, 2.44 mmol) over 5 h. The reaction was allowed to stir at 0 °C until completion monitored by thin layer chromatography. The crude mixture was concentrated under reduced pressure and extracted with toluene and water. The organic extract was washed with 2 M hydrochloric acid (3 times, 10 mL), saturated sodium bicarbonate (3 times, 10 mL) before it was dried with brine and anhydrous sodium sulfate. The organic extract was filtered, concentrated under reduced pressure, and purified via silica gel column chromatography with an eluent of 15% ether in pentane, to yield **11** (273 mg, 43% yield) as a yellow oil.

^{1}**H NMR** (400 MHz, $CDCl_3$) δ_H 7.78 (1H, d, J = 2.8 Hz), 7.38 (1H, dd, J = 8.9, 2.9 Hz), 6.88 (1H, d, J = 9.2 Hz), 2.59 (1H, s), 1.72 (6H, s), 1.63 (6H, s) ppm.

^{13}**C{**^{1}**H} NMR** (101 MHz, ($CDCl_3$) δ 161.2, 152.0, 150.4, 131.3, 122.0, 117.8, 113.6, 106.5, 85.5, 74.8, 73.6, 29.5 (2C), 25.9 (2C) ppm.

$\mathbf{R_f}$ = 0.37 (20% ethyl acetate in hexane, visualized by UV lamp).

HRMS = (ESI) *m/z*: $[M+H]^+$ calculated for $C_{15}H_{16}O_4$ 261.1121; Found 261.1118.

Synthesis of 6-(Buta-2,3-dien-1-yloxy)-2,2-dimethyl-4H-benzo[d][1,3]dioxin-4-one (9)

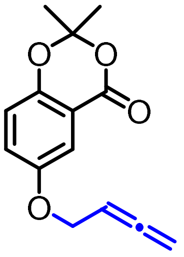

9

To a flame dried flask equipped with a stir bar, solid triphenylphosphine (456.3 mg, 1.74 mmol, 1.5 eq) was added followed by subsequent addition of 6-hydroxy-2,2-dimethyl-4*H*-benzo[*d*][1,3]dioxin-4-one (225.3 mg, 1.16 mmol, 1.0 eq) and buta-2,3-dien-1-ol (122.6 mg, 1.75 mmol, 1.5 eq) dissolved in dry and degassed tetrahydrofuran (3.31 mL, 0.35 M). The flask was sealed and flushed with N_2 before being cooled to 0 °C, and diisopropyl azodicarboxylate (351.84 mg, 341.59 µL, 1.74 mmol, 1.5 eq) was added dropwise. The resulting reaction mixture was allowed to warm to room temperature and left to stir overnight. Once the reaction was complete, as monitored by thin layer chromatography, the crude was concentrated and redissolved in ethyl acetate, washed with sodium hydroxide (2x, 2 M), DI water (2x), then dried with brine and sodium sulfate. The product was purified via column chromatography (10% isocratic EA/Hex, CV = 60 mL, product elutes after 2 CV) to yield **9** (87.3 mg, 0.35 mmol, 31% yield) as a clear oil.

^{1}H NMR (400 MHz, $CDCl_3$) 7.43 (1H, d, J = 3.1 Hz), 7.15 (1H, dd, J = 8.9, 3.1 Hz), 6.89 (1H, d, J = 9.0 Hz), 5.37 (1H, quint, J = 6.6 Hz), 4.89 (2H, dt, J_d = 6.6 Hz, J_t = 2.5 Hz), 4.57 (2H, dt, J_d = 6.8 Hz, J_t = 2.5 Hz), 1.72 (6H, s) ppm.

^{13}C{^{1}H} NMR (101 MHz, ($CDCl_3$) δ 209.8, 161.4, 153.6, 150.5, 125.7, 118.5, 113.8, 112.6, 106.6, 86.7, 77.4, 66.7, 25.8 (2C) ppm.

R_f = 0.51 (30% ethyl acetate in hexane, visualized by UV lamp).

HRMS = (ESI) *m/z*: $[M+H]^+$ calculated for $C_{14}H_{14}O_4$ 247.0975; Found 247.0961.

Synthesis of Buta-2,3-dien-1-ol (S5)

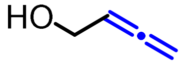

S5

Lithium aluminum hydride (3630 mg, 95.67 mmol) was added portion-wise to a flask of 4-chlorobut-2-yn-1-ol (1000 mg, 9.57 mmol) dissolved in anhydrous diethyl ether (5.5 mL, 0.45 M) at 0 °C. After addition was complete, the mixture was allowed to warm to room temperature and stirred for additional 30 minutes before it was cooled to 0 °C again and quenched by the addition of solid sodium sulfate decahydrate slowly until no more smoke is produced, followed by the addition of a saturated aqueous solution of potassium sodium tartrate tetrahydrate. The resulting slurry was left to stir overnight. The crude mixture was filtered through a pad of celite and rinsed with ether before being concentrated under reduced pressure. The concentrated filtrate containing **S5** was carried on to the next step without purification.

^{1}H NMR (400 MHz, $CDCl_3$) 5.36 (1H, quint, J = 6.3 Hz), 4.87 (2H, dt, J_d = 6.3, J_t = 3.0 Hz), 4.16 (2H, dt, J_d = 5.8, J_t = 3.0 Hz) ppm, confirmed with the literature source of *Org. Lett.* **2019**, *21*, 9672-9676.

Synthesis of 3-Bromo-2,5-dihydroxybenzoic acid (S6)

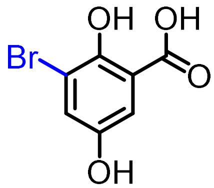

S6

Bromine (2.5 mL,48.6 mmol) was added to a stirred solution of 2,5-dihydroxybenzoic acid (5g, 32.4 mmol) in glacial acetic acid (200 mL, 0.16 M) at room temperature for 48 hours. Once completed, the reaction mixture was quenched with a solution of saturated sodium thiosulfate, and the acetic acid was removed under reduced pressure. The residue was acidified with 2M HCl, extracted with diethyl ether twice. The combined organic layers were dried over Na_2SO_4, and the solvent removed under reduced pressure. The off-white solids were washed with pentane, then dichloromethane and allowed to dry. Once dried, a 10% solution of acetone hexane was used to wash the solids. This yielded **S6** (2.6g, 35%) as flaky tan crystals. Referenced from Preece, L.; Studies Towards a Total Synthesis of Lactonamycin. Doctoral Dissertation. **2011**.

^{1}**H NMR** (400z MHz, Acetone-*d*$_6$) δ_H 11.19 (1H, s), 7.39 (1H, d, *J* = 3.2 Hz), 7.36 (1H, d, *J* = 3.2 Hz) ppm.

^{13}**C{^{1}H} NMR** (101 MHz, Acetone-*d*$_6$) δ 171.3, 152.2, 149.7, 126.8, 115.0, 113.2, 110.4 ppm.

m.p. range: 217.1 – 219.5 °C.

R$_f$ = 0.25 (20% ethyl acetate, 10% acetic acid in hexane, visualized by UV lamp).

HRMS = (ESI) *m/z*: $[M+H]^+$ calculated for $C_7H_5BrO_4$ 232.9450; Found 232.9453.

Synthesis of 6-Hydroxy-2,2-dimethyl-8-phenyl-4H-benzo[d][1,3]dioxin-4-one (S7)

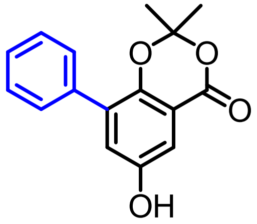

S7

Bromo lactone **S4** (150 mg, 0.48 mmol), Cs_2CO_3 (312.8 mg, 0.96 mmol), phenyl boronic acid (81.7 mg, 0.67 mmol), and $PdCl_2(PPh_3)_2$ (45.8 mg, 12.1 mol%) were dissolved in degassed dioxane (3.5 mL, 0.14 M) at room temperature. After stirring for one hour under reflux in an oil bath, the reaction was quenched with saturated aqueous NH_4Cl. The mixture was extracted with ethyl acetate three times, washed with bring once, then dried over Na_2SO_4 and concentrated under reduced pressure. The product purified through column chromatography with an eluent of 0% to 25% ethyl acetate in hexane to afford **S7** (87.6 mg, 63%) as an off-white/yellow solid.

^{1}**H NMR** (500 MHz, $CDCl_3$) δ_H 7.51-7.36 (6H, m), 7.17 (1H, d, *J* = 3.1 Hz), 1.72 (6H, s) ppm.

^{13}**C{^{1}H} NMR** (125 MHz, $CDCl_3$) δ 161.6, 150.6, 147.1, 135.6, 132.3, 129.1 (2C), 128.5 (2C), 128.1, 125.0, 114.8, 114.0, 106.5, 25.8 (2C) ppm.

R$_f$ = 0.2 (20% ethyl acetate/hexane, visualized by UV lamp).

HRMS = (ESI) *m/z*: $[M+H]^+$ calculated for $C_{16}H_{14}O_4$ 271.0965; Found 271.0963.

Synthesis of 5-(Allyloxy)-2-hydroxybenzoic acid (S8)

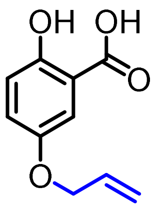

S8

To a suspension of NaH (1716 mg, 42.8 mmol) in dry DMF (18 mL), a solution of 2,5-dihydroxybenzoic acid (3000 mg, 19.5 mmol) in dry DMF (6 mL) was added dropwise. After 2 hours at room temperature, allyl bromide (1.7 mL, 19.5 mmol) was added as a solution in dry DMF (2.4 mL), dropwise. The mixture stirred for 2 hours at room temperature, then reduced to one-third the volume under vacuum. Cold water was added, and aqueous 3M HCl was used to adjust the pH to 3. The mixture was extracted with ethyl acetate three times. The combined organic fractions dried over sodium sulfate, filtered, and evaporated to give the crude mixture. The product was purified through column chromatography with an eluent of 0% to 10% ethyl acetate in hexane to yield **S8** (2995 mg, 79%) as a cream-colored solid.

^{1}**H NMR** (400 MHz, $CDCl_3$) δ_H 10.10 (1H, s), 7.37 (1H, d, *J* = 3.2 Hz), 7.17 (1H, dd, *J* = 9.1, 3.2 Hz), 6.95 (1H, d, *J* = 9.1 Hz), 6.05 (1H, ddt, J_d = 16.0, 10.6, J_t = 5.4 Hz), 5.42 (1H, d, *J* = 16.9), 5.30 (1H, d, *J* = 10.9), 4.52 (2H, d, *J* = 5.4 Hz) ppm.

^{13}**C{^{1}H} NMR** (101 MHz, $CDCl_3$) δ 175.0, 157.0, 151.3, 133.1, 126.5, 119.0, 118.1, 113.7, 110.9, 69.7 ppm.

R$_f$ = 0.39 (20% ethyl acetate, 80% hexane, 1% acetic acid, visualized by UV lamp).

HRMS = (ESI) *m/z*: $[M+H]^+$ calculated for $C_{10}H_{10}O_4$ 195.0657; Found 195.0657.

Synthesis of 5-(Allyloxy)-3-formyl-2-hydroxybenzoic acid (S9)

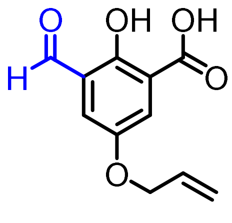

S9

To a solution of **S8** (624 mg, 3.21 mmol) in TFA (5.4 mL), a solution of hexamethylenetetramine (2.117 g, 15.1 mmol) in TFA (5.4 mL) was added. The reaction mixture was stirred at 90 °C in an oil bath for 16 hours. After completion, heating was stopped and 2M HCl was added, and the solution stirred for 3 hours at room temperature. The organic product was extracted with ethyl acetate three times. The combined organic layers were dried over sodium sulfate, and solvent removed under vacuum. Product purified through column chromatography with an eluent of 0% to 25% ethyl acetate in hexane with 1% acetic acid to yield **S9** (341.1 mg, 48%) as a yellow solid.

^{1}H NMR (400 MHz, $CDCl_3$) δ_H 11.51 (1H, br s), 10.26 (1H, s), 7.85 (1H, d, J = 3.3 Hz), 7.55 (1H, d, J = 3.3 Hz), 6.03 (1H, ddt, J_d = 16.0, 10.5 Hz, J_t = 5.3 Hz), 5.43 (1H, d, J = 17.3 Hz), 5.33 (1H, d, J = 10.7 Hz), 4.58 (2H, d, J = 5.1 Hz) ppm.

^{13}C{^{1}H} NMR (125 MHz, $CDCl_3$) δ 191.2, 170.2, 157.6, 151.4, 132.5, 124.7, 123.8, 122.4, 118.6, 114.9, 69.9 ppm.

R_f = 0.42 (20% ethyl acetate, 80% hexane, 1% acetic acid, visualized by UV lamp).

HRMS = (ESI) *m/z*: $[M+H]^+$ calculated for $C_{11}H_{11}O_5$ 233.0601; Found 223.0609.

Synthesis of 6-Hydroxy-2,2-dimethyl-8-((trimethylsilyl)ethynyl)-4H-benzo[d][1,3]dioxin-4-one (S10)

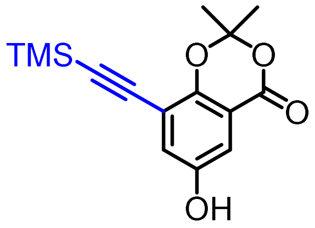

S10

Bromo lactone **S4** (100 mg, 0.366 mmol) was added to triethylamine (3.7 mL) under N_2 atmosphere. The mixture was degassed with N_2 for 15 minutes. CuI (7 mg, 0.1 mmol), $Pd(PPh_3)_2Cl_2$ (25.7 mg, 0.1 mmol), and TMS acetylene (77.2 µL, 0.549 mmol) were added sequentially, and the reaction mixture was stirred at 60 °C in an oil bath for 1 hour. The mixture was diluted with ethyl acetate and filtered through celite. The filtrate was washed with solutions of 1 M HCl, saturated sodium bicarbonate and brine. The organic layer was dried with Na_2SO_4 and concentrated *in vacuo*. The product was purified through column chromatography with an eluent of 0% to 20% ethyl acetate in hexane to yield **S10** (28.8 mg, 27%) as an orange solid.

^{1}H NMR (400 MHz, $CDCl_3$) δ_H 7.47 (1H, d, J = 3.4 Hz), 7.22 (1H, d, J = 3.2 Hz), 6.45 (1H, br s), 1.75 (6H, s), 0.25 (9H, s) ppm.

^{13}C{^{1}H} NMR (101 MHz, $CDCl_3$) δ 161.8, 150.9, 150.7, 127.8, 115.6, 114.2, 113.9, 107.3, 101.2, 98.0, 25.9 (2C), 0.0 (3C) ppm.

R_f = 0.81 (50% ethyl acetate, visualized by UV lamp).

HRMS = (ESI) *m/z*: $[M+H]^+$ calculated for $C_{15}H_{19}O_4Si$ 291.1047; Found 291.1052.

Synthesis of 8-Ethynyl-6-hydroxy-2,2-dimethyl-4H-benzo[d][1,3]dioxin-4-one (S11)

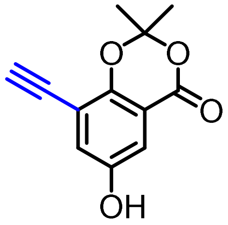

S11

Protected alkyne **S10** (24 mg, 0.0826 mmol) was dissolved in THF (1 mL, 0.08 M), and 1M TBAF in THF (91 µL, 0.091 mmol) was added to the reaction mixture at room temperature. The solution stirred for 15 minutes. After the removal of solvent *in vacuo*, the product was purified by column chromatography with an eluent of 10% to 35% ethyl acetate in hexane to yield alkyne **S11** (10.5 mg, 58%) as an off-white solid.

^{1}H NMR (400 MHz, Acetone-d_6) δ_H 8.81 (1H, s), 7.35 (1H, d, J = 2.9 Hz), 7.24 (1H, d, J = 3.2 Hz), 3.93 (1H, s), 1.72 (6H, s) ppm.

^{13}C{^{1}H} NMR (101 MHz, Acetone-d_6) δ 160.5, 152.8, 151.0, 127.7, 116.1, 115.6, 113.8, 107.4, 84.8, 77.9, 25.8 (2C) ppm.

R$_f$ = 0.47 (25% ethyl acetate, visualized by UV lamp).

HRMS = (ESI) *m/z*: [M+H]$^+$ calculated for $C_{12}H_{11}O_4$ 219.0652; Found 219.0656.

Synthesis of 8-(1-Benzyl-1H-1,2,3-triazol-4-yl)-6-hydroxy-2,2-dimethyl-4H-benzo[d][1,3]dioxin-4-one (S12)

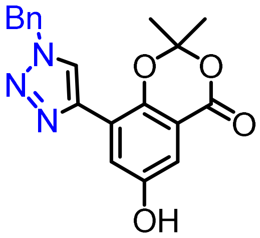

S12

To a solution of **S11** (10 mg, 0.0458 mmol) and azide (6.9 µL, 0.0550 mmol) in a 1:1 mixture of THF/H_2O (0.7 mL, 0.07 M) in a reaction vial, a catalytic amount of $CuSO_4$ (1.5 mg, 0.0096 mmol) and sodium ascorbate (8.1 mg, 0.0458 mmol) were added sequentially at room temperature. After stirring until completion determined by TLC, the reaction mixture was diluted with ethyl acetate and the organic layer was extracted. The organic layer was then dried over Na_2SO_4 and concentrated *in vacuo*. The crude extract was purified through silica gel chromatography with an eluent of 50% to 70% ethyl acetate in hexane yielded **S12** as a yellow solid (14.1 mg, 86%).

^{1}H NMR (400 MHz, DMSO-d_6) δ_H 9.87 (1H, s), 8.55 (1H, s), 7.87 (1H, d, J = 3.2 Hz), 7.41-7.31 (5H, m), 7.18 (1H, d, J = 3.2 Hz), 5.69 (2H, s), 1.74 (6H, s) ppm.

^{13}C{^{1}H} NMR (101 MHz, DMSO-d_6) δ 160.2, 152.5, 144.3, 139.9, 136.1, 128.8 (2C), 128.1, 127.8 (2C), 124.5, 120.9, 120.0, 114.5, 113.4, 106.5, 52.8, 25.3 (2C) ppm.

R$_f$ = 0.42 (50% ethyl acetate in hexane, visualized by UV lamp).

HRMS = (ESI) *m/z*: [M+H]$^+$ calculated for $C_{19}H_{18}N_3O_4$ 352.1292; Found 352.1290.

Synthesis of 7-Hydroxy-1-tetralone (S13)

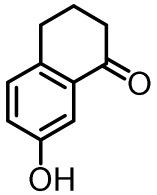

S13

To a dry round bottom flask containing aluminum chloride (7.6 g, 59.8 mmol) in toluene (20 mL), the starting material, 7-methoxy-1-tetralone (2 g, 11.4 mmol), were added slowly. The reaction mixture was refluxed at 108 ˚C in an oil bath for 1 hour then quenched with cool water slowly at about 90 ˚C (13 mL, 720 mmol). The reaction mixture was extracted using water and ethyl acetate three times. The combined organic extracts were extracted using 20% w/v NaOH (aq.) solution (× 3), and the combined aqueous extracts were then acidified to pH ~2 using 6 M HCl (aq.) solution, causing large amounts of the phenol product to precipitate. The acidified aqueous mixture was extracted using ethyl acetate (× 3), washed with brine, dried over Na_2SO_4, evaporated under reduced pressure,

washed using hexanes (× 3), then further dried under vacuum to yield **S13** as a pale-brown, semicrystalline solid powder (1.8 g, 98%).

^{1}H NMR (400 MHz, Acetone-d_6) δ_H 8.46 (1H, s), 7.37 (1H, d, J = 2.7 Hz), 7.16 (1H, d, J = 8.3 Hz), 7.00 (1H, dd, J = 8.4, 2.7 Hz), 2.86 (2H, t, J = 6.8 Hz), 2.53 (2H, t, J = 6.5 Hz), 2.08-2.01 (2H, m) ppm, confirmed with the literature source *J. Med. Chem.* **1998**, *41*, 1068-1083.

Synthesis of 7-(Allyloxy)-1-methylene-1,2,3,4-tetrahydronaphthalene (20)

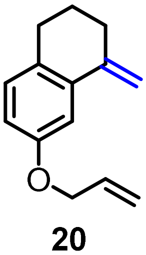

20

To a round bottom flask containing THF (27 mL, 0.09 M), Wittig salt (3.341 g, 6.8 mmol) and NaH (163.53 mg, 6.8 mmol) were added and the mixture was stirred for an hour at room temperature. 7-(Allyloxy)-3,4-dihydronaphthalen-1(2H)-one, dissolved in 1-2 mL THF was then added to the round bottom flask and the resulting reaction mixture was then stirred for 12 hours. The resulting mixture was extracted with ethyl acetate, washed with water, and purified on silica gel column chromatography (isocratic elution of 10% ethyl acetate in hexanes). This yielded **20** (330 mg, 95 % yield) as a red oil.

^{1}H NMR (400 MHz, $CDCl_3$) δ_H 7.18 (1H, d, J = 2.7 Hz), 7.01 (1H, dt, J_d = 8.4 Hz, J_t = 1.0 Hz), 6.77 (1H, dd, J = 8.4, 2.7 Hz), 6.07 (1H, ddt, J_d = 17.3, 10.6 Hz, J_t = 5.4 Hz), 5.45-5.39 (2H, m), 5.29 (1H, dq, J_d = 10.5 Hz, J_q = 1.4 Hz), 4.95 (1H, q, J = 1.5 Hz), 4.54 (2H, dt, J_d = 5.4 Hz, J_t = 1.6 Hz), 2.78 (2H, t, J = 6.5 Hz), 2.54-2.49 (2H, m), 1.86 (2H, quint, J = 6.3 Hz) ppm.

^{13}C{^{1}H} NMR (101 MHz, $CDCl_3$) δ 156.9, 143.7, 135.7, 133.6, 130.2, 130.2, 117.8, 115.2, 109.8, 108.2, 69.1, 33.3, 29.8, 24.2 ppm.

R_f = 0.83 (20% ethyl acetate in hexane, visualized by UV lamp).

HRMS = (ESI) *m/z*: $[M+H]^+$ calculated for $C_{14}H_{16}O$ 201.1274, Found 201.1278.

Synthesis of 7-(Benzyloxy)-3,4-dihydronaphthalen-1(2H)-one (S14)

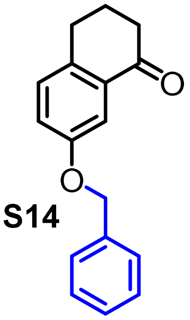

S14

To a dry microwave vial, potassium carbonate (400 mg, 2 mmol) was added to **S13** (400 mg, 2 mmol), dissolved in acetone (11 mL, 0.2 M) along with a catalytic amount of sodium iodide. Benzyl bromide (323 µL, 2.70 mmol) was added to the reaction mixture last, and the reaction was refluxed at 54 °C in an oil bath for 18 hours. The resulting reaction was extracted with ethyl acetate, washed with water, and purified by silica gel column chromatography (isocratic elution of 15% ethyl acetate in hexanes) to yield **S14** (589.9 mg, 95% yield) as a light orange solid.

^{1}H NMR (400 MHz, $CDCl_3$) δ_H 7.60 (1H, d, J = 2.6 Hz), 7.1 – 7.44 (7H, m), 5.08 (2H, s), 2.89 (2H, t, J = 6.2 Hz), 2.63 (2H, t, J = 6.6 Hz), 2.19 – 2.05 (2H, m) ppm, confirmed with the literature source *Tetrahedron. Lett.* **2014**, *55*, 682-685.

Synthesis of 7-(Benzyloxy)-1-methylene-1,2,3,4-tetrahydronaphthalene (S15)

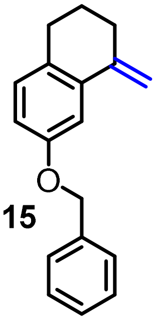

S15

In a dry round bottom flask, the Wittig salt (3.34 g, 9.35 mmol), and potassium *tert*-butoxide (1.05 g, 9.35 mmol) was stirred for an hour in tetrahydrofuran (27 mL, 0.09 M). **S14** (590 mg, 2.3 mmol) was then added to the reaction mixture and this was capped and stirred at room temperature for two days. The resulting reaction was extracted with ethyl acetate, washed with water and purified by silica gel column chromatography (isocratic elution of 15% ethyl acetate in hexanes) to yield **S15** (583.8 mg, 99 % yield) as a deep yellow oil.

^{1}H NMR (400 MHz, $CDCl_3$) δ_H 7.48-7.30 (5H, m), 7.26 (1H, d, J = 2.9 Hz), 7.03 (1H, d, J = 8.5 Hz), 6.85 (1H, dd, J = 8.4, 2.5 Hz), 5.44 (1H, s), 5.08 (2H, s), 4.96 (1H, q, J = 1.4 Hz), 2.79 (2H, t, J = 6.7 Hz), 2.53 (2H, dt, J_d = 6.1, J_t = 1.2 Hz), 1.87 (2H, quint, J = 6.1 Hz) ppm.

^{13}C{^{1}H} NMR (101 MHz, $CDCl_3$) δ 157.1, 143.6, 137.3, 135.7, 130.3, 128.7 (2C), 128.1, 127.7 (2C), 115.2, 109.9, 108.3, 70.2, 33.3, 29.8, 24.1 ppm.

R_f = 0.81 (10% ethyl acetate in hexane, visualized by UV lamp).

HRMS = (ESI) *m/z*: $[M+H]^+$ calculated for $C_{18}H_{19}O$ 251.1430; Found 251.1428.

Synthesis of 8-Methyl-5,6,7,8-tetrahydronaphthalen-2-ol (S16)

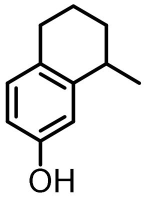

S16

To a small round bottom flask, **S15** (580 mg, 2.3 mmol), palladium (10%) on carbon (320 mg, 3.0 mmol) and methanol (80 mL, 0.3 M) were combined under nitrogen gas. The walls of the round bottom flask were washed with methanol to ensure that the palladium on carbon was completely suspended in methanol before replacing the nitrogen balloon with two hydrogen balloons. The reaction mixture was stirred for 18 hours at room temperature. The palladium was filtered out using a Buchner funnel topped with filter paper and an inch thick layer of celite and subsequently filtered through cotton. This resulted in **S16** being formed (330 mg, 89 % yield) as a yellow liquid.

^{1}H NMR (400 MHz, $CDCl_3$) δ_H 6.91 (1H, d, J = 8.2 Hz), 6.67 (1H, d, J = 2.4 Hz), 6.58 (1H, dd, J = 8.2, 2.7 Hz), 4.59 (1H, s), 2.88 – 2.58 (3H, m), 1.95 – 1.38 (4H, m), 1.26 (3H, d, J = 6.0 Hz) ppm.

^{13}C{^{1}H} NMR (125 MHz, $CDCl_3$) δ 153.4, 143.8, 130.1, 129.3, 114.5, 112.9, 32.7, 31.5, 29.3, 22.9, 20.8 ppm.

R_f = 0.88 (20% ethyl acetate in hexane, visualized by UV lamp).

HRMS = (ESI) *m/z*: $[M+H]^+$ calculated for $C_{11}H_{15}O$ 163.1117; Found 163.1116.

Synthesis of 7-(Allyloxy)-1,2,3,4-tetrahydronaphthalen-1-ol (23)

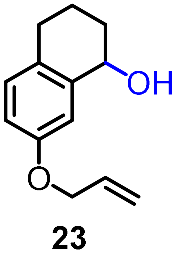

23

In a round bottom flask, **21** (80 mg, 0.396 mmol) was dissolved in dry methanol (2.47 mL, 0.16 M) and cooled down to 0 °C. $NaBH_4$ (35.5 mg, 0.938 mmol) was added to this mixture carefully and allowed to stir for two hours at the same temperature. The ice bath was removed to warm up to room temperature, and slowly quenched with ethanol followed by DI water. The organics were extracted with ethyl acetate three times, dried with Na_2SO_4 and the solvent removed under vacuum. The product was purified through silica gel column chromatography with an isocratic eluent of 30% ethyl acetate in hexane to afford **23** as an off-white oil (81 mg, quant.).

^{1}H NMR (400 MHz, $CDCl_3$) δ_H 7.02-6.98 (2H, m), 6.80 (1H, dd, J = 8.4, 2.7 Hz), 6.06 (1H, ddt, J_d = 16.9, 10.5 Hz, J_t= 5.3 Hz), 5.41 (1H, dq, J_d = 17.3 Hz, J_q = 1.3 Hz), 5.28 (1H, dq, J_d = 10.5 Hz, J_q = 1.4 Hz), 4.74 (1H, t, J = 4.9 Hz), 4.53 (2H, dt, J_d = 5.3, J_t = 1.5 Hz), 2.76 (1H, dt, J_d = 16.5 Hz, J_t = 5.8 Hz), 2.66 (1H, dt, J_d = 17.0 Hz, J_t = 6.6 Hz), 2.05 – 1.50 (4H, m), 1.61 (1H, br s) ppm.

^{13}C{^{1}H} NMR (101 MHz, $CDCl_3$) δ 157.1, 139.9, 133.5, 130.1, 129.5, 117.7, 115.2, 113.8, 69.0, 68.6, 32.5, 28.6, 19.3 ppm.

R_f = 0.21 (15% ethyl acetate in hexane, visualized by UV lamp).

HRMS = (ESI) *m/z*: $[M+H]^+$ calculated for $C_{13}H_{17}O_2$ 205.1223; Found 205.1231.

Synthesis of 7-(Allyloxy)-1,2,3,4-tetrahydronaphthalen-1-yl acetate (22)

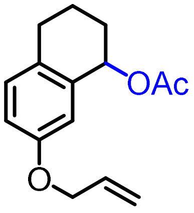

22

To a solution of **23** (200 mg, 0.979 mmol), in pyridine (excess), and dry THF (3.3 mL, 0.3 M), acetic anhydride (277.7 µL, 2.94 mmol) was added dropwise over 5 minutes and allowed to stir at room temperature for 22 hours. Once completed and determined by TLC analysis, the reaction was quenched with 1M $HCl_{(aq)}$ and extracted three times with diethyl ether. The organic extracts were dried with Na_2SO_4 and concentrated under vacuum to isolate crude mixture, which was purified through silica gel column chromatography with an eluent of 20% to 30% ether in hexane to yield **22** as an off-white oil (198.3 mg, 82%).

^{1}H NMR (400 MHz, $CDCl_3$) δ_H 7.04 (1H, m), 6.85-6.81 (2H, m), 6.04 (1H, ddt, J_d = 17.4, 10.6 Hz, J_t = 5.4 Hz), 5.95 (1H, t, J = 4.1 Hz), 5.41 (1H, dq, J_d = 17.2 Hz, J_q = 1.8 Hz), 5.28 (1H, dq, J_d = 10.4 Hz, J_q = 1.5 Hz), 4.50 (2H, dt, J_d = 5.3 Hz, J_t = 1.6 Hz), 2.79 (1H, dt, J_d = 16.8 Hz, J_t = 5.0 Hz), 2.67 (1H, dt, J_d = 17.0 Hz, J_t = 8.5 Hz), 2.09 (3H, s), 2.00-1.75 (4H, m) ppm.

^{13}C{^{1}H} NMR (101 MHz, $CDCl_3$) δ 171.0, 156.9, 135.5, 133.4, 130.3, 130.1, 117.8, 115.6, 114.7, 70.3, 69.0, 29.2, 28.3, 21.6, 19.1 ppm.

R_f = 0.69 (40% diethyl ether in hexane, visualized by $KMnO_4$ stain).

HRMS = The parent ion was not observed by positive or negative mode, but the fragment without the acetate group was observed, indicative of the McLafferty Rearrangement. (ESI) *m/z* $[M+H]^+$ calculated for $C_{13}H_{15}O$ 187.1117, found 187.1117.

Synthesis of 3-Allyloxybenzoic acid (S17)

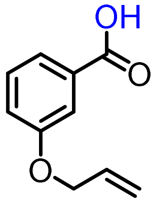

S17

To a solution of **S2** (1.1 g, 6.0 mmol) in a 1:1 mixture of MeOH and H_2O (25 mL, 0.13 M), NaOH (480 mg, 12.0 mmol) was added, and the reaction mixture was refluxed for three hours. The reaction mixture was then concentrated *in vacuo* to remove the MeOH and the aqueous layer was acidified with 10% HCl to a pH of 2. The reaction mixture was extracted with ethyl acetate three times and the combined organic fractions were washed with water and brine. The organic layer was dried with Na_2SO_4, filtered off, and the ethyl acetate was concentrated under reduced pressure to yield **S17** as a white solid (1.0 g, 94%).

^{1}H NMR (400 MHz, $CDCl_3$) δ_H 7.72 (1H, dt, J_d = 7.5 Hz, J_t = 1.3 Hz), 7.63 (1H, apparent t, J = 1.8 Hz), 7.38 (1H, t, J = 7.9 Hz), 7.18 (1H, dd, J = 8.2, 2.7 Hz), 6.07 (1H, ddt, J_d = 17.3, 10.5 Hz, J_t = 5.2 Hz), 5.45 (1H, dq,

J_d = 17.2 Hz, J_q = 1.6 Hz), 5.32 (1H, dq, J_d = 10.5 Hz, J_q = 1.5 Hz), 4.61 (2H, dt, J_d = 5.5 Hz, J_t = 1.5 Hz) ppm, confirmed with the literature source *Tetrahedron Lett.*, **2007**, *48,* 7380-7382.

Synthesis of 3-(Allyloxy)benzamide (28)

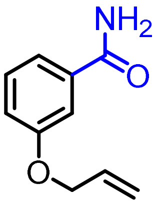

28

Acid **S17** (250 mg, 1.4 mmol), was dissolved in dry THF (4.2 mL, 0.33 M) in a round bottom flask with a magnetic stir bar. Thionyl chloride (153 µL, 2.1 mmol) was added dropwise to the solution and the solution was warmed to 50 °C in an oil bath for one hour. The reaction was sequentially cooled to room temperature and 0 °C, at which point ice-cold ammonium hydroxide (1.4 mL, 10.8 mmol) was added slowly to the solution and stirred for five minutes. The reaction mixture was extracted with $CHCl_3$ three times. The organic extracts were dried with Na_2SO_4 and concentrated down to afford a resulting solid. The solids were washed with hexane to afford **28** as a white solid (136 mg, 55%).

^{1}H NMR (400 MHz, Acetone-*d$_6$*) δ_H 7.51 (2H, d, J = 6.0 Hz), 7.36 (1H, t, J = 8.0 Hz), 7.10 (1H, dd, J = 8.1, 1.4 Hz), 6.70 (1H, br s), 6.08 (1H, ddt, J_d = 16.6, 11.0 Hz, J_t = 5.3 Hz), 5.43 (1H, d, J = 16.8 Hz), 5.25 (1H, d, J = 10.9 Hz), 4.62 (2H, d, J = 5.2 Hz), 2.92 (1H, br s) ppm.

^{13}C{^{1}H} NMR (101 MHz, Acetone-*d$_6$*) δ 168.7, 159.6, 136.9, 134.6, 130.2, 120.7, 118.7, 117.5, 114.4, 69.4 ppm.

R_f = 0.95 (30% methanol in dichloromethane, visualized by UV lamp).

HRMS = (ESI) *m/z*: $[M+H]^+$ calculated for $C_{10}H_{12}NO_2$ 178.0863; Found 178.0857.

Synthesis of 5-(2,3-Dihydroxypropyl)-6-hydroxy-2,2-dimethyl-4H-benzo[d][1,3]dioxin-4-one (S18)

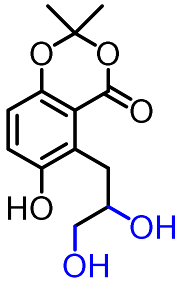

S18

Phenol **1a** (20 mg, 0.054 mmol) was added to a round bottom flask with a magnetic stir bar with NMO (15 mg, 0.128 mmol) which was then dissolved in a mixture of 9:1 acetone:water (1 mL, 0.9 M). The reaction mixture was allowed to stir at room temperature for two minutes before OsO_4 as a 4% solution in water (5.4 µL, 1 mol %) was added. The reaction mixture stirred for 18 hours before quenching with aqueous sodium sulfite, and the product was extracted with ethyl acetate three times. The combined organic layers were washed with brine, and then dried with Na_2SO_4 before concentrating down *in vacuo* to yield **S18** as a light grey/white solid (16.4 mg, 71%).

^{1}H NMR (400 MHz, $CDCl_3$) δ_H 7.17 (1H, d, J = 8.5 Hz), 6.81 (1H, d, J = 9.1 Hz), 4.25-4.17 (1H, m), 3.89 (1H, dd, J = 11.0, 3.8 Hz), 3.53 (1H, dd, J = 11.0, 6.7 Hz), 3.40 (1H, dd, J = 14.7, 2.9 Hz), 3.17 (1H, dd, J = 14.0, 7.4 Hz), 1.70 (3H, s), 1.68 (3H, s) ppm, all O-H peaks not observed.

^{13}C{^{1}H} NMR (101 MHz, Acetone-*d$_6$*) δ 161.4, 153.1, 151.2, 129.8, 125.2, 117.1, 114.6, 105.8, 74.5, 66.8, 31.4, 26.1, 25.0 ppm.

R_f = 0.42 (75% ethyl acetate in hexane, visualized by UV lamp).

HRMS = (ESI) *m/z*: $[M+H]^+$ calculated for $C_{13}H_{17}O_6$ 269.1019; Found 269.1018.

Synthesis of Botyroisocoumarin A (47) starting from diol S18

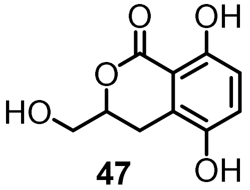

47

Diol **S18** (20 mg, 0.0746 mmol) was dissolved in a screw-cap vial charged with a magnetic stir bar in a 9:1 mixture of acetone and water (0.5 mL, 0.15 M). NaOH (3.9 mg, 0.0969 mmol) was added at once to the mixture, and the reaction stirred at room temperature for 15 hours, determined complete by TLC. The mixture was quenched with 4 drops of 1 M HCl to around pH 1 and stirred for five additional minutes. The acetone was removed under vacuum and the reaction mixture was extracted with ethyl acetate three times. The combined organic layers were washed with brine once, dried with Na_2SO_4, filtered, and concentrated down before purification through column chromatography with an eluent of 20% to 40% acetone in hexane to yield **45** (8.2 mg, 52%) as a white solid.

Synthesis of Botyroisocoumarin A (47) starting from lactone 1a

Lactone **1a** (20 mg, 0.0853 mmol) and NMO (29.9 mg, 0.256 mmol) were added to a vial that contained a 1:1 mixture acetone and DI water (1.7 mL, 0.05 M) and allowed to stir for 5 minutes. OsO_4 (10.3 µL, 1.9 mol%) as a 4% solution in water was added to the reaction mixture and allowed to continue to stir for 24 hours to consume the alkene completely. Diol **S18** was confirmed via TLC, and then 2M NaOH (55.3 µL, 0.111 mmol) was added and stirred for 30 minutes, confirming consumption of the diol (**S18**) intermediate. Once the reaction was completed, 1M HCl was used to quench the reaction to around pH 1 and then the acetone was concentrated under vacuum. The residue was then extracted with ethyl acetate three times, and the combined organic fractions were washed with brine, then dried with Na_2SO_4, filtered, and concentrated down before purification through column chromatography with an eluent of 5% to 75% acetone in hexane to yield **45** (9 mg, 57%) as a white solid.

^{1}H NMR (400 MHz, MeOD) δ_H 7.04 (1H, d, J = 9.1 Hz), 6.71 (1H, d, J = 9.0 Hz), 4.59 (1H, m), 3.86 (1H, dd, J = 12.2, 4.0 Hz), 3.80 (1H, dd, J = 12.1, 5.0 Hz), 3.13 (1H, dd, J = 16.6, 3.3 Hz), 2.79 (1H, dd, J = 16.9, 12.0 Hz) ppm, confirmed with the literature source *J. Antibiot.*, **2015**, *68* 653-656.

Synthesis of 5-Hydroxymellein (46)

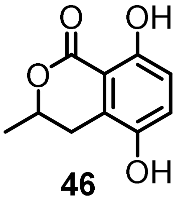

46

Lactone **1a** (20 mg, 0.0853 mmol) was dissolved in THF (1 mL, 0.08 M) in a round bottom flask equipped with a magnetic stir bar. Sulfuric acid (455 µL, 8.5 mmol) was added slowly over five minutes, then DI water (2.3 µL, 0.0939 mmol) was added at room temperature and stirred for 5 additional minutes at 60 °C in an oil bath. The reaction mixture was concentrated under reduced pressure to remove the solvent and extracted with ethyl acetate three times. The combined organic fractions were washed with brine, dried with Na_2SO_4 and concentrated in vacuo. **44** was purified through column chromatography with an eluent of 10% to 35% ethyl acetate in hexane to yield **44** as a yellow solid (11.7 mg, 68%).

^{1}H NMR (400 MHz, DMSO-d_6) δ_H 10.39 (1H, s), 9.35 (1H, s), 7.07 (1H, d, J = 8.3 Hz), 6.72 (1H, d, J = 9.1 Hz), 4.73 (1H, dqd, J_d = 3.3, 12.4 Hz, J_q = 7.8 Hz), 3.08 (1H, dd, J = 17.0, 3.4 Hz), 2.59 (1H, dd, J = 16.8, 11.5 Hz), 1.43 (3H, d, J = 6.3 Hz) ppm, confirmed with the literature source *Phytochem. Lett.*, **1994**, *35*, 825-826.

Synthesis of 5-Hydroxy-2-methoxybenzonitrile (S19)

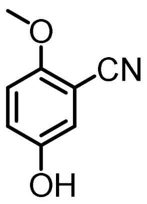

S19

To an oven dried round bottom flask equipped with a stir bar, 2,5-dimethoxybenzonitrile (2 g, 12.3 mmol) was dissolved in dry and degassed toluene (61 mL, 0.1 M). $AlCl_3$ (2.9 g, 22.1 mmol) and NaI (3.7 g, 24.5 mmol) were slowly and carefully added to the mixture at 0 °C. The reaction mixture was then heated to 100 °C with a heating mantle and allowed to stir for 22 hours. The reaction mixture was quenched with ice cold water at 0 °C, slowly. The mixture was then diluted with ethyl acetate and washed four times with aqueous 2M NaOH. The combined aqueous extracts were then acidified to pH 1 and washed four times with ethyl acetate. The combined organic fractions were dried with Na_2SO_4, filtered, and concentrated down. **S19** was purified through column chromatography with an eluent of 10% to 50% ethyl acetate in hexane to yield **S19** as a white solid (595 mg, 33%).

^{1}H NMR (400 MHz, $CDCl_3$) δ_H 7.06-7.03 (2H, m), 6.86 (1H, d, J = 10.0 Hz), 4.69 (1H, br s), 3.88 (3H, s) ppm, confirmed with the literature source *J. Org. Chem.* **1999**, *64*, 9719-9721.

^{1}H and ^{13}C NMR Spectra of Compounds

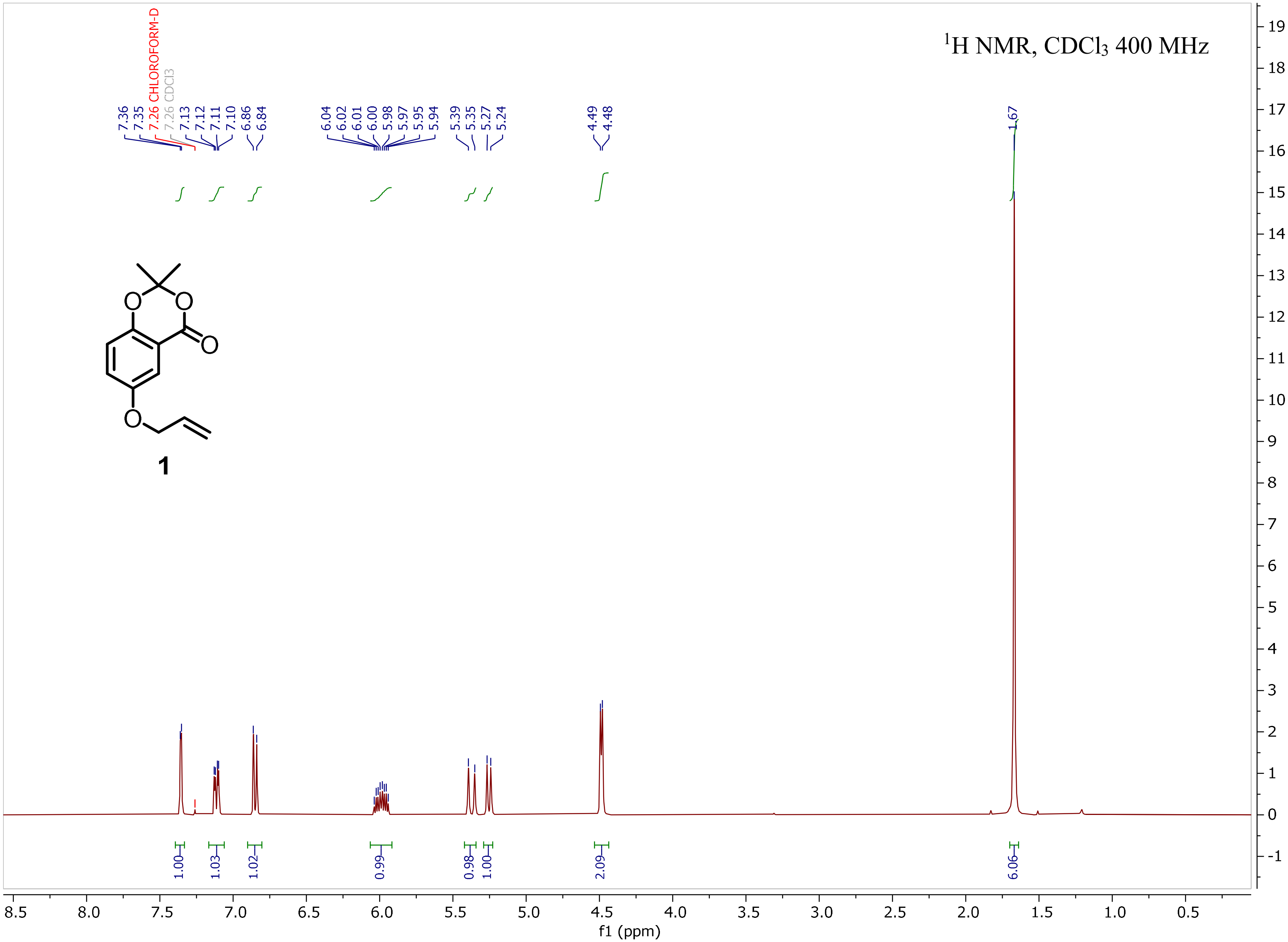

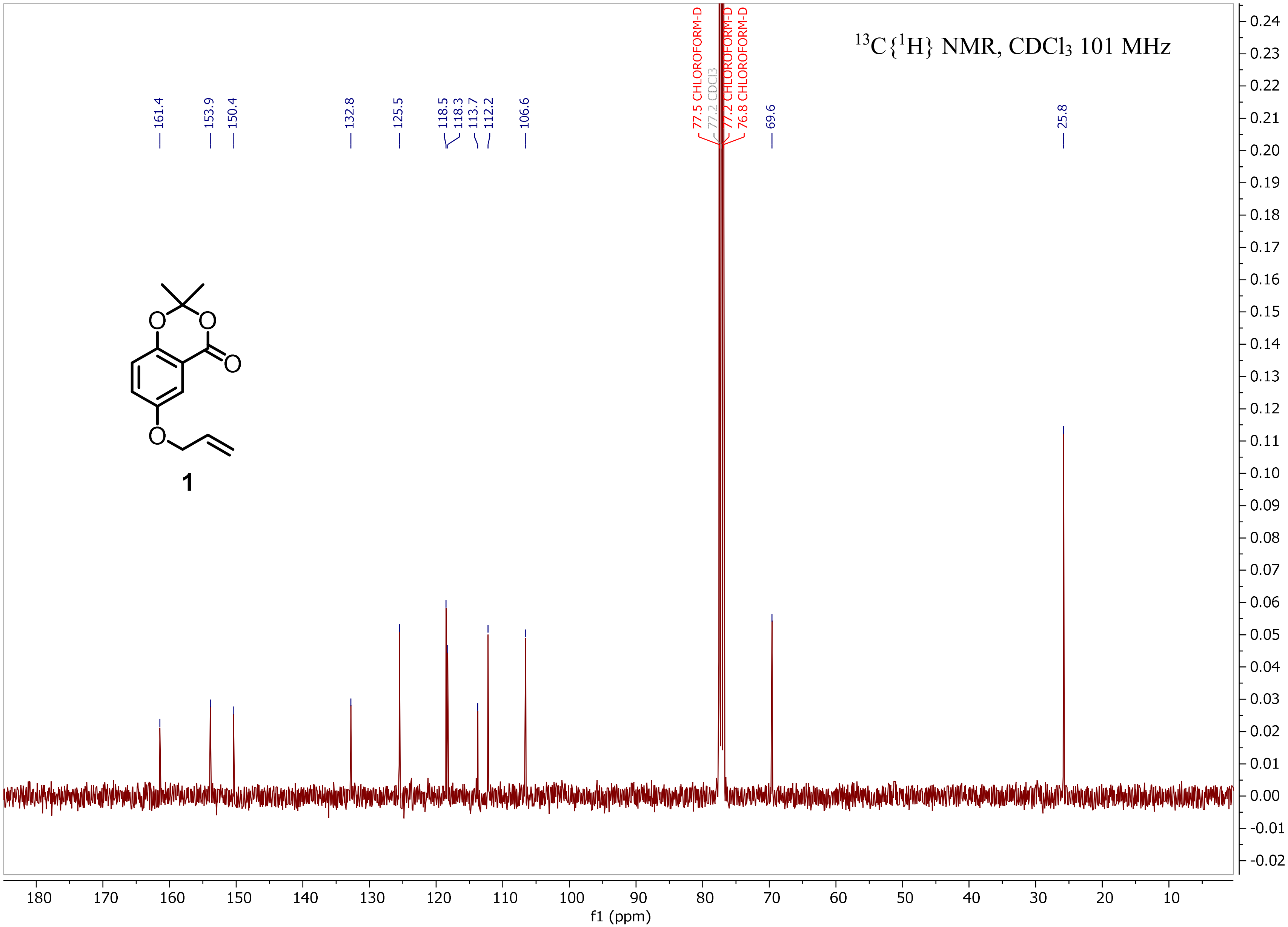
13C{1H} NMR, CDCl3 101 MHz
161.4
153.9
150.4
132.8
125.5
118.5
118.3
113.7
112.2
106.6
77.5 CHLOROFORM-D
77.2 CDCl3
77.2 CHLOROFORM-D
76.8 CHLOROFORM-D
69.6
25.8
1
f1 (ppm)

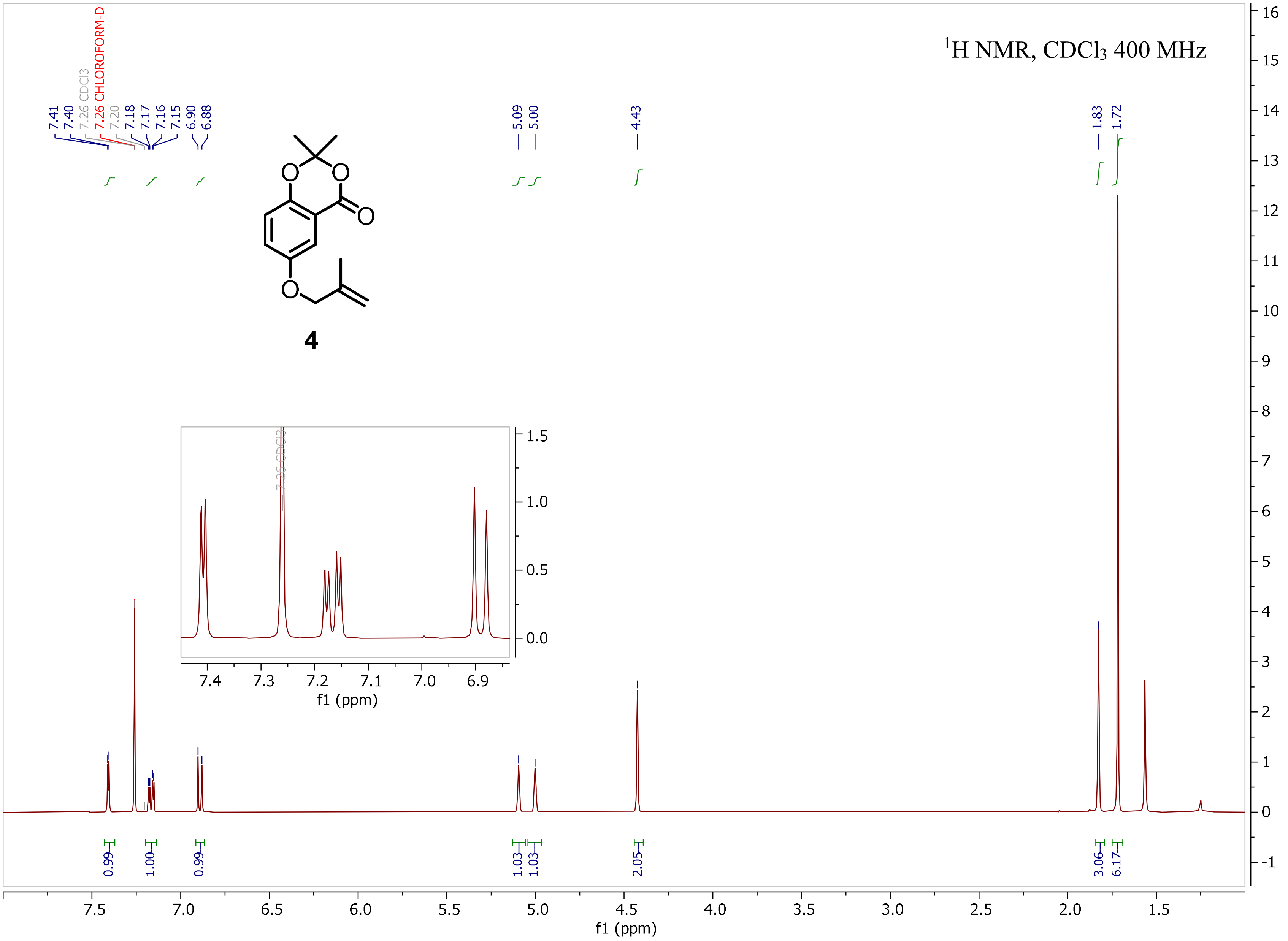

^{1}H NMR, CDCl$_3$ 400 MHz
4
f1 (ppm)

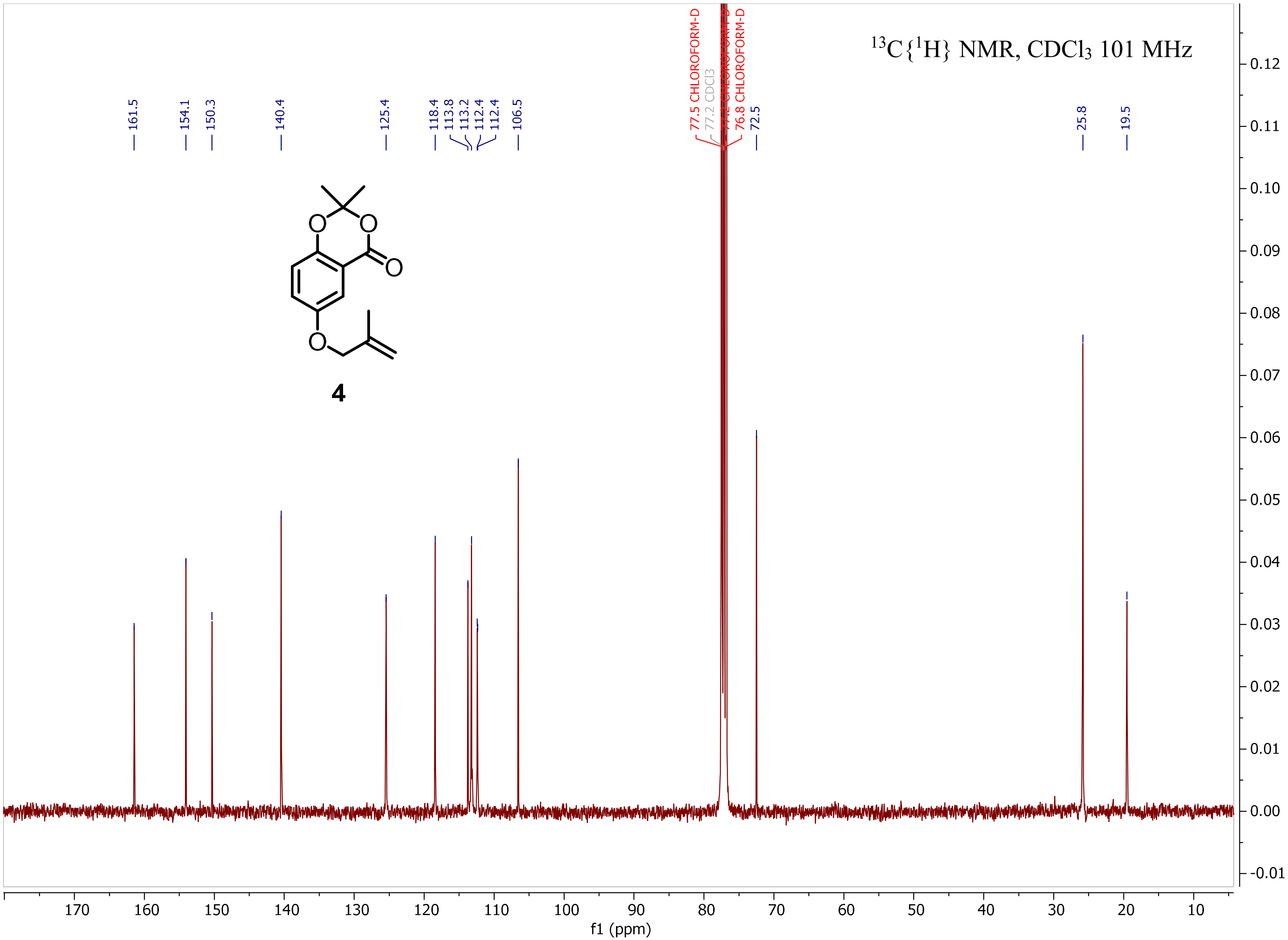
$^{13}C\{^1H\}$ NMR, $CDCl_3$ 101 MHz
161.5
154.1
150.3
140.4
125.4
118.4
113.8
113.2
112.4
112.4
106.5
77.5 CHLOROFORM-D
77.2 CDCl3
77.2 CHLOROFORM-D
76.8 CHLOROFORM-D
72.5
25.8
19.5
4
f1 (ppm)

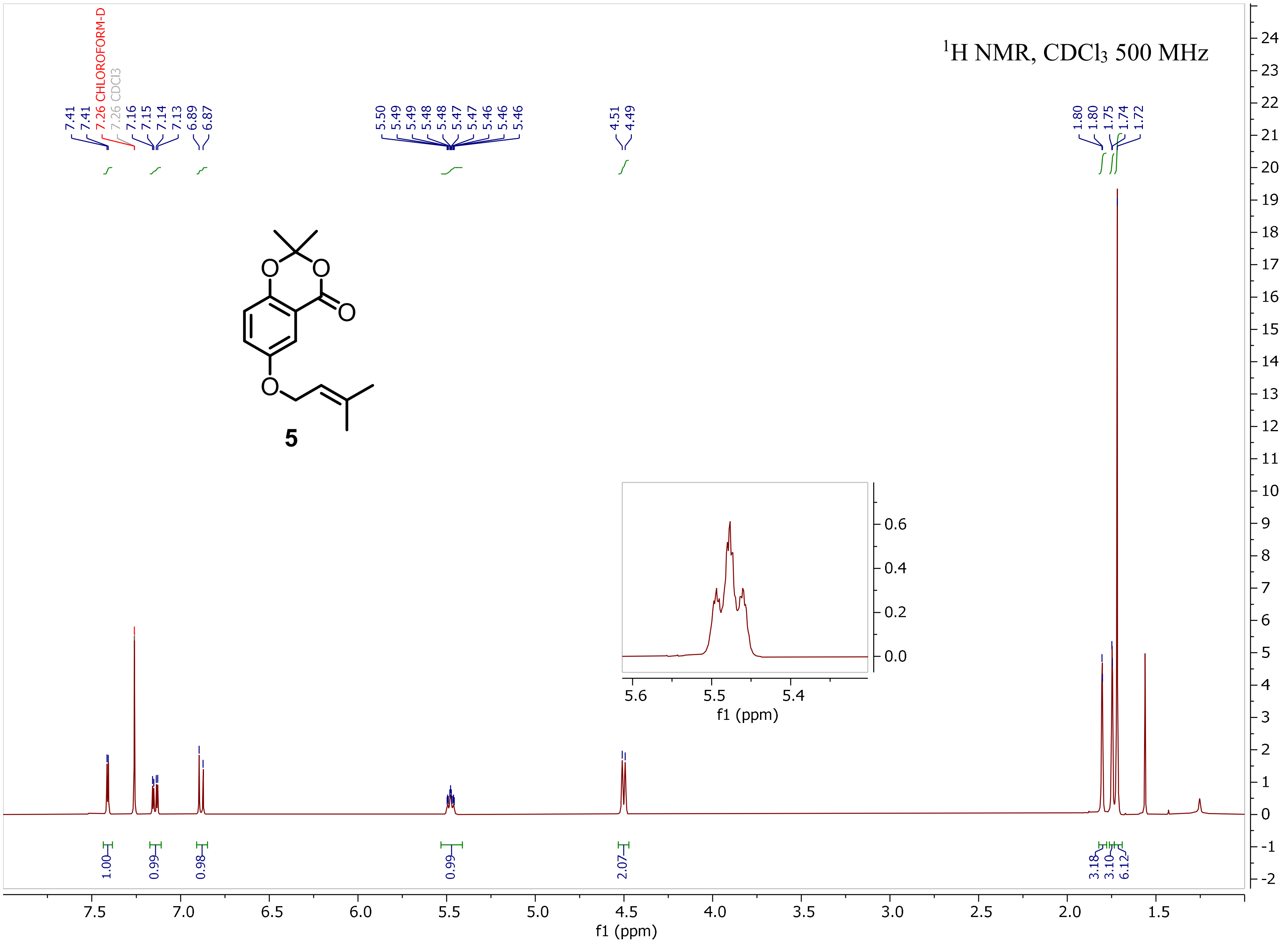

¹H NMR, CDCl3 500 MHz
5
7.41
7.41
7.26 CHLOROFORM-D
7.26 CDCl3
7.16
7.15
7.14
7.13
6.89
6.87
5.50
5.49
5.49
5.48
5.48
5.47
5.47
5.46
5.46
5.46
4.51
4.49
1.80
1.80
1.75
1.74
1.72
1.00
0.99
0.98
0.99
2.07
3.18
3.10
6.12
f1 (ppm)

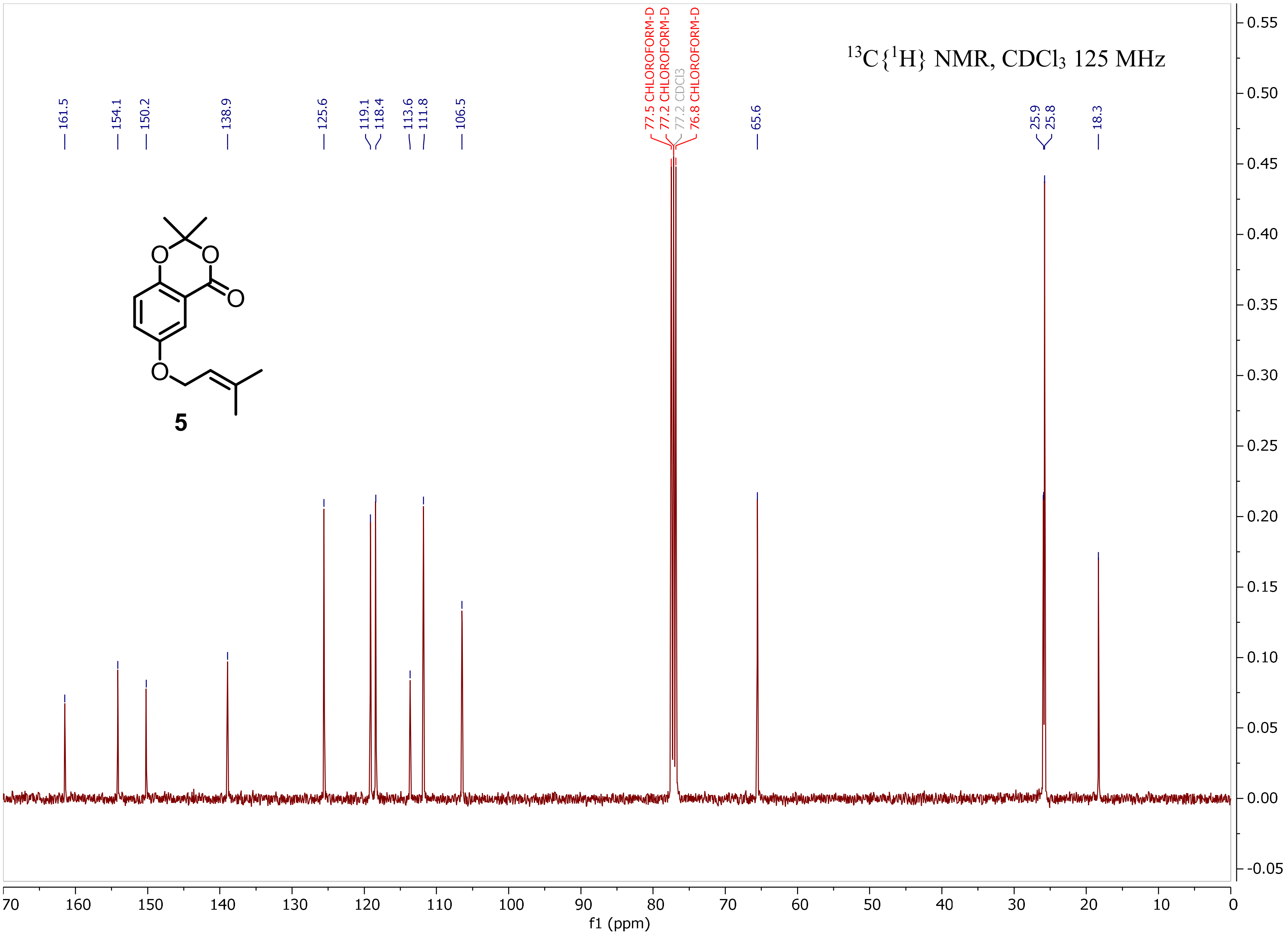

13C{1H} NMR, CDCl3 125 MHz
161.5
154.1
150.2
138.9
125.6
119.1
118.4
113.6
111.8
106.5
77.5 CHLOROFORM-D
77.2 CHLOROFORM-D
77.2 CDCl3
76.8 CHLOROFORM-D
65.6
25.9
25.8
18.3
5
f1 (ppm)

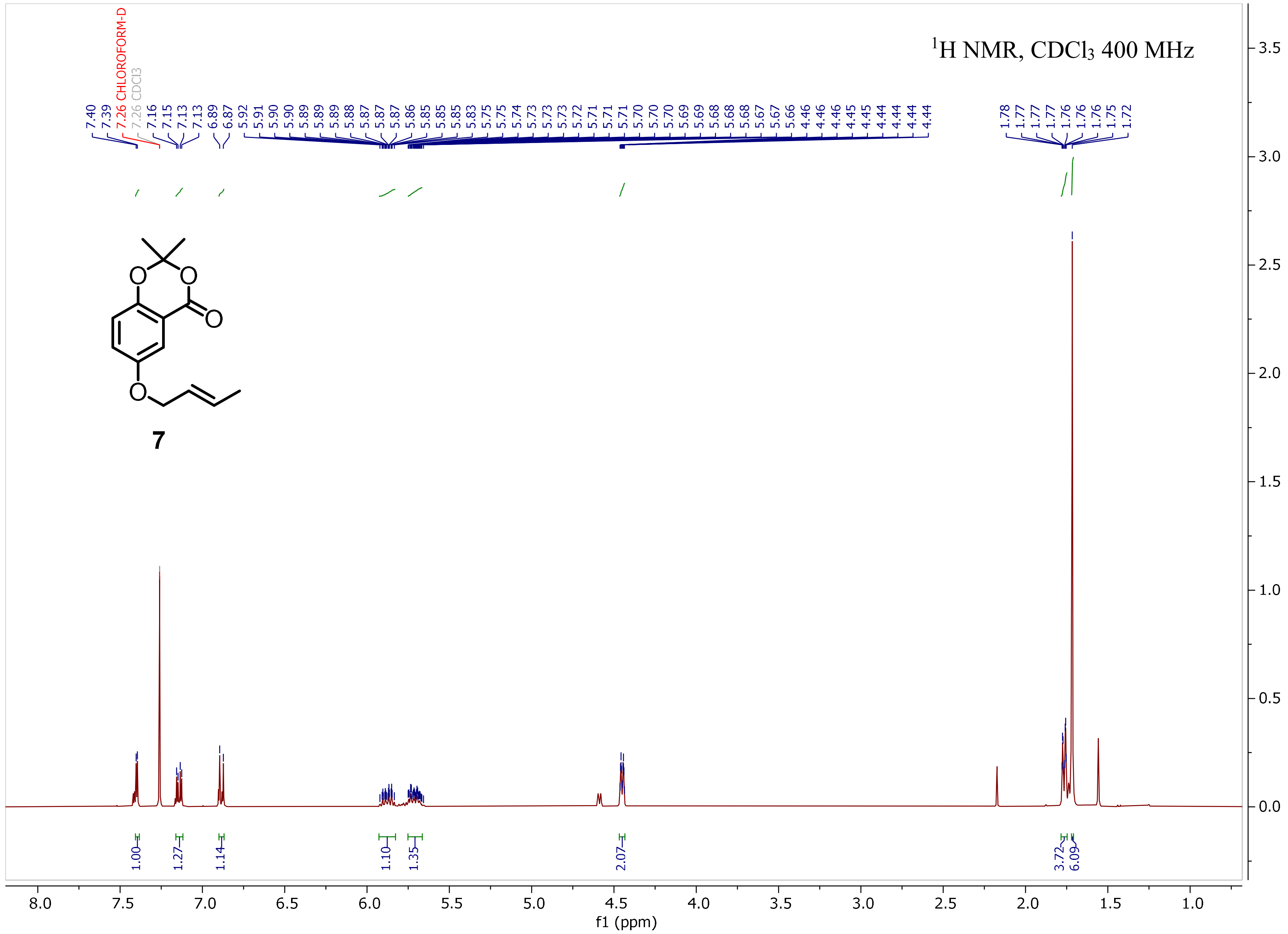

^{1}H NMR, CDCl3 400 MHz
7
f1 (ppm)

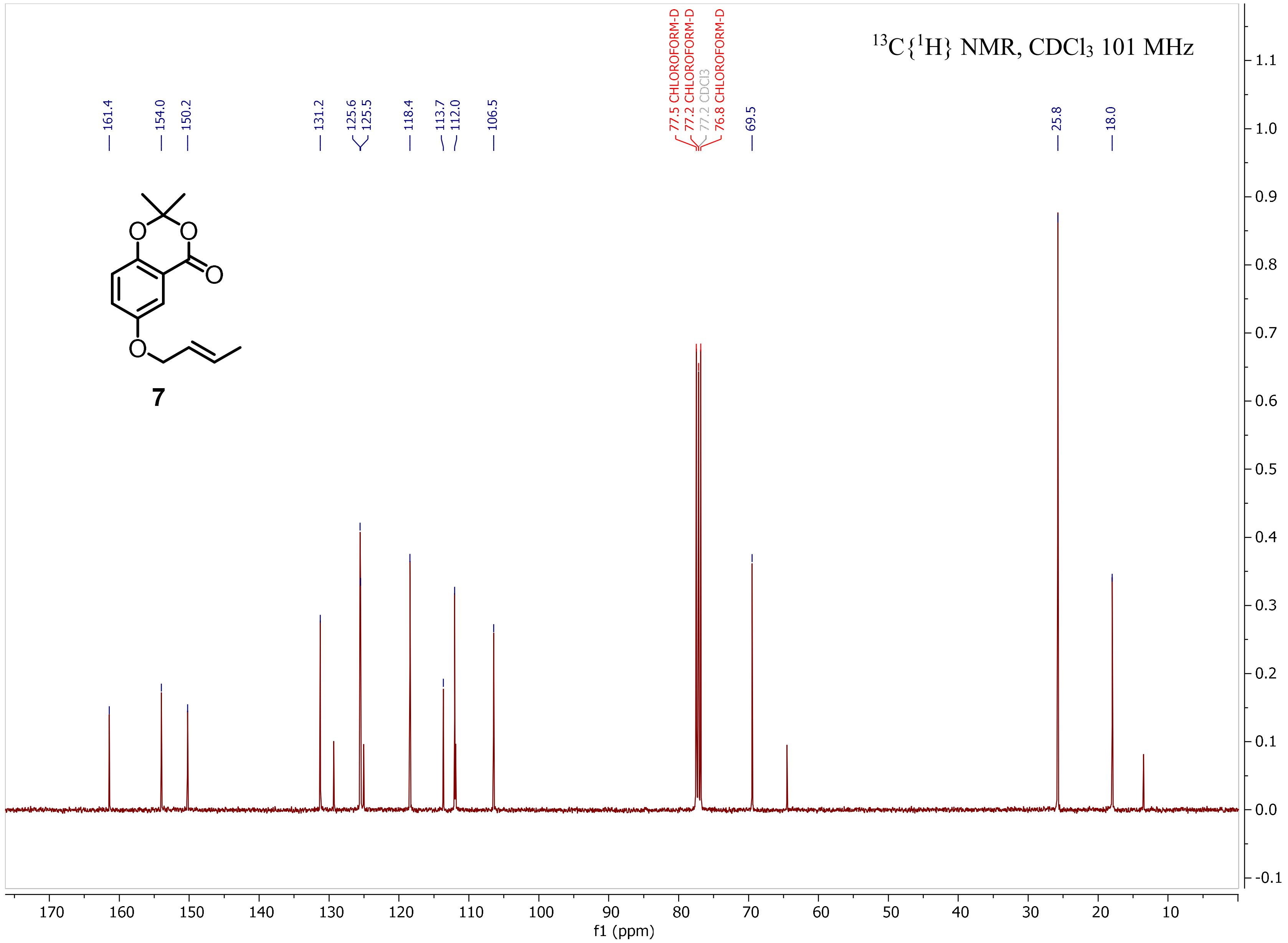

$^{13}C\{^1H\}$ NMR, $CDCl_3$ 101 MHz
161.4
154.0
150.2
131.2
125.6
125.5
118.4
113.7
112.0
106.5
77.5 CHLOROFORM-D
77.2 CHLOROFORM-D
77.2 CDCl3
76.8 CHLOROFORM-D
69.5
25.8
18.0
7
f1 (ppm)

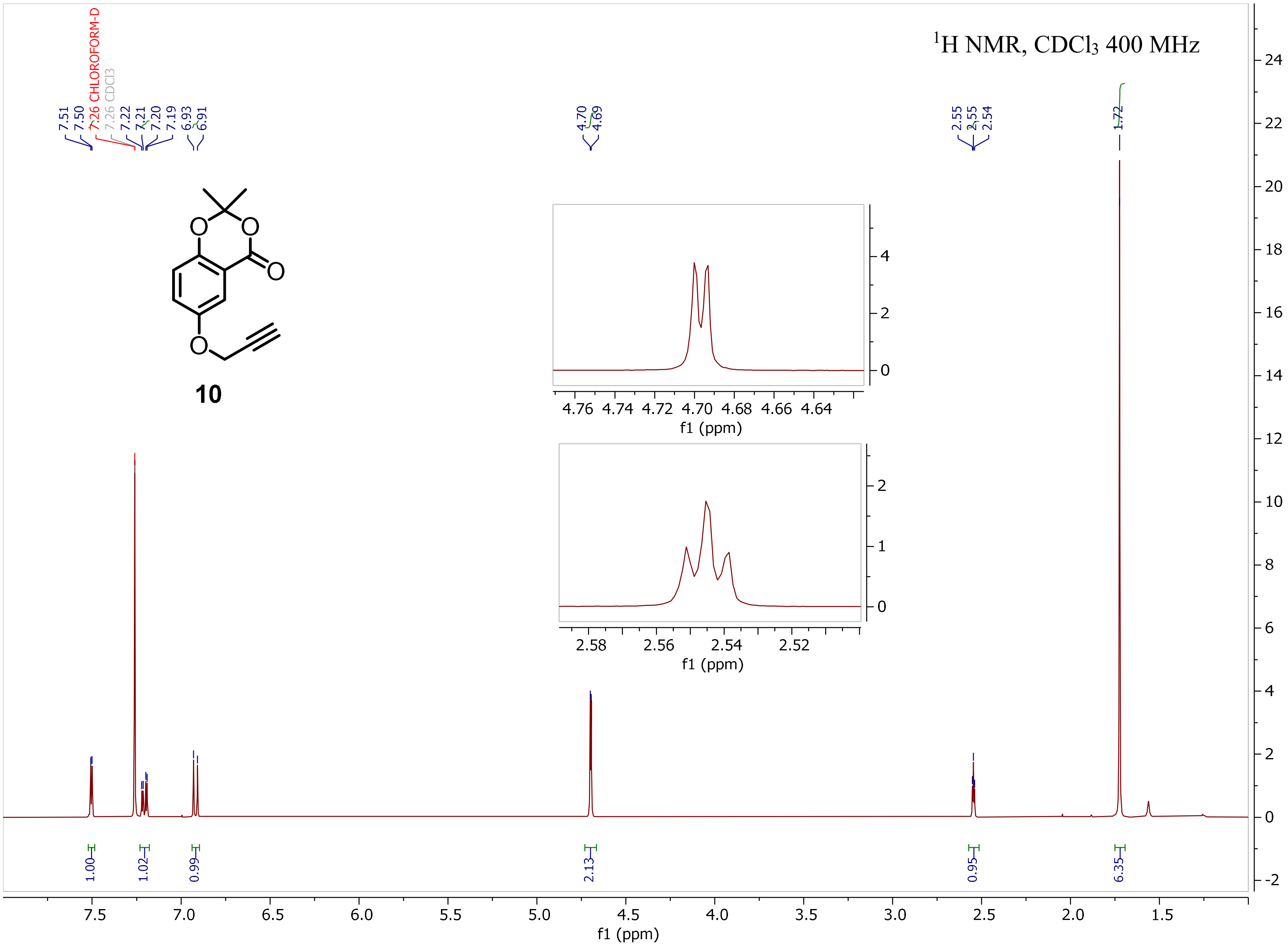

1H NMR, CDCl3 400 MHz
10
7.51
7.50
7.26 CHLOROFORM-D
7.26 CDCl3
7.22
7.21
7.20
7.19
6.93
6.91
4.70
4.69
2.55
2.55
2.54
1.72
1.00
1.02
0.99
2.13
0.95
6.35
f1 (ppm)

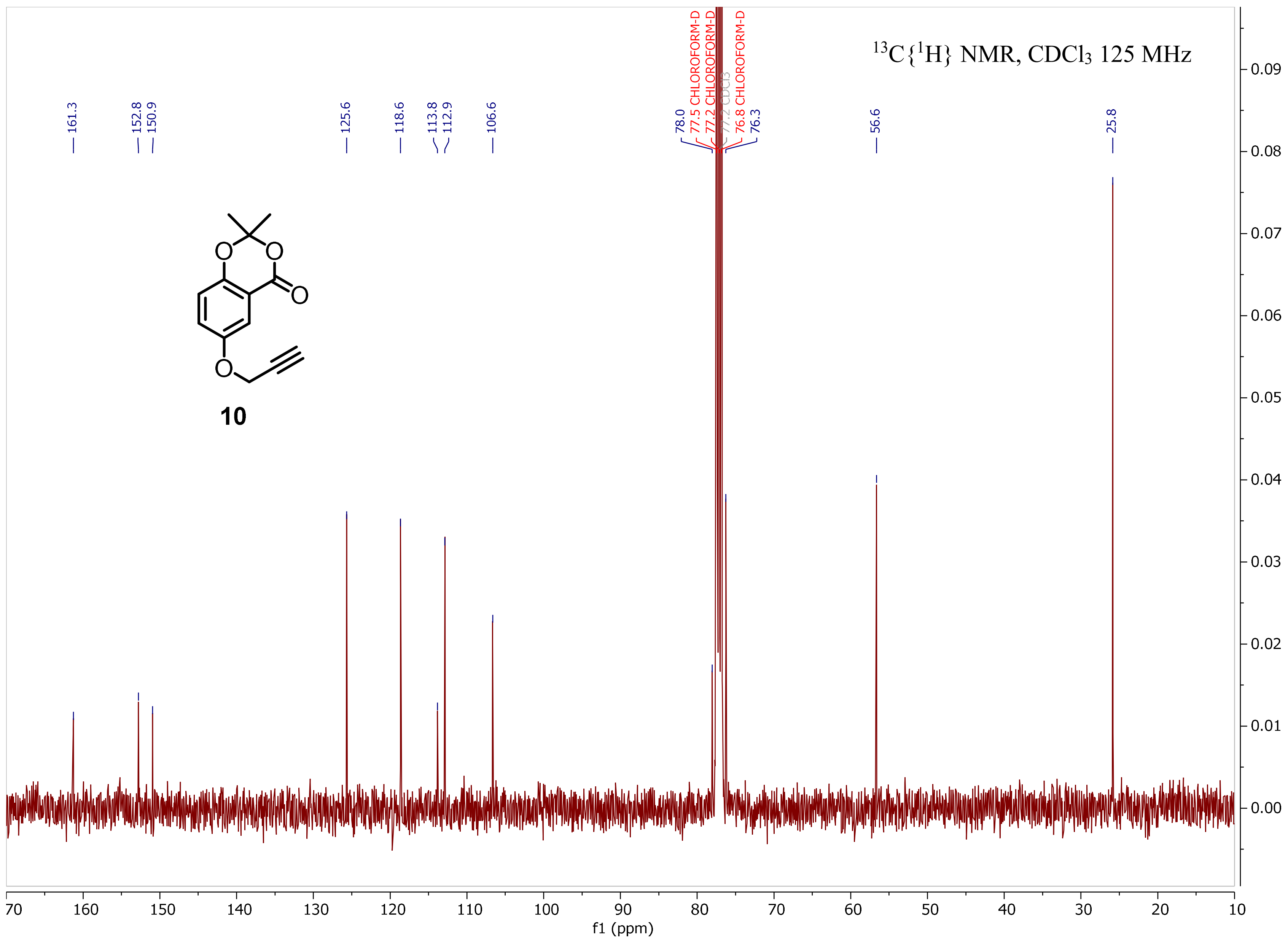

$^{13}C\{^1H\}$ NMR, $CDCl_3$ 125 MHz
161.3
152.8
150.9
125.6
118.6
113.8
112.9
106.6
78.0
77.5 CHLOROFORM-D
77.2 CHLOROFORM-D
77.2 CDCl3
76.8 CHLOROFORM-D
76.3
56.6
25.8
10
f1 (ppm)

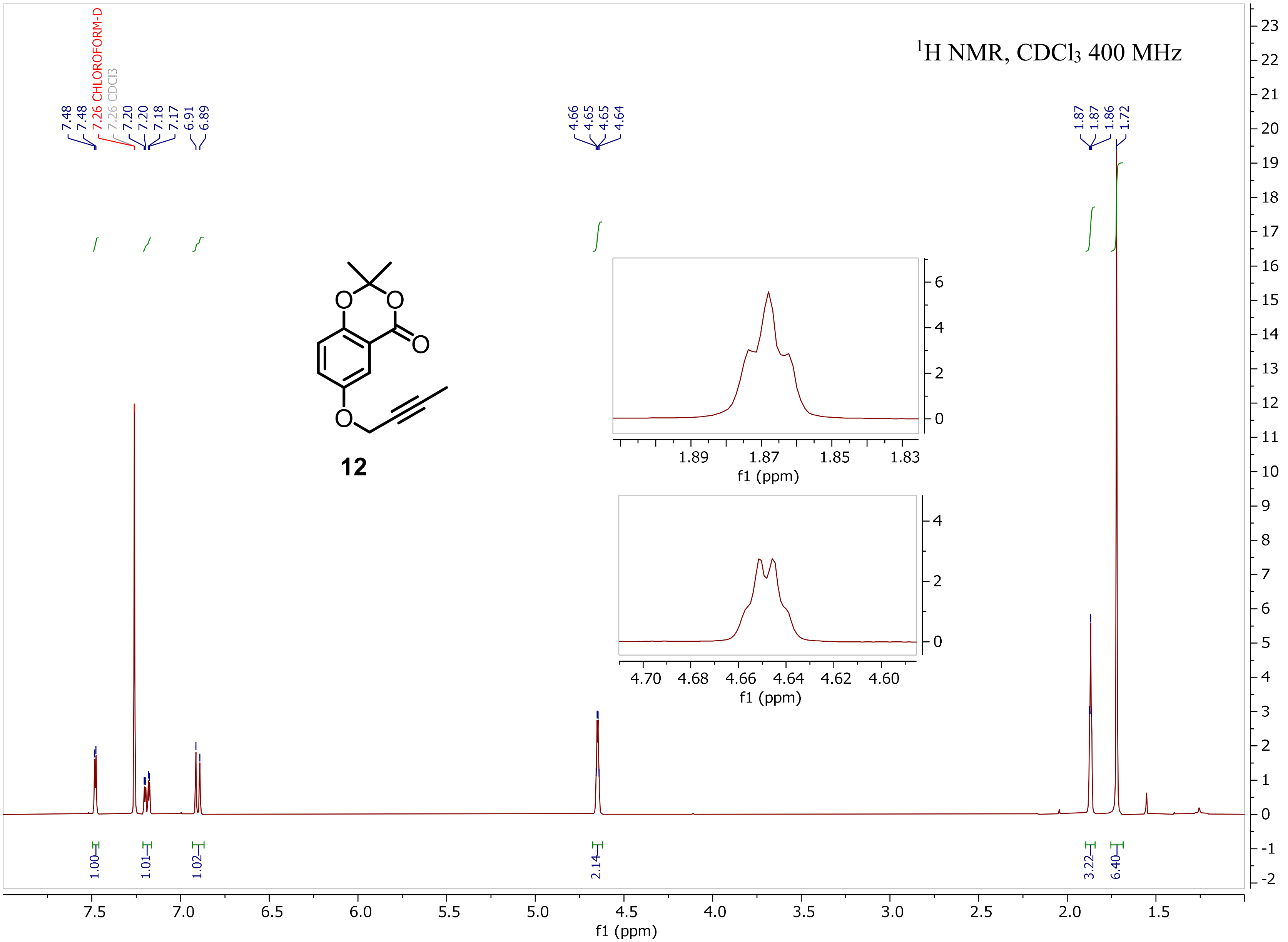

^{1}H NMR, CDCl$_3$ 400 MHz
7.48
7.48
7.26 CHLOROFORM-D
7.26 CDCl3
7.20
7.20
7.18
7.17
6.91
6.89
4.66
4.65
4.65
4.64
1.87
1.87
1.86
1.72
O
O
O
O
12
1.00
1.01
1.02
2.14
3.22
6.40
f1 (ppm)

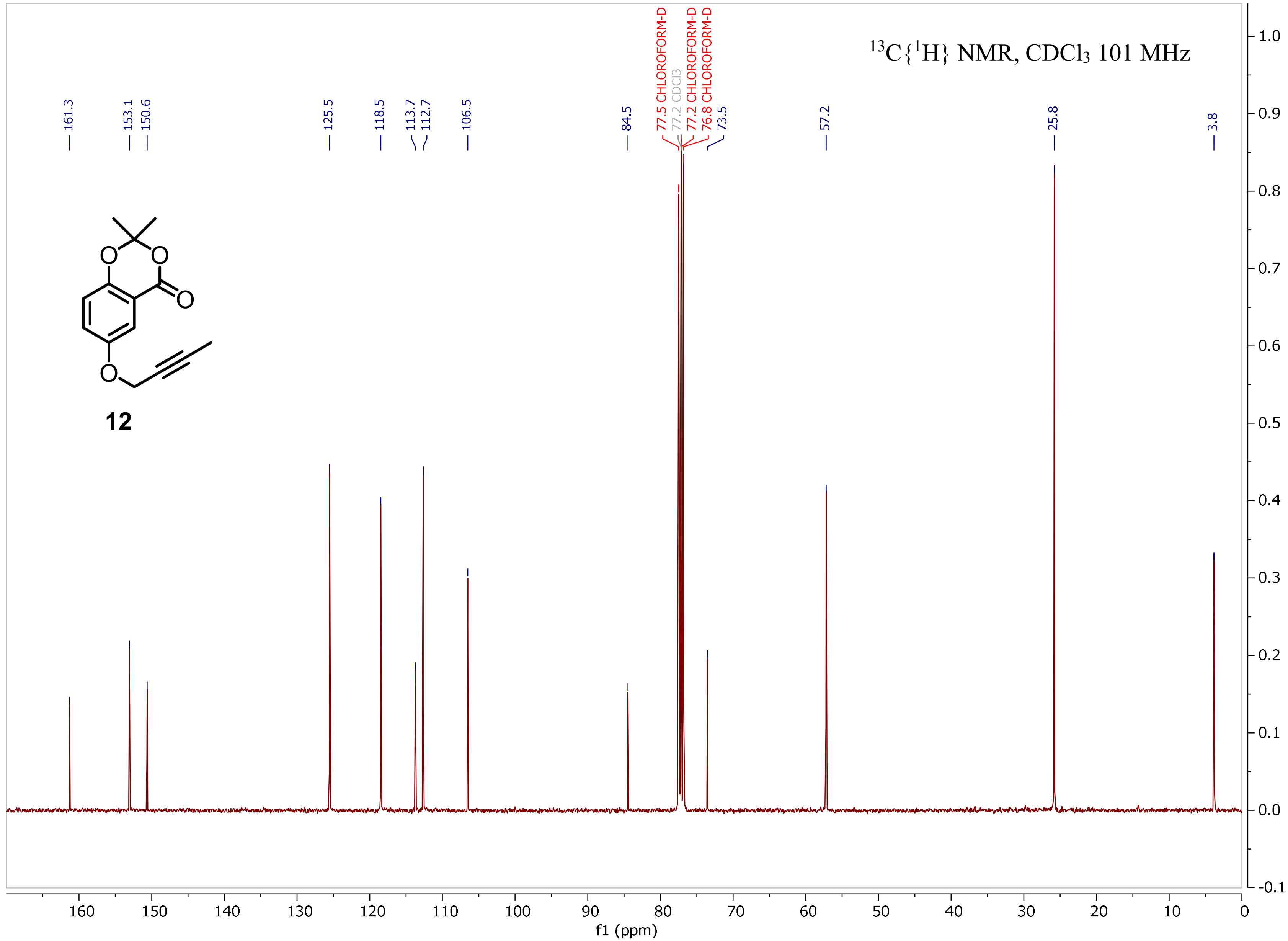
$^{13}C\{^1H\}$ NMR, $CDCl_3$ 101 MHz
161.3
153.1
150.6
125.5
118.5
113.7
112.7
106.5
84.5
77.5 CHLOROFORM-D
77.2 CDCl3
77.2 CHLOROFORM-D
76.8 CHLOROFORM-D
73.5
57.2
25.8
3.8
12
f1 (ppm)

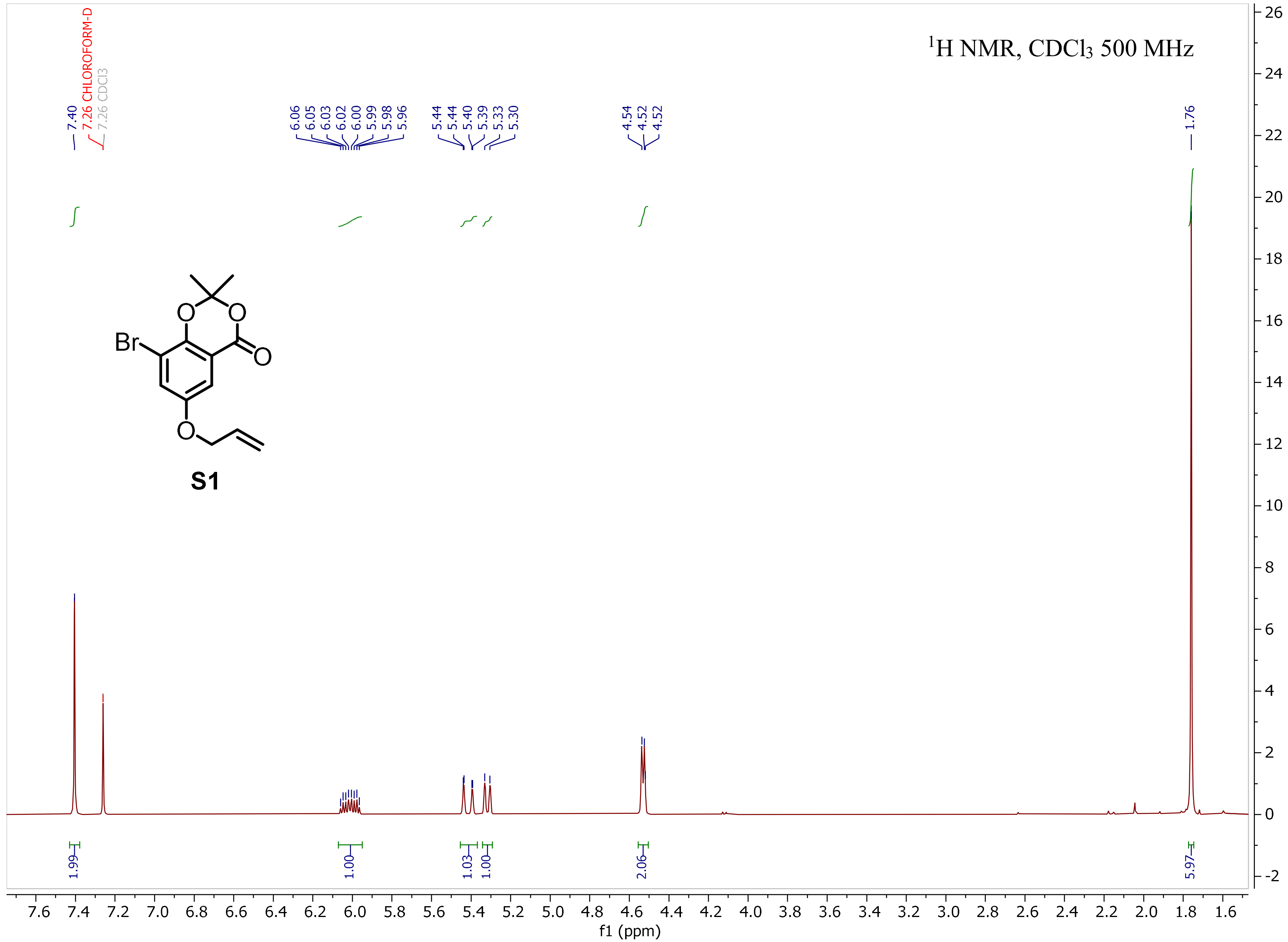

1H NMR, CDCl3 500 MHz
7.40
7.26 CHLOROFORM-D
7.26 CDCl3
6.06
6.05
6.03
6.02
6.00
5.99
5.98
5.96
5.44
5.44
5.40
5.39
5.33
5.30
4.54
4.52
4.52
1.76
Br
O
O
O
O
S1
1.99
1.00
1.03
1.00
2.06
5.97
f1 (ppm)

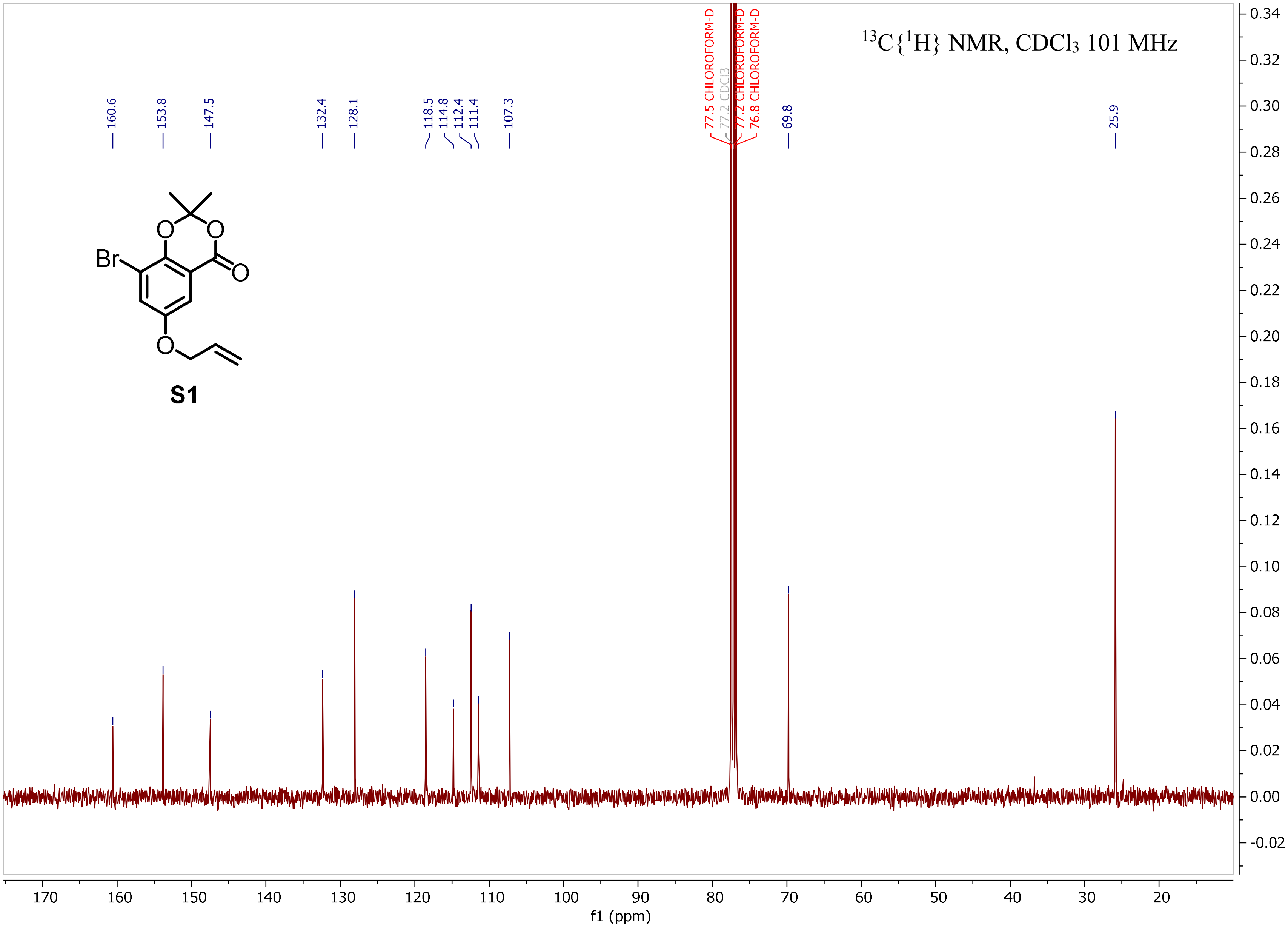

$^{13}C\{^1H\}$ NMR, CDCl3 101 MHz
160.6
153.8
147.5
132.4
128.1
118.5
114.8
112.4
111.4
107.3
77.5 CHLOROFORM-D
77.2 CDCl3
77.2 CHLOROFORM-D
76.8 CHLOROFORM-D
69.8
25.9
Br
O
S1
f1 (ppm)

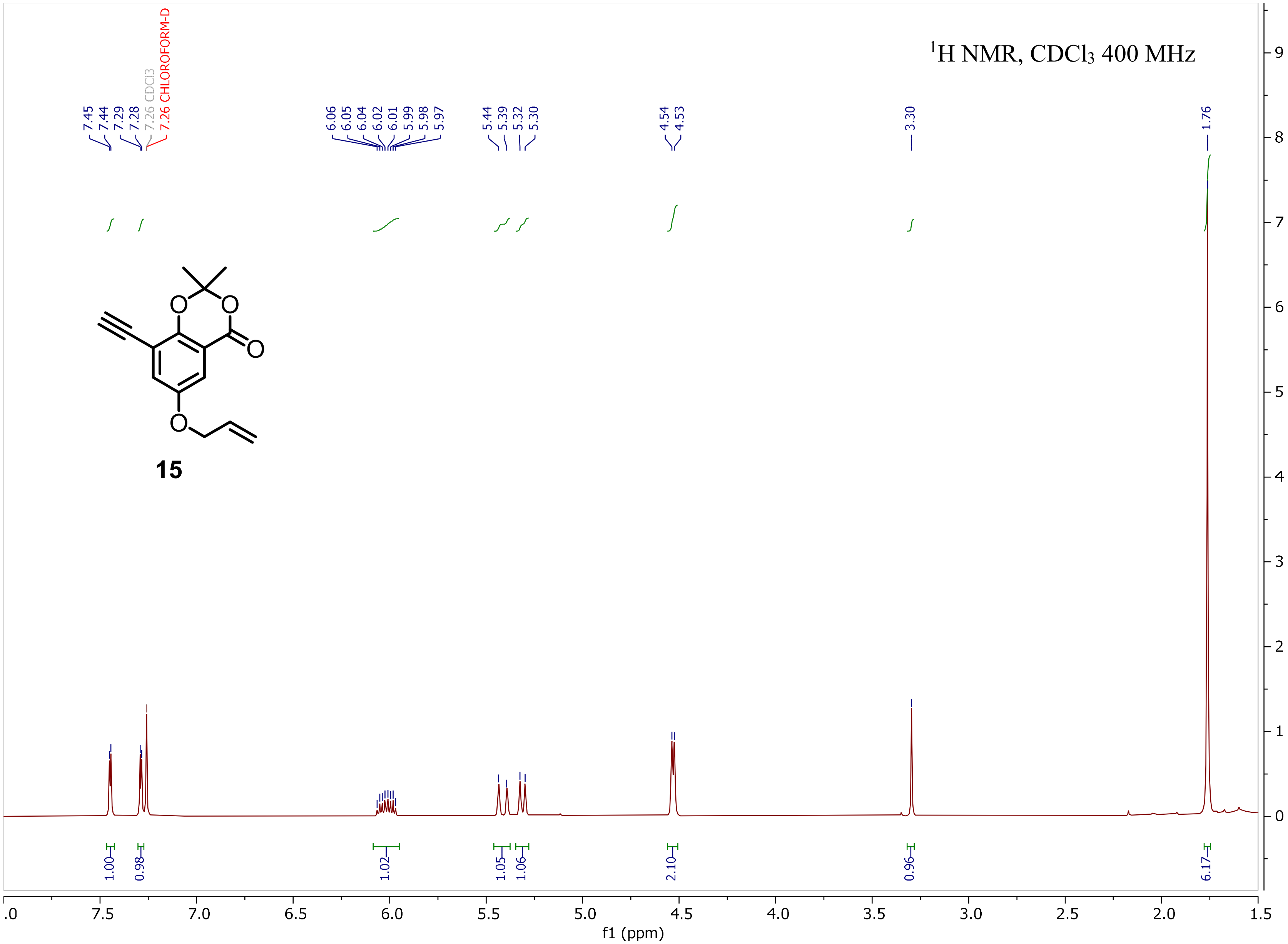

1H NMR, CDCl3 400 MHz
7.45
7.44
7.29
7.28
7.26 CDCl3
7.26 CHLOROFORM-D
6.06
6.05
6.04
6.02
6.01
5.99
5.98
5.97
5.44
5.39
5.32
5.30
4.54
4.53
3.30
1.76
1.00
0.98
1.02
1.05
1.06
2.10
0.96
6.17
f1 (ppm)

15

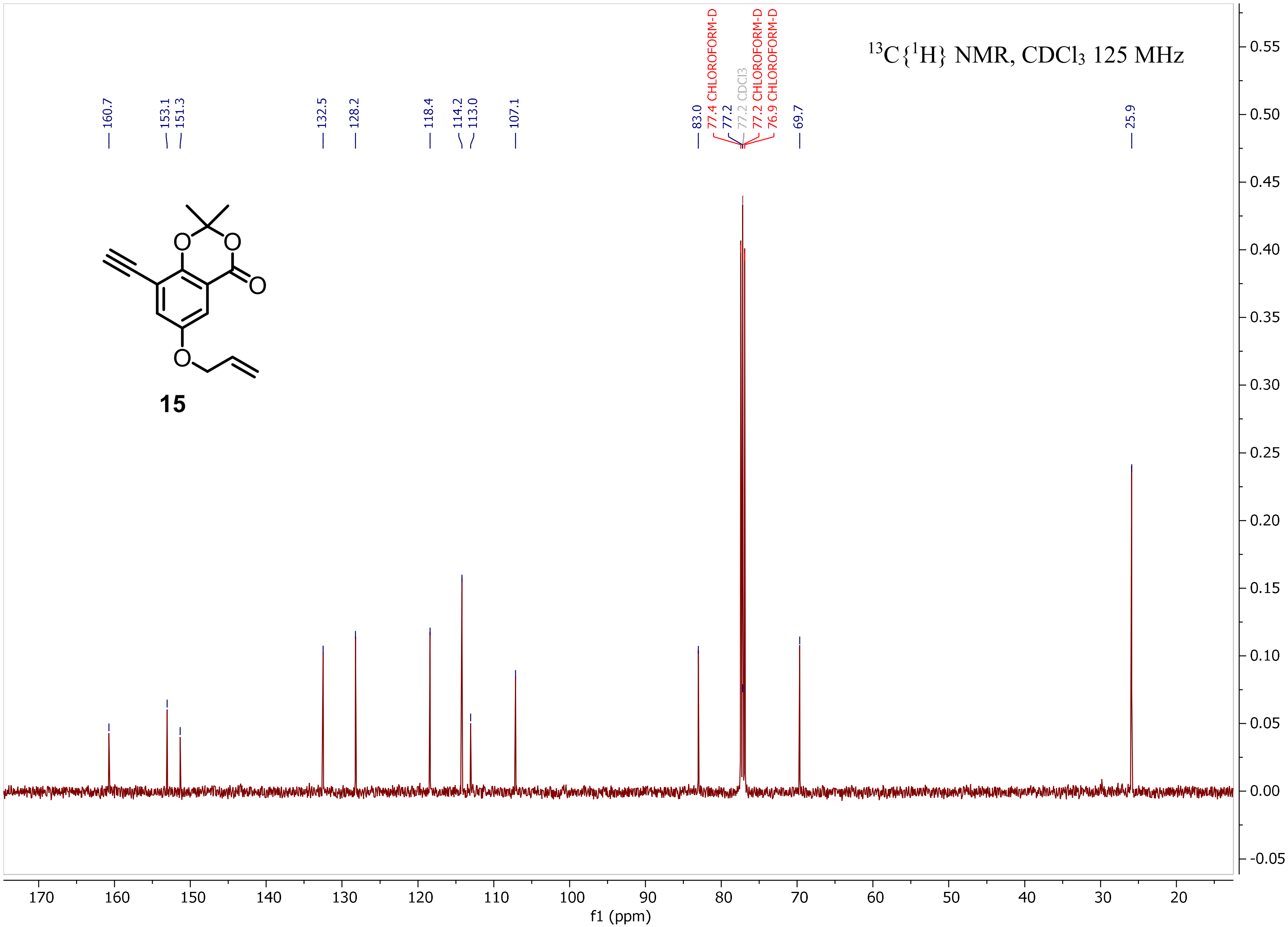
$^{13}C\{^1H\}$ NMR, $CDCl_3$ 125 MHz
160.7
153.1
151.3
132.5
128.2
118.4
114.2
113.0
107.1
83.0
77.4 CHLOROFORM-D
77.2
77.2 CDCl3
77.2 CHLOROFORM-D
76.9 CHLOROFORM-D
69.7
25.9
15
f1 (ppm)

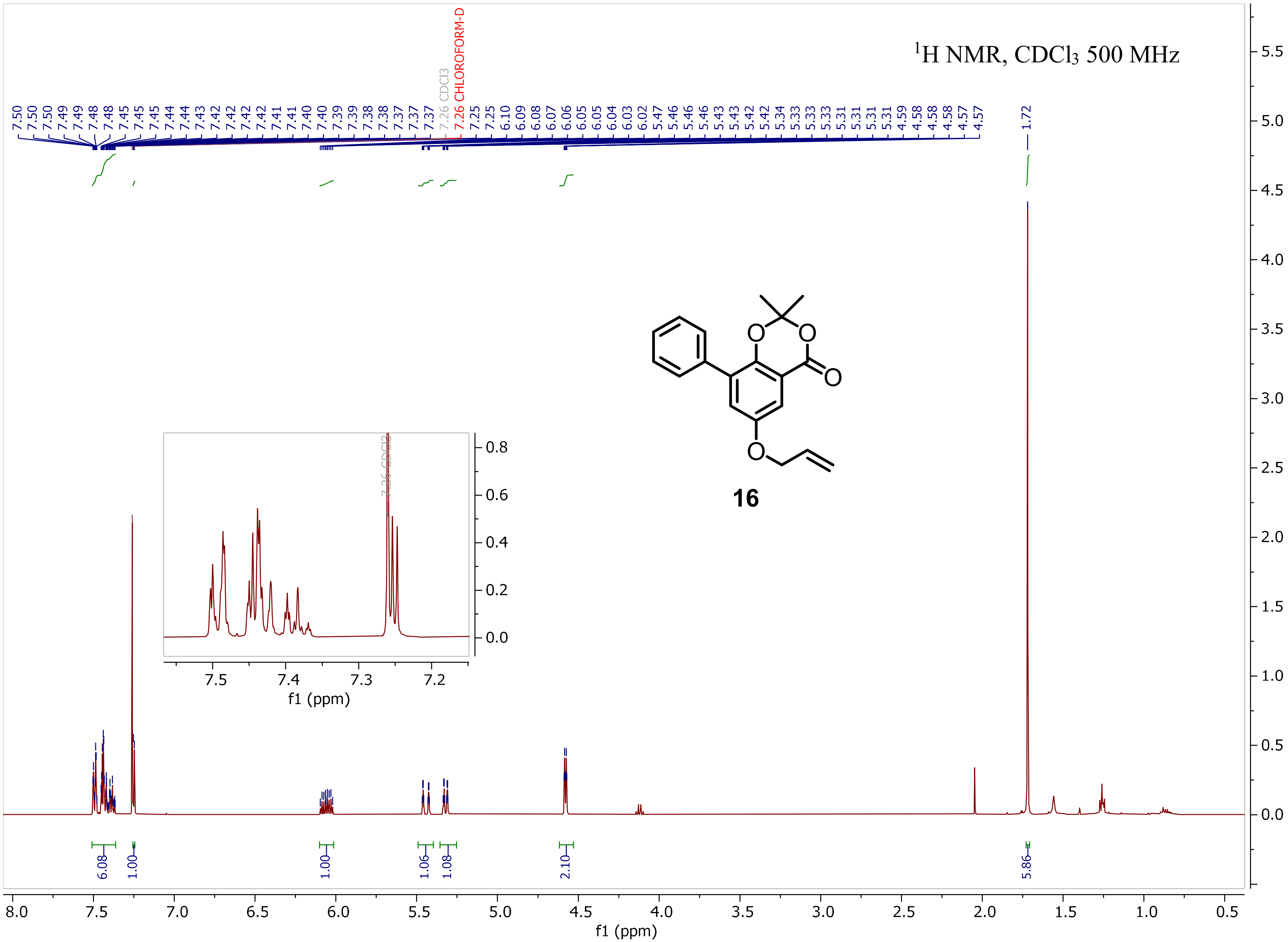

1H NMR, CDCl3 500 MHz
7.50 7.50 7.50 7.49 7.49 7.48 7.48 7.45 7.45 7.44 7.44 7.43 7.42 7.42 7.42 7.42 7.42 7.41 7.41 7.40 7.40 7.39 7.39 7.38 7.38 7.37 7.37 7.26 CDCl3 7.26 CHLOROFORM-D 7.25 7.25 6.10 6.09 6.08 6.07 6.06 6.05 6.05 6.04 6.03 6.02 5.47 5.46 5.46 5.46 5.43 5.43 5.42 5.42 5.34 5.33 5.33 5.33 5.31 5.31 5.31 5.31 4.59 4.58 4.58 4.58 4.57 4.57
1.72
16
6.08
1.00
1.00
1.06
1.08
2.10
5.86
f1 (ppm)

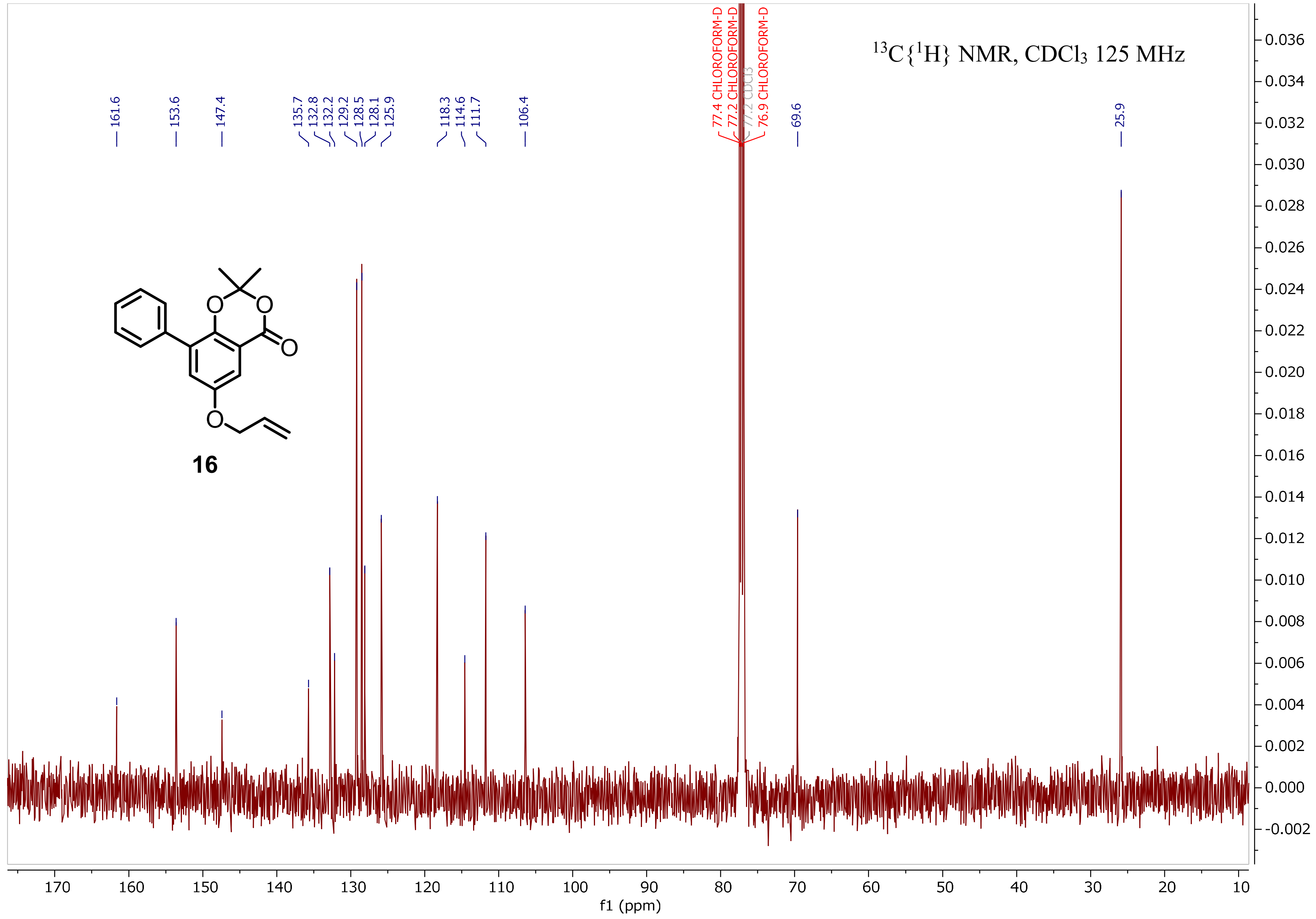

$^{13}C\{^{1}H\}$ NMR, $CDCl_3$ 125 MHz
161.6
153.6
147.4
135.7
132.8
132.2
129.2
128.5
128.1
125.9
118.3
114.6
111.7
106.4
77.4 CHLOROFORM-D
77.2 CHLOROFORM-D
77.2 CDCl3
76.9 CHLOROFORM-D
69.6
25.9
16
f1 (ppm)

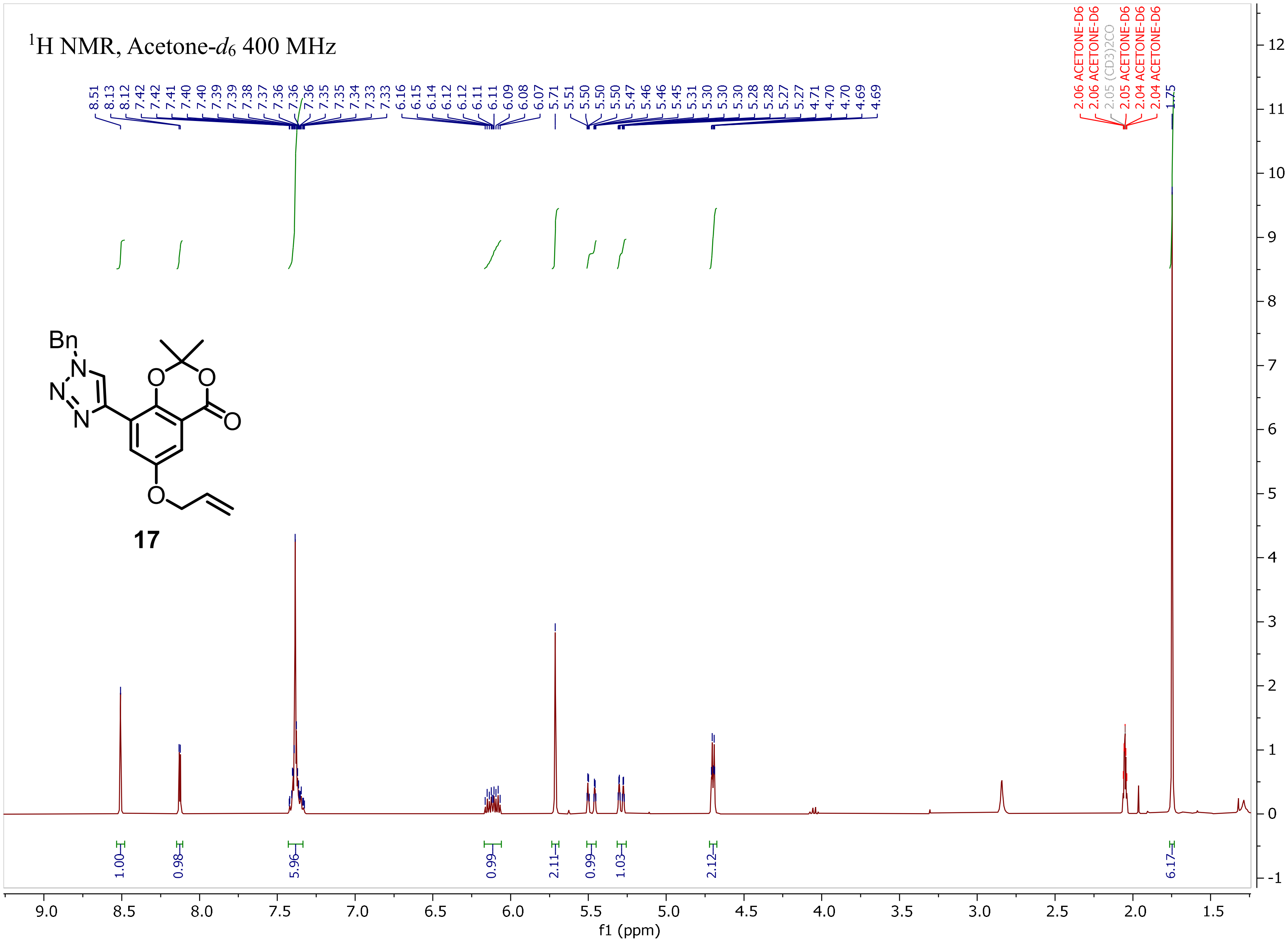

^{1}H NMR, Acetone-d_6 400 MHz
8.51
8.13
8.12
7.42
7.42
7.41
7.40
7.40
7.39
7.39
7.38
7.37
7.36
7.36
7.36
7.35
7.35
7.34
7.33
7.33
6.16
6.15
6.14
6.12
6.12
6.11
6.11
6.09
6.08
6.07
5.71
5.51
5.50
5.50
5.50
5.47
5.46
5.46
5.45
5.31
5.30
5.30
5.30
5.28
5.27
5.27
4.71
4.70
4.70
4.69
4.69
2.06 ACETONE-D6
2.06 ACETONE-D6
2.05 (CD3)2CO
2.05 ACETONE-D6
2.04 ACETONE-D6
2.04 ACETONE-D6
1.75
Bn
N
N
N
O
O
O
O
17
1.00
0.98
5.96
0.99
2.11
0.99
1.03
2.12
6.17
f1 (ppm)

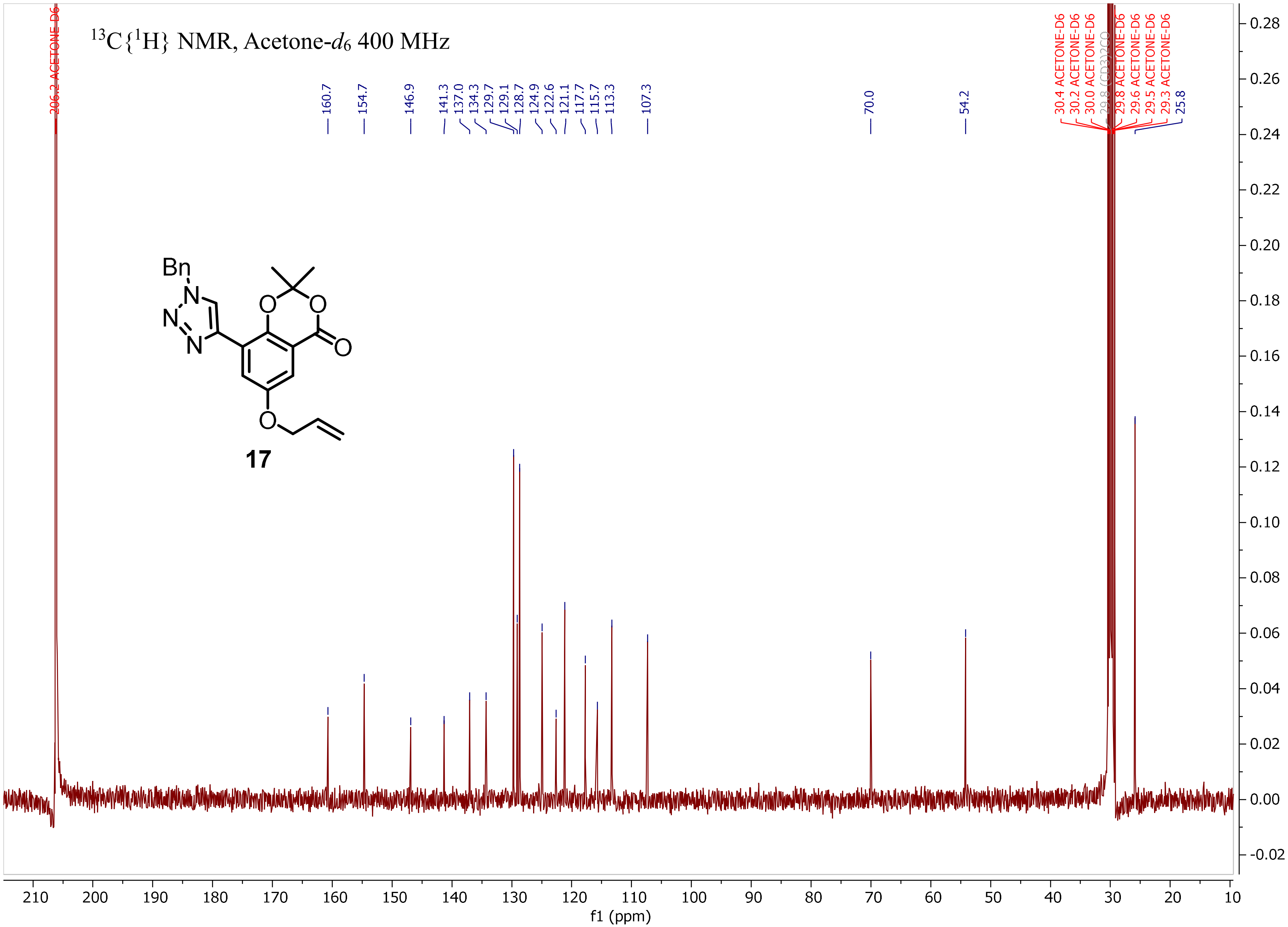

$^{13}C\{^1H\}$ NMR, Acetone-d_6 400 MHz
206.2 ACETONE-D6
160.7
154.7
146.9
141.3
137.0
134.3
129.7
129.1
128.7
124.9
122.6
121.1
117.7
115.7
113.3
107.3
70.0
54.2
30.4 ACETONE-D6
30.2 ACETONE-D6
30.0 ACETONE-D6
29.8 ACETONE-D6
29.6 ACETONE-D6
29.5 ACETONE-D6
29.3 ACETONE-D6
25.8
Bn
N
O
17
f1 (ppm)

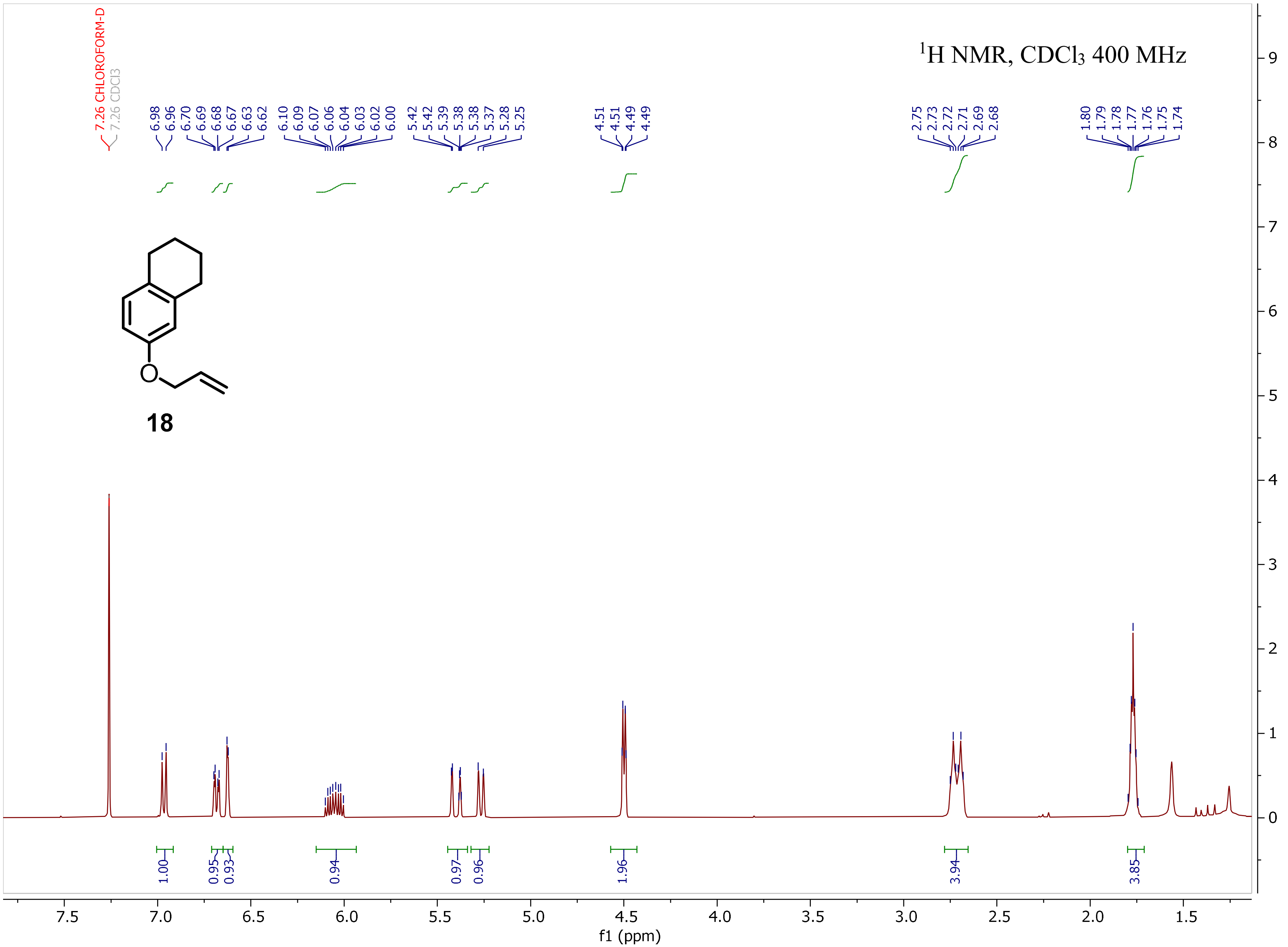

¹H NMR, CDCl3 400 MHz
18
f1 (ppm)

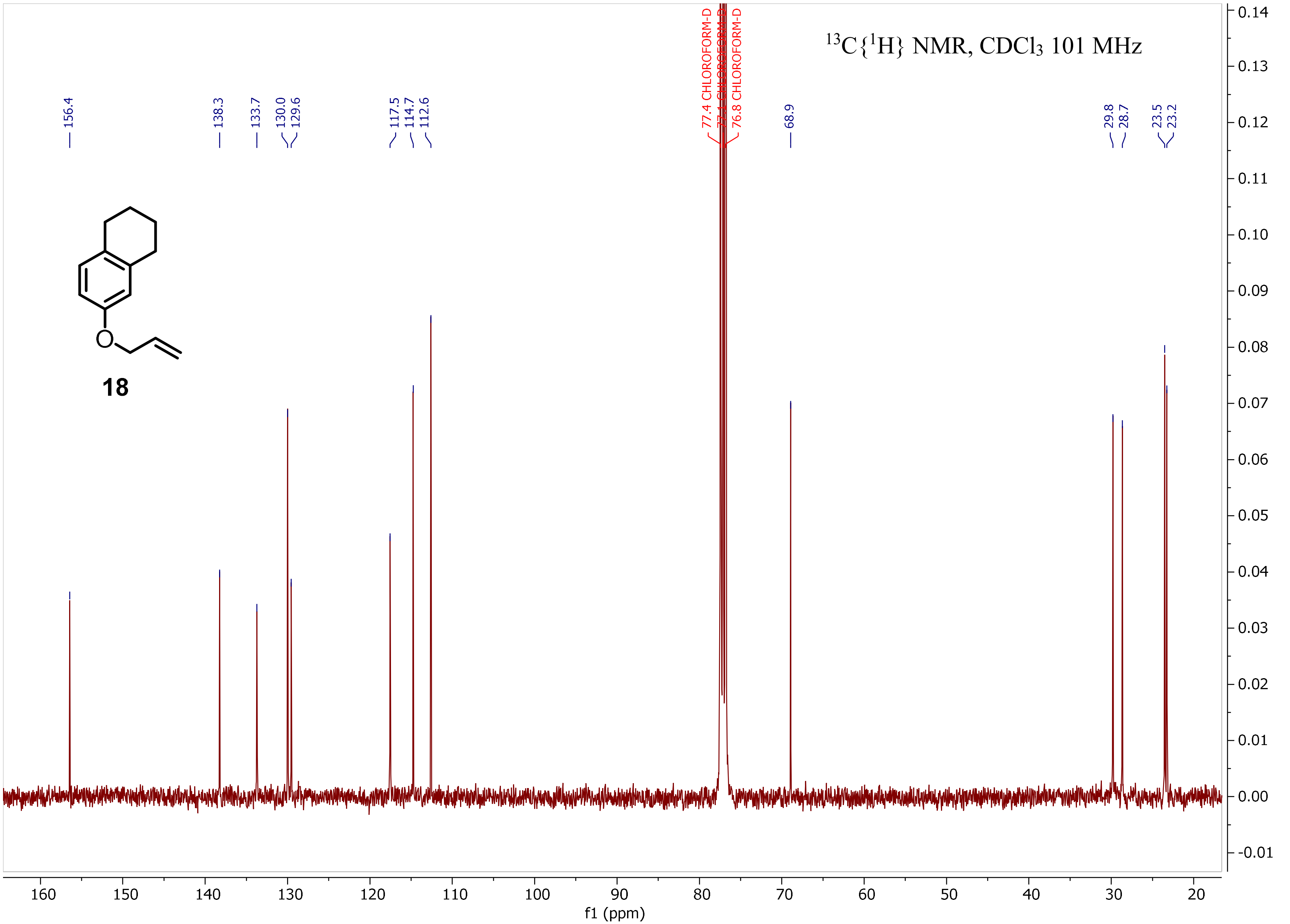

$^{13}C\{^{1}H\}$ NMR, $CDCl_3$ 101 MHz
156.4
138.3
133.7
130.0
129.6
117.5
114.7
112.6
77.4 CHLOROFORM-D
77.1 CHLOROFORM-D
76.8 CHLOROFORM-D
68.9
29.8
28.7
23.5
23.2
18
f1 (ppm)

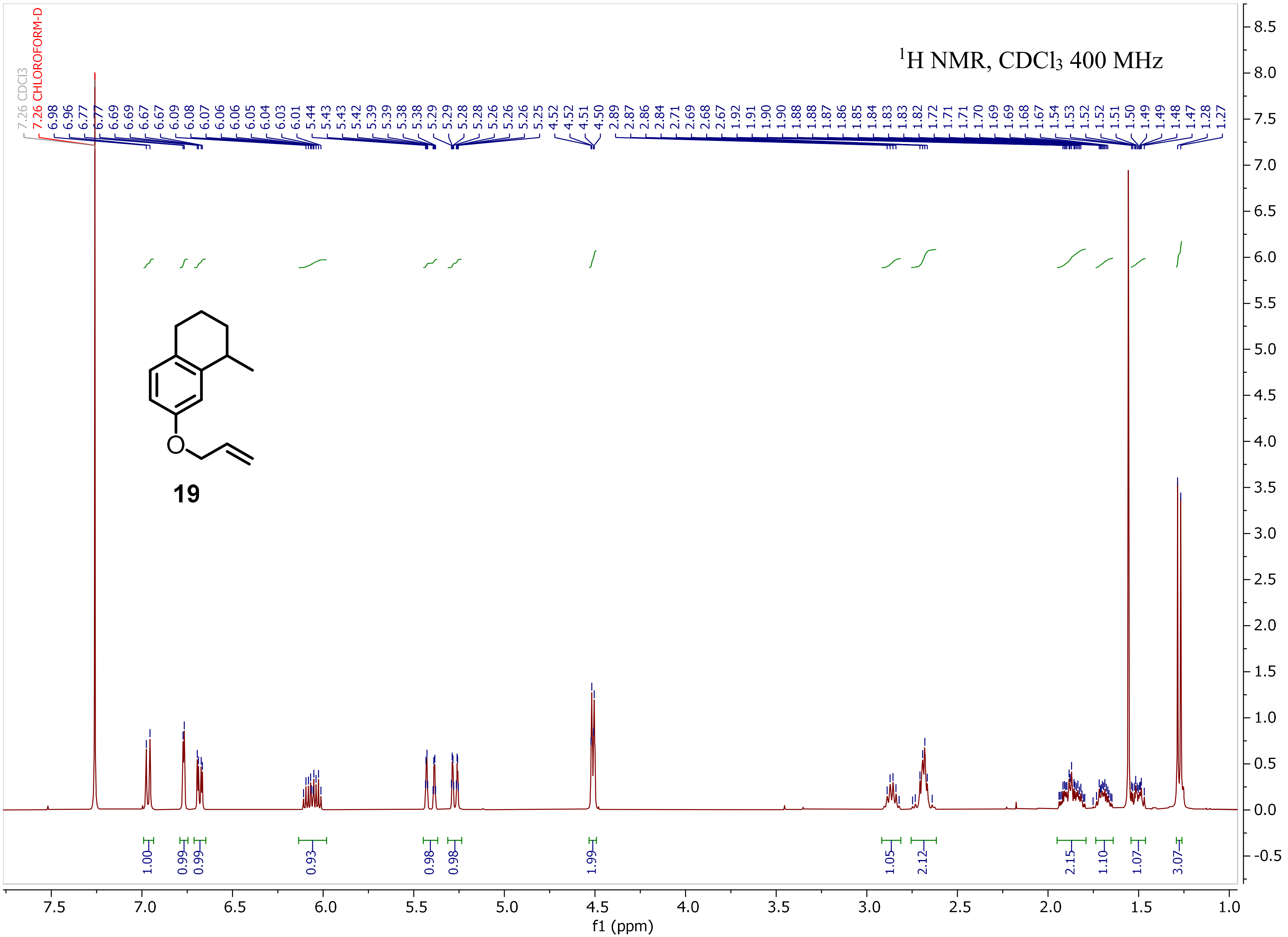

¹H NMR, CDCl3 400 MHz
19
7.26 CDCl3
7.26 CHLOROFORM-D
6.98
6.96
6.77
6.77
6.69
6.69
6.67
6.67
6.09
6.08
6.07
6.06
6.06
6.05
6.04
6.03
6.01
5.44
5.43
5.43
5.42
5.39
5.39
5.38
5.38
5.29
5.29
5.28
5.28
5.26
5.26
5.25
4.52
4.52
4.51
4.50
2.89
2.87
2.86
2.84
2.71
2.69
2.68
2.67
1.92
1.91
1.90
1.90
1.88
1.88
1.87
1.86
1.85
1.84
1.83
1.83
1.82
1.72
1.71
1.71
1.70
1.69
1.69
1.68
1.67
1.54
1.53
1.52
1.52
1.51
1.50
1.49
1.48
1.47
1.28
1.27
1.00
0.99
0.99
0.93
0.98
0.98
1.99
1.05
2.12
2.15
1.10
1.07
3.07
f1 (ppm)

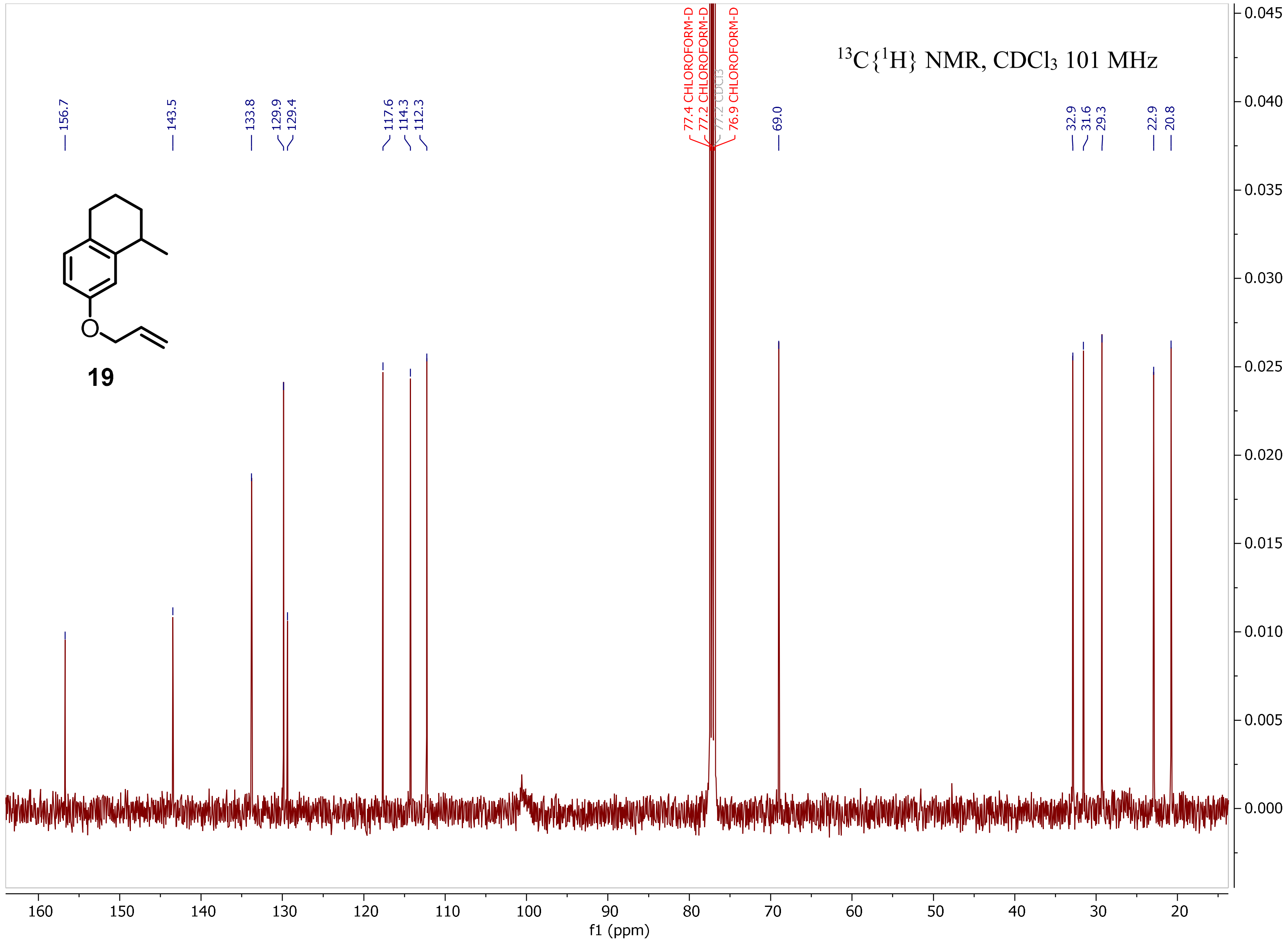

$^{13}C\{^1H\}$ NMR, $CDCl_3$ 101 MHz
19
156.7
143.5
133.8
129.9
129.4
117.6
114.3
112.3
77.4 CHLOROFORM-D
77.2 CHLOROFORM-D
77.2 CDCl3
76.9 CHLOROFORM-D
69.0
32.9
31.6
29.3
22.9
20.8
f1 (ppm)

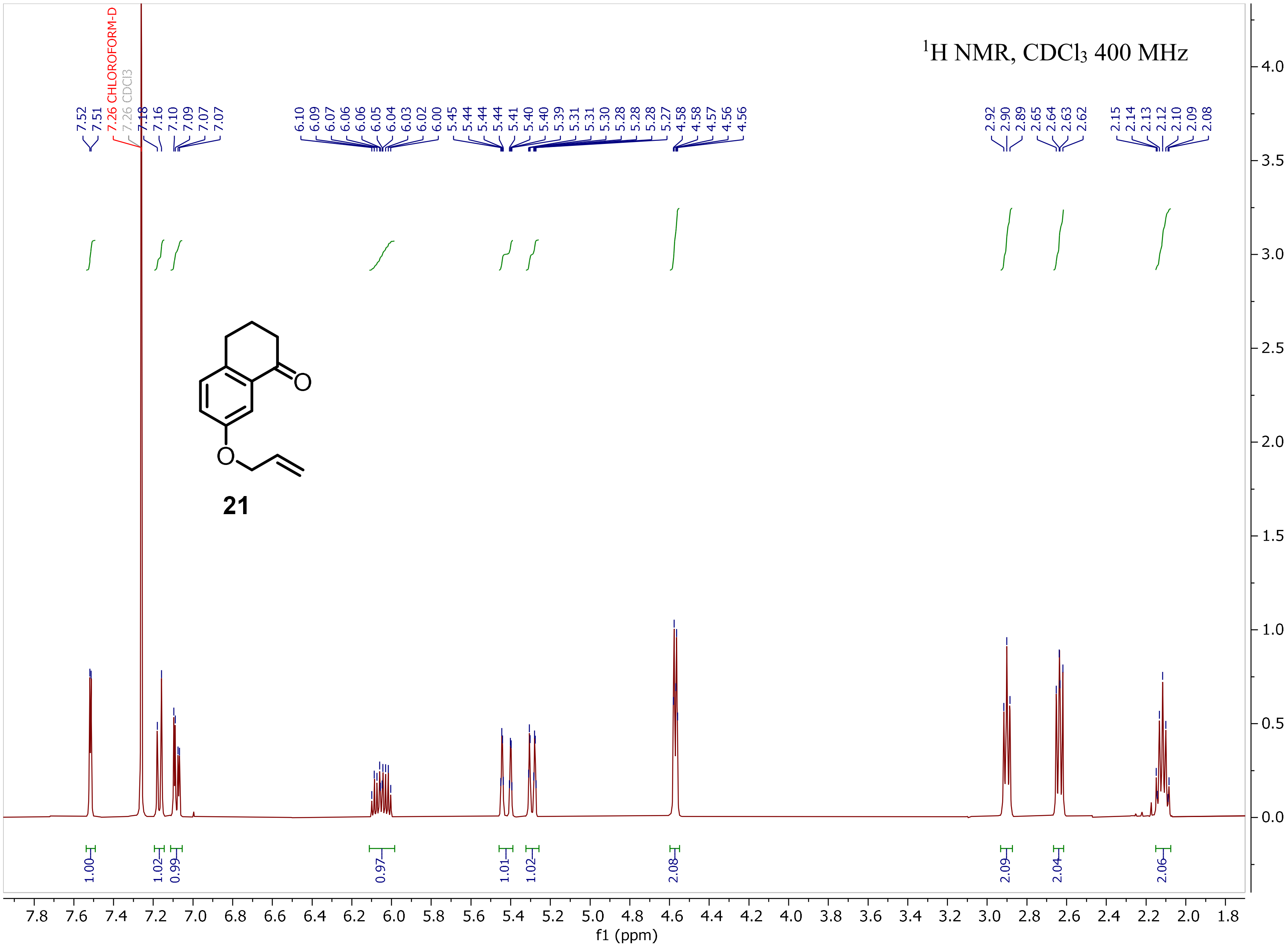

^{1}H NMR, CDCl3 400 MHz
21
f1 (ppm)

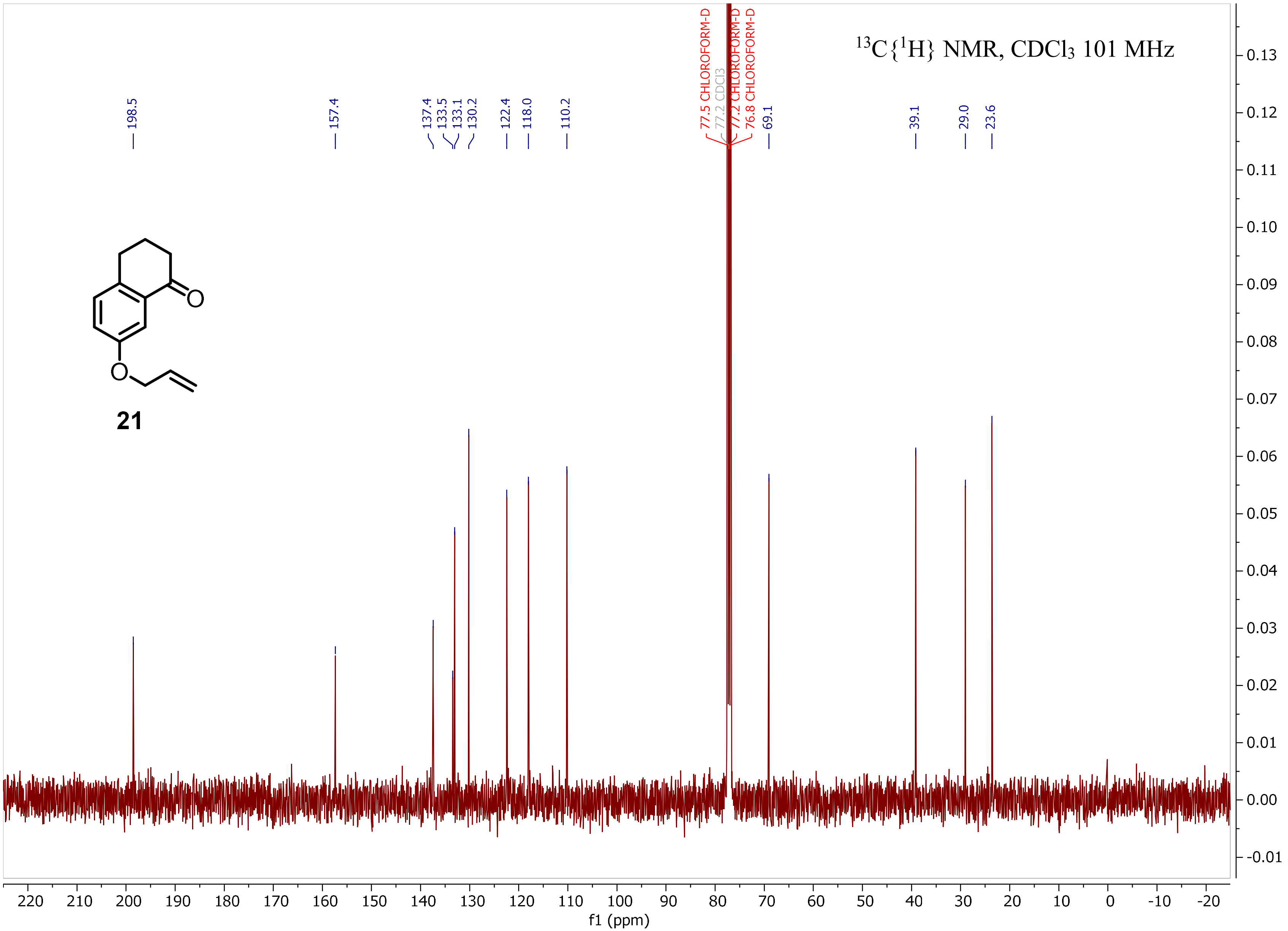

$^{13}C\{^{1}H\}$ NMR, $CDCl_3$ 101 MHz
198.5
157.4
137.4
133.5
133.1
130.2
122.4
118.0
110.2
77.5 CHLOROFORM-D
77.2 CDCl3
77.2 CHLOROFORM-D
76.8 CHLOROFORM-D
69.1
39.1
29.0
23.6
21
f1 (ppm)

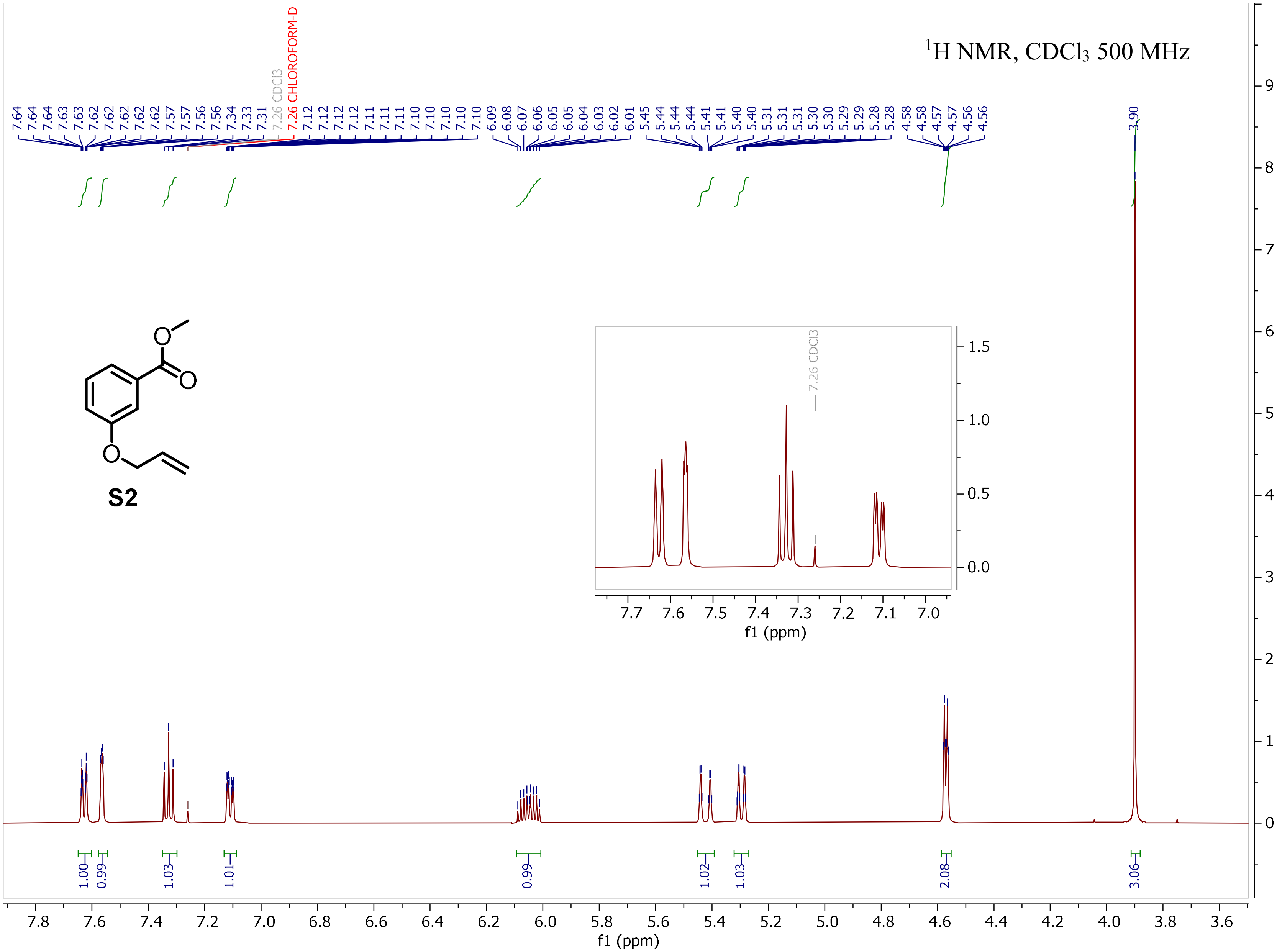

1H NMR, CDCl3 500 MHz
S2
7.26 CHLOROFORM-D
7.26 CDCl3
f1 (ppm)

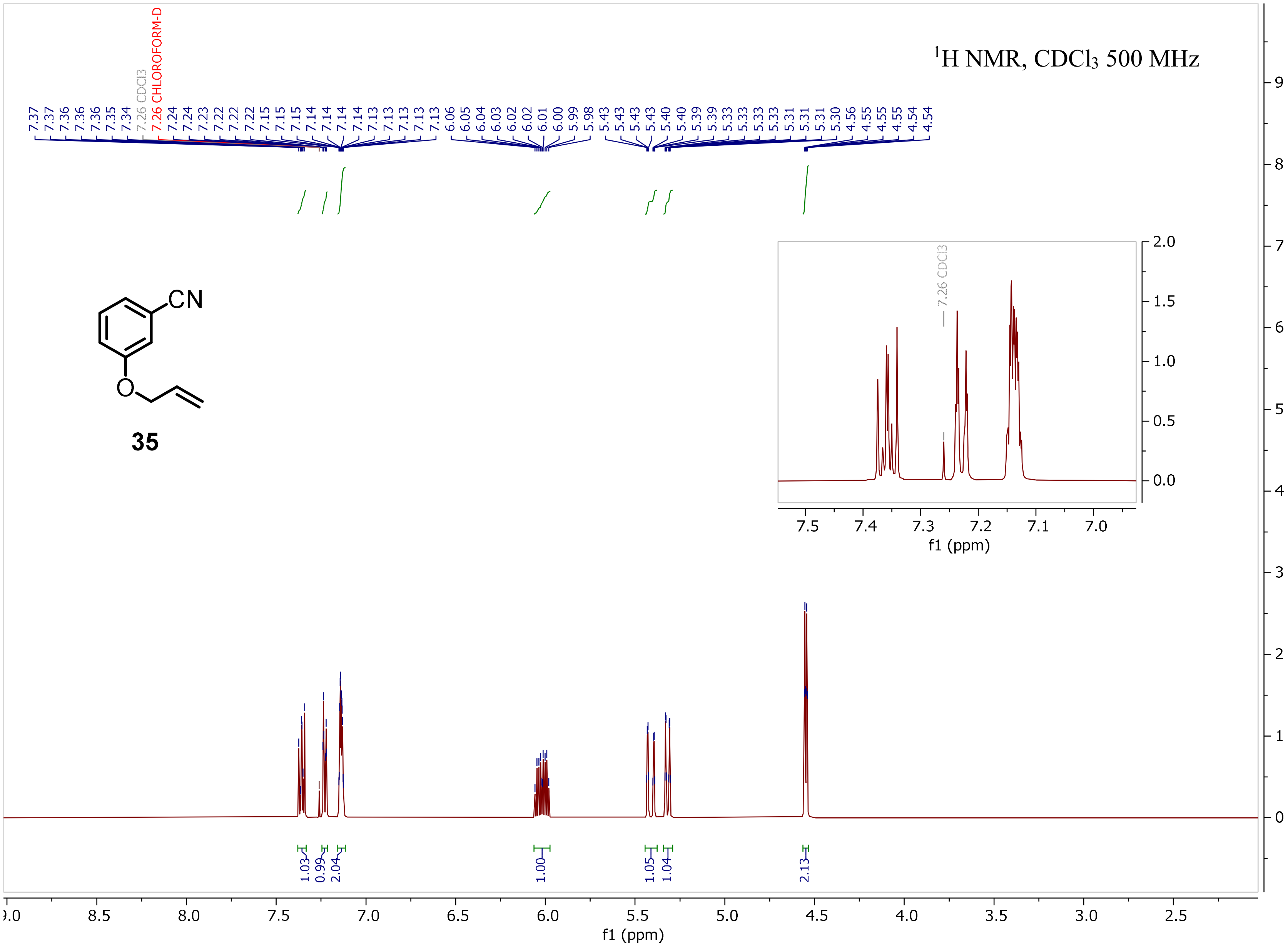

1H NMR, CDCl3 500 MHz
7.37 7.37 7.36 7.36 7.35 7.34 7.26 CDCl3 7.26 CHLOROFORM-D 7.24 7.24 7.23 7.22 7.22 7.22 7.15 7.15 7.14 7.14 7.14 7.14 7.13 7.13 7.13 7.13 7.13 6.06 6.05 6.04 6.03 6.02 6.02 6.01 6.00 5.99 5.98 5.43 5.43 5.43 5.43 5.40 5.40 5.39 5.39 5.33 5.33 5.33 5.33 5.31 5.31 5.31 5.30 4.56 4.55 4.55 4.55 4.54 4.54
CN
O
35
7.26 CDCl3
f1 (ppm)
1.03 0.99 2.04 1.00 1.05 1.04 2.13
f1 (ppm)

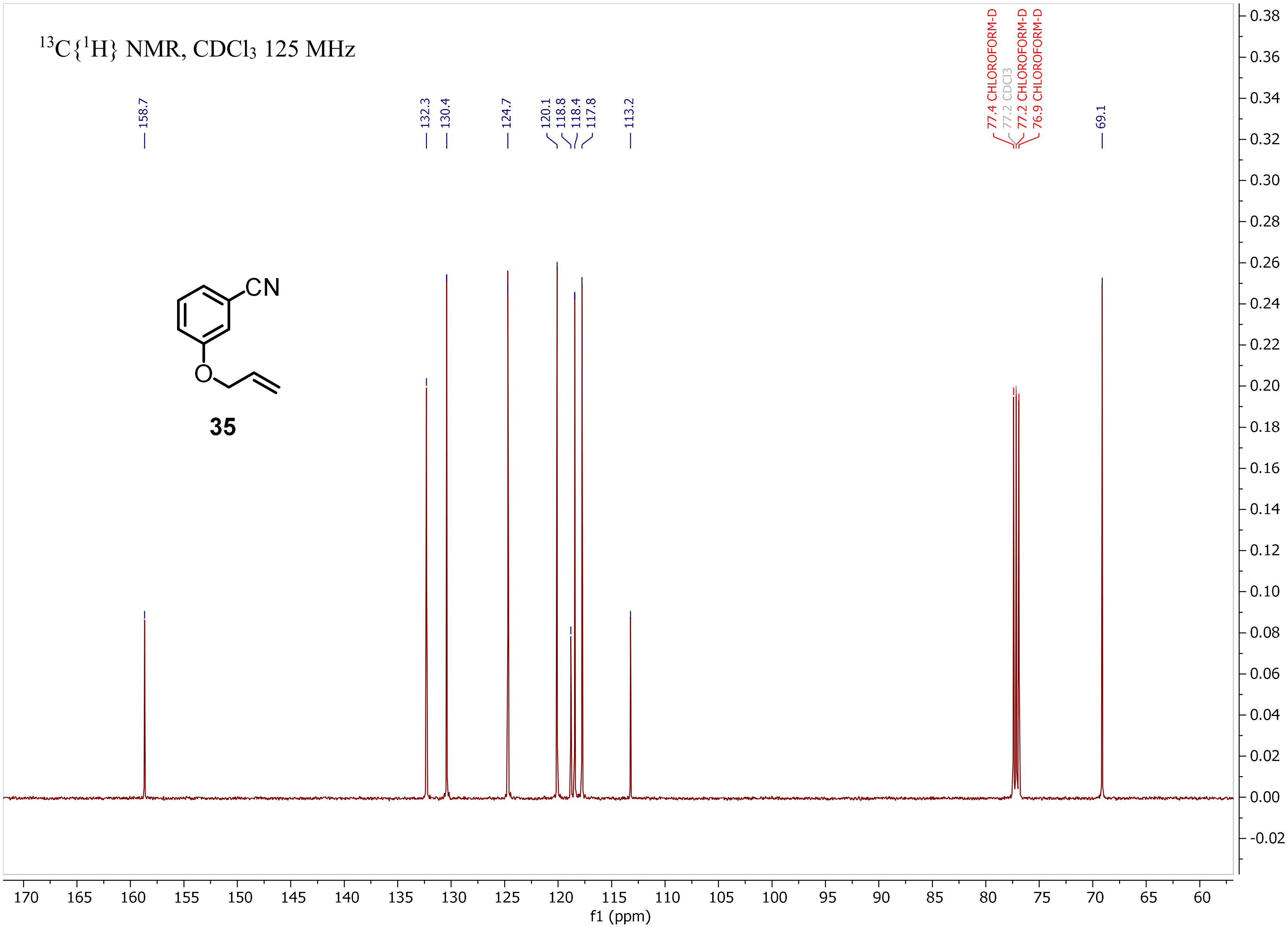
13C{1H} NMR, CDCl3 125 MHz
158.7
132.3
130.4
124.7
120.1
118.8
118.4
117.8
113.2
77.4 CHLOROFORM-D
77.2 CDCl3
77.2 CHLOROFORM-D
76.9 CHLOROFORM-D
69.1
CN
O
35
f1 (ppm)

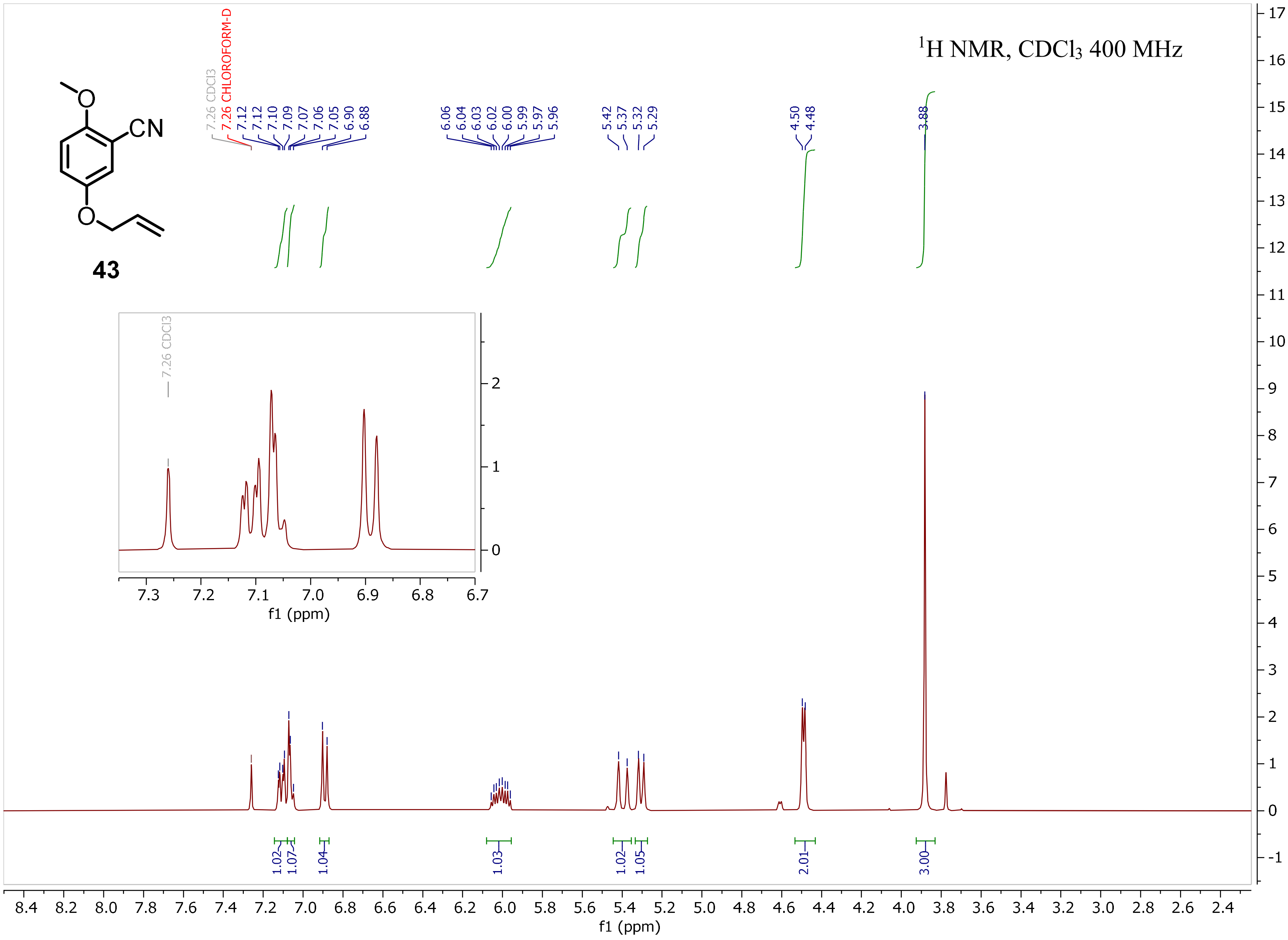

^{1}H NMR, CDCl$_3$ 400 MHz
O
CN
O
43
7.26 CDCl3
7.26 CHLOROFORM-D
7.12
7.12
7.10
7.09
7.07
7.06
7.05
6.90
6.88
6.06
6.04
6.03
6.02
6.00
5.99
5.97
5.96
5.42
5.37
5.32
5.29
4.50
4.48
3.88
1.02
1.07
1.04
1.03
1.02
1.05
2.01
3.00
f1 (ppm)

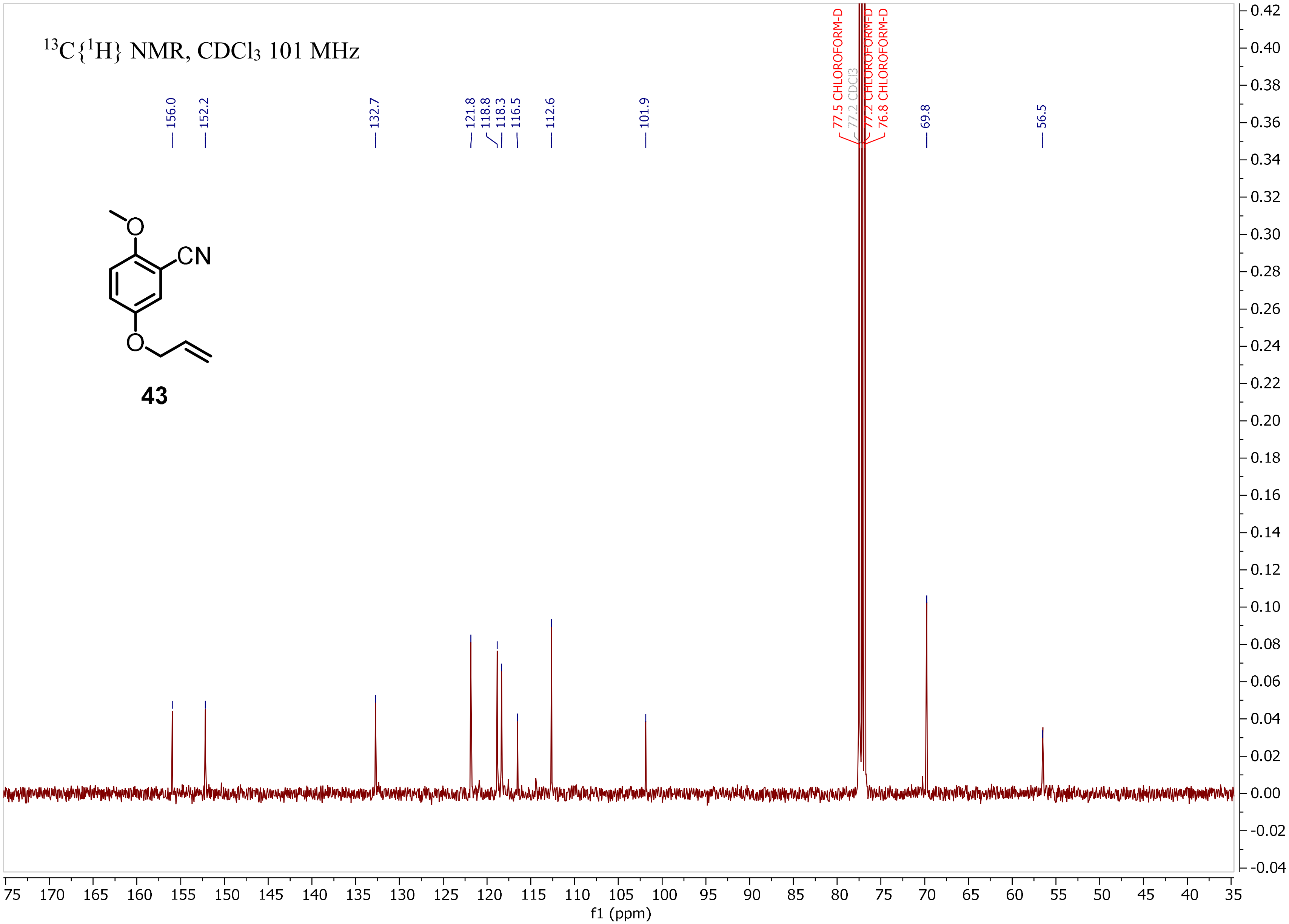

$^{13}C\{^{1}H\}$ NMR, $CDCl_3$ 101 MHz
156.0
152.2
132.7
121.8
118.8
118.3
116.5
112.6
101.9
77.5 CHLOROFORM-D
77.2 CDCl3
77.2 CHLOROFORM-D
76.8 CHLOROFORM-D
69.8
56.5
O
CN
O
43
f1 (ppm)

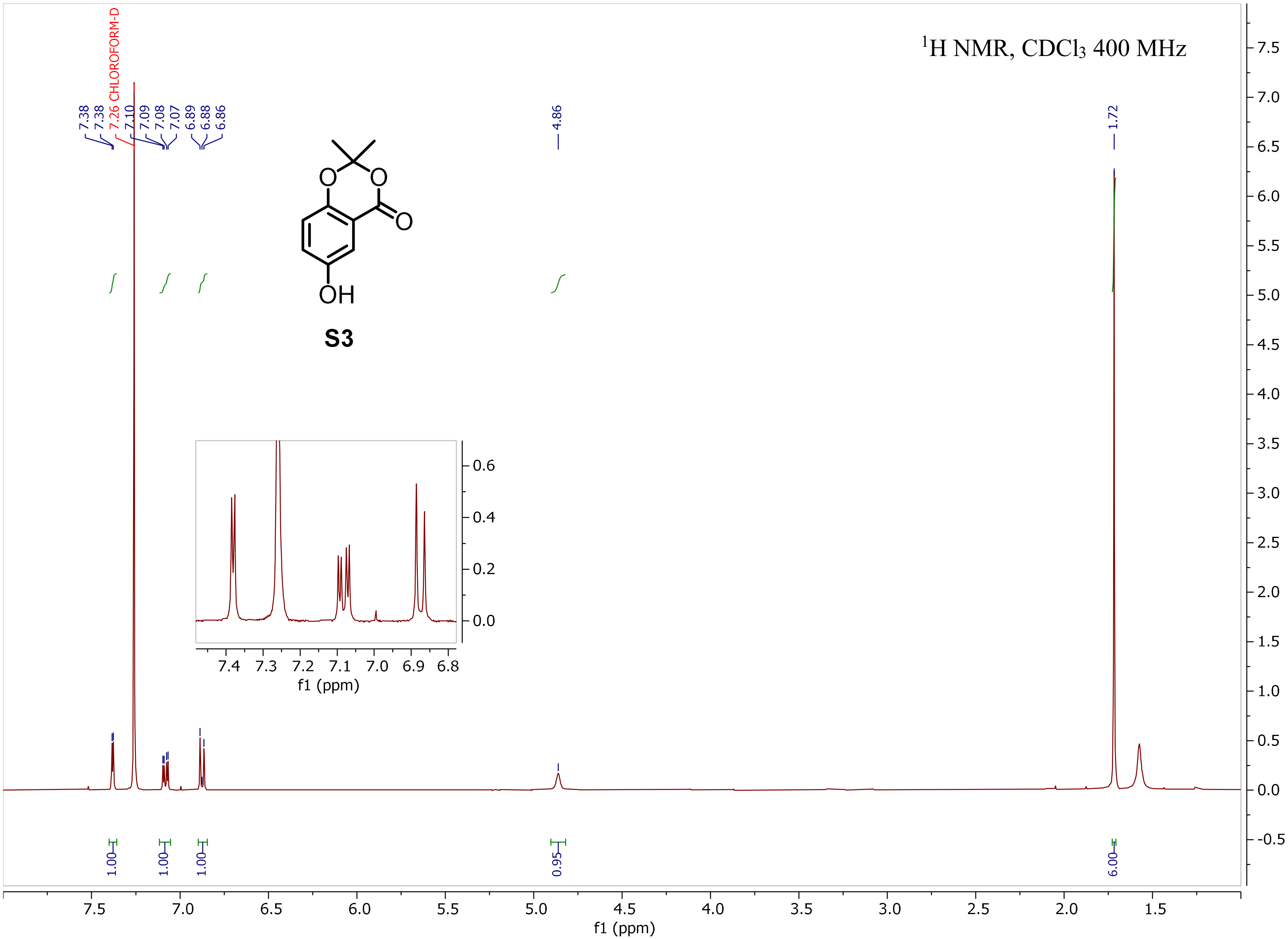

1H NMR, CDCl3 400 MHz
7.38
7.38
7.26 CHLOROFORM-D
7.10
7.09
7.08
7.07
6.89
6.88
6.86
4.86
1.72
OH
S3
1.00
1.00
1.00
0.95
6.00
f1 (ppm)

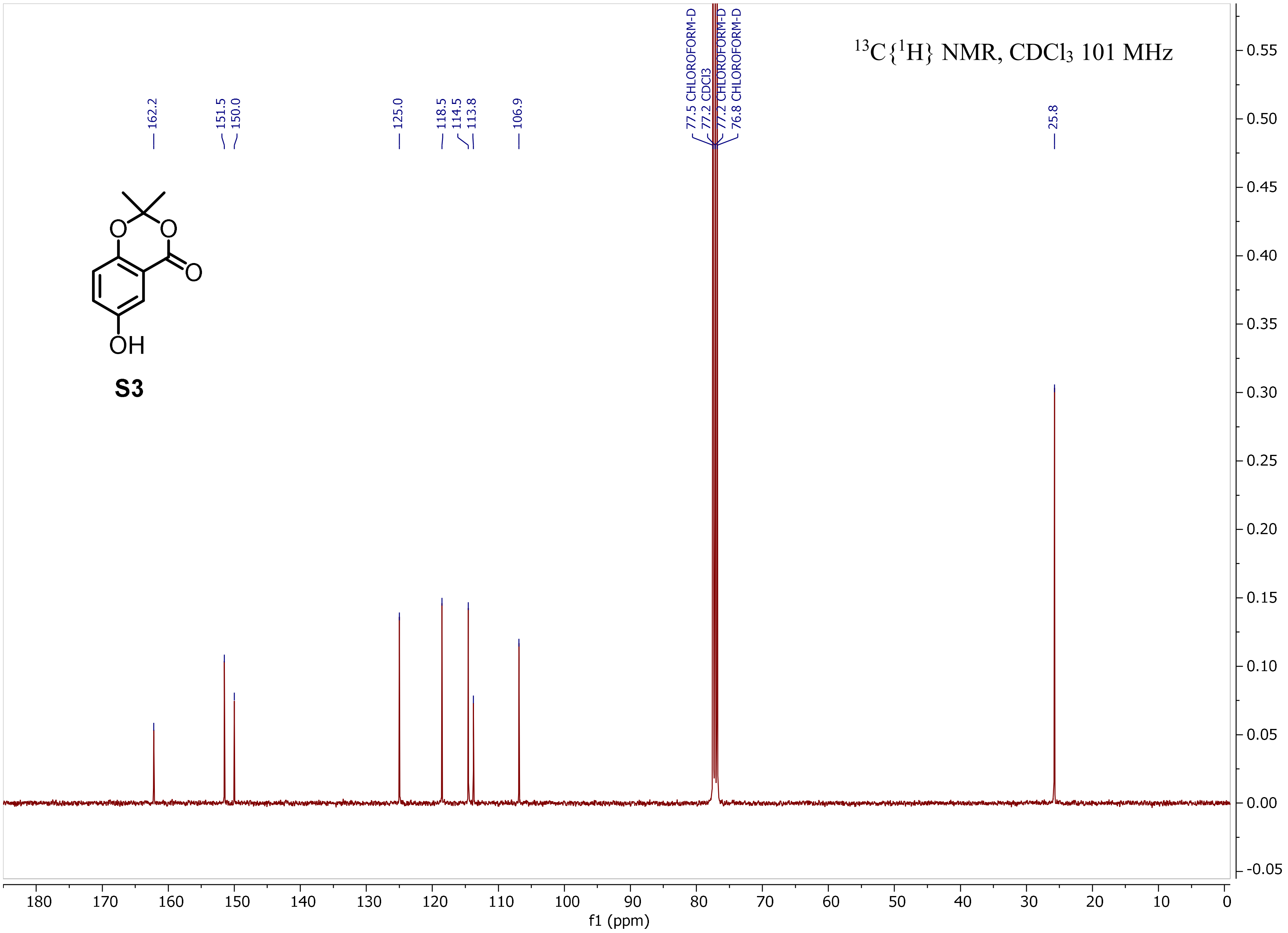

$^{13}C\{^1H\}$ NMR, $CDCl_3$ 101 MHz
162.2
151.5
150.0
125.0
118.5
114.5
113.8
106.9
77.5 CHLOROFORM-D
77.2 CDCl3
77.2 CHLOROFORM-D
76.8 CHLOROFORM-D
25.8
OH
O
S3
f1 (ppm)

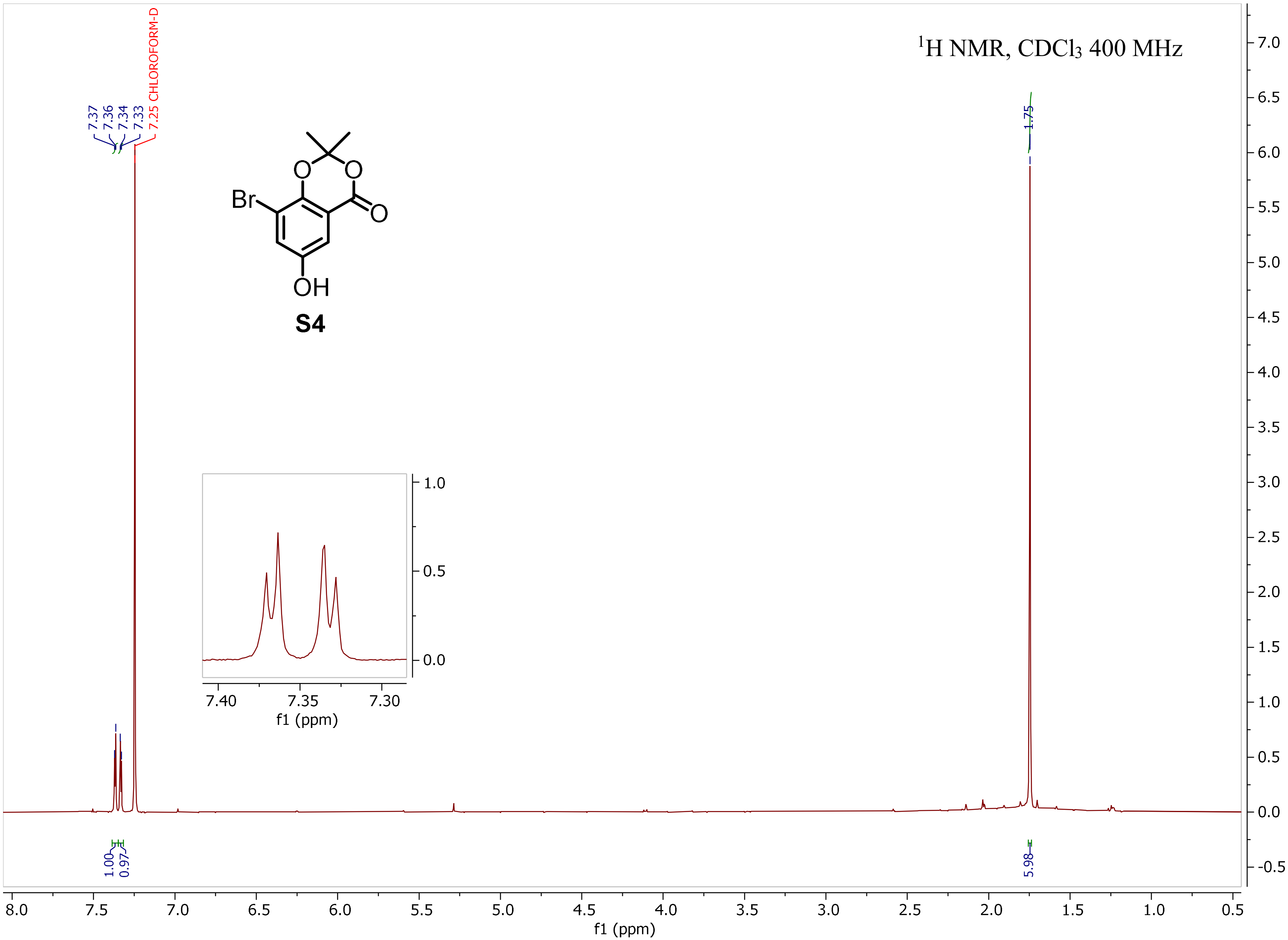

¹H NMR, CDCl3 400 MHz
7.37
7.36
7.34
7.33
7.25 CHLOROFORM-D
1.75
Br
O
O
O
OH
S4
1.00
0.97
5.98
f1 (ppm)

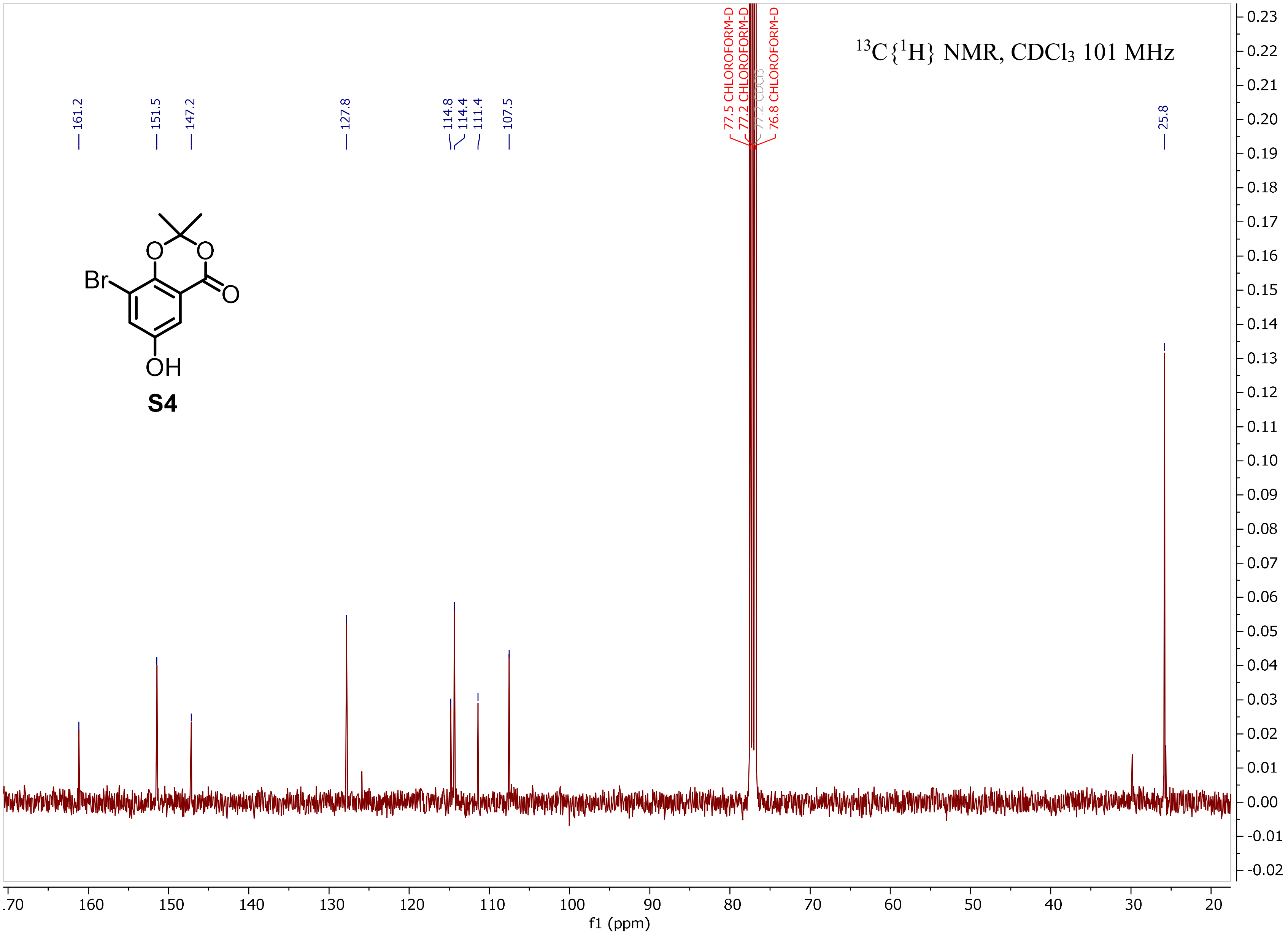

13C{1H} NMR, CDCl3 101 MHz
161.2
151.5
147.2
127.8
114.8
114.4
111.4
107.5
77.5 CHLOROFORM-D
77.2 CHLOROFORM-D
77.2 CDCl3
76.8 CHLOROFORM-D
25.8
Br
O
O
O
OH
S4
f1 (ppm)

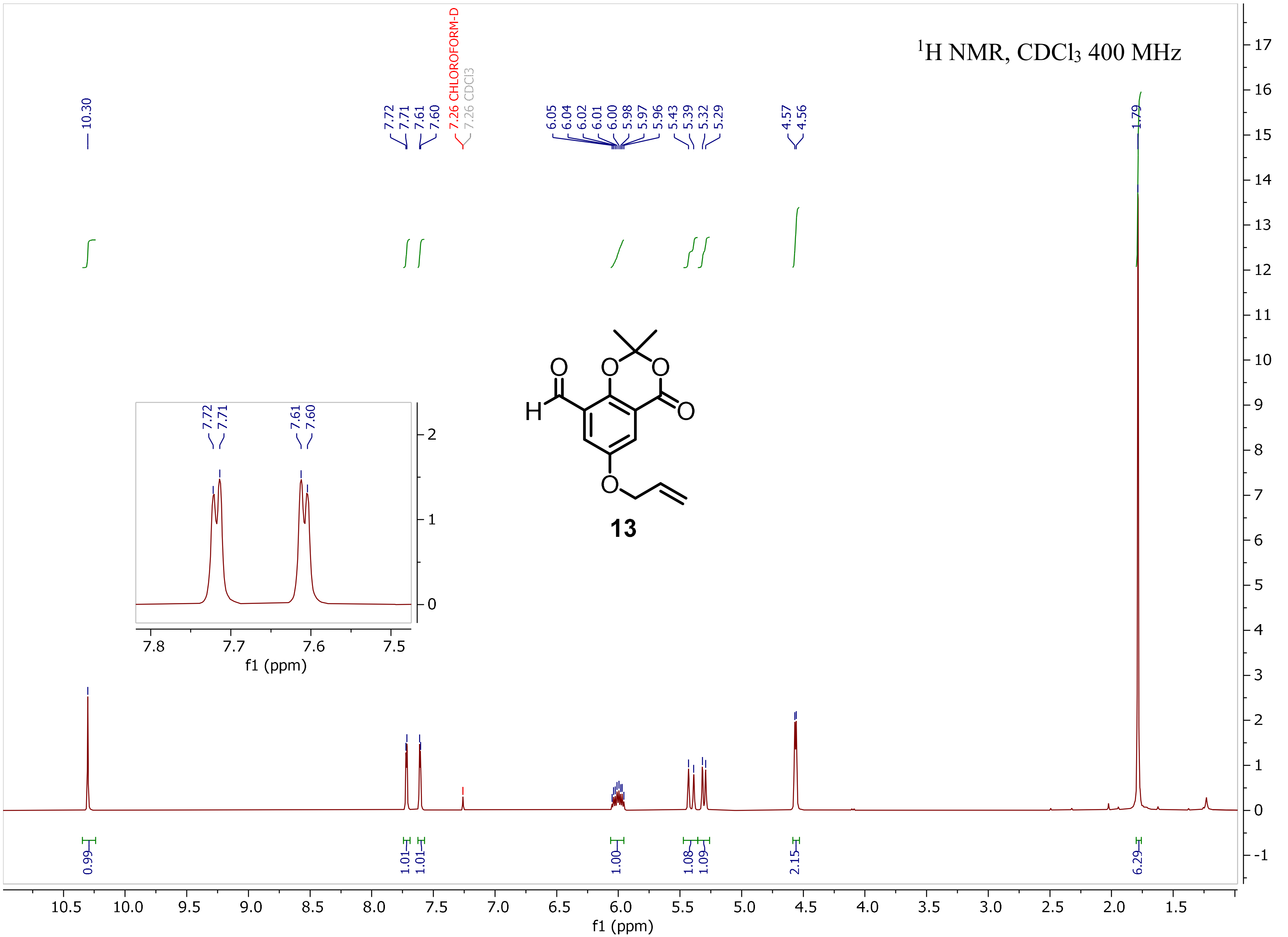

^{1}H NMR, CDCl3 400 MHz
10.30
7.72
7.71
7.61
7.60
7.26 CHLOROFORM-D
7.26 CDCl3
6.05
6.04
6.02
6.01
6.00
5.98
5.97
5.96
5.43
5.39
5.32
5.29
4.57
4.56
1.79
0.99
1.01
1.01
1.00
1.08
1.09
2.15
6.29
f1 (ppm)
13

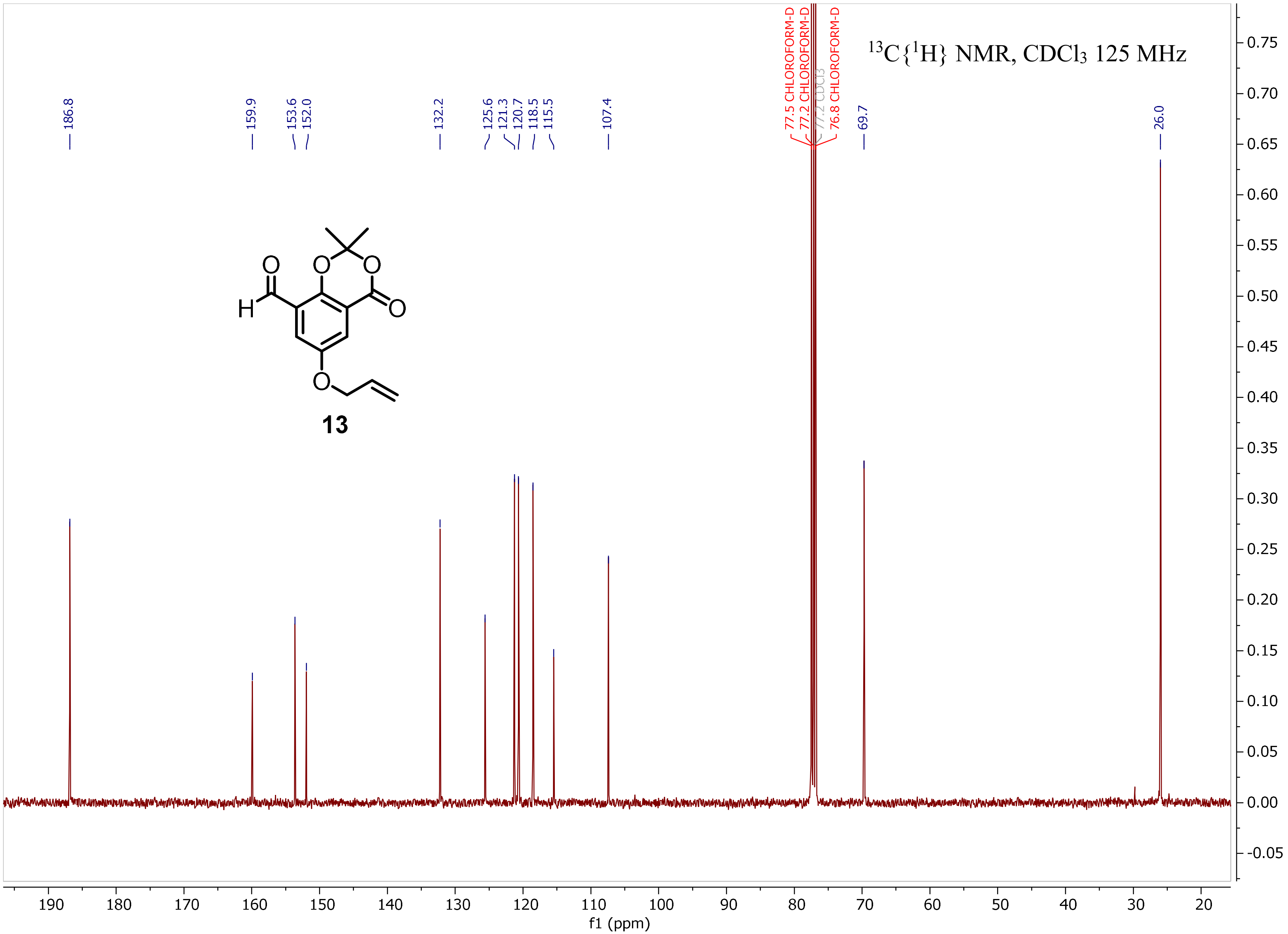

$^{13}C\{^1H\}$ NMR, $CDCl_3$ 125 MHz
186.8
159.9
153.6
152.0
132.2
125.6
121.3
120.7
118.5
115.5
107.4
77.5 CHLOROFORM-D
77.2 CHLOROFORM-D
77.2 CDCl3
76.8 CHLOROFORM-D
69.7
26.0
13
f1 (ppm)

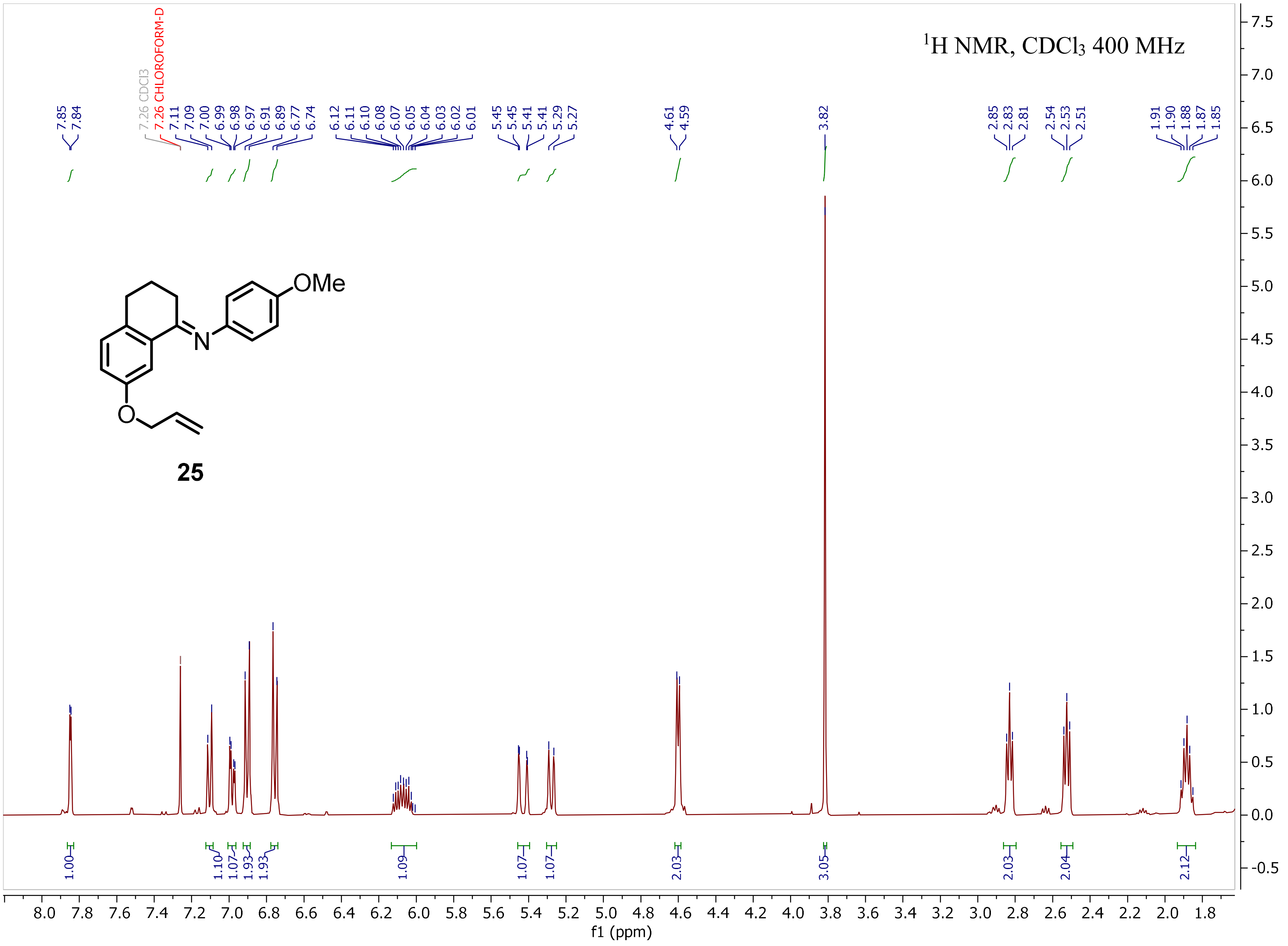

1H NMR, CDCl3 400 MHz
7.85 7.84 7.26 CDCl3 7.26 CHLOROFORM-D 7.11 7.09 7.00 6.99 6.98 6.97 6.91 6.89 6.77 6.74 6.12 6.11 6.10 6.08 6.07 6.05 6.04 6.03 6.02 6.01 5.45 5.45 5.41 5.41 5.29 5.27 4.61 4.59 3.82 2.85 2.83 2.81 2.54 2.53 2.51 1.91 1.90 1.88 1.87 1.85
OMe
N
O
25
1.00 1.10 1.07 1.93 1.93 1.09 1.07 1.07 2.03 3.05 2.03 2.04 2.12
f1 (ppm)

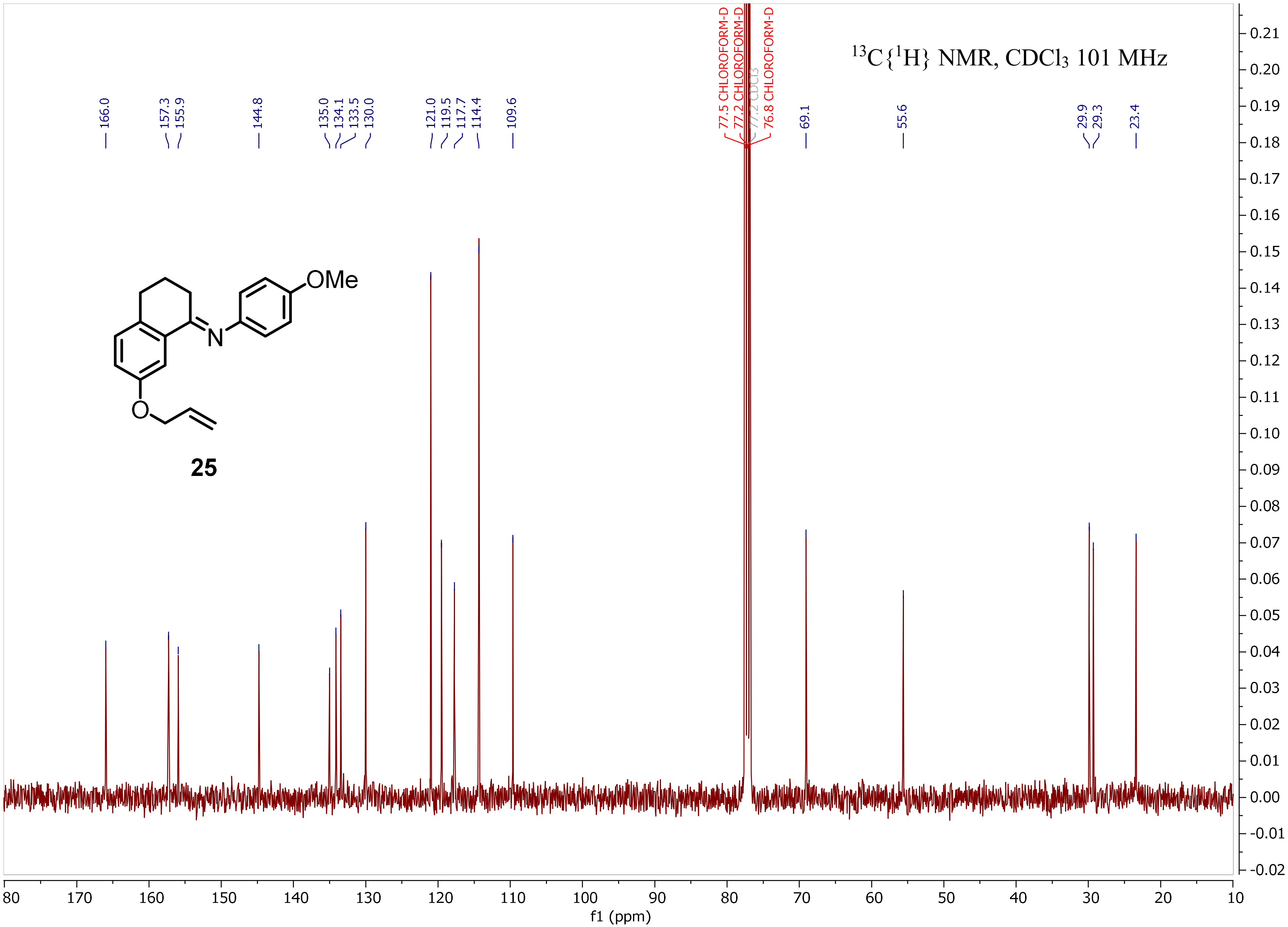

¹³C{¹H} NMR, CDCl₃ 101 MHz
166.0
157.3
155.9
144.8
135.0
134.1
133.5
130.0
121.0
119.5
117.7
114.4
109.6
77.5 CHLOROFORM-D
77.2 CHLOROFORM-D
77.2 CDCl3
76.8 CHLOROFORM-D
69.1
55.6
29.9
29.3
23.4
OMe
N
O
25
f1 (ppm)

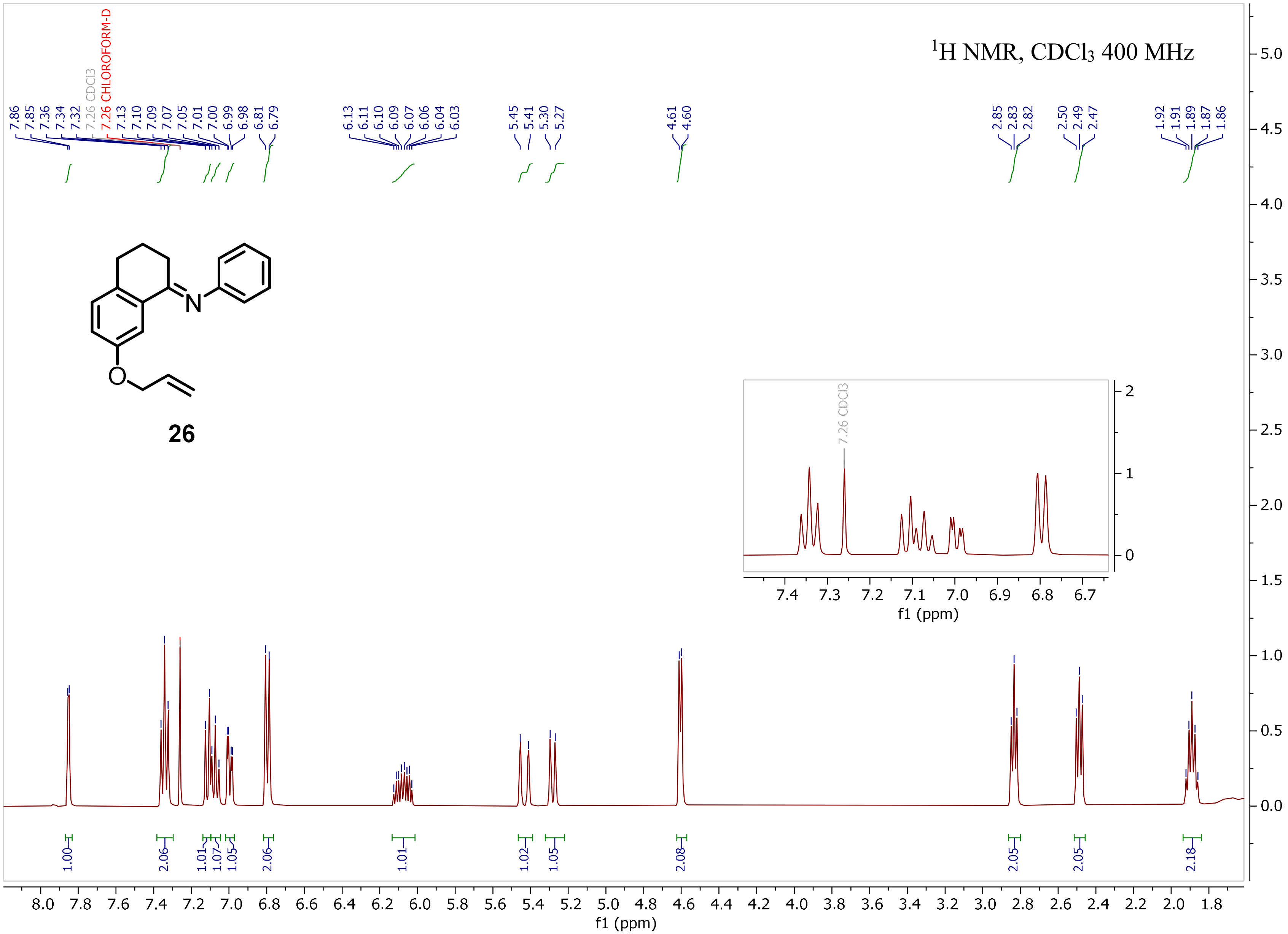

^{1}H NMR, CDCl$_3$ 400 MHz
7.86
7.85
7.36
7.34
7.32
7.26 CDCl3
7.26 CHLOROFORM-D
7.13
7.10
7.09
7.07
7.05
7.01
7.00
6.99
6.98
6.81
6.79
6.13
6.11
6.10
6.09
6.07
6.06
6.04
6.03
5.45
5.41
5.30
5.27
4.61
4.60
2.85
2.83
2.82
2.50
2.49
2.47
1.92
1.91
1.89
1.87
1.86
N
O
26
1.00
2.06
1.01
1.07
1.05
2.06
1.01
1.02
1.05
2.08
2.05
2.05
2.18
f1 (ppm)

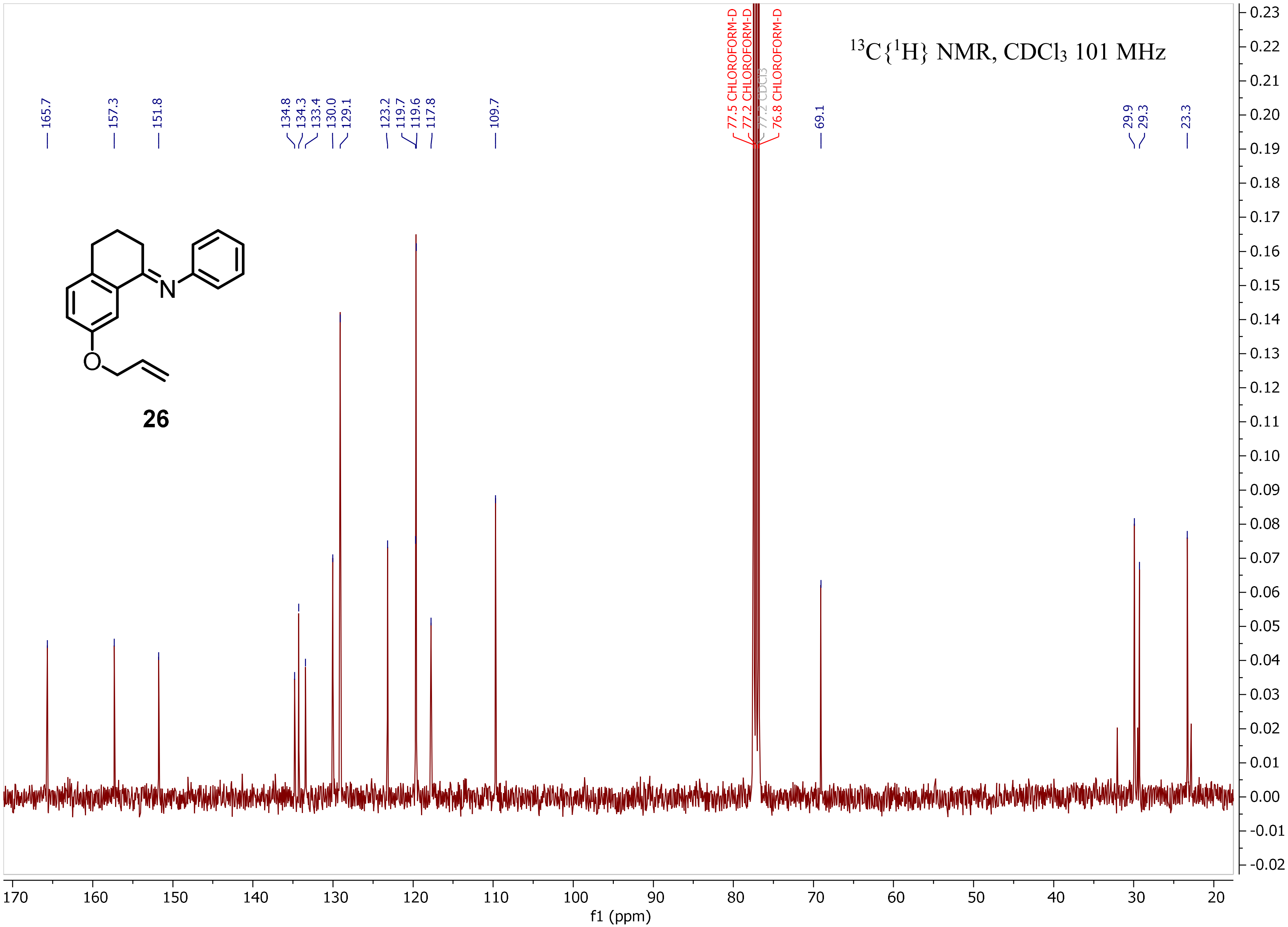

$^{13}C\{^{1}H\}$ NMR, $CDCl_3$ 101 MHz
165.7
157.3
151.8
134.8
134.3
133.4
130.0
129.1
123.2
119.7
119.6
117.8
109.7
77.5 CHLOROFORM-D
77.2 CHLOROFORM-D
77.2 CDCl3
76.8 CHLOROFORM-D
69.1
29.9
29.3
23.3
N
O
26
f1 (ppm)

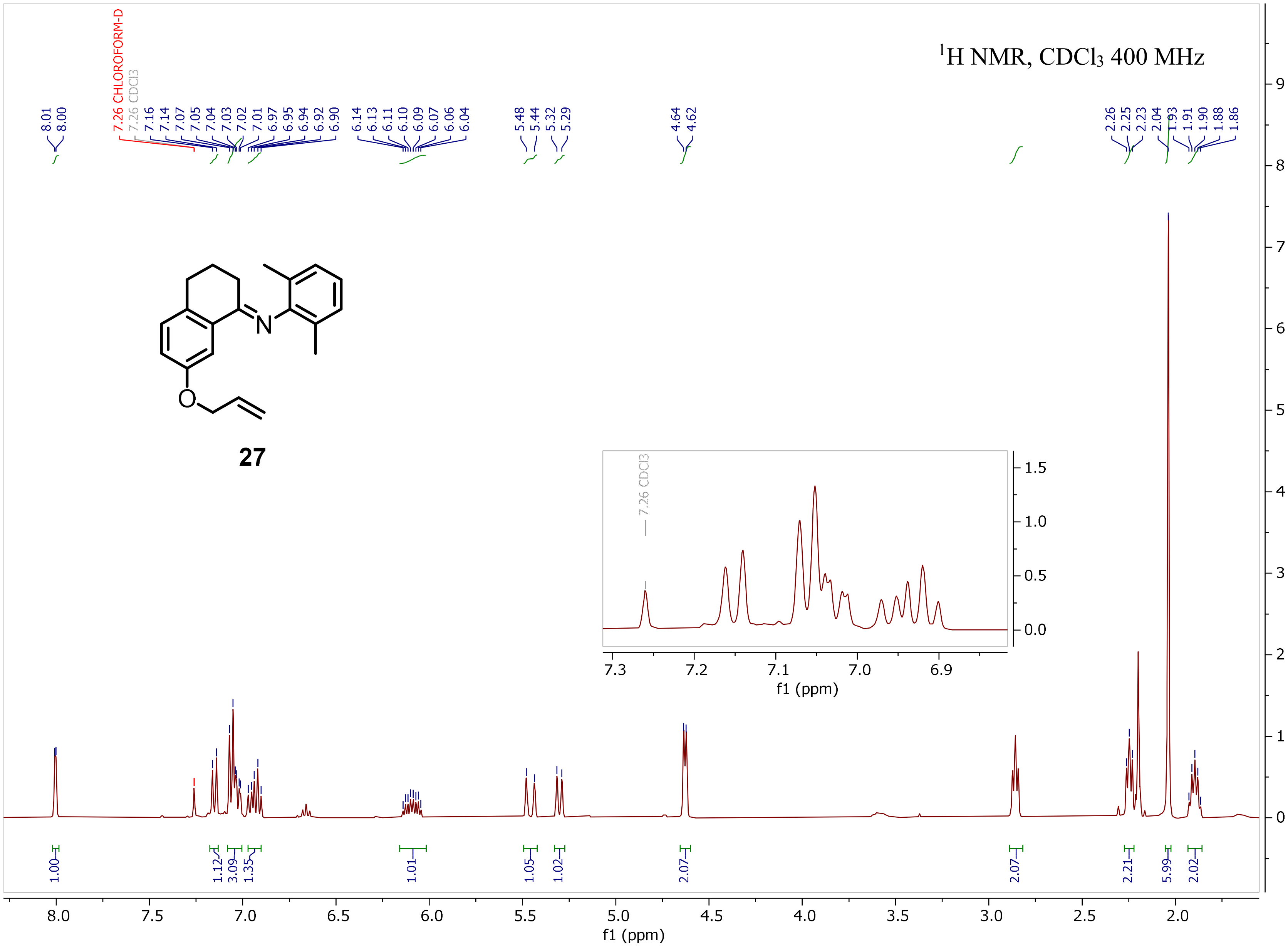
1H NMR, CDCl3 400 MHz
27
f1 (ppm)

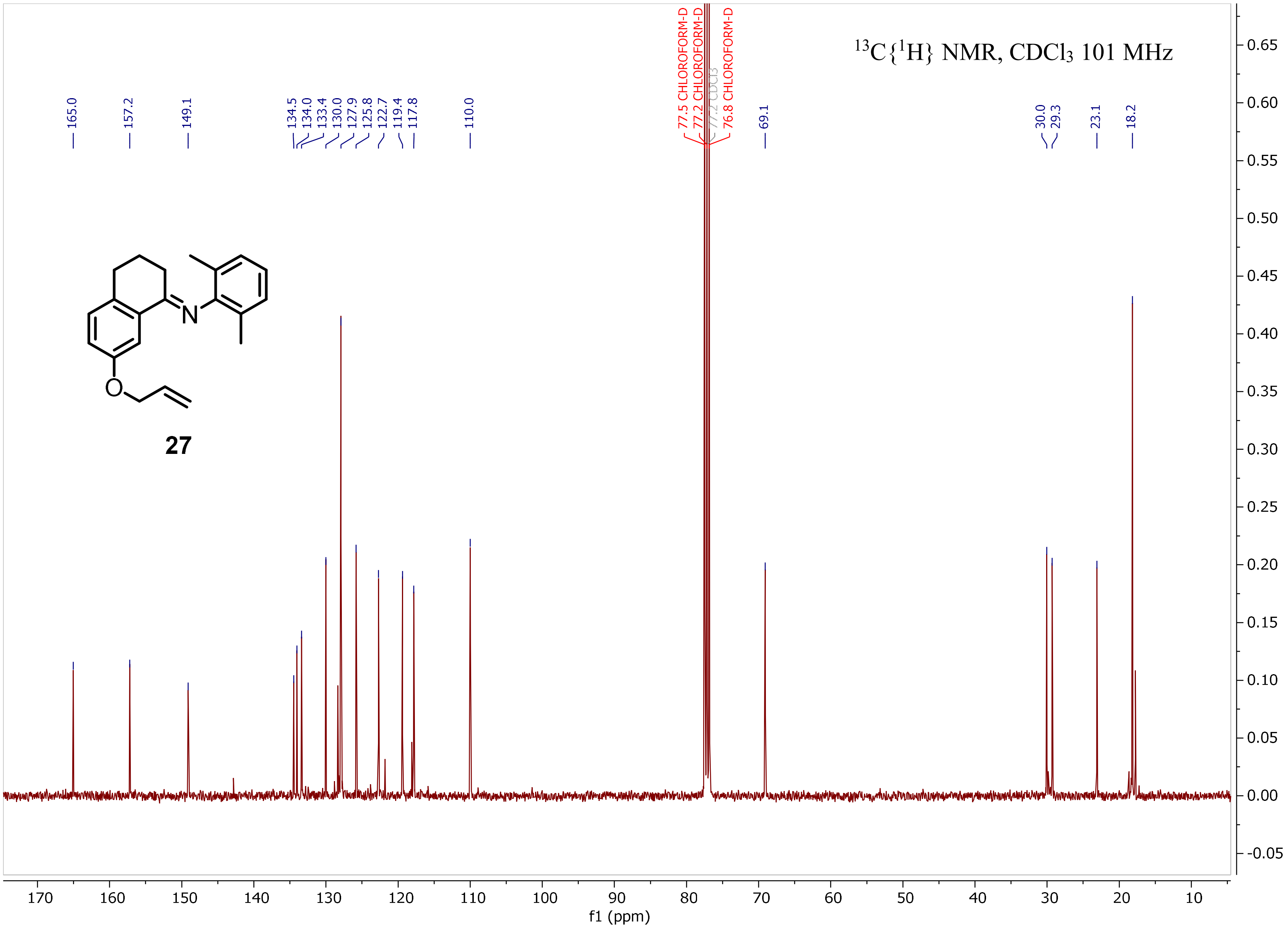

$^{13}C\{^1H\}$ NMR, CDCl3 101 MHz
165.0
157.2
149.1
134.5
134.0
133.4
130.0
127.9
125.8
122.7
119.4
117.8
110.0
77.5 CHLOROFORM-D
77.2 CHLOROFORM-D
77.2 CDCl3
76.8 CHLOROFORM-D
69.1
30.0
29.3
23.1
18.2
N
O
27
f1 (ppm)

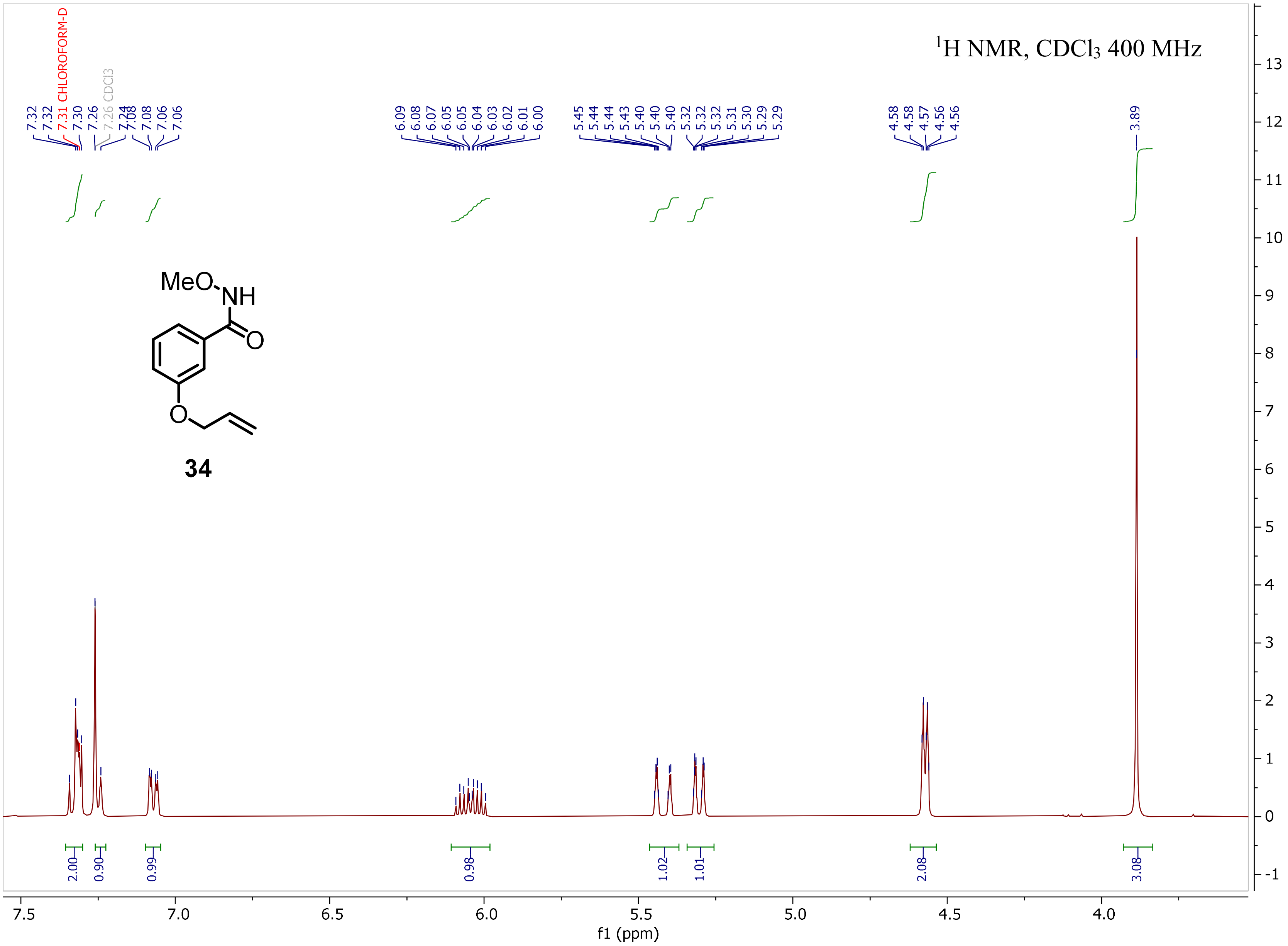

1H NMR, CDCl3 400 MHz
MeO
NH
O
O
34
f1 (ppm)

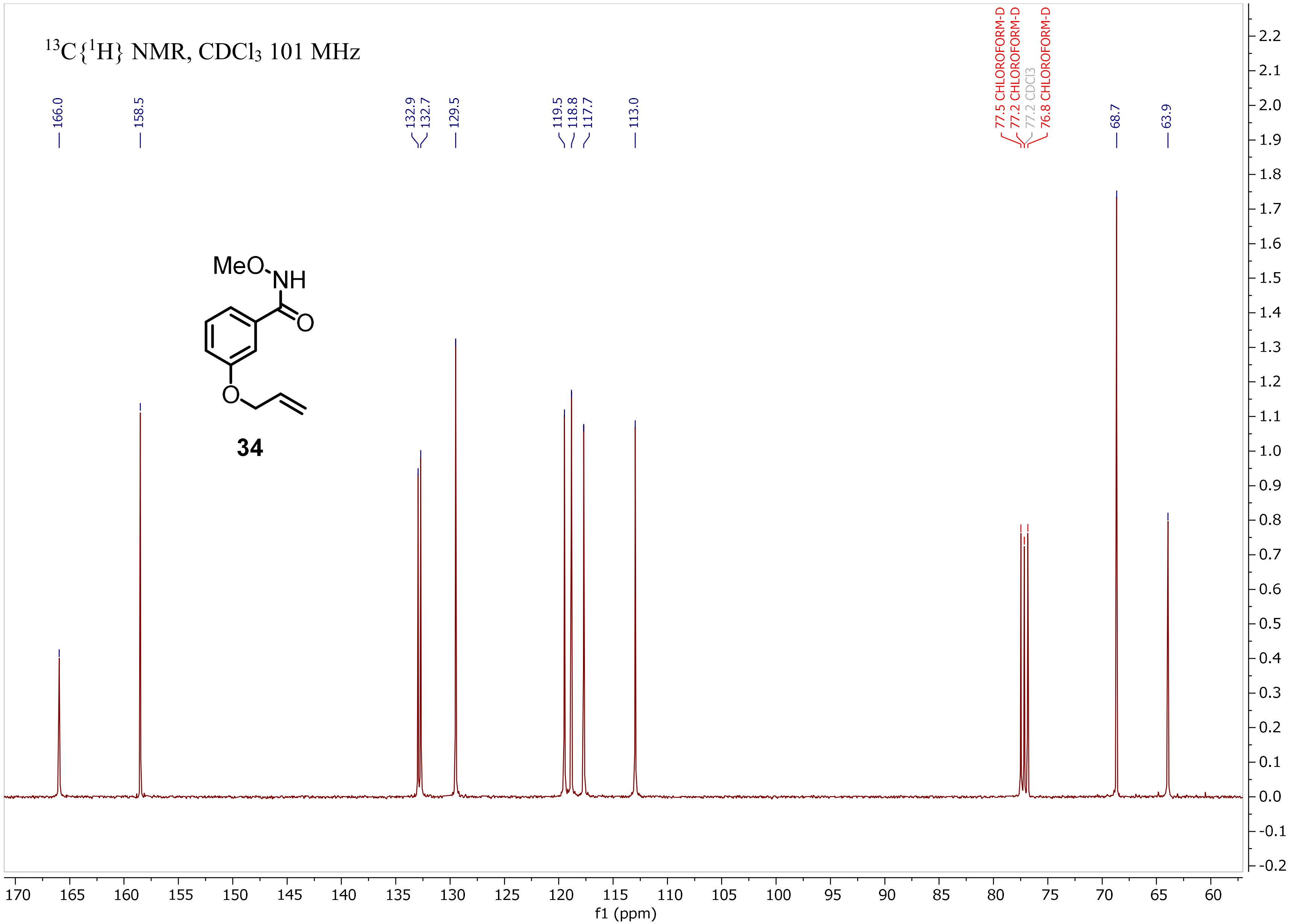
$^{13}C\{^{1}H\}$ NMR, $CDCl_3$ 101 MHz
166.0
158.5
132.9
132.7
129.5
119.5
118.8
117.7
113.0
77.5 CHLOROFORM-D
77.2 CHLOROFORM-D
77.2 CDCl3
76.8 CHLOROFORM-D
68.7
63.9
MeO
NH
O
O
34
f1 (ppm)

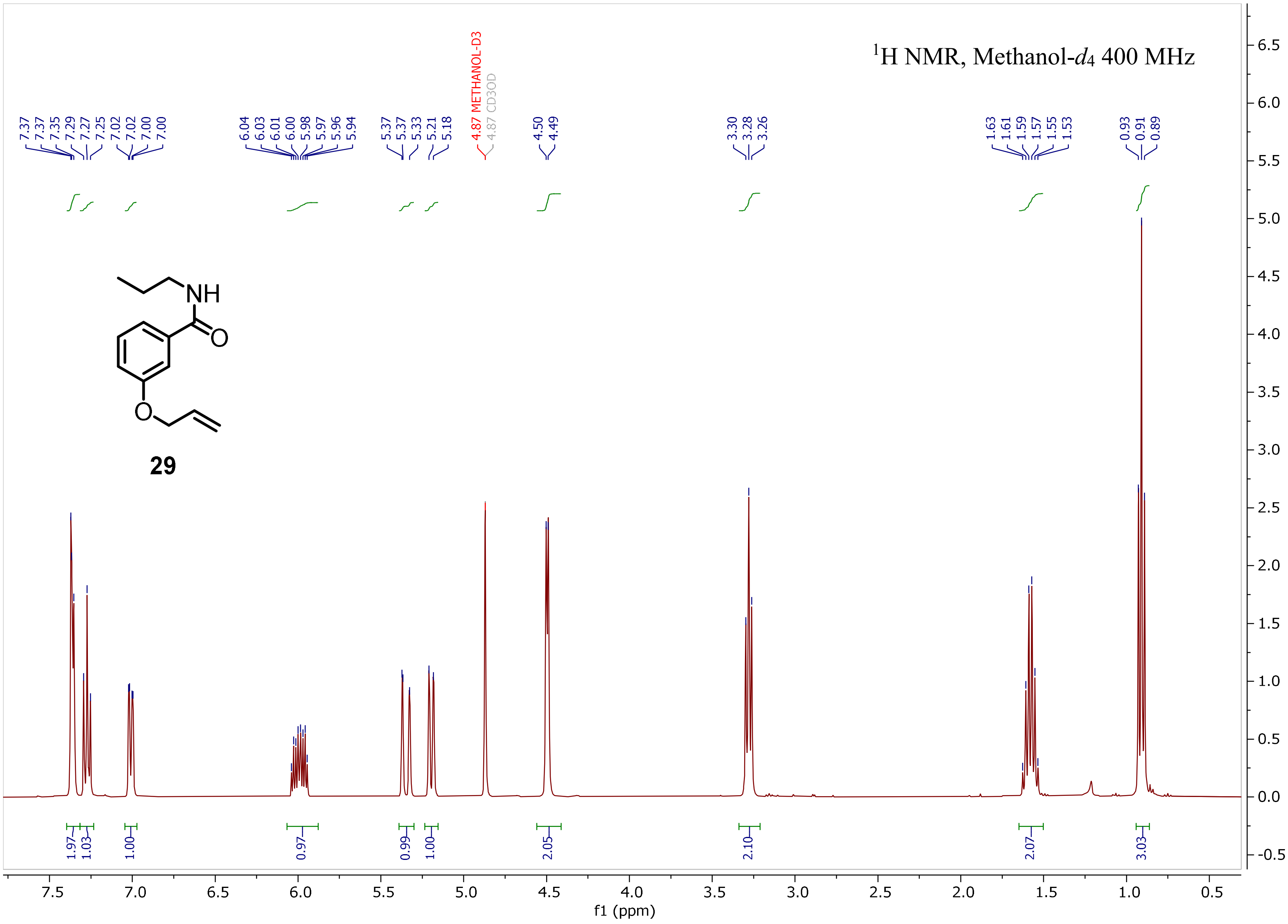
1H NMR, Methanol-d4 400 MHz
7.37
7.37
7.35
7.29
7.27
7.25
7.02
7.02
7.00
7.00
6.04
6.03
6.01
6.00
5.98
5.97
5.96
5.94
5.37
5.37
5.33
5.21
5.18
4.87 METHANOL-D3
4.87 CD3OD
4.50
4.49
3.30
3.28
3.26
1.63
1.61
1.59
1.57
1.55
1.53
0.93
0.91
0.89
NH
O
O
29
1.97
1.03
1.00
0.97
0.99
1.00
2.05
2.10
2.07
3.03
f1 (ppm)

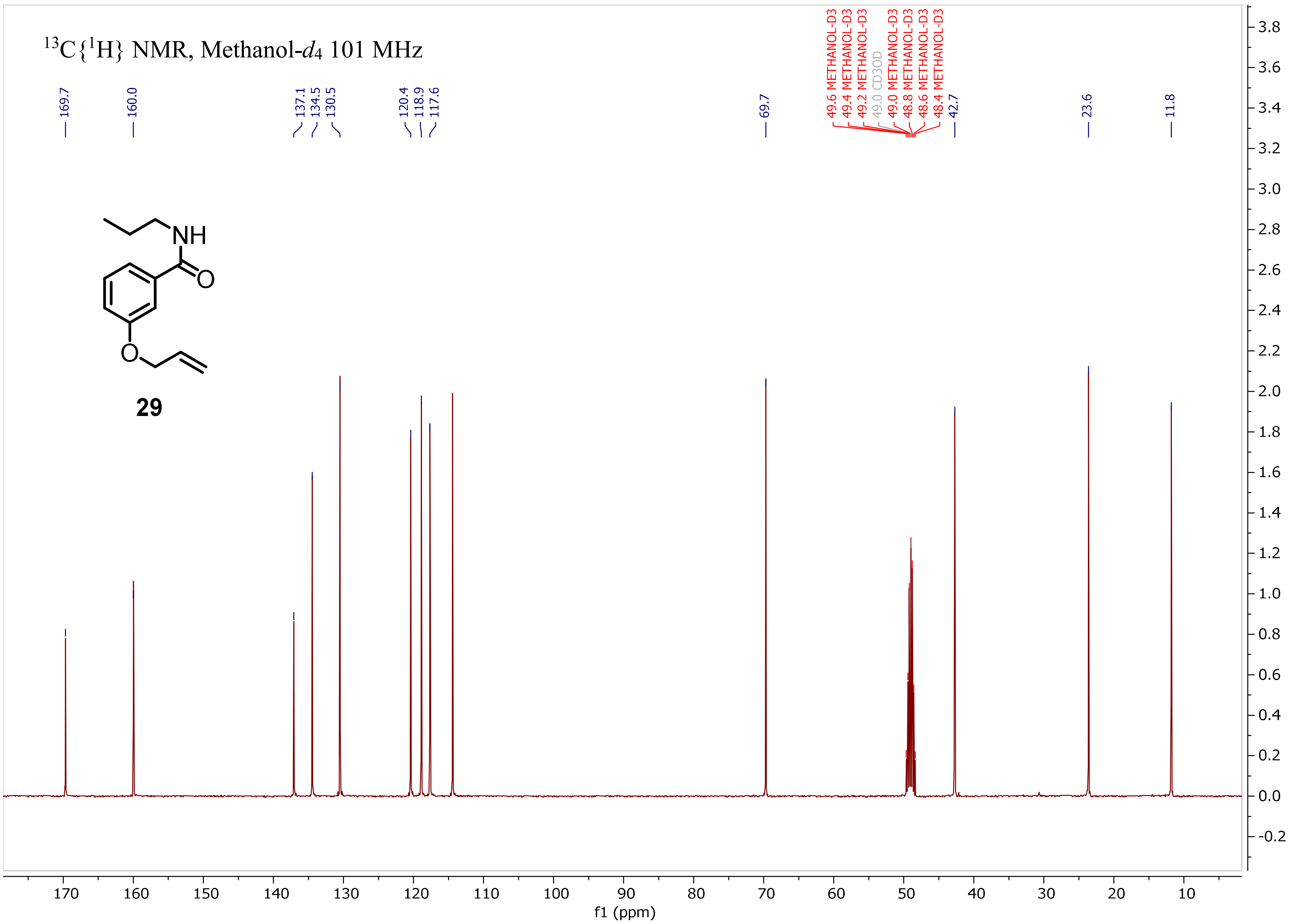

$^{13}C\{^1H\}$ NMR, Methanol-d_4 101 MHz
169.7
160.0
137.1
134.5
130.5
120.4
118.9
117.6
69.7
49.6 METHANOL-D3
49.4 METHANOL-D3
49.2 METHANOL-D3
49.0 CD3OD
49.0 METHANOL-D3
48.8 METHANOL-D3
48.6 METHANOL-D3
48.4 METHANOL-D3
42.7
23.6
11.8
NH
O
O
29
f1 (ppm)

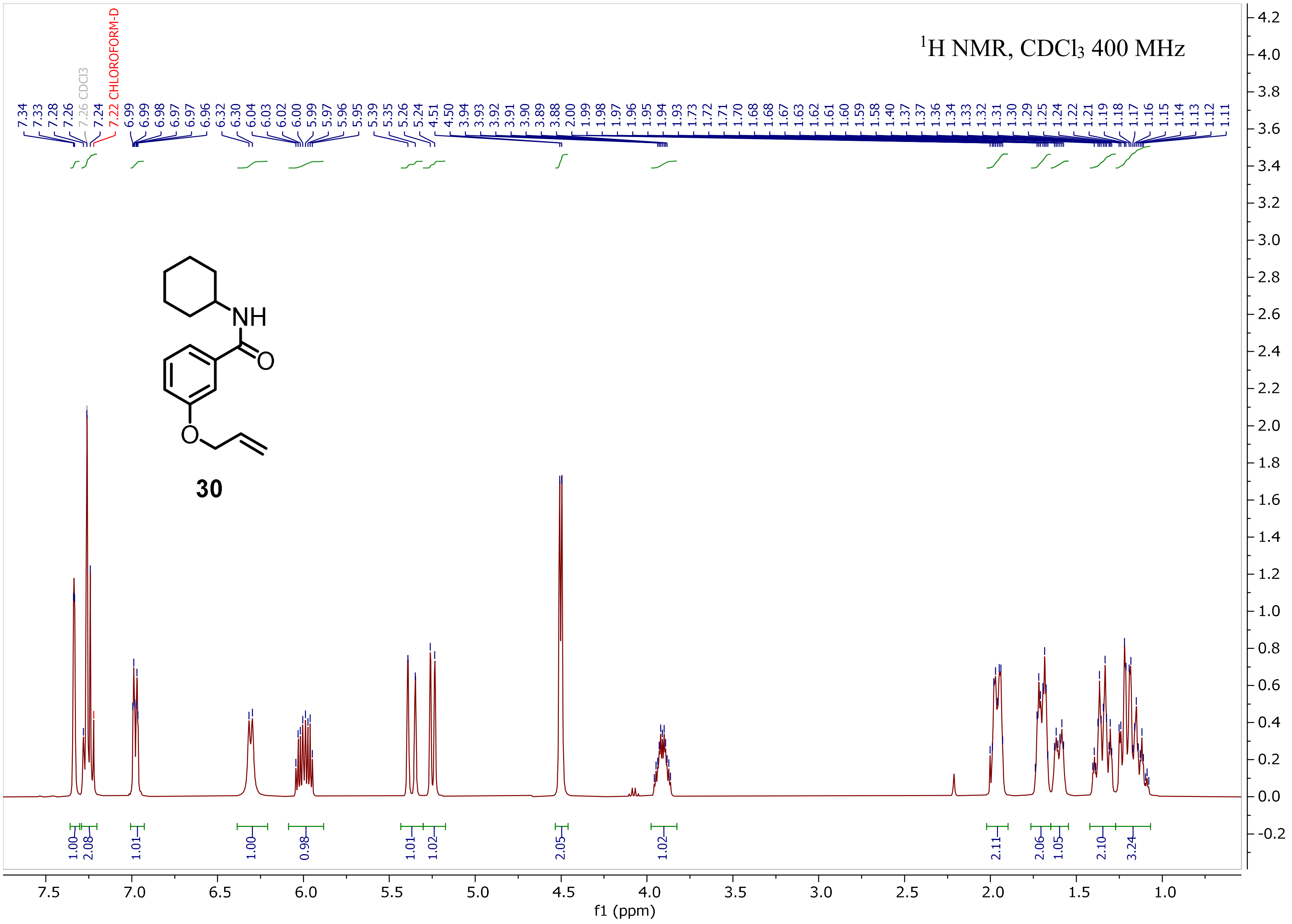

1H NMR, CDCl3 400 MHz
NH
O
O
30
f1 (ppm)

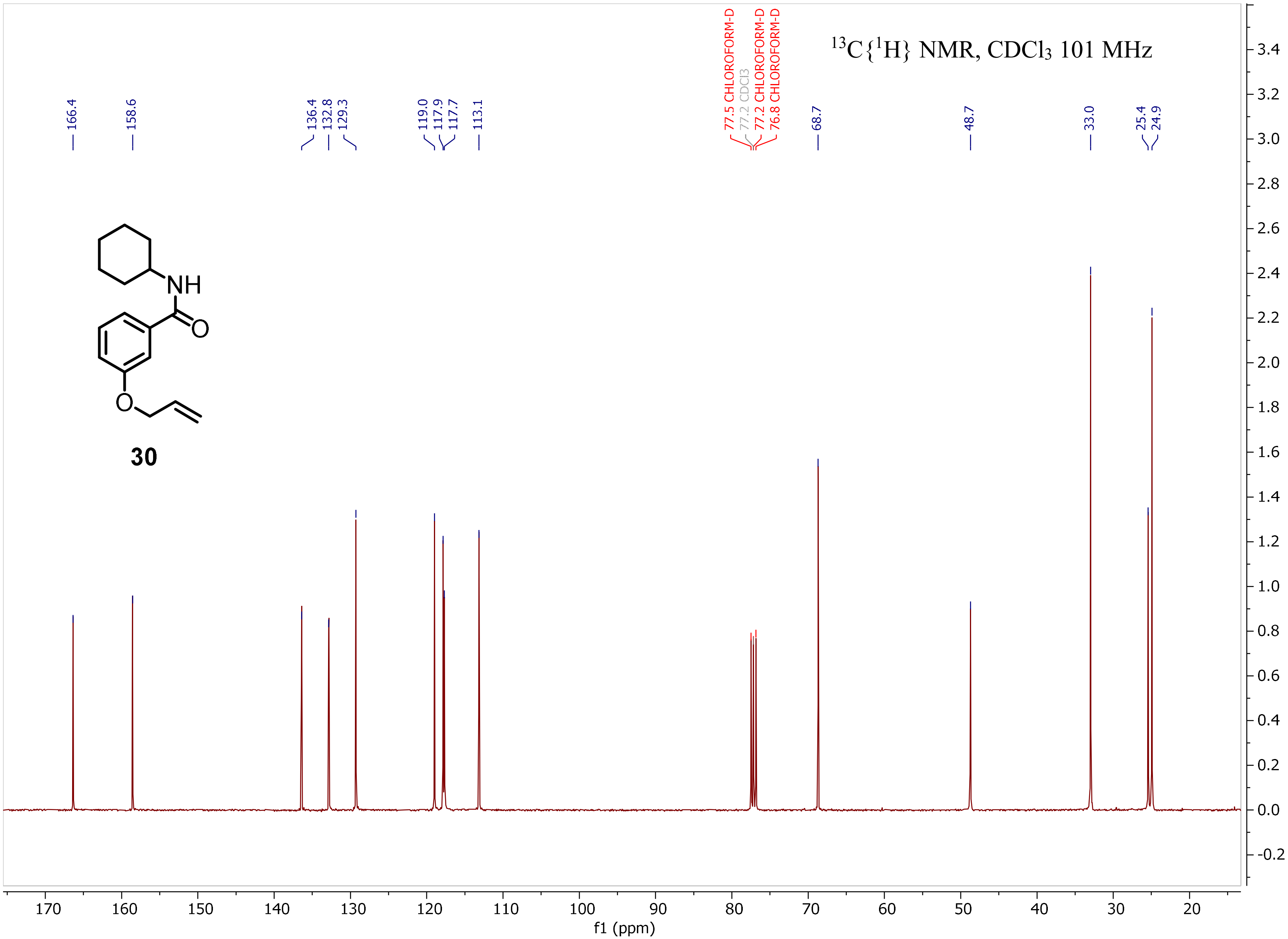

$^{13}C\{^1H\}$ NMR, $CDCl_3$ 101 MHz
166.4
158.6
136.4
132.8
129.3
119.0
117.9
117.7
113.1
77.5 CHLOROFORM-D
77.2 CDCl3
77.2 CHLOROFORM-D
76.8 CHLOROFORM-D
68.7
48.7
33.0
25.4
24.9
NH
O
O
30
f1 (ppm)

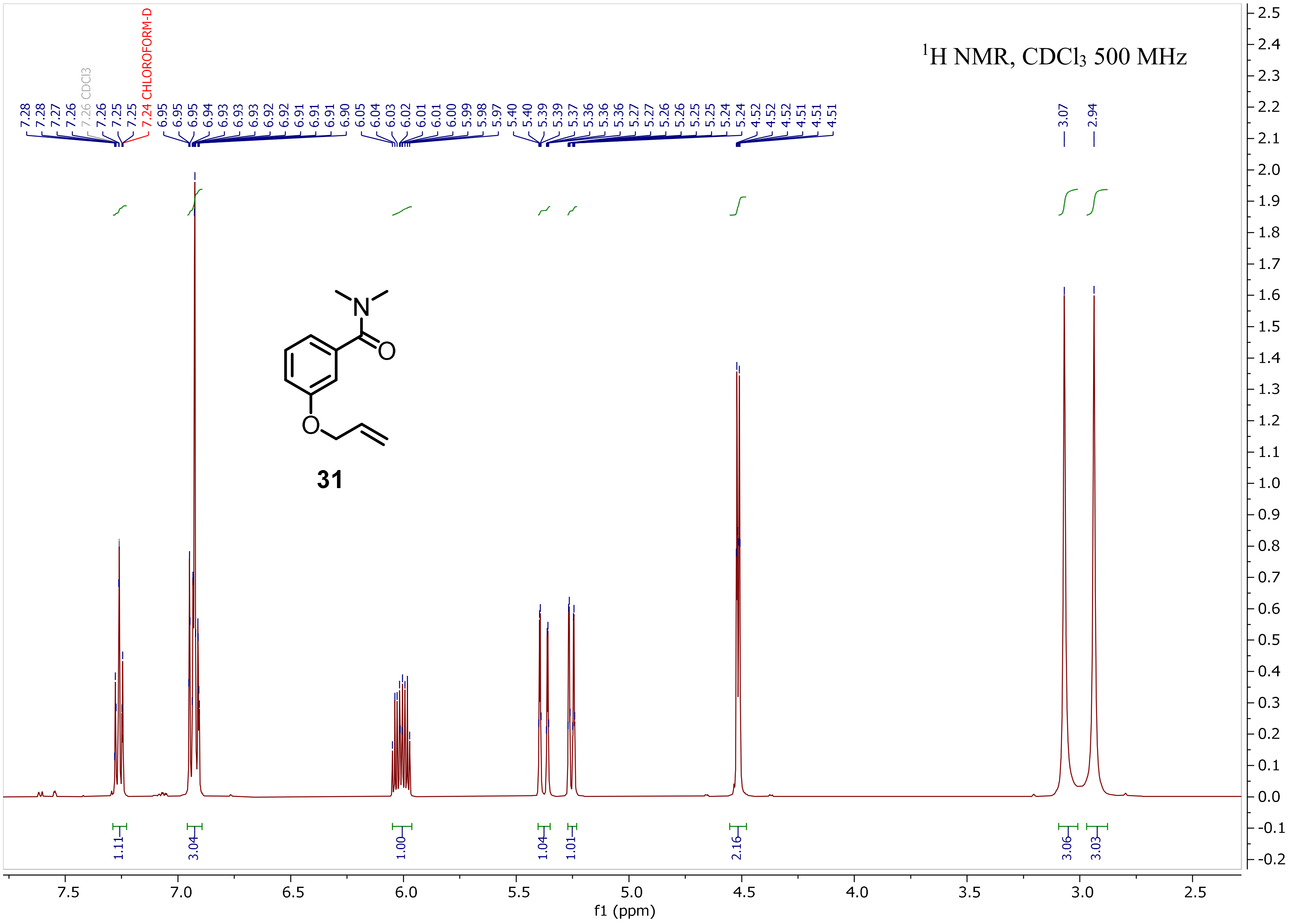

¹H NMR, CDCl3 500 MHz
31
f1 (ppm)

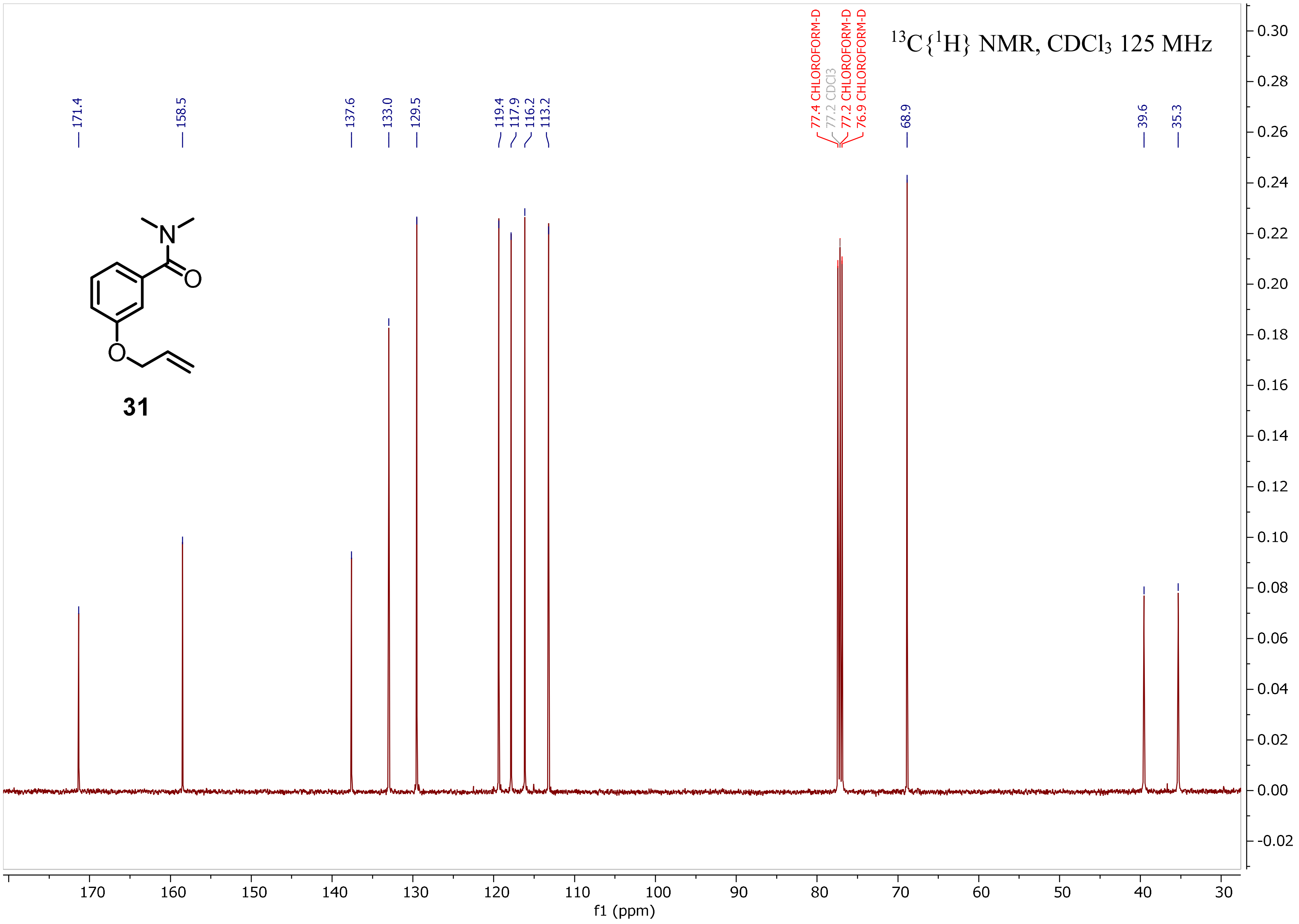

$^{13}C\{^1H\}$ NMR, $CDCl_3$ 125 MHz
171.4
158.5
137.6
133.0
129.5
119.4
117.9
116.2
113.2
77.4 CHLOROFORM-D
77.2 CDCl3
77.2 CHLOROFORM-D
76.9 CHLOROFORM-D
68.9
39.6
35.3
N
O
O
31
0.30
0.28
0.26
0.24
0.22
0.20
0.18
0.16
0.14
0.12
0.10
0.08
0.06
0.04
0.02
0.00
-0.02
170
160
150
140
130
120
110
100
90
80
70
60
50
40
30
f1 (ppm)

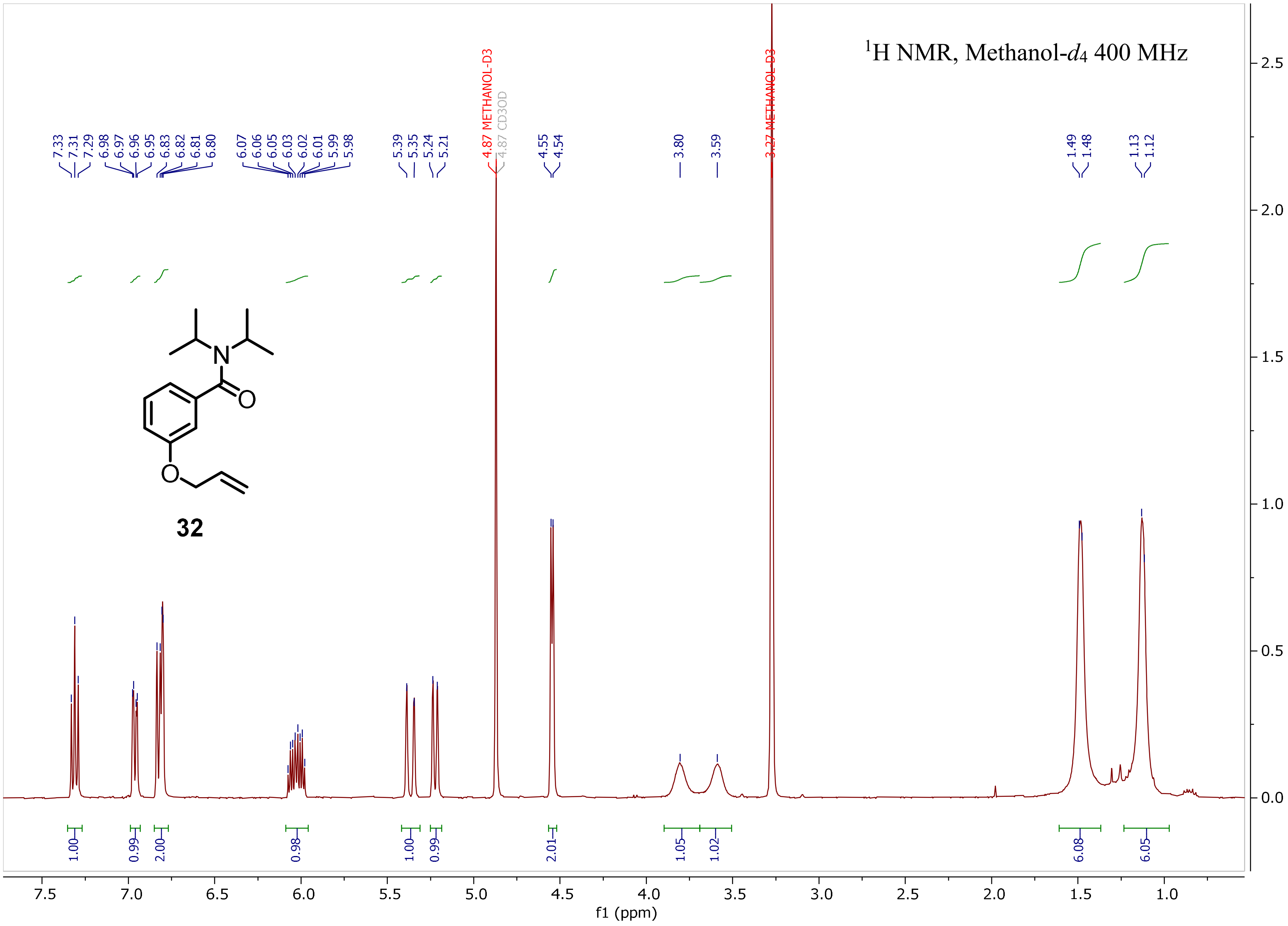

1H NMR, Methanol-d4 400 MHz
32
f1 (ppm)

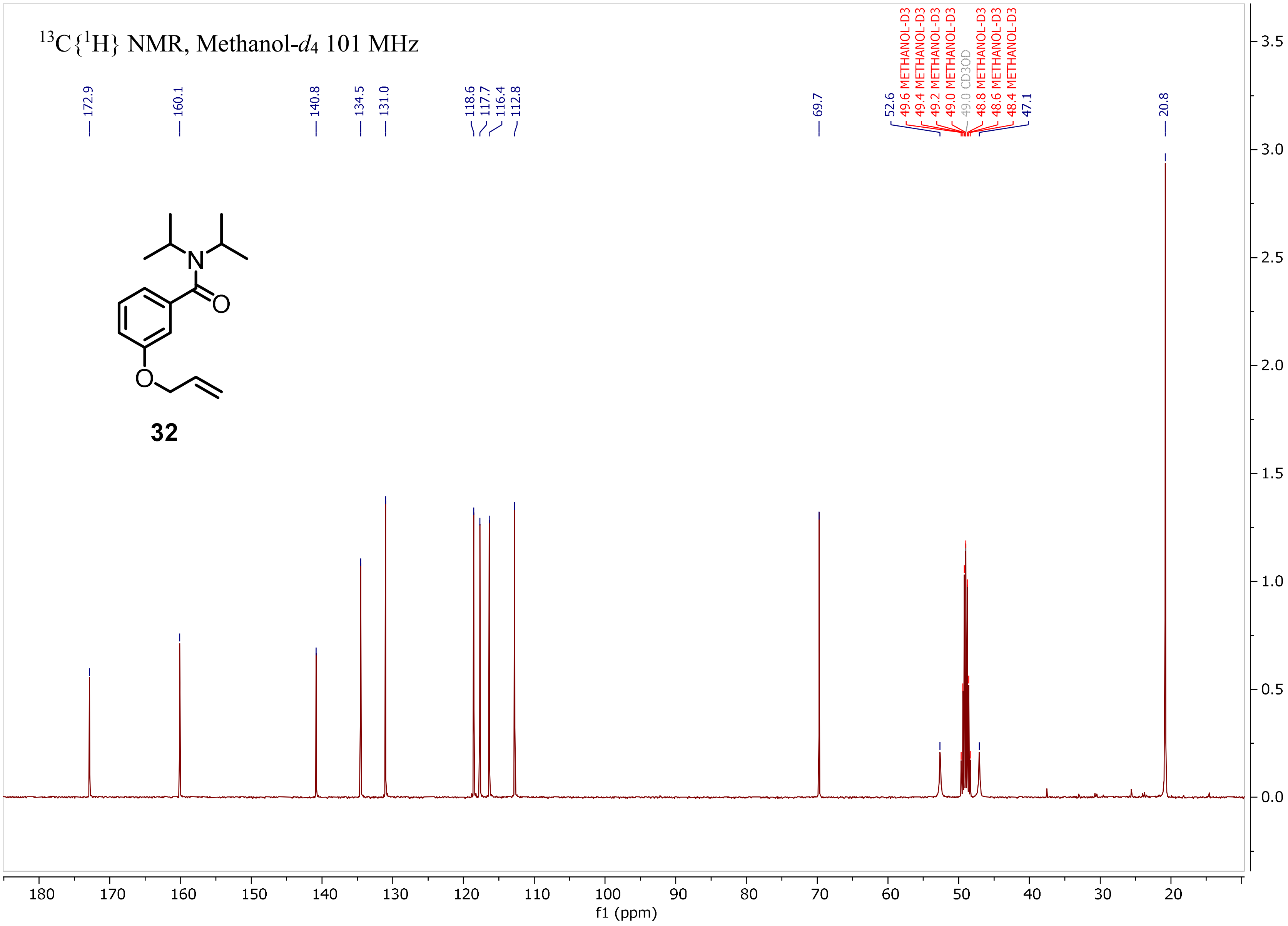

$^{13}C\{^1H\}$ NMR, Methanol-d_4 101 MHz
172.9
160.1
140.8
134.5
131.0
118.6
117.7
116.4
112.8
69.7
52.6
49.6 METHANOL-D3
49.4 METHANOL-D3
49.2 METHANOL-D3
49.0 METHANOL-D3
49.0 CD3OD
48.8 METHANOL-D3
48.6 METHANOL-D3
48.4 METHANOL-D3
47.1
20.8
N
O
O
32
f1 (ppm)

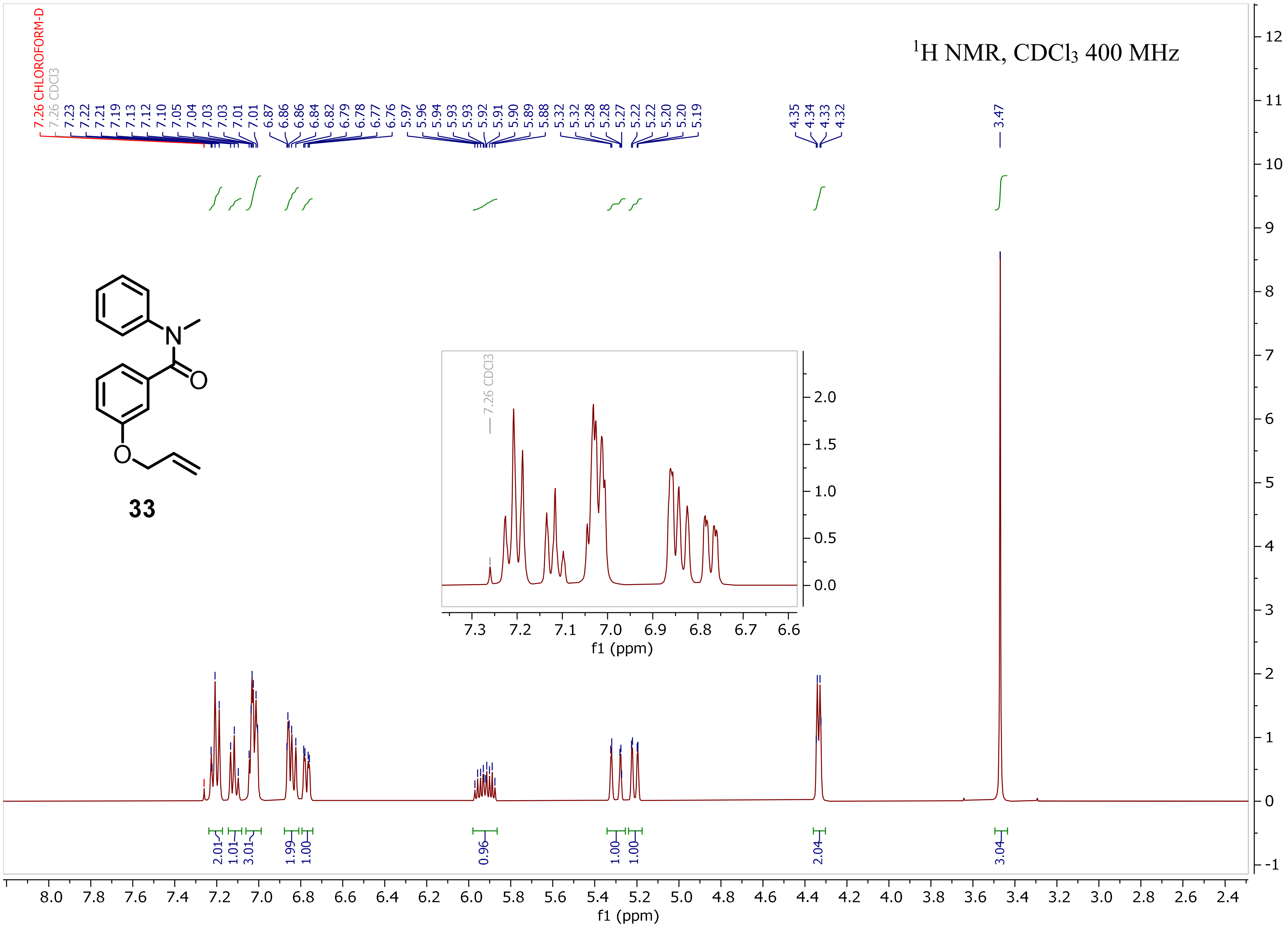

^{1}H NMR, CDCl$_3$ 400 MHz
33
f1 (ppm)

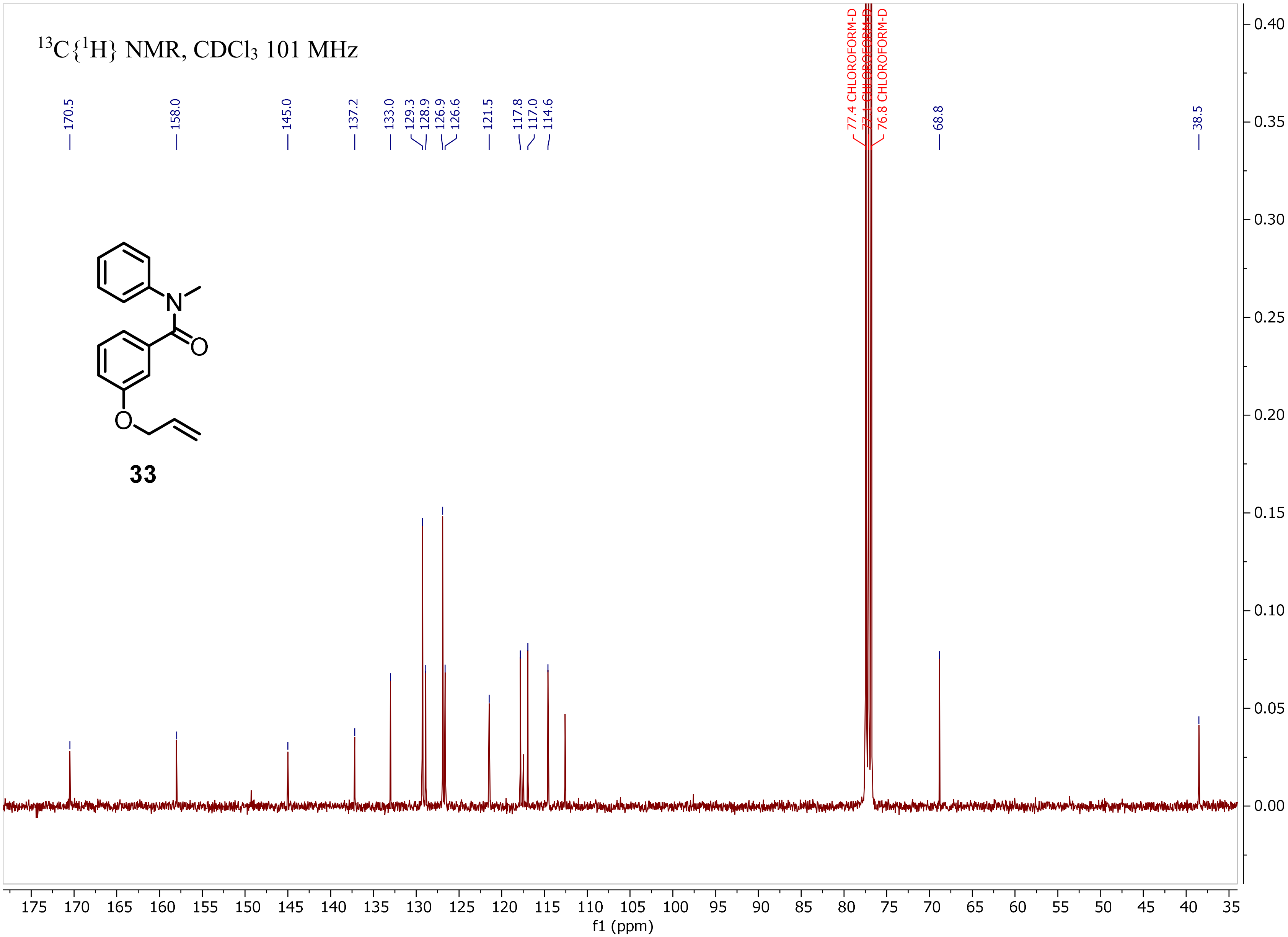

$^{13}C\{^1H\}$ NMR, $CDCl_3$ 101 MHz
170.5
158.0
145.0
137.2
133.0
129.3
128.9
126.9
126.6
121.5
117.8
117.0
114.6
77.4 CHLOROFORM-D
77.1 CHLOROFORM-D
76.8 CHLOROFORM-D
68.8
38.5
33
f1 (ppm)

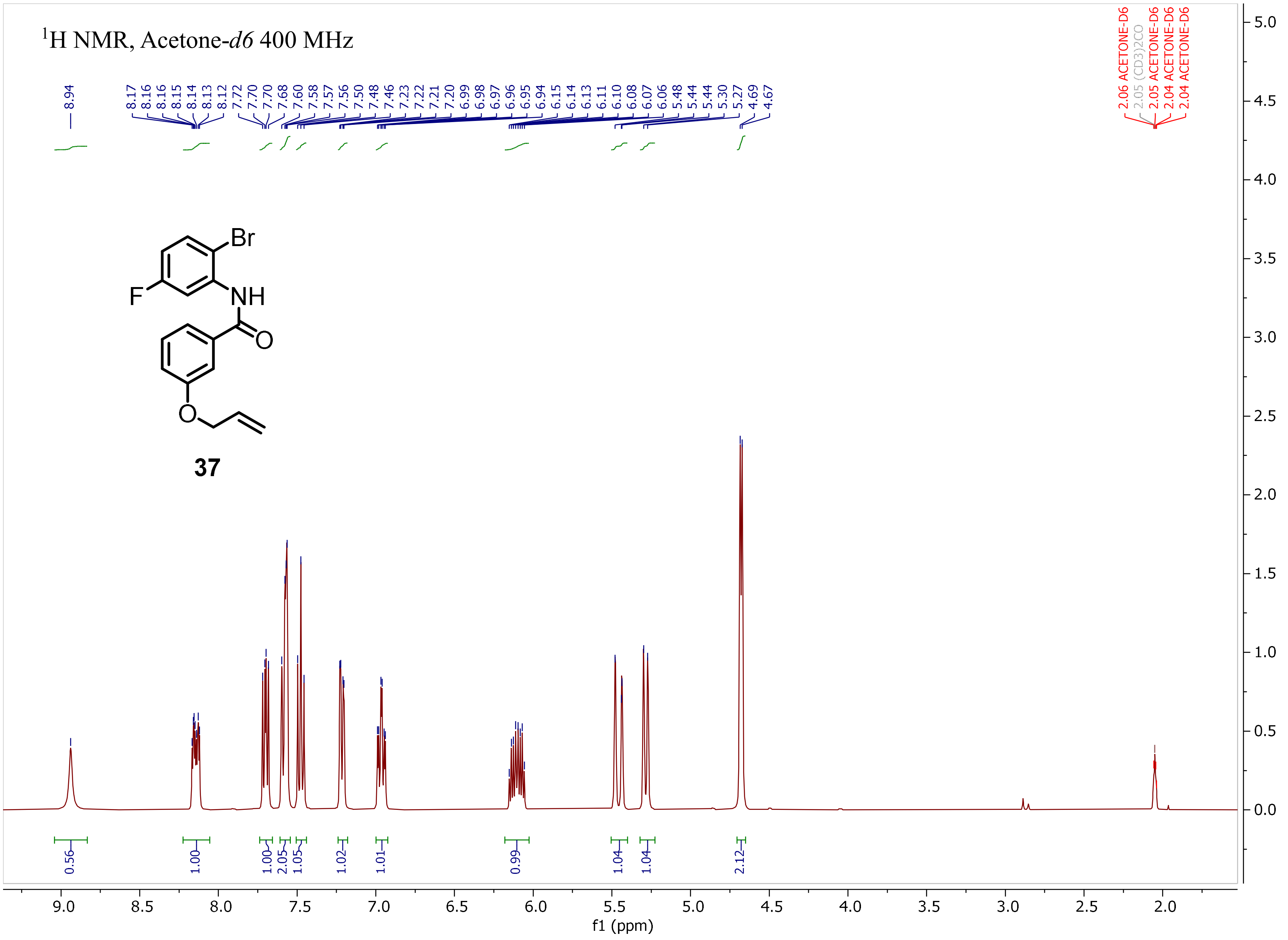

¹H NMR, Acetone-d6 400 MHz
37
f1 (ppm)

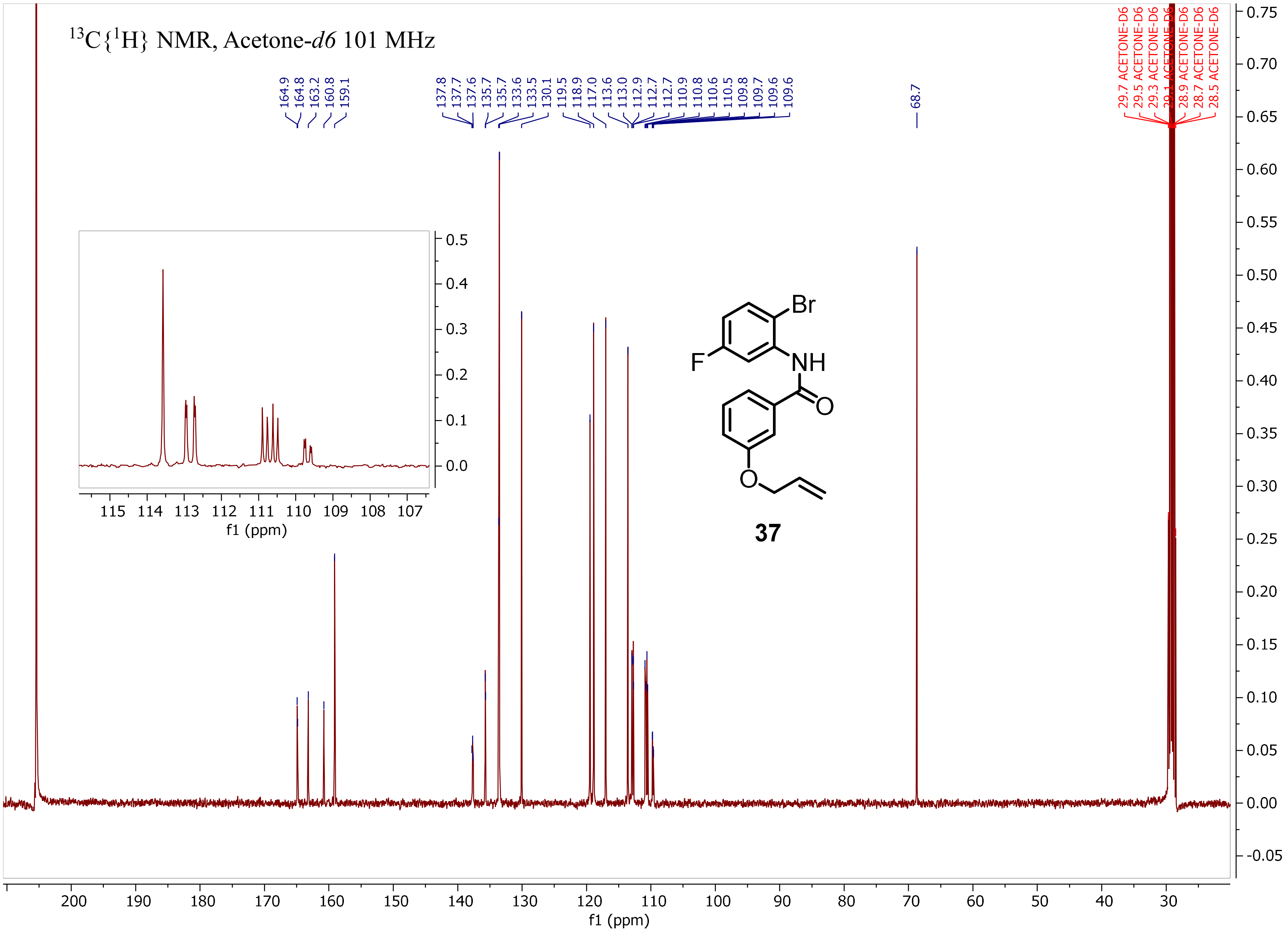

13C{1H} NMR, Acetone-d6 101 MHz
164.9
164.8
163.2
160.8
159.1
137.8
137.7
137.6
135.7
135.7
133.6
133.5
130.1
119.5
118.9
117.0
113.6
113.0
112.9
112.7
112.7
110.9
110.8
110.6
110.5
109.8
109.7
109.6
109.6
68.7
29.7 ACETONE-D6
29.5 ACETONE-D6
29.3 ACETONE-D6
29.1 ACETONE-D6
28.9 ACETONE-D6
28.7 ACETONE-D6
28.5 ACETONE-D6
Br
F
NH
O
O
37
f1 (ppm)

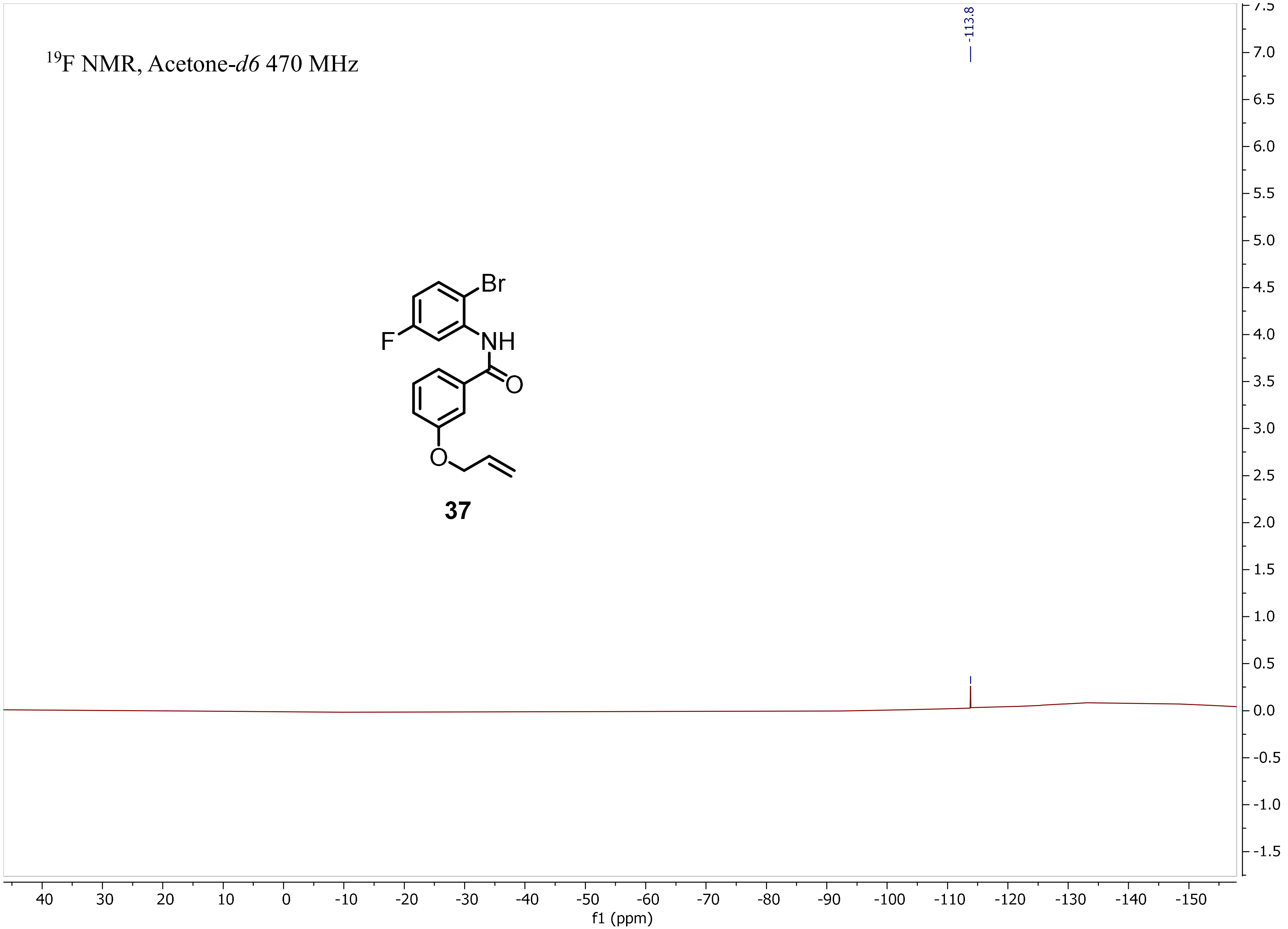
^{19}F NMR, Acetone-*d6* 470 MHz
-113.8
Br
F
NH
O
O
37
f1 (ppm)

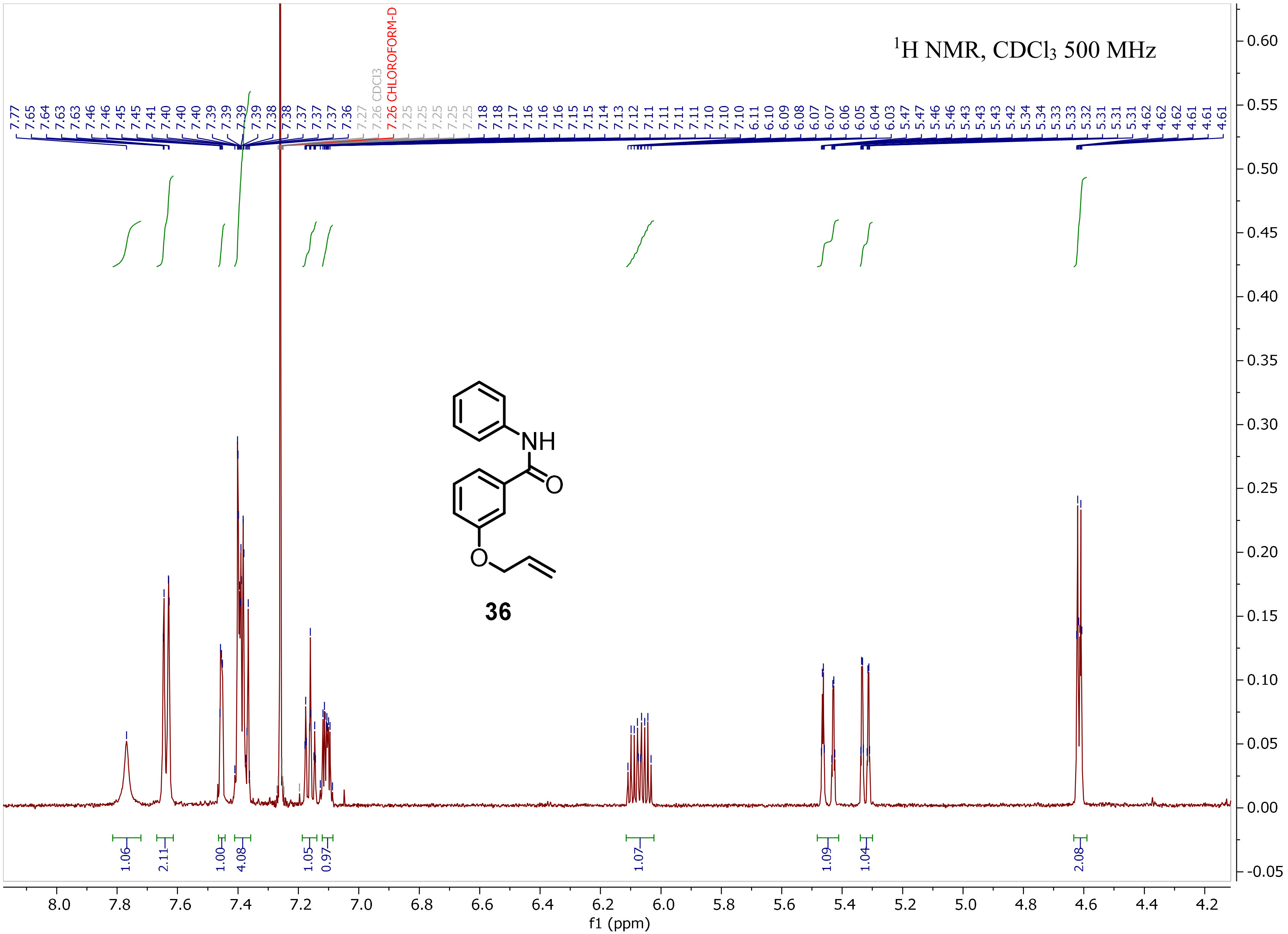

1H NMR, CDCl3 500 MHz
36
NH
O
O
f1 (ppm)

$^{13}C\{^{1}H\}$ NMR, $CDCl_3$ 101 MHz

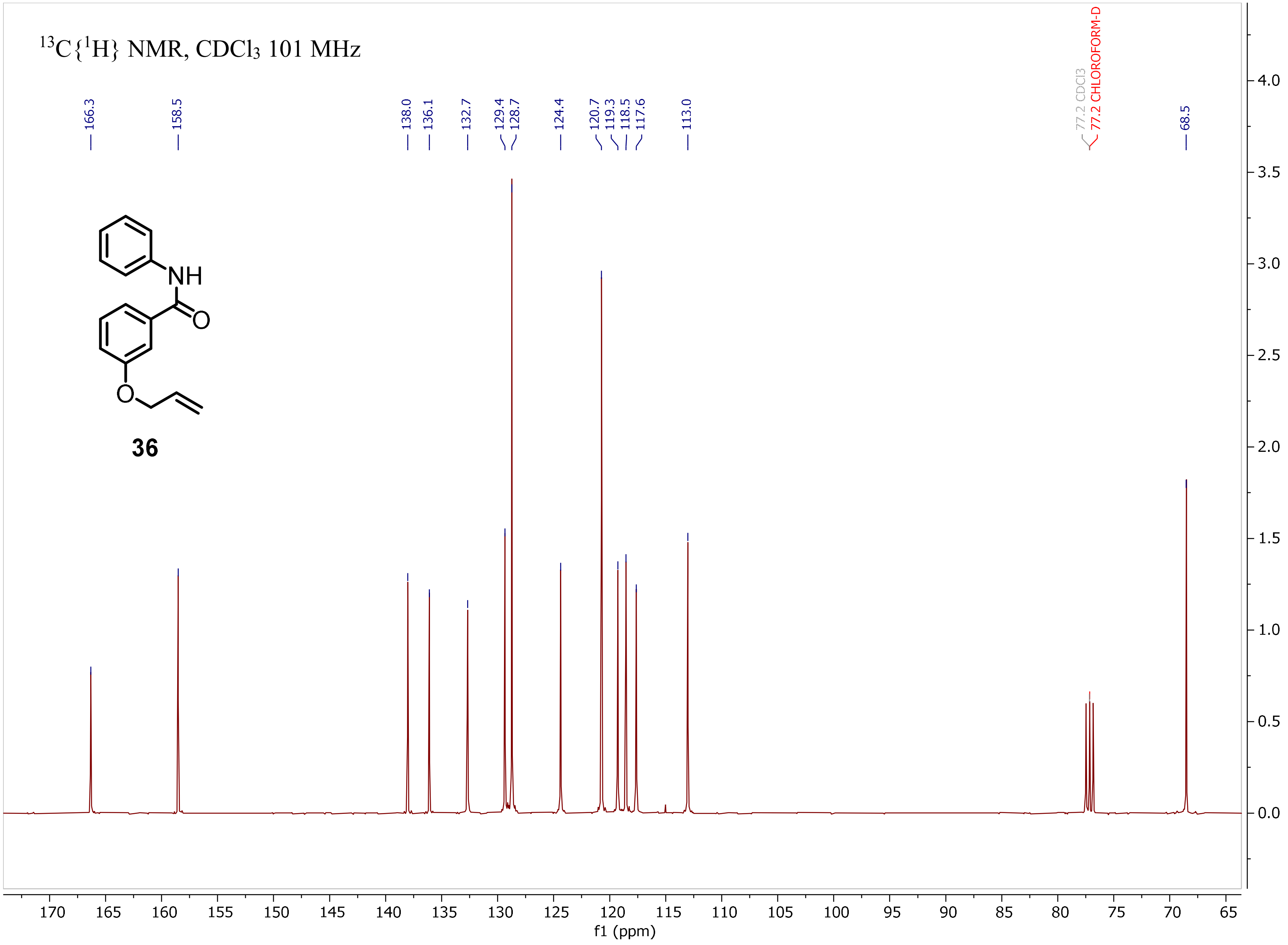

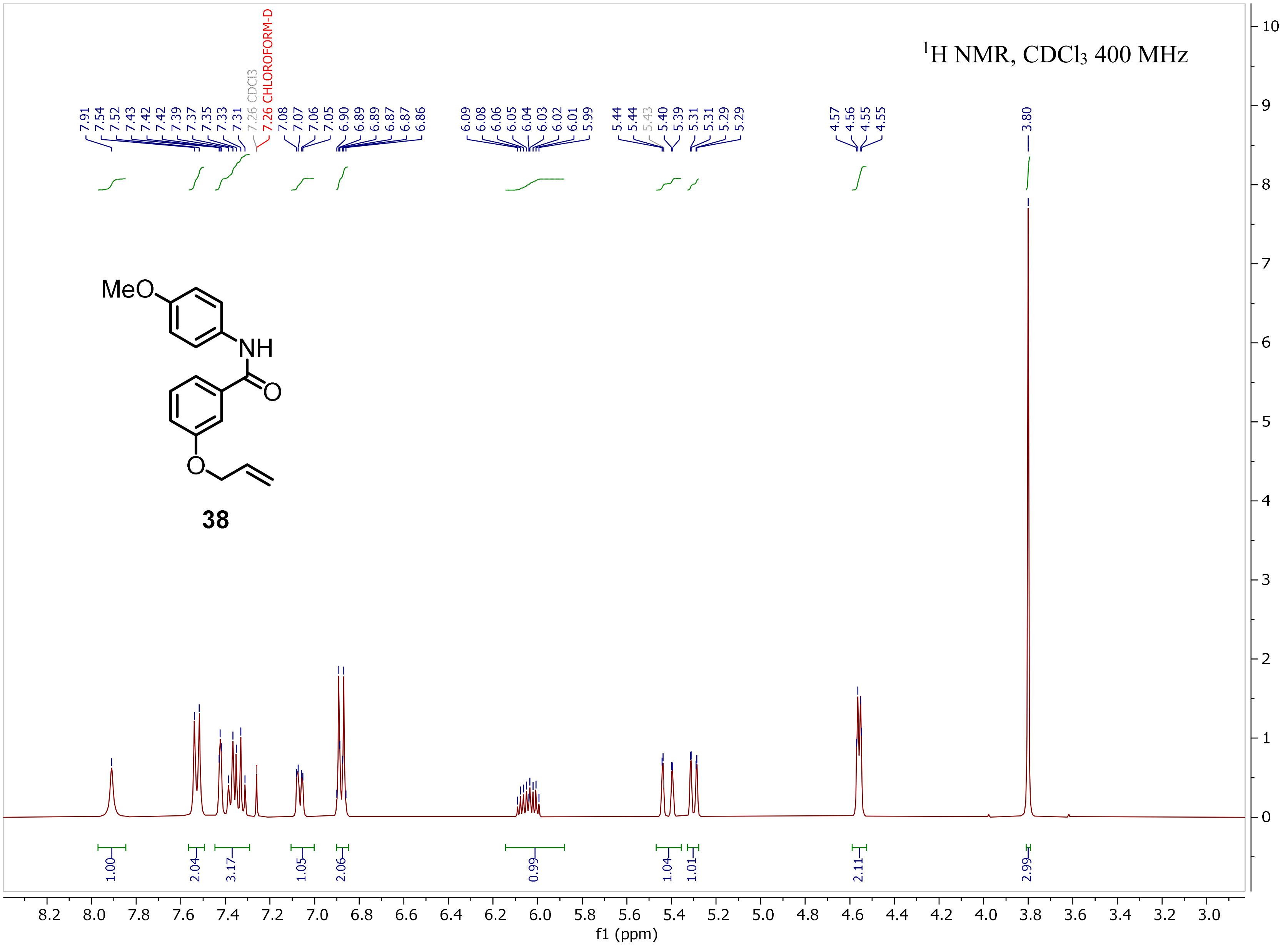

^{1}H NMR, $CDCl_3$ 400 MHz
MeO
NH
O
O
38
f1 (ppm)

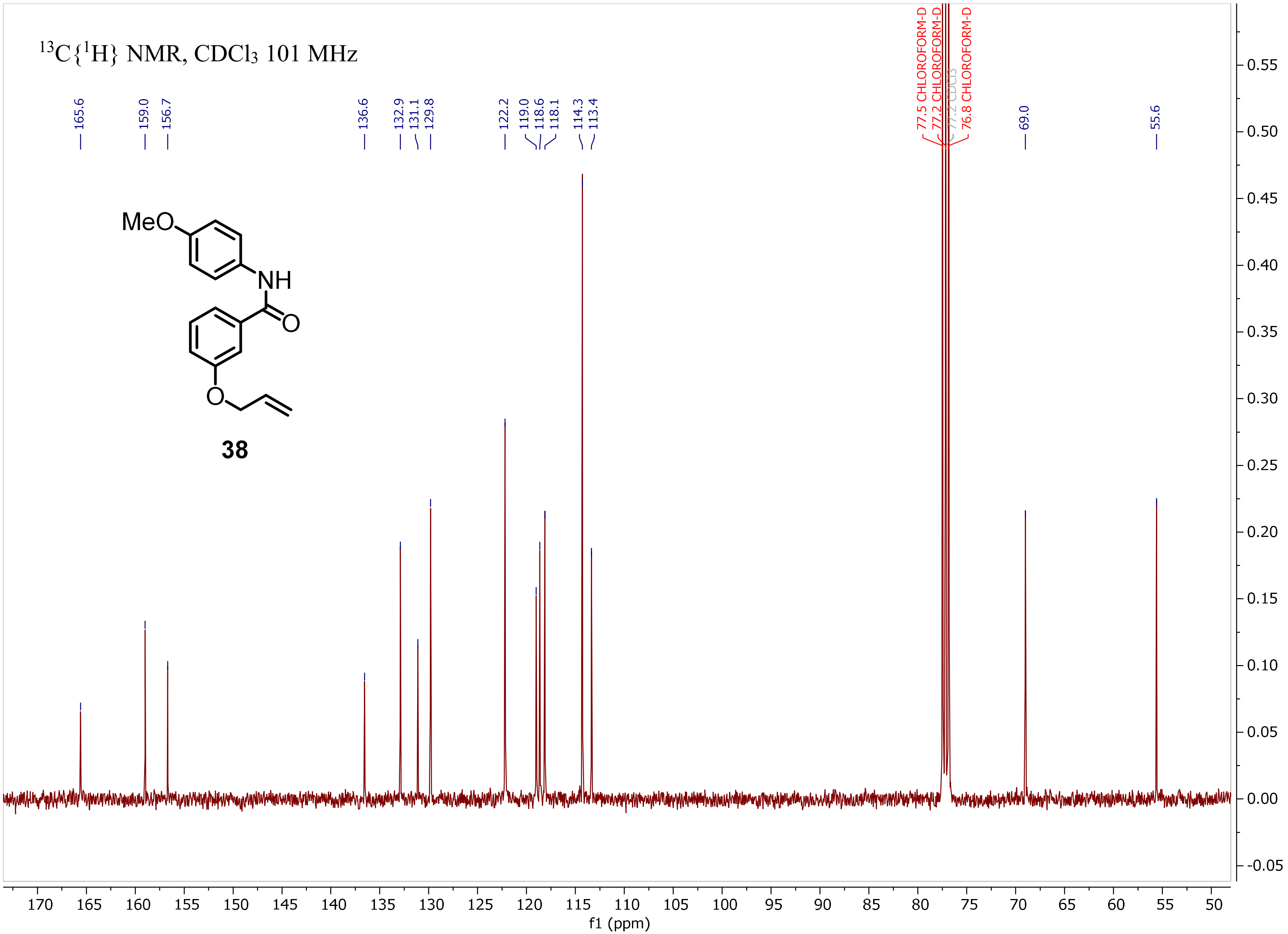

$^{13}C\{^1H\}$ NMR, CDCl3 101 MHz
165.6
159.0
156.7
136.6
132.9
131.1
129.8
122.2
119.0
118.6
118.1
114.3
113.4
77.5 CHLOROFORM-D
77.2 CHLOROFORM-D
77.2 CDCl3
76.8 CHLOROFORM-D
69.0
55.6
MeO
NH
O
O
38
f1 (ppm)

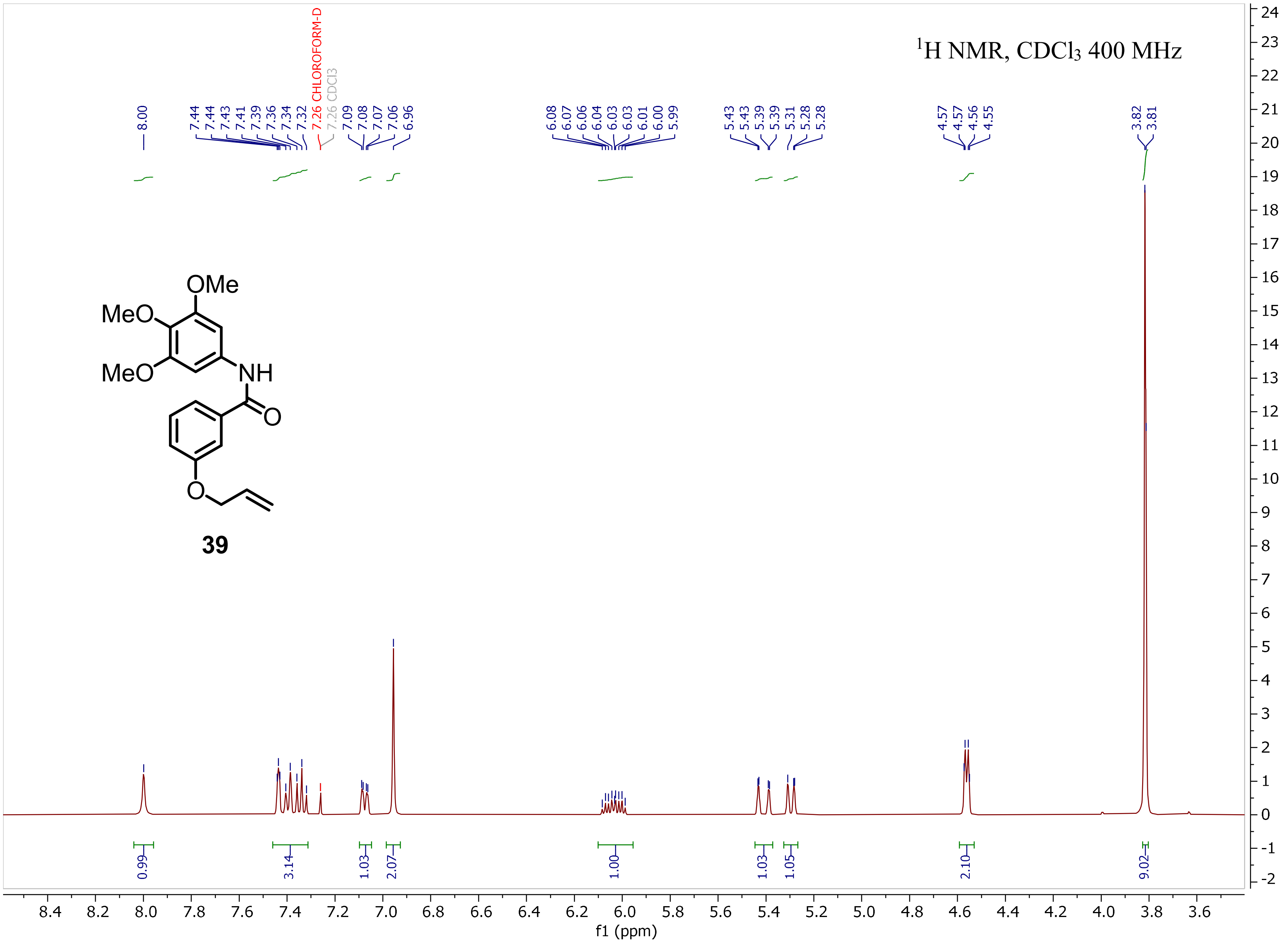

^{1}H NMR, CDCl$_3$ 400 MHz
39
f1 (ppm)

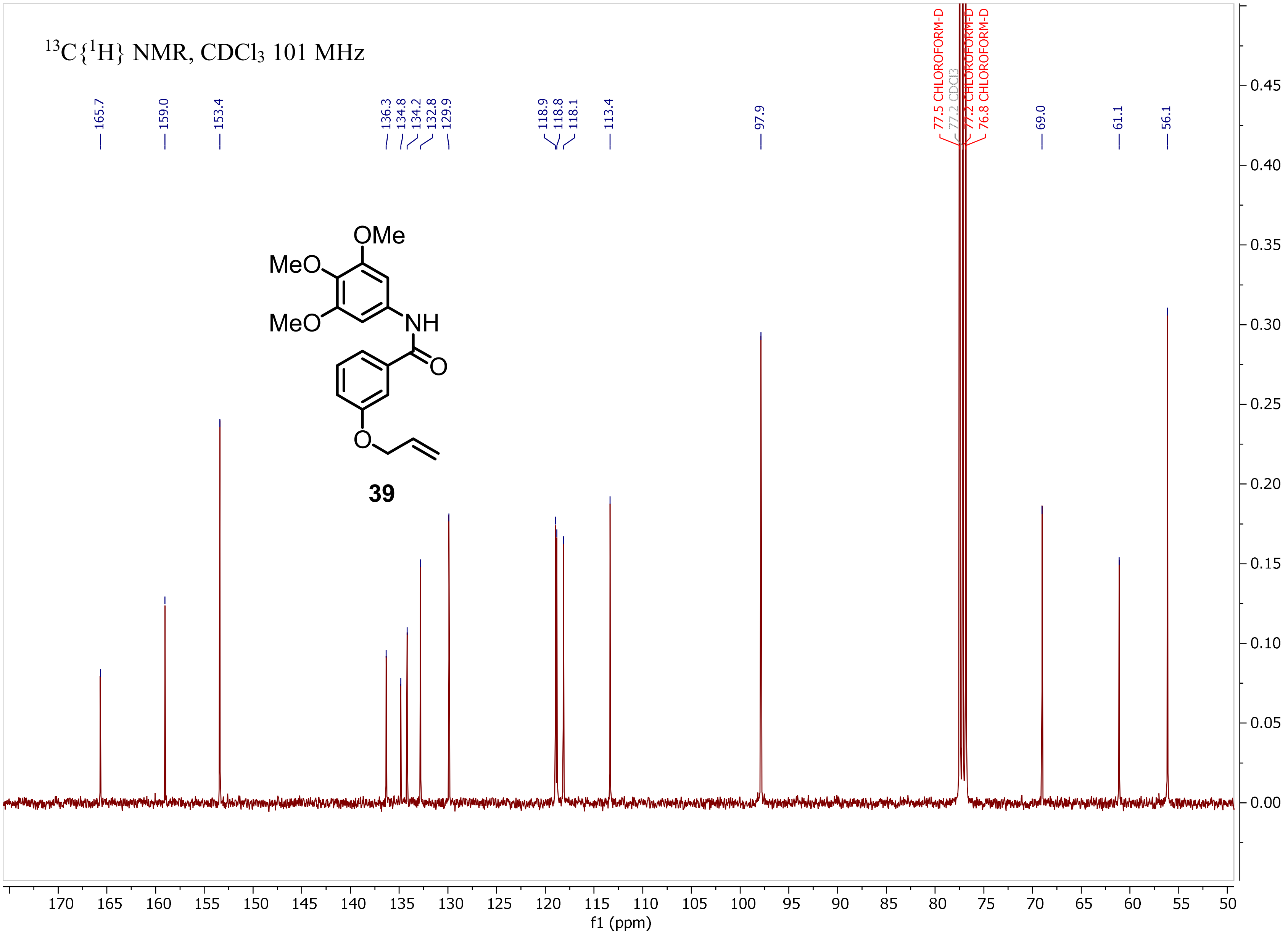

$^{13}C\{^1H\}$ NMR, $CDCl_3$ 101 MHz
165.7
159.0
153.4
136.3
134.8
134.2
132.8
129.9
118.9
118.8
118.1
113.4
97.9
77.5 CHLOROFORM-D
77.2 CDCl3
77.2 CHLOROFORM-D
76.8 CHLOROFORM-D
69.0
61.1
56.1
OMe
MeO
MeO
NH
O
O
39
f1 (ppm)

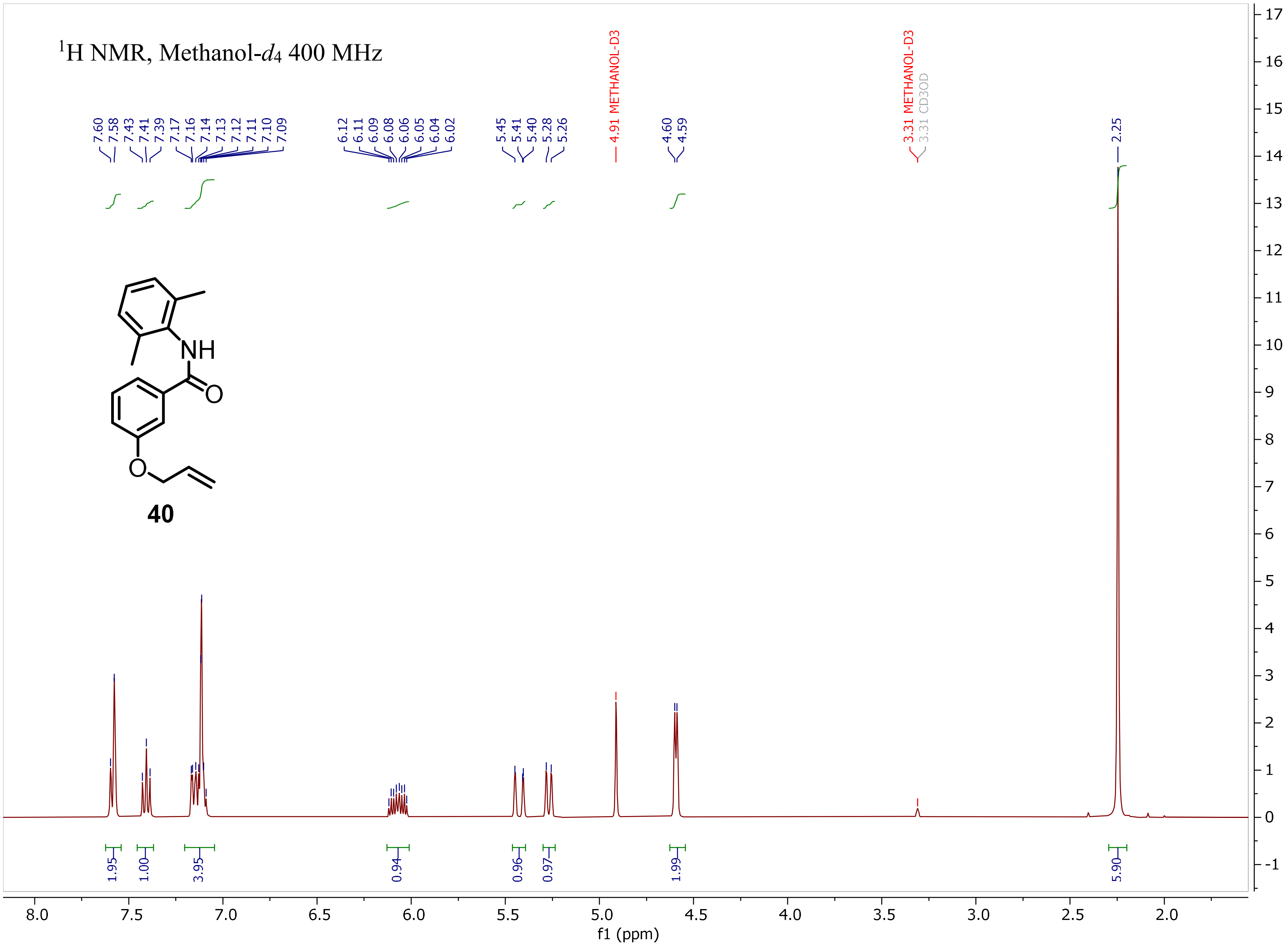

^{1}H NMR, Methanol-d_4 400 MHz
7.60 7.58 7.43 7.41 7.39 7.17 7.16 7.14 7.13 7.12 7.11 7.10 7.09
6.12 6.11 6.09 6.08 6.06 6.05 6.04 6.02
5.45 5.41 5.40 5.28 5.26
4.91 METHANOL-D3
4.60 4.59
3.31 METHANOL-D3
3.31 CD3OD
2.25
NH
O
O
40
1.95 1.00 3.95 0.94 0.96 0.97 1.99 5.90
f1 (ppm)

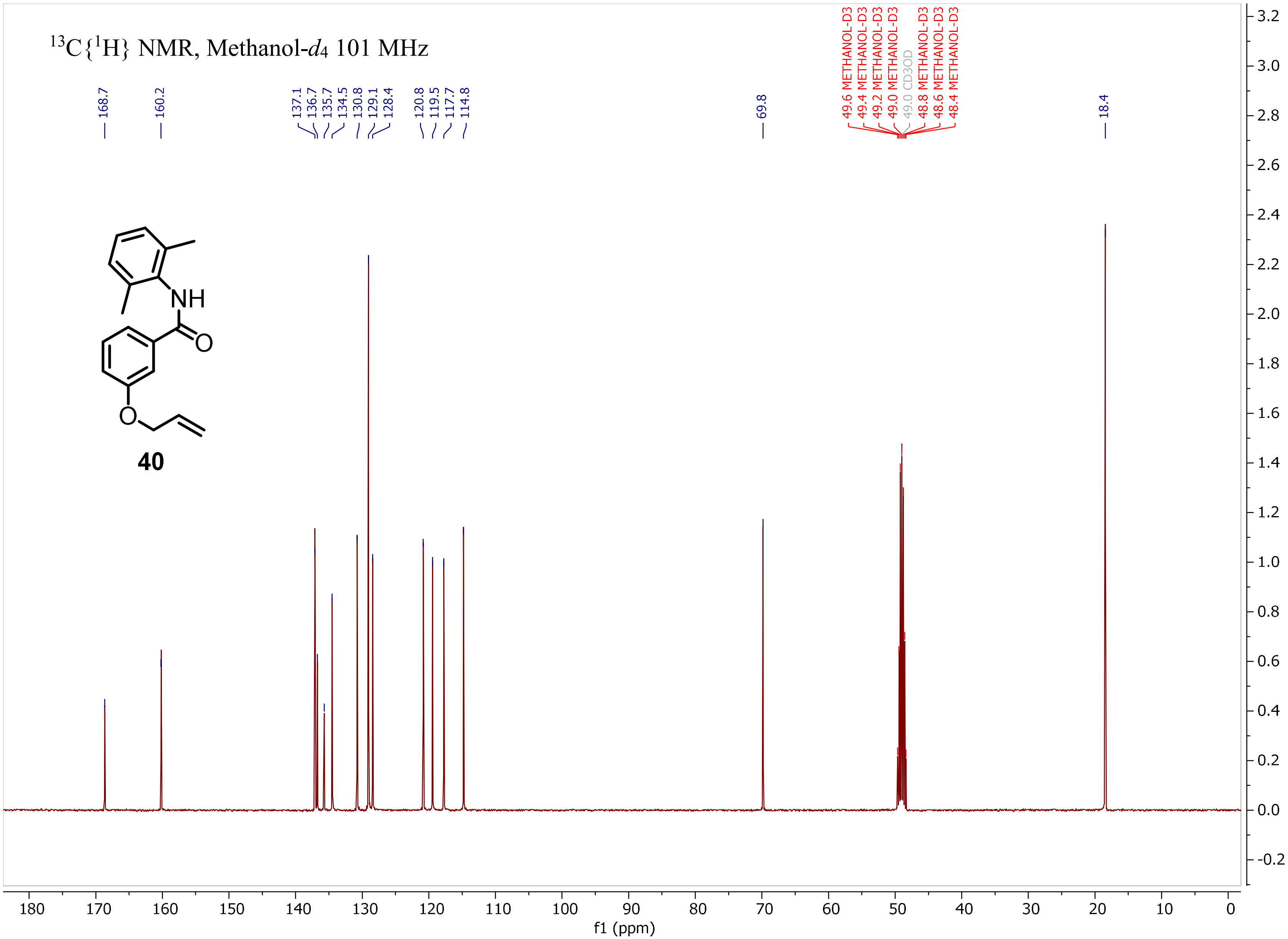

$^{13}C\{^1H\}$ NMR, Methanol-d_4 101 MHz
168.7
160.2
137.1
136.7
135.7
134.5
130.8
129.1
128.4
120.8
119.5
117.7
114.8
69.8
49.6 METHANOL-D3
49.4 METHANOL-D3
49.2 METHANOL-D3
49.0 METHANOL-D3
49.0 CD3OD
48.8 METHANOL-D3
48.6 METHANOL-D3
48.4 METHANOL-D3
18.4
NH
O
O
40
f1 (ppm)

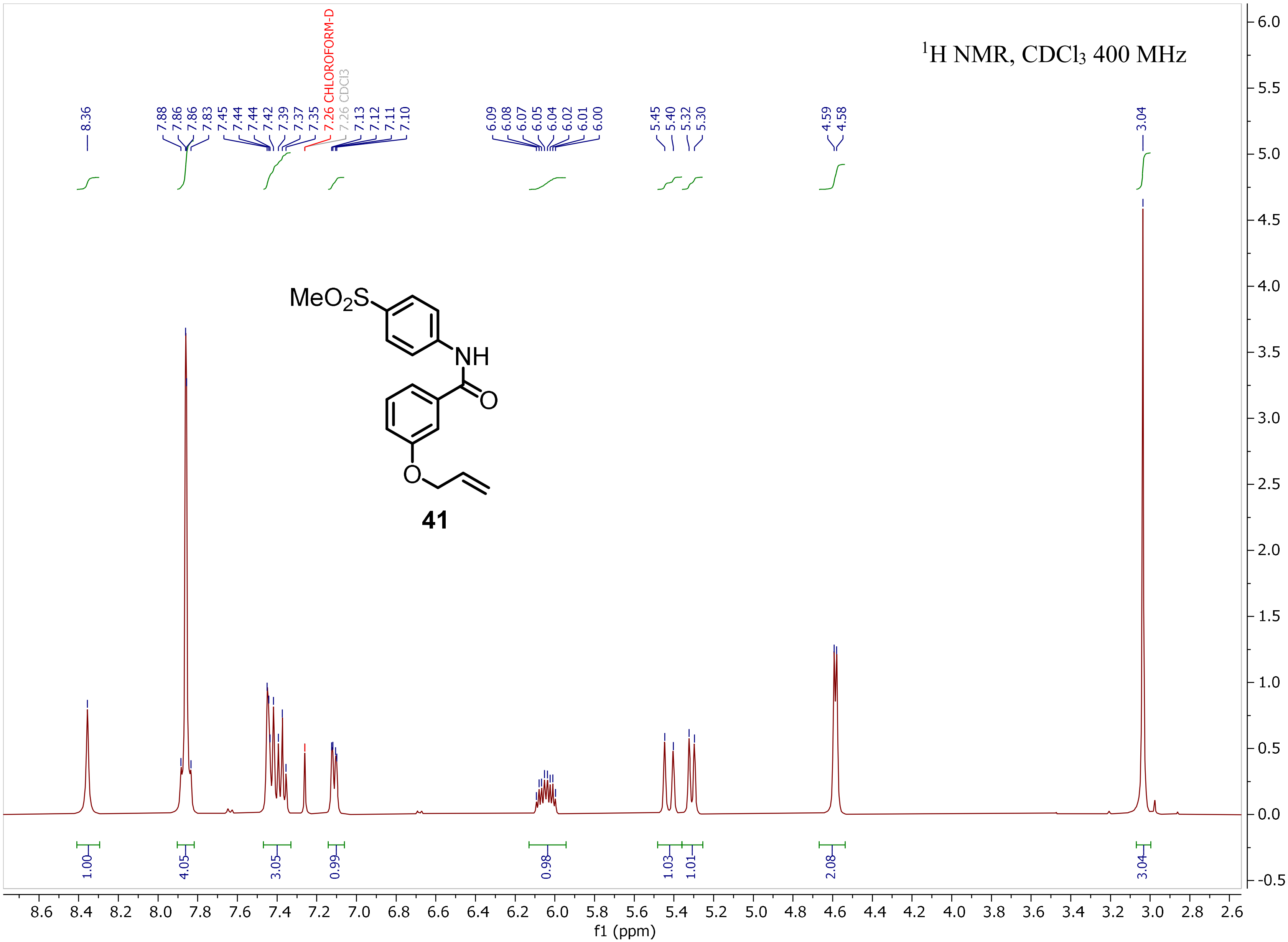

^{1}H NMR, CDCl$_3$ 400 MHz
MeO$_2$S
NH
O
O
41
8.36
7.88
7.86
7.86
7.83
7.45
7.44
7.44
7.42
7.39
7.37
7.35
7.26 CHLOROFORM-D
7.26 CDCl3
7.13
7.12
7.11
7.10
6.09
6.08
6.07
6.05
6.04
6.02
6.01
6.00
5.45
5.40
5.32
5.30
4.59
4.58
3.04
1.00
4.05
3.05
0.99
0.98
1.03
1.01
2.08
3.04
f1 (ppm)

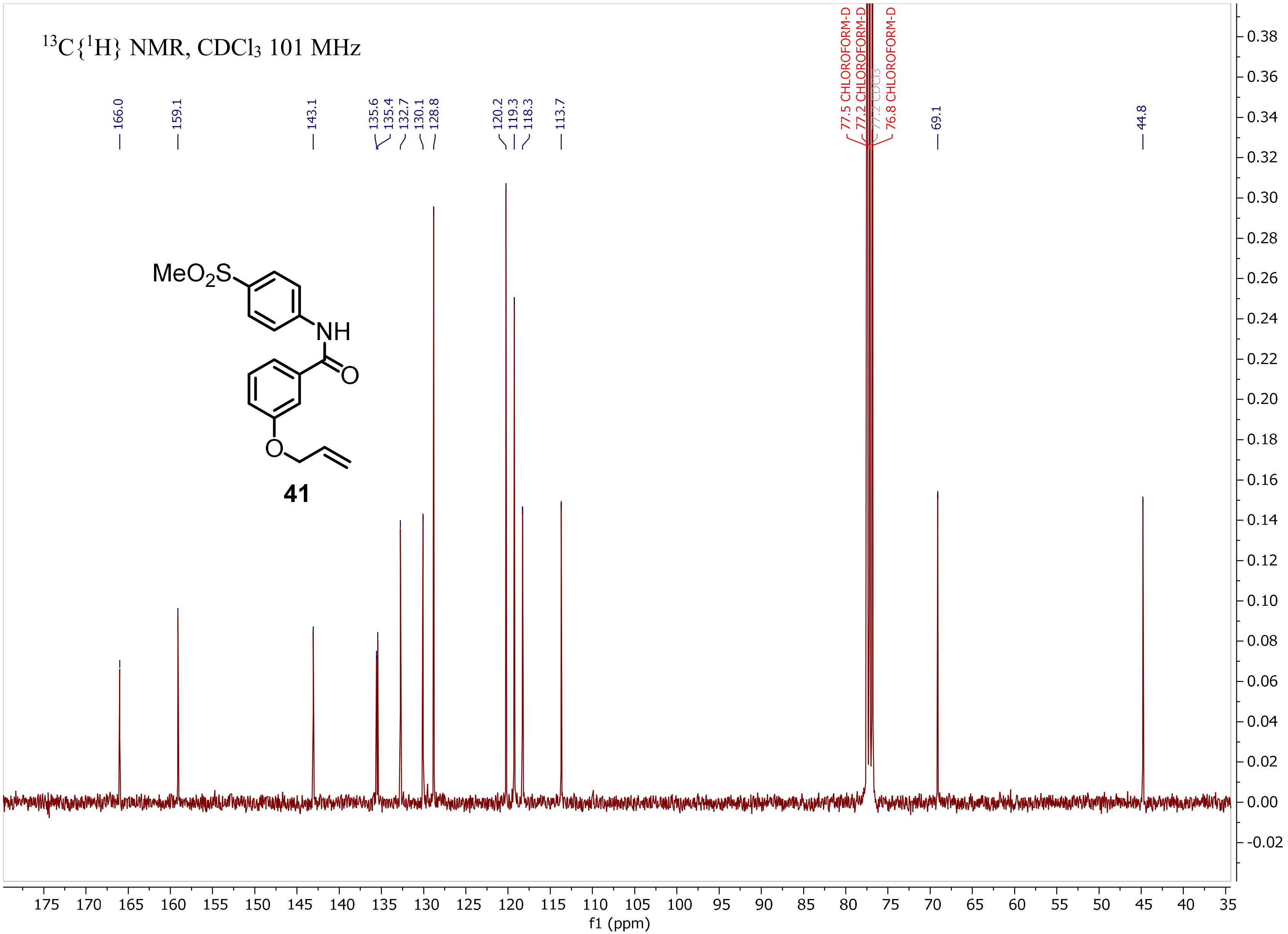

$^{13}C\{^{1}H\}$ NMR, $CDCl_3$ 101 MHz
166.0
159.1
143.1
135.6
135.4
132.7
130.1
128.8
120.2
119.3
118.3
113.7
77.5 CHLOROFORM-D
77.2 CHLOROFORM-D
77.2 CDCl3
76.8 CHLOROFORM-D
69.1
44.8
MeO2S
NH
O
O
41
f1 (ppm)

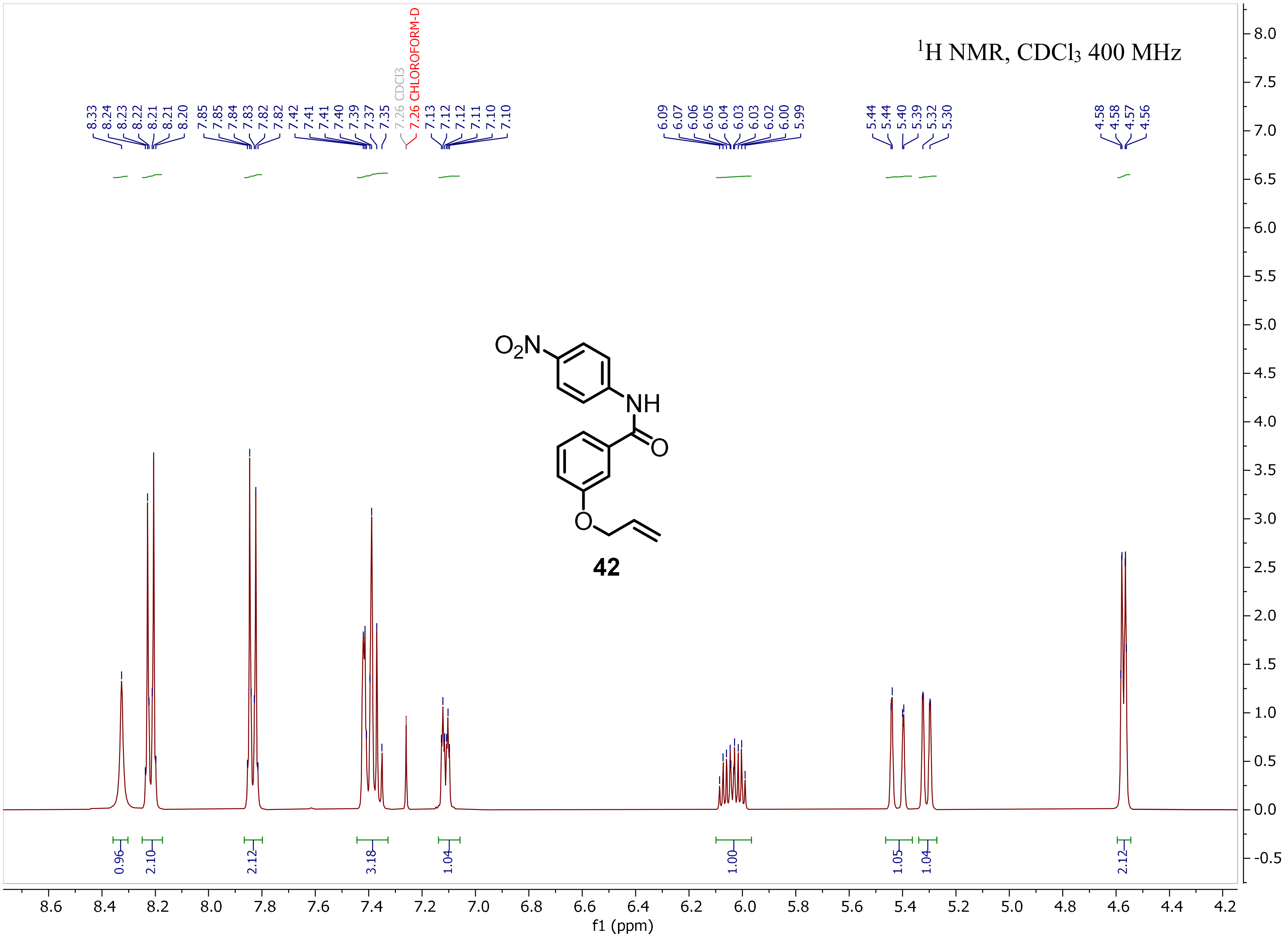

¹H NMR, CDCl3 400 MHz
8.33 8.24 8.23 8.22 8.21 8.21 8.20 7.85 7.85 7.84 7.83 7.82 7.42 7.41 7.41 7.40 7.39 7.37 7.35
7.26 CDCl3
7.26 CHLOROFORM-D
7.13 7.12 7.12 7.11 7.10 7.10
6.09 6.07 6.06 6.05 6.04 6.03 6.03 6.02 6.00 5.99
5.44 5.44 5.40 5.39 5.32 5.30
4.58 4.58 4.57 4.56
0.96 2.10 2.12 3.18 1.04 1.00 1.05 1.04 2.12
O2N
NH
O
O
42
f1 (ppm)

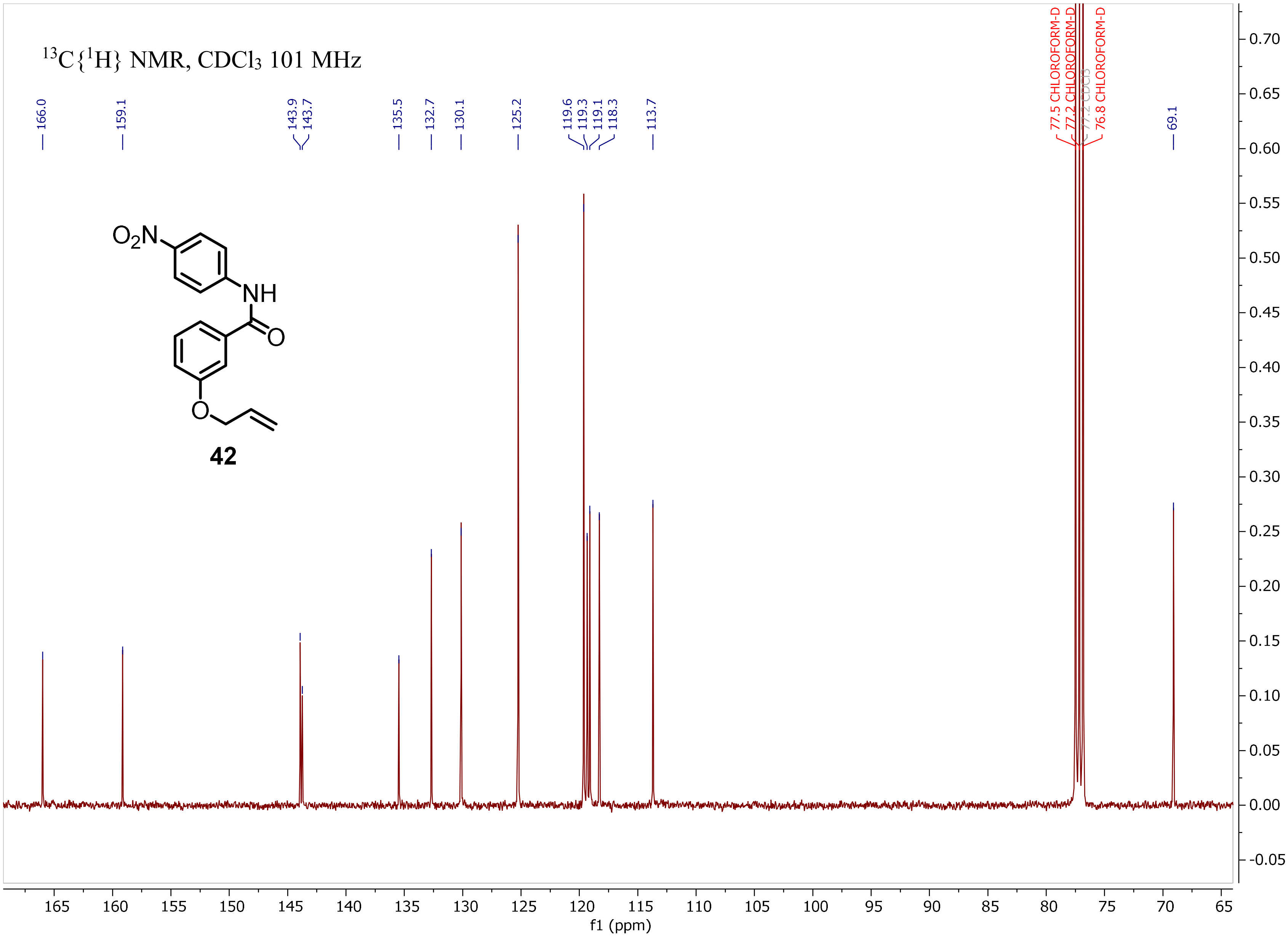

$^{13}C\{^1H\}$ NMR, $CDCl_3$ 101 MHz
166.0
159.1
143.9
143.7
135.5
132.7
130.1
125.2
119.6
119.3
119.1
118.3
113.7
77.5 CHLOROFORM-D
77.2 CHLOROFORM-D
77.2 CDCl3
76.8 CHLOROFORM-D
69.1
O2N
NH
O
O
42
f1 (ppm)

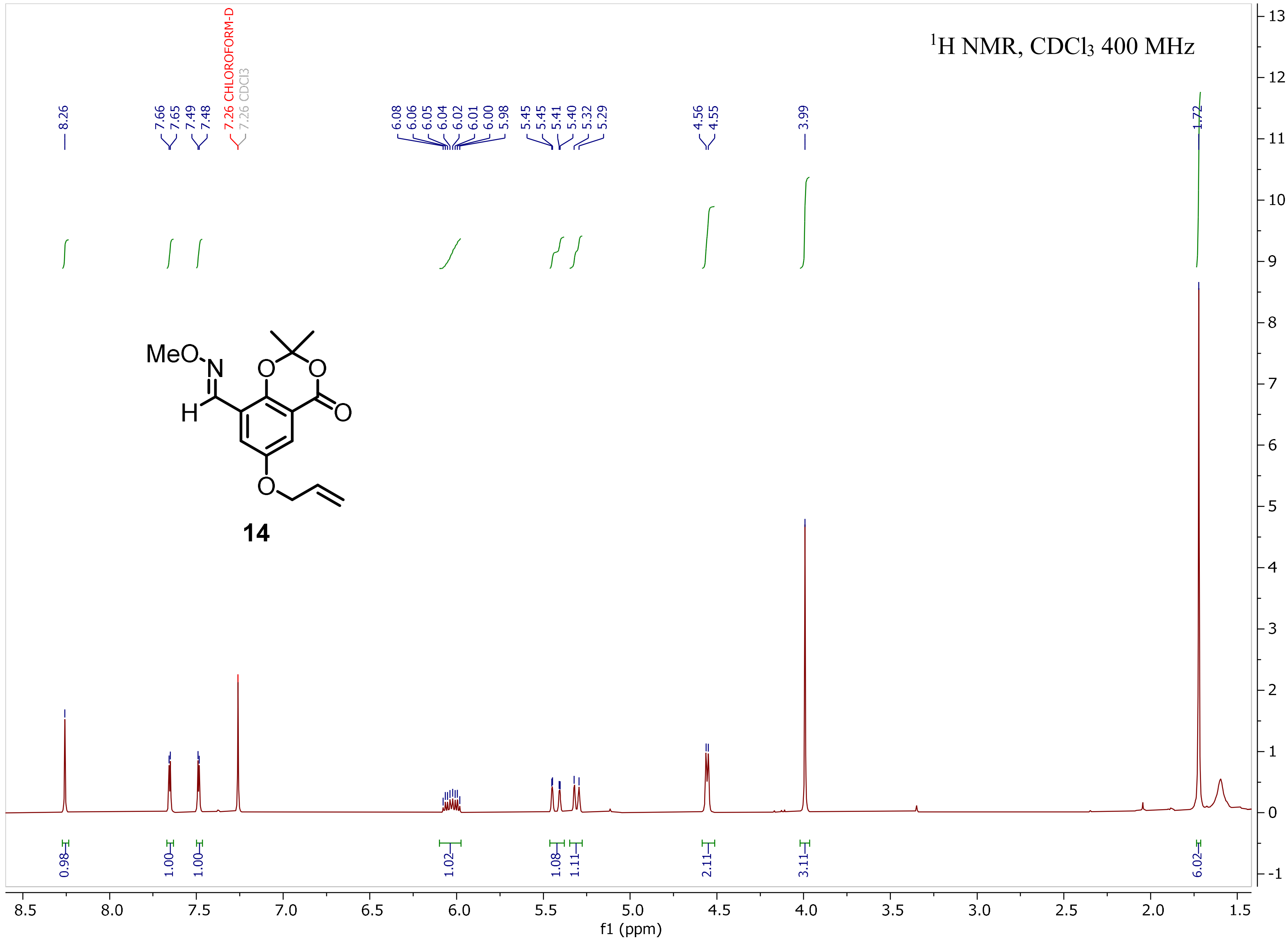

1H NMR, CDCl3 400 MHz
8.26
7.66
7.65
7.49
7.48
7.26 CHLOROFORM-D
7.26 CDCl3
6.08
6.06
6.05
6.04
6.02
6.01
6.00
5.98
5.45
5.45
5.41
5.40
5.32
5.29
4.56
4.55
3.99
1.72
MeO
N
H
O
O
O
O
14
0.98
1.00
1.00
1.02
1.08
1.11
2.11
3.11
6.02
f1 (ppm)

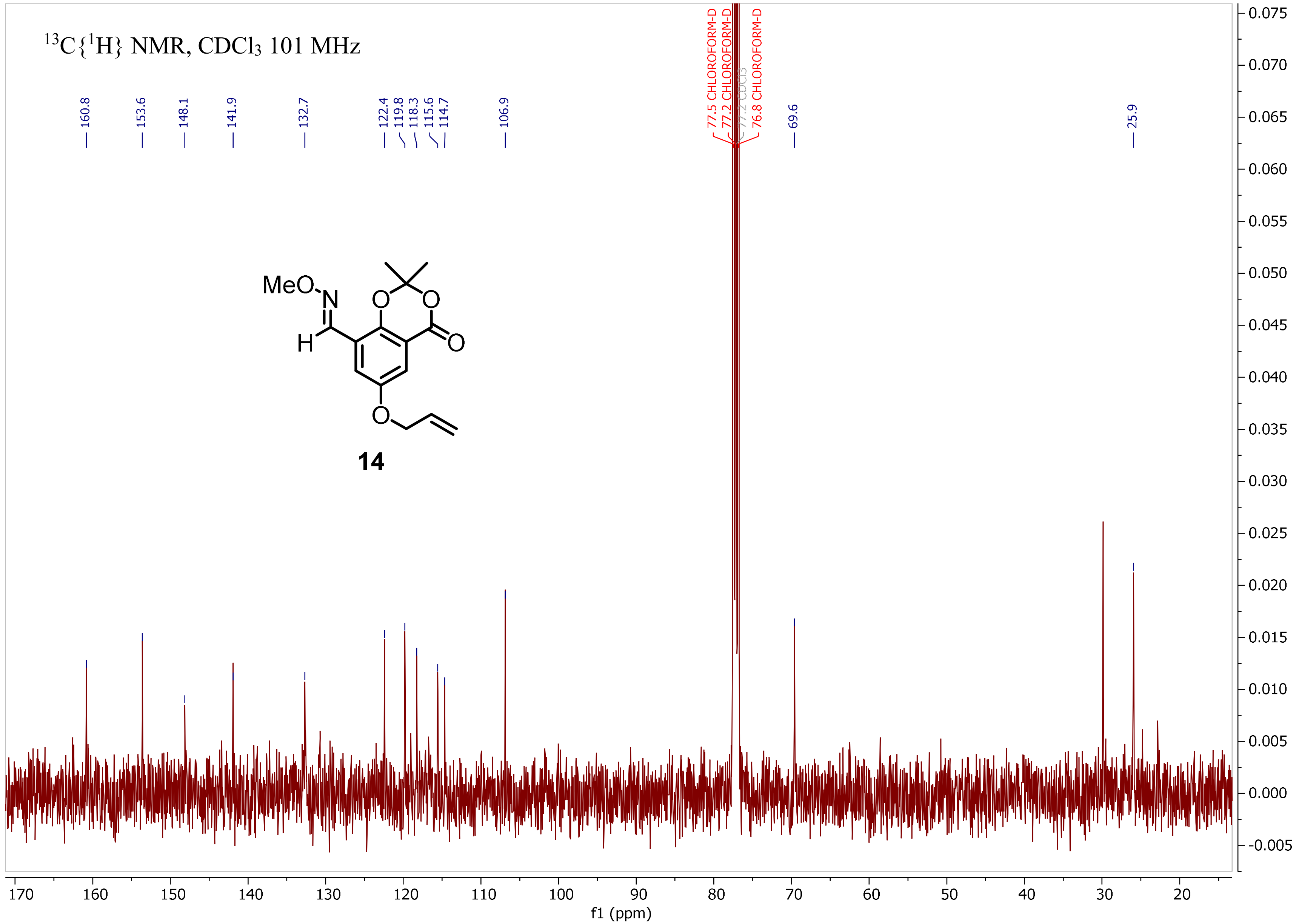

$^{13}C\{^{1}H\}$ NMR, $CDCl_3$ 101 MHz
160.8
153.6
148.1
141.9
132.7
122.4
119.8
118.3
115.6
114.7
106.9
77.5 CHLOROFORM-D
77.2 CHLOROFORM-D
77.2 CDCl3
76.8 CHLOROFORM-D
69.6
25.9
MeO
N
H
O
O
O
O
14
f1 (ppm)

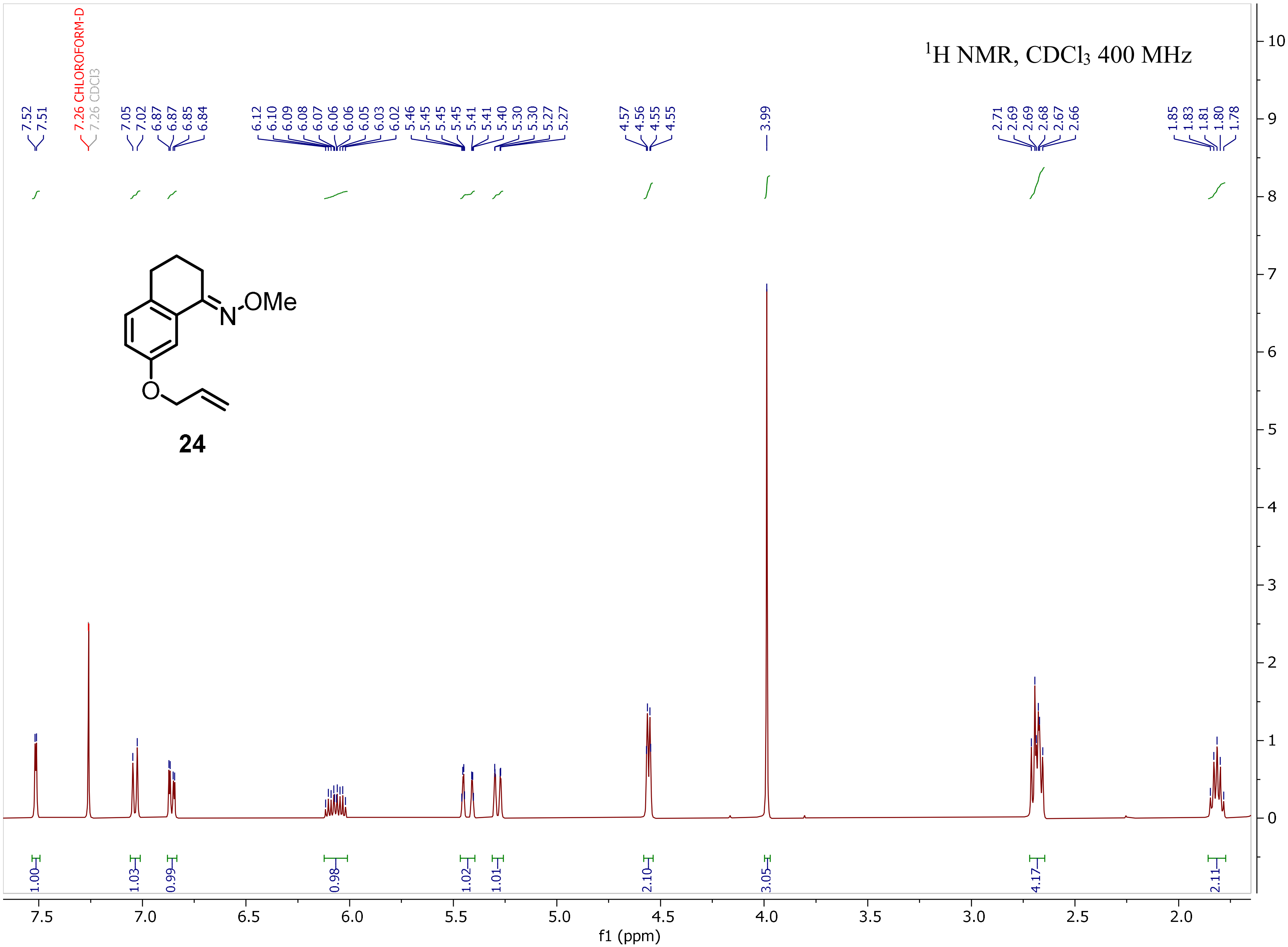
1H NMR, CDCl3 400 MHz
7.52
7.51
7.26 CHLOROFORM-D
7.26 CDCl3
7.05
7.02
6.87
6.87
6.85
6.84
6.12
6.10
6.09
6.08
6.07
6.06
6.06
6.05
6.03
6.02
5.46
5.45
5.45
5.45
5.41
5.41
5.40
5.30
5.30
5.27
5.27
4.57
4.56
4.55
4.55
3.99
2.71
2.69
2.69
2.68
2.67
2.66
1.85
1.83
1.81
1.80
1.78
OMe
N
O
24
1.00
1.03
0.99
0.98
1.02
1.01
2.10
3.05
4.17
2.11
f1 (ppm)

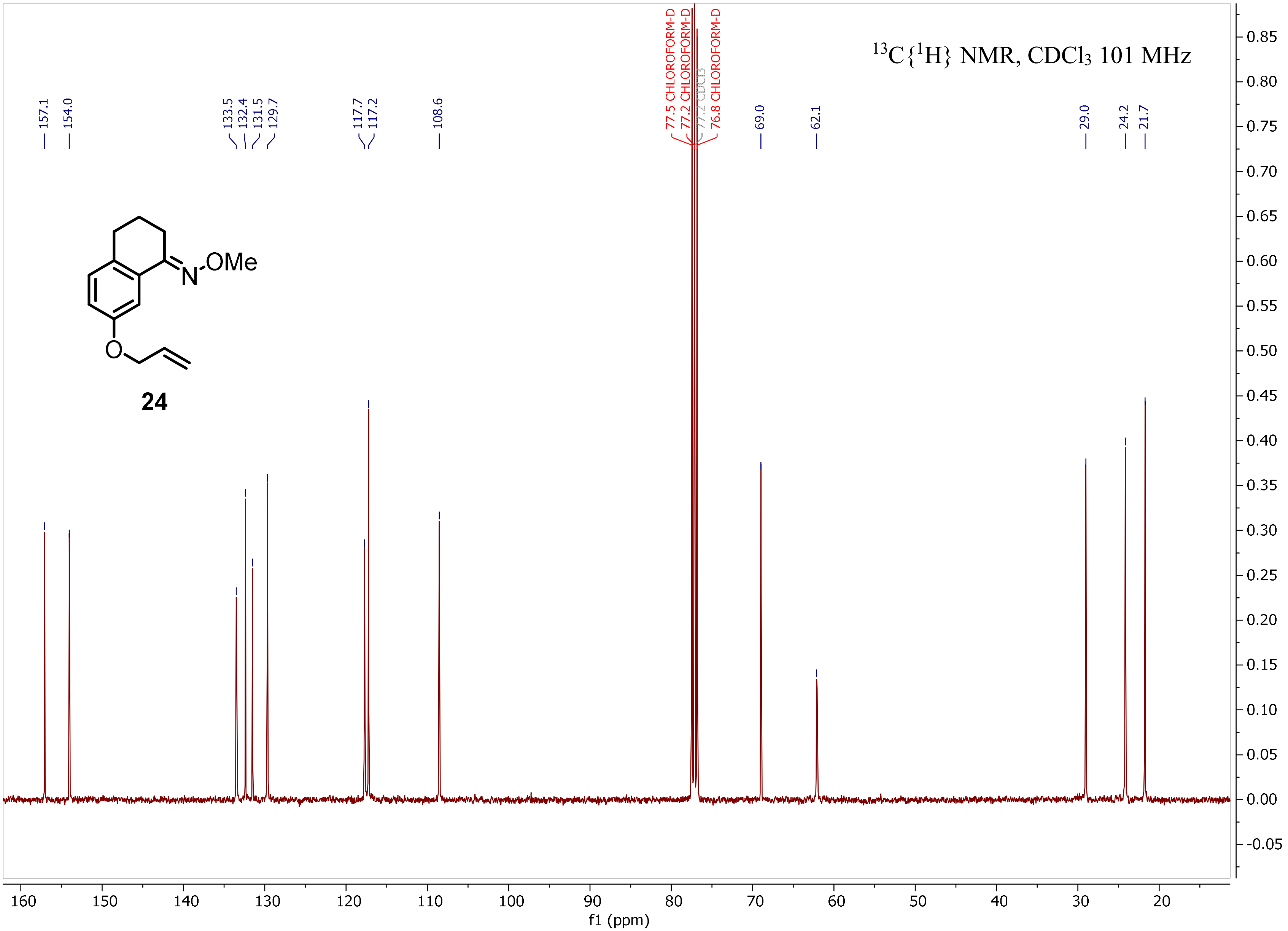

$^{13}C\{^1H\}$ NMR, $CDCl_3$ 101 MHz
157.1
154.0
133.5
132.4
131.5
129.7
117.7
117.2
108.6
77.5 CHLOROFORM-D
77.2 CHLOROFORM-D
77.2 CDCl3
76.8 CHLOROFORM-D
69.0
62.1
29.0
24.2
21.7
OMe
N
O
24
f1 (ppm)

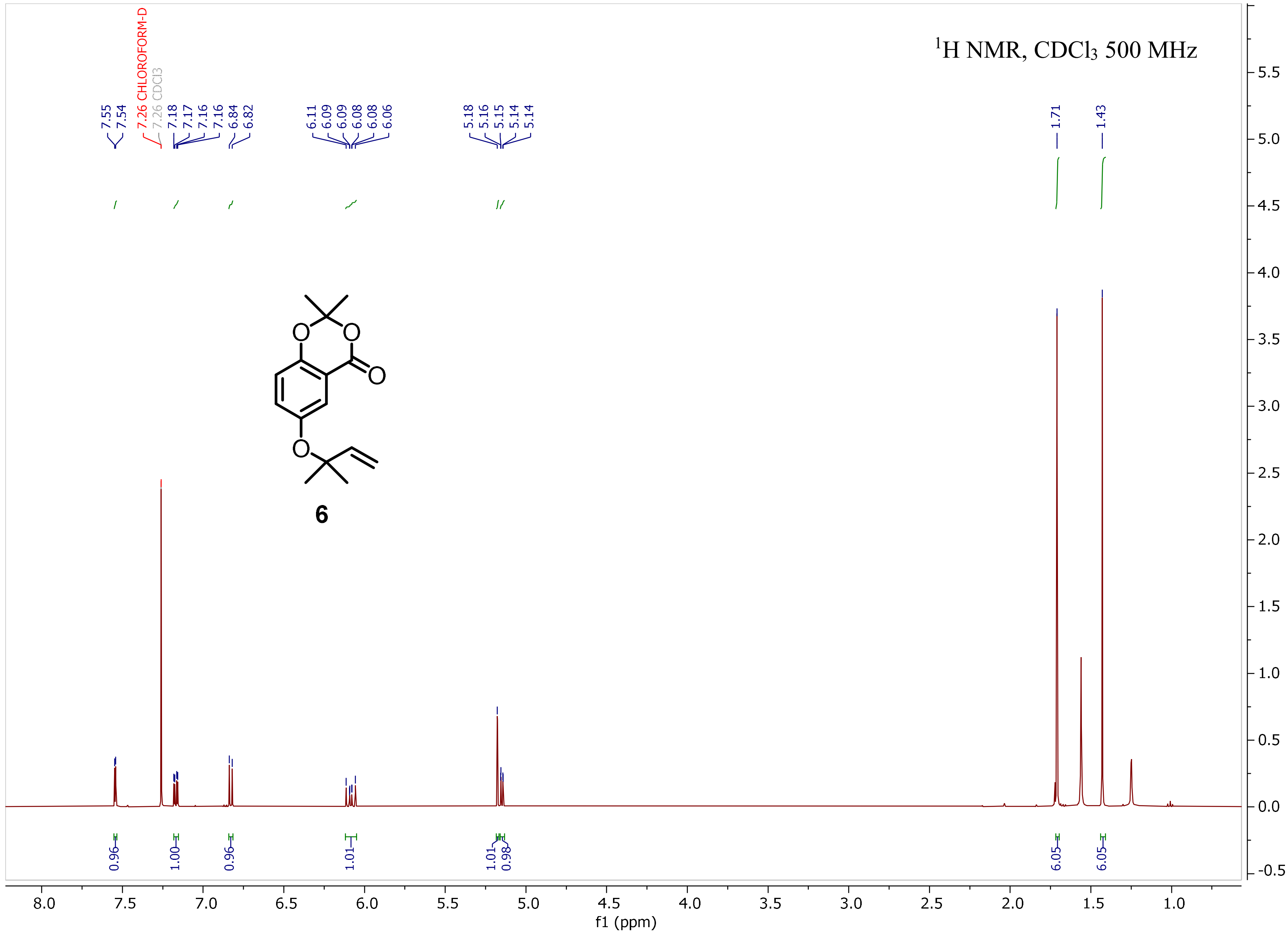

^{1}H NMR, $CDCl_3$ 500 MHz
7.55
7.54
7.26 CHLOROFORM-D
7.26 CDCl3
7.18
7.17
7.16
7.16
6.84
6.82
6.11
6.09
6.09
6.08
6.08
6.06
5.18
5.16
5.15
5.14
5.14
1.71
1.43
6
0.96
1.00
0.96
1.01
1.01
0.98
6.05
6.05
f1 (ppm)

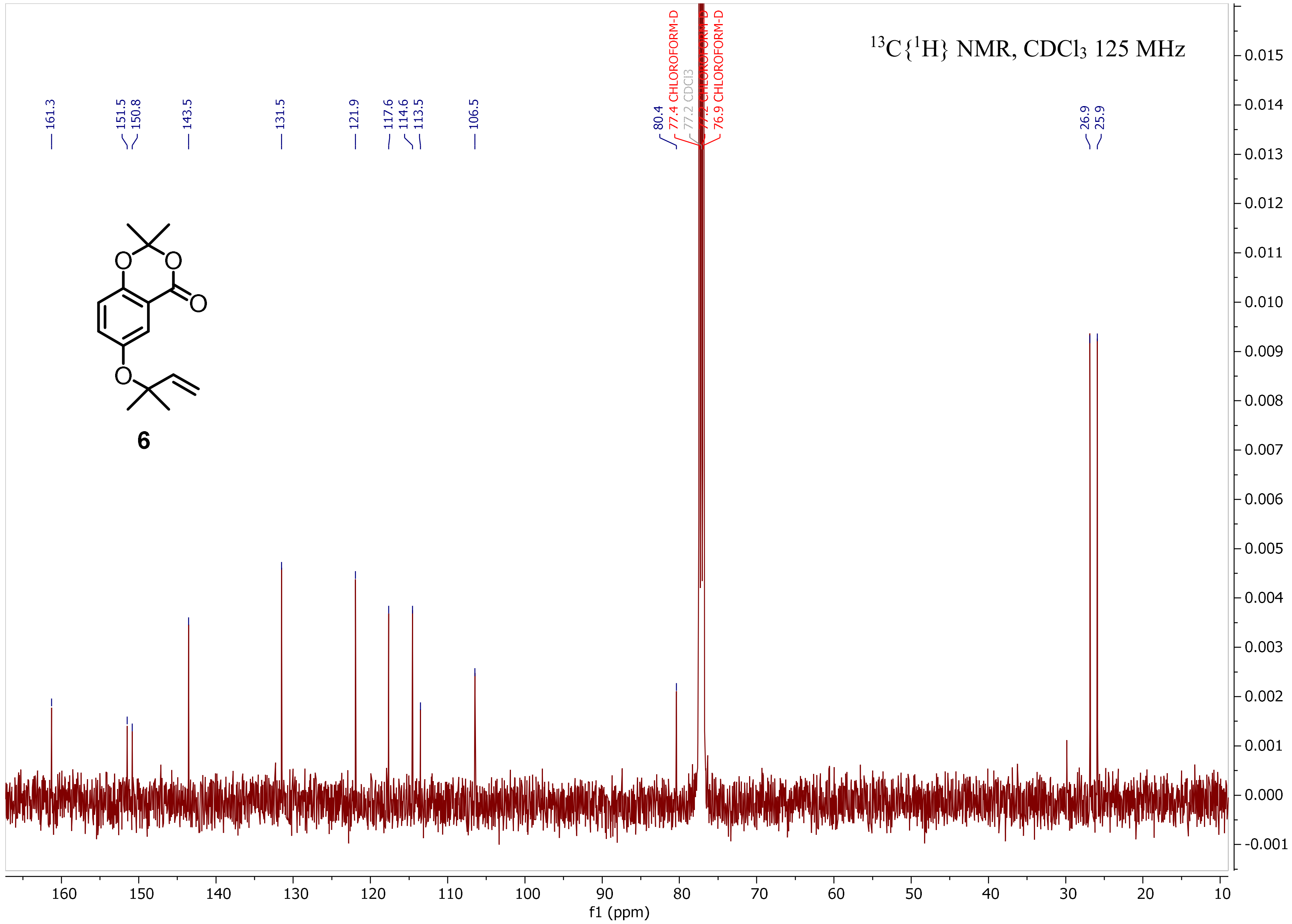
$^{13}C\{^1H\}$ NMR, $CDCl_3$ 125 MHz
161.3
151.5
150.8
143.5
131.5
121.9
117.6
114.6
113.5
106.5
80.4
77.4 CHLOROFORM-D
77.2 CDCl3
77.2 CHLOROFORM-D
76.9 CHLOROFORM-D
26.9
25.9
f1 (ppm)
6

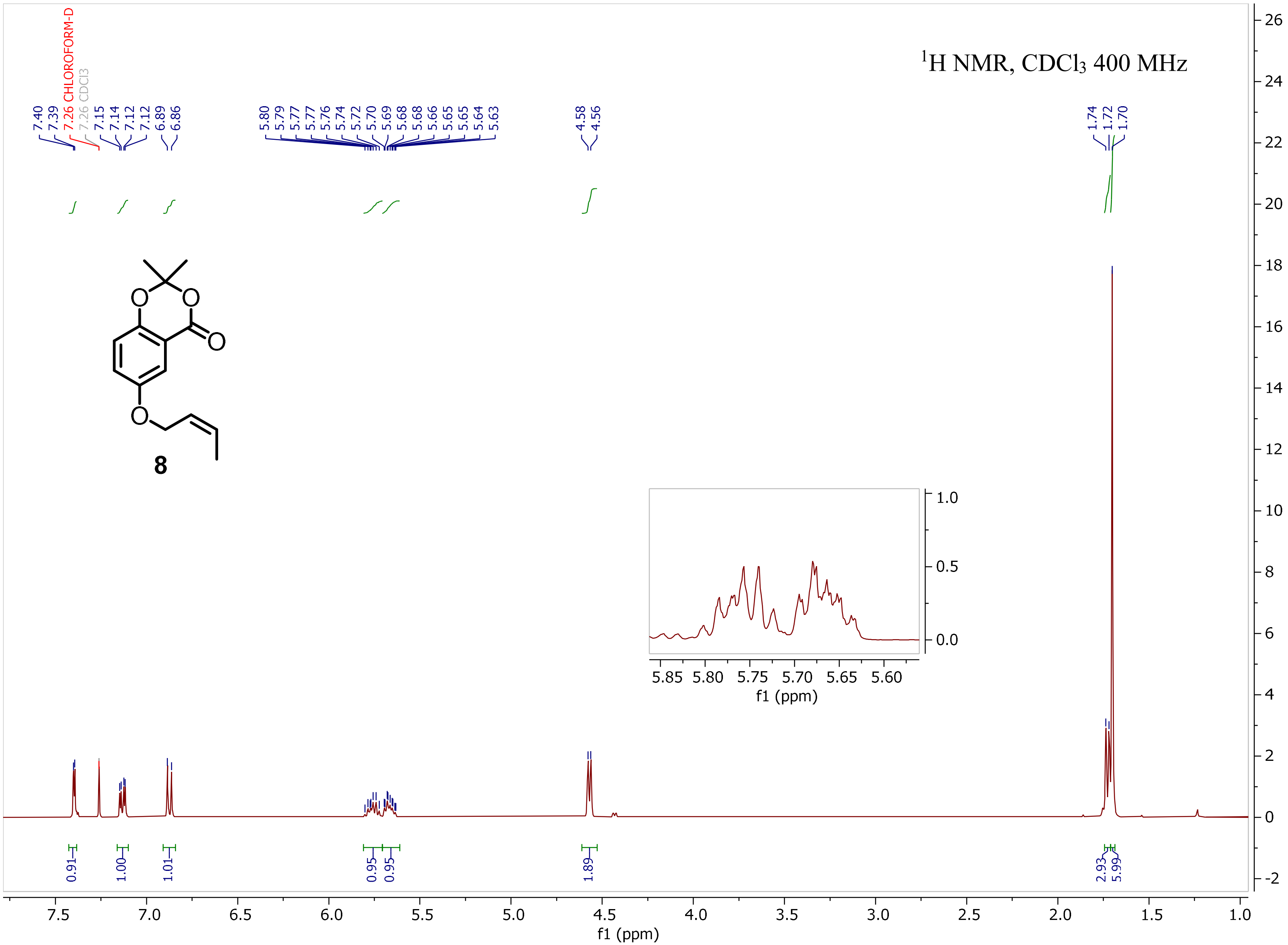
1H NMR, CDCl3 400 MHz
8
f1 (ppm)

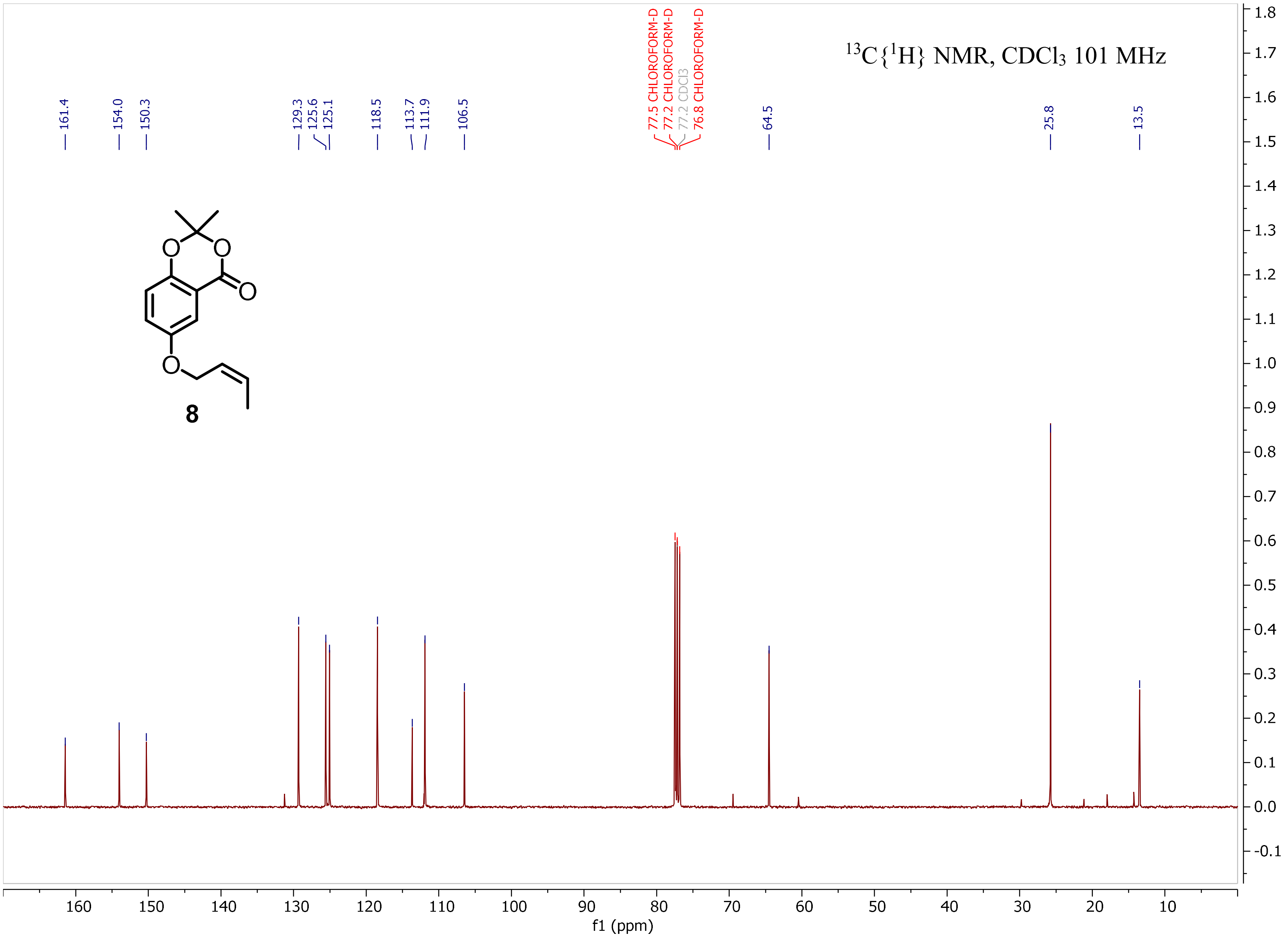

$^{13}C\{^1H\}$ NMR, $CDCl_3$ 101 MHz
161.4
154.0
150.3
129.3
125.6
125.1
118.5
113.7
111.9
106.5
77.5 CHLOROFORM-D
77.2 CHLOROFORM-D
77.2 CDCl3
76.8 CHLOROFORM-D
64.5
25.8
13.5
8
f1 (ppm)

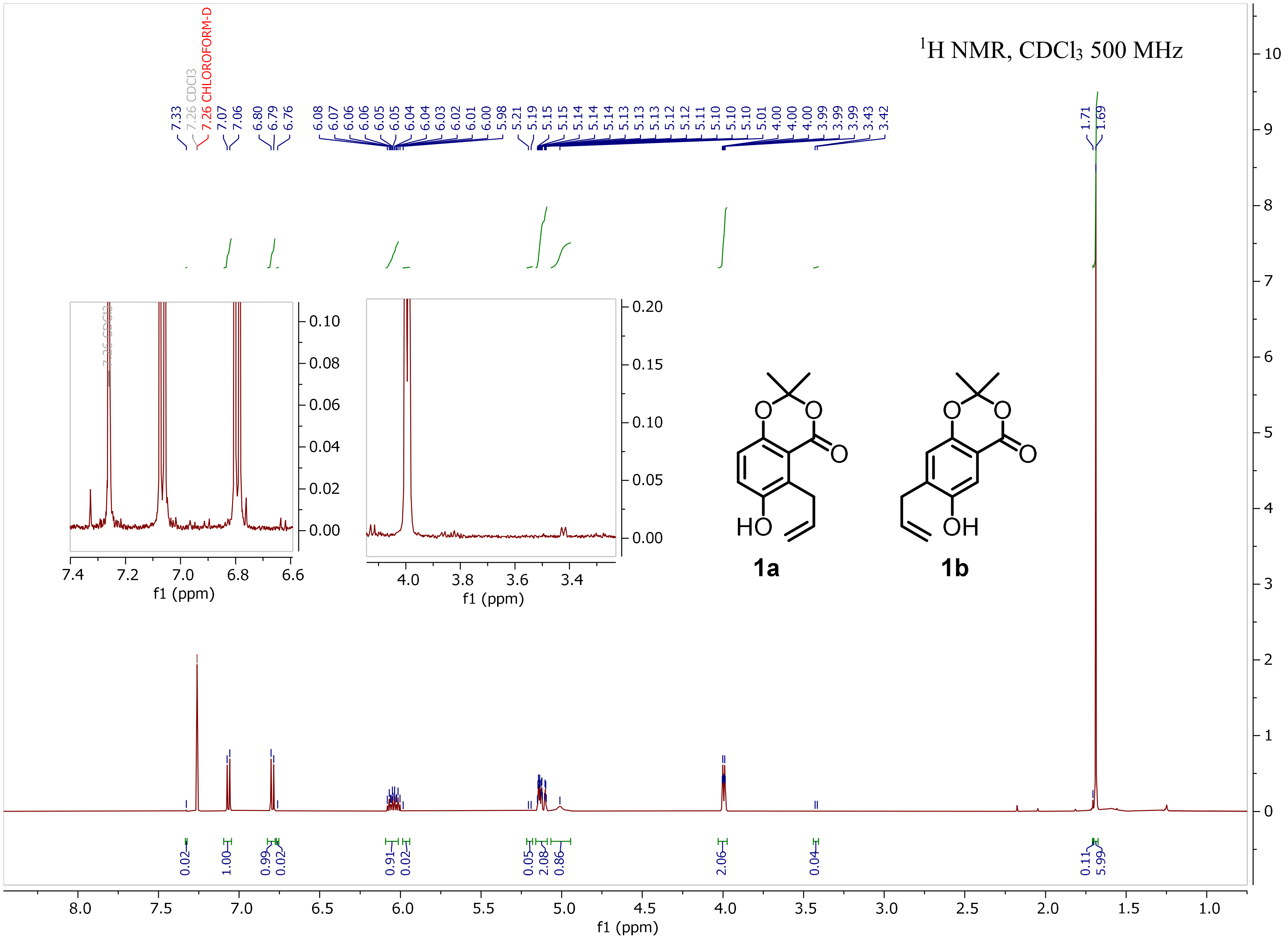

1H NMR, CDCl3 500 MHz
1a
1b
HO
OH
f1 (ppm)

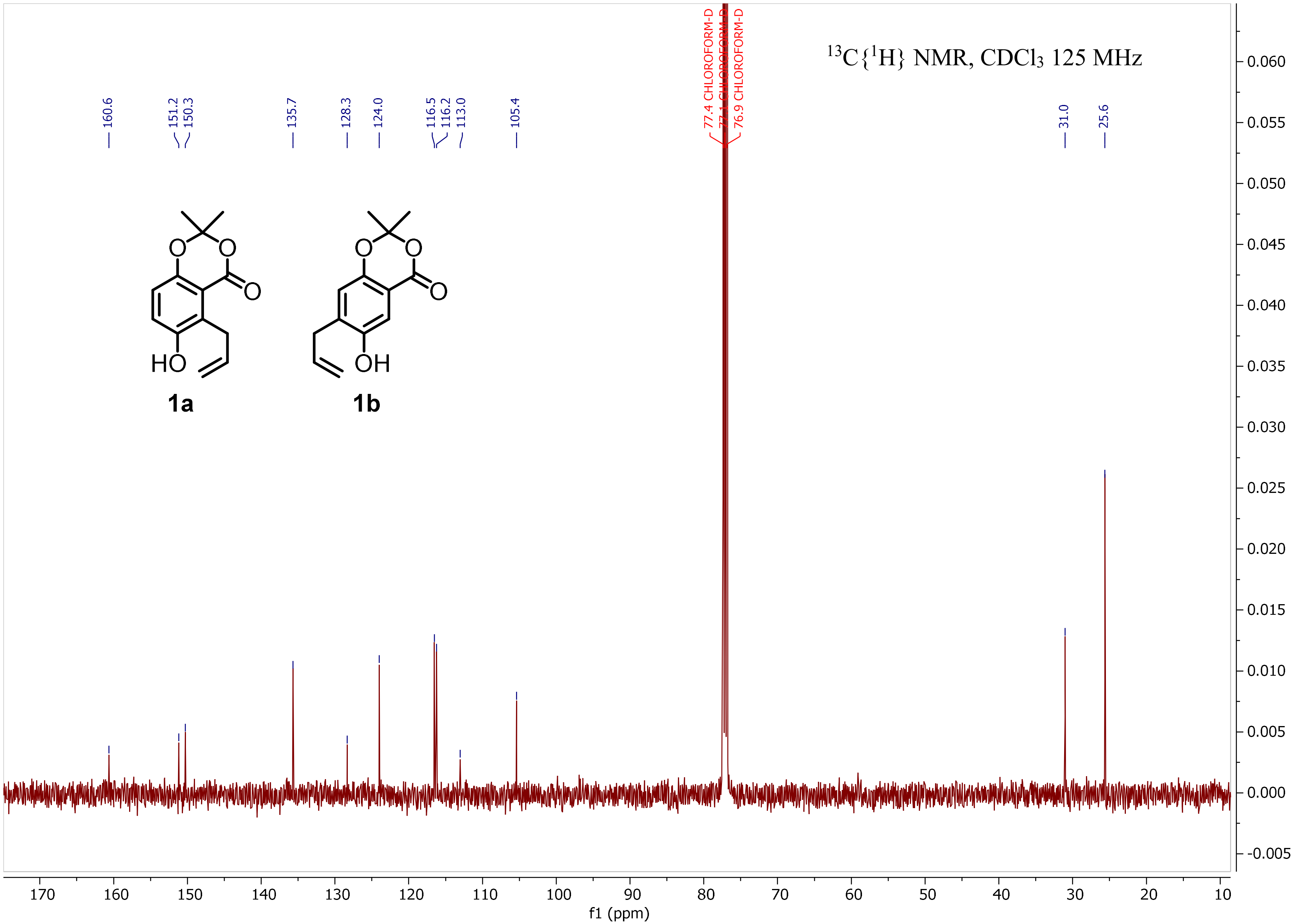

$^{13}C\{^1H\}$ NMR, $CDCl_3$ 125 MHz
160.6
151.2
150.3
135.7
128.3
124.0
116.5
116.2
113.0
105.4
77.4 CHLOROFORM-D
77.1 CHLOROFORM-D
76.9 CHLOROFORM-D
31.0
25.6
1a
1b
f1 (ppm)

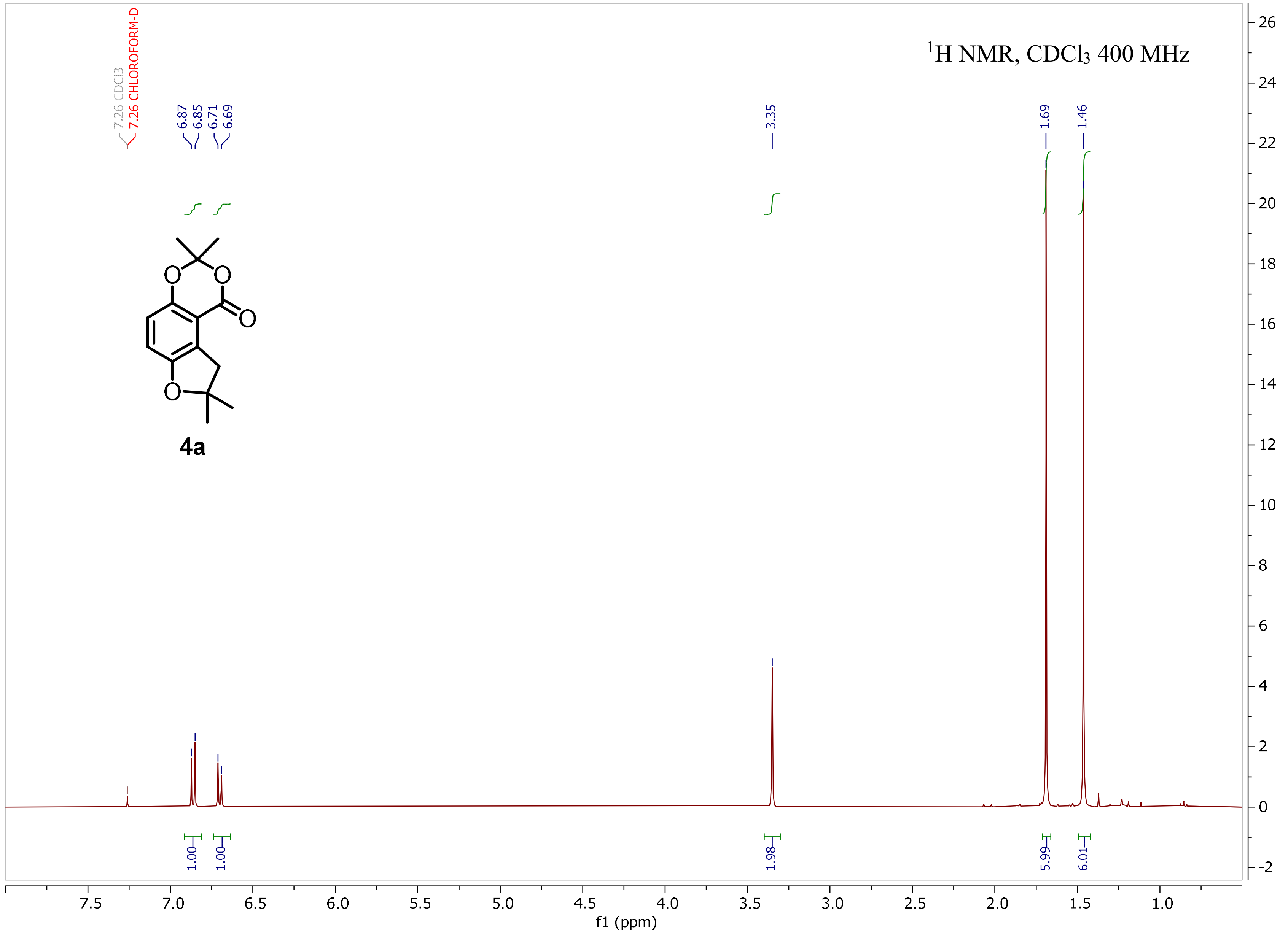

^{1}H NMR, CDCl$_3$ 400 MHz
7.26 CDCl3
7.26 CHLOROFORM-D
6.87
6.85
6.71
6.69
3.35
1.69
1.46
4a
1.00
1.00
1.98
5.99
6.01
f1 (ppm)

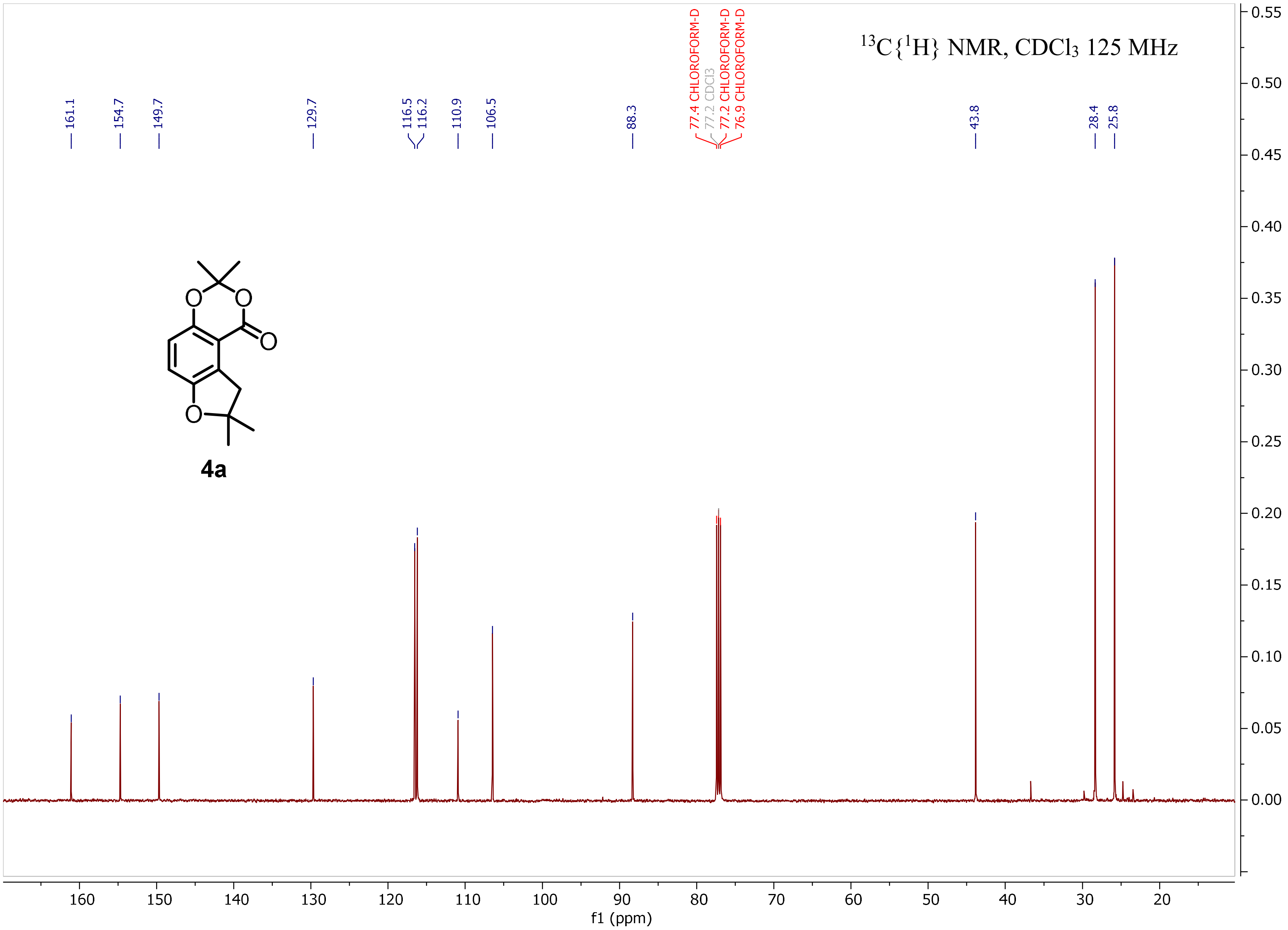

$^{13}C\{^{1}H\}$ NMR, $CDCl_3$ 125 MHz
161.1
154.7
149.7
129.7
116.5
116.2
110.9
106.5
88.3
77.4 CHLOROFORM-D
77.2 CDCl3
77.2 CHLOROFORM-D
76.9 CHLOROFORM-D
43.8
28.4
25.8
4a
f1 (ppm)

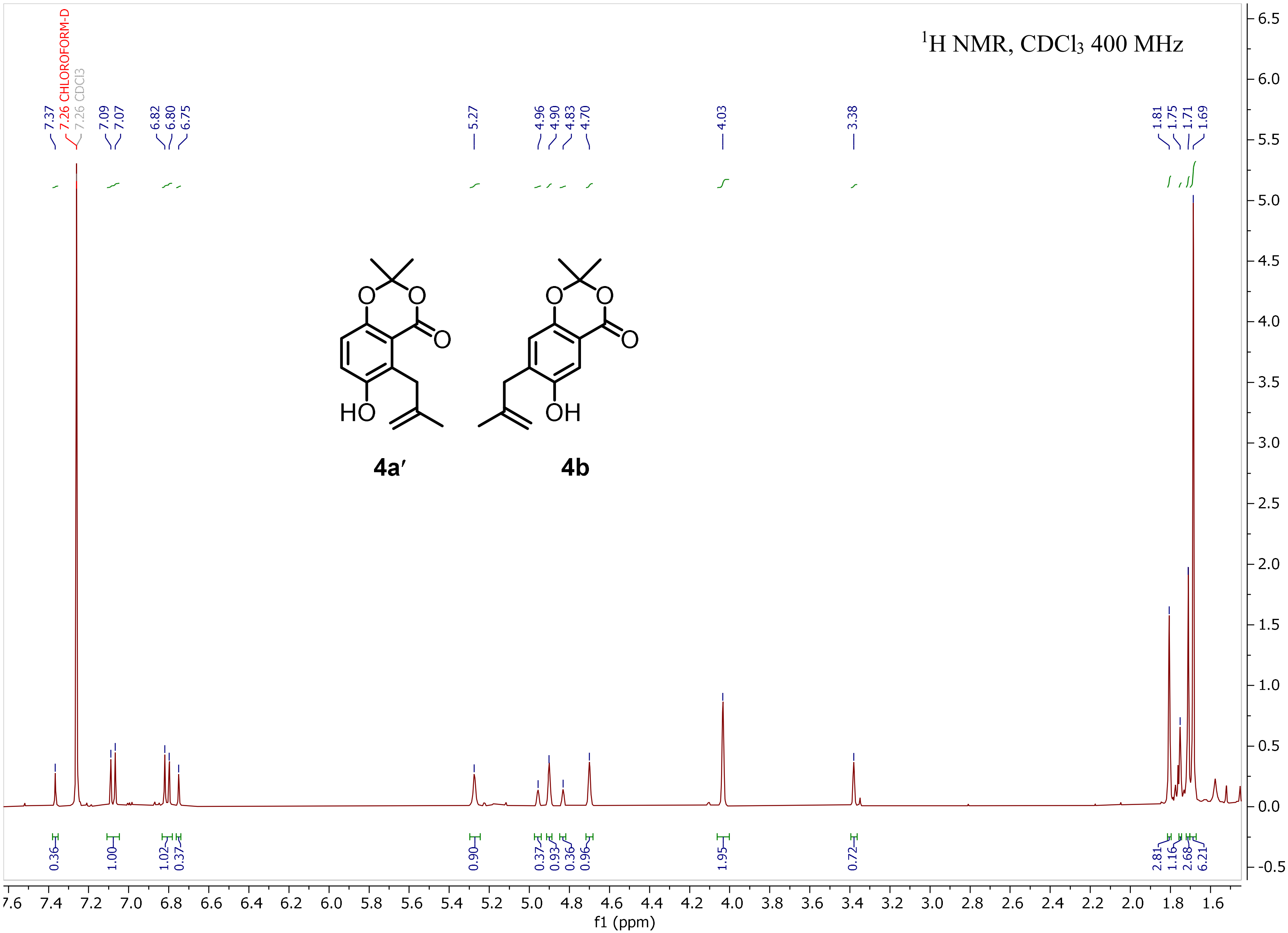

¹H NMR, CDCl3 400 MHz
4a′
4b
f1 (ppm)

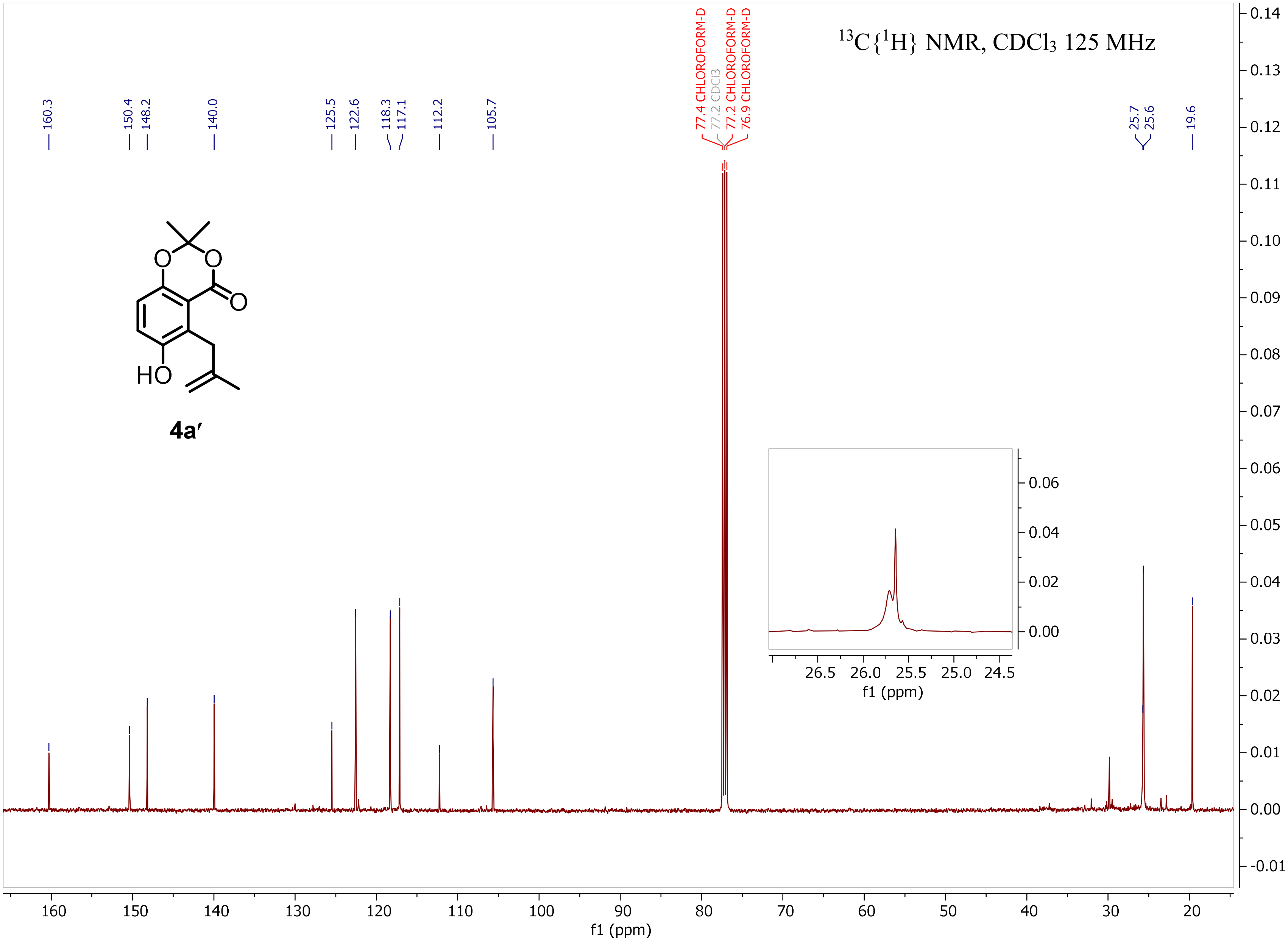

¹³C{¹H} NMR, CDCl₃ 125 MHz
160.3
150.4
148.2
140.0
125.5
122.6
118.3
117.1
112.2
105.7
77.4 CHLOROFORM-D
77.2 CDCl3
77.2 CHLOROFORM-D
76.9 CHLOROFORM-D
25.7
25.6
19.6
4a′
f1 (ppm)

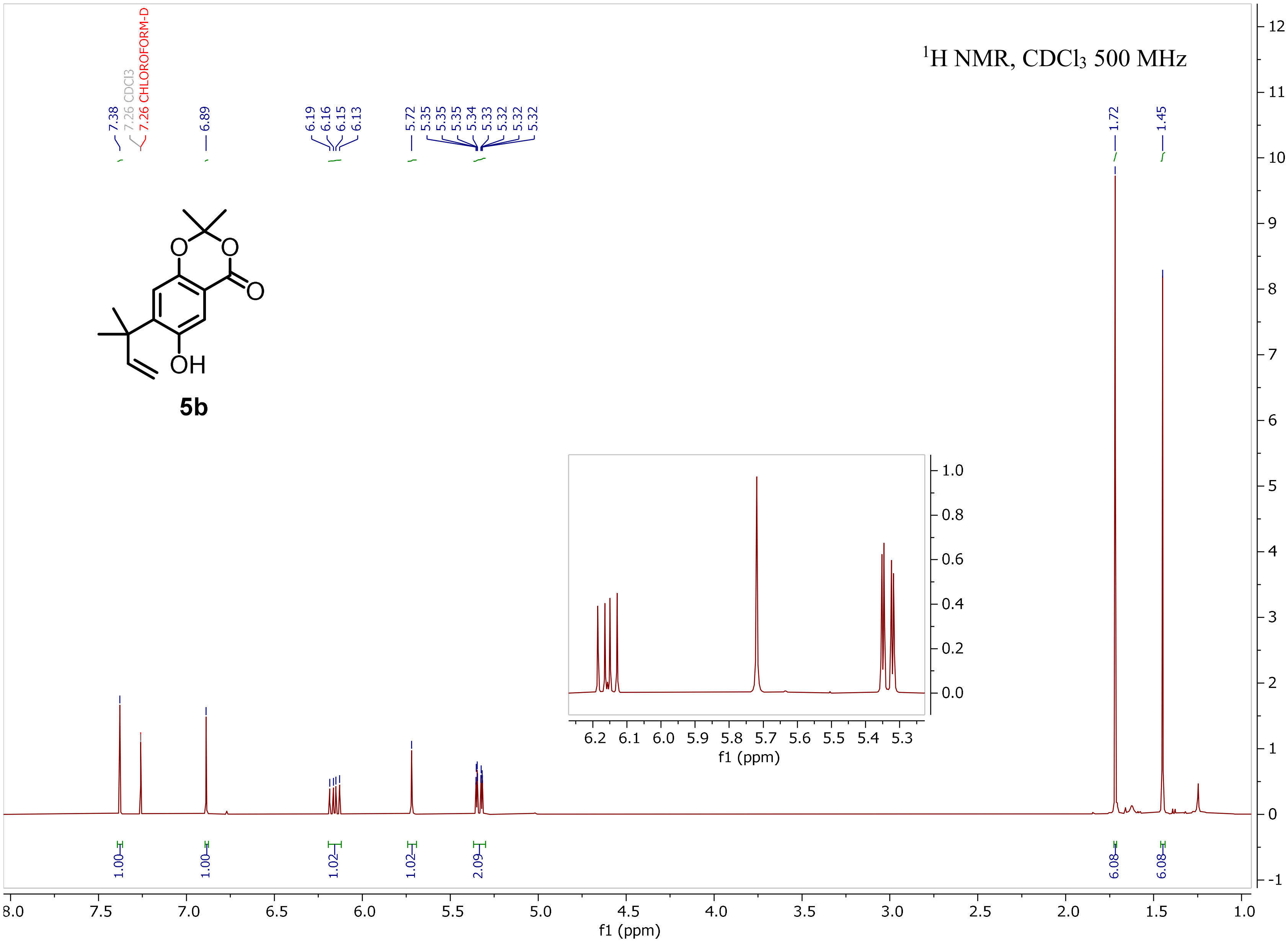

¹H NMR, CDCl3 500 MHz
7.38
7.26 CDCl3
7.26 CHLOROFORM-D
6.89
6.19
6.16
6.15
6.13
5.72
5.35
5.35
5.35
5.34
5.33
5.32
5.32
5.32
1.72
1.45
OH
5b
1.00
1.00
1.02
1.02
2.09
6.08
6.08
f1 (ppm)

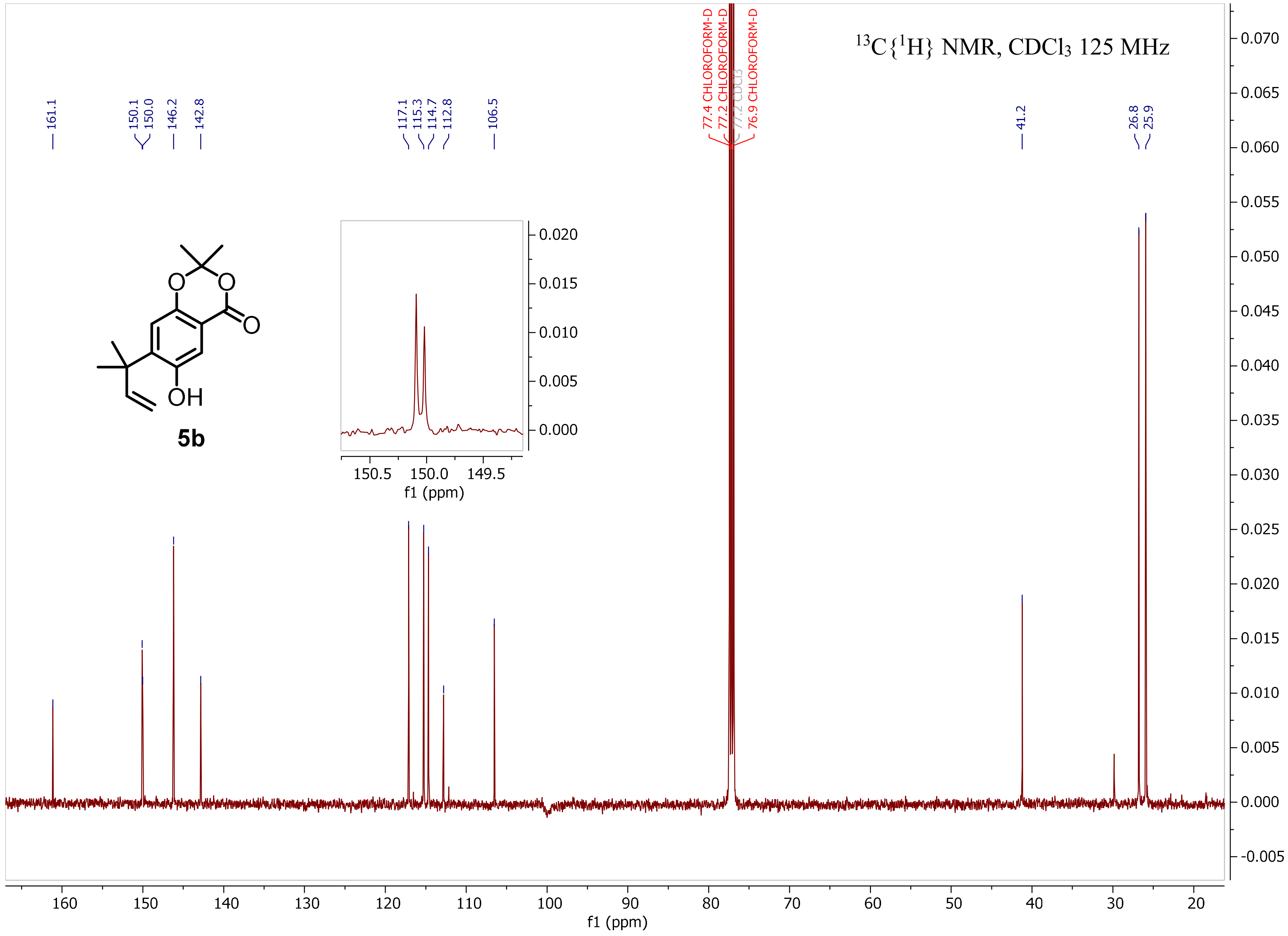

$^{13}C\{^1H\}$ NMR, $CDCl_3$ 125 MHz
161.1
150.1
150.0
146.2
142.8
117.1
115.3
114.7
112.8
106.5
77.4 CHLOROFORM-D
77.2 CHLOROFORM-D
77.2 CDCl3
76.9 CHLOROFORM-D
41.2
26.8
25.9
OH
O
5b
150.5
150.0
149.5
f1 (ppm)
0.020
0.015
0.010
0.005
0.000
160
150
140
130
120
110
100
90
80
70
60
50
40
30
20
f1 (ppm)
0.070
0.065
0.060
0.055
0.050
0.045
0.040
0.035
0.030
0.025
0.020
0.015
0.010
0.005
0.000
-0.005

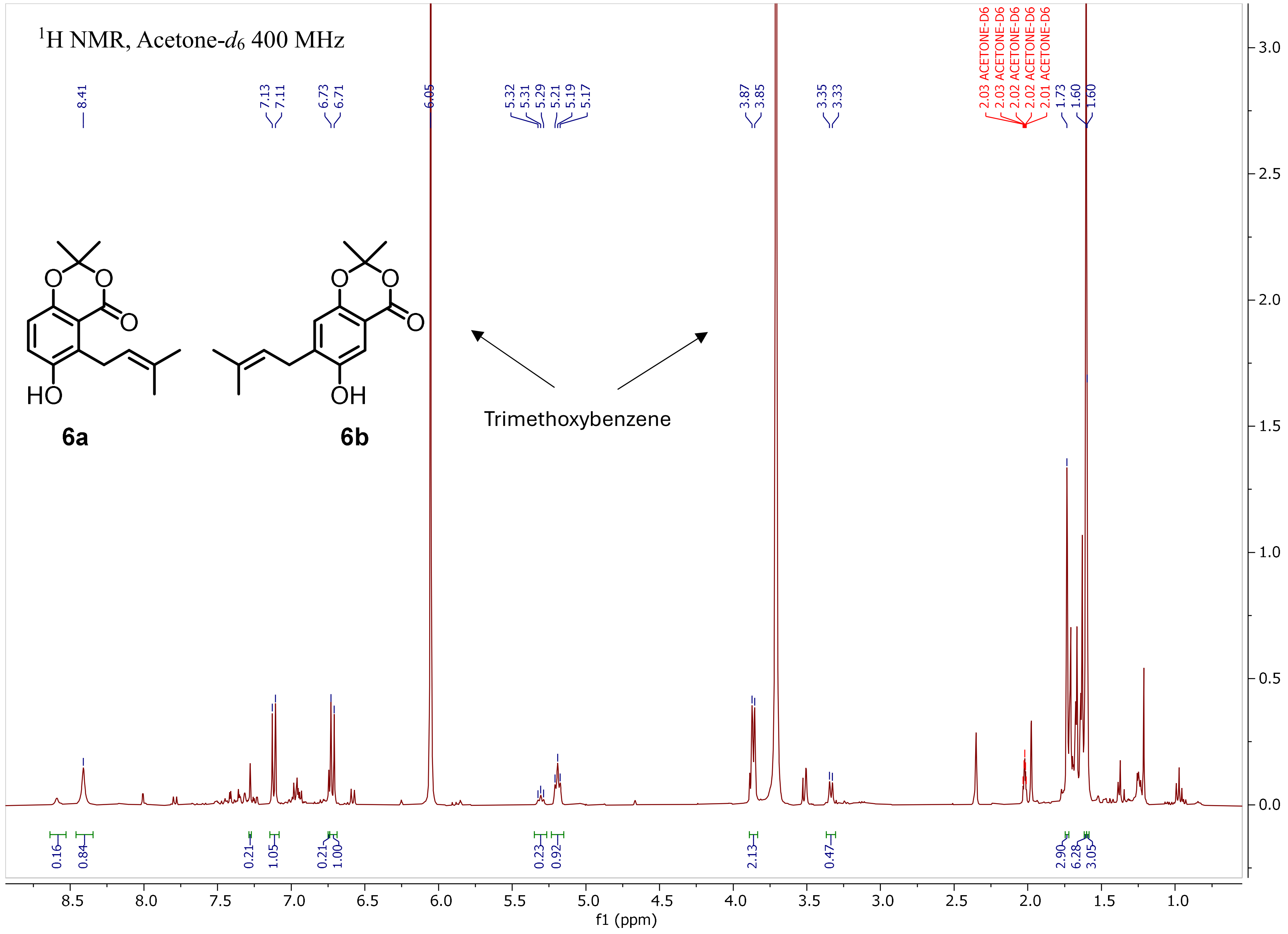

^{1}H NMR, Acetone-d_6 400 MHz
6a
6b
Trimethoxybenzene
8.41
7.13
7.11
6.73
6.71
6.05
5.32
5.31
5.29
5.21
5.19
5.17
3.87
3.85
3.35
3.33
2.03 ACETONE-D6
2.03 ACETONE-D6
2.02 ACETONE-D6
2.02 ACETONE-D6
2.01 ACETONE-D6
1.73
1.60
1.60
0.16
0.84
0.21
1.05
0.21
1.00
0.23
0.92
2.13
0.47
2.90
6.28
3.05
f1 (ppm)

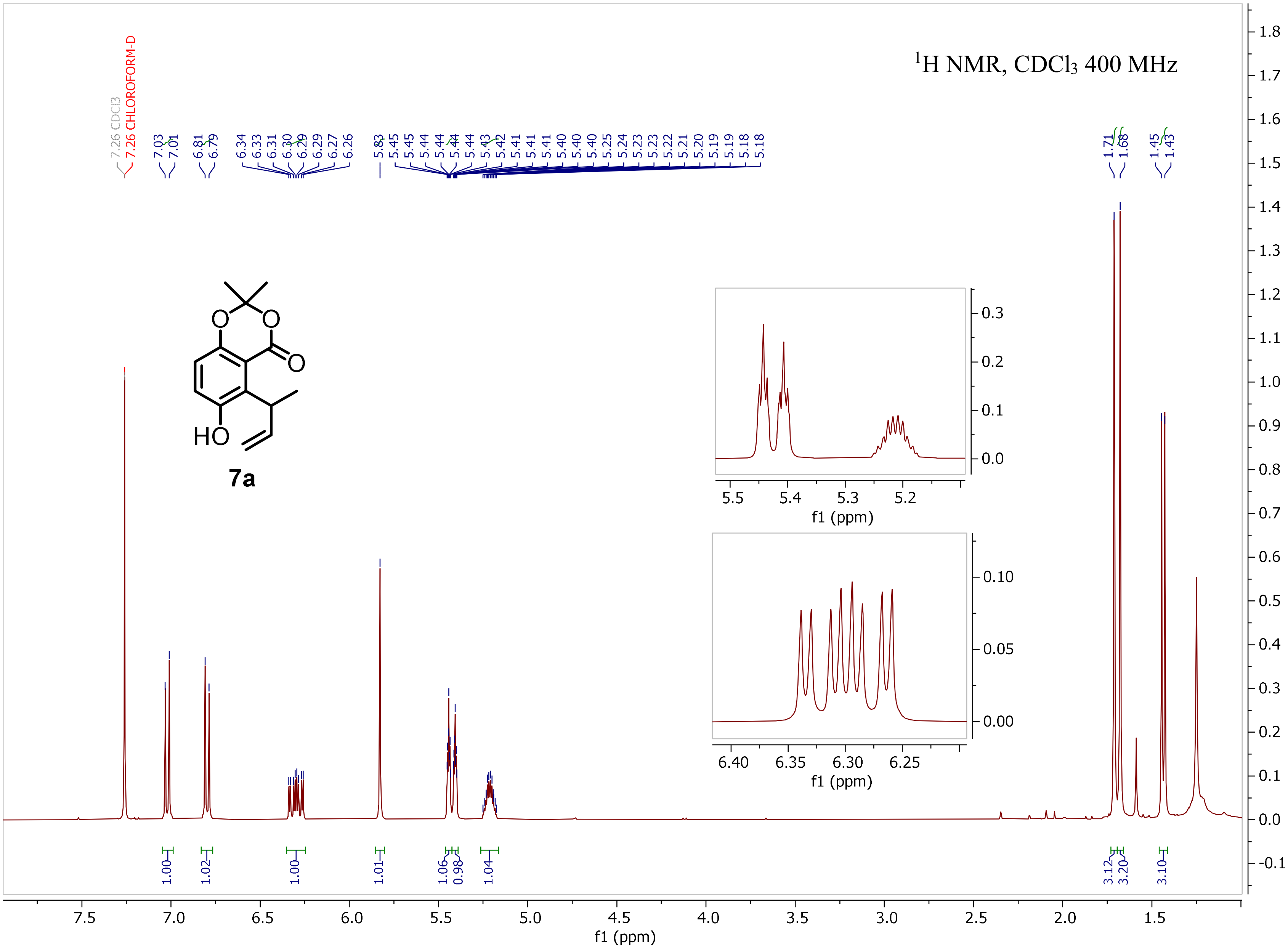

1H NMR, CDCl3 400 MHz
7a
HO
O
f1 (ppm)

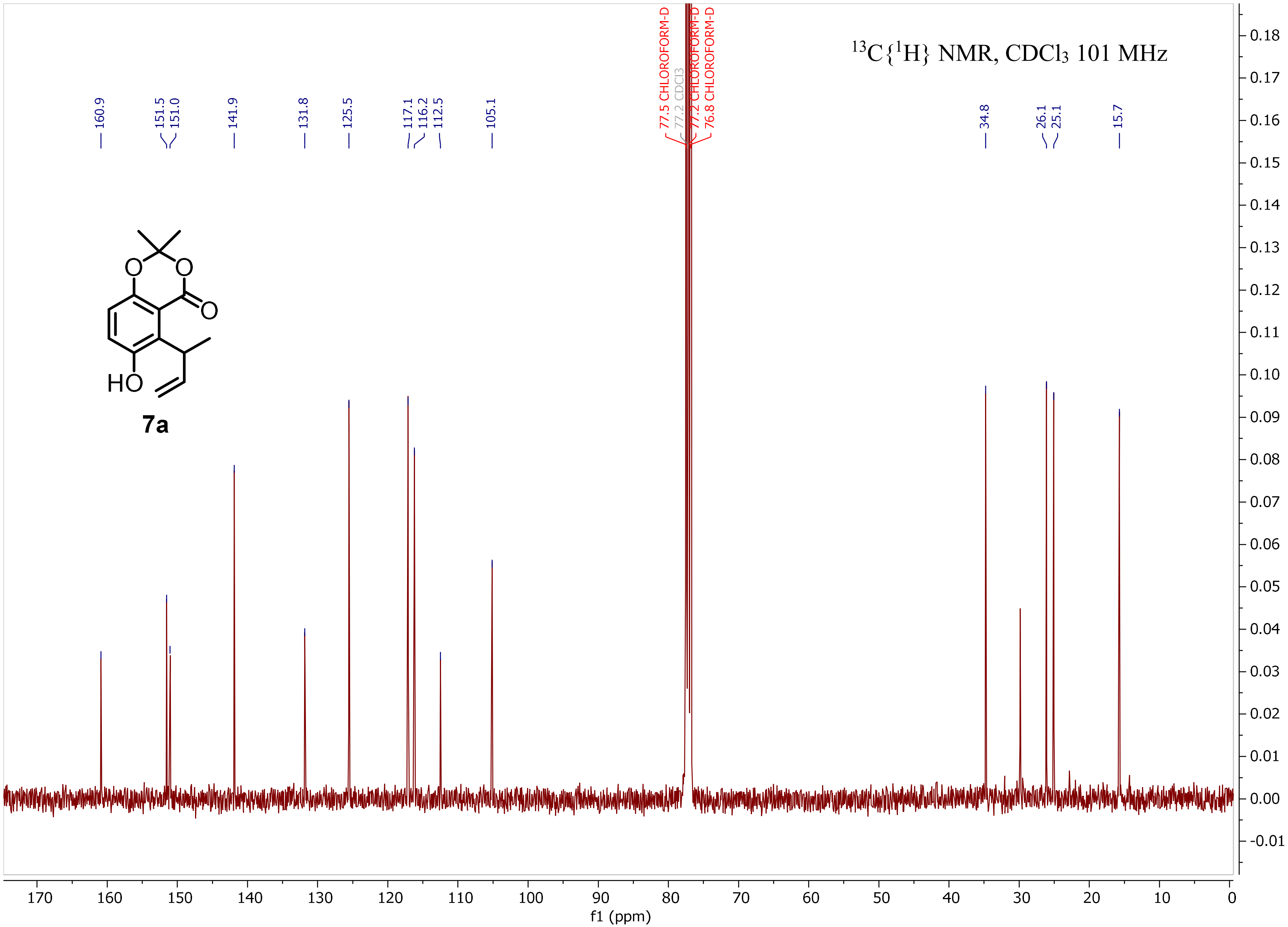

$^{13}C\{^1H\}$ NMR, $CDCl_3$ 101 MHz
160.9
151.5
151.0
141.9
131.8
125.5
117.1
116.2
112.5
105.1
77.5 CHLOROFORM-D
77.2 CDCl3
77.2 CHLOROFORM-D
76.8 CHLOROFORM-D
34.8
26.1
25.1
15.7
O
O
O
HO
7a
f1 (ppm)

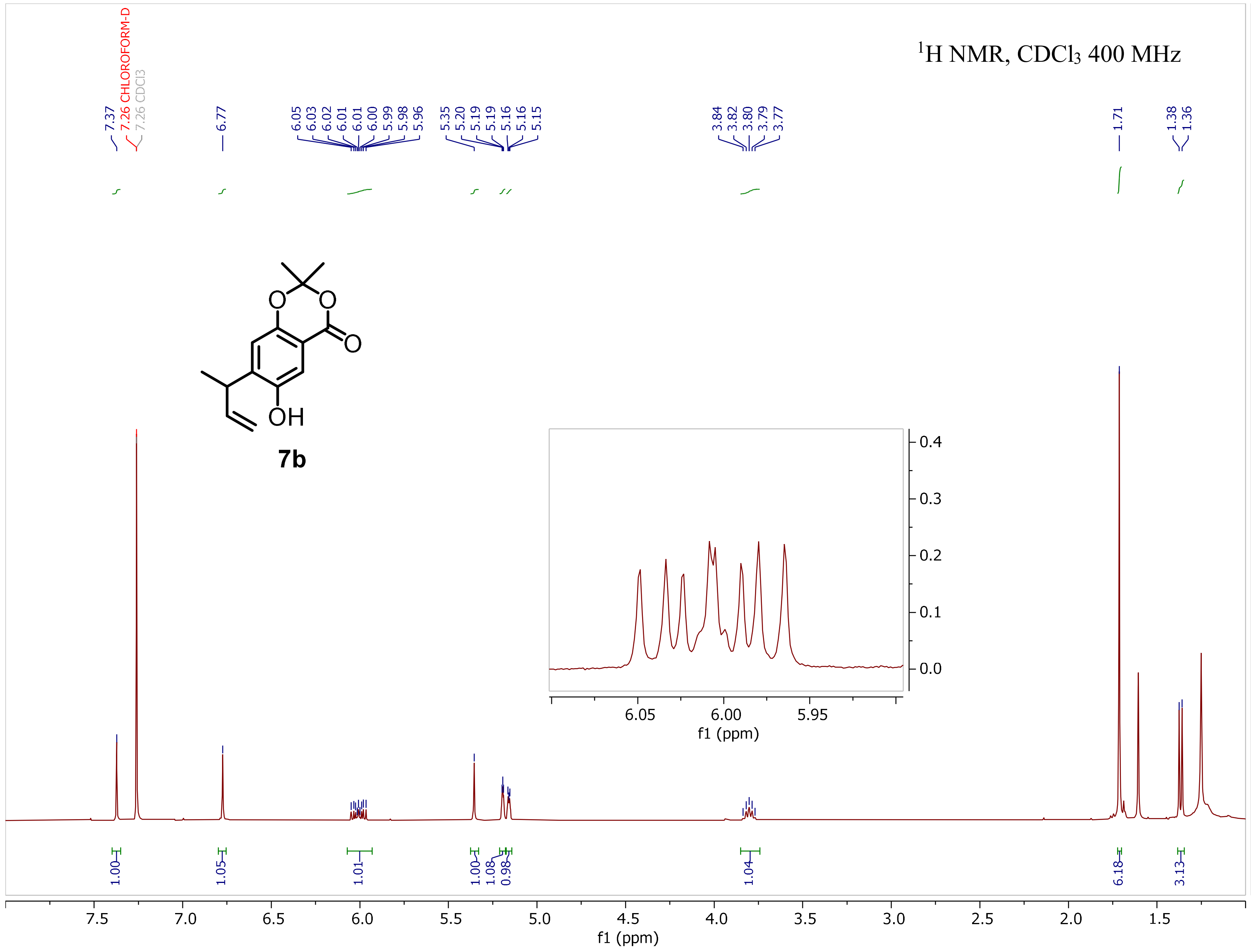

¹H NMR, CDCl₃ 400 MHz
7.37
7.26 CHLOROFORM-D
7.26 CDCl3
6.77
6.05 6.03 6.02 6.01 6.01 6.00 5.99 5.98 5.96
5.35 5.20 5.19 5.19 5.16 5.16 5.15
3.84 3.82 3.80 3.79 3.77
1.71
1.38 1.36
OH
7b
1.00
1.05
1.01
1.00
1.08
0.98
1.04
6.18
3.13
f1 (ppm)

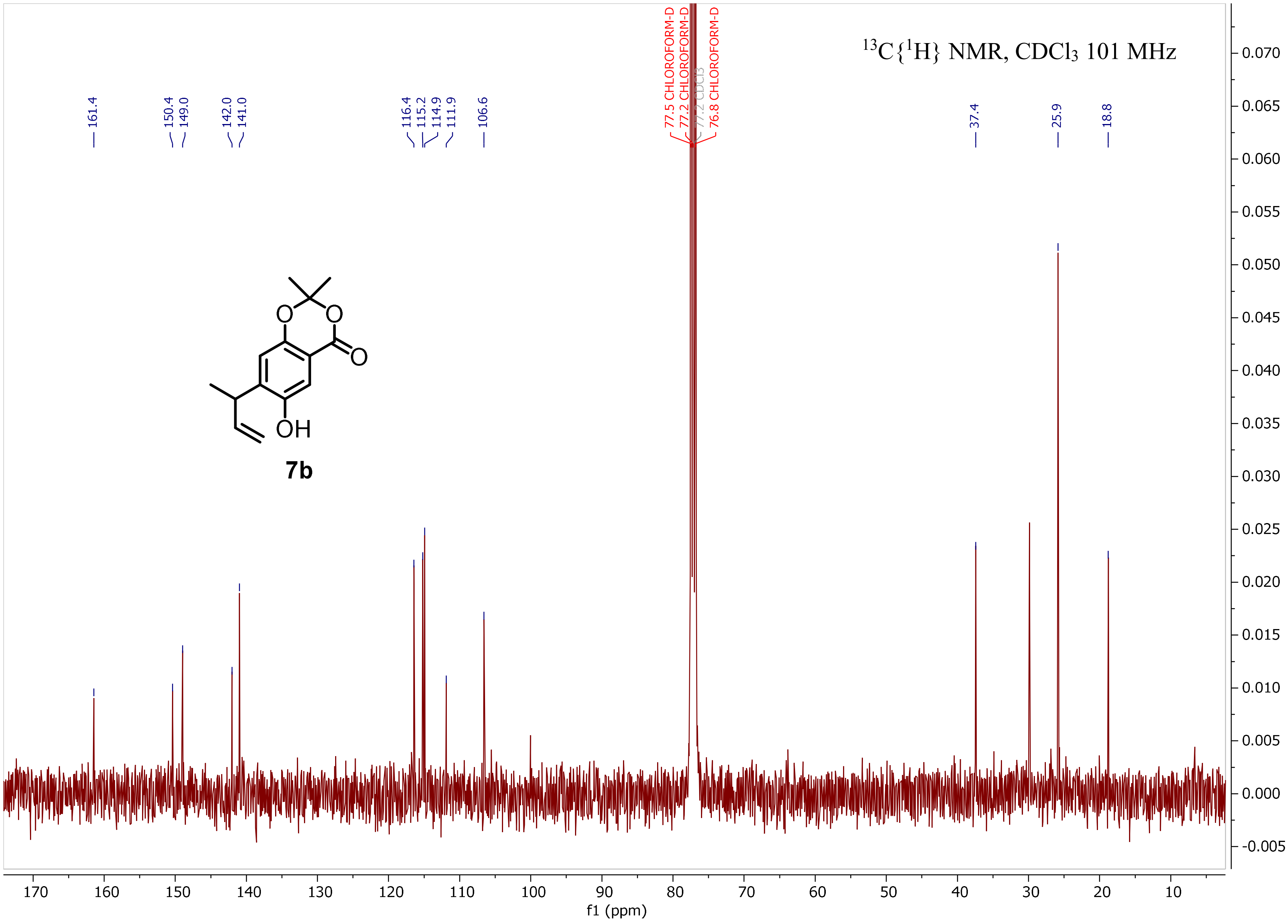
$^{13}C\{^1H\}$ NMR, $CDCl_3$ 101 MHz
161.4
150.4
149.0
142.0
141.0
116.4
115.2
114.9
111.9
106.6
77.5 CHLOROFORM-D
77.2 CHLOROFORM-D
77.2 CDCl3
76.8 CHLOROFORM-D
37.4
25.9
18.8
OH
7b
f1 (ppm)

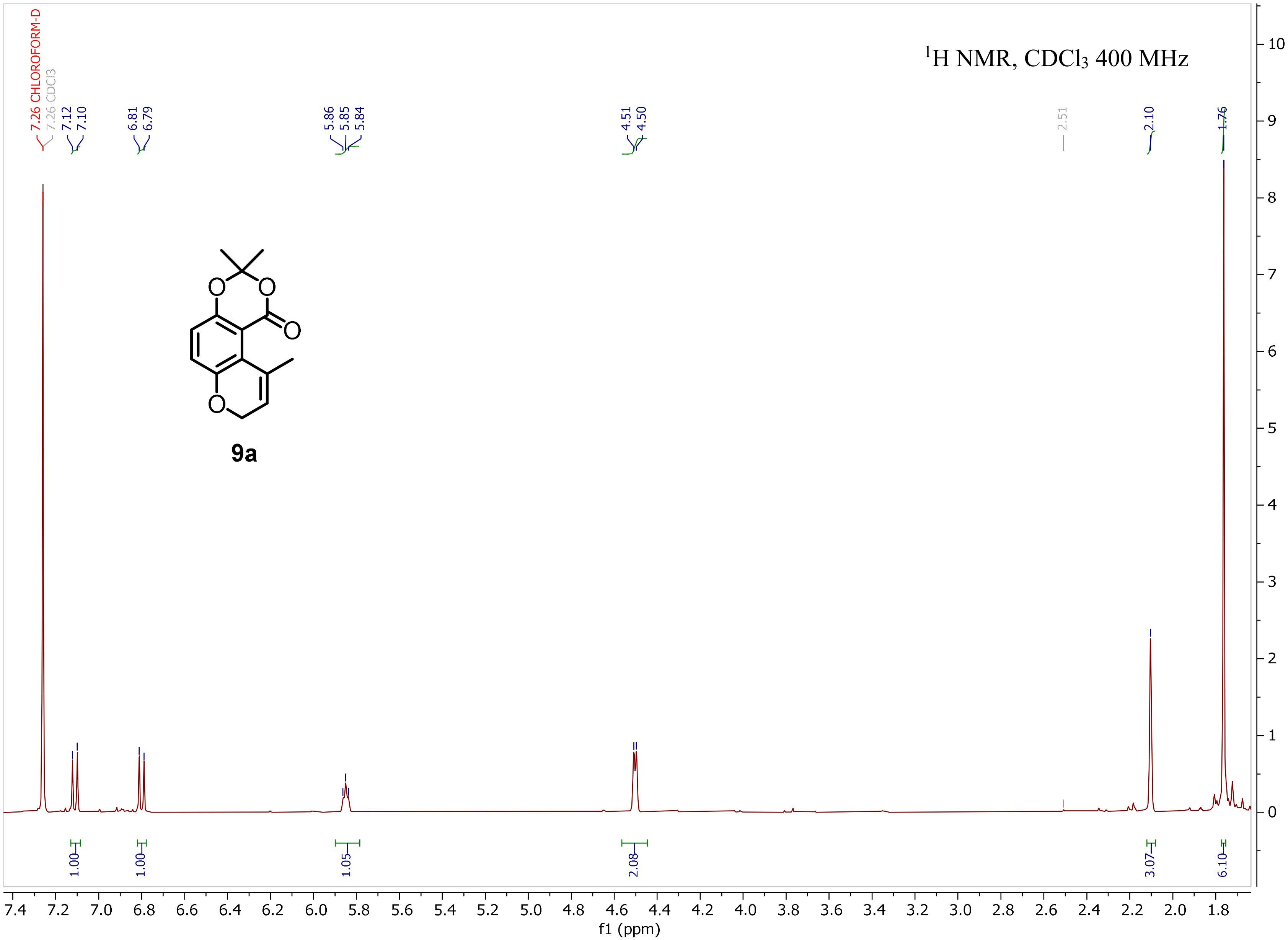

1H NMR, CDCl3 400 MHz
7.26 CHLOROFORM-D
7.26 CDCl3
7.12
7.10
6.81
6.79
5.86
5.85
5.84
4.51
4.50
2.51
2.10
1.76
1.00
1.00
1.05
2.08
3.07
6.10
9a
f1 (ppm)

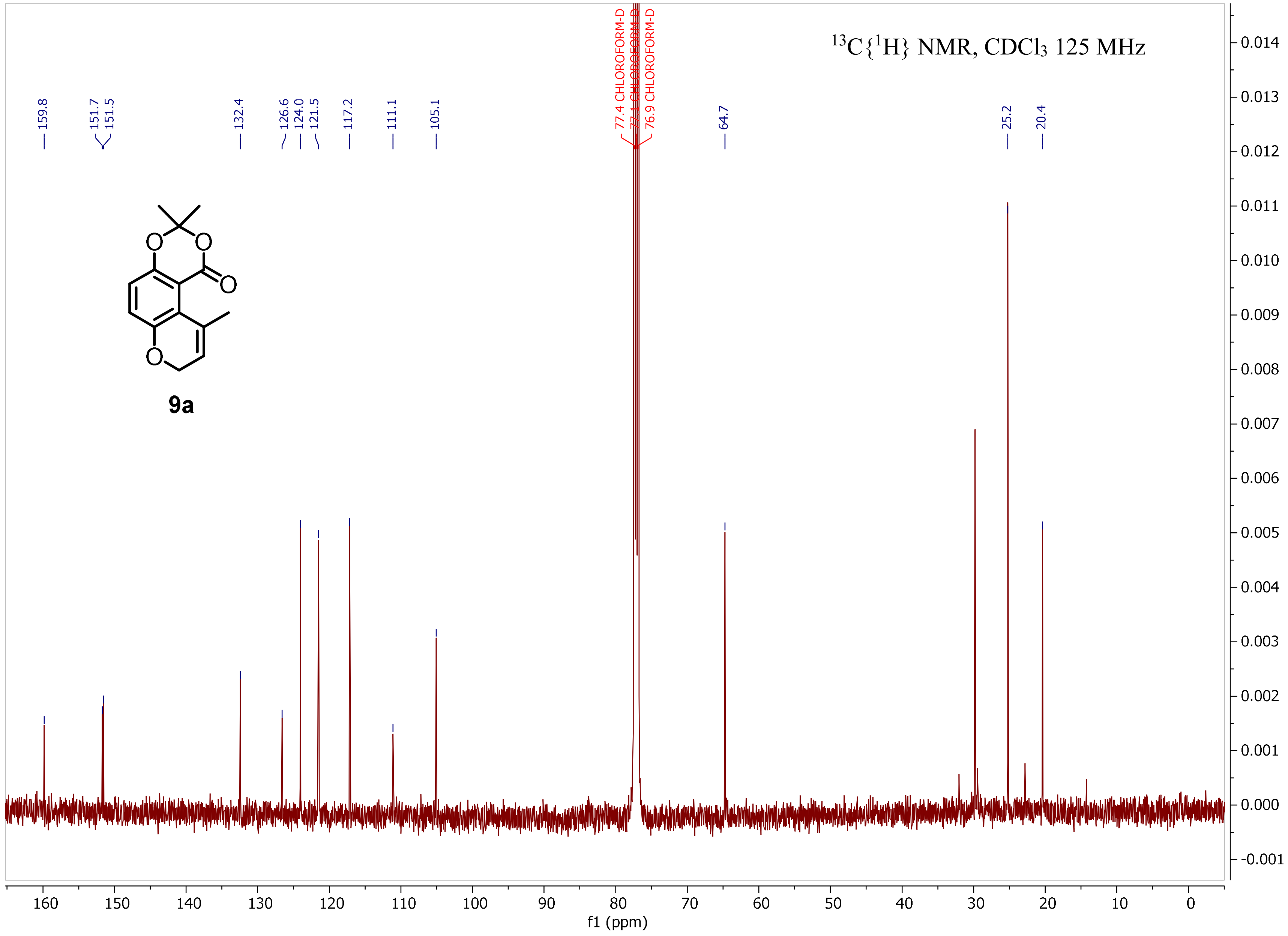

$^{13}C\{^1H\}$ NMR, $CDCl_3$ 125 MHz
159.8
151.7
151.5
132.4
126.6
124.0
121.5
117.2
111.1
105.1
77.4 CHLOROFORM-D
77.1 CHLOROFORM-D
76.9 CHLOROFORM-D
64.7
25.2
20.4
9a
f1 (ppm)

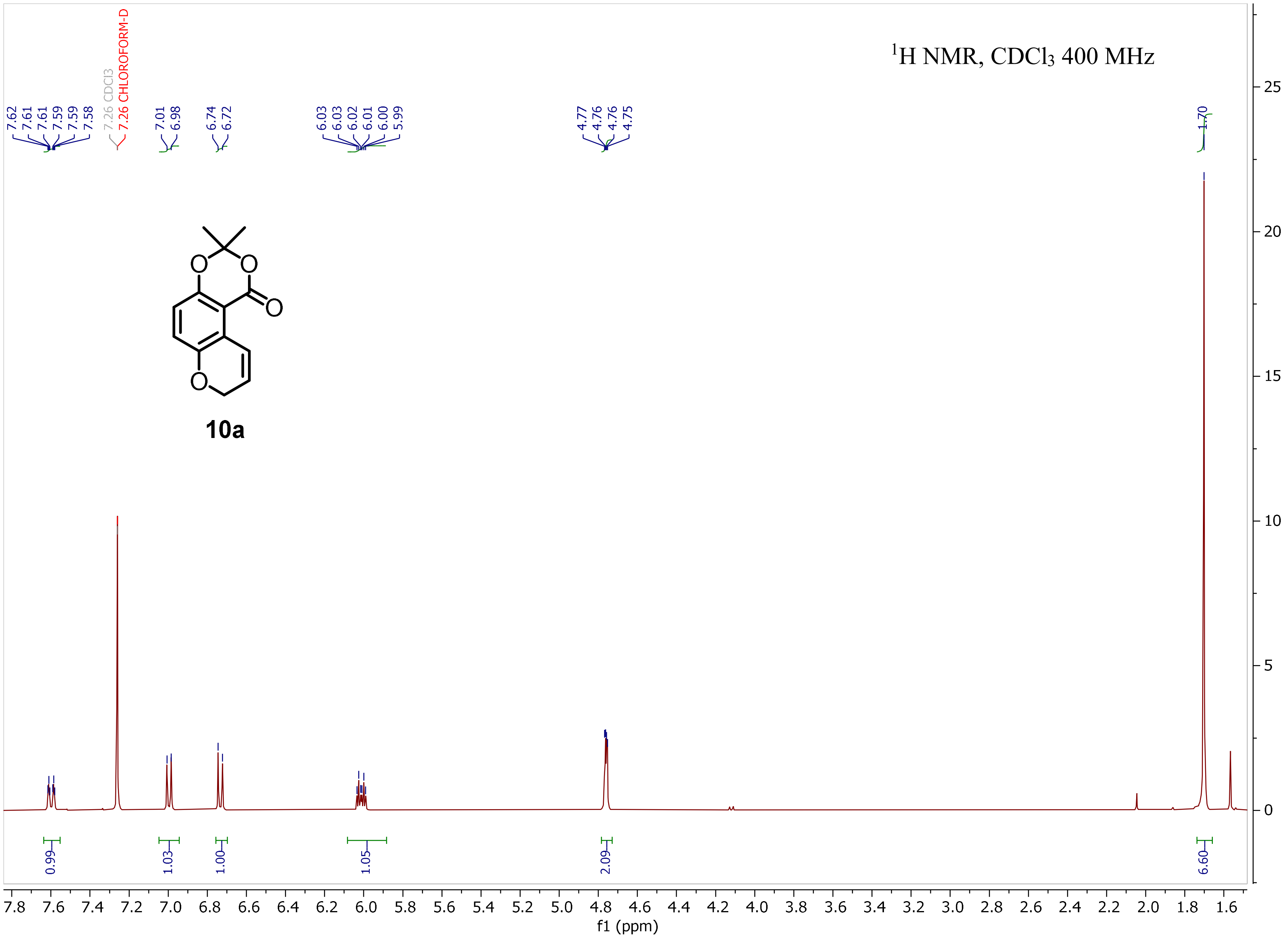

1H NMR, CDCl3 400 MHz
10a
f1 (ppm)

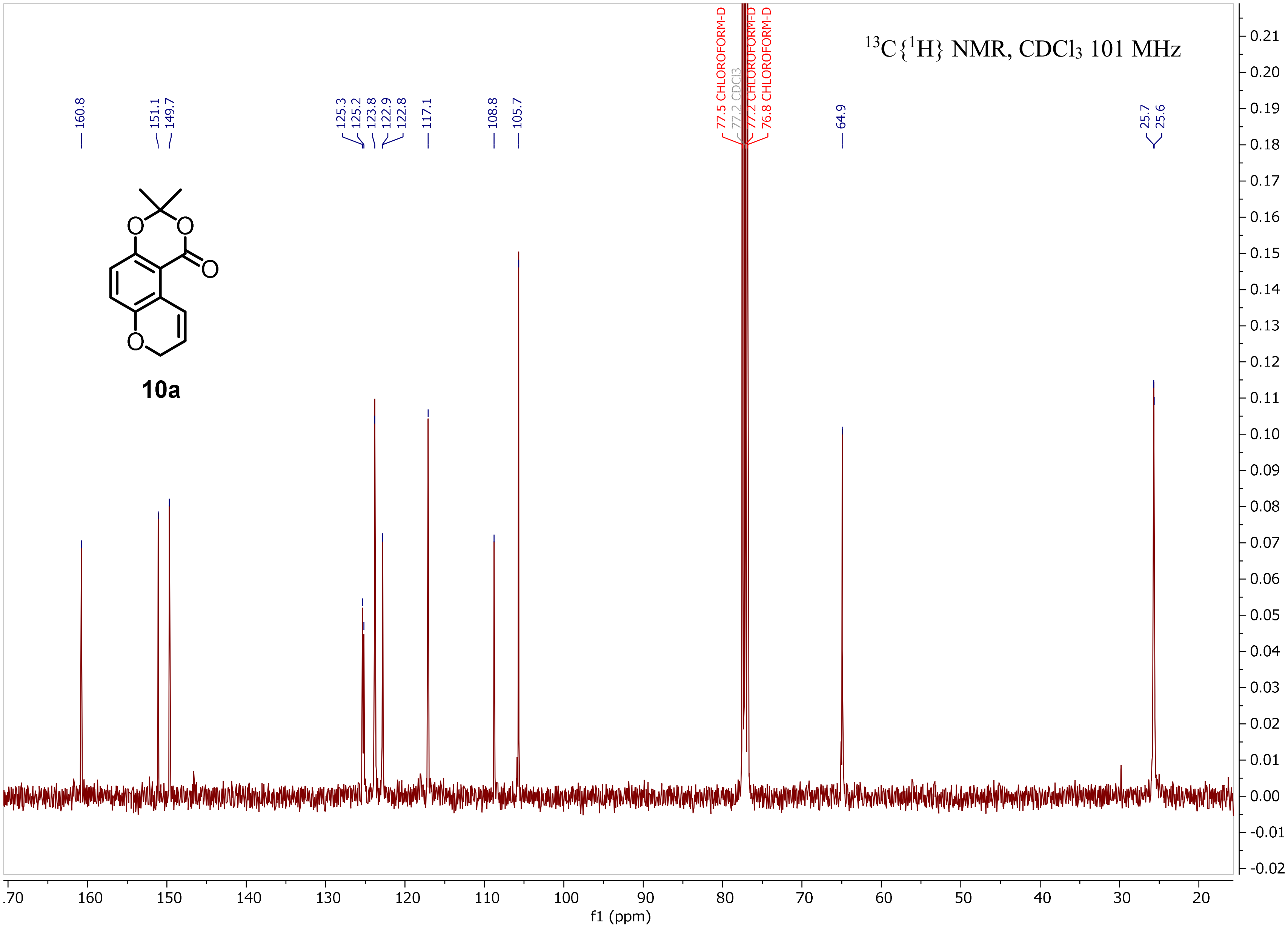
13C{1H} NMR, CDCl3 101 MHz
160.8
151.1
149.7
125.3
125.2
123.8
122.9
122.8
117.1
108.8
105.7
77.5 CHLOROFORM-D
77.2 CDCl3
77.2 CHLOROFORM-D
76.8 CHLOROFORM-D
64.9
25.7
25.6
10a
f1 (ppm)

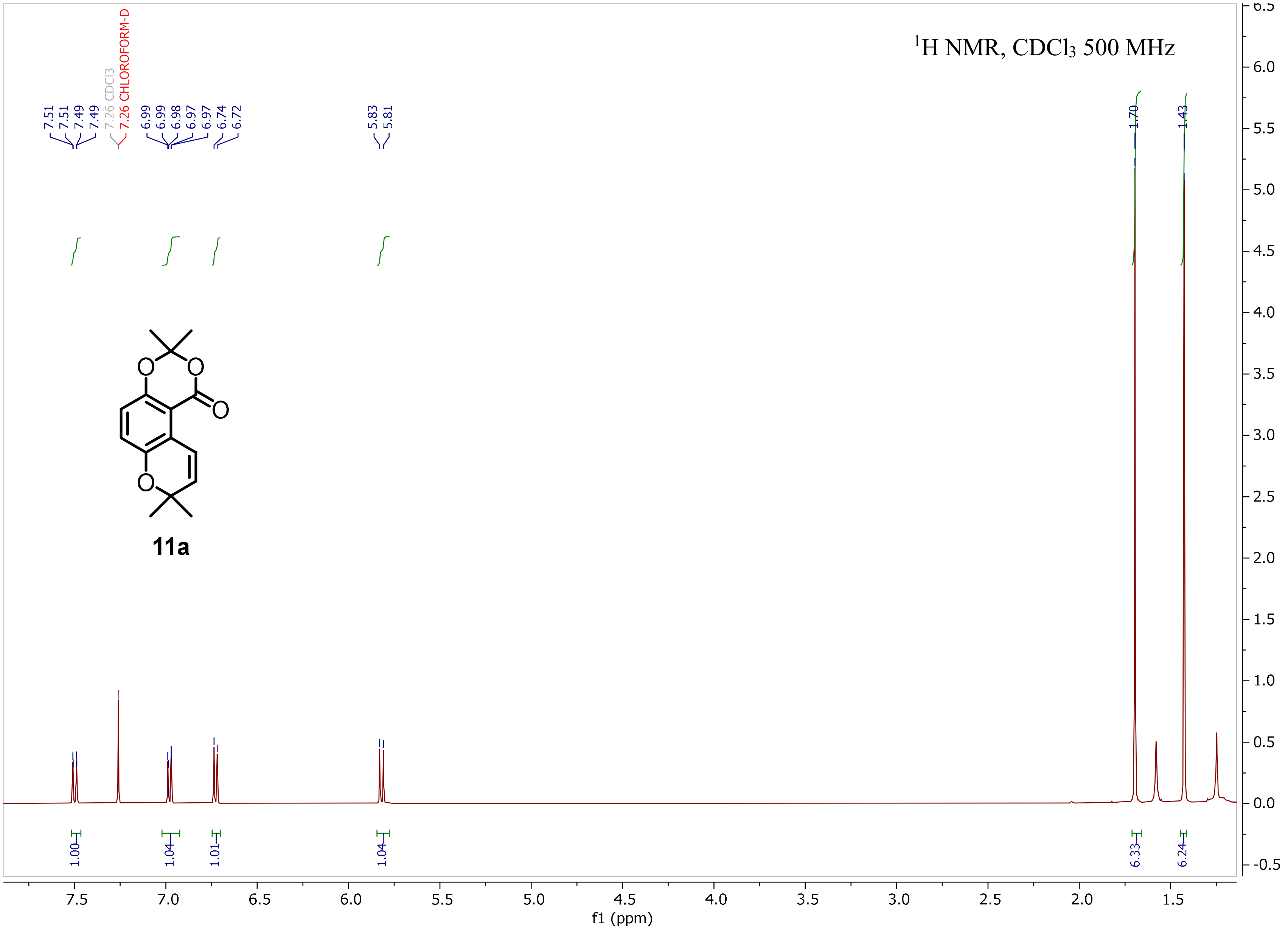

1H NMR, CDCl3 500 MHz
7.51
7.51
7.49
7.49
7.26 CDCl3
7.26 CHLOROFORM-D
6.99
6.99
6.98
6.97
6.97
6.74
6.72
5.83
5.81
1.70
1.43
11a
1.00
1.04
1.01
1.04
6.33
6.24
f1 (ppm)

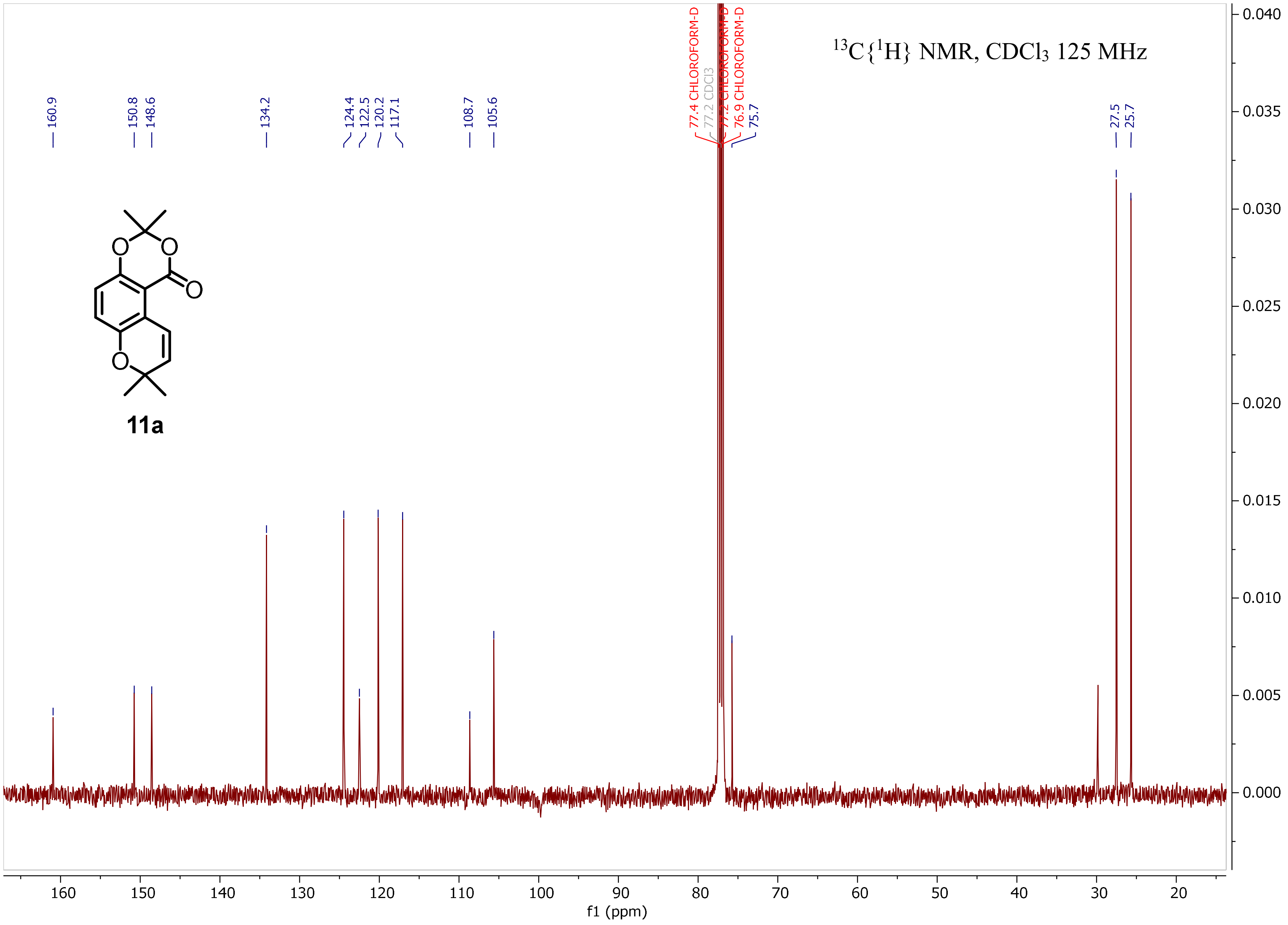

$^{13}C\{^1H\}$ NMR, $CDCl_3$ 125 MHz
160.9
150.8
148.6
134.2
124.4
122.5
120.2
117.1
108.7
105.6
77.4 CHLOROFORM-D
77.2 CDCl3
77.2 CHLOROFORM-D
76.9 CHLOROFORM-D
75.7
27.5
25.7
11a
f1 (ppm)

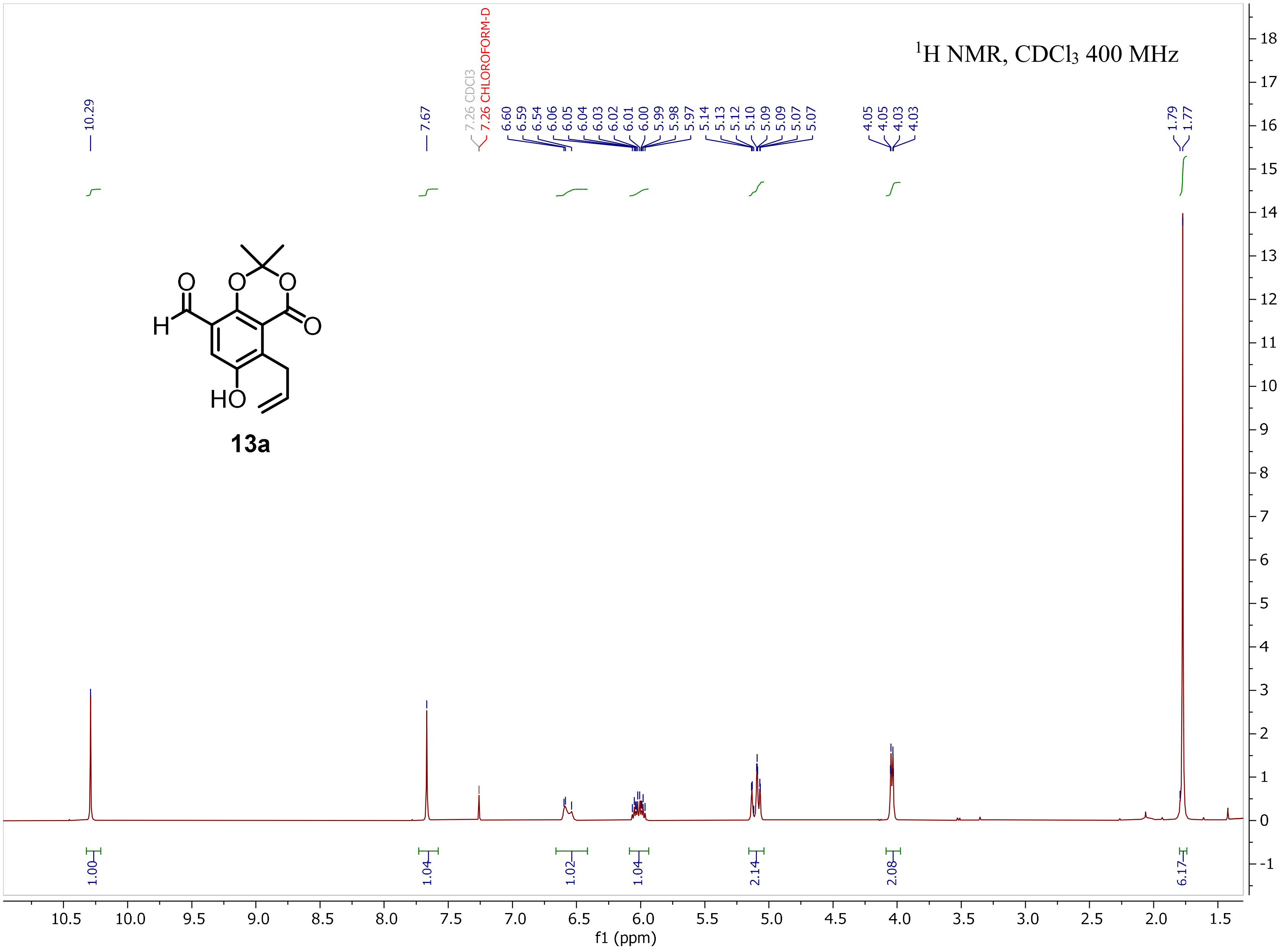

^{1}H NMR, CDCl$_3$ 400 MHz
13a
f1 (ppm)

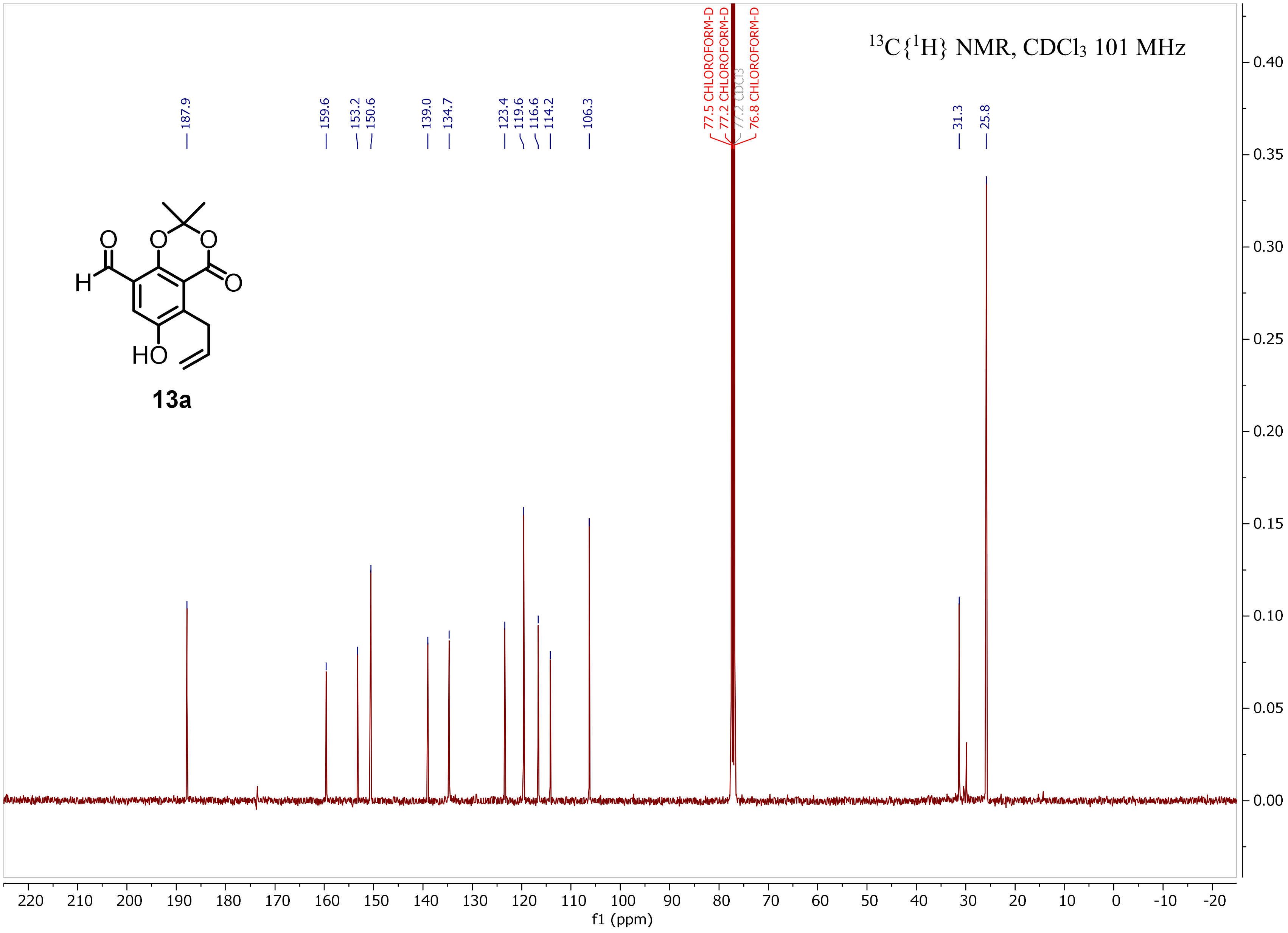

$^{13}C\{^1H\}$ NMR, $CDCl_3$ 101 MHz
187.9
159.6
153.2
150.6
139.0
134.7
123.4
119.6
116.6
114.2
106.3
77.5 CHLOROFORM-D
77.2 CHLOROFORM-D
77.2 CDCl3
76.8 CHLOROFORM-D
31.3
25.8
13a
f1 (ppm)

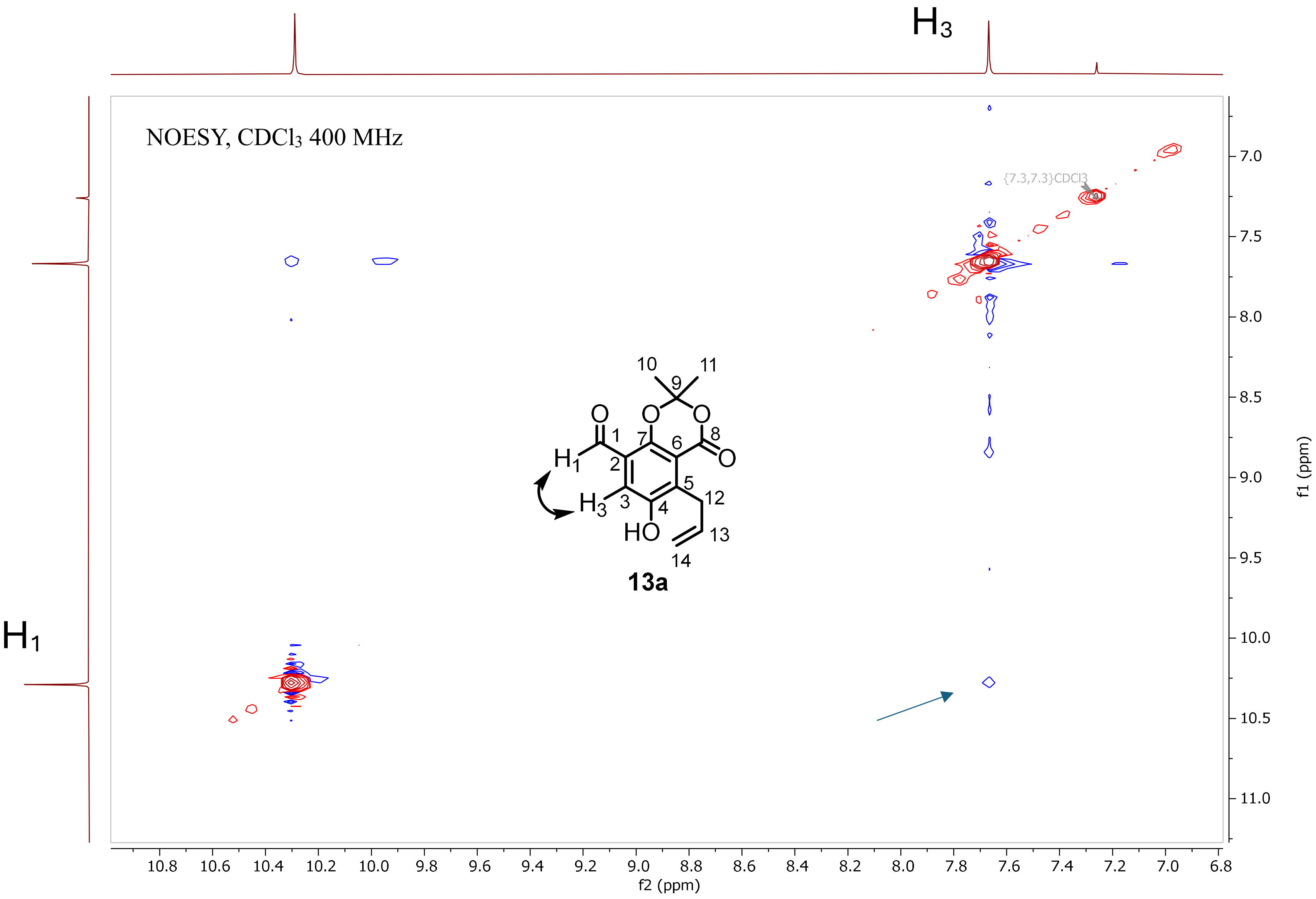
NOESY, CDCl3 400 MHz
H3
H1
{7.3,7.3}CDCl3
10
11
9
O
O
O
O
1
7
6
8
H1
2
5
H3
3
4
12
HO
13
14
13a
f2 (ppm)
f1 (ppm)
10.8
10.6
10.4
10.2
10.0
9.8
9.6
9.4
9.2
9.0
8.8
8.6
8.4
8.2
8.0
7.8
7.6
7.4
7.2
7.0
6.8
7.5
8.0
8.5
9.5
10.5
11.0

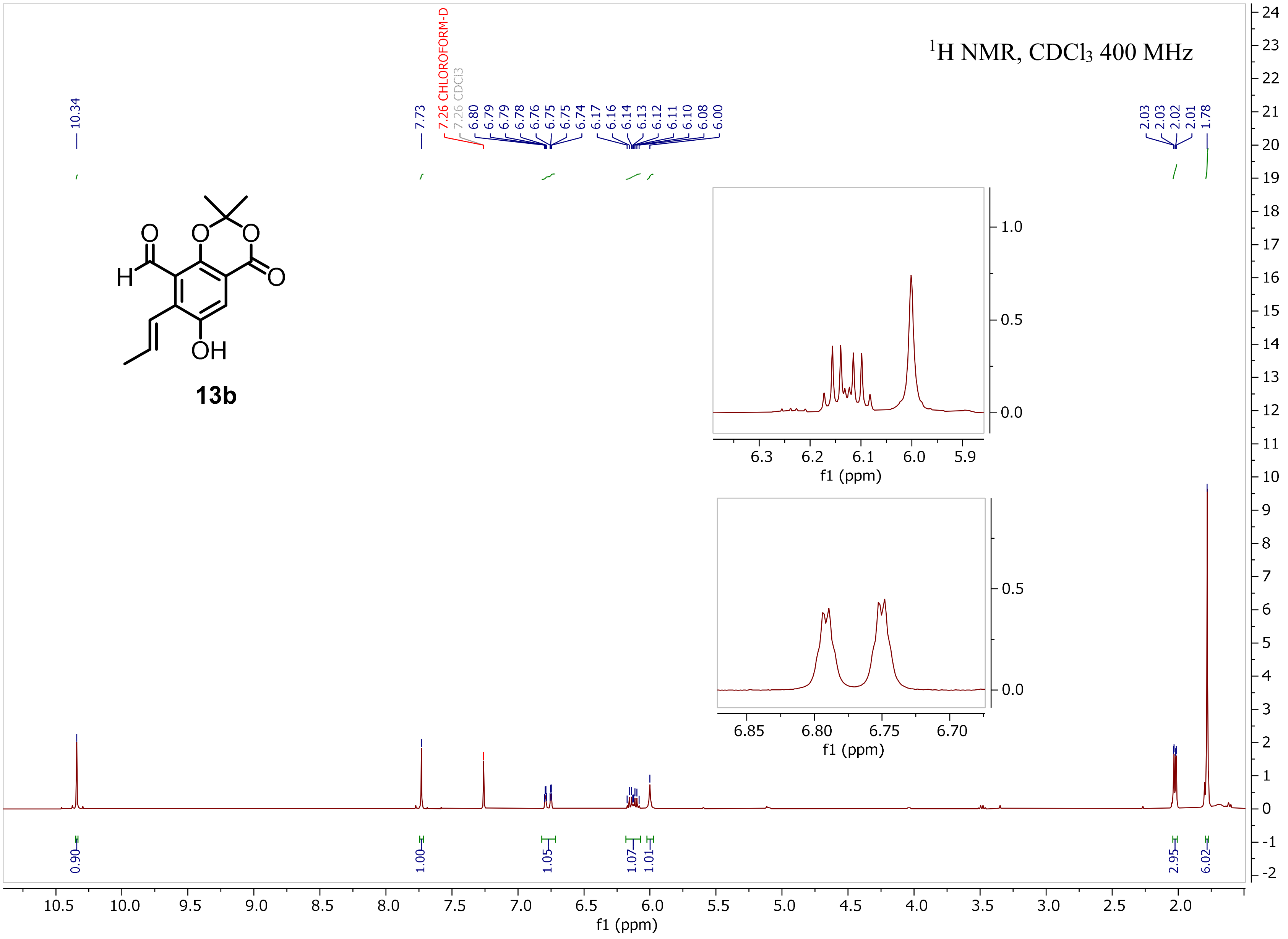

1H NMR, CDCl3 400 MHz
13b
10.34
7.73
7.26 CHLOROFORM-D
7.26 CDCl3
6.80
6.79
6.79
6.78
6.76
6.75
6.75
6.74
6.17
6.16
6.14
6.13
6.12
6.11
6.10
6.08
6.00
2.03
2.03
2.02
2.01
1.78
0.90
1.00
1.05
1.07
1.01
2.95
6.02
f1 (ppm)

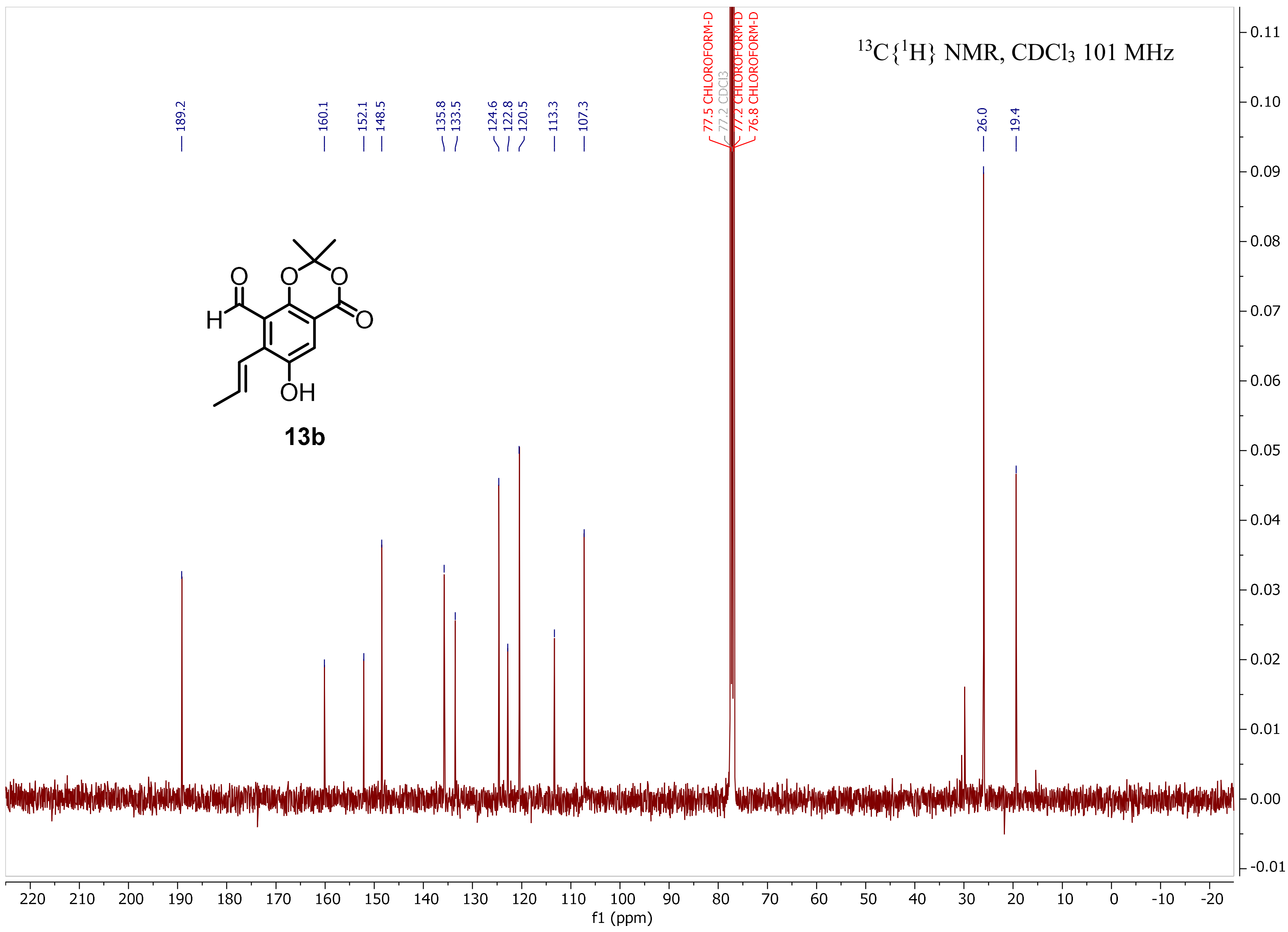

$^{13}C\{^1H\}$ NMR, $CDCl_3$ 101 MHz
189.2
160.1
152.1
148.5
135.8
133.5
124.6
122.8
120.5
113.3
107.3
77.5 CHLOROFORM-D
77.2 CDCl3
77.2 CHLOROFORM-D
76.8 CHLOROFORM-D
26.0
19.4
13b
f1 (ppm)

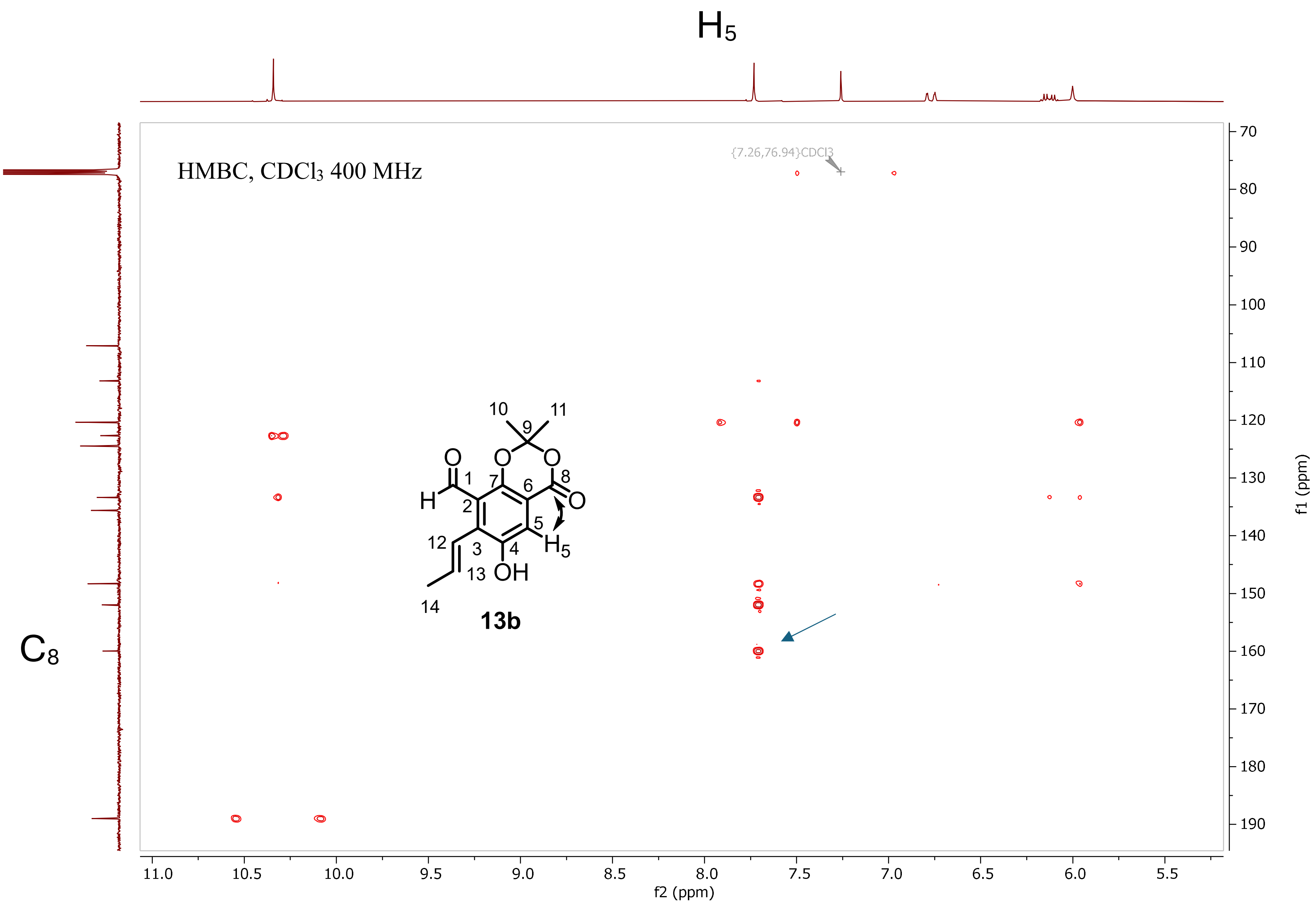
H5
HMBC, CDCl3 400 MHz
{7.26,76.94}CDCl3
C8
10
11
9
O
O
O
1
7
6
8
O
H
2
5
H5
12
3
4
13
OH
14
13b
11.0
10.5
10.0
9.5
9.0
8.5
8.0
7.5
7.0
6.5
6.0
5.5
f2 (ppm)
70
80
90
100
110
120
130
140
150
160
170
180
190
f1 (ppm)

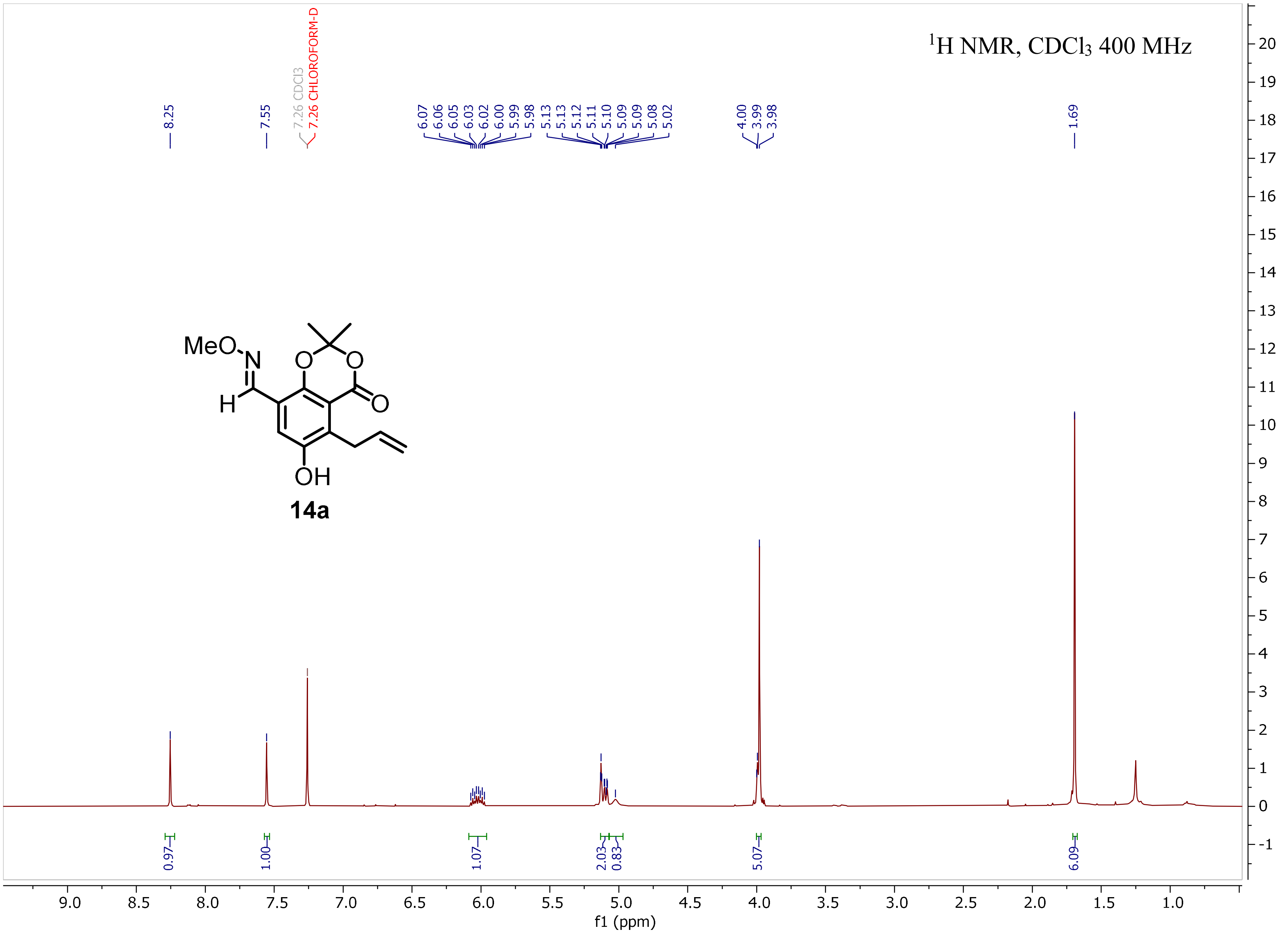

¹H NMR, CDCl3 400 MHz
8.25
7.55
7.26 CDCl3
7.26 CHLOROFORM-D
6.07
6.06
6.05
6.03
6.02
6.00
5.99
5.98
5.13
5.13
5.12
5.11
5.10
5.09
5.09
5.08
5.02
4.00
3.99
3.98
1.69
MeO
N
H
O
O
O
OH
14a
0.97
1.00
1.07
2.03
0.83
5.07
6.09
9.0
8.5
8.0
7.5
7.0
6.5
6.0
5.5
5.0
4.5
4.0
3.5
3.0
2.5
2.0
1.5
1.0
f1 (ppm)

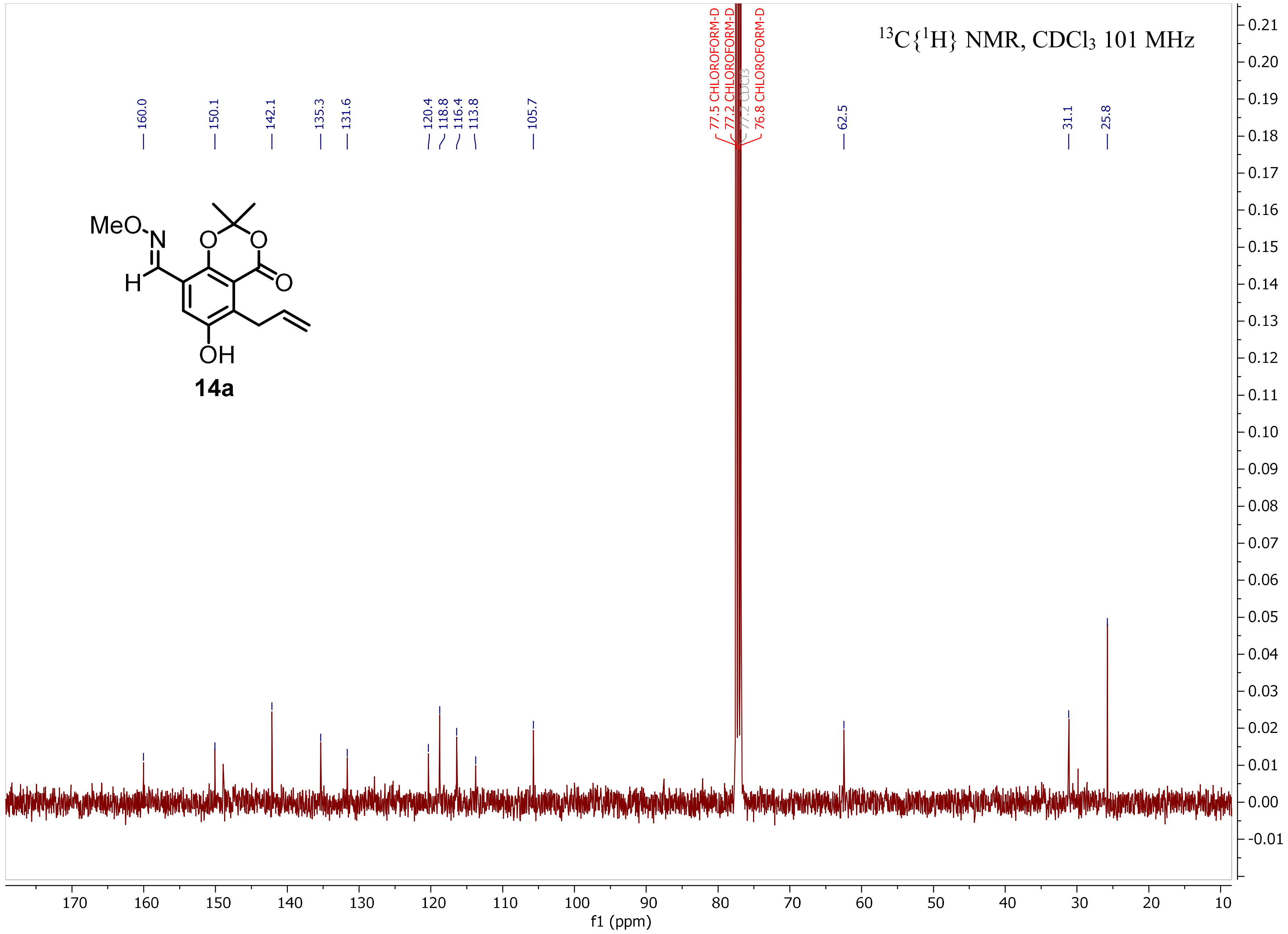

^{13}C{^{1}H} NMR, CDCl$_3$ 101 MHz
160.0
150.1
142.1
135.3
131.6
120.4
118.8
116.4
113.8
105.7
77.5 CHLOROFORM-D
77.2 CHLOROFORM-D
77.2 CDCl3
76.8 CHLOROFORM-D
62.5
31.1
25.8
MeO
N
H
O
O
O
OH
14a
f1 (ppm)

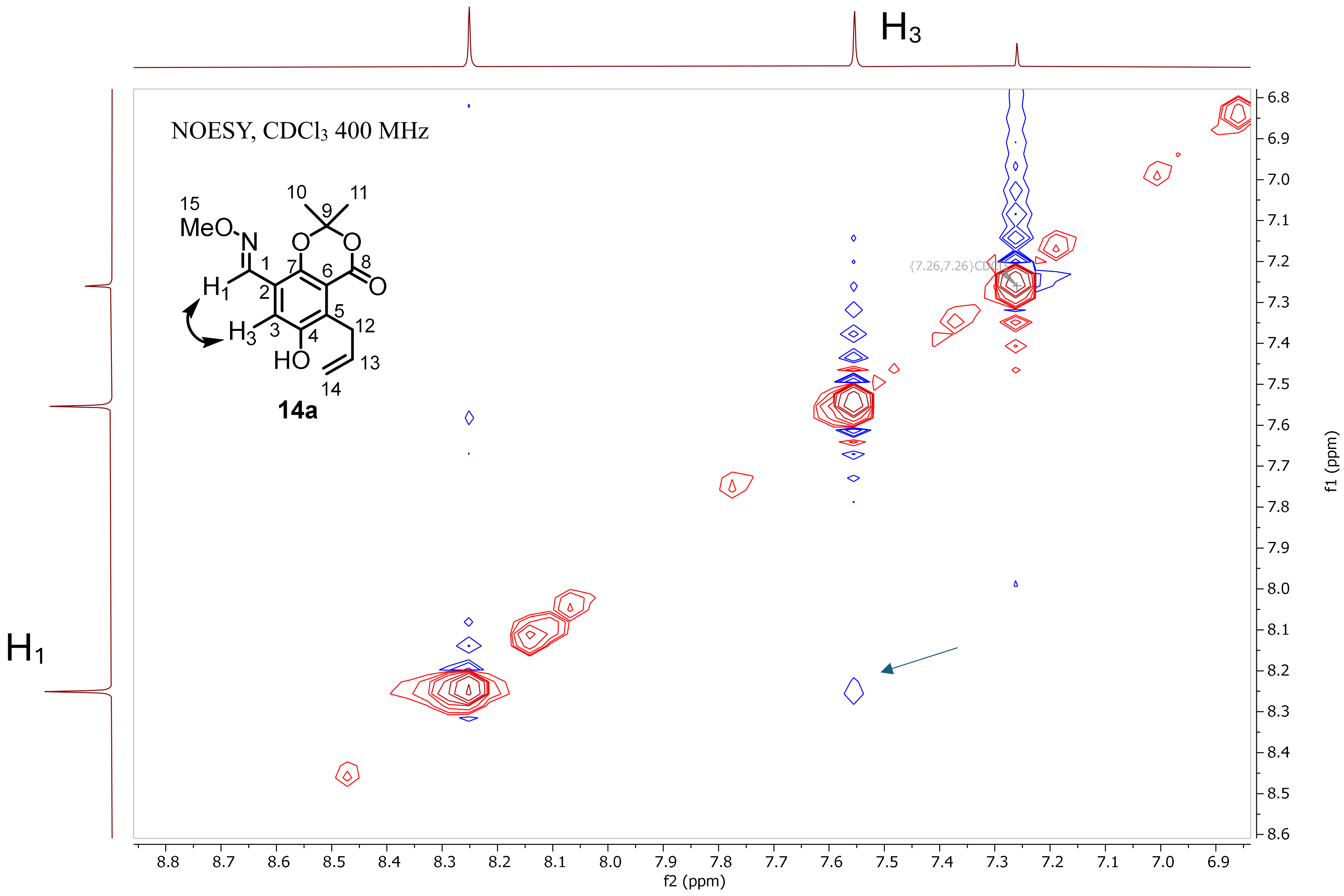

NOESY, CDCl3 400 MHz
H3
H1
14a
{7.26,7.26}CDCl3
f2 (ppm)
f1 (ppm)

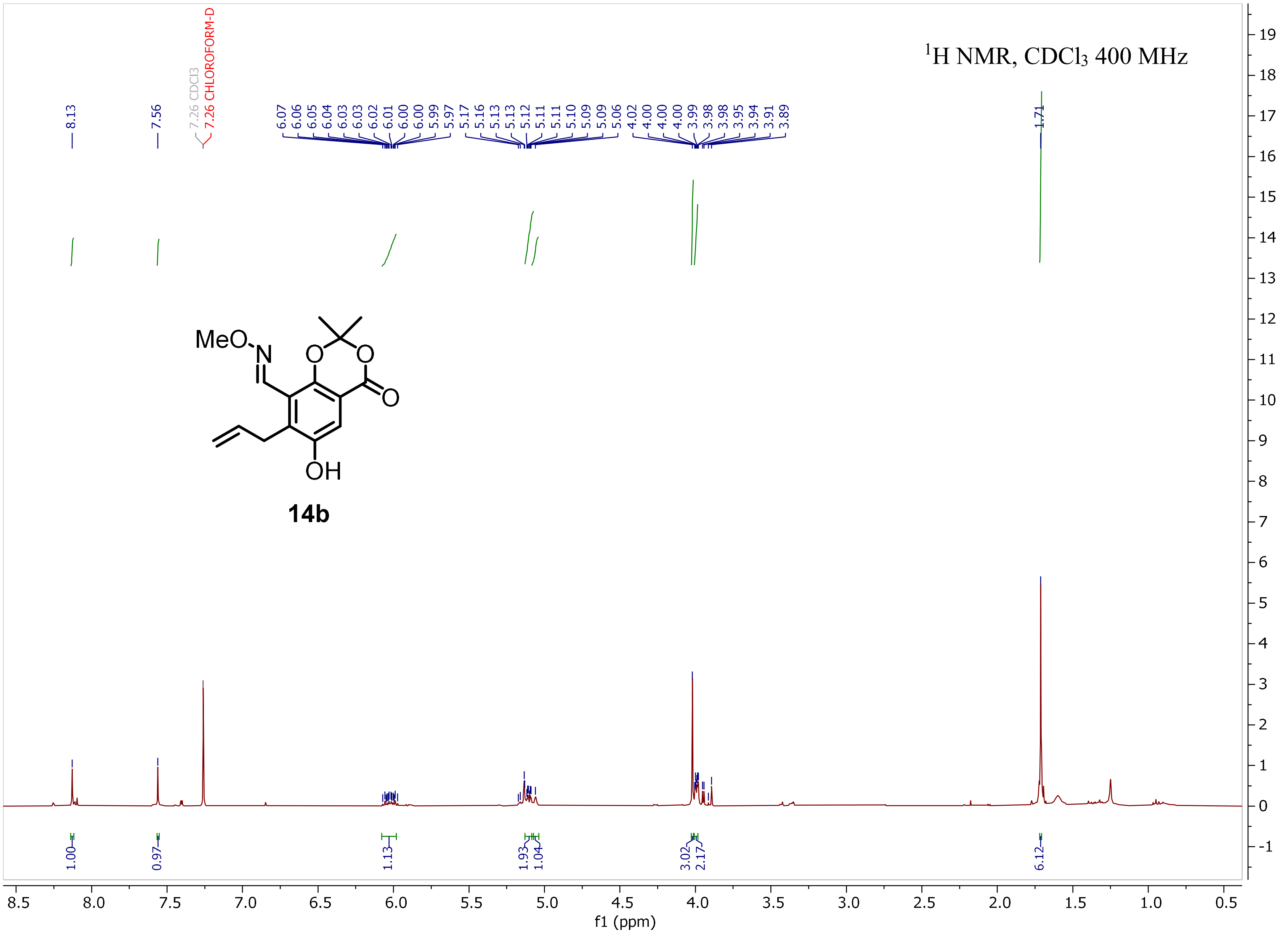

1H NMR, CDCl3 400 MHz
14b
f1 (ppm)

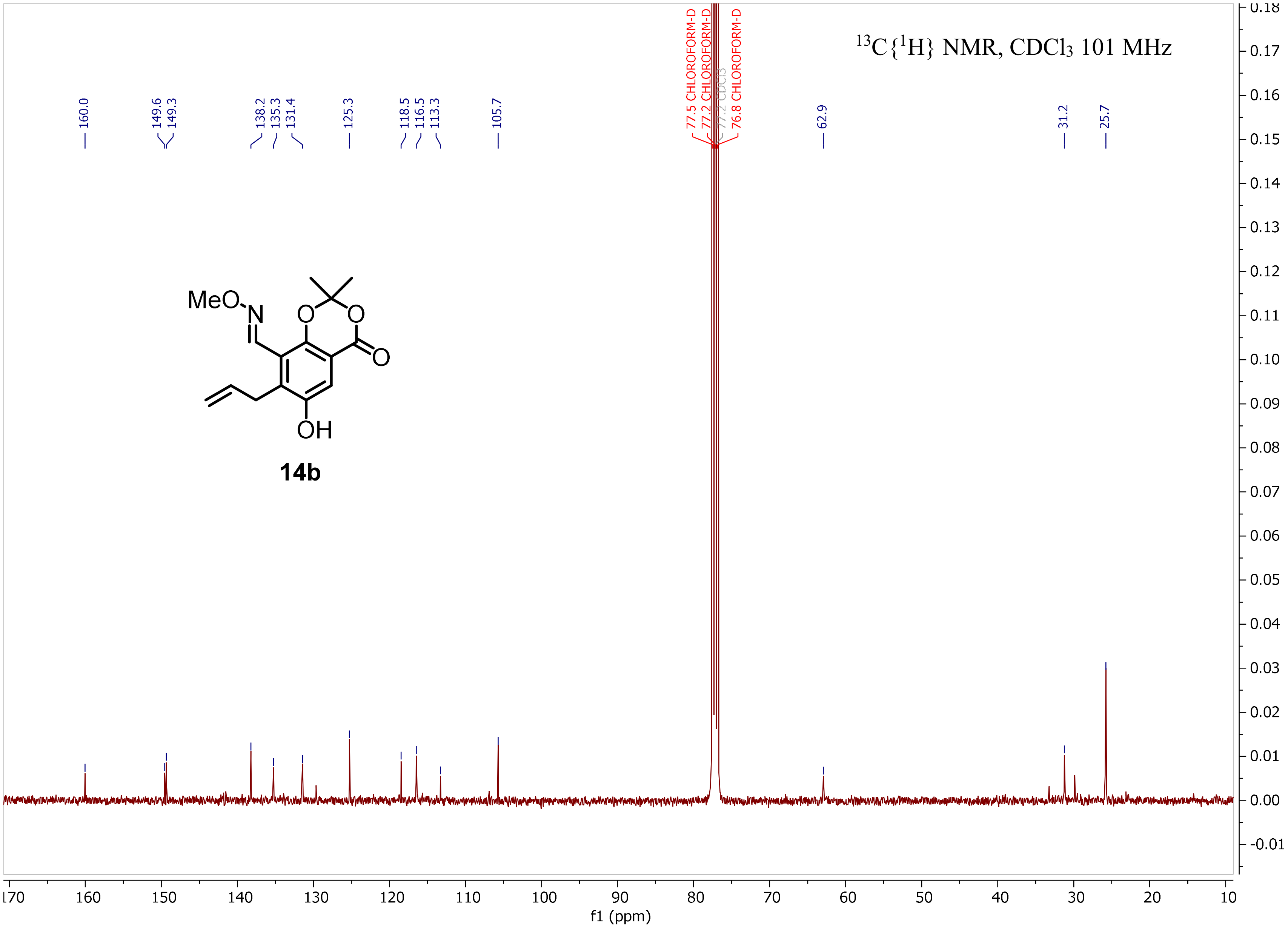
13C{1H} NMR, CDCl3 101 MHz
160.0
149.6
149.3
138.2
135.3
131.4
125.3
118.5
116.5
113.3
105.7
77.5 CHLOROFORM-D
77.2 CHLOROFORM-D
77.2 CDCl3
76.8 CHLOROFORM-D
62.9
31.2
25.7
MeO
N
O
O
O
OH
14b
f1 (ppm)

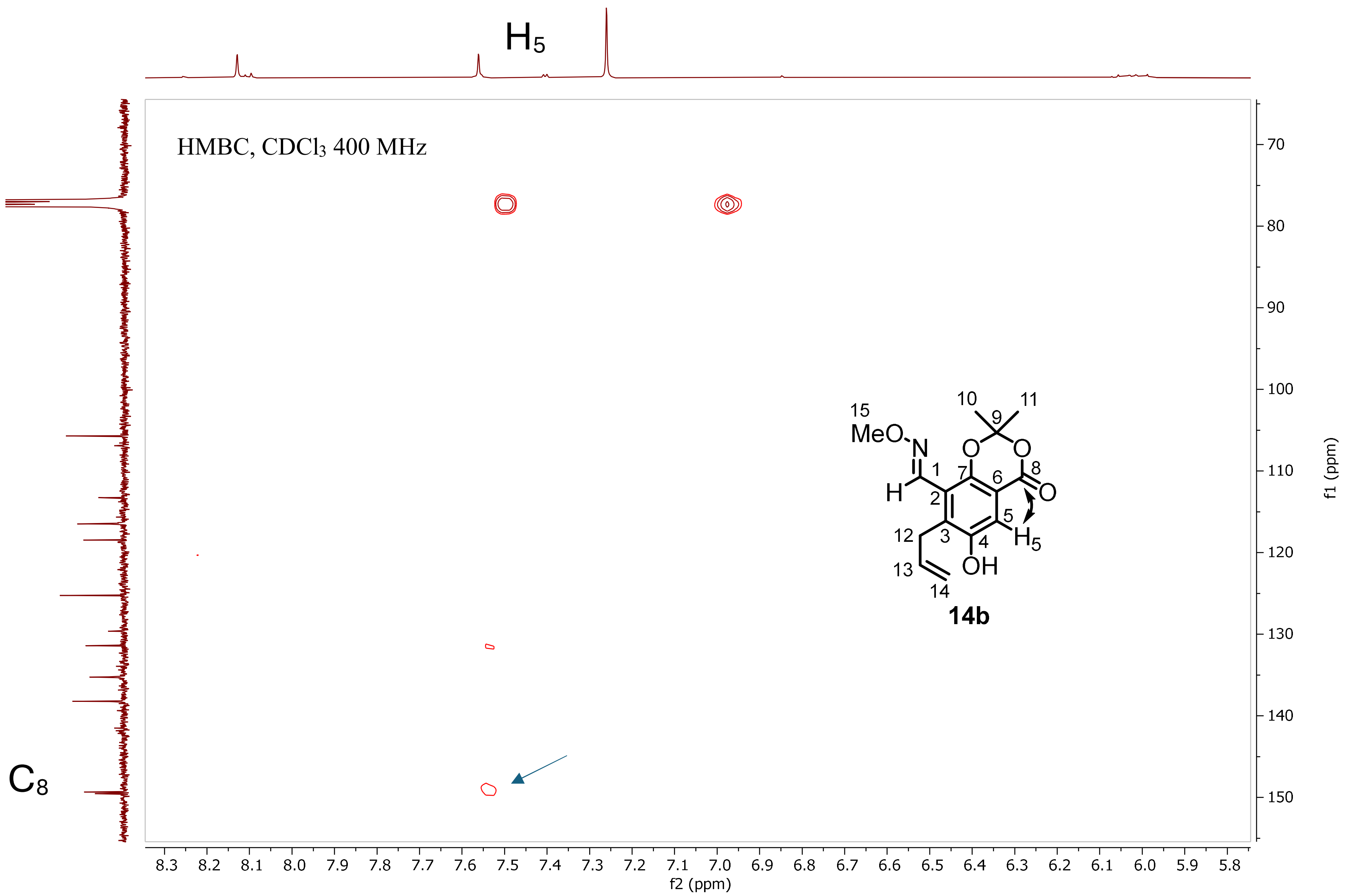
H5
HMBC, CDCl3 400 MHz
C8
15
MeO
N
10
9
11
O
O
8
O
7
6
1
H
2
5
H5
12
3
4
OH
13
14
14b
f1 (ppm)
f2 (ppm)

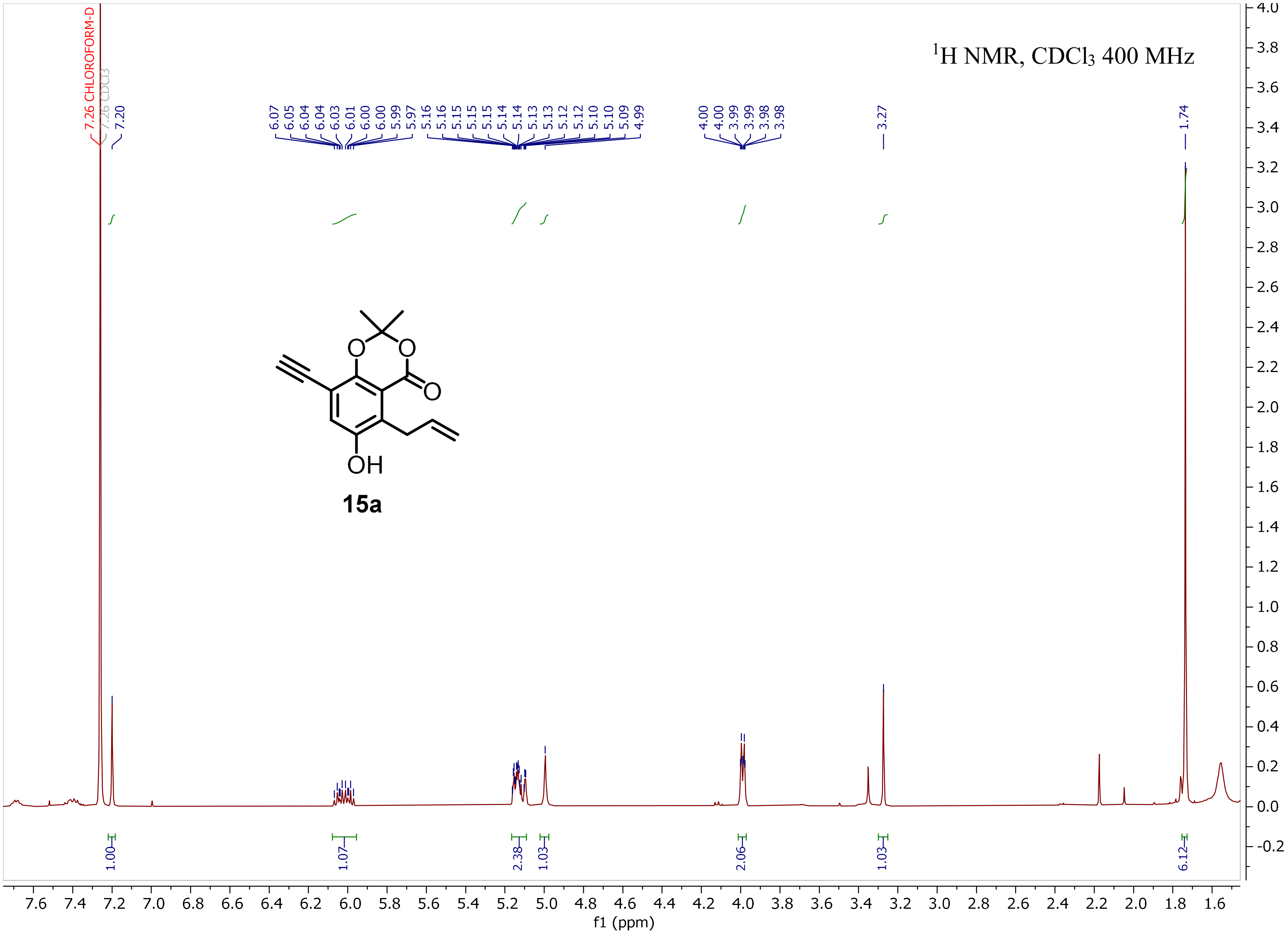

1H NMR, CDCl3 400 MHz
7.26 CHLOROFORM-D
7.26 CDCl3
7.20
6.07 6.05 6.04 6.04 6.03 6.01 6.00 6.00 5.99 5.97
5.16 5.16 5.15 5.15 5.15 5.14 5.14 5.13 5.13 5.12 5.12 5.10 5.10 5.09 4.99
4.00 4.00 3.99 3.99 3.98 3.98
3.27
1.74
1.00 1.07 2.38 1.03 2.06 1.03 6.12
OH
15a
f1 (ppm)

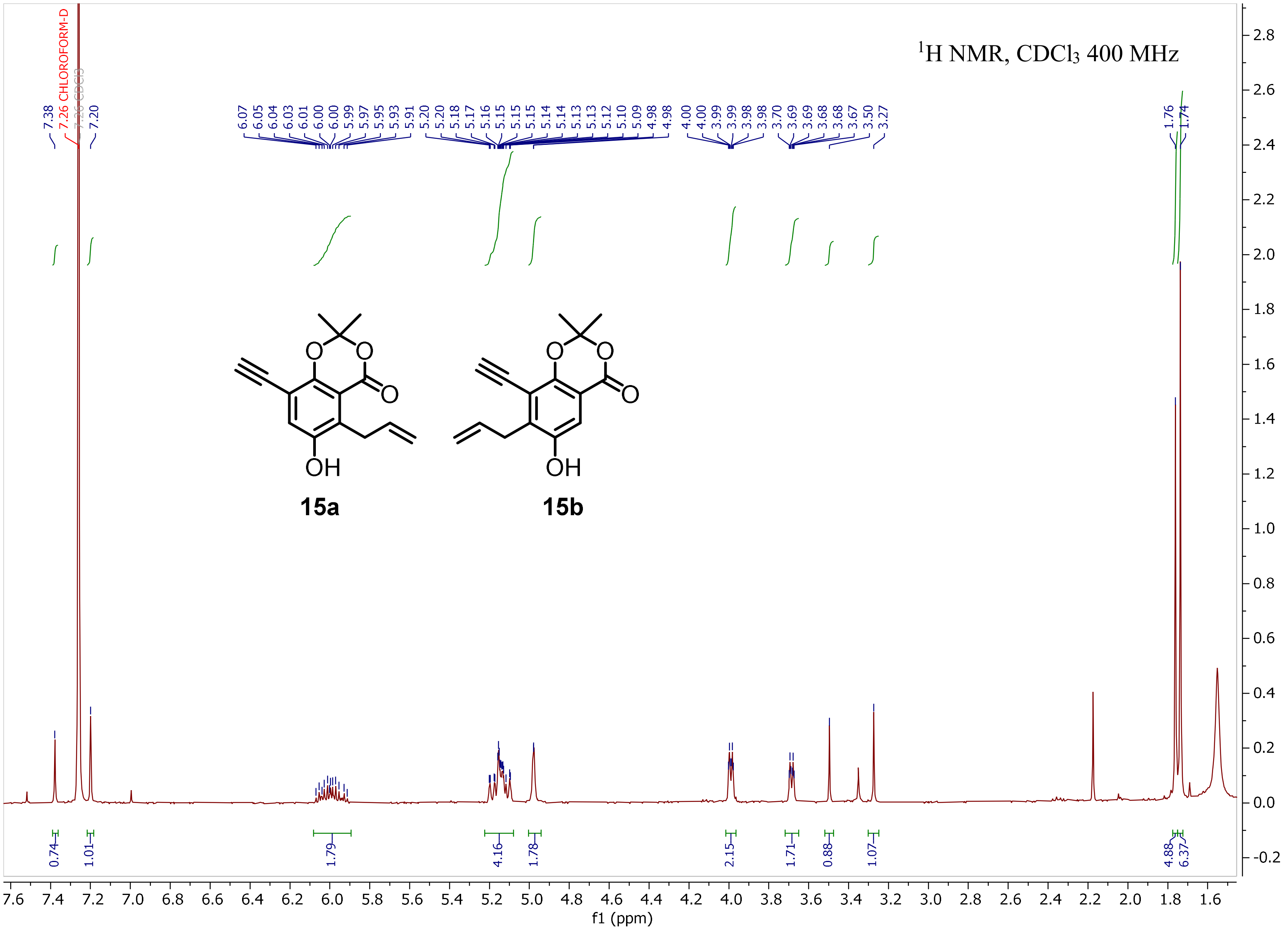

1H NMR, CDCl3 400 MHz
7.26 CHLOROFORM-D
15a
15b
f1 (ppm)

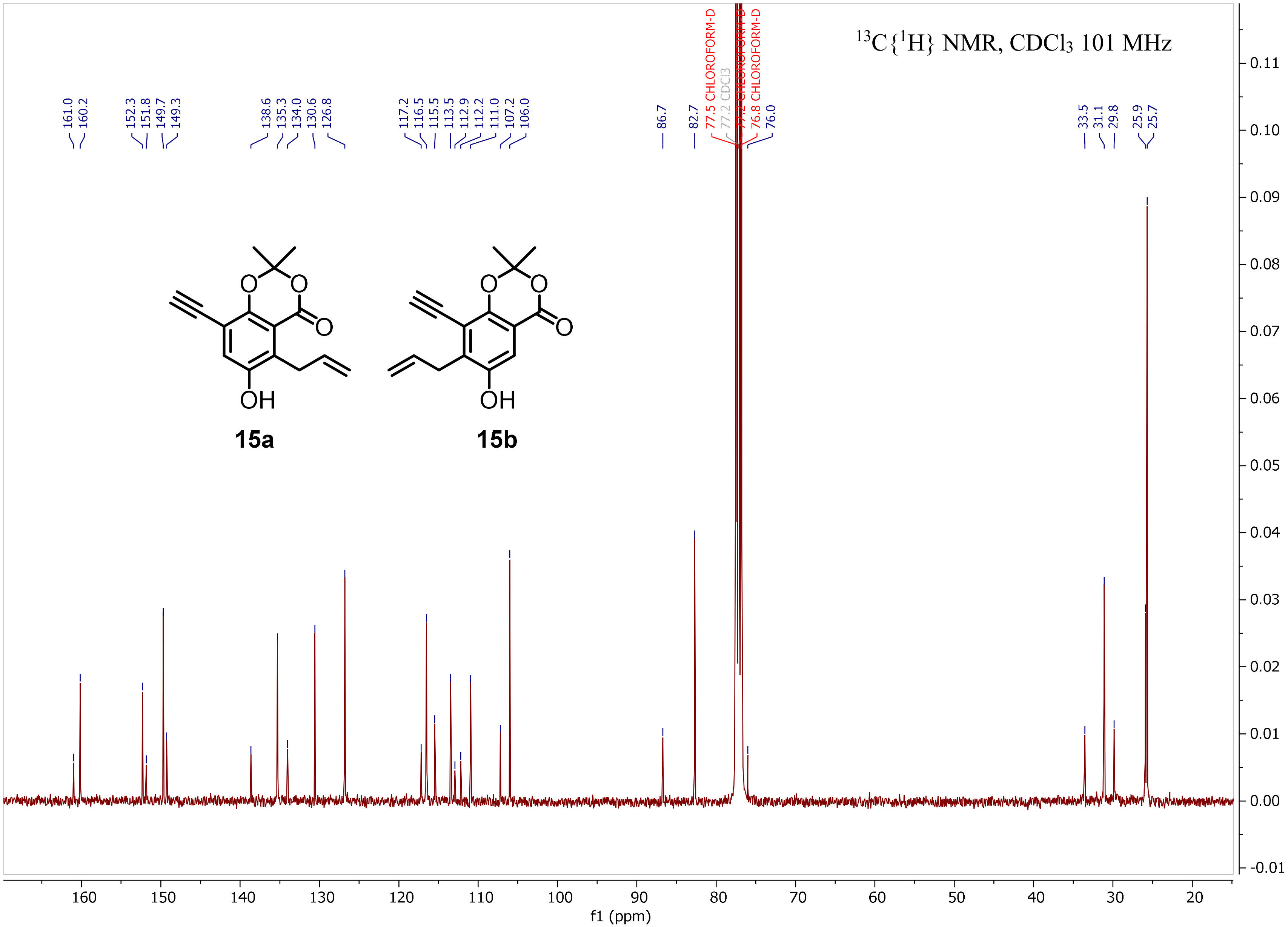
13C{1H} NMR, CDCl3 101 MHz
161.0
160.2
152.3
151.8
149.7
149.3
138.6
135.3
134.0
130.6
126.8
117.2
116.5
115.5
113.5
112.9
112.2
111.0
107.2
106.0
86.7
82.7
77.5 CHLOROFORM-D
77.2 CDCl3
77.2 CHLOROFORM-D
76.8 CHLOROFORM-D
76.0
33.5
31.1
29.8
25.9
25.7
O
O
O
OH
15a
15b
f1 (ppm)

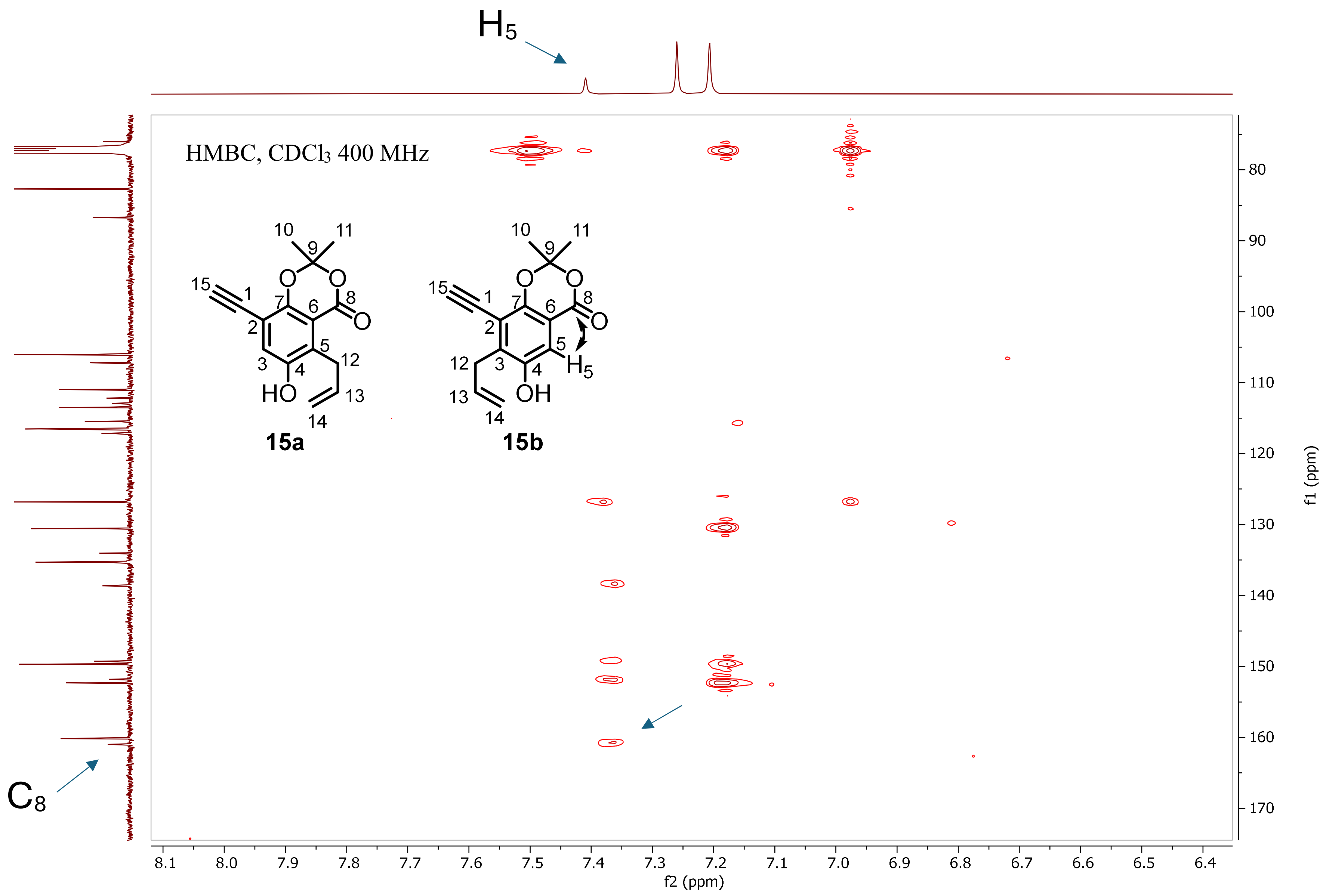
H5
HMBC, CDCl3 400 MHz
15a
15b
C8
f2 (ppm)
f1 (ppm)

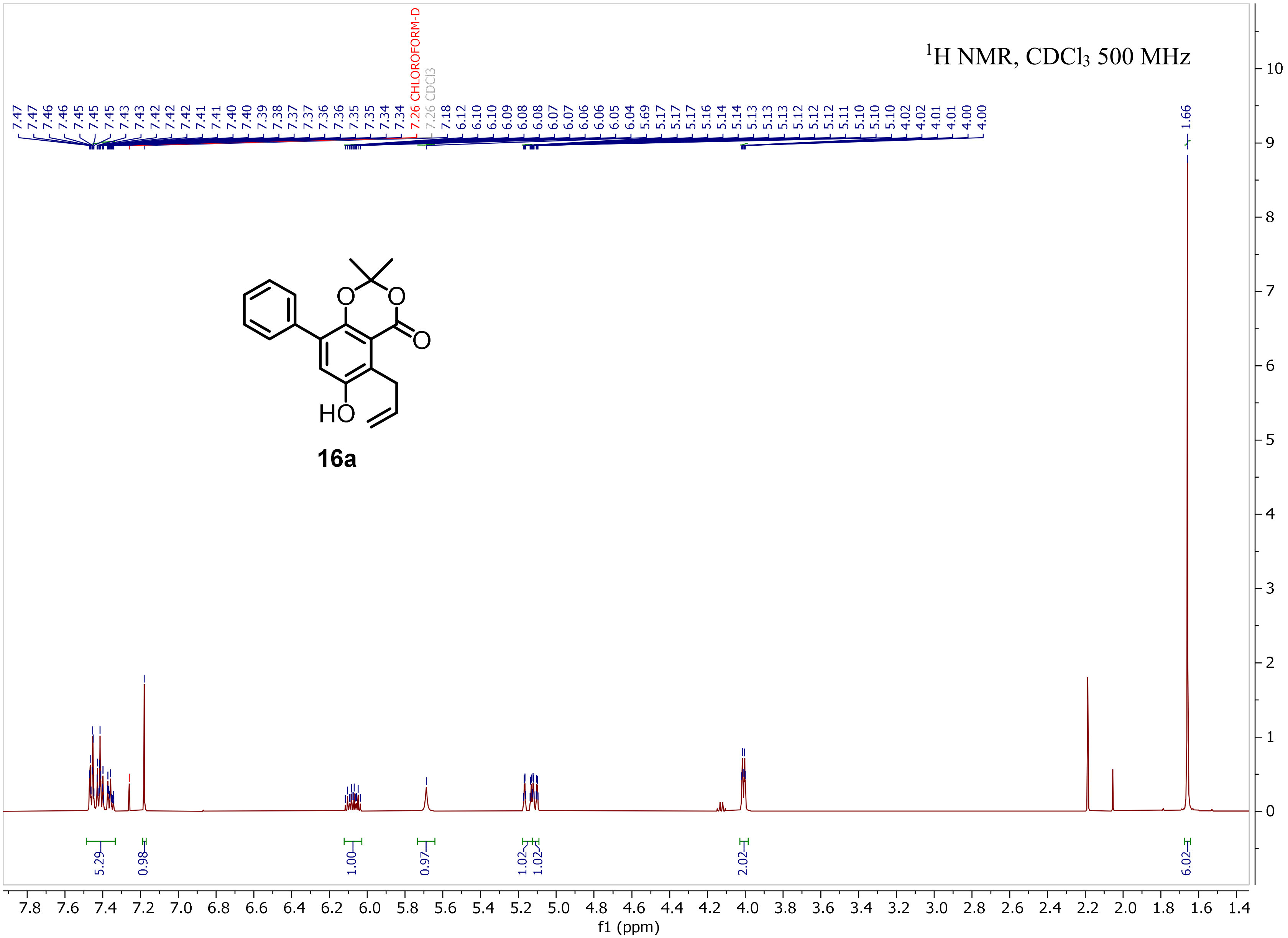

1H NMR, CDCl3 500 MHz
7.47 7.47 7.46 7.45 7.45 7.45 7.43 7.42 7.42 7.42 7.41 7.41 7.40 7.40 7.39 7.38 7.37 7.36 7.36 7.35 7.35 7.34 7.34 7.26 CHLOROFORM-D 7.26 CDCl3 7.18 6.12 6.10 6.10 6.09 6.08 6.08 6.07 6.07 6.06 6.06 6.05 6.04 5.69 5.17 5.17 5.17 5.16 5.14 5.14 5.13 5.13 5.13 5.12 5.12 5.12 5.11 5.10 5.10 5.10 4.02 4.02 4.01 4.01 4.00 4.00
1.66
HO
16a
5.29
0.98
1.00
0.97
1.02
1.02
2.02
6.02
f1 (ppm)

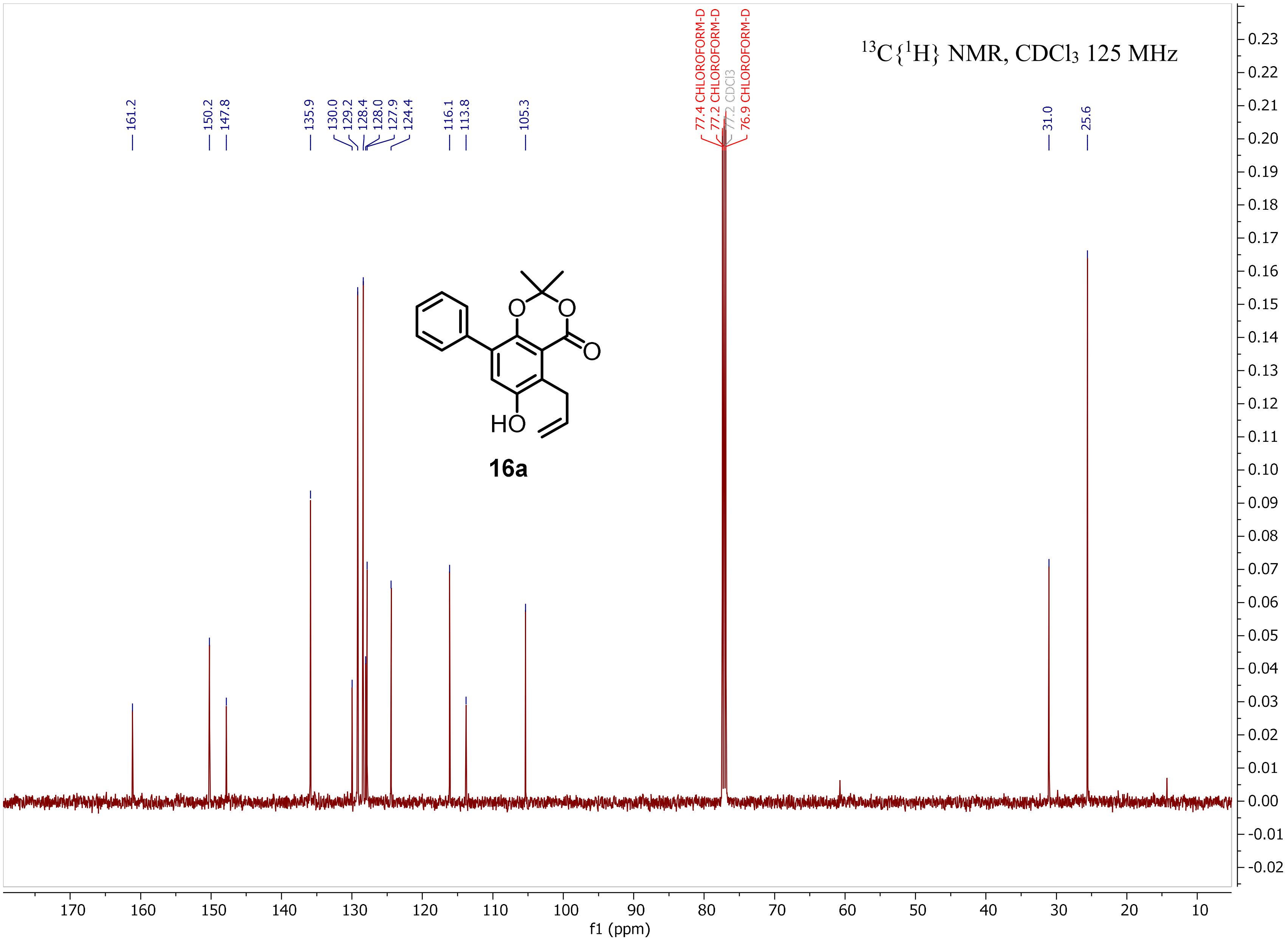

$^{13}C\{^1H\}$ NMR, $CDCl_3$ 125 MHz
161.2
150.2
147.8
135.9
130.0
129.2
128.4
128.0
127.9
124.4
116.1
113.8
105.3
77.4 CHLOROFORM-D
77.2 CHLOROFORM-D
77.2 CDCl3
76.9 CHLOROFORM-D
31.0
25.6
HO
O
16a
f1 (ppm)

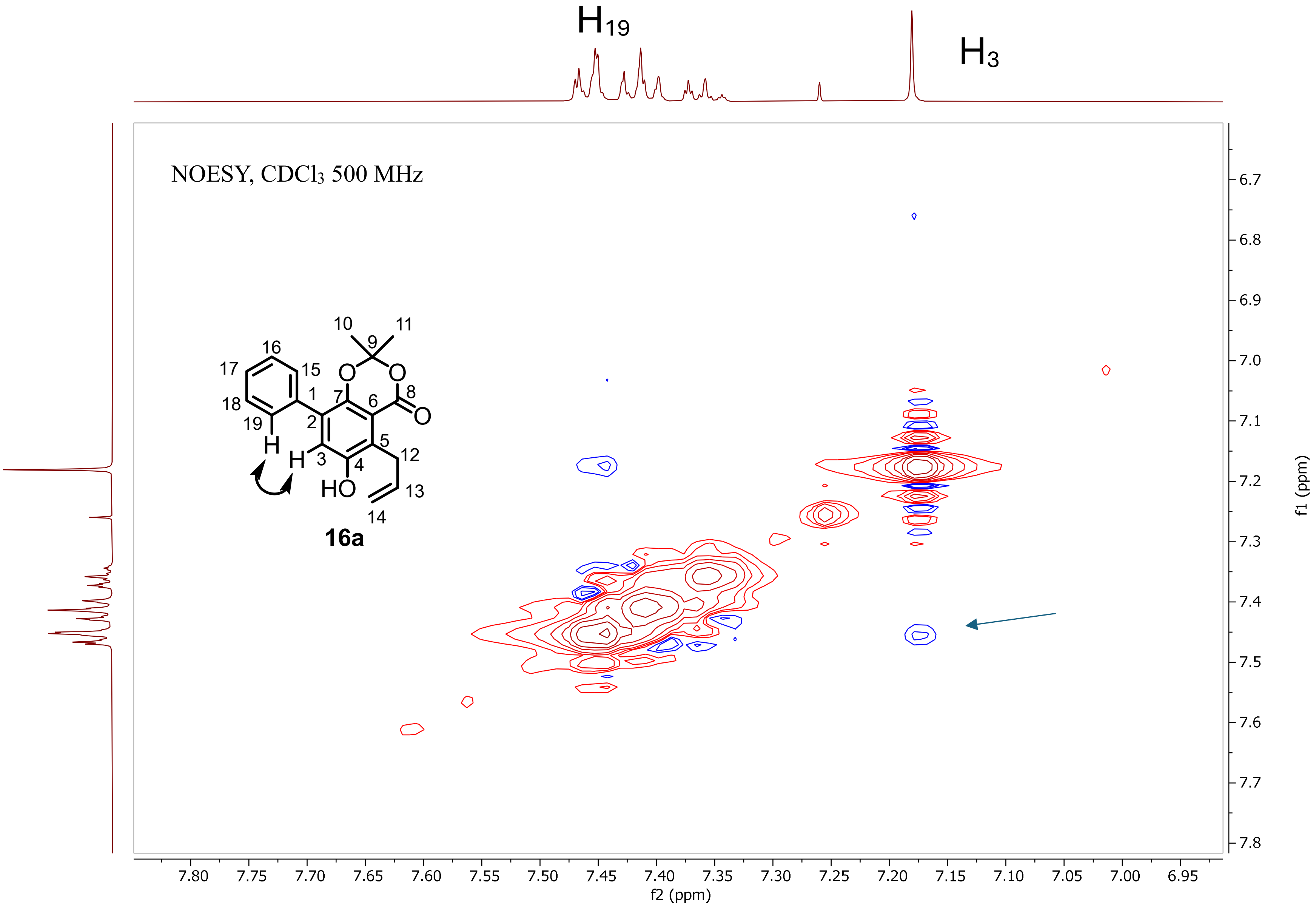
H19
H3
NOESY, CDCl3 500 MHz
16a
f2 (ppm)
f1 (ppm)

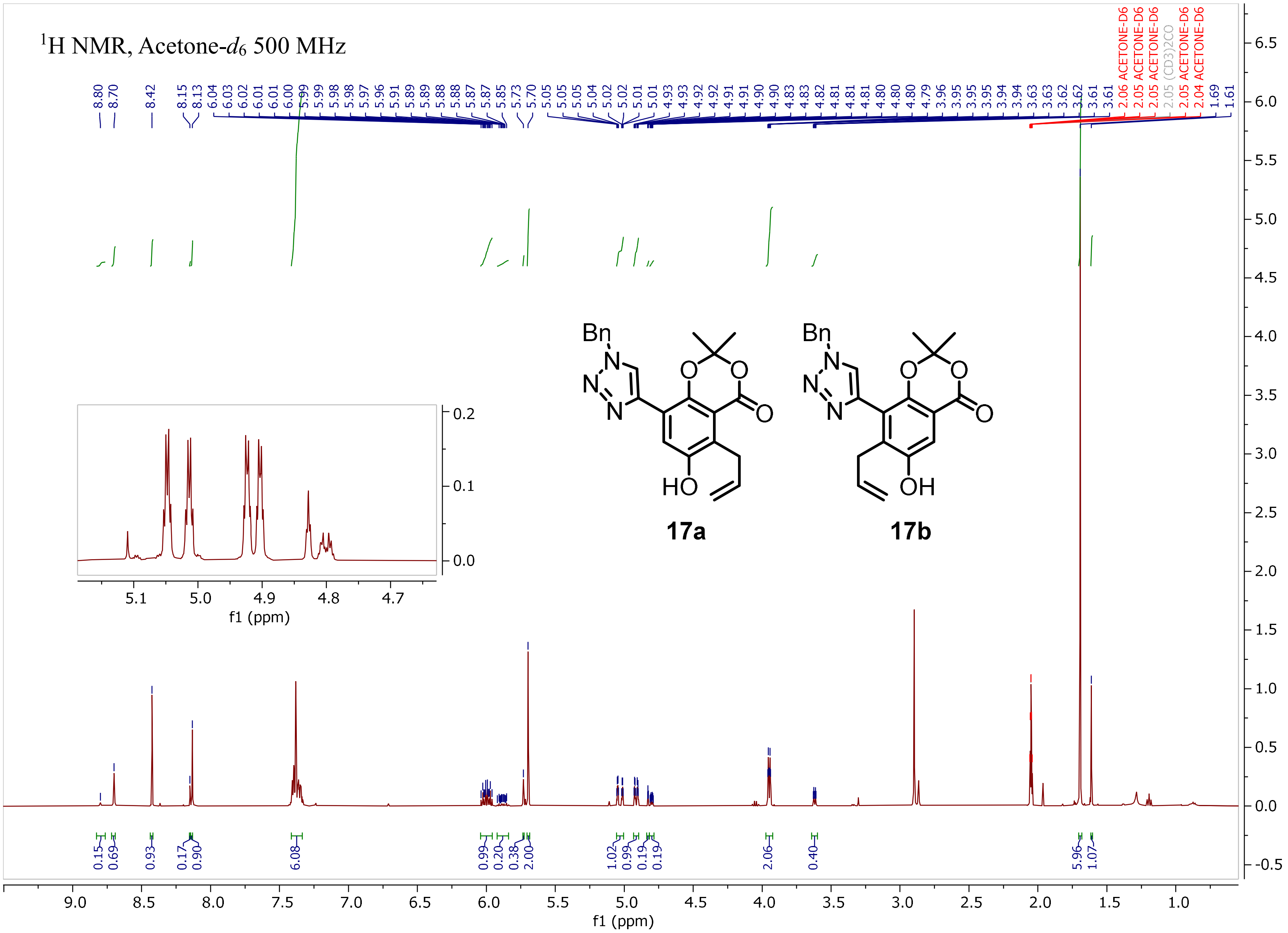

^{1}H NMR, Acetone-d_6 500 MHz
17a
17b
f1 (ppm)

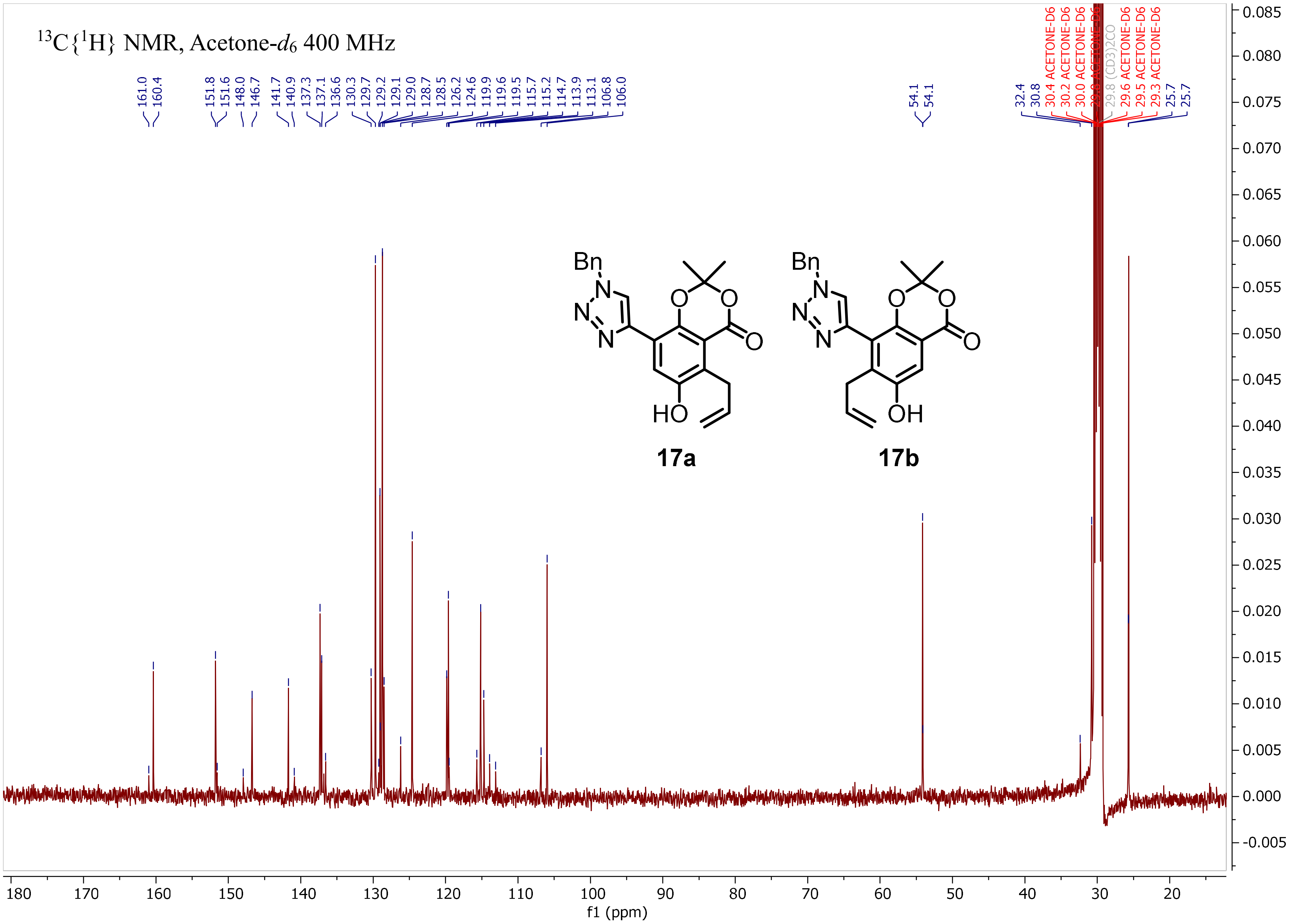

$^{13}C\{^1H\}$ NMR, Acetone-d_6 400 MHz
161.0
160.4
151.8
151.6
148.0
146.7
141.7
140.9
137.3
137.1
136.6
130.3
129.7
129.2
129.1
129.0
128.7
128.5
126.2
124.6
119.9
119.6
119.5
115.7
115.2
114.7
113.9
113.1
106.8
106.0
54.1
54.1
32.4
30.8
30.4 ACETONE-D6
30.2 ACETONE-D6
30.0 ACETONE-D6
29.8 (CD3)2CO
29.6 ACETONE-D6
29.5 ACETONE-D6
29.3 ACETONE-D6
25.7
25.7
Bn
N
N
N
O
O
O
HO
OH
17a
17b
f1 (ppm)

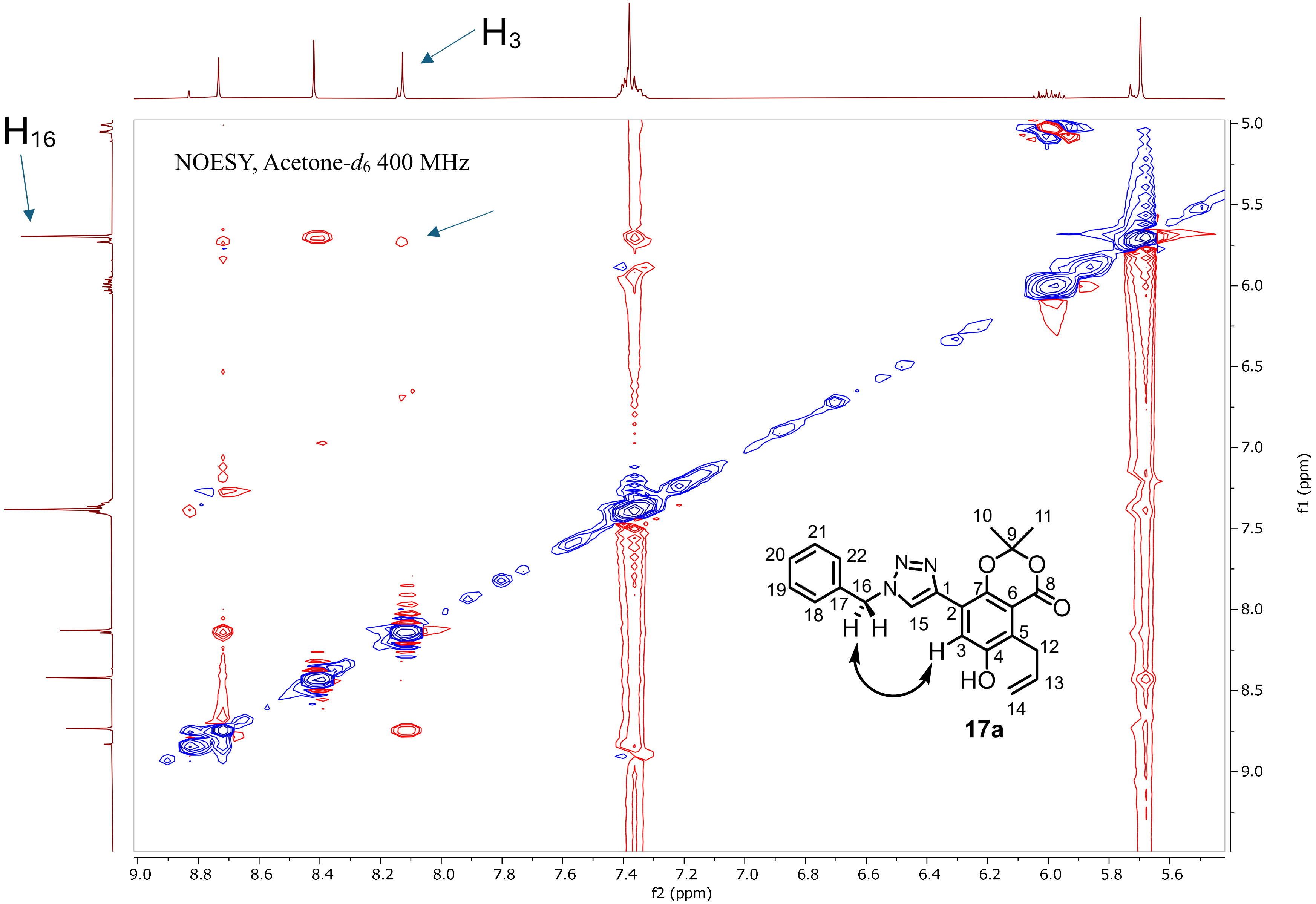

H3
H16
NOESY, Acetone-d6 400 MHz
f2 (ppm)
f1 (ppm)
9.0
8.8
8.6
8.4
8.2
8.0
7.8
7.6
7.4
7.2
7.0
6.8
6.6
6.4
6.2
6.0
5.8
5.6
5.0
5.5
6.0
6.5
7.0
7.5
8.0
8.5
9.0
10
11
9
21
20
22
N=N
O
O
8
19
16
N
1
7
6
O
17
18
2
15
H
H
5
H
3
4
12
HO
13
14
17a

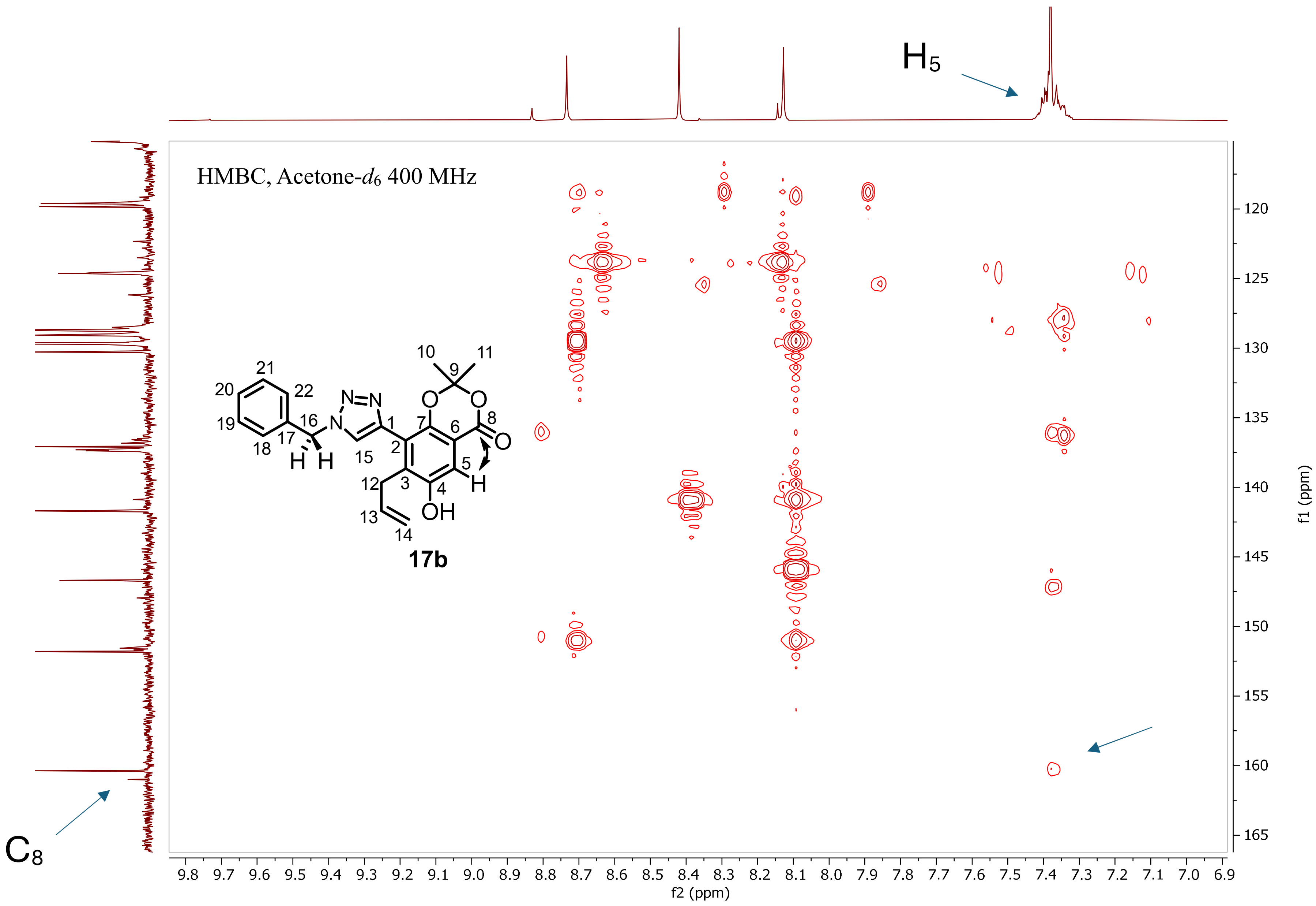

H5
HMBC, Acetone-d_6 400 MHz
10
11
9
21
20
22
N=N
O
O
8
19
16
N
1
7
6
O
18
17
2
15
5
H H
12
3
4
H
13
OH
14
17b
C8
120
125
130
135
140
145
150
155
160
165
f1 (ppm)
9.8 9.7 9.6 9.5 9.4 9.3 9.2 9.1 9.0 8.9 8.8 8.7 8.6 8.5 8.4 8.3 8.2 8.1 8.0 7.9 7.8 7.7 7.6 7.5 7.4 7.3 7.2 7.1 7.0 6.9
f2 (ppm)

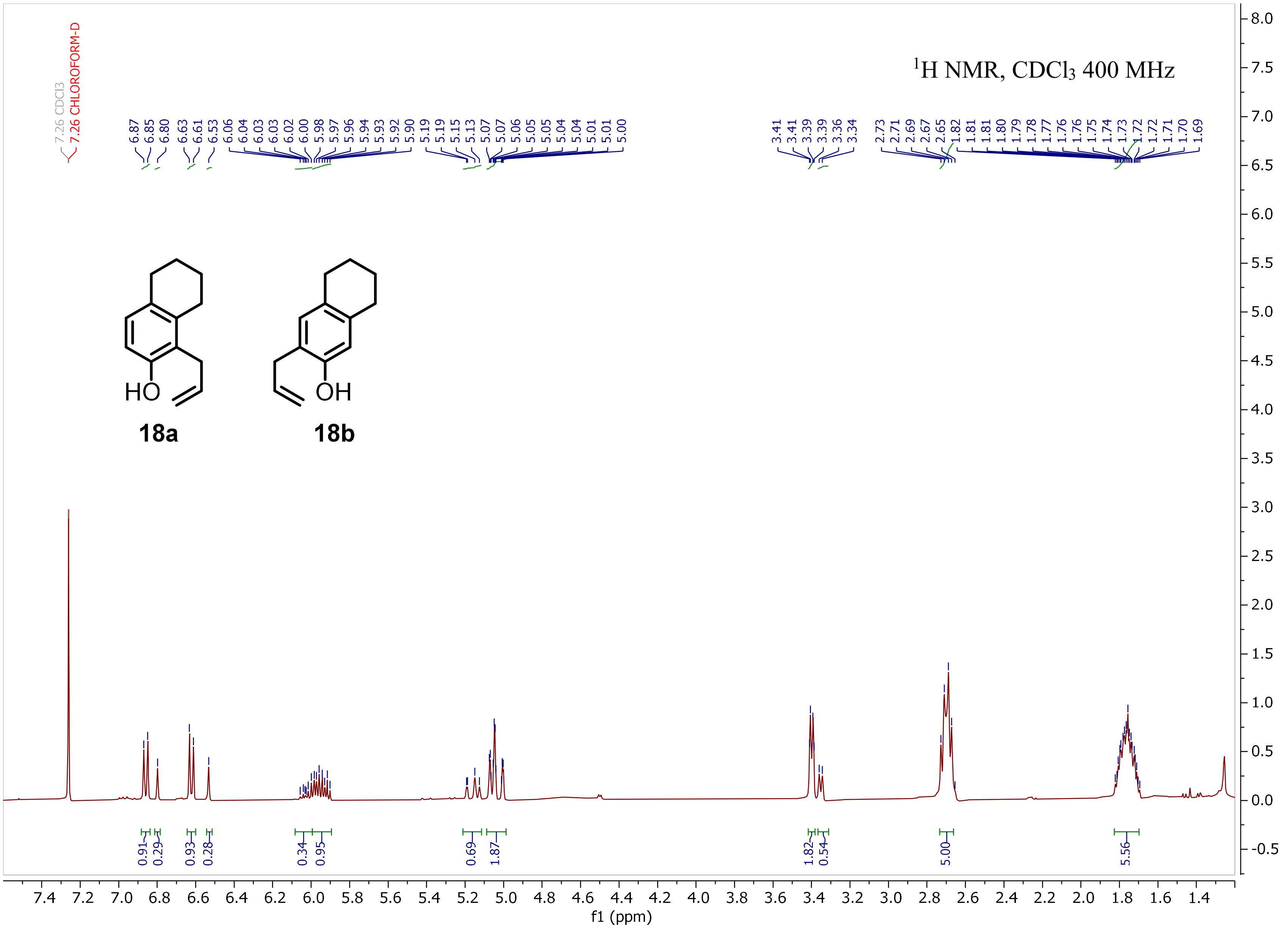

^{1}H NMR, CDCl$_3$ 400 MHz
7.26 CDCl3
7.26 CHLOROFORM-D
HO
OH
18a
18b
f1 (ppm)

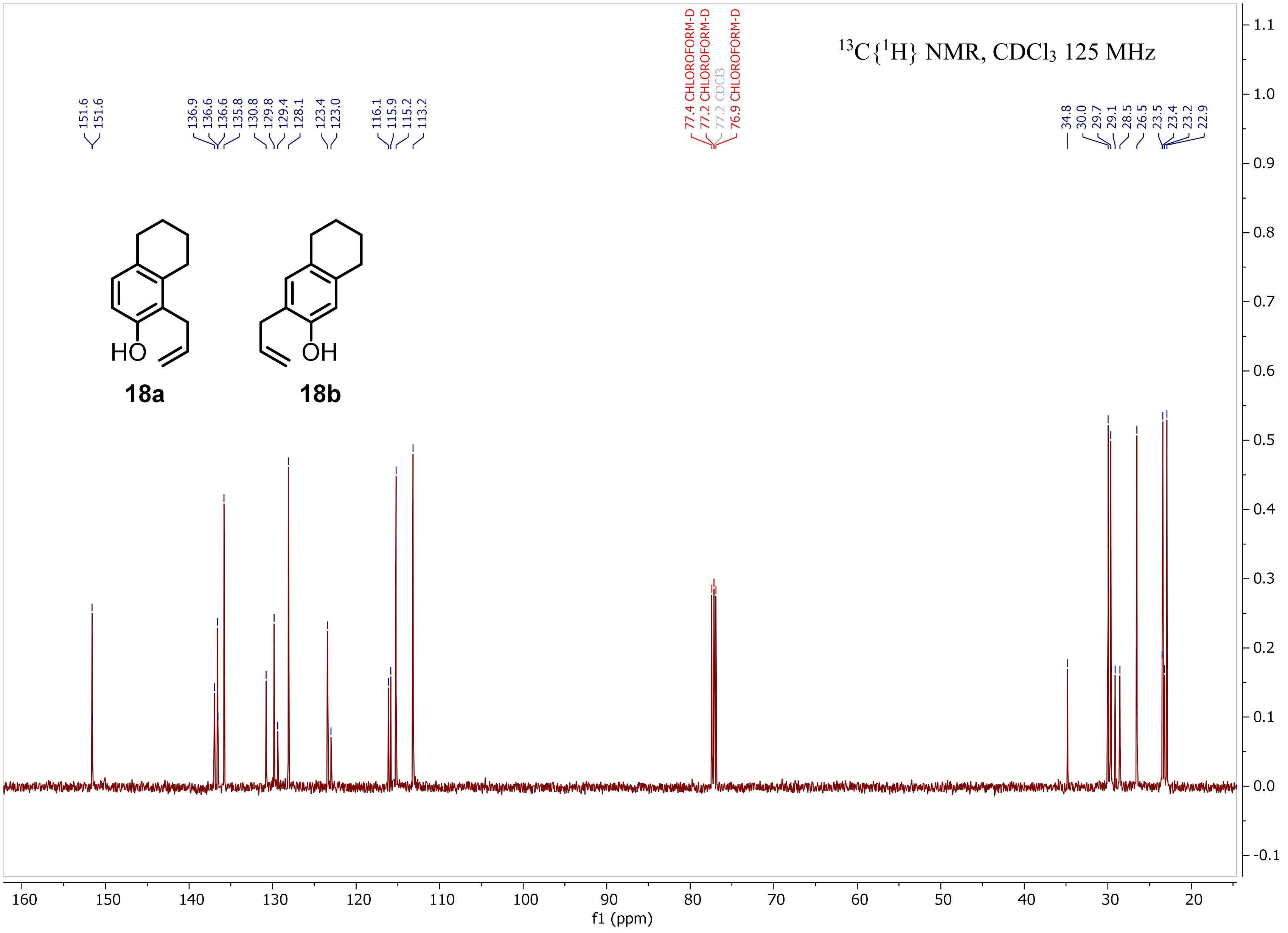

$^{13}C\{^1H\}$ NMR, $CDCl_3$ 125 MHz
151.6
151.6
136.9
136.6
136.6
135.8
130.8
129.8
129.4
128.1
123.4
123.0
116.1
115.9
115.2
113.2
77.4 CHLOROFORM-D
77.2 CHLOROFORM-D
77.2 CDCl3
76.9 CHLOROFORM-D
34.8
30.0
29.7
29.1
28.5
26.5
23.5
23.4
23.2
22.9
HO
18a
OH
18b
f1 (ppm)

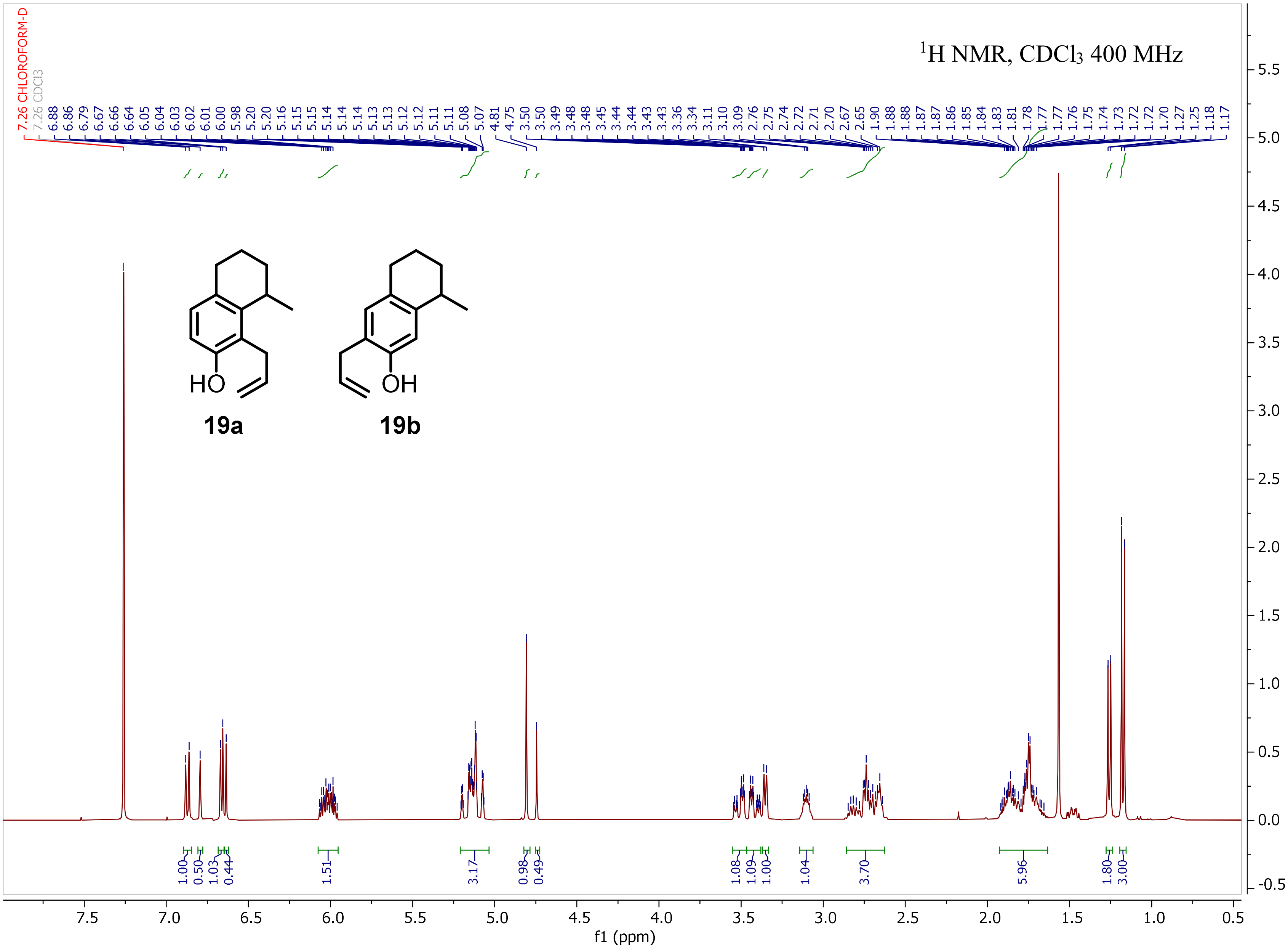

1H NMR, CDCl3 400 MHz
7.26 CHLOROFORM-D
7.26 CDCl3
6.88 6.86 6.79 6.67 6.66 6.64 6.05 6.04 6.03 6.02 6.01 6.00 5.98 5.20 5.16 5.15 5.15 5.14 5.14 5.14 5.13 5.13 5.12 5.12 5.11 5.11 5.08 5.07 4.81 4.75 3.50 3.50 3.49 3.48 3.48 3.45 3.44 3.44 3.43 3.43 3.36 3.34 3.11 3.10 3.09 2.76 2.75 2.74 2.72 2.71 2.70 2.67 2.65 1.90 1.88 1.88 1.87 1.87 1.86 1.85 1.84 1.83 1.81 1.78 1.77 1.77 1.76 1.75 1.74 1.73 1.72 1.72 1.70 1.27 1.25 1.18 1.17
HO
OH
19a
19b
1.00 0.50 1.03 0.44 1.51 3.17 0.98 0.49 1.08 1.09 1.00 1.04 3.70 5.96 1.80 3.00
f1 (ppm)

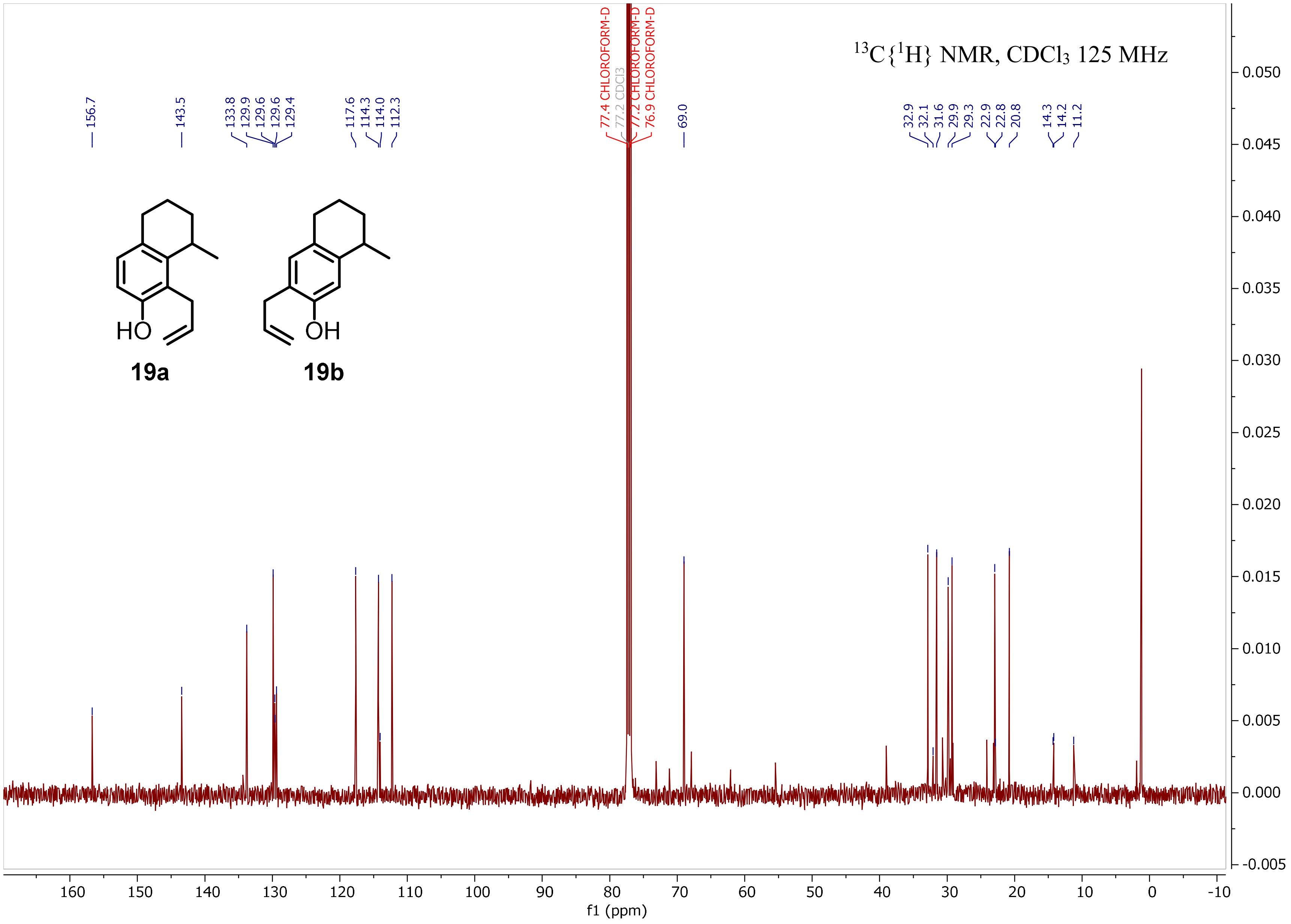

$^{13}C\{^1H\}$ NMR, $CDCl_3$ 125 MHz
156.7
143.5
133.8
129.9
129.6
129.6
129.4
117.6
114.3
114.0
112.3
77.4 CHLOROFORM-D
77.2 CDCl3
77.2 CHLOROFORM-D
76.9 CHLOROFORM-D
69.0
32.9
32.1
31.6
29.9
29.3
22.9
22.8
20.8
14.3
14.2
11.2
HO
OH
19a
19b
f1 (ppm)

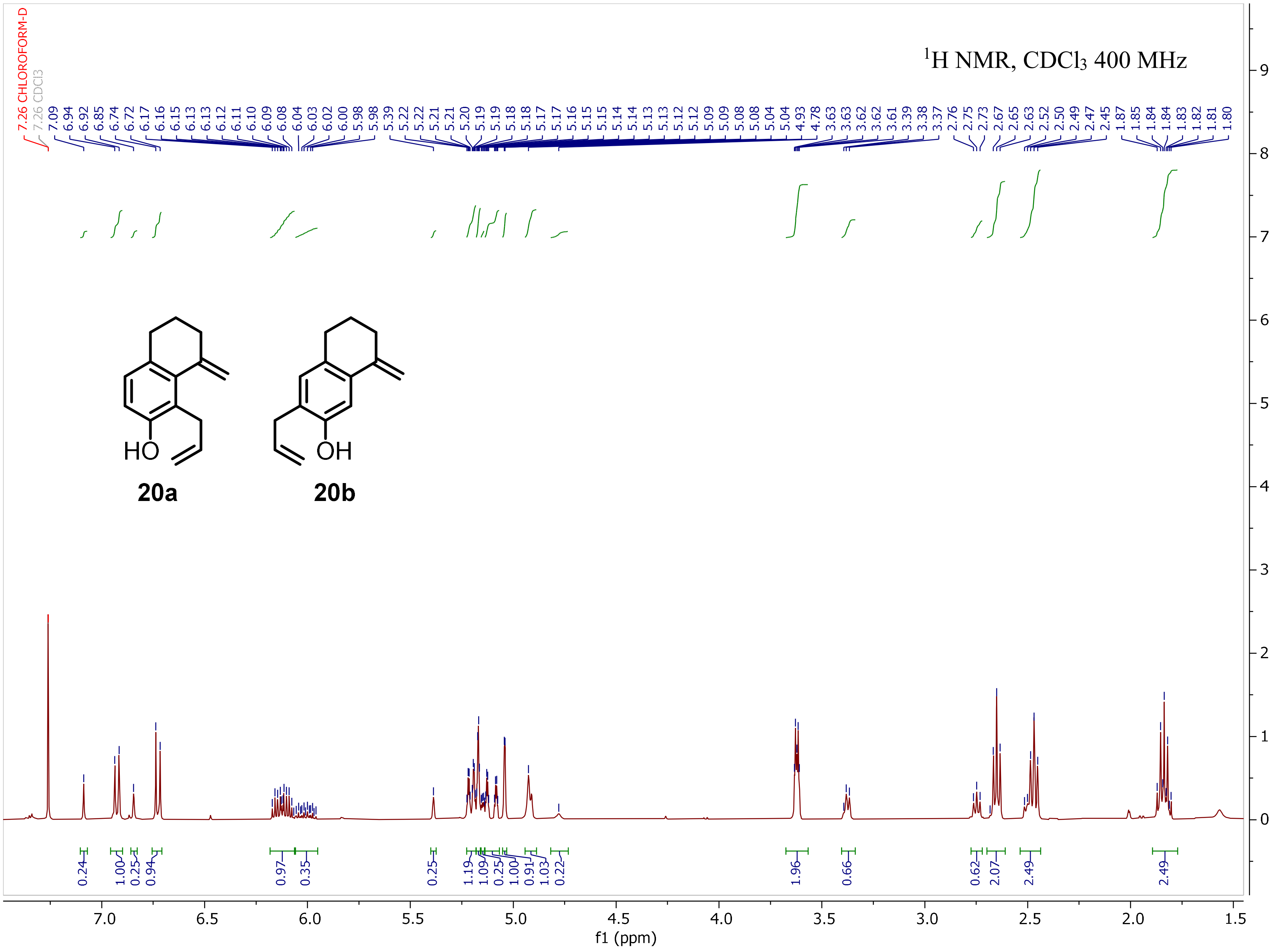

¹H NMR, CDCl3 400 MHz
HO
OH
20a
20b
f1 (ppm)

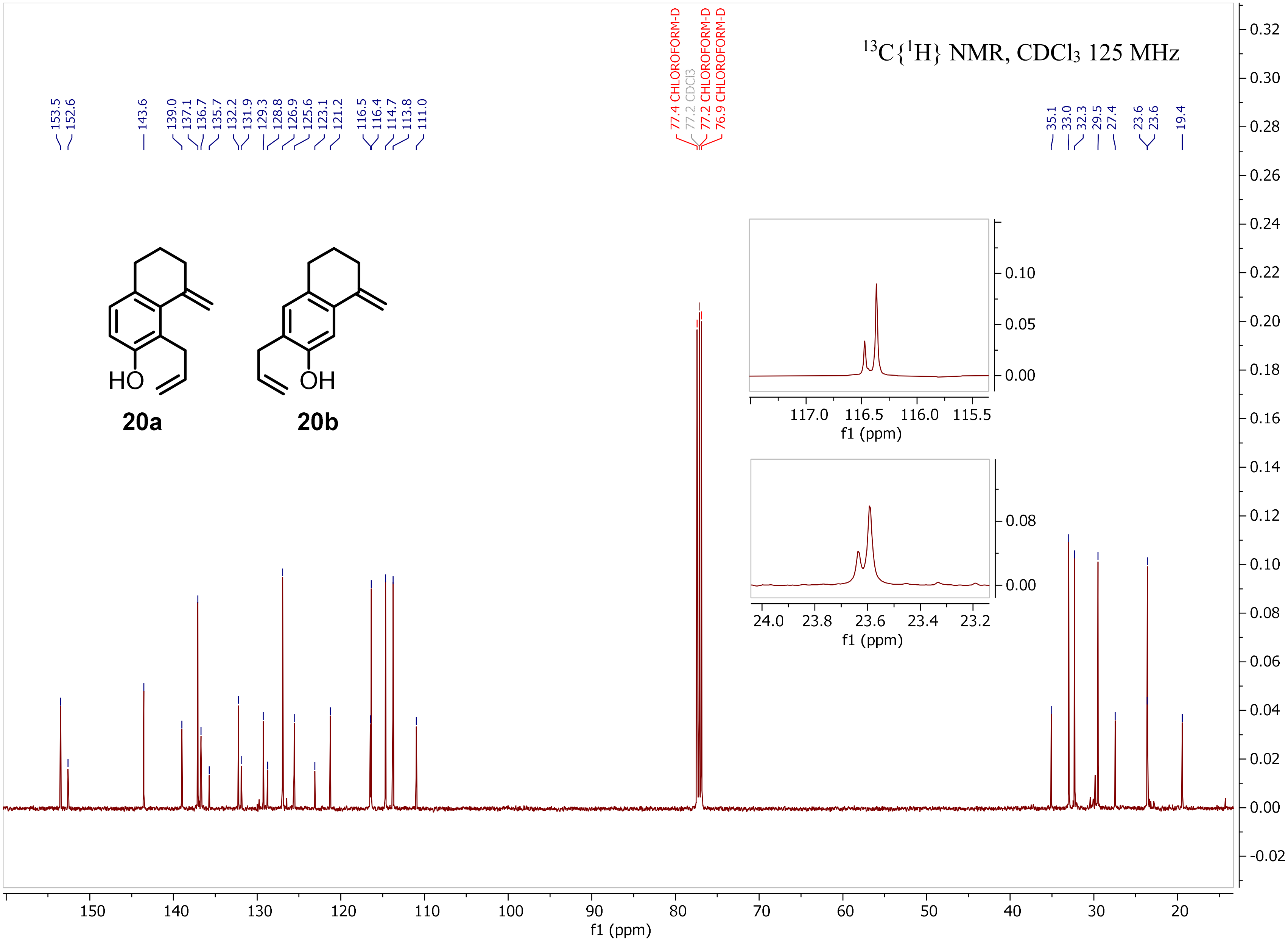

$^{13}C\{^1H\}$ NMR, $CDCl_3$ 125 MHz
153.5
152.6
143.6
139.0
137.1
136.7
135.7
132.2
131.9
129.3
128.8
126.9
125.6
123.1
121.2
116.5
116.4
114.7
113.8
111.0
77.4 CHLOROFORM-D
77.2 CDCl3
77.2 CHLOROFORM-D
76.9 CHLOROFORM-D
35.1
33.0
32.3
29.5
27.4
23.6
23.6
19.4
HO
OH
20a
20b
f1 (ppm)

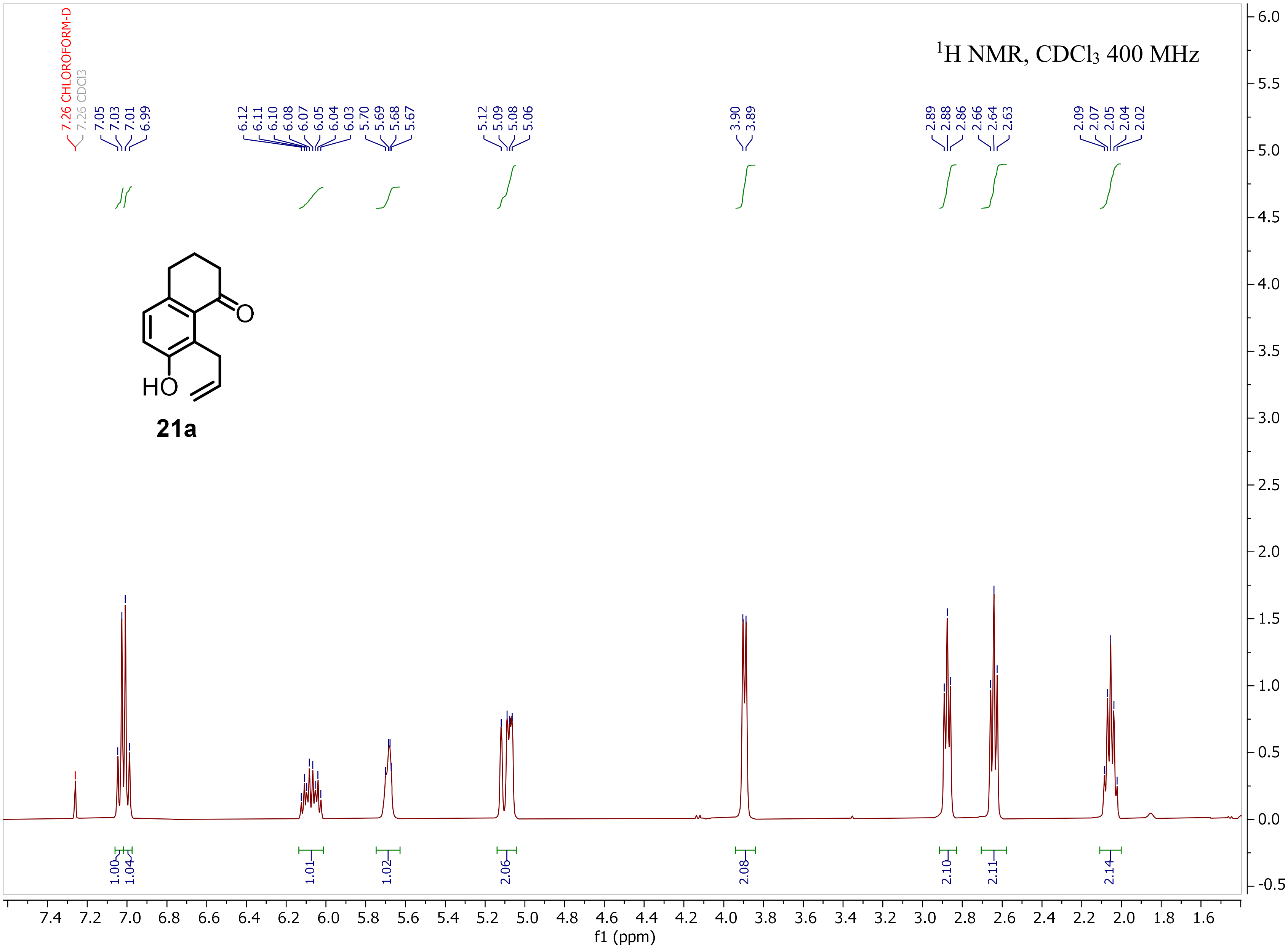

1H NMR, CDCl3 400 MHz
7.26 CHLOROFORM-D
7.26 CDCl3
7.05
7.03
7.01
6.99
6.12
6.11
6.10
6.08
6.07
6.05
6.04
6.03
5.70
5.69
5.68
5.67
5.12
5.09
5.08
5.06
3.90
3.89
2.89
2.88
2.86
2.66
2.64
2.63
2.09
2.07
2.05
2.04
2.02
HO
O
21a
1.00
1.04
1.01
1.02
2.06
2.08
2.10
2.11
2.14
f1 (ppm)

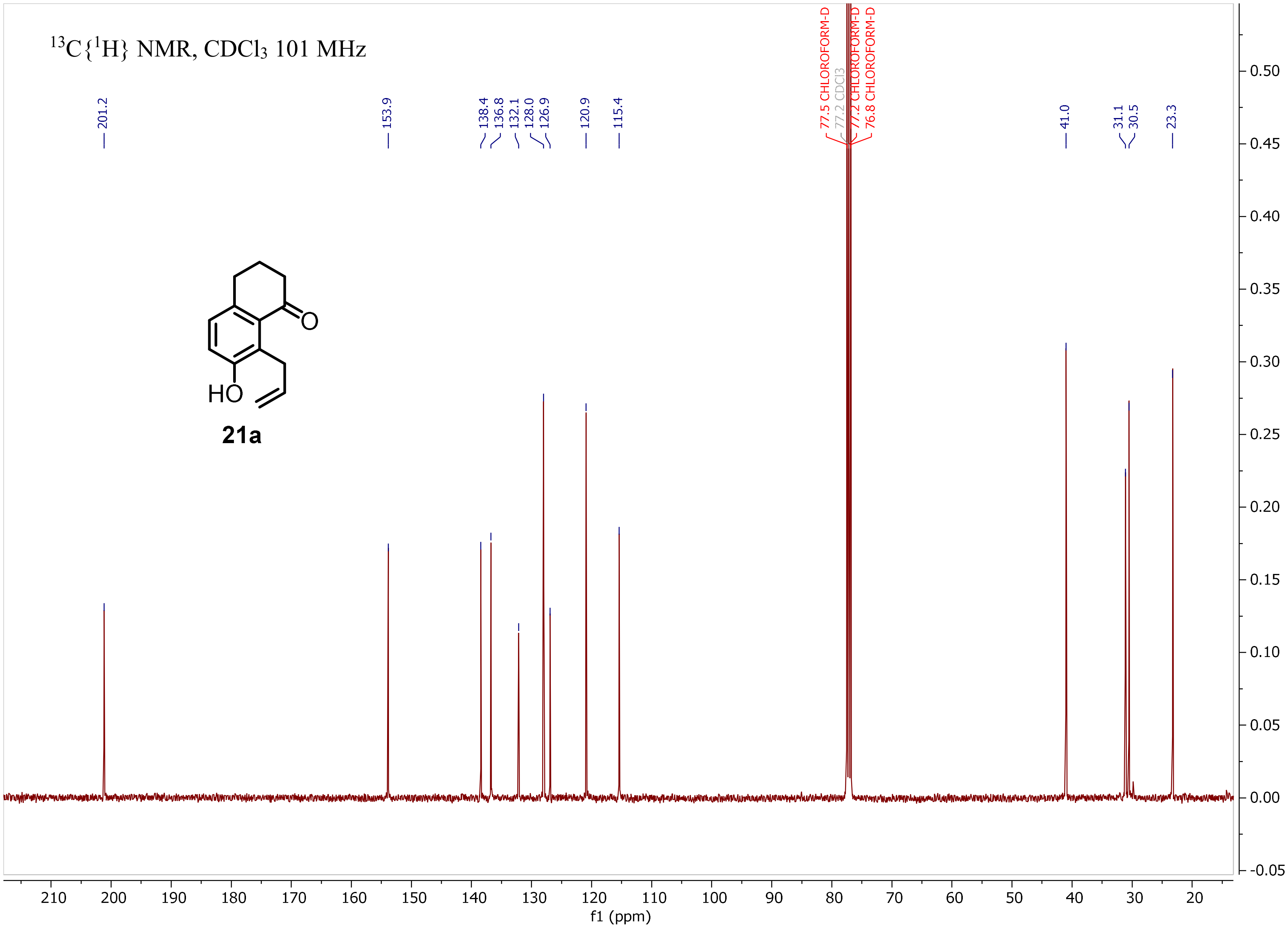

$^{13}C\{^1H\}$ NMR, $CDCl_3$ 101 MHz
201.2
153.9
138.4
136.8
132.1
128.0
126.9
120.9
115.4
77.5 CHLOROFORM-D
77.2 CDCl3
77.2 CHLOROFORM-D
76.8 CHLOROFORM-D
41.0
31.1
30.5
23.3
HO
O
21a
f1 (ppm)

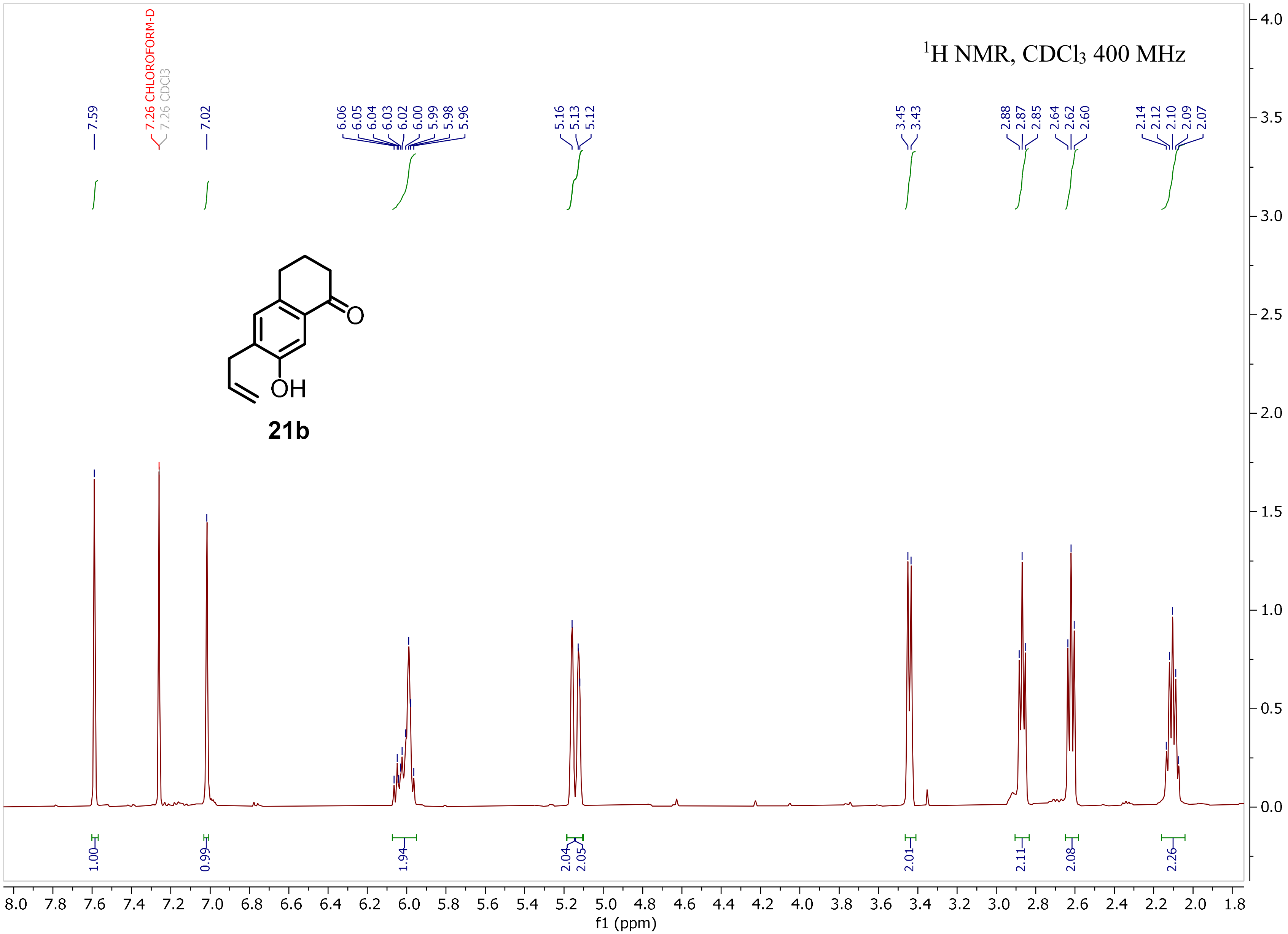

1H NMR, CDCl3 400 MHz
7.59
7.26 CHLOROFORM-D
7.26 CDCl3
7.02
6.06 6.05 6.04 6.03 6.02 6.00 5.99 5.98 5.96
5.16 5.13 5.12
3.45 3.43
2.88 2.87 2.85 2.64 2.62 2.60
2.14 2.12 2.10 2.09 2.07
OH
O
21b
1.00
0.99
1.94
2.04
2.05
2.01
2.11
2.08
2.26
f1 (ppm)

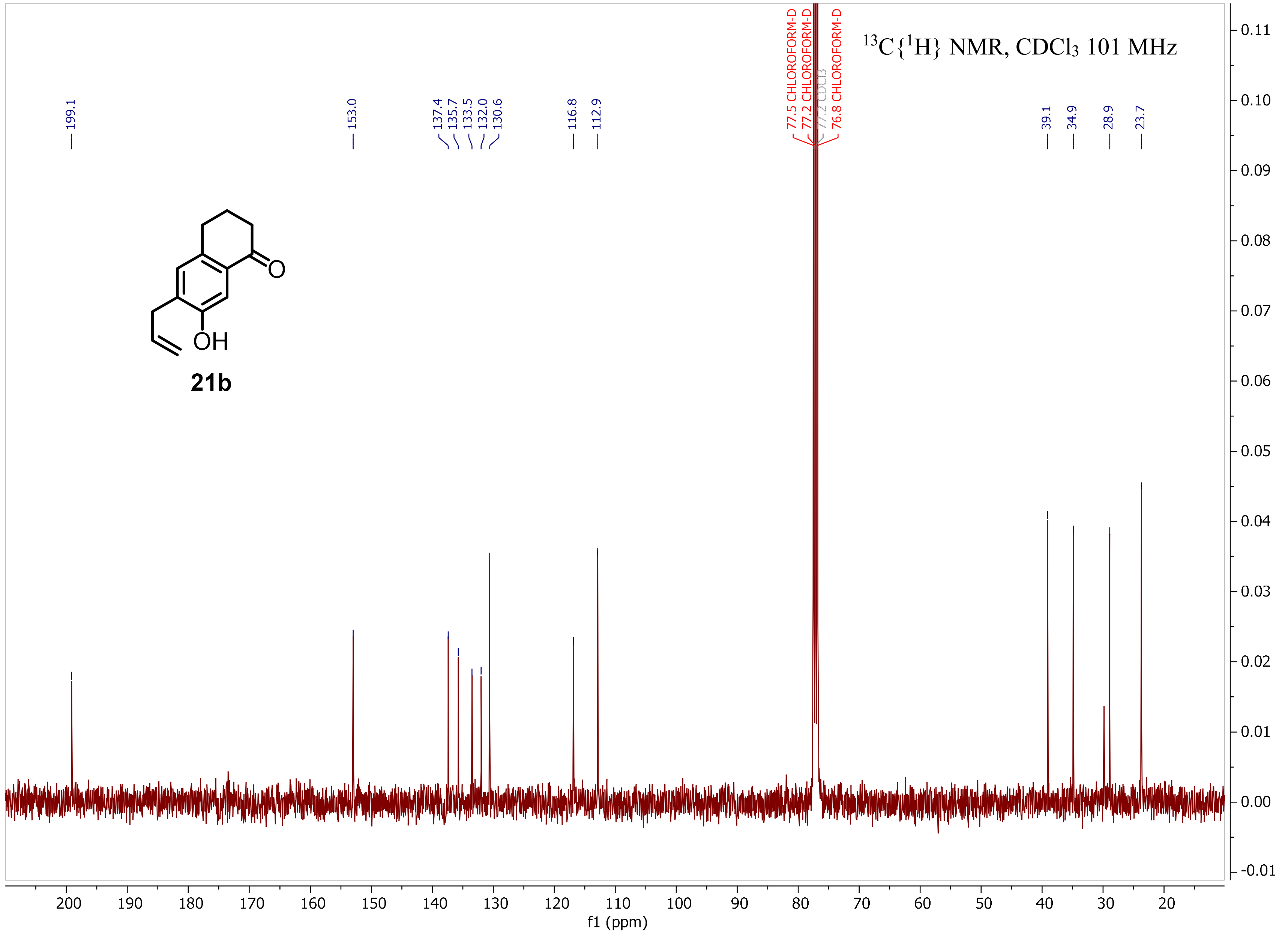

13C{1H} NMR, CDCl3 101 MHz
199.1
153.0
137.4
135.7
133.5
132.0
130.6
116.8
112.9
77.5 CHLOROFORM-D
77.2 CHLOROFORM-D
77.2 CDCl3
76.8 CHLOROFORM-D
39.1
34.9
28.9
23.7
OH
O
21b
f1 (ppm)

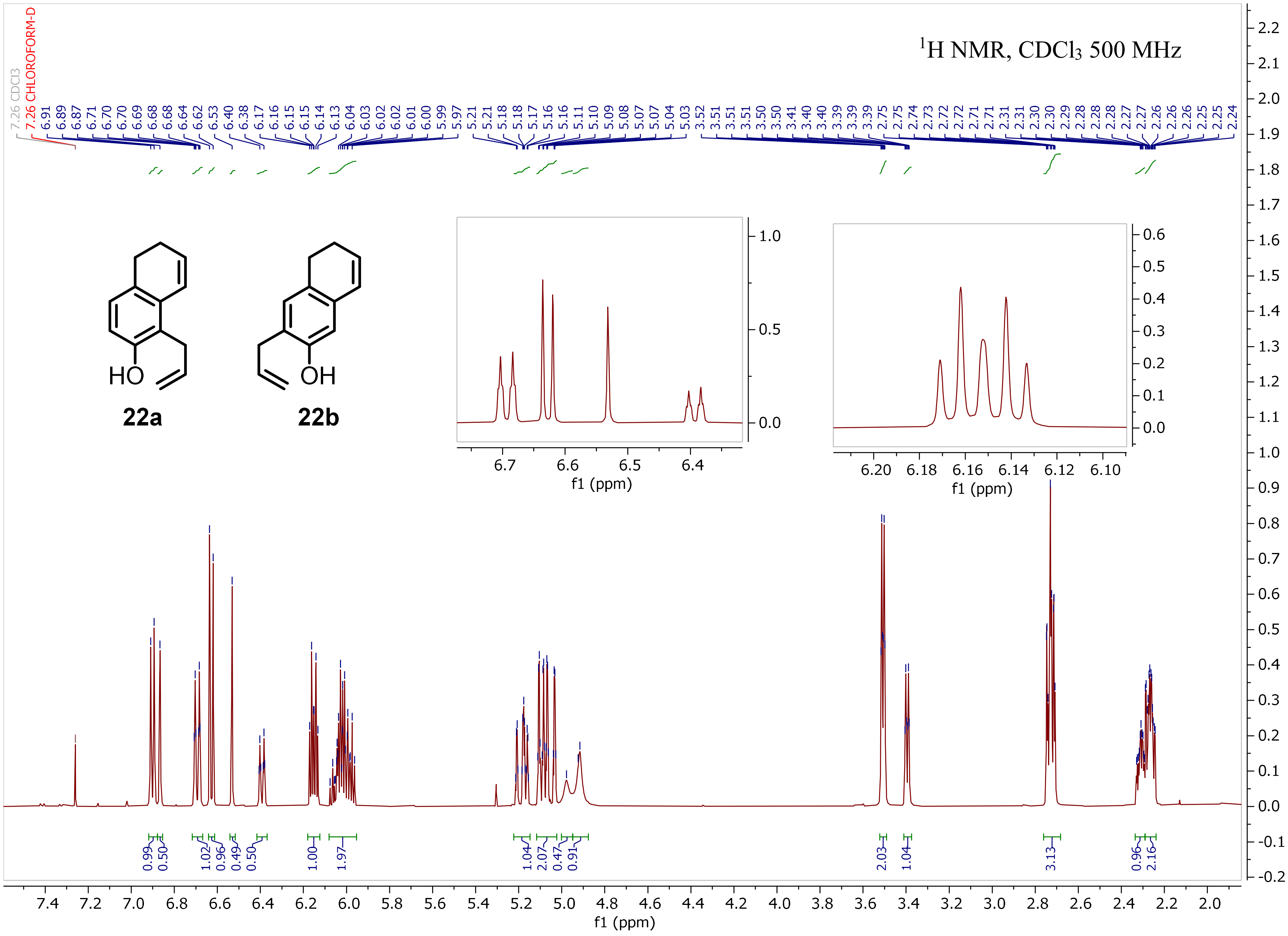

¹H NMR, CDCl3 500 MHz
22a
22b
HO
OH
f1 (ppm)

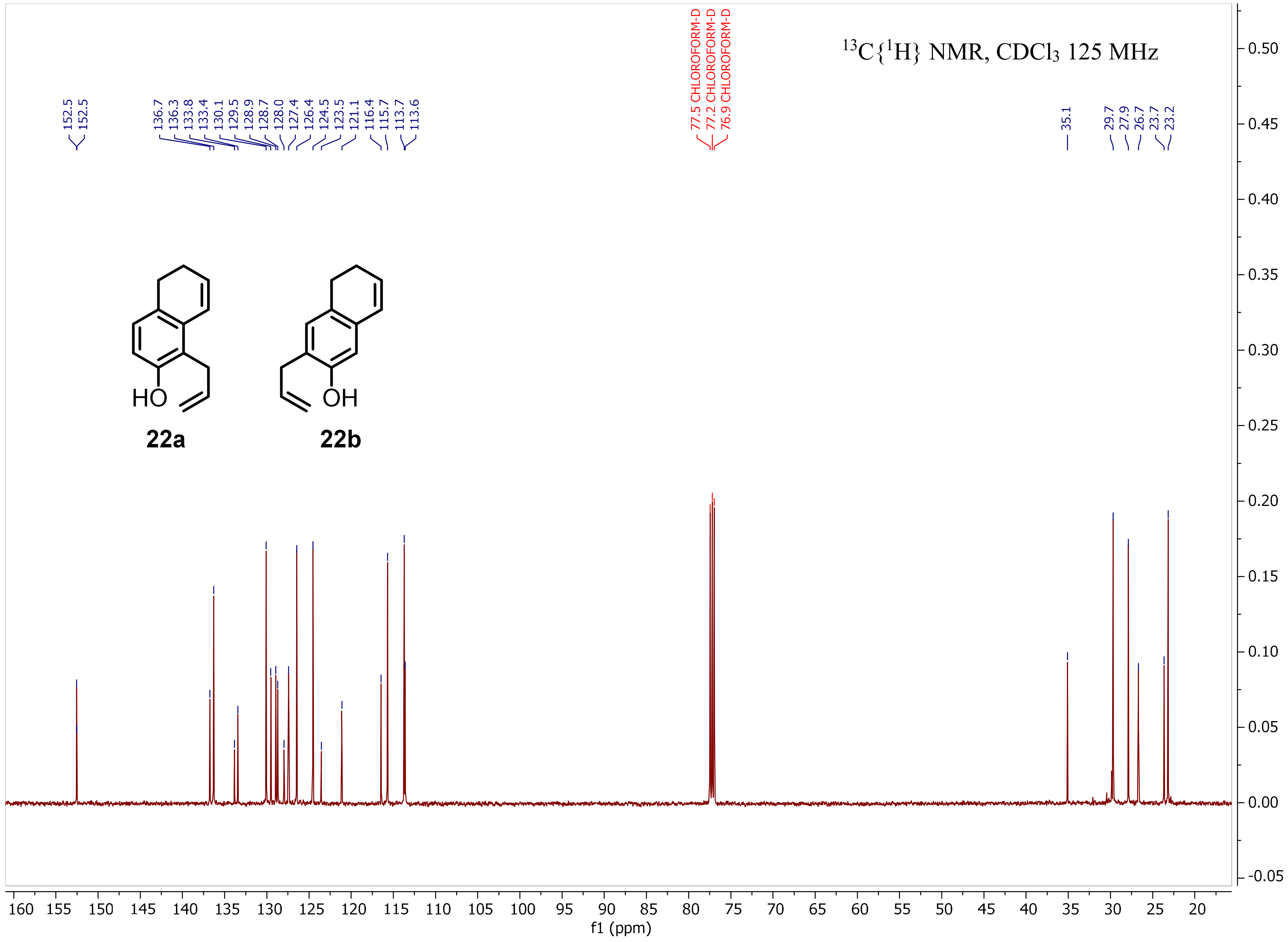

$^{13}C\{^1H\}$ NMR, $CDCl_3$ 125 MHz
152.5
152.5
136.7
136.3
133.8
133.4
130.1
129.5
128.9
128.7
128.0
127.4
126.4
124.5
123.5
121.1
116.4
115.7
113.7
113.6
77.5 CHLOROFORM-D
77.2 CHLOROFORM-D
76.9 CHLOROFORM-D
35.1
29.7
27.9
26.7
23.7
23.2
HO
OH
22a
22b
f1 (ppm)

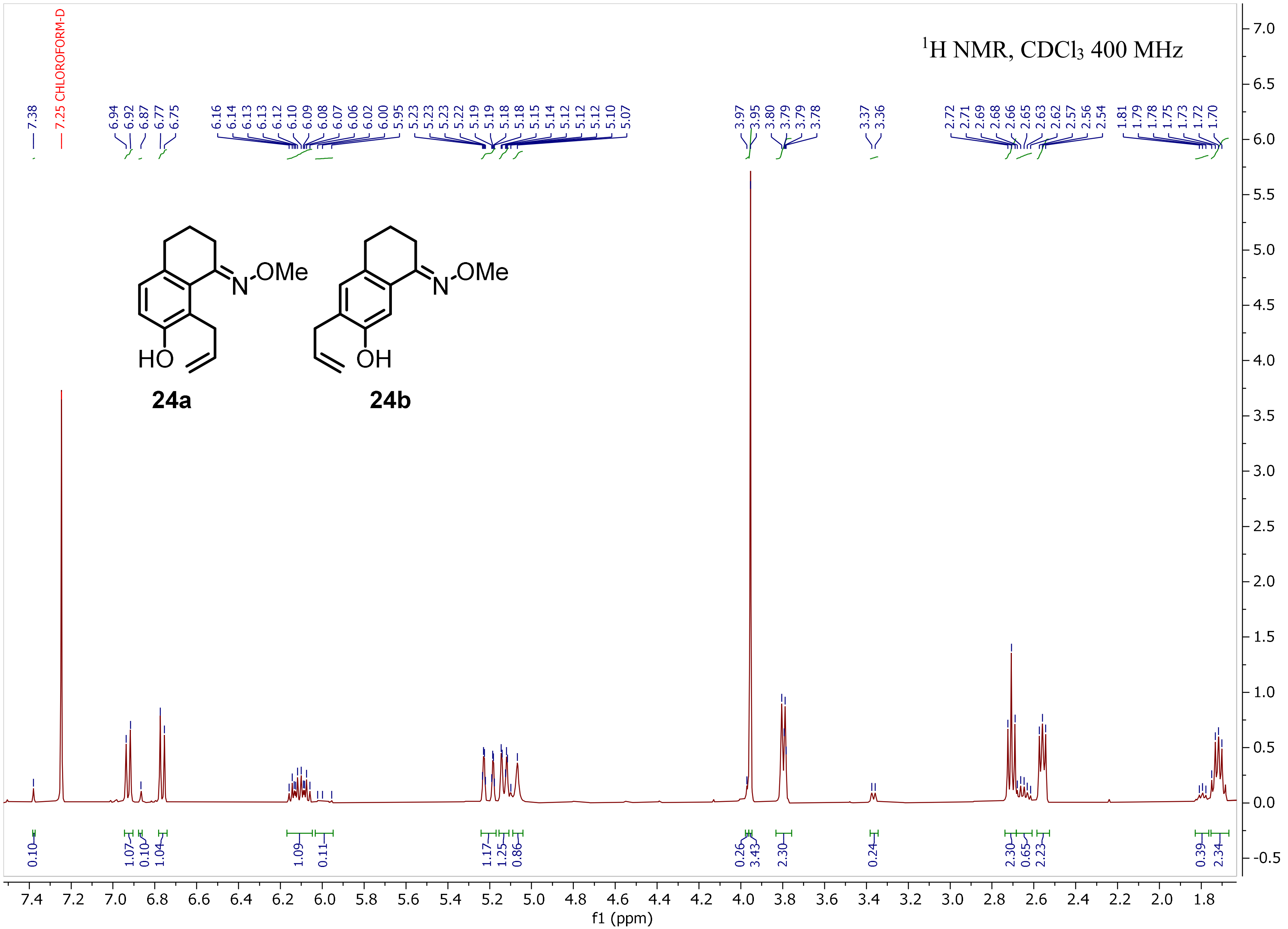

1H NMR, CDCl3 400 MHz
7.25 CHLOROFORM-D
7.38
6.94 6.92 6.87 6.77 6.75
6.16 6.14 6.13 6.13 6.12 6.10 6.09 6.08 6.07 6.06 6.02 6.00 5.95
5.23 5.23 5.23 5.22 5.19 5.19 5.18 5.18 5.15 5.14 5.12 5.12 5.12 5.10 5.07
3.97 3.95 3.80 3.79 3.79 3.78
3.37 3.36
2.72 2.71 2.69 2.68 2.66 2.65 2.63 2.62 2.57 2.56 2.54
1.81 1.79 1.78 1.75 1.73 1.72 1.70
OMe
N
HO
OH
24a
24b
0.10 1.07 0.10 1.04 1.09 0.11 1.17 1.25 0.86 0.26 3.43 2.30 0.24 2.30 0.65 2.23 0.39 2.34
f1 (ppm)

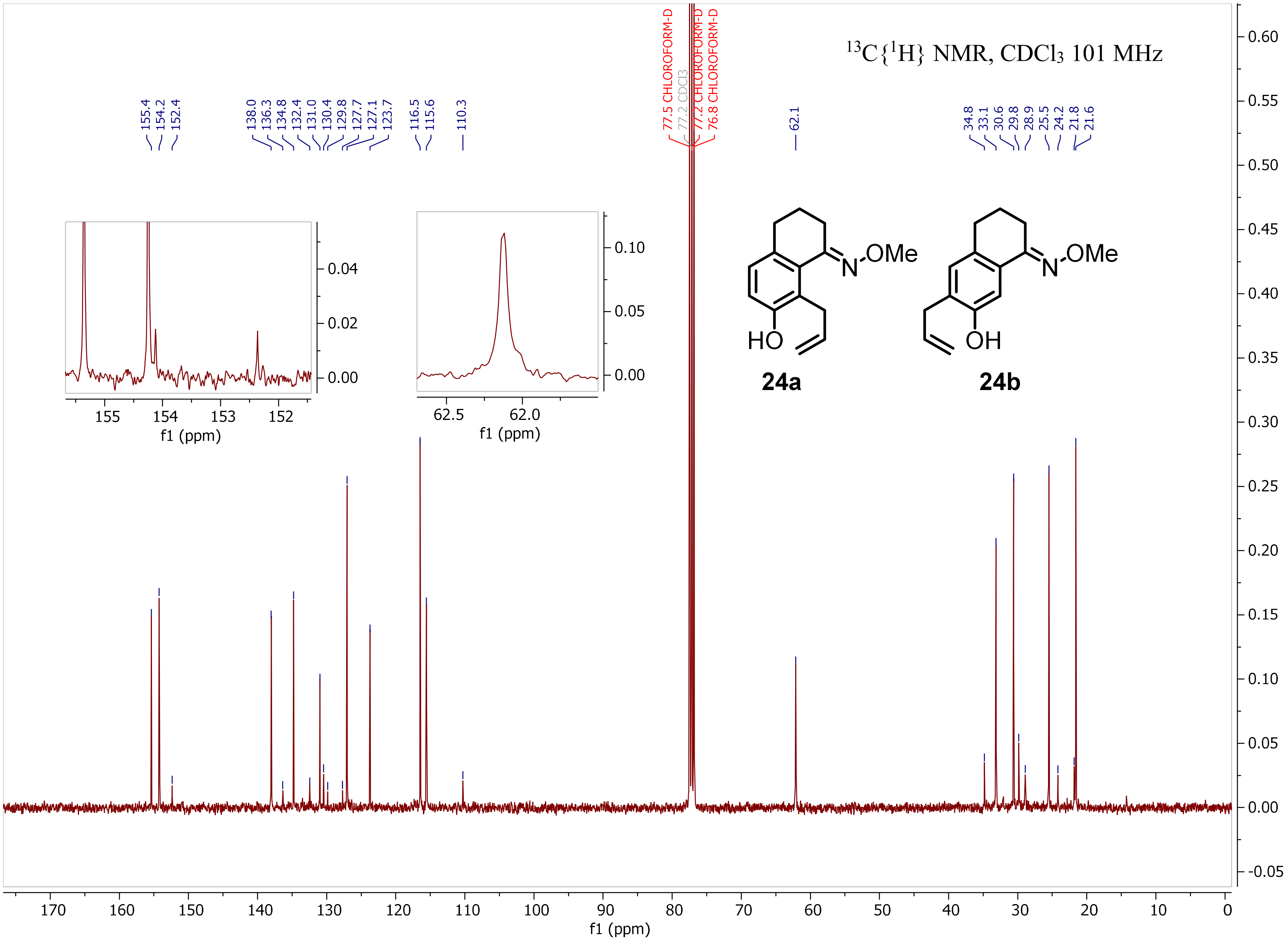

$^{13}C\{^{1}H\}$ NMR, $CDCl_3$ 101 MHz
155.4
154.2
152.4
138.0
136.3
134.8
132.4
131.0
130.4
129.8
127.7
127.1
123.7
116.5
115.6
110.3
77.5 CHLOROFORM-D
77.2 CDCl3
77.2 CHLOROFORM-D
76.8 CHLOROFORM-D
62.1
34.8
33.1
30.6
29.8
28.9
25.5
24.2
21.8
21.6
OMe
N
HO
24a
OMe
N
OH
24b
f1 (ppm)

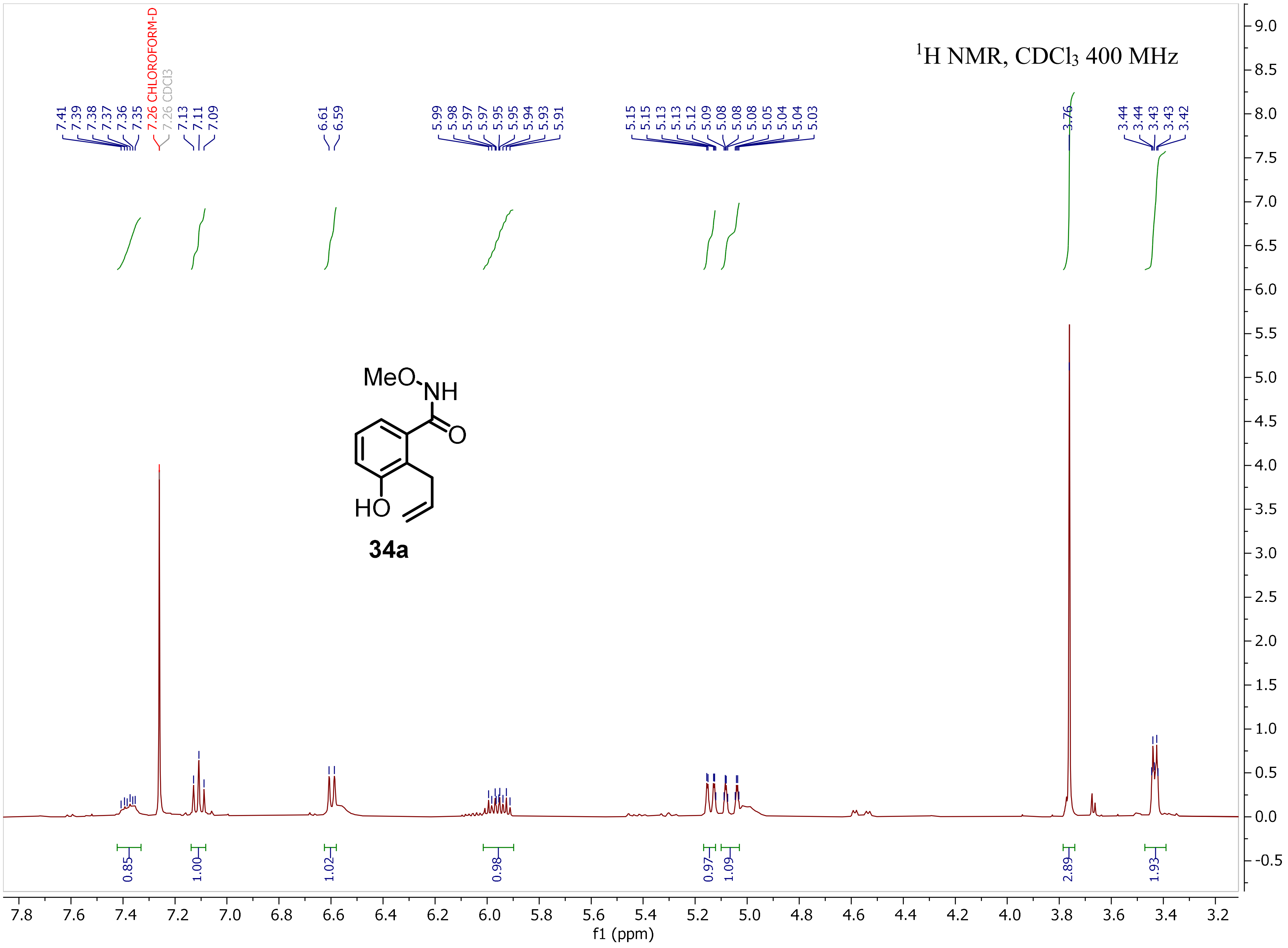

1H NMR, CDCl3 400 MHz
7.41
7.39
7.38
7.37
7.36
7.35
7.26 CHLOROFORM-D
7.26 CDCl3
7.13
7.11
7.09
6.61
6.59
5.99
5.98
5.97
5.97
5.95
5.95
5.94
5.93
5.91
5.15
5.15
5.13
5.13
5.12
5.09
5.08
5.08
5.05
5.04
5.04
5.03
3.76
3.44
3.44
3.43
3.43
3.42
MeO
NH
O
HO
34a
0.85
1.00
1.02
0.98
0.97
1.09
2.89
1.93
f1 (ppm)

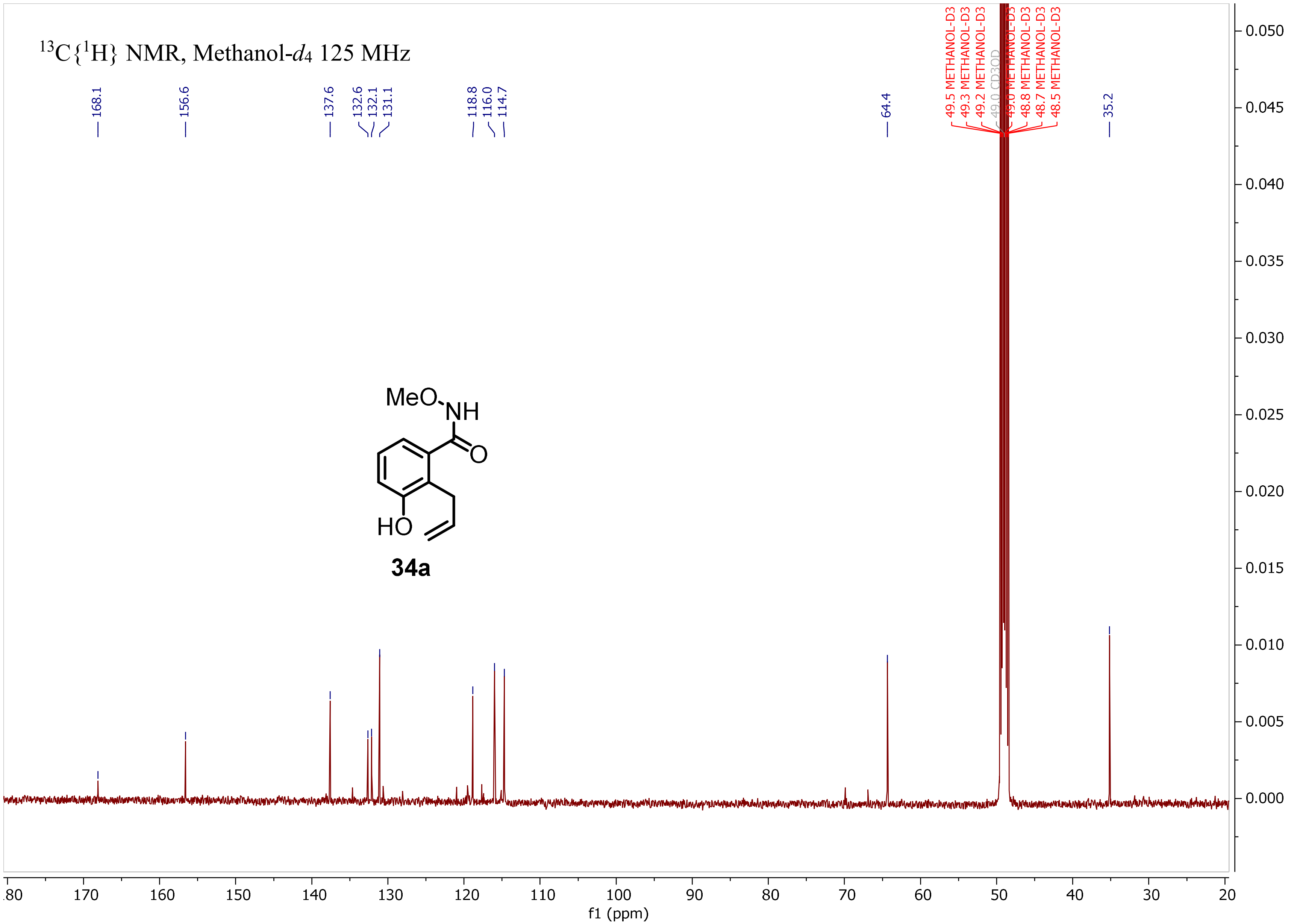

$^{13}C\{^1H\}$ NMR, Methanol-d_4 125 MHz
168.1
156.6
137.6
132.6
132.1
131.1
118.8
116.0
114.7
64.4
49.5 METHANOL-D3
49.3 METHANOL-D3
49.2 METHANOL-D3
49.0 CD3OD
49.0 METHANOL-D3
48.8 METHANOL-D3
48.7 METHANOL-D3
48.5 METHANOL-D3
35.2
MeO
NH
O
HO
34a
f1 (ppm)

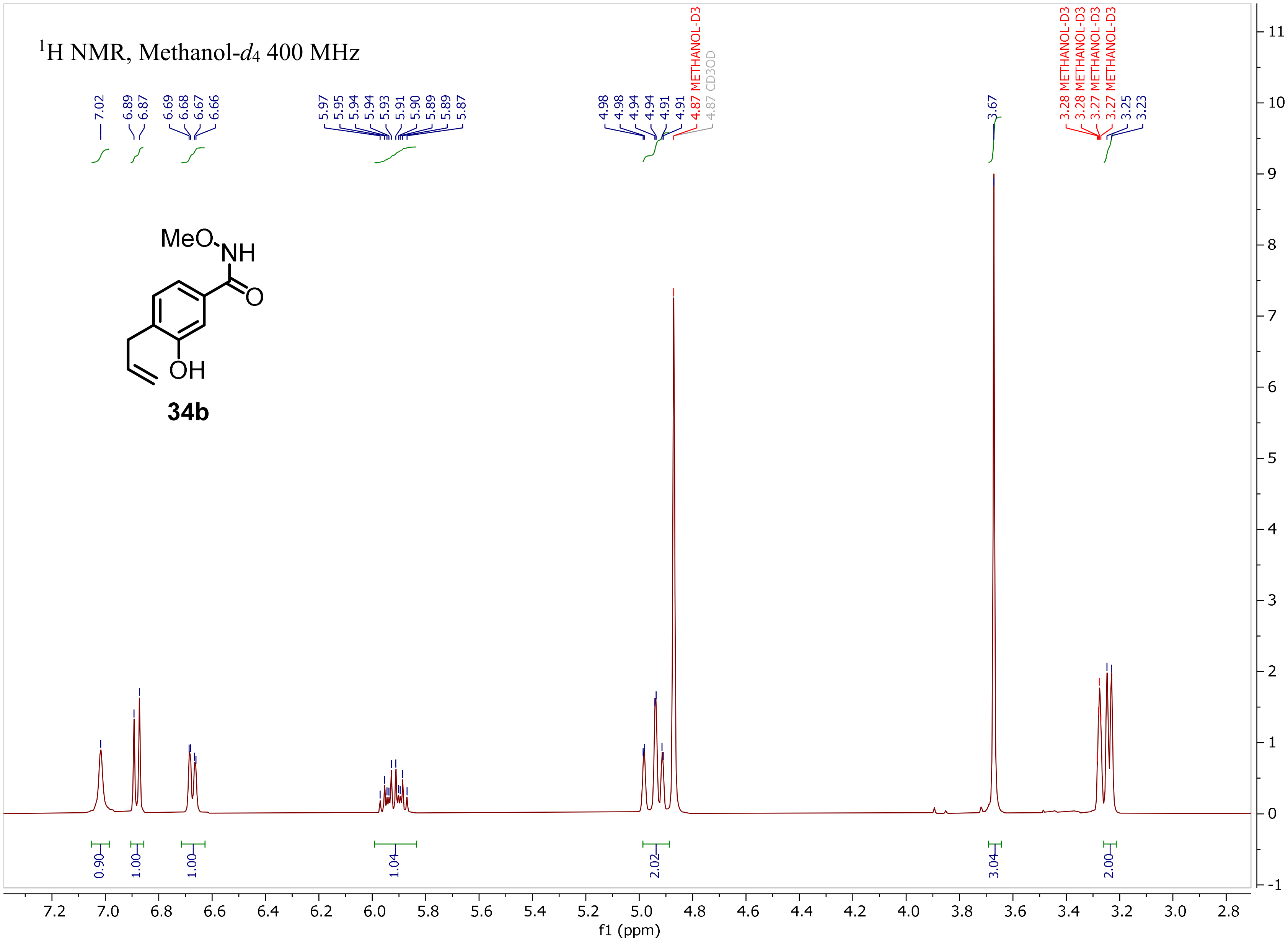

^{1}H NMR, Methanol-d4 400 MHz
MeO
NH
O
OH
34b
7.02
6.89
6.87
6.69
6.68
6.67
6.66
5.97
5.95
5.94
5.94
5.93
5.91
5.90
5.89
5.89
5.87
4.98
4.98
4.94
4.94
4.91
4.91
4.87 METHANOL-D3
4.87 CD3OD
3.67
3.28 METHANOL-D3
3.28 METHANOL-D3
3.27 METHANOL-D3
3.27 METHANOL-D3
3.25
3.23
0.90
1.00
1.00
1.04
2.02
3.04
2.00
f1 (ppm)

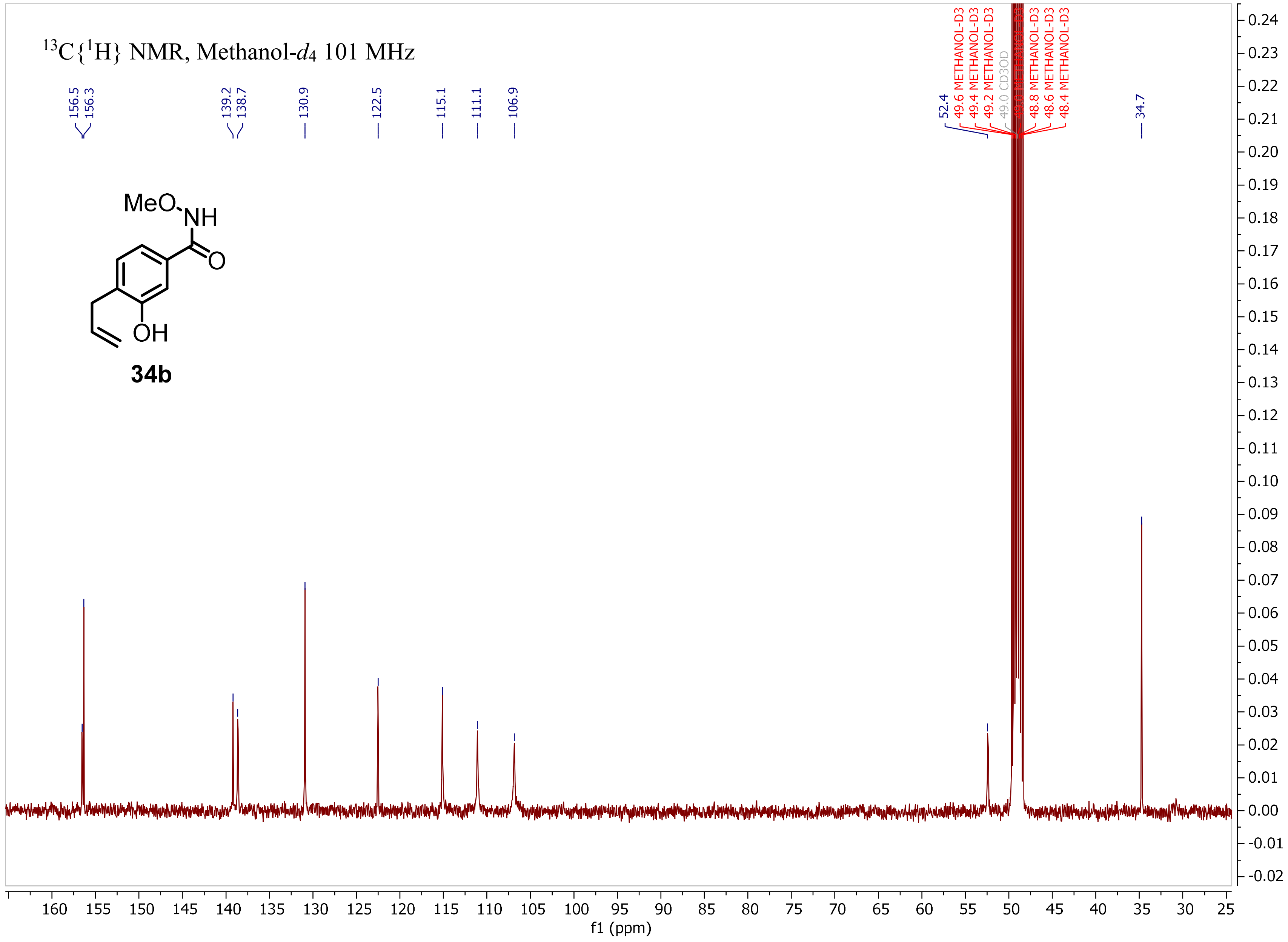

¹³C{¹H} NMR, Methanol-d4 101 MHz
156.5
156.3
139.2
138.7
130.9
122.5
115.1
111.1
106.9
52.4
49.6 METHANOL-D3
49.4 METHANOL-D3
49.2 METHANOL-D3
49.0 CD3OD
48.8 METHANOL-D3
48.6 METHANOL-D3
48.4 METHANOL-D3
34.7
MeO
NH
O
OH
34b
f1 (ppm)

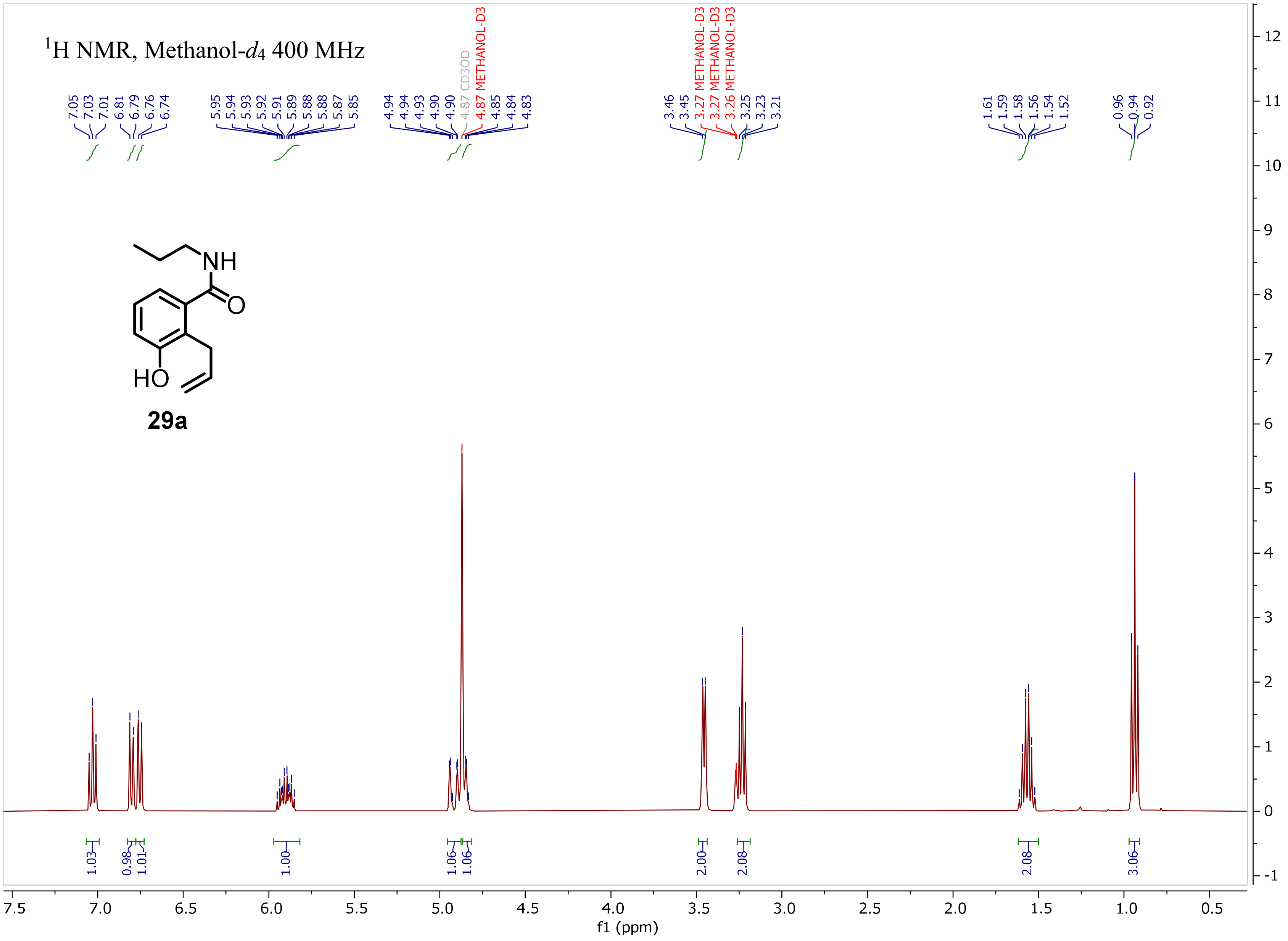

¹H NMR, Methanol-d4 400 MHz
7.05
7.03
7.01
6.81
6.79
6.76
6.74
5.95
5.94
5.93
5.92
5.91
5.89
5.88
5.88
5.87
5.85
4.94
4.94
4.93
4.90
4.90
4.87 CD3OD
4.87 METHANOL-D3
4.85
4.84
4.83
3.46
3.45
3.27 METHANOL-D3
3.27 METHANOL-D3
3.26 METHANOL-D3
3.25
3.23
3.21
1.61
1.59
1.58
1.56
1.54
1.52
0.96
0.94
0.92
NH
O
HO
29a
1.03
0.98
1.01
1.00
1.06
1.06
2.00
2.08
2.08
3.06
f1 (ppm)

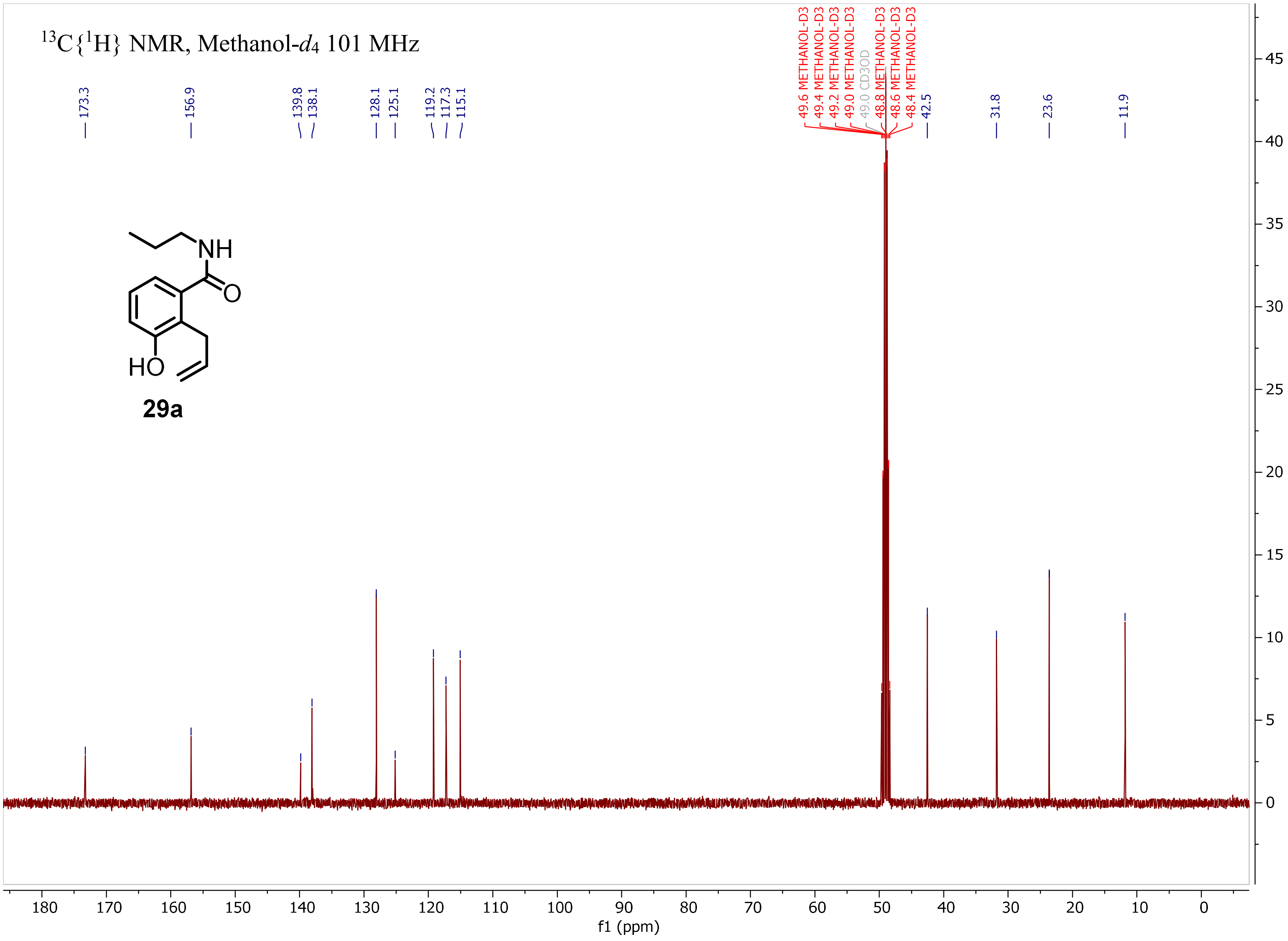

$^{13}C\{^{1}H\}$ NMR, Methanol-d_4 101 MHz
173.3
156.9
139.8
138.1
128.1
125.1
119.2
117.3
115.1
49.6 METHANOL-D3
49.4 METHANOL-D3
49.2 METHANOL-D3
49.0 METHANOL-D3
49.0 CD3OD
48.8 METHANOL-D3
48.6 METHANOL-D3
48.4 METHANOL-D3
42.5
31.8
23.6
11.9
NH
O
HO
29a
f1 (ppm)

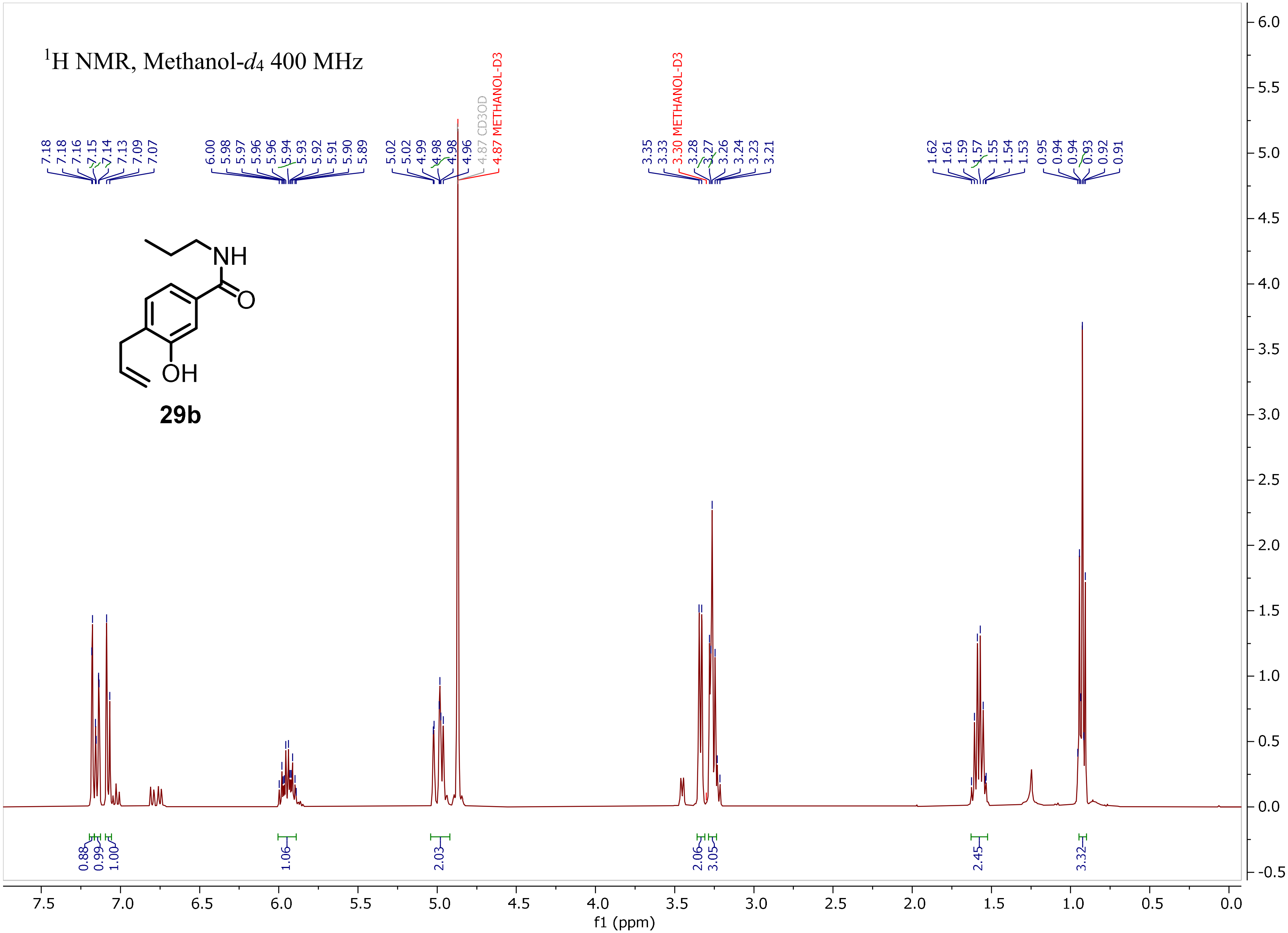

¹H NMR, Methanol-d4 400 MHz
NH
O
OH
29b
7.18 7.18 7.16 7.15 7.14 7.13 7.09 7.07
6.00 5.98 5.97 5.96 5.96 5.94 5.93 5.92 5.91 5.90 5.89
5.02 5.02 4.99 4.98 4.98 4.96 4.87 CD3OD 4.87 METHANOL-D3
3.35 3.33 3.30 METHANOL-D3 3.28 3.27 3.26 3.24 3.23 3.21
1.62 1.61 1.59 1.57 1.55 1.54 1.53 0.95 0.94 0.94 0.93 0.92 0.91
0.88 0.99 1.00 1.06 2.03 2.06 3.05 2.45 3.32
f1 (ppm)

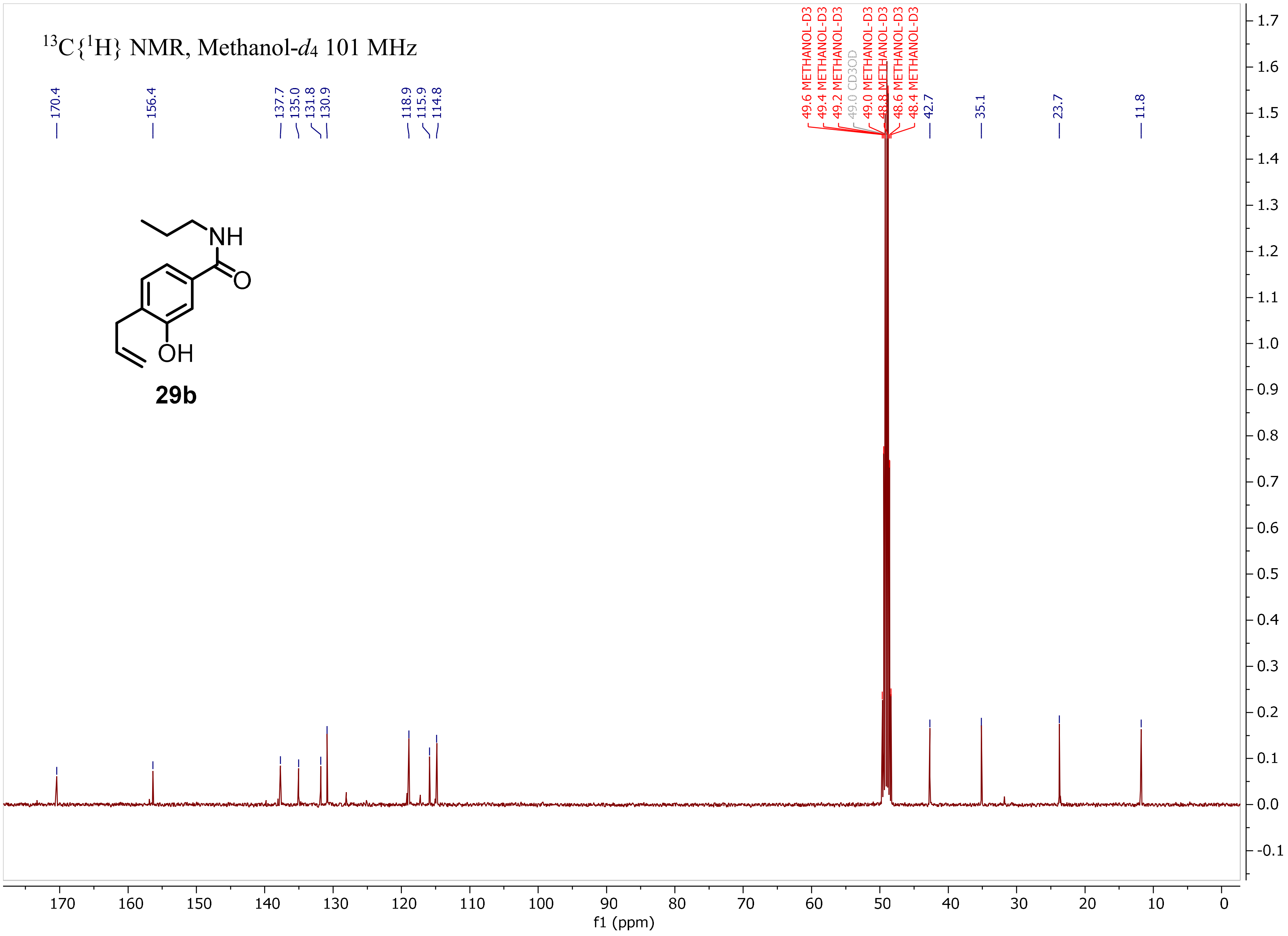

$^{13}C\{^1H\}$ NMR, Methanol-d_4 101 MHz
170.4
156.4
137.7
135.0
131.8
130.9
118.9
115.9
114.8
49.6 METHANOL-D3
49.4 METHANOL-D3
49.2 METHANOL-D3
49.0 CD3OD
49.0 METHANOL-D3
48.8 METHANOL-D3
48.6 METHANOL-D3
48.4 METHANOL-D3
42.7
35.1
23.7
11.8
NH
O
OH
29b
f1 (ppm)

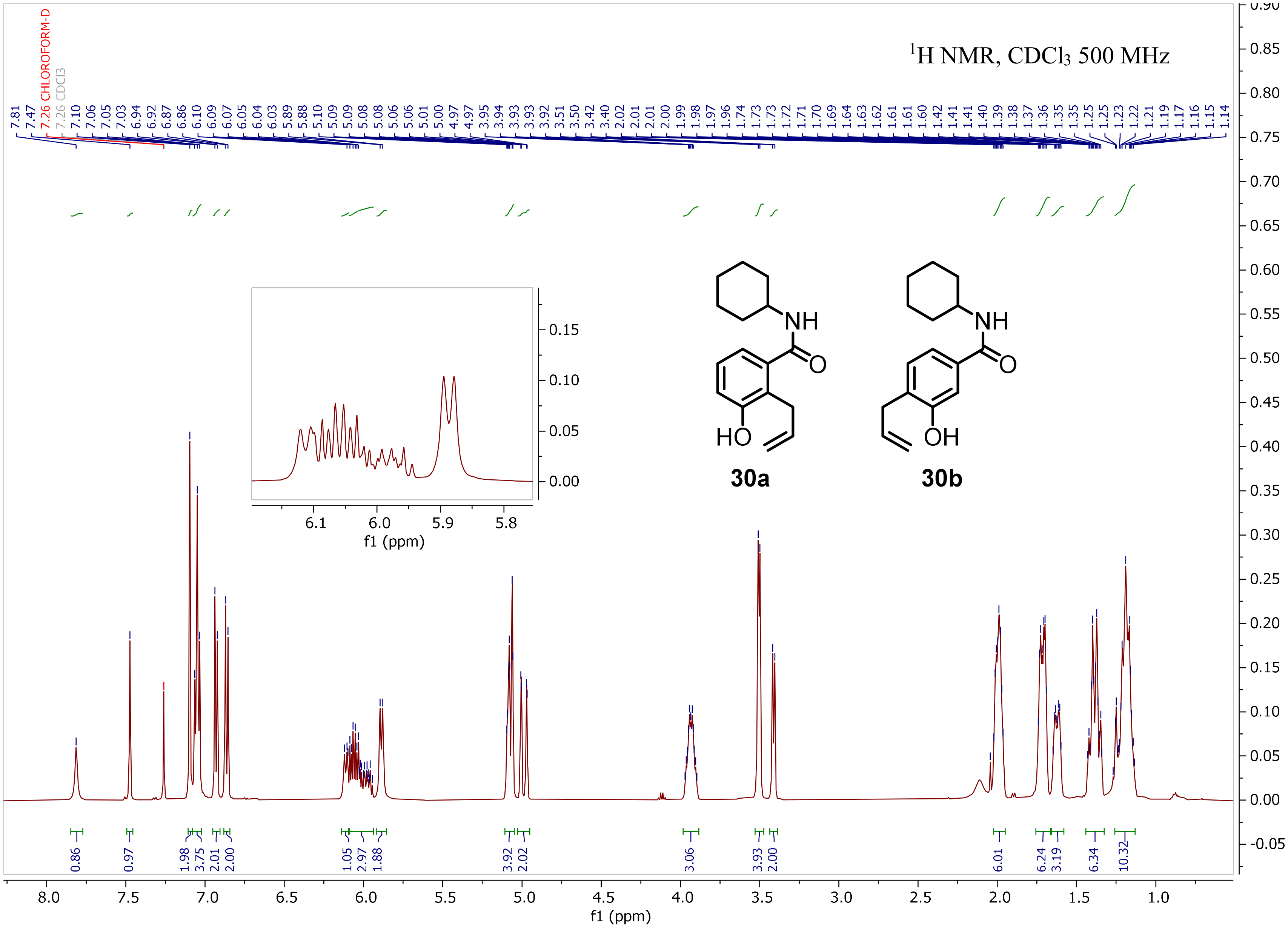

^{1}H NMR, CDCl3 500 MHz
30a
30b
f1 (ppm)

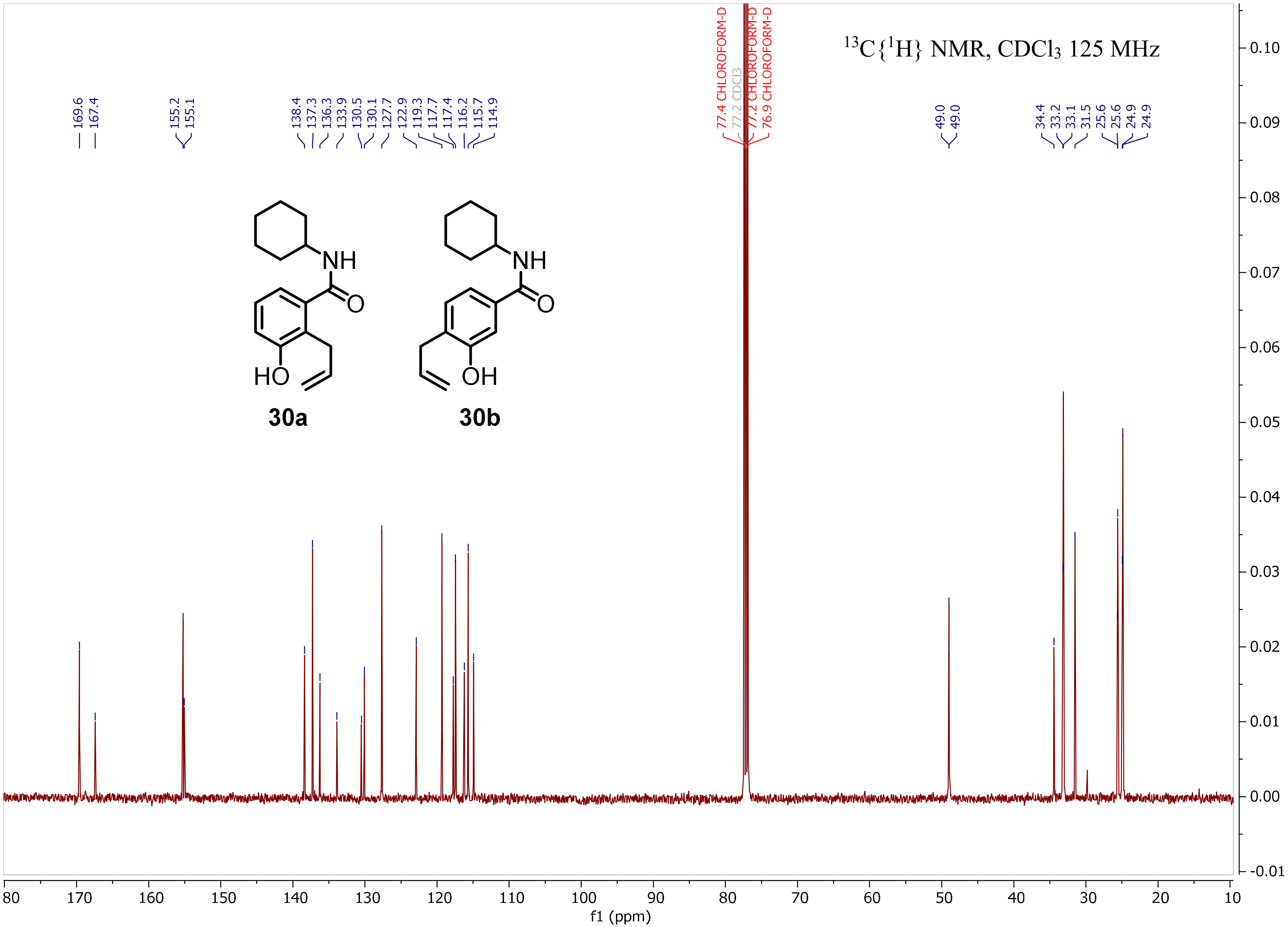

$^{13}C\{^1H\}$ NMR, $CDCl_3$ 125 MHz
169.6
167.4
155.2
155.1
138.4
137.3
136.3
133.9
130.5
130.1
127.7
122.9
119.3
117.7
117.4
116.2
115.7
114.9
77.4 CHLOROFORM-D
77.2 CDCl3
77.2 CHLOROFORM-D
76.9 CHLOROFORM-D
49.0
49.0
34.4
33.2
33.1
31.5
25.6
25.6
24.9
24.9
NH
O
HO
OH
30a
30b
f1 (ppm)

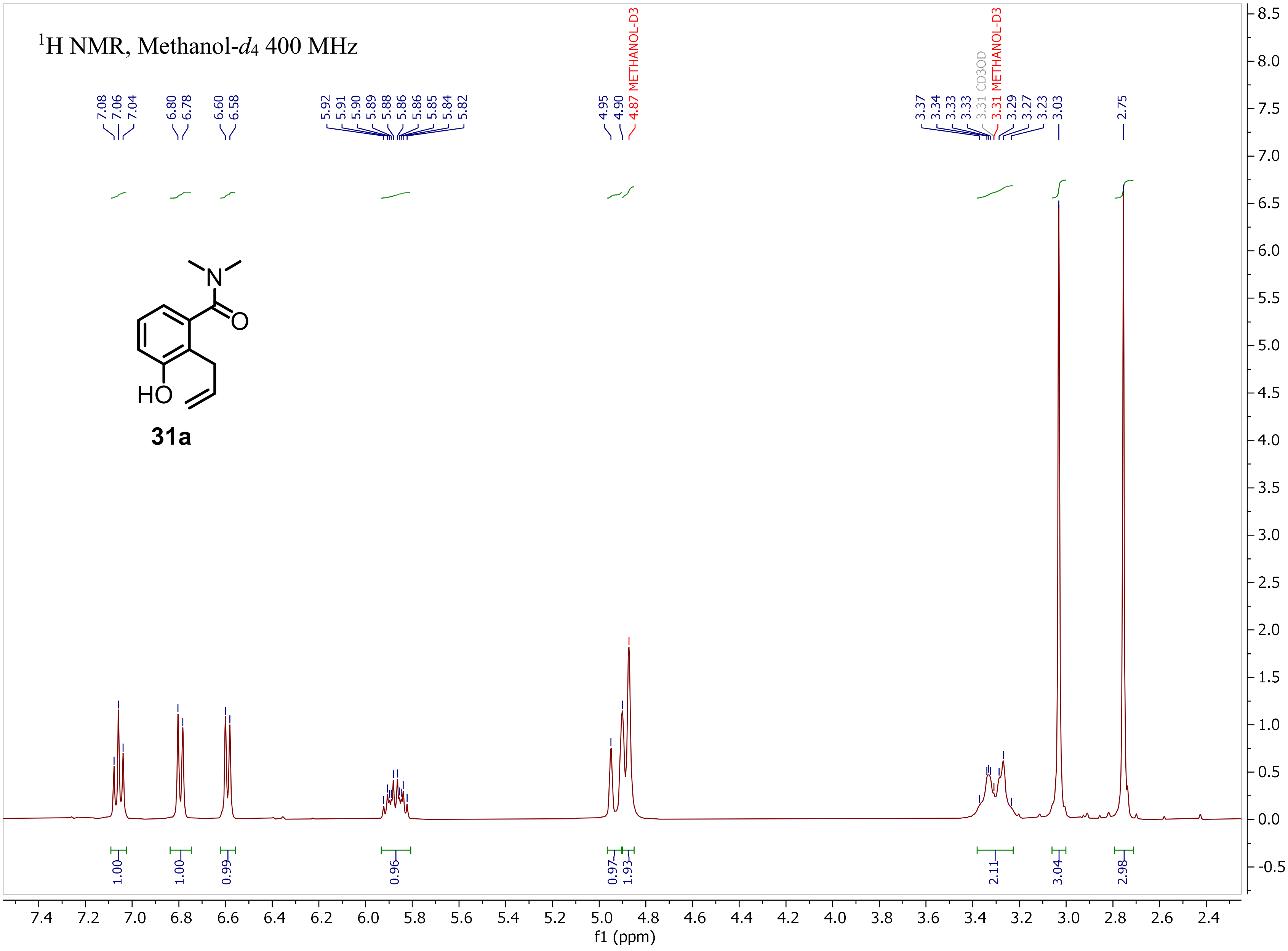

^{1}H NMR, Methanol-d_4 400 MHz
31a
f1 (ppm)

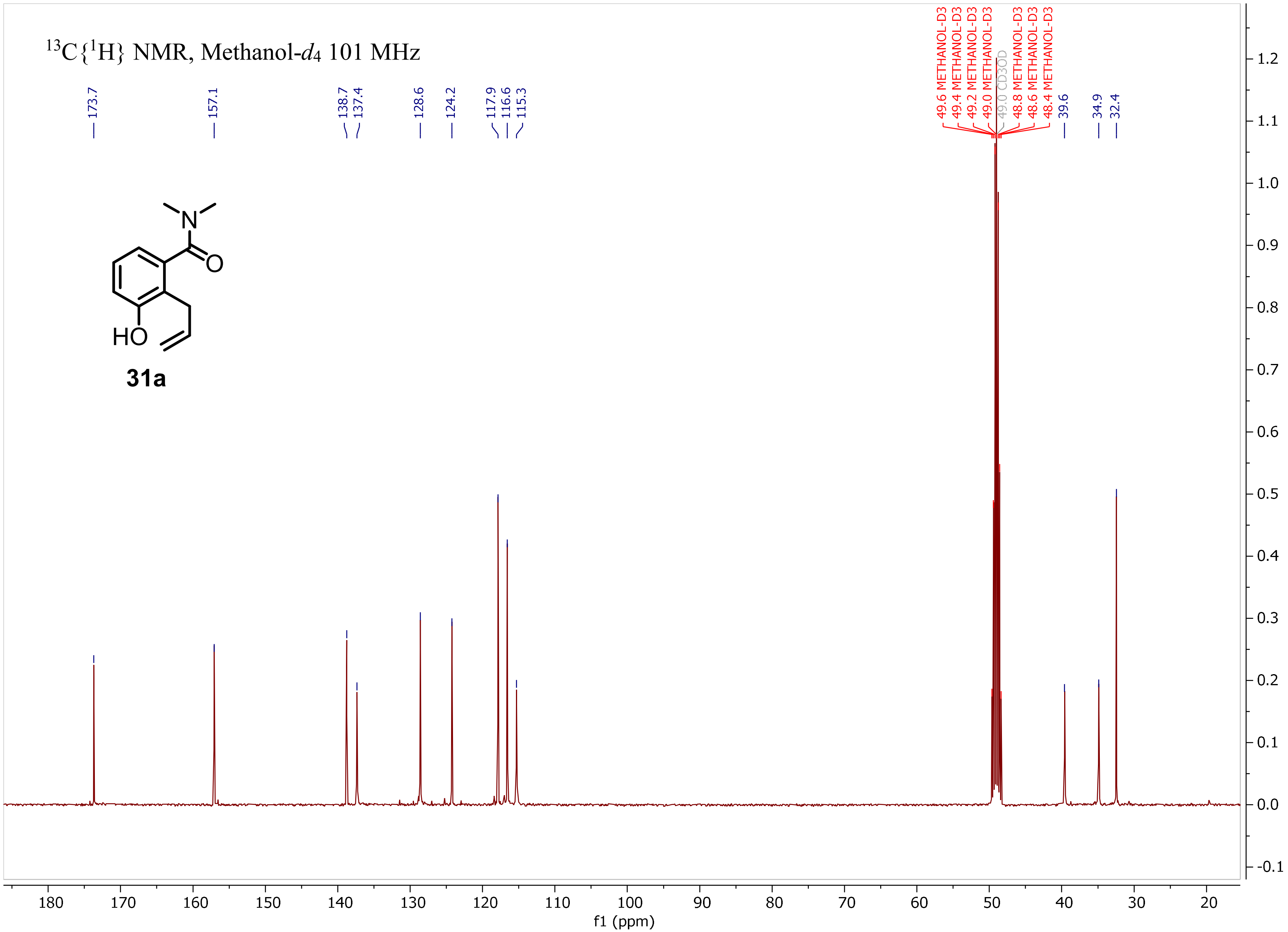
$^{13}C\{^{1}H\}$ NMR, Methanol-d_4 101 MHz
173.7
157.1
138.7
137.4
128.6
124.2
117.9
116.6
115.3
49.6 METHANOL-D3
49.4 METHANOL-D3
49.2 METHANOL-D3
49.0 METHANOL-D3
49.0 CD3OD
48.8 METHANOL-D3
48.6 METHANOL-D3
48.4 METHANOL-D3
39.6
34.9
32.4
HO
N
O
31a
f1 (ppm)

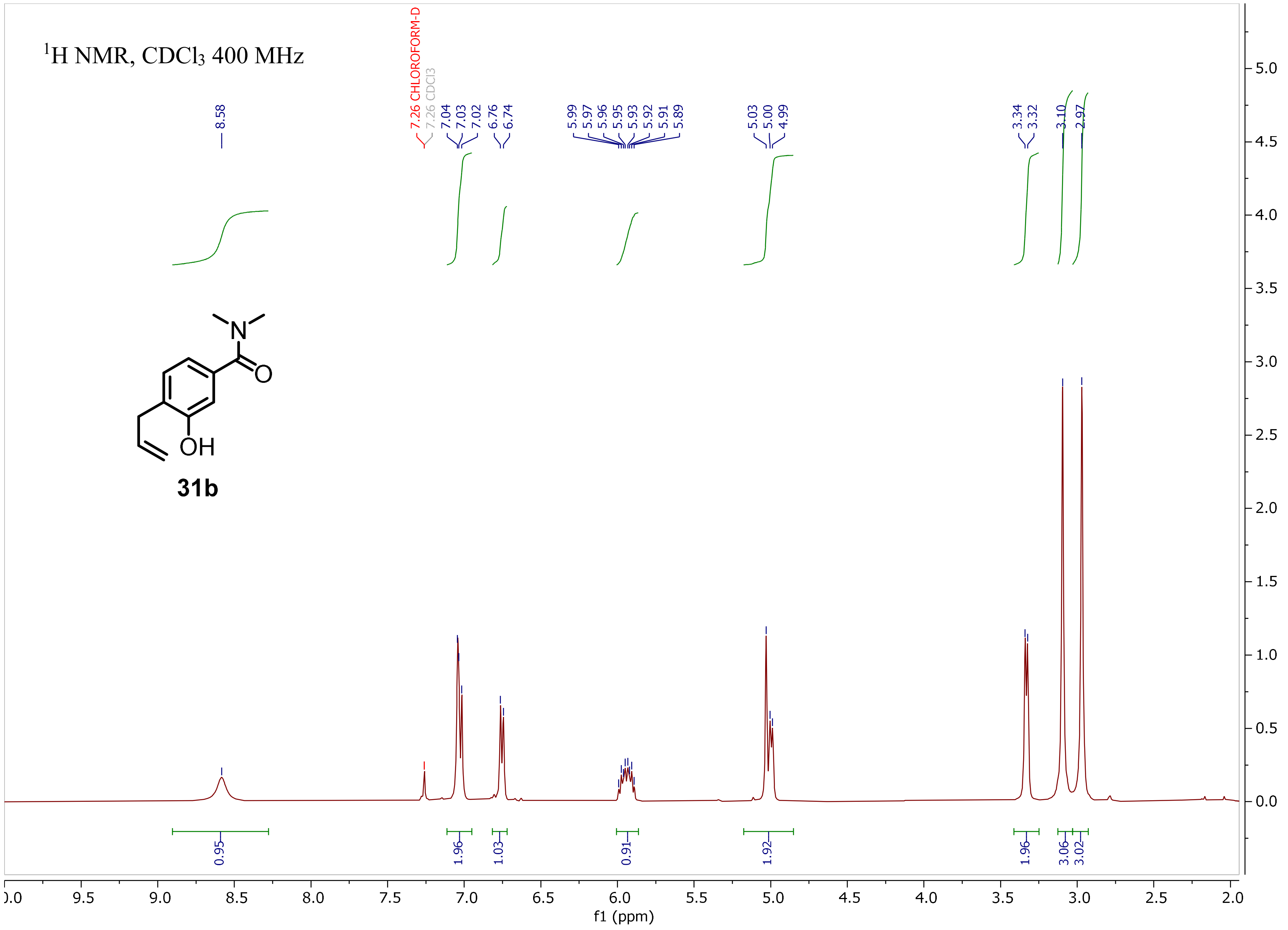

¹H NMR, CDCl₃ 400 MHz
7.26 CHLOROFORM-D
7.26 CDCl3
8.58
7.04
7.03
7.02
6.76
6.74
5.99
5.97
5.96
5.95
5.93
5.92
5.91
5.89
5.03
5.00
4.99
3.34
3.32
3.10
2.97
N
O
OH
31b
0.95
1.96
1.03
0.91
1.92
1.96
3.06
3.02
f1 (ppm)

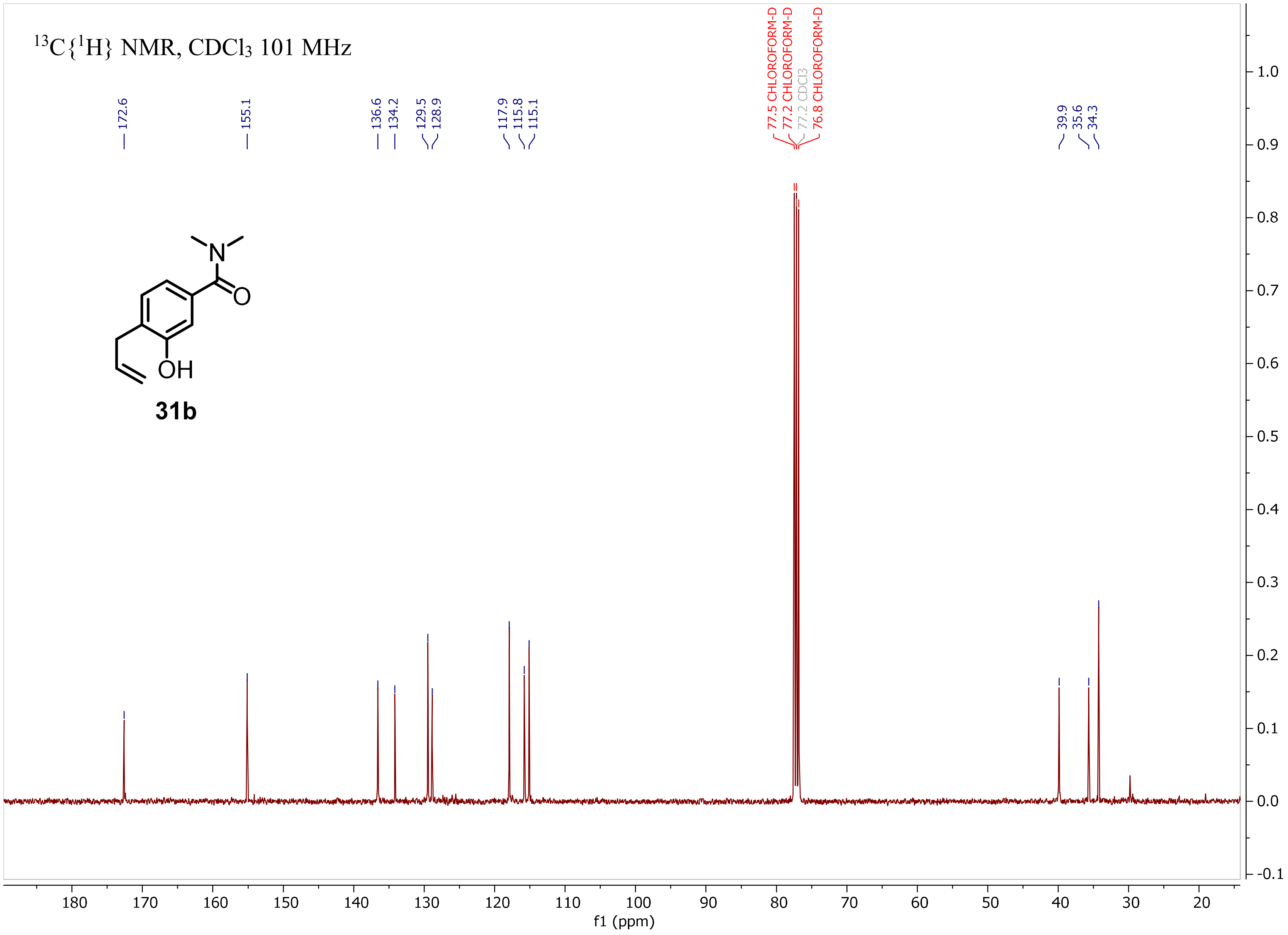
$^{13}C\{^{1}H\}$ NMR, $CDCl_3$ 101 MHz
172.6
155.1
136.6
134.2
129.5
128.9
117.9
115.8
115.1
77.5 CHLOROFORM-D
77.2 CHLOROFORM-D
77.2 CDCl3
76.8 CHLOROFORM-D
39.9
35.6
34.3
N
O
OH
31b
f1 (ppm)

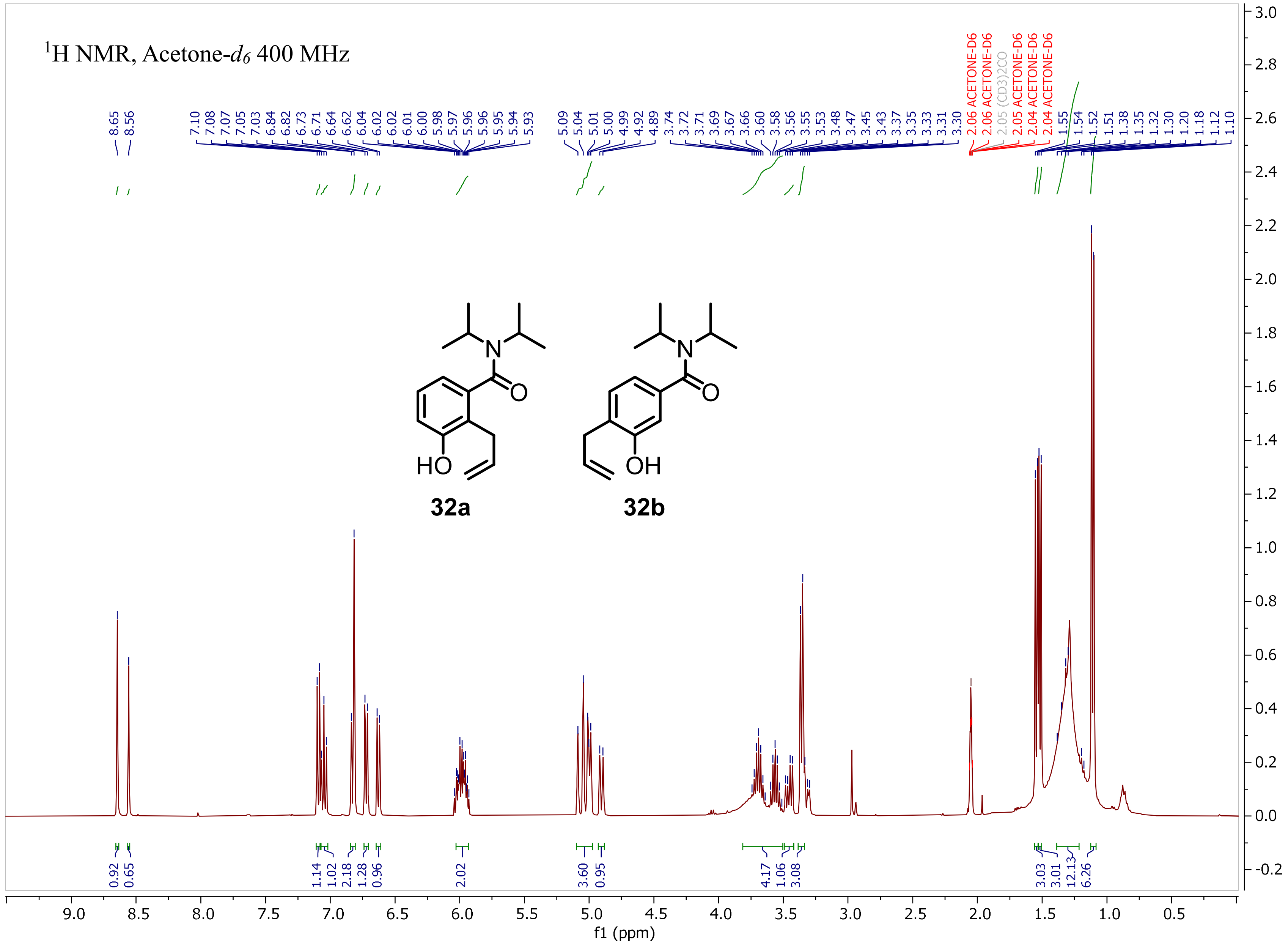

¹H NMR, Acetone-d6 400 MHz
32a
32b
f1 (ppm)

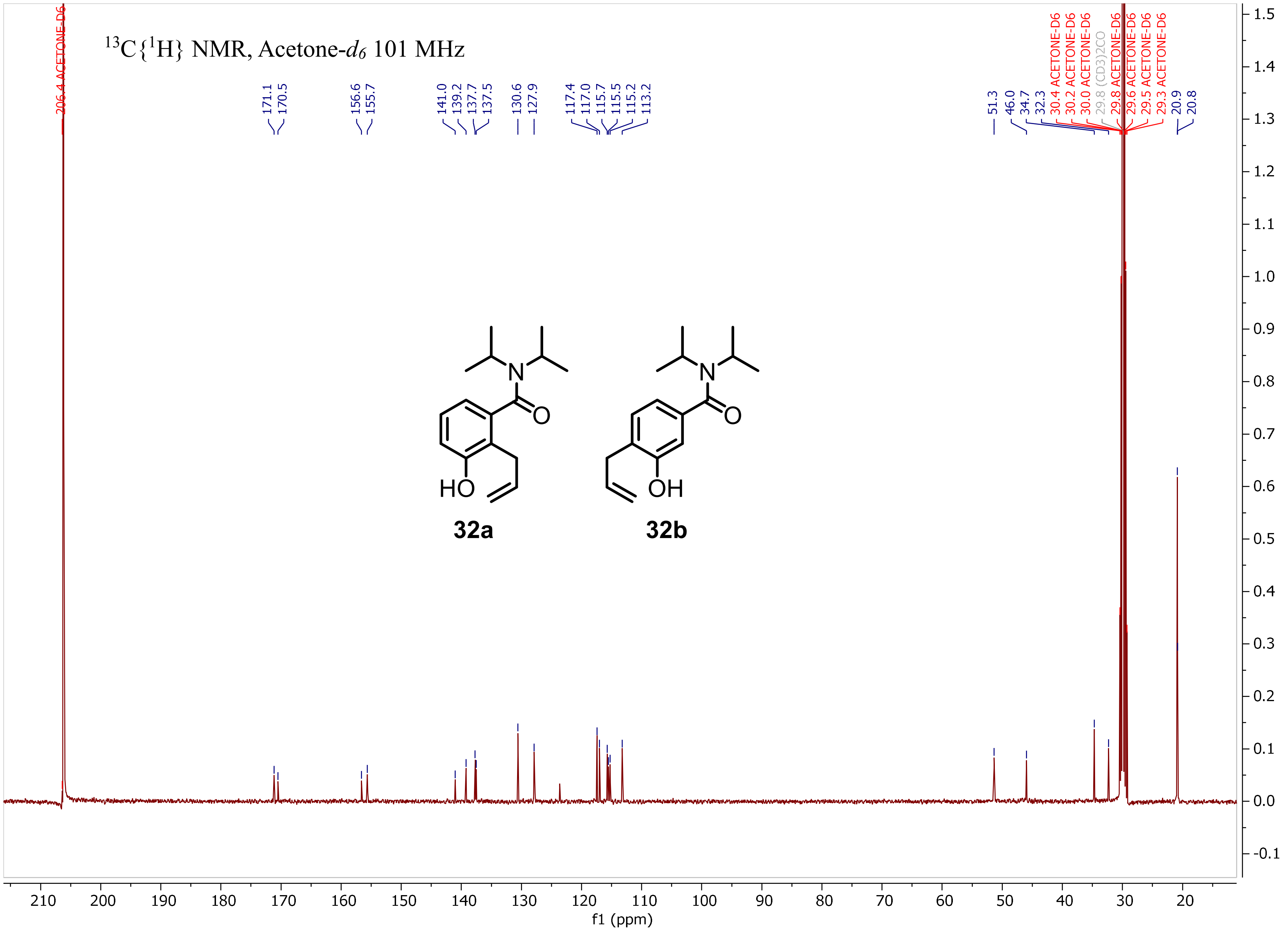

$^{13}C\{^1H\}$ NMR, Acetone-d_6 101 MHz
206.4 ACETONE-D6
171.1
170.5
156.6
155.7
141.0
139.2
137.7
137.5
130.6
127.9
117.4
117.0
115.7
115.5
115.2
113.2
51.3
46.0
34.7
32.3
30.4 ACETONE-D6
30.2 ACETONE-D6
30.0 ACETONE-D6
29.8 (CD3)2CO
29.8 ACETONE-D6
29.6 ACETONE-D6
29.5 ACETONE-D6
29.3 ACETONE-D6
20.9
20.8
N
O
HO
OH
32a
32b
f1 (ppm)

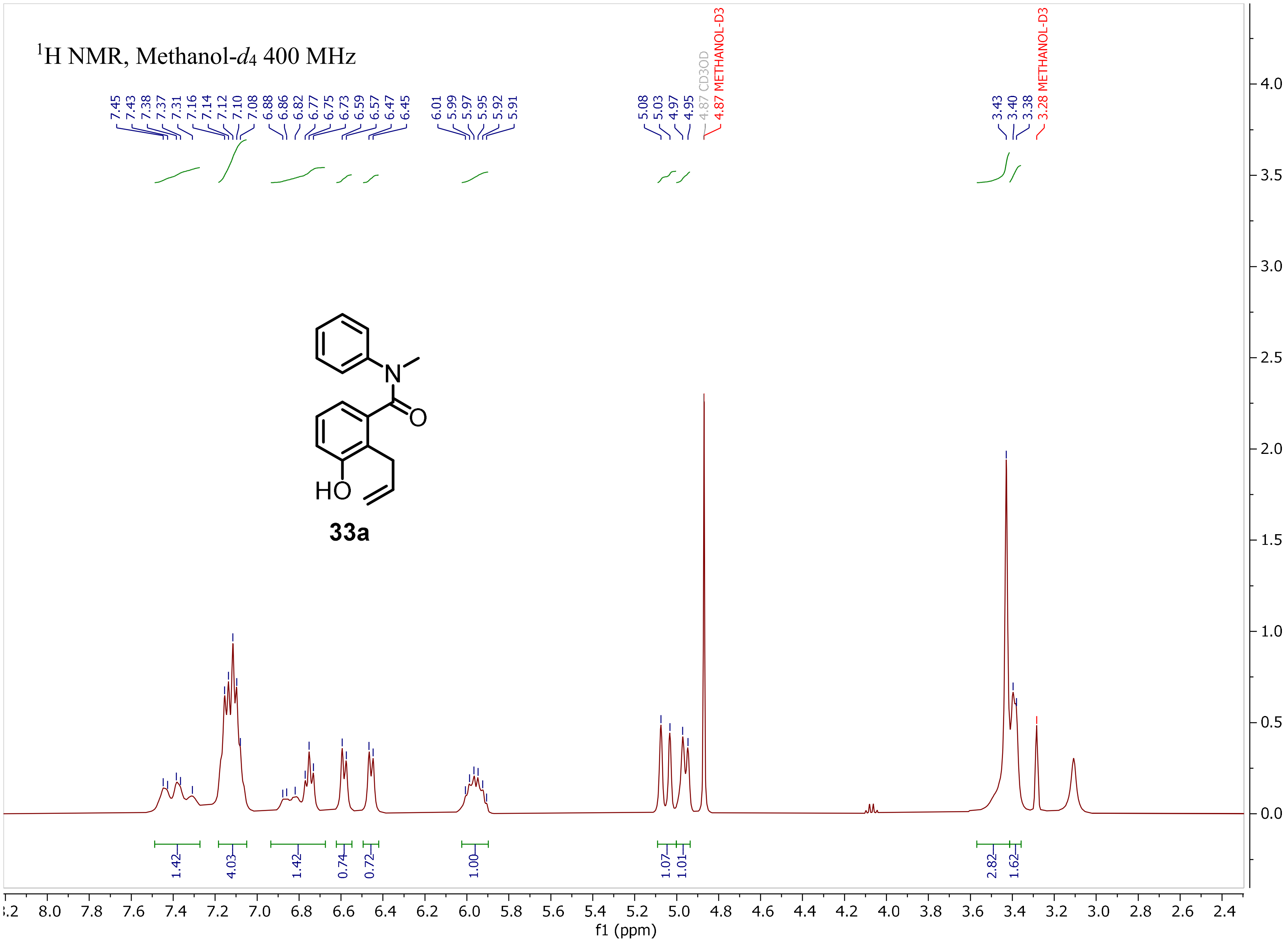

1H NMR, Methanol-d4 400 MHz
7.45
7.43
7.38
7.37
7.31
7.16
7.14
7.12
7.10
7.08
6.88
6.86
6.82
6.77
6.75
6.73
6.59
6.57
6.47
6.45
6.01
5.99
5.97
5.95
5.92
5.91
5.08
5.03
4.97
4.95
4.87 CD3OD
4.87 METHANOL-D3
3.43
3.40
3.38
3.28 METHANOL-D3
N
O
HO
33a
1.42
4.03
1.42
0.74
0.72
1.00
1.07
1.01
2.82
1.62
f1 (ppm)

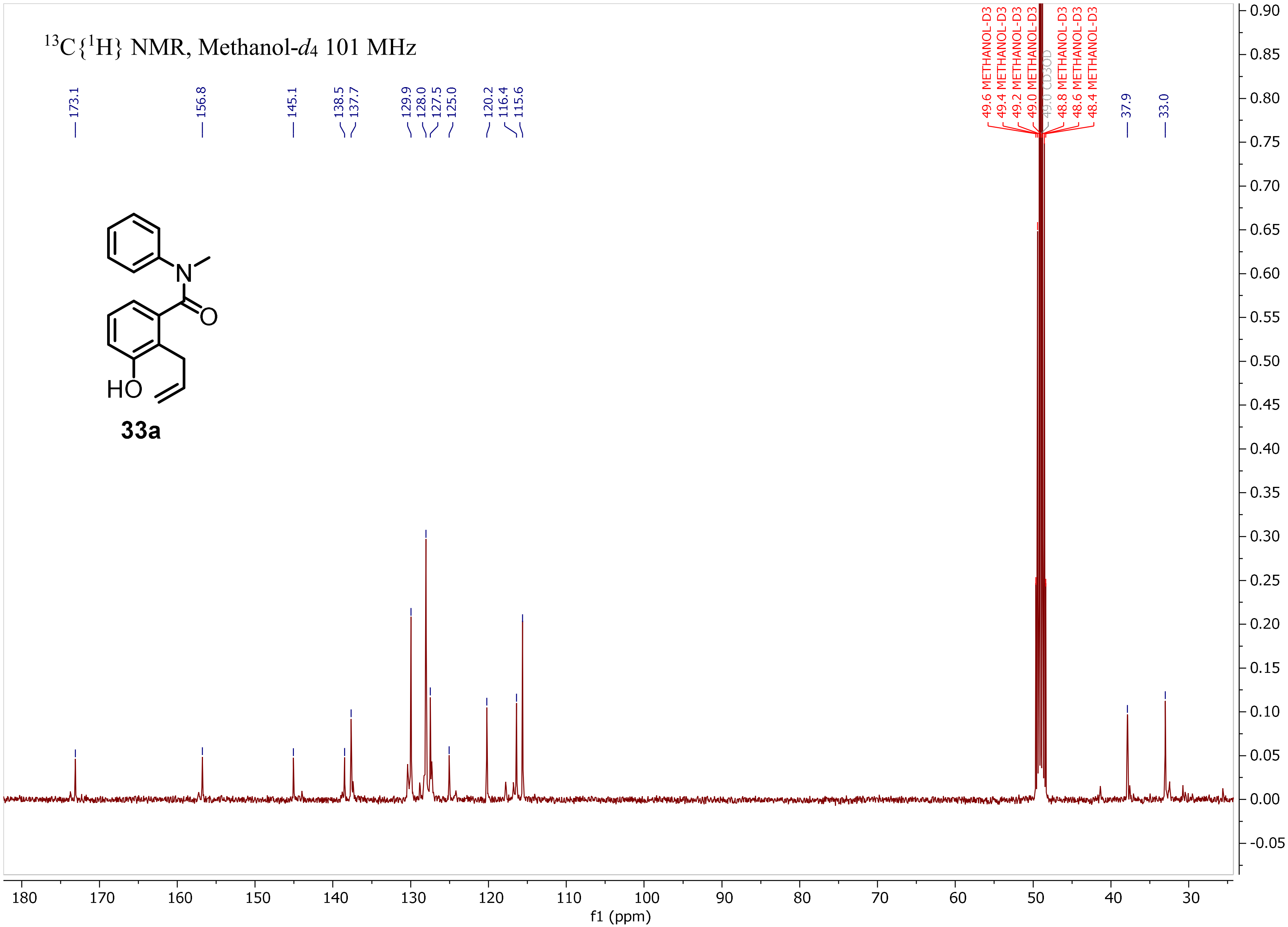

$^{13}C\{^{1}H\}$ NMR, Methanol-d_4 101 MHz
173.1
156.8
145.1
138.5
137.7
129.9
128.0
127.5
125.0
120.2
116.4
115.6
49.6 METHANOL-D3
49.4 METHANOL-D3
49.2 METHANOL-D3
49.0 METHANOL-D3
49.0 CD3OD
48.8 METHANOL-D3
48.6 METHANOL-D3
48.4 METHANOL-D3
37.9
33.0
N
O
HO
33a
f1 (ppm)

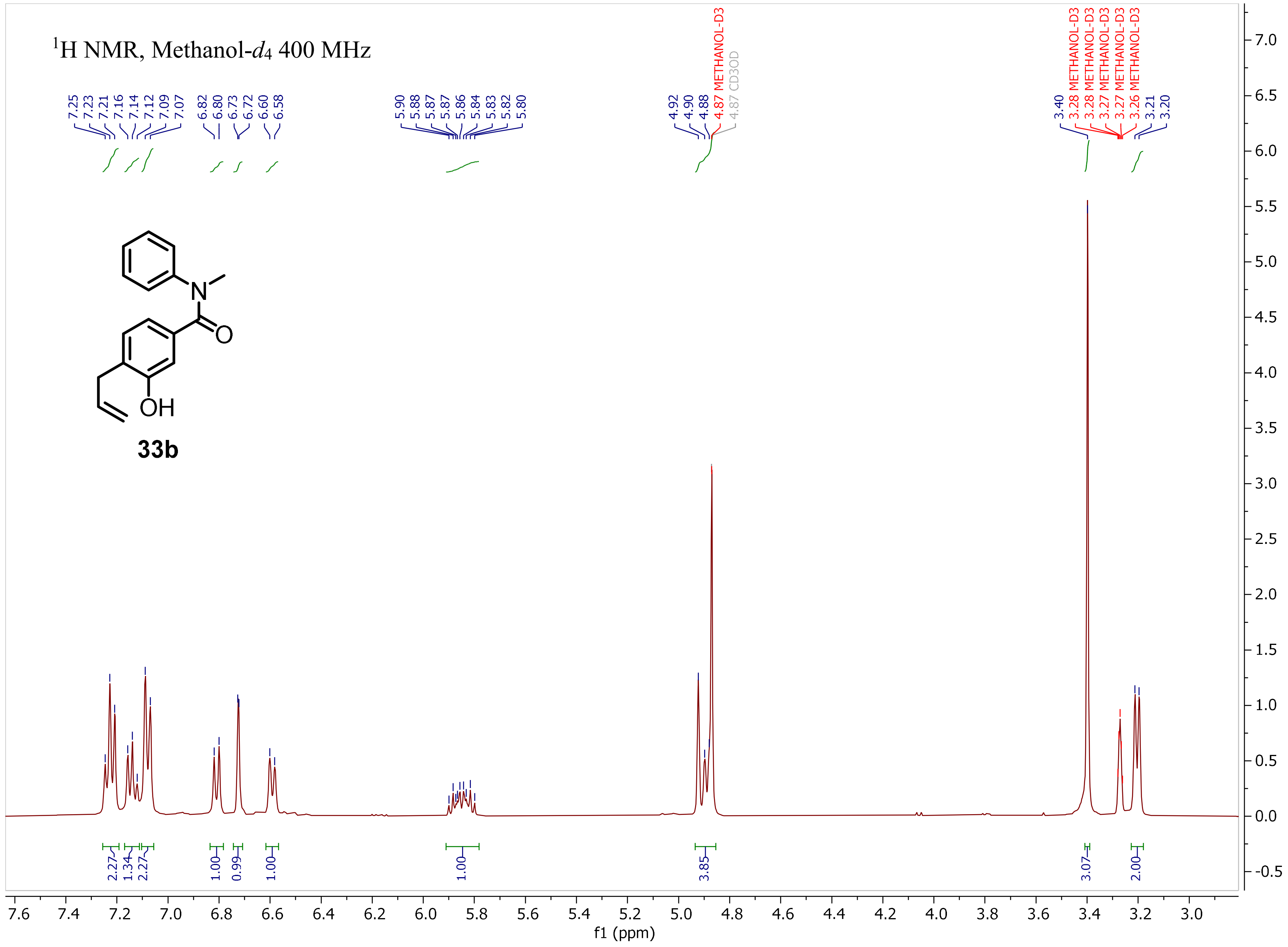

^{1}H NMR, Methanol-d_4 400 MHz
33b
OH
N
O
7.25 7.23 7.21 7.16 7.14 7.12 7.09 7.07 6.82 6.80 6.73 6.72 6.60 6.58
5.90 5.88 5.87 5.87 5.86 5.84 5.83 5.82 5.80
4.92 4.90 4.88 4.87 METHANOL-D3 4.87 CD3OD
3.40 3.28 METHANOL-D3 3.28 METHANOL-D3 3.27 METHANOL-D3 3.27 METHANOL-D3 3.26 METHANOL-D3 3.21 3.20
2.27 1.34 2.27 1.00 0.99 1.00 1.00 3.85 3.07 2.00
7.6 7.4 7.2 7.0 6.8 6.6 6.4 6.2 6.0 5.8 5.6 5.4 5.2 5.0 4.8 4.6 4.4 4.2 4.0 3.8 3.6 3.4 3.2 3.0
f1 (ppm)
7.0 6.5 6.0 5.5 5.0 4.5 4.0 3.5 3.0 2.5 2.0 1.5 1.0 0.5 0.0 -0.5

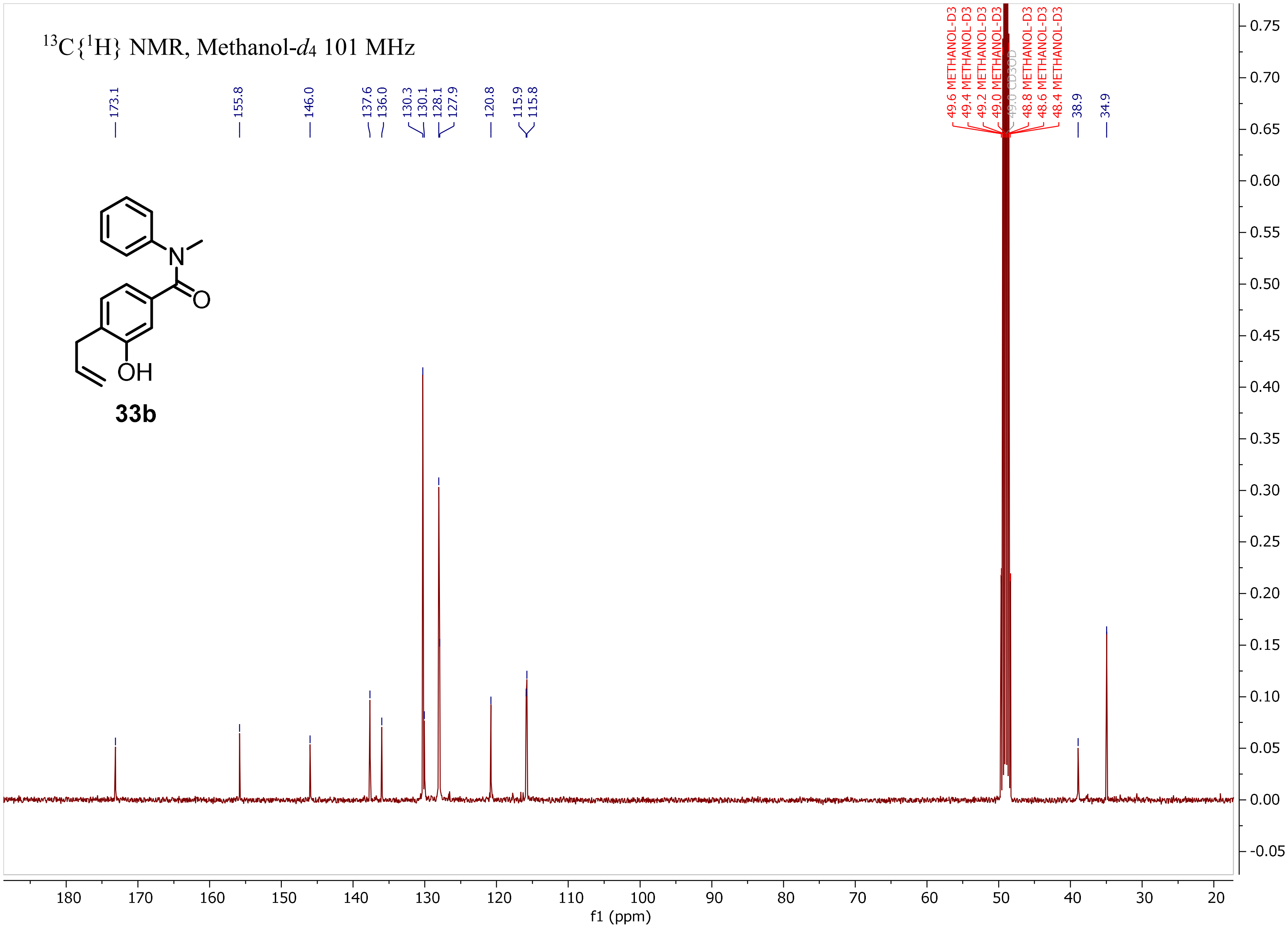

$^{13}C\{^{1}H\}$ NMR, Methanol-d_4 101 MHz
173.1
155.8
146.0
137.6
136.0
130.3
130.1
128.1
127.9
120.8
115.9
115.8
49.6 METHANOL-D3
49.4 METHANOL-D3
49.2 METHANOL-D3
49.0 METHANOL-D3
49.0 CD3OD
48.8 METHANOL-D3
48.6 METHANOL-D3
48.4 METHANOL-D3
38.9
34.9
N
O
OH
33b
f1 (ppm)

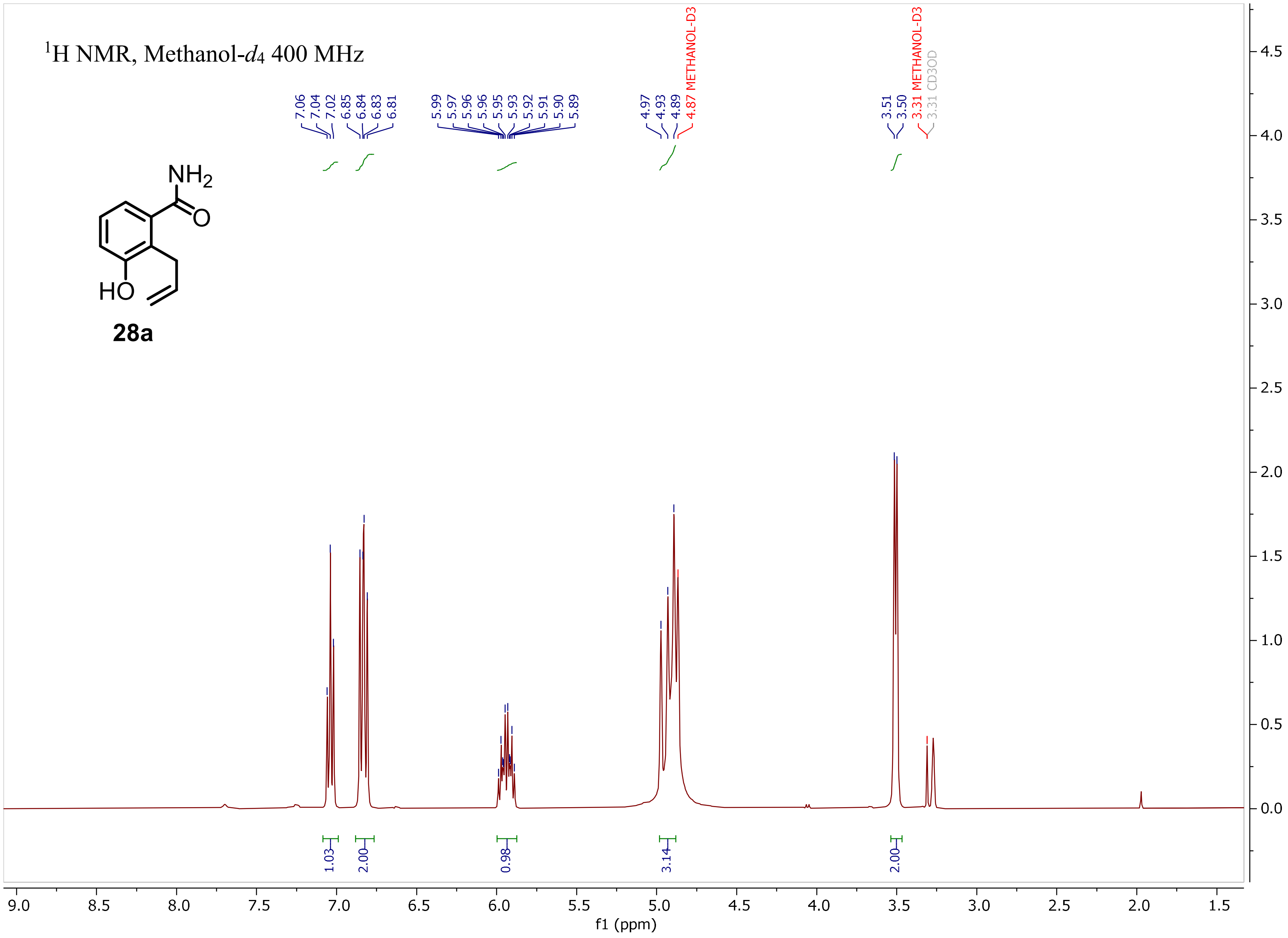

1H NMR, Methanol-d4 400 MHz
NH2
O
HO
28a
7.06
7.04
7.02
6.85
6.84
6.83
6.81
5.99
5.97
5.96
5.96
5.95
5.93
5.92
5.91
5.90
5.89
4.97
4.93
4.89
4.87 METHANOL-D3
3.51
3.50
3.31 METHANOL-D3
3.31 CD3OD
1.03
2.00
0.98
3.14
2.00
f1 (ppm)

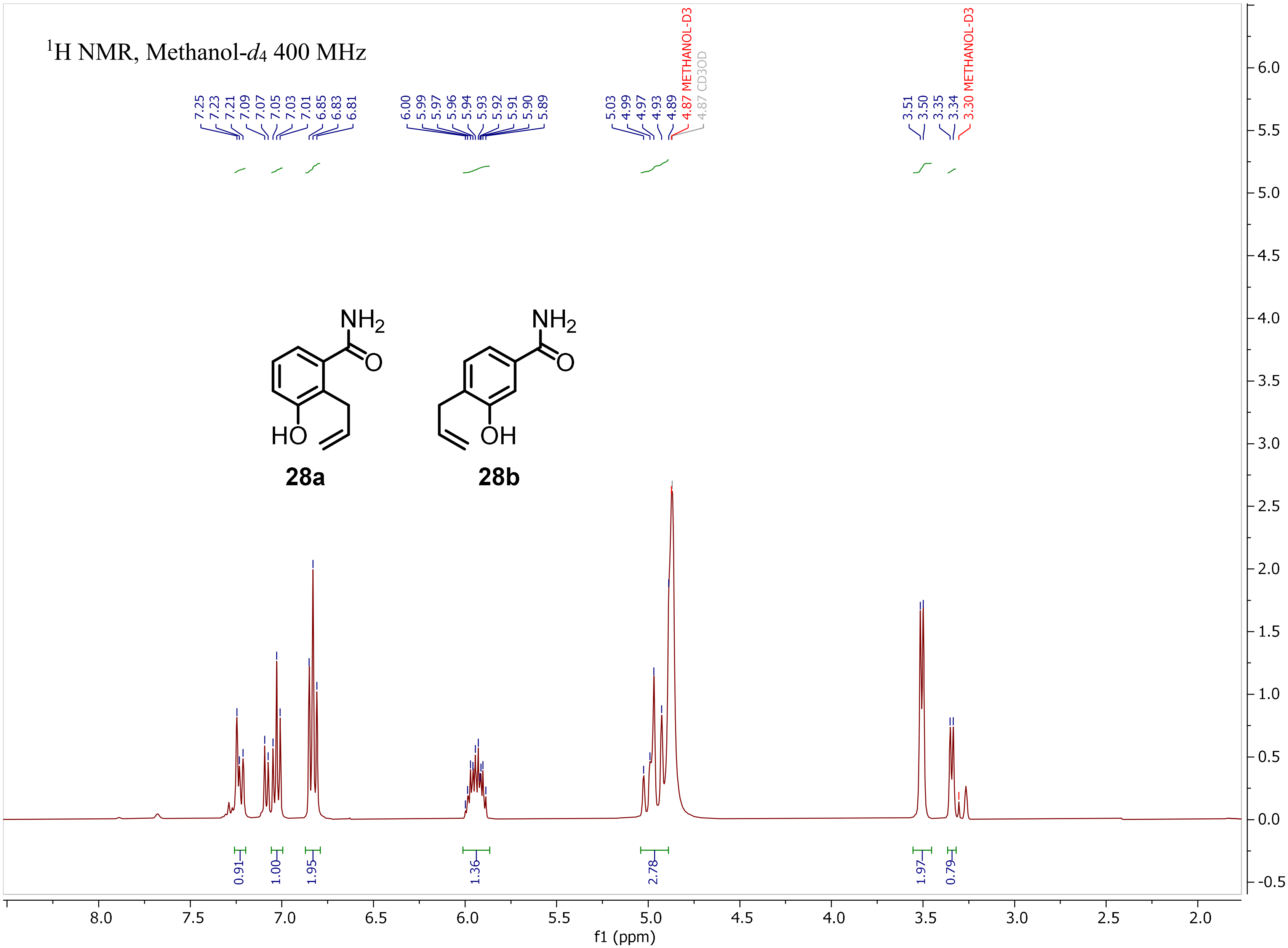

1H NMR, Methanol-d4 400 MHz
7.25 7.23 7.21 7.09 7.07 7.05 7.03 7.01 6.85 6.83 6.81
6.00 5.99 5.97 5.96 5.94 5.93 5.92 5.91 5.90 5.89
5.03 4.99 4.97 4.93 4.89
4.87 METHANOL-D3
4.87 CD3OD
3.51 3.50 3.35 3.34
3.30 METHANOL-D3
NH2
O
HO
OH
28a
28b
0.91 1.00 1.95 1.36 2.78 1.97 0.79
f1 (ppm)

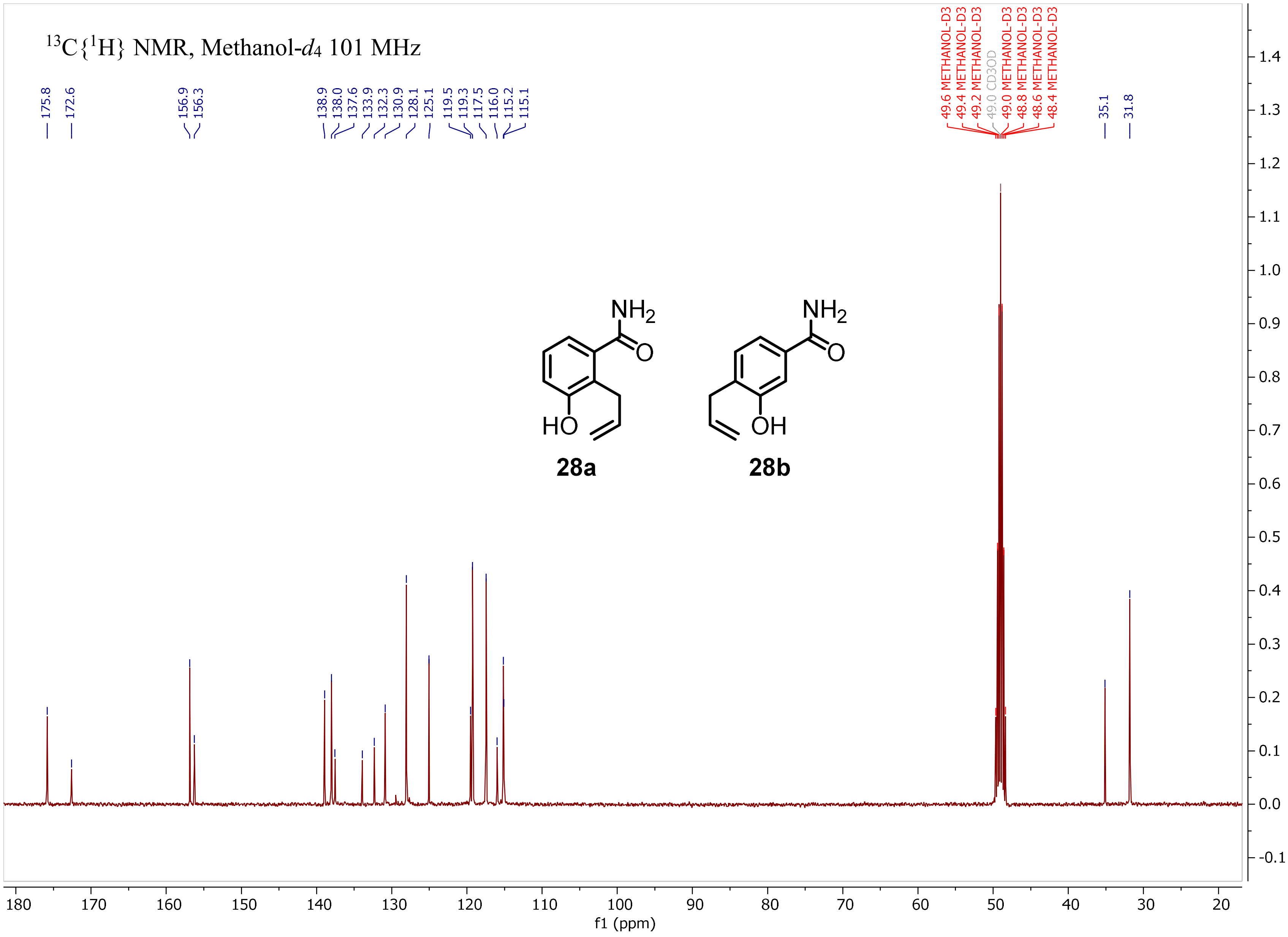

$^{13}C\{^{1}H\}$ NMR, Methanol-d_4 101 MHz
175.8
172.6
156.9
156.3
138.9
138.0
137.6
133.9
132.3
130.9
128.1
125.1
119.5
119.3
117.5
116.0
115.2
115.1
49.6 METHANOL-D3
49.4 METHANOL-D3
49.2 METHANOL-D3
49.0 CD3OD
49.0 METHANOL-D3
48.8 METHANOL-D3
48.6 METHANOL-D3
48.4 METHANOL-D3
35.1
31.8
NH2
O
HO
OH
28a
28b
f1 (ppm)

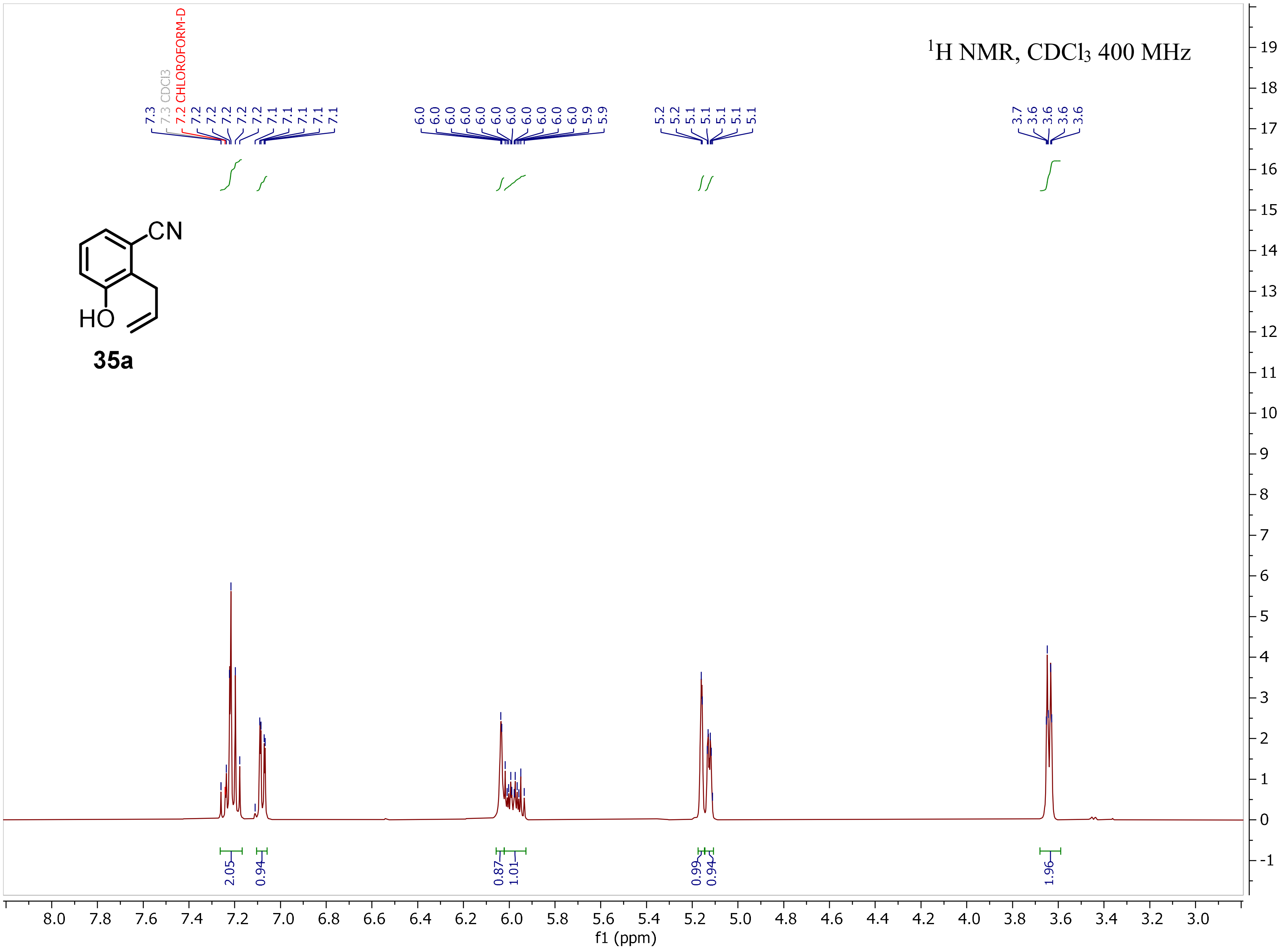

1H NMR, CDCl3 400 MHz
CN
HO
35a
f1 (ppm)

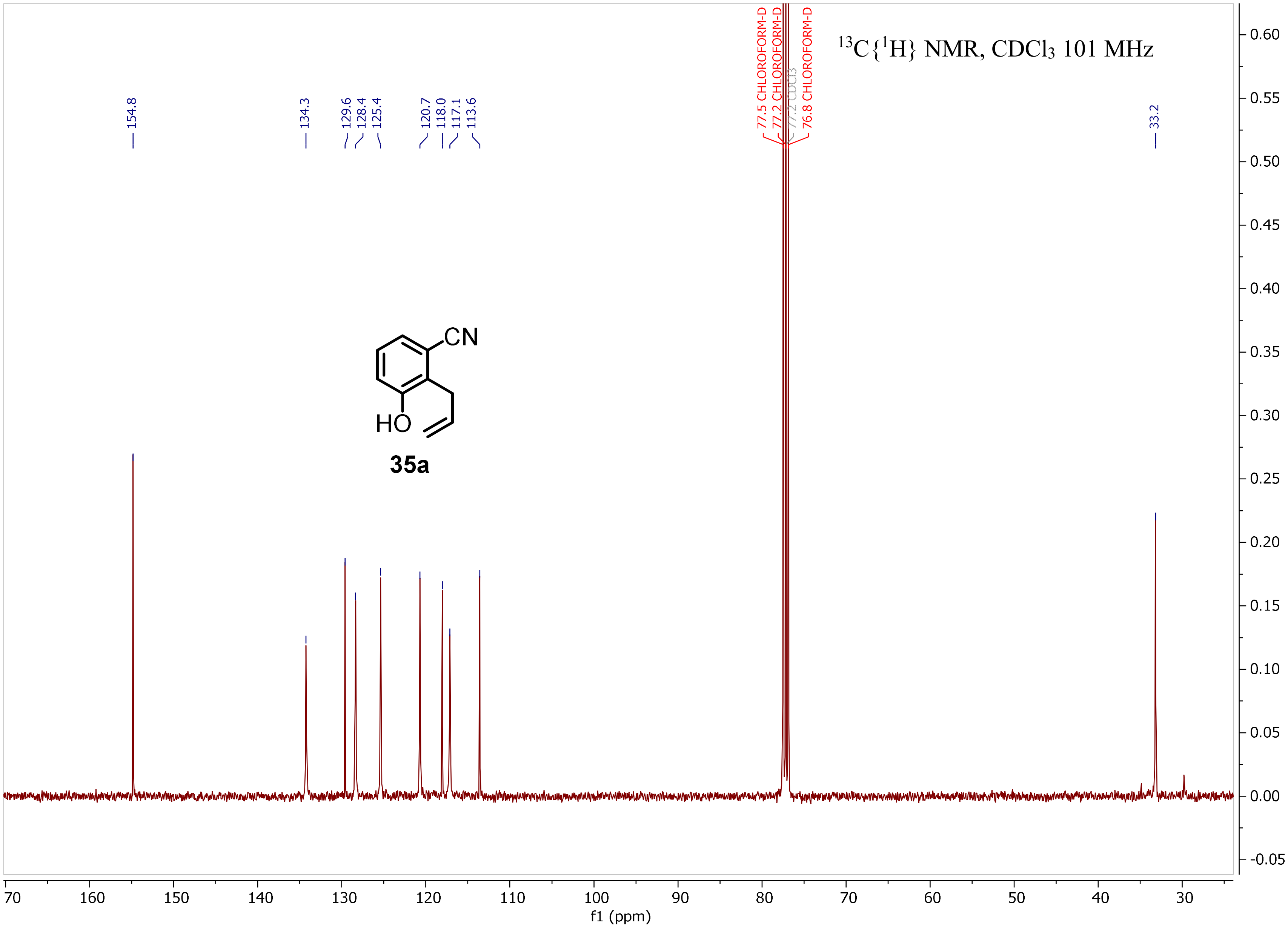

$^{13}C\{^1H\}$ NMR, $CDCl_3$ 101 MHz
154.8
134.3
129.6
128.4
125.4
120.7
118.0
117.1
113.6
77.5 CHLOROFORM-D
77.2 CHLOROFORM-D
77.2 CDCl3
76.8 CHLOROFORM-D
33.2
CN
HO
35a
f1 (ppm)

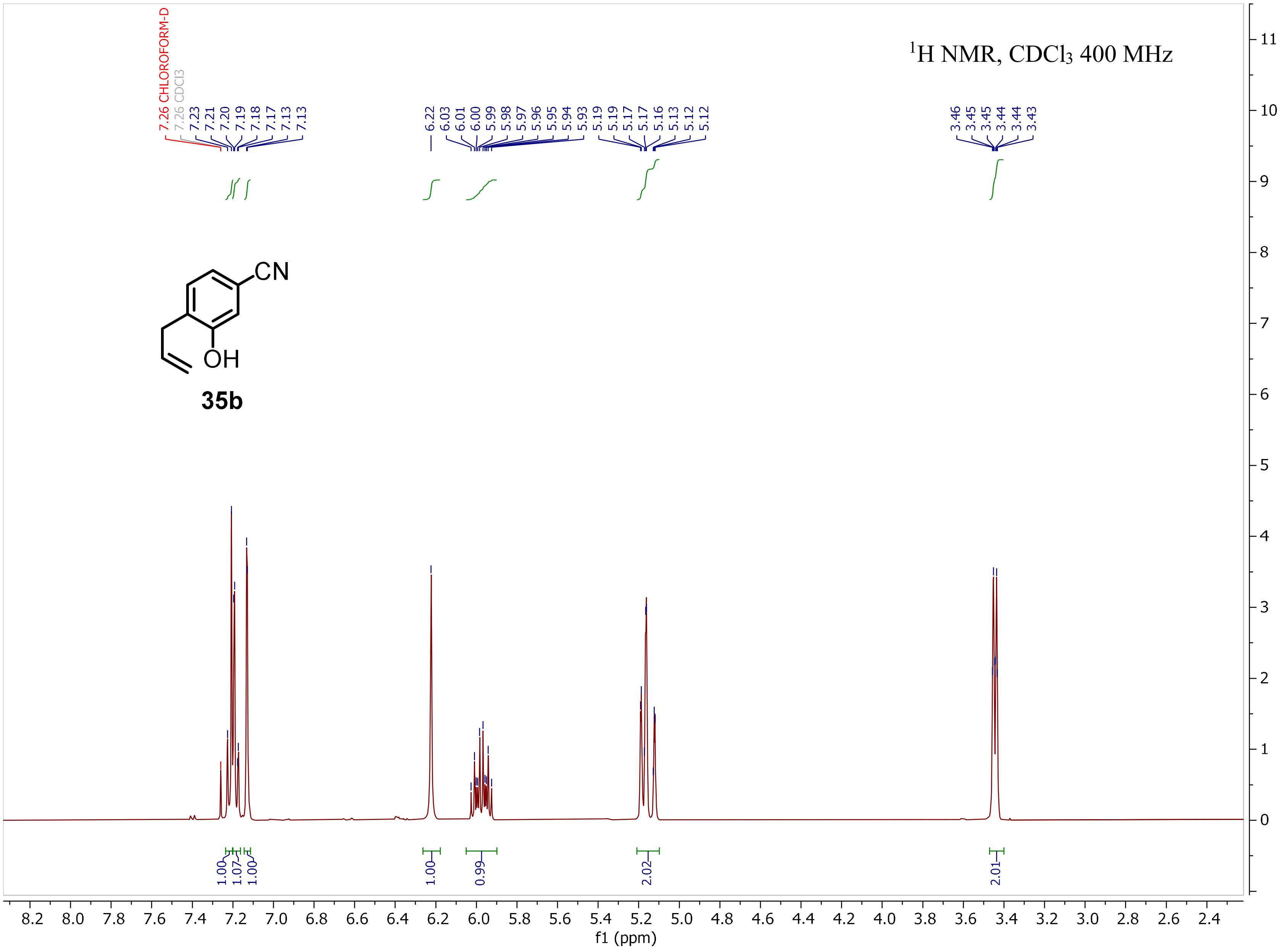

1H NMR, CDCl3 400 MHz
7.26 CHLOROFORM-D
7.26 CDCl3
7.23 7.21 7.20 7.19 7.18 7.17 7.13 7.13
6.22 6.03 6.01 6.00 5.99 5.98 5.97 5.96 5.95 5.94 5.93
5.19 5.19 5.17 5.17 5.16 5.13 5.12 5.12
3.46 3.45 3.45 3.44 3.44 3.43
CN
OH
35b
1.00 1.07 1.00 1.00 0.99 2.02 2.01
f1 (ppm)

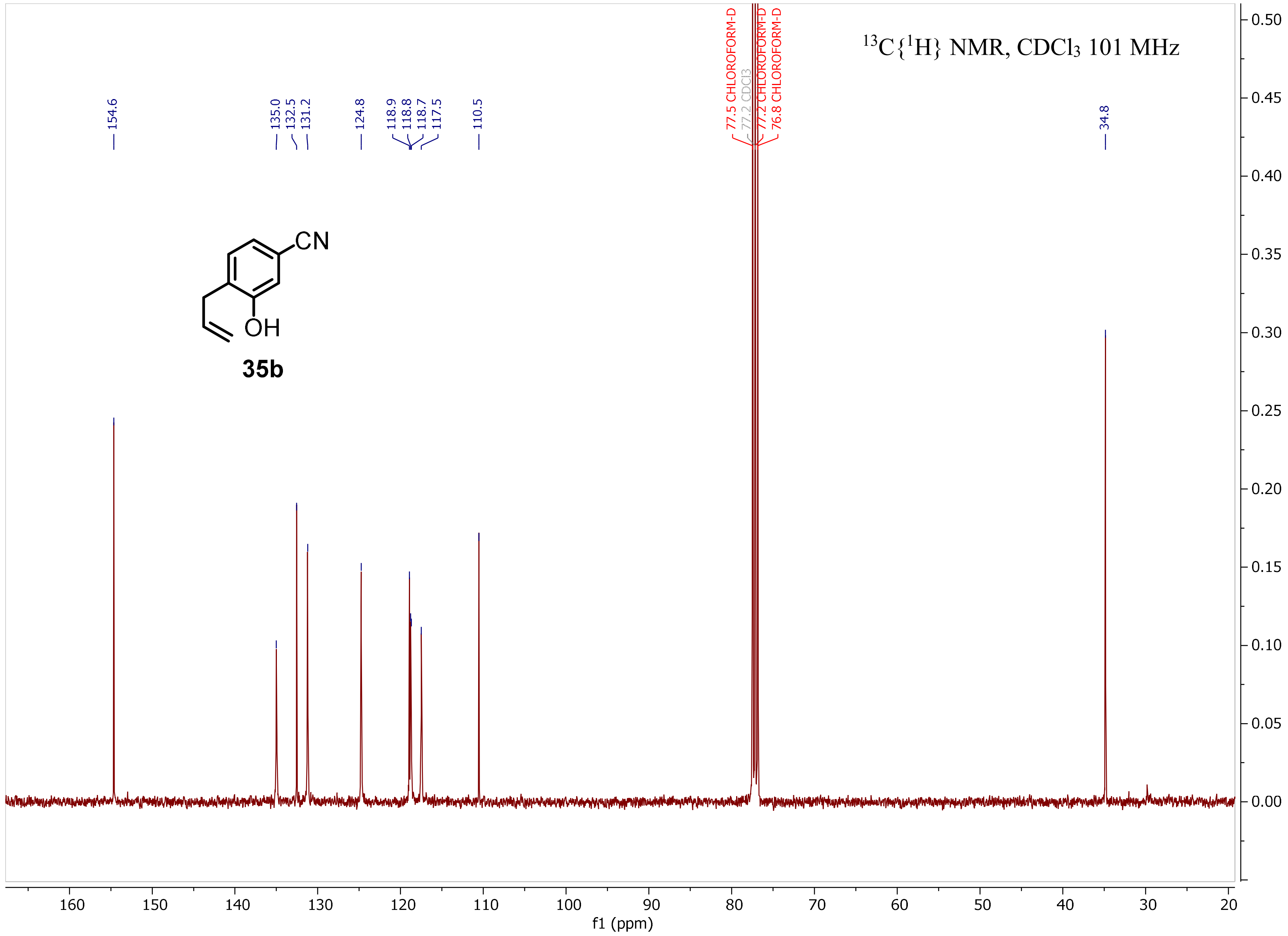

$^{13}C\{^1H\}$ NMR, CDCl₃ 101 MHz
154.6
135.0
132.5
131.2
124.8
118.9
118.8
118.7
117.5
110.5
77.5 CHLOROFORM-D
77.2 CDCl3
77.2 CHLOROFORM-D
76.8 CHLOROFORM-D
34.8
CN
OH
35b
f1 (ppm)

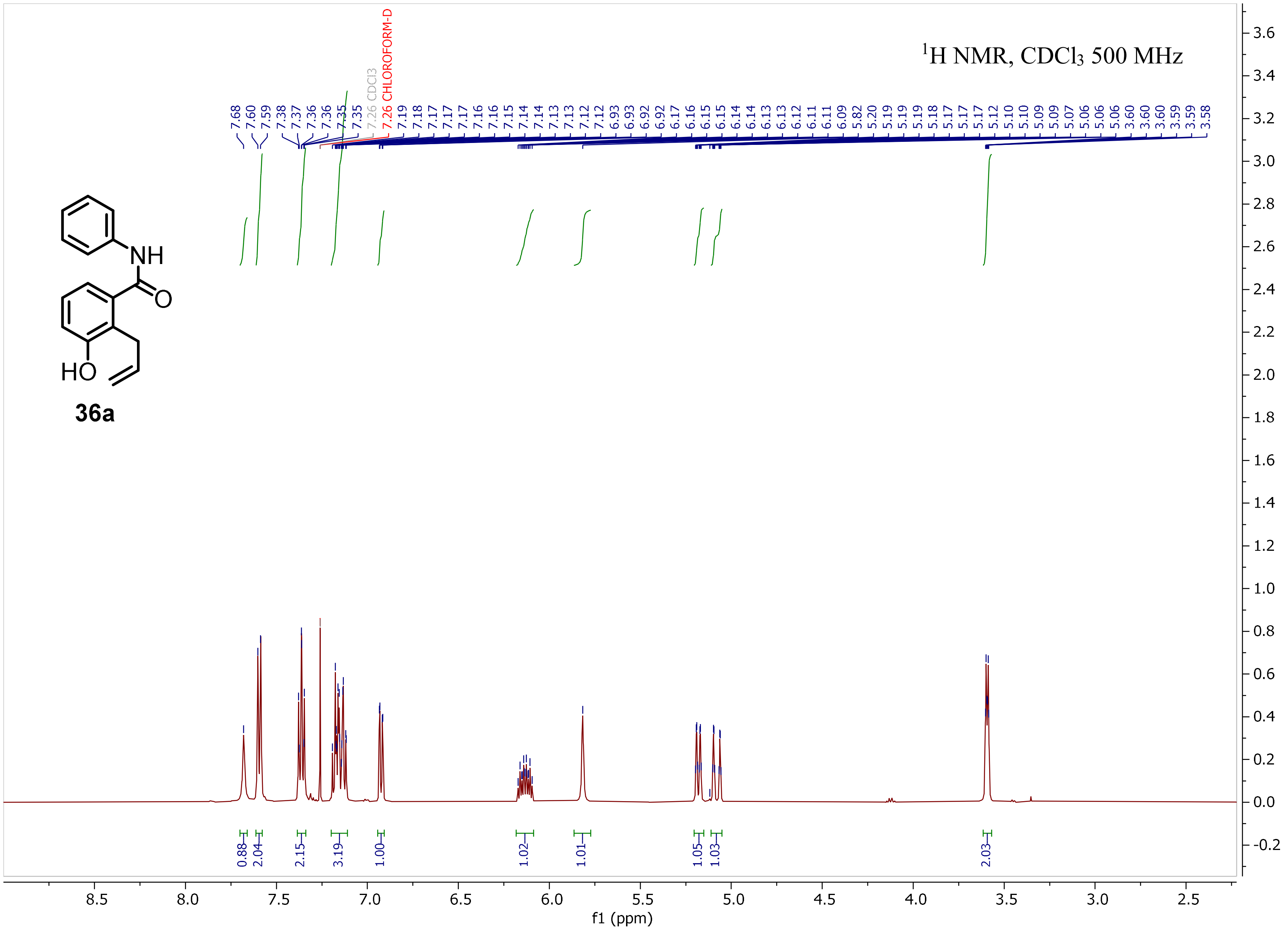

^{1}H NMR, CDCl$_3$ 500 MHz
7.68 7.60 7.59 7.38 7.37 7.36 7.36 7.35 7.35 7.26 CDCl3 7.26 CHLOROFORM-D 7.19 7.18 7.17 7.17 7.17 7.16 7.16 7.15 7.14 7.14 7.13 7.13 7.12 7.12 6.93 6.93 6.92 6.92 6.17 6.16 6.15 6.15 6.14 6.14 6.13 6.13 6.12 6.11 6.11 6.09 5.82 5.20 5.19 5.19 5.19 5.18 5.17 5.17 5.17 5.12 5.10 5.10 5.09 5.09 5.07 5.06 5.06 5.06 3.60 3.60 3.59 3.59 3.58
NH
O
HO
36a
0.88 2.04 2.15 3.19 1.00 1.02 1.01 1.05 1.03 2.03
f1 (ppm)

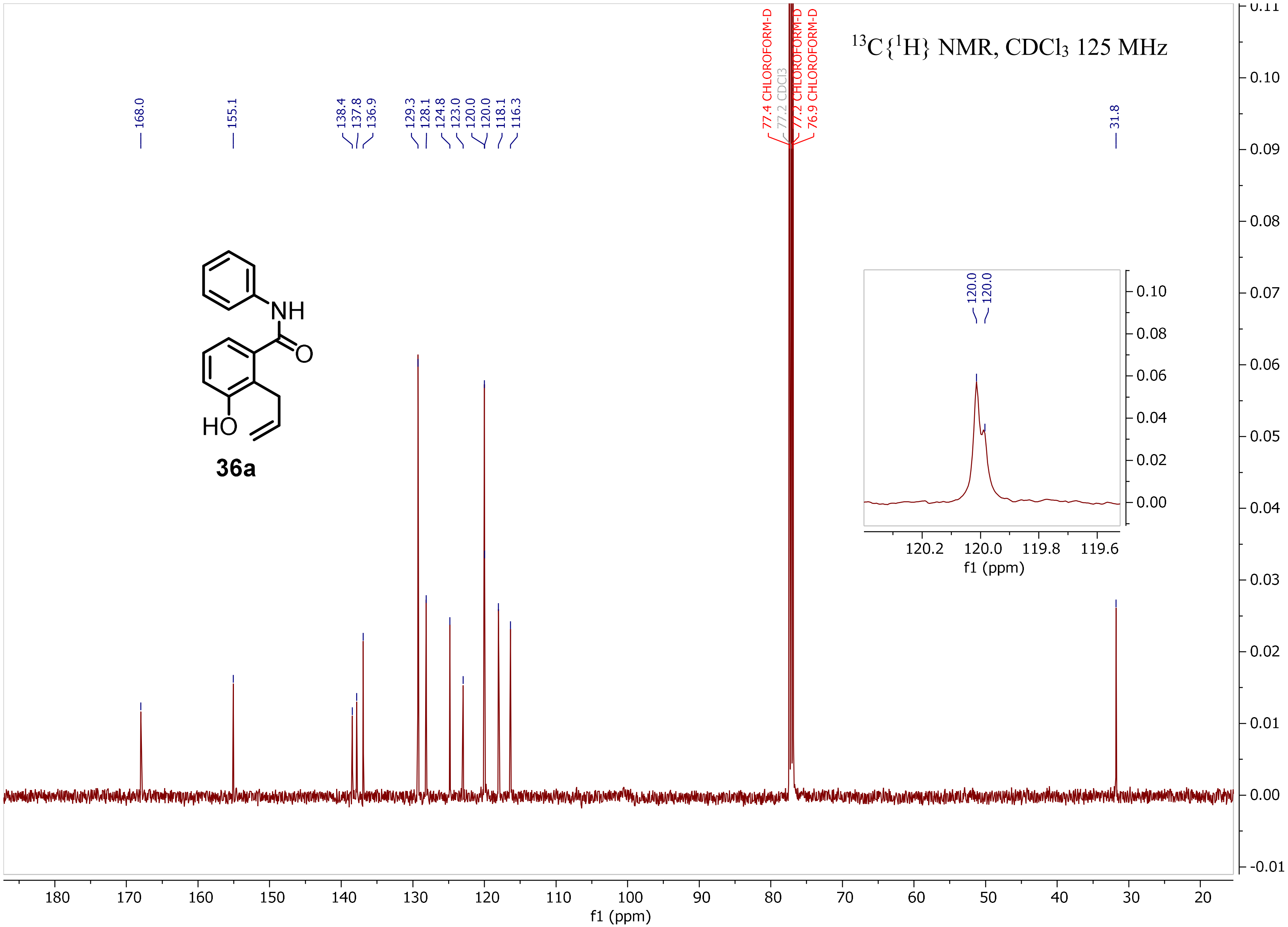

$^{13}C\{^1H\}$ NMR, $CDCl_3$ 125 MHz
168.0
155.1
138.4
137.8
136.9
129.3
128.1
124.8
123.0
120.0
120.0
118.1
116.3
77.4 CHLOROFORM-D
77.2 CDCl3
77.2 CHLOROFORM-D
76.9 CHLOROFORM-D
31.8
NH
O
HO
36a
f1 (ppm)

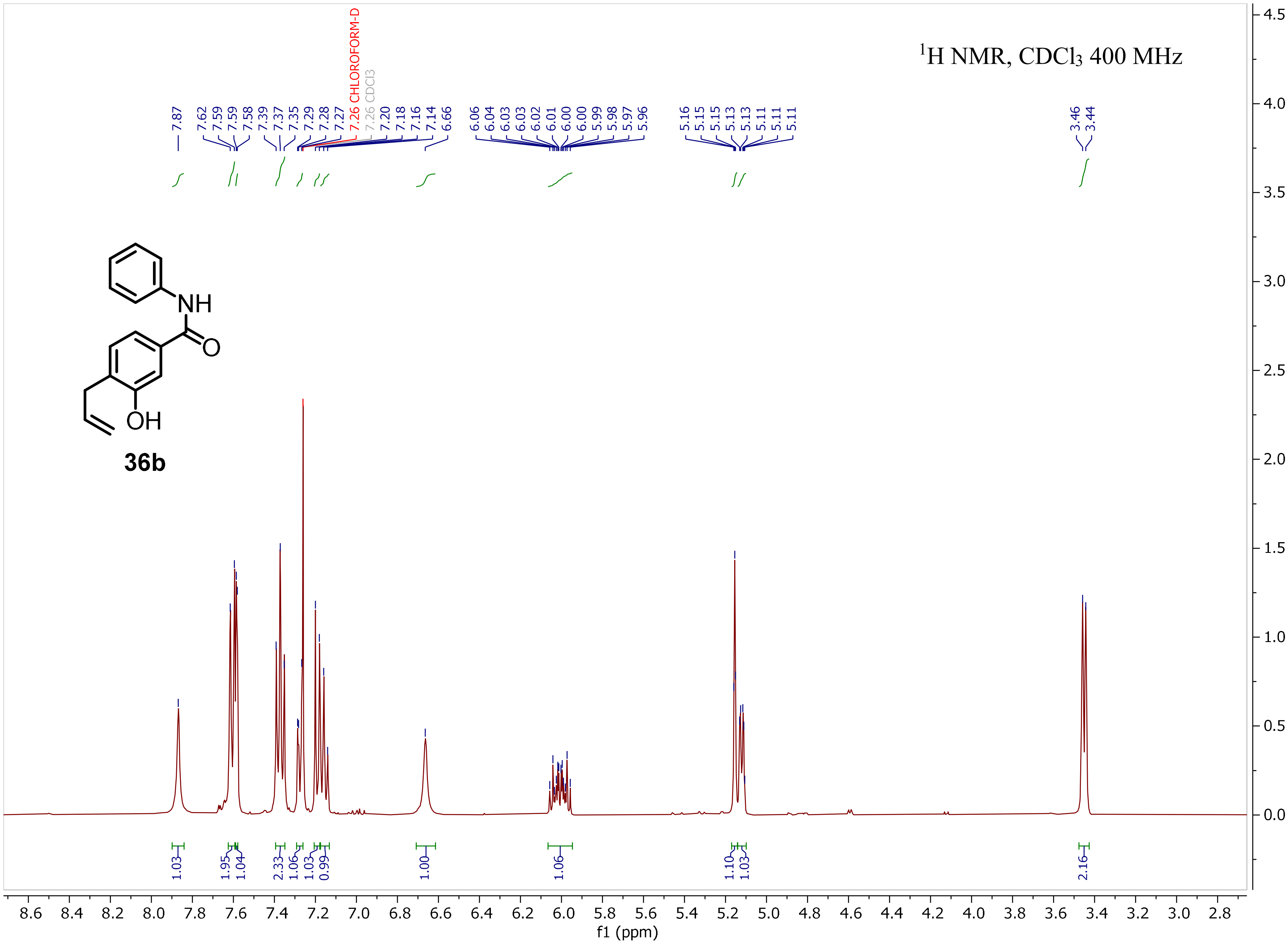

1H NMR, CDCl3 400 MHz
7.87
7.62
7.59
7.59
7.58
7.39
7.37
7.35
7.29
7.28
7.27
7.26 CHLOROFORM-D
7.26 CDCl3
7.20
7.18
7.16
7.14
6.66
6.06
6.04
6.03
6.03
6.02
6.01
6.00
6.00
5.99
5.98
5.97
5.96
5.16
5.15
5.15
5.13
5.13
5.11
5.11
5.11
3.46
3.44
NH
O
OH
36b
1.03
1.95
1.04
2.33
1.06
1.03
0.99
1.00
1.06
1.10
1.03
2.16
f1 (ppm)

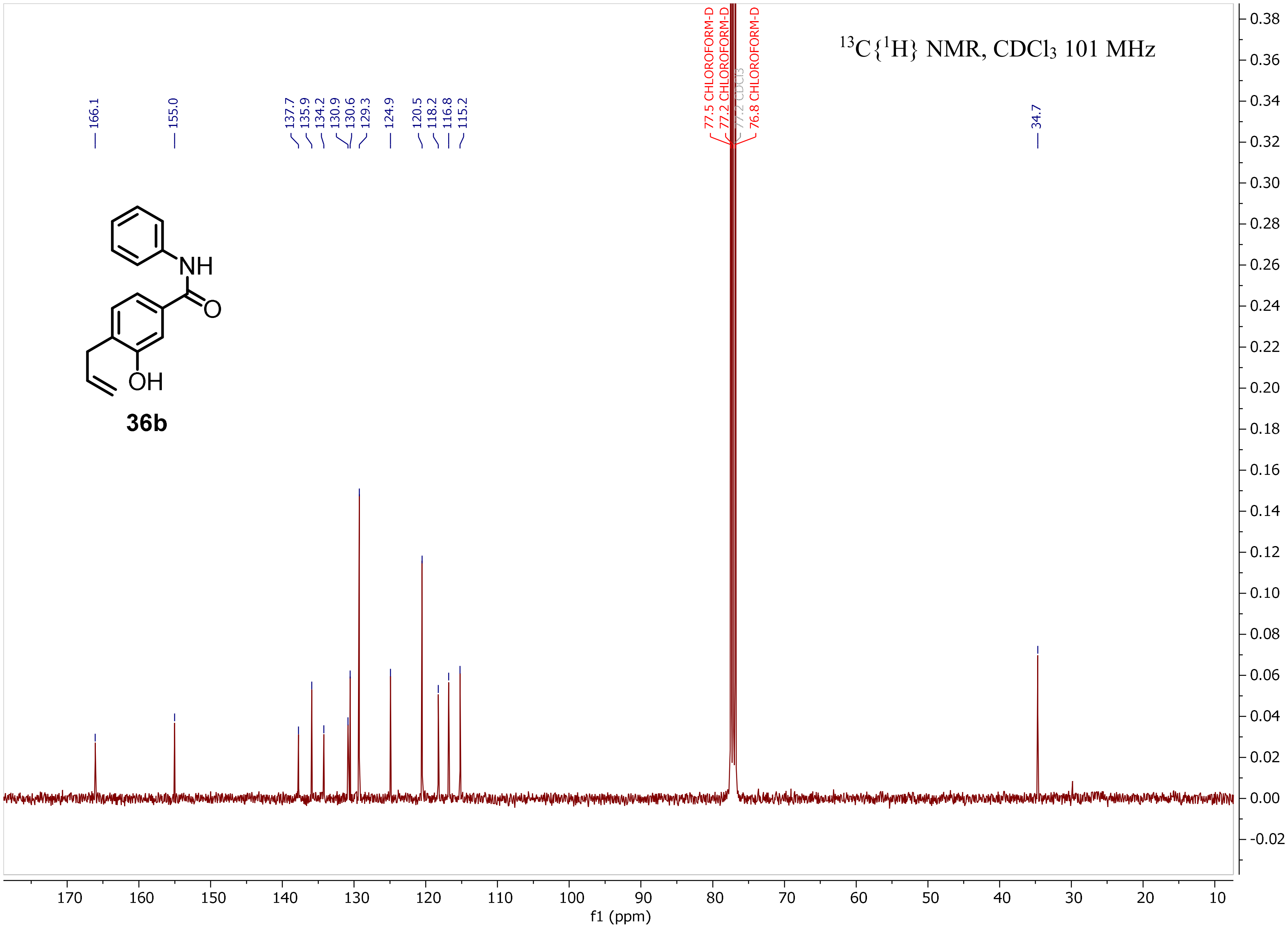

$^{13}C\{^1H\}$ NMR, $CDCl_3$ 101 MHz
166.1
155.0
137.7
135.9
134.2
130.9
130.6
129.3
124.9
120.5
118.2
116.8
115.2
77.5 CHLOROFORM-D
77.2 CHLOROFORM-D
77.2 CDCl3
76.8 CHLOROFORM-D
34.7
NH
O
OH
36b
f1 (ppm)

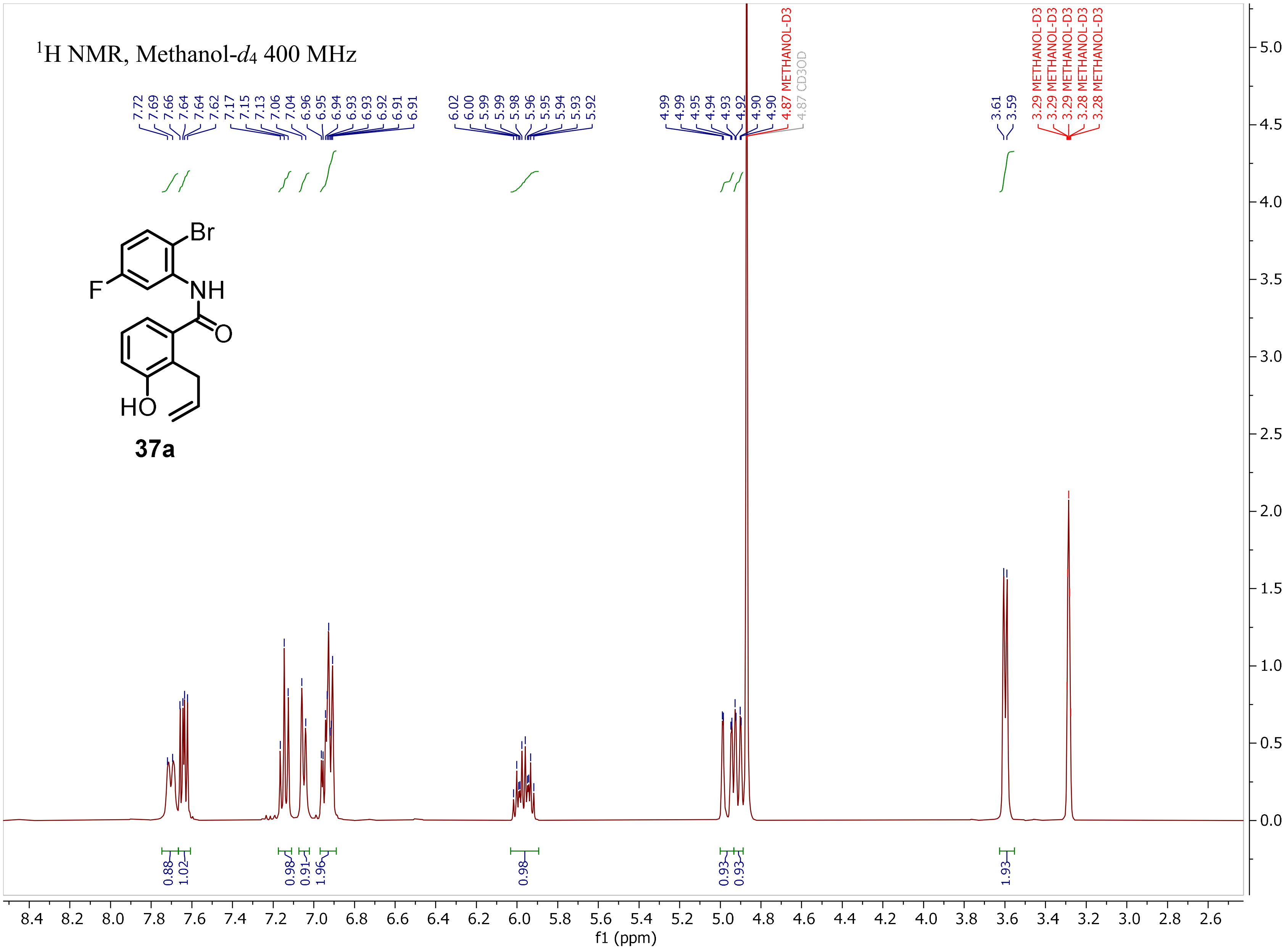

1H NMR, Methanol-d4 400 MHz
Br
F
NH
O
HO
37a
7.72 7.69 7.66 7.64 7.64 7.62 7.17 7.15 7.13 7.06 7.04 6.96 6.95 6.94 6.93 6.93 6.92 6.91 6.91
6.02 6.00 5.99 5.99 5.98 5.96 5.95 5.94 5.93 5.92
4.99 4.99 4.95 4.94 4.93 4.92 4.90 4.90 4.87 METHANOL-D3 4.87 CD3OD
3.61 3.59
3.29 METHANOL-D3 3.29 METHANOL-D3 3.29 METHANOL-D3 3.28 METHANOL-D3 3.28 METHANOL-D3
0.88 1.02 0.98 0.91 1.96 0.98 0.93 0.93 1.93
f1 (ppm)

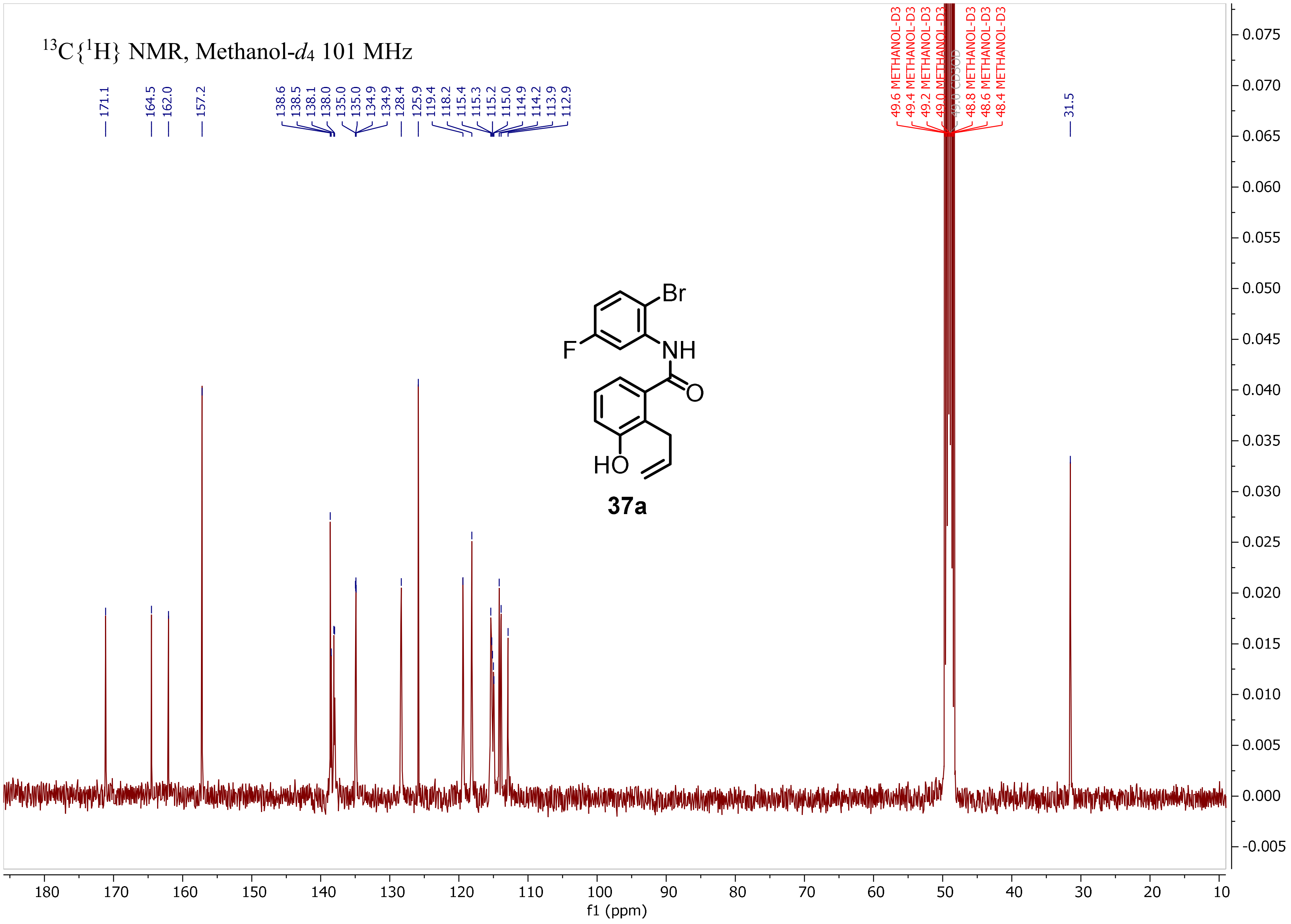

$^{13}C\{^{1}H\}$ NMR, Methanol-d_4 101 MHz
171.1
164.5
162.0
157.2
138.6
138.5
138.1
138.0
135.0
135.0
134.9
134.9
128.4
125.9
119.4
118.2
115.4
115.3
115.2
115.0
114.9
114.2
113.9
112.9
49.6 METHANOL-D3
49.4 METHANOL-D3
49.2 METHANOL-D3
49.0 METHANOL-D3
49.0 CD3OD
48.8 METHANOL-D3
48.6 METHANOL-D3
48.4 METHANOL-D3
31.5
Br
F
NH
O
HO
37a
f1 (ppm)

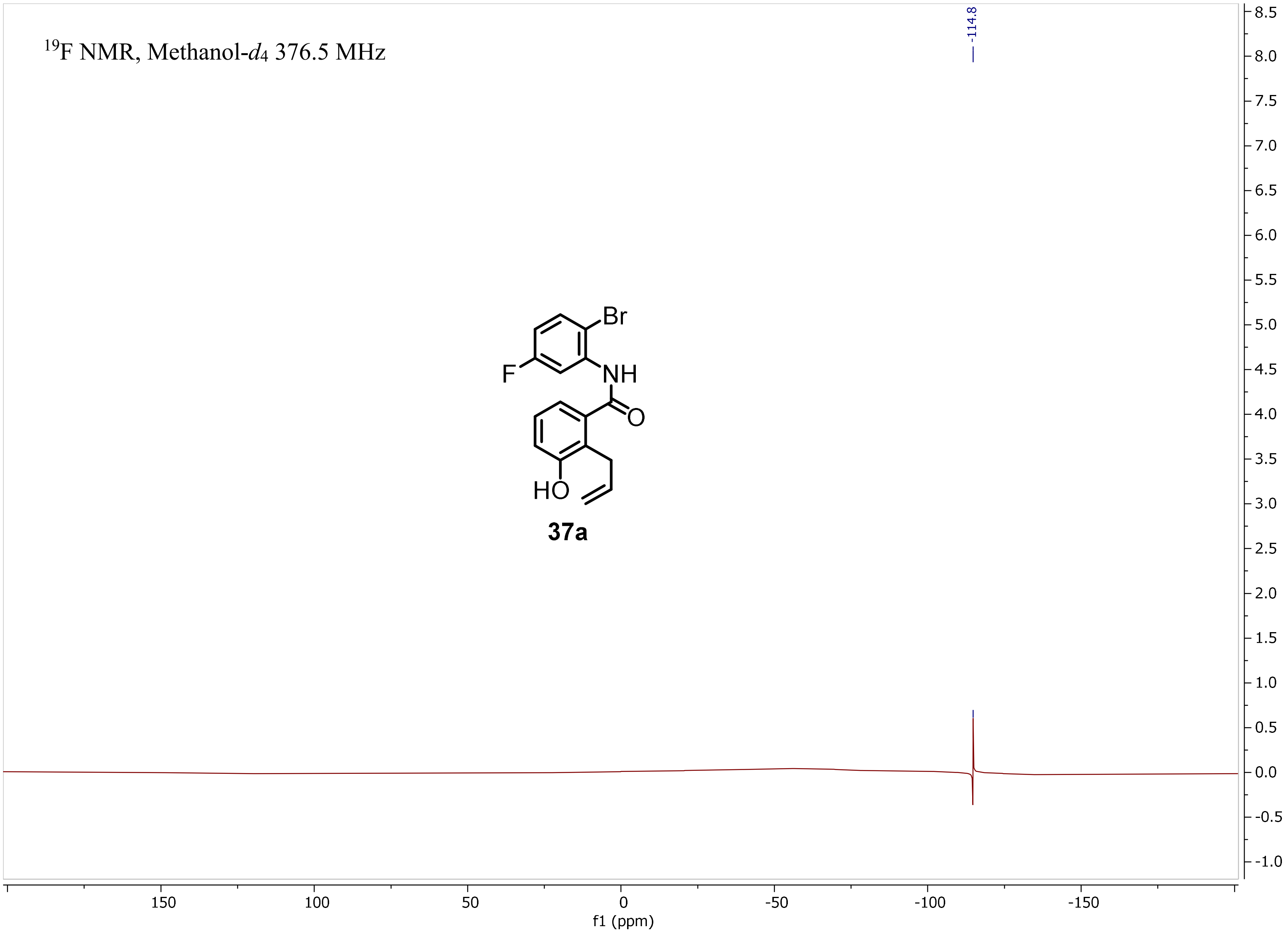

^{19}F NMR, Methanol-d_4 376.5 MHz
-114.8
Br
F
NH
O
HO
37a
f1 (ppm)

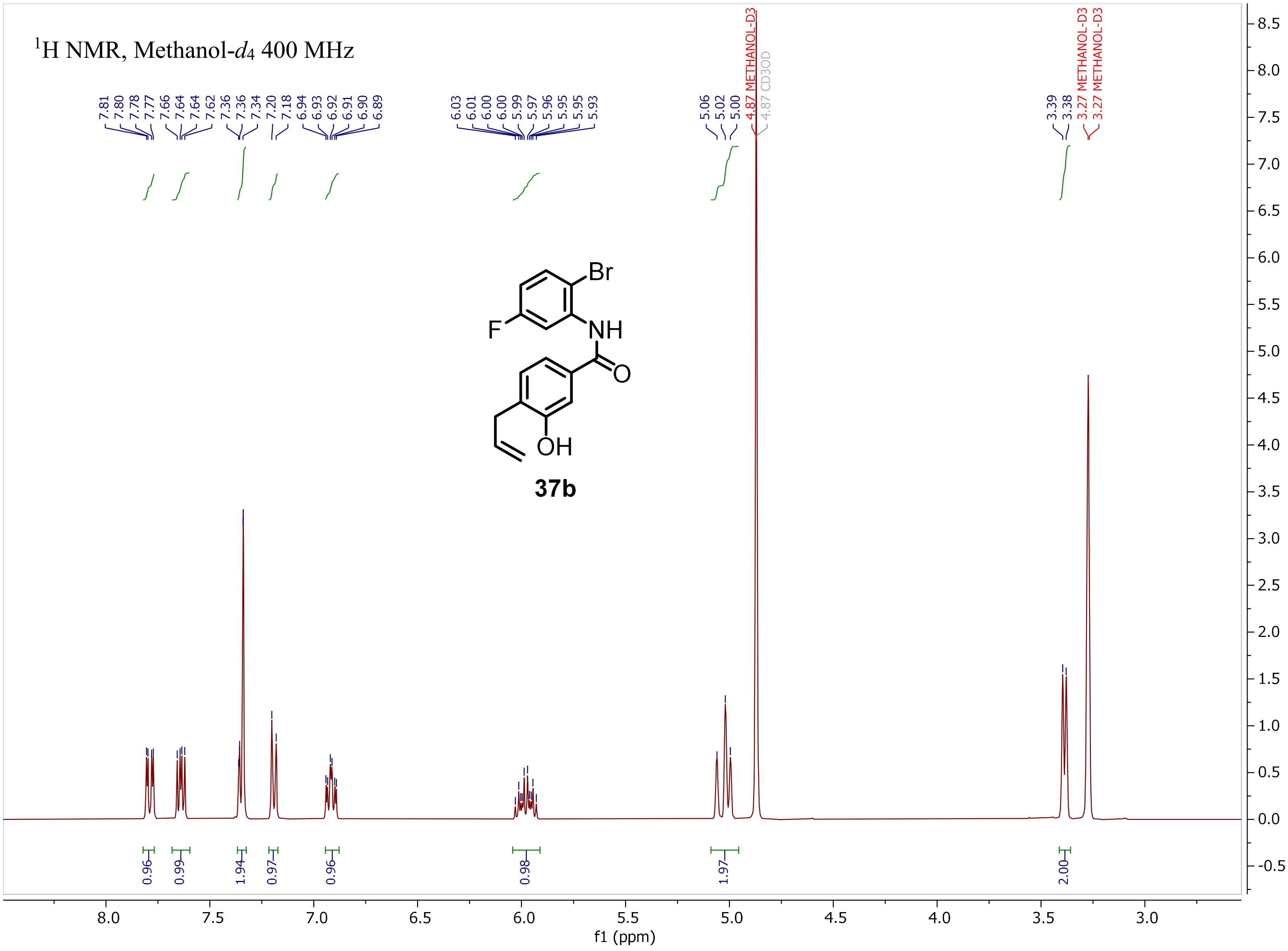

^{1}H NMR, Methanol-d_4 400 MHz
7.81 7.80 7.78 7.77 7.66 7.64 7.64 7.62 7.36 7.36 7.34 7.20 7.18 6.94 6.93 6.92 6.91 6.90 6.89
6.03 6.01 6.00 6.00 5.99 5.97 5.96 5.95 5.95 5.93
5.06 5.02 5.00
4.87 METHANOL-D3
4.87 CD3OD
3.39 3.38
3.27 METHANOL-D3
3.27 METHANOL-D3
Br
F
NH
O
OH
37b
0.96 0.99 1.94 0.97 0.96 0.98 1.97 2.00
f1 (ppm)

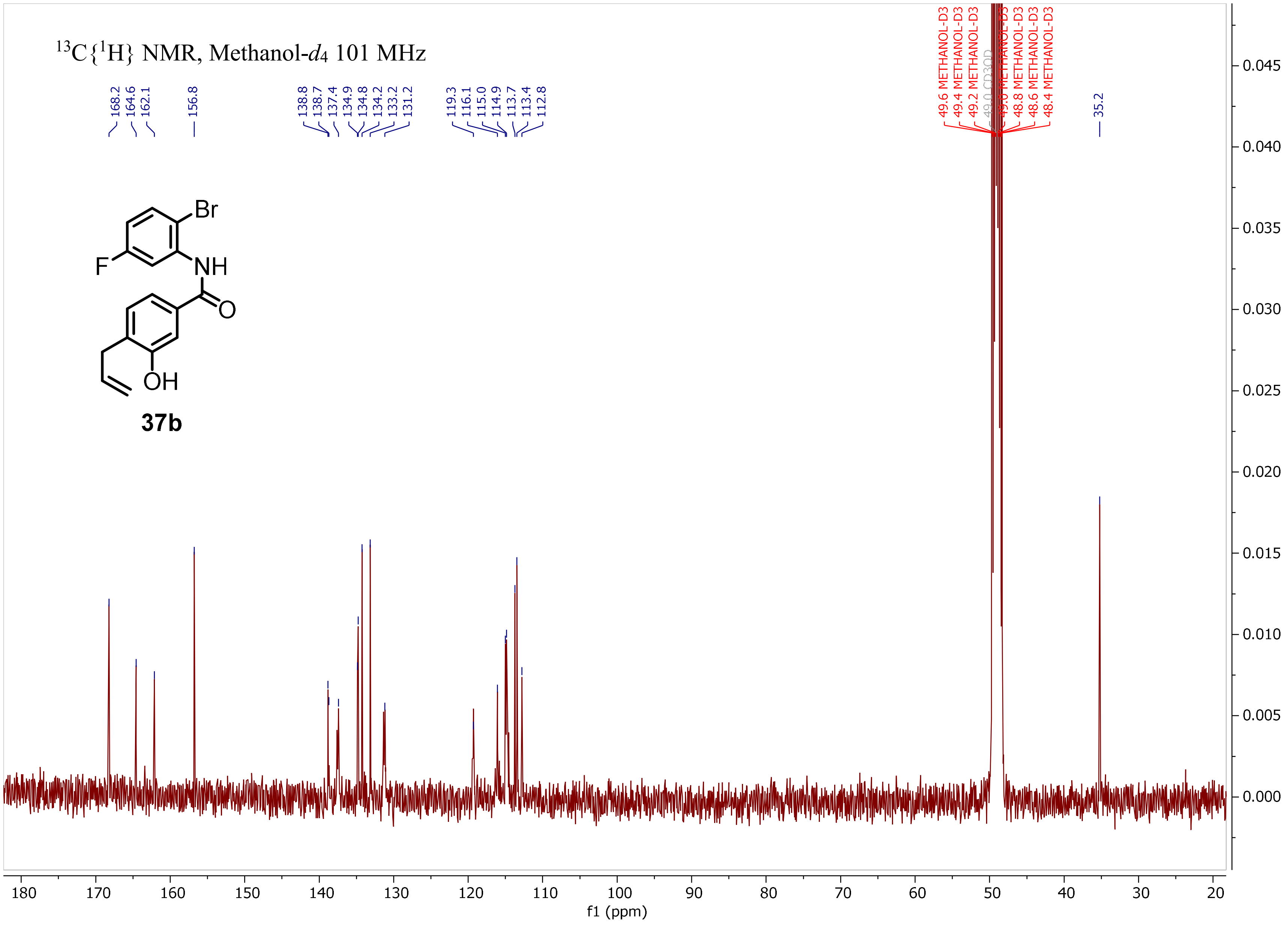

$^{13}C\{^1H\}$ NMR, Methanol-d_4 101 MHz
168.2
164.6
162.1
156.8
138.8
138.7
137.4
134.9
134.8
134.2
133.2
131.2
119.3
116.1
115.0
114.9
113.7
113.4
112.8
49.6 METHANOL-D3
49.4 METHANOL-D3
49.2 METHANOL-D3
48.8 METHANOL-D3
48.6 METHANOL-D3
48.4 METHANOL-D3
35.2
Br
F
NH
O
OH
37b
f1 (ppm)

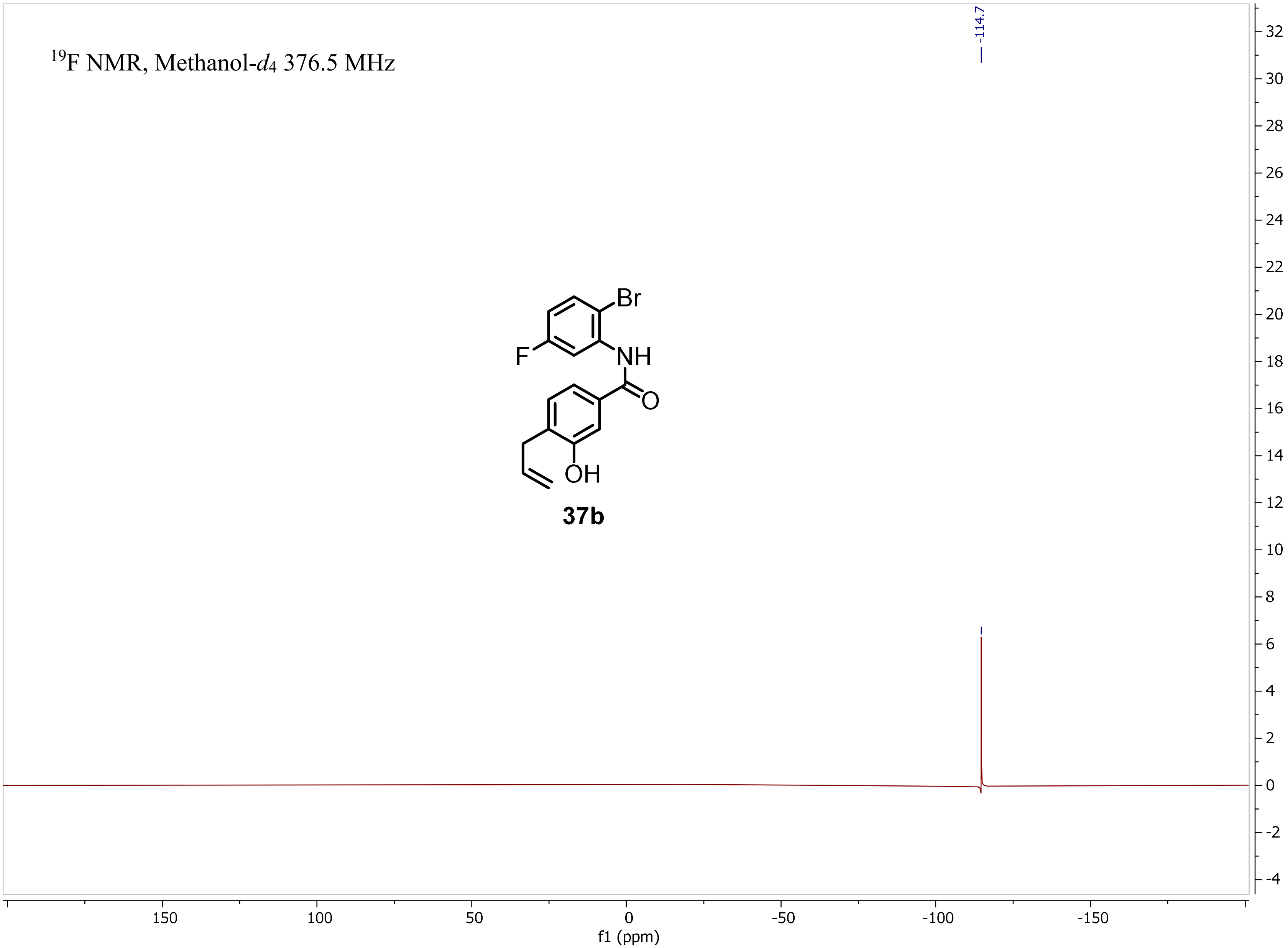

19F NMR, Methanol-d4 376.5 MHz
-114.7
Br
NH
F
O
OH
37b
f1 (ppm)

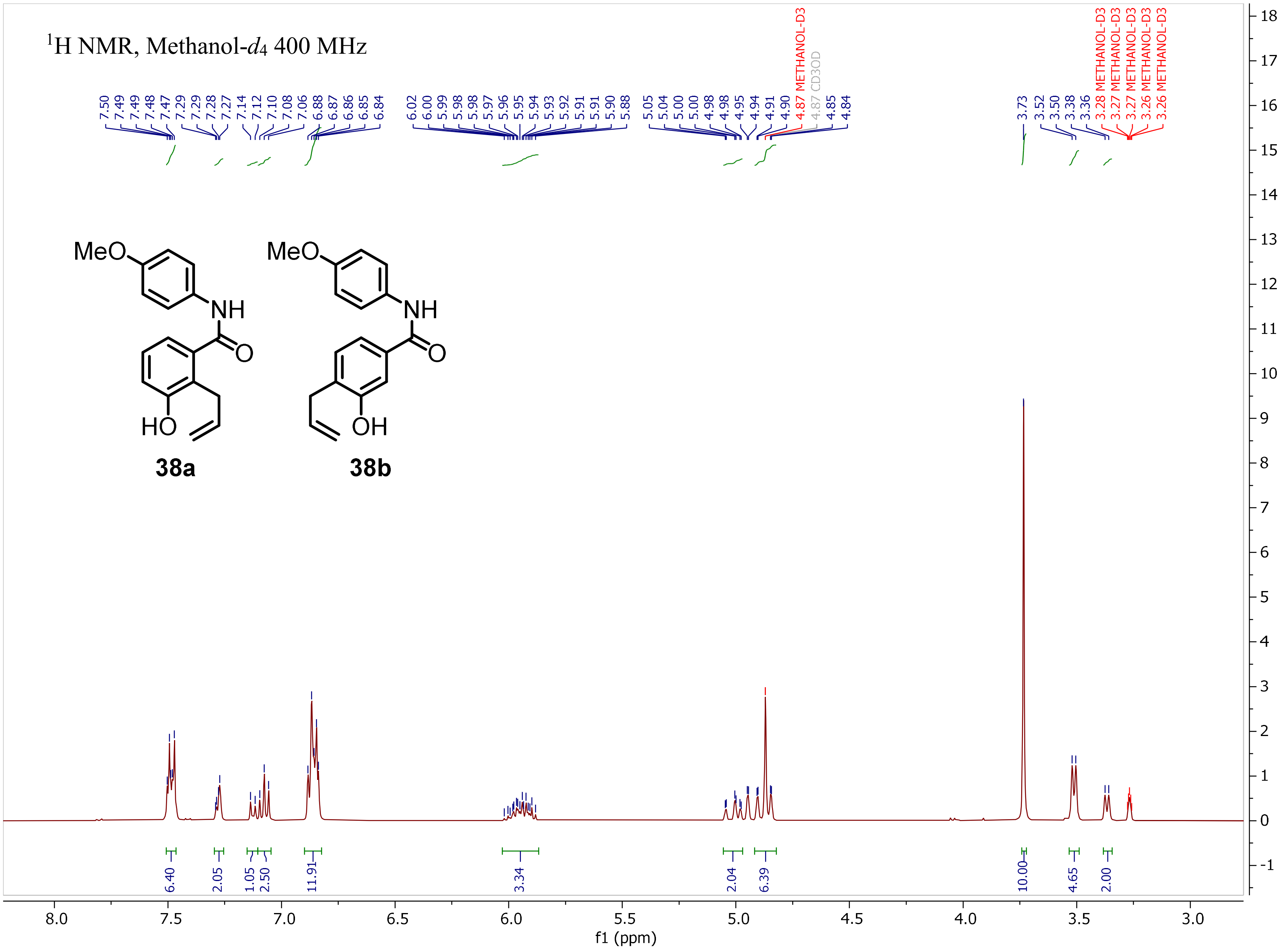

^{1}H NMR, Methanol-d_4 400 MHz
MeO
NH
O
HO
38a
MeO
NH
O
OH
38b
f1 (ppm)

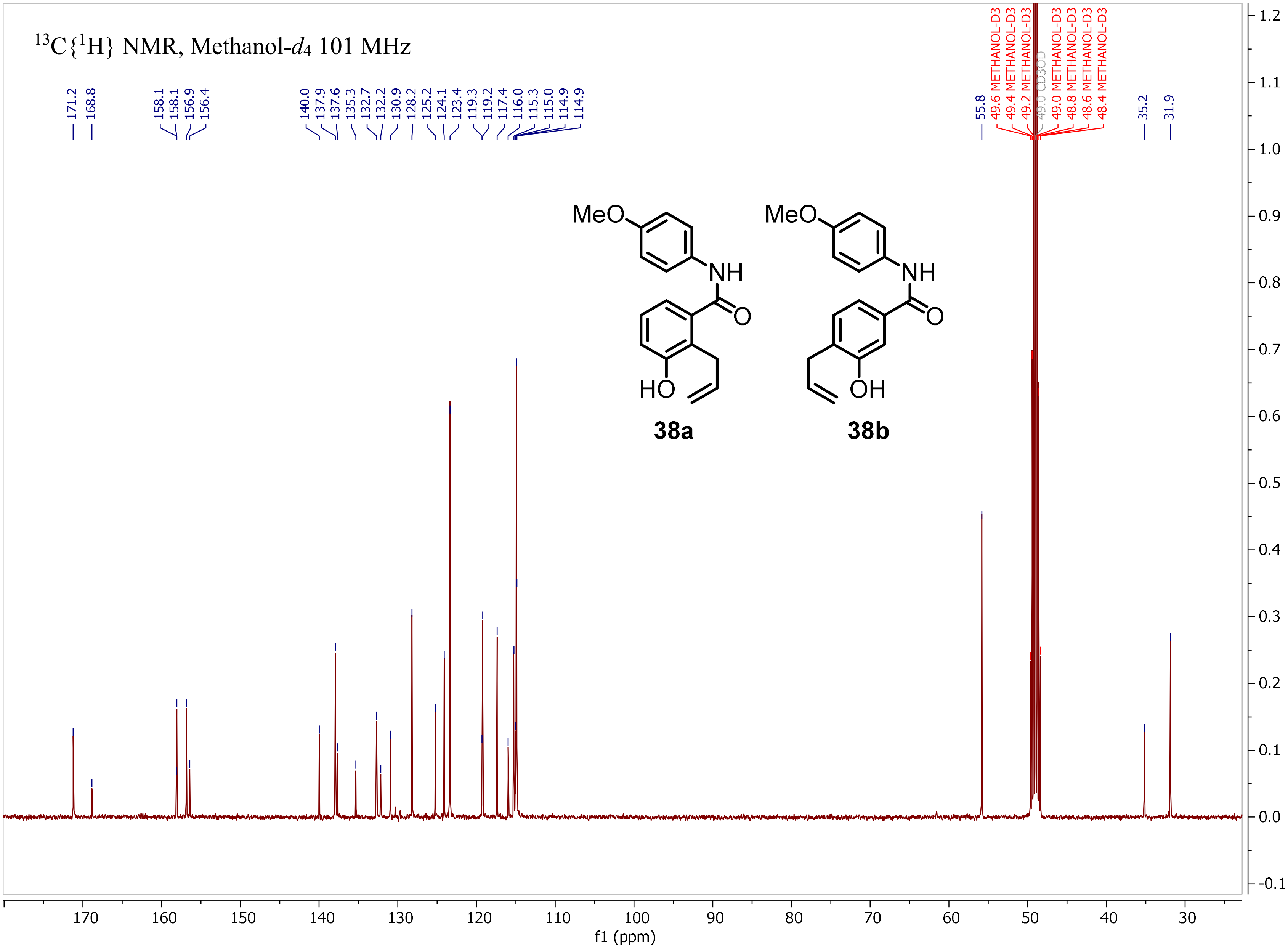

$^{13}C\{^1H\}$ NMR, Methanol-d_4 101 MHz
171.2
168.8
158.1
158.1
156.9
156.4
140.0
137.9
137.6
135.3
132.7
132.2
130.9
128.2
125.2
124.1
123.4
119.3
119.2
117.4
116.0
115.3
115.0
114.9
114.9
55.8
49.6 METHANOL-D3
49.4 METHANOL-D3
49.2 METHANOL-D3
49.0 CD3OD
49.0 METHANOL-D3
48.8 METHANOL-D3
48.6 METHANOL-D3
48.4 METHANOL-D3
35.2
31.9
MeO
NH
O
HO
38a
MeO
NH
O
OH
38b
f1 (ppm)

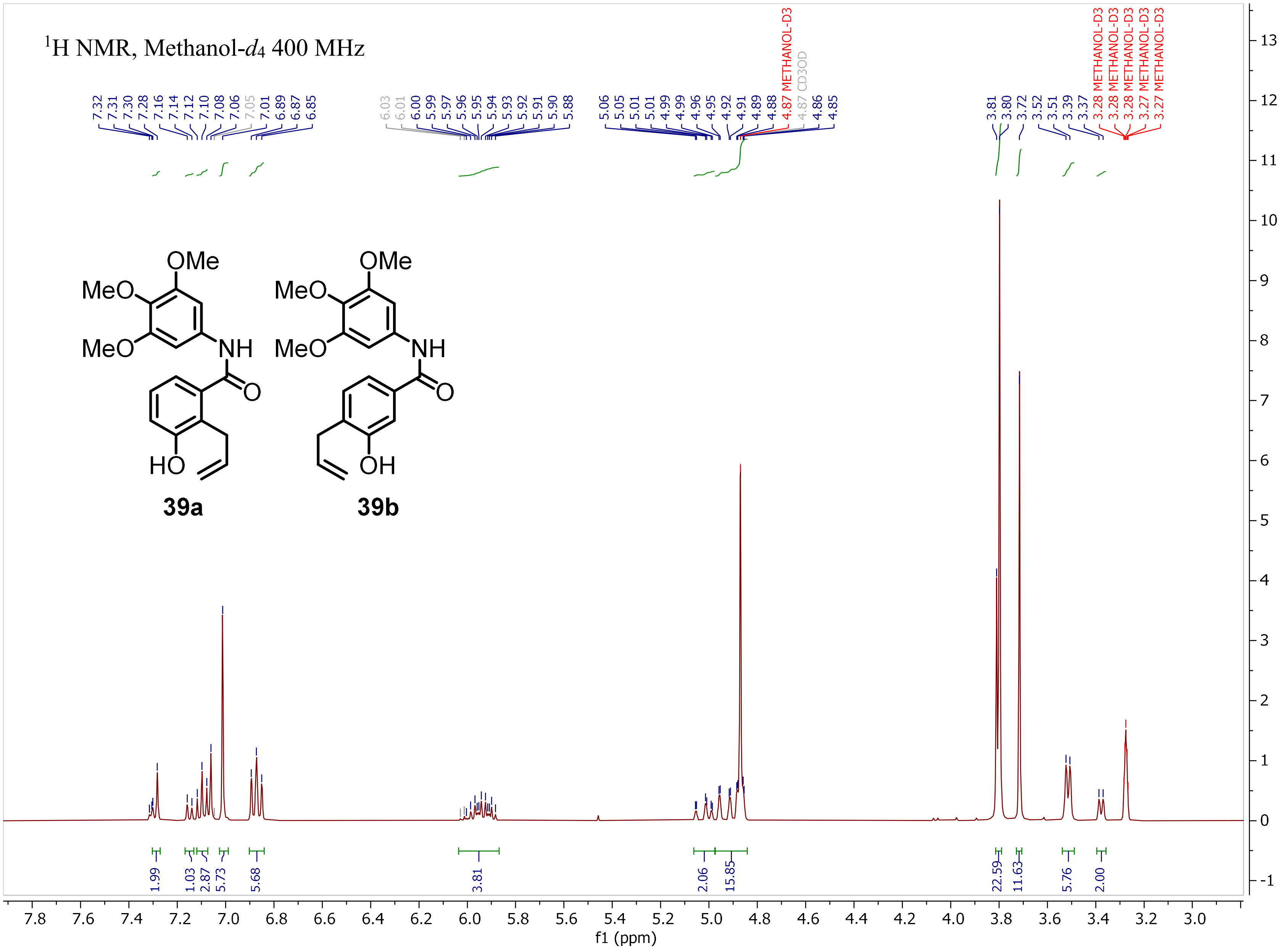

^{1}H NMR, Methanol-d_4 400 MHz
39a
39b
f1 (ppm)

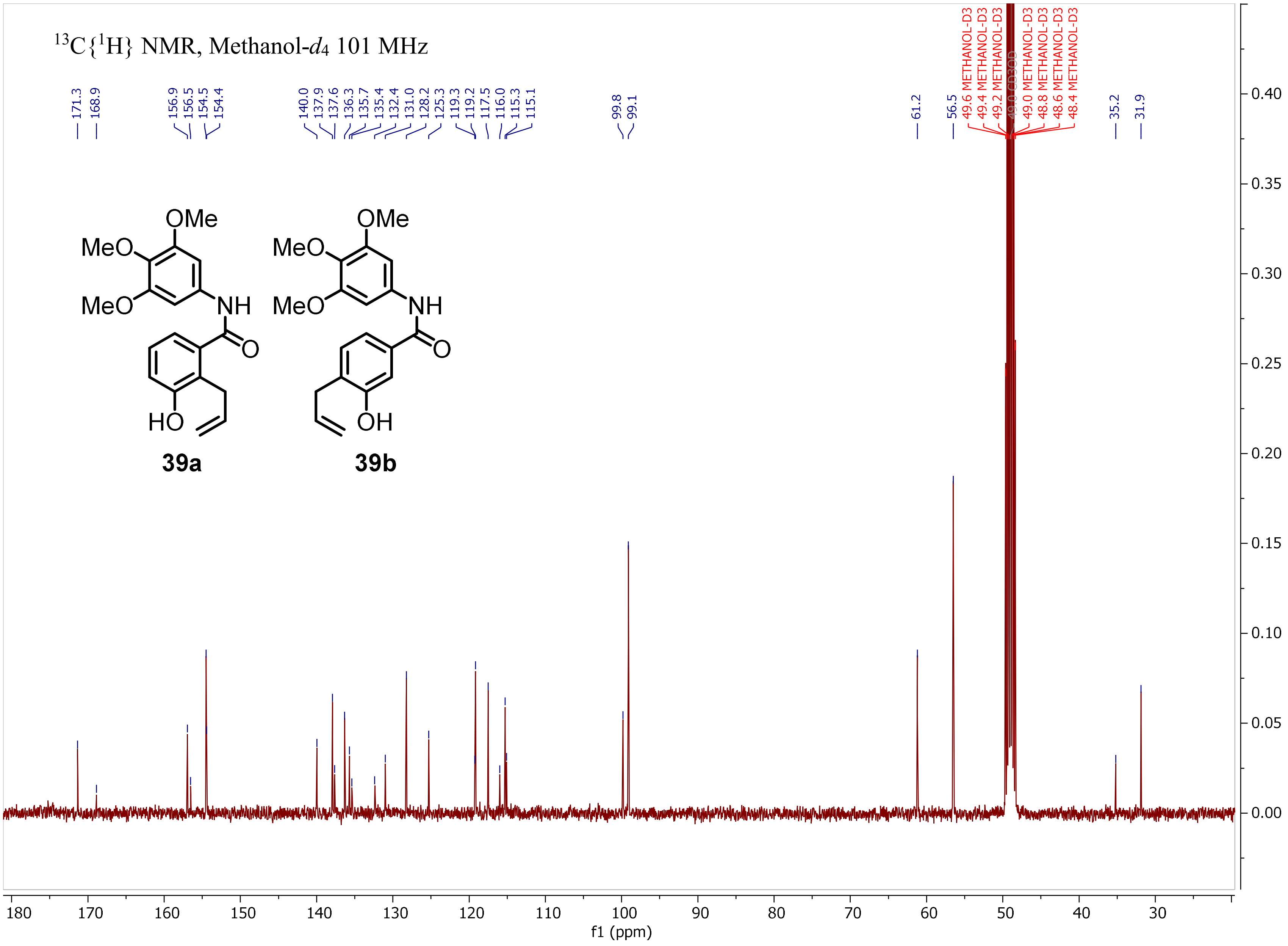
13C{1H} NMR, Methanol-d4 101 MHz
171.3
168.9
156.9
156.5
154.5
154.4
140.0
137.9
137.6
136.3
135.7
135.4
132.4
131.0
128.2
125.3
119.3
119.2
117.5
116.0
115.3
115.1
99.8
99.1
61.2
56.5
49.6 METHANOL-D3
49.4 METHANOL-D3
49.2 METHANOL-D3
49.0 CD3OD
49.0 METHANOL-D3
48.8 METHANOL-D3
48.6 METHANOL-D3
48.4 METHANOL-D3
35.2
31.9
OMe
MeO
MeO
NH
O
HO
39a
OMe
MeO
MeO
NH
O
OH
39b
f1 (ppm)

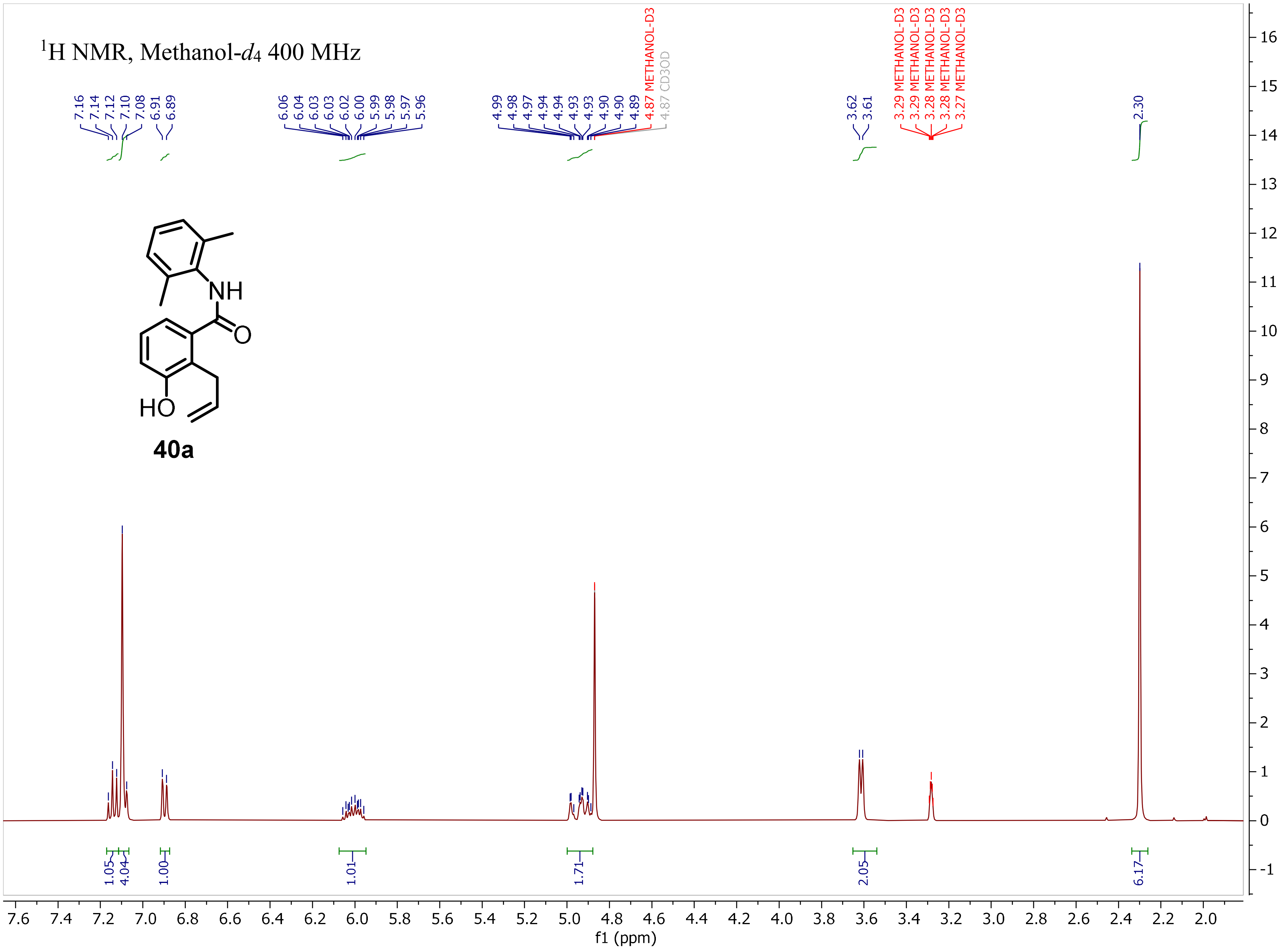

^{1}H NMR, Methanol-d_4 400 MHz
7.16
7.14
7.12
7.10
7.08
6.91
6.89
6.06
6.04
6.03
6.03
6.02
6.00
5.99
5.98
5.97
5.96
4.99
4.98
4.97
4.94
4.94
4.93
4.93
4.90
4.90
4.89
4.87 METHANOL-D3
4.87 CD3OD
3.62
3.61
3.29 METHANOL-D3
3.29 METHANOL-D3
3.28 METHANOL-D3
3.28 METHANOL-D3
3.27 METHANOL-D3
2.30
NH
O
HO
40a
1.05
4.04
1.00
1.01
1.71
2.05
6.17
f1 (ppm)

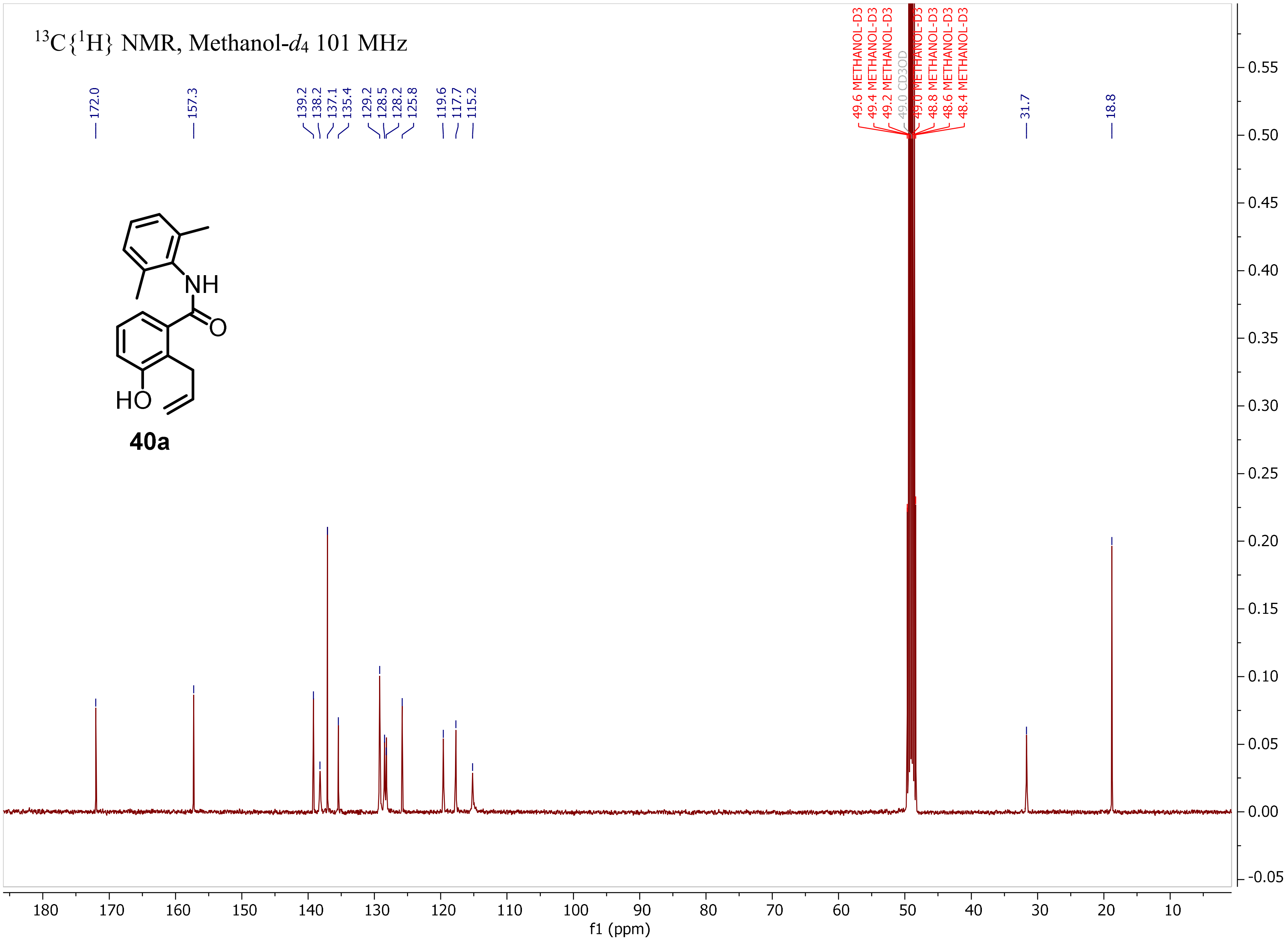
13C{1H} NMR, Methanol-d4 101 MHz
172.0
157.3
139.2
138.2
137.1
135.4
129.2
128.5
128.2
125.8
119.6
117.7
115.2
49.6 METHANOL-D3
49.4 METHANOL-D3
49.2 METHANOL-D3
49.0 CD3OD
49.0 METHANOL-D3
48.8 METHANOL-D3
48.6 METHANOL-D3
48.4 METHANOL-D3
31.7
18.8
NH
O
HO
40a
f1 (ppm)

^{1}H NMR, Methanol-d_4 400 MHz

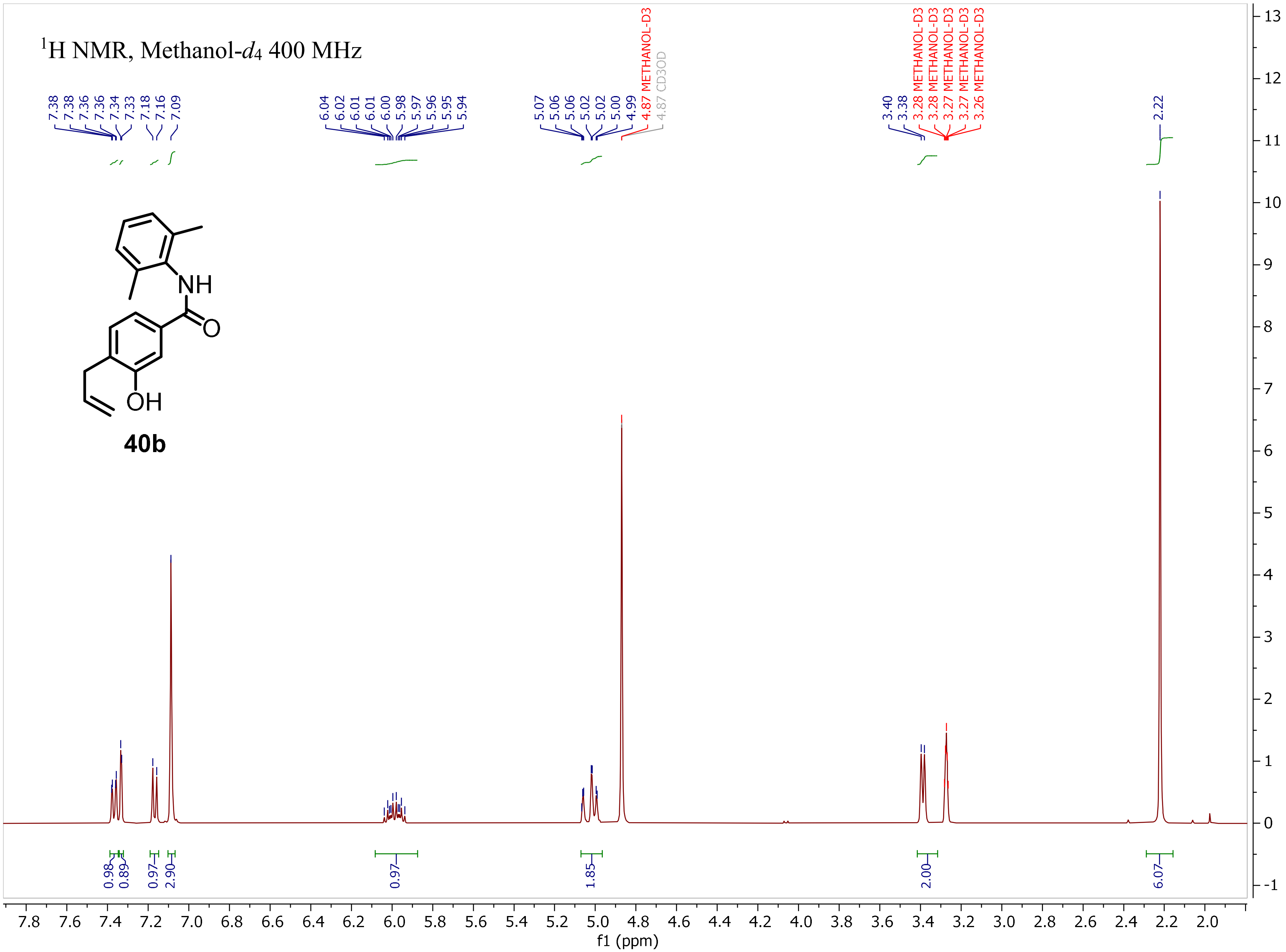

40b

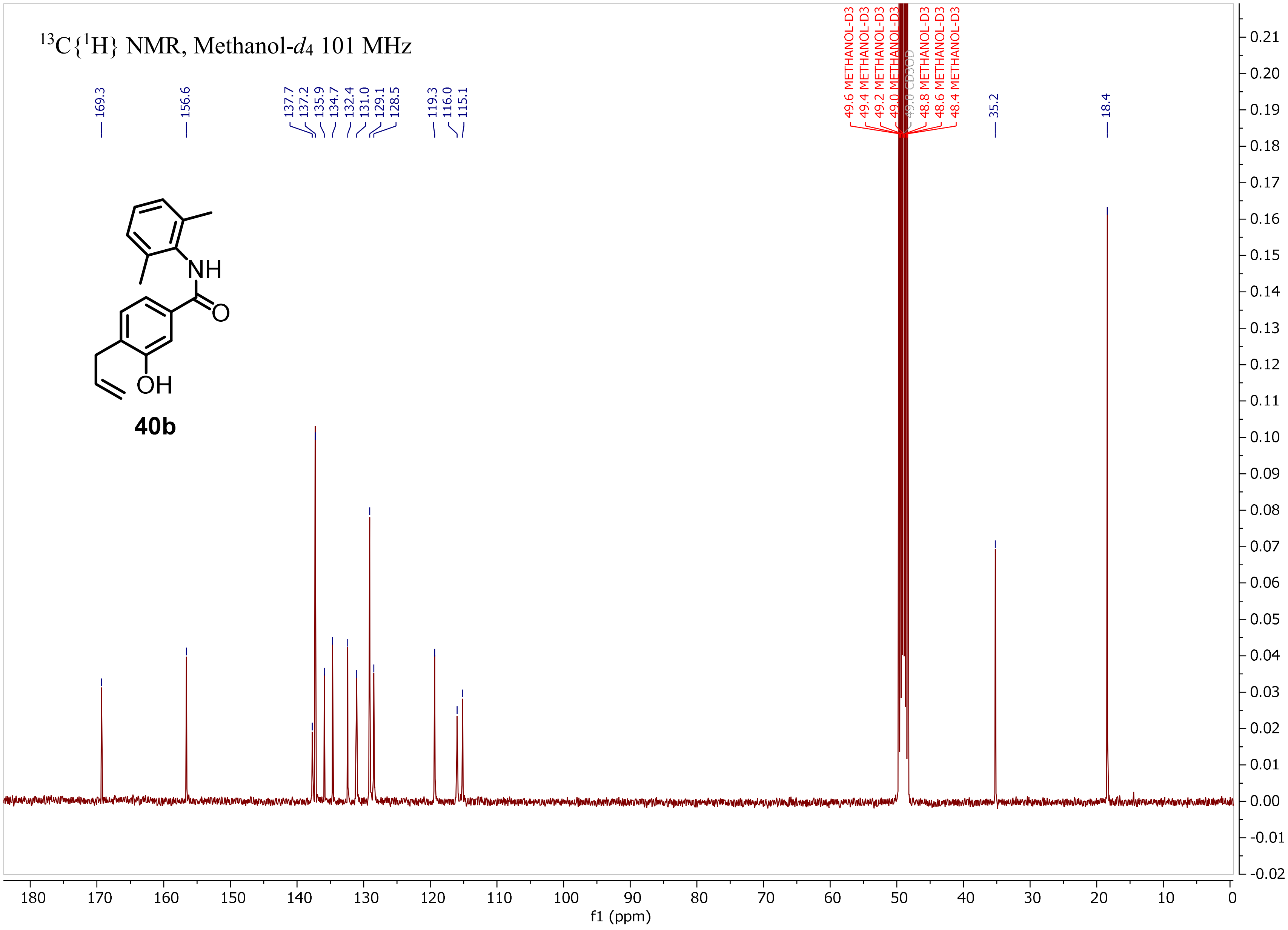

13C{1H} NMR, Methanol-d4 101 MHz
169.3
156.6
137.7
137.2
135.9
134.7
132.4
131.0
129.1
128.5
119.3
116.0
115.1
49.6 METHANOL-D3
49.4 METHANOL-D3
49.2 METHANOL-D3
49.0 METHANOL-D3
48.8 METHANOL-D3
48.6 METHANOL-D3
48.4 METHANOL-D3
35.2
18.4
NH
O
OH
40b
f1 (ppm)

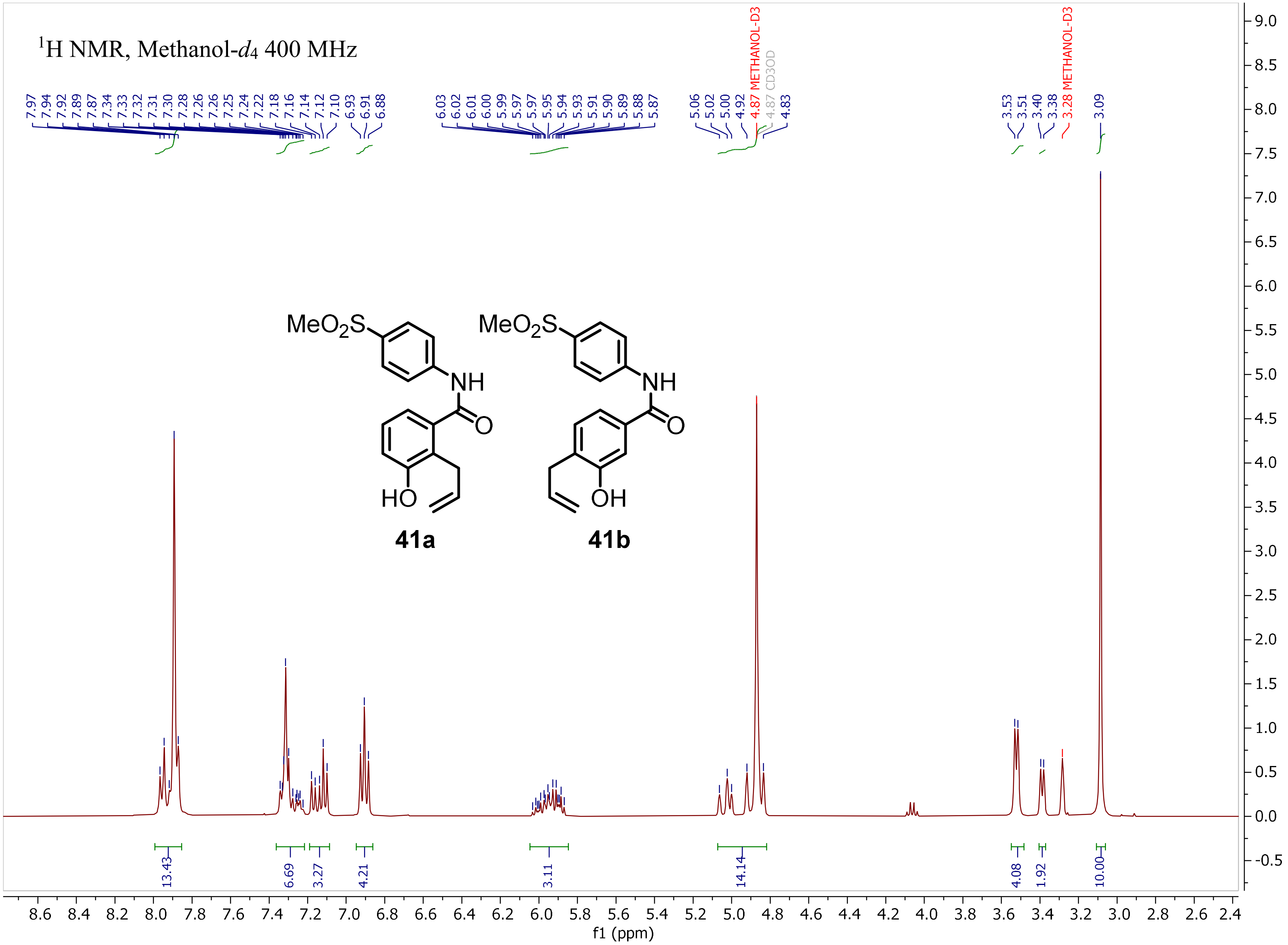

^{1}H NMR, Methanol-d_4 400 MHz
7.97 7.94 7.92 7.89 7.87 7.34 7.33 7.32 7.31 7.30 7.28 7.26 7.26 7.25 7.24 7.22 7.18 7.16 7.14 7.12 7.10 6.93 6.91 6.88
6.03 6.02 6.01 6.00 5.99 5.97 5.95 5.94 5.93 5.91 5.90 5.89 5.88 5.87
5.06 5.02 5.00 4.92 4.87 METHANOL-D3 4.87 CD3OD 4.83
3.53 3.51 3.40 3.38 3.28 METHANOL-D3 3.09
MeO2S
NH
O
HO
OH
41a
41b
13.43 6.69 3.27 4.21 3.11 14.14 4.08 1.92 10.00
8.6 8.4 8.2 8.0 7.8 7.6 7.4 7.2 7.0 6.8 6.6 6.4 6.2 6.0 5.8 5.6 5.4 5.2 5.0 4.8 4.6 4.4 4.2 4.0 3.8 3.6 3.4 3.2 3.0 2.8 2.6 2.4
f1 (ppm)
9.0 8.5 8.0 7.5 7.0 6.5 6.0 5.5 5.0 4.5 4.0 3.5 3.0 2.5 2.0 1.5 1.0 0.5 0.0 -0.5

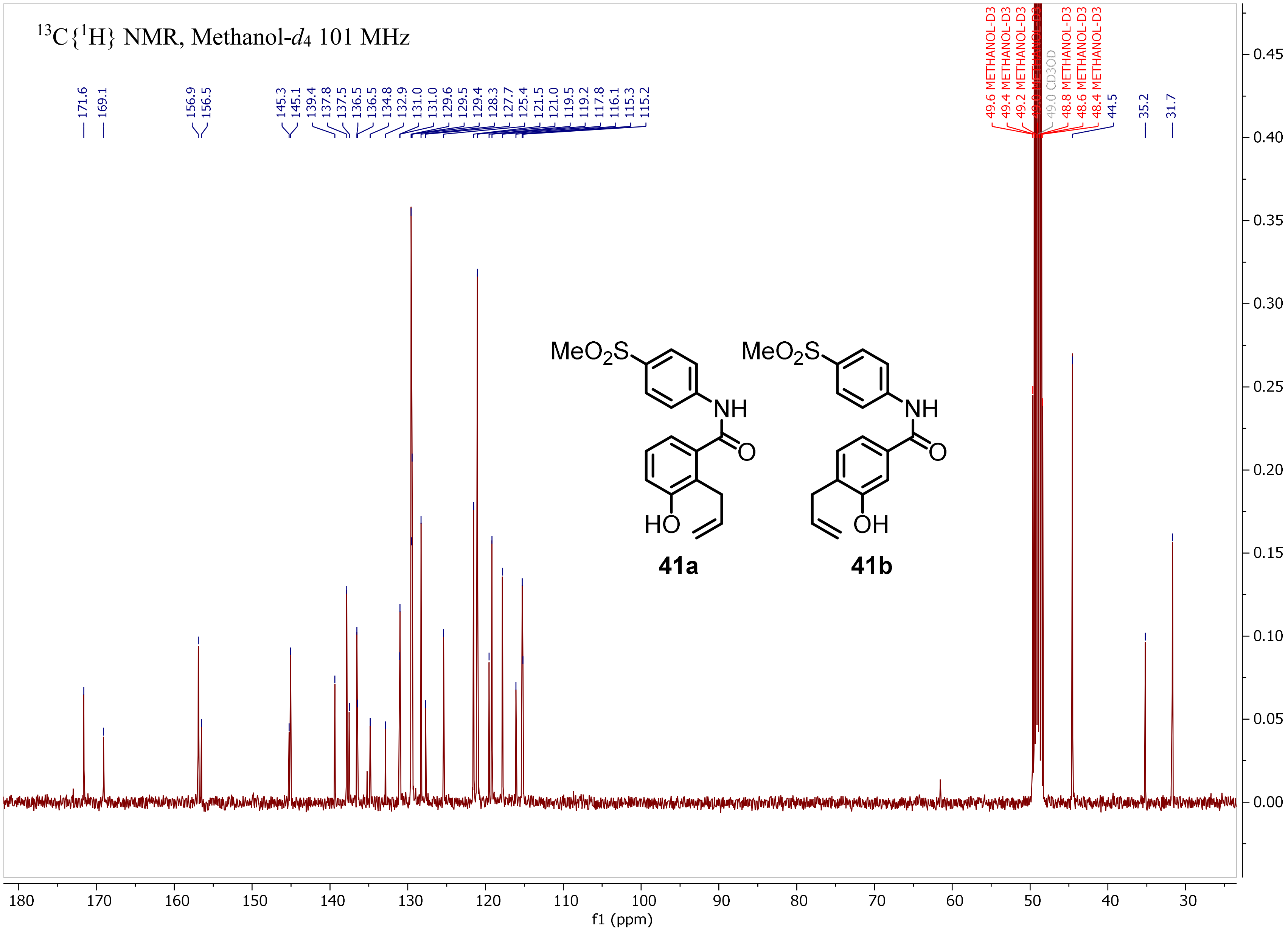

$^{13}C\{^{1}H\}$ NMR, Methanol-d_4 101 MHz
171.6
169.1
156.9
156.5
145.3
145.1
139.4
137.8
137.5
136.5
136.5
134.8
132.9
131.0
131.0
129.6
129.5
129.4
128.3
127.7
125.4
121.5
121.0
119.5
119.2
117.8
116.1
115.3
115.2
49.6 METHANOL-D3
49.4 METHANOL-D3
49.2 METHANOL-D3
49.0 CD3OD
48.8 METHANOL-D3
48.6 METHANOL-D3
48.4 METHANOL-D3
44.5
35.2
31.7
MeO2S
NH
O
HO
OH
41a
41b
f1 (ppm)

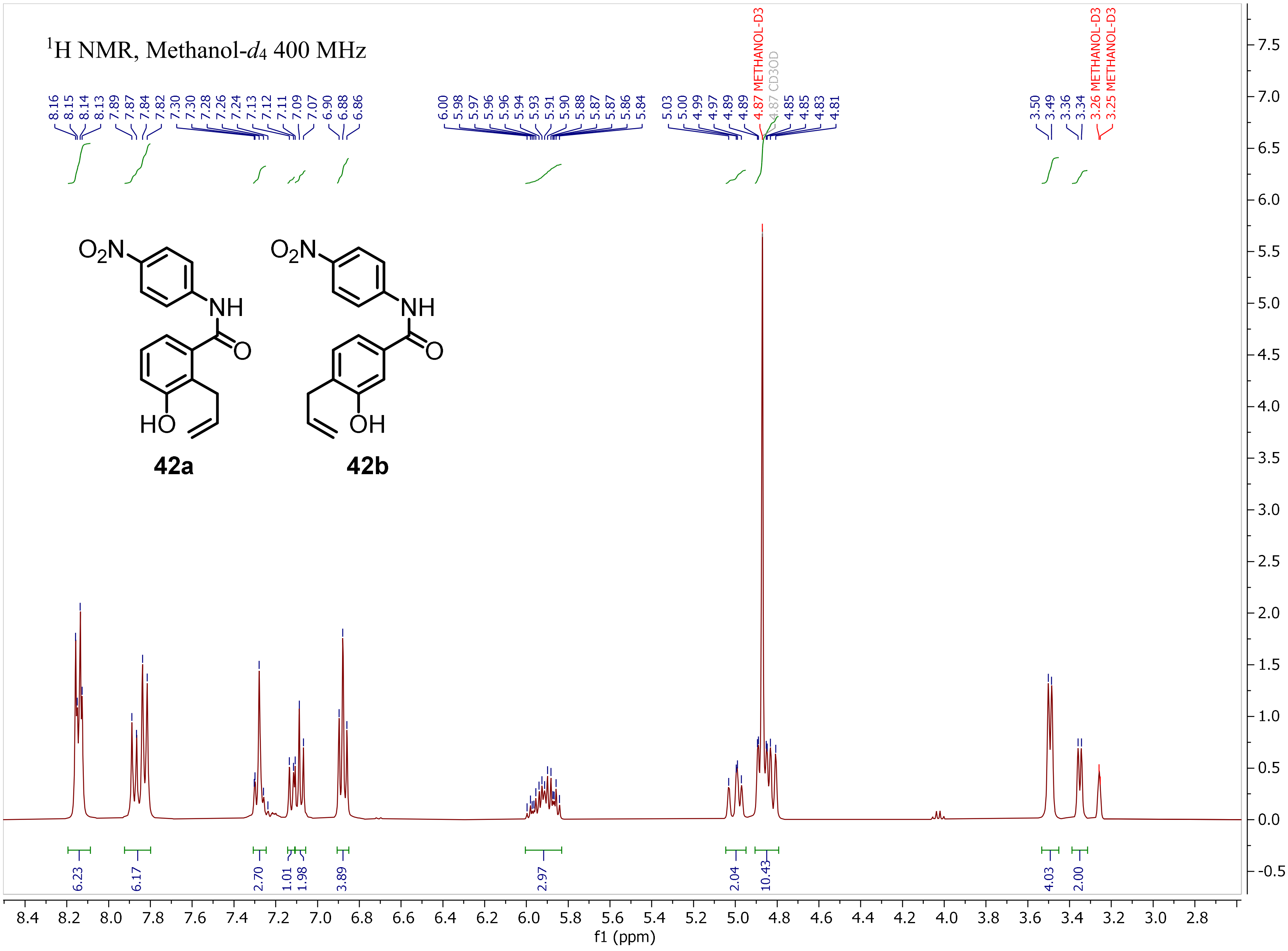

^{1}H NMR, Methanol-d_4 400 MHz
42a
42b
f1 (ppm)

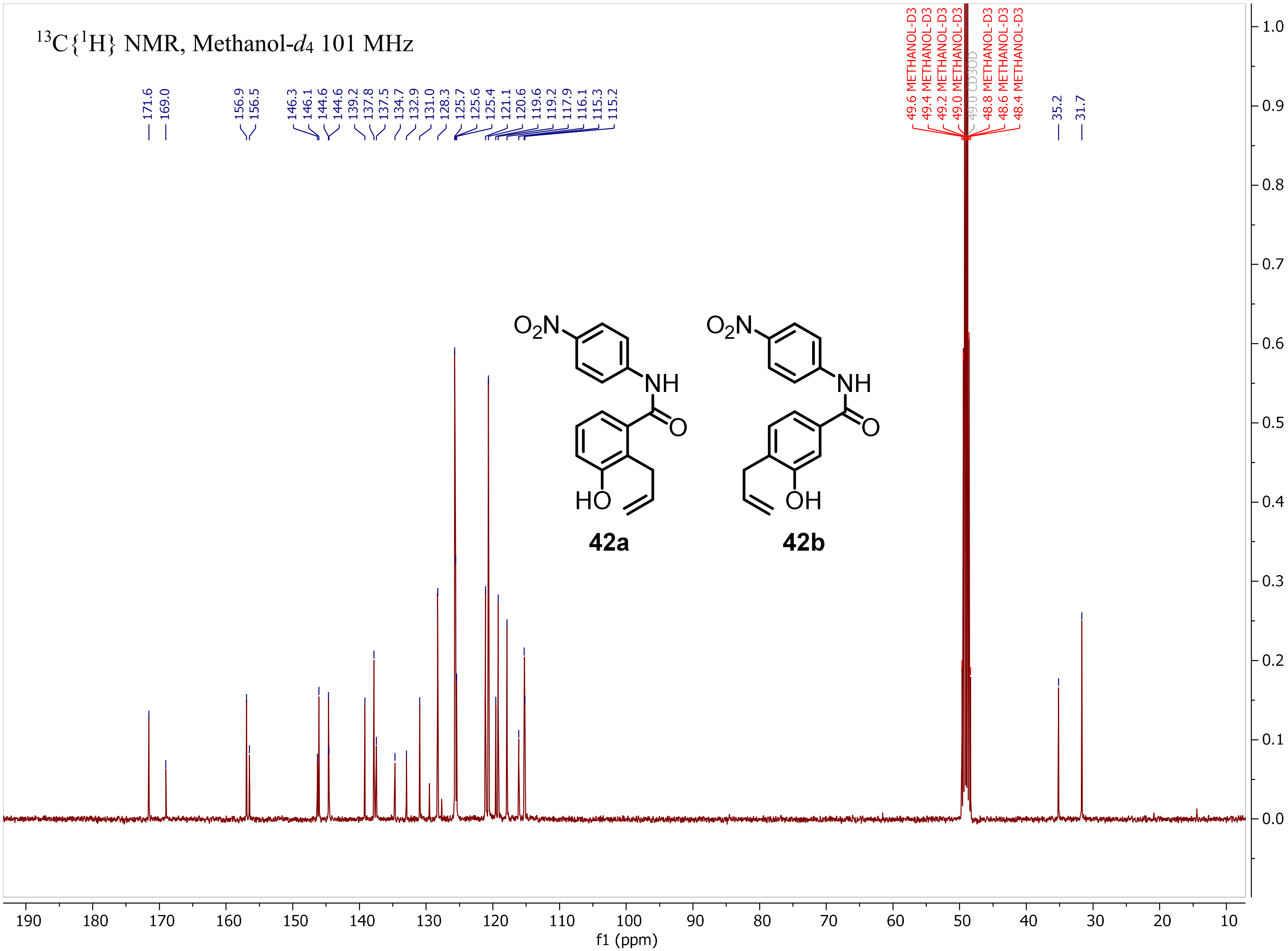

$^{13}C\{^1H\}$ NMR, Methanol-d_4 101 MHz
171.6
169.0
156.9
156.5
146.3
146.1
144.6
144.6
139.2
137.8
137.5
134.7
132.9
131.0
128.3
125.7
125.6
125.4
121.1
120.6
119.6
119.2
117.9
116.1
115.3
115.2
49.6 METHANOL-D3
49.4 METHANOL-D3
49.2 METHANOL-D3
49.0 METHANOL-D3
49.0 CD3OD
48.8 METHANOL-D3
48.6 METHANOL-D3
48.4 METHANOL-D3
35.2
31.7
O2N
NH
O
HO
OH
42a
42b
f1 (ppm)

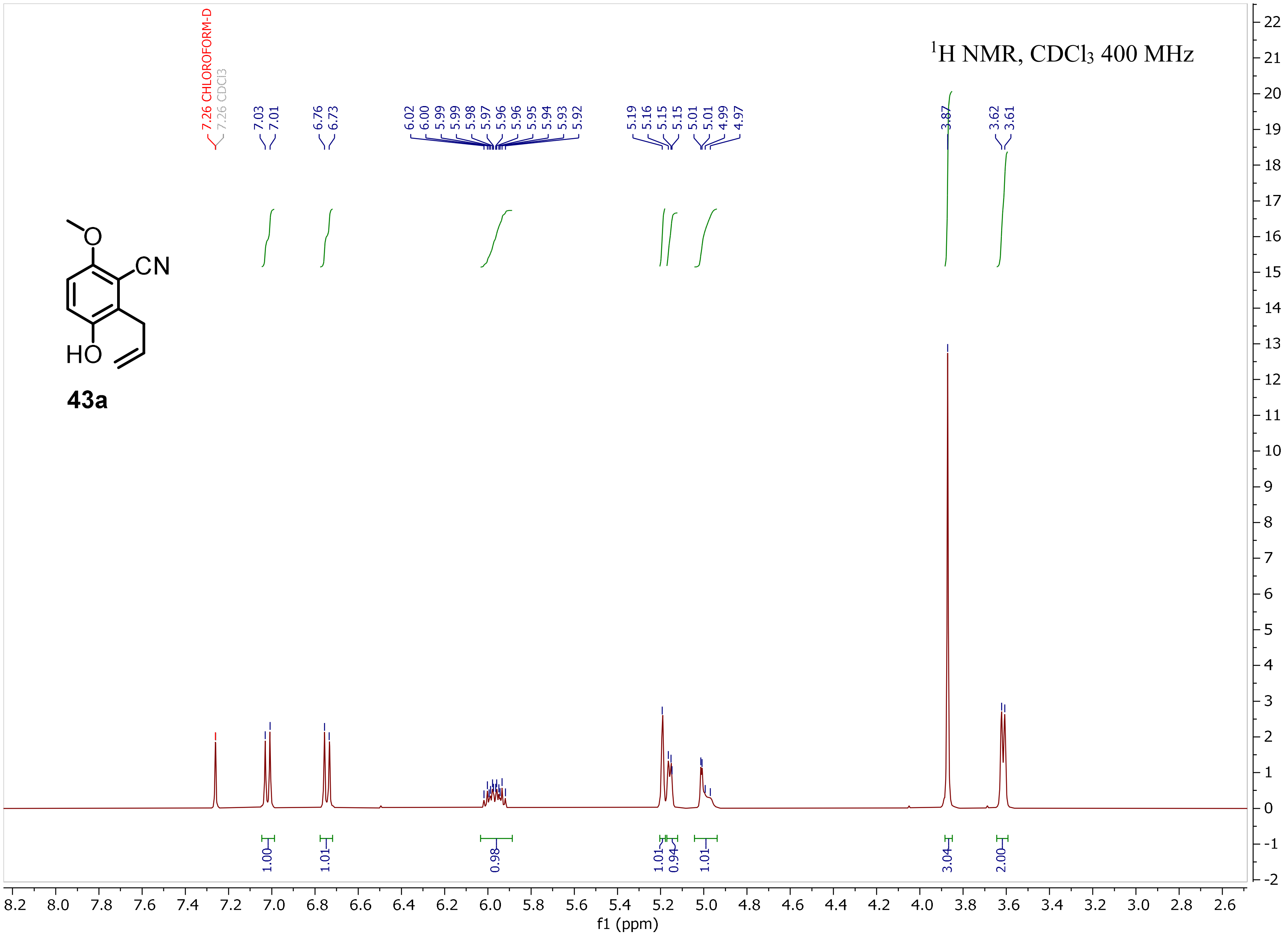

7.26 CHLOROFORM-D
7.26 CDCl3
7.03
7.01
6.76
6.73
6.02
6.00
5.99
5.99
5.98
5.97
5.96
5.96
5.95
5.94
5.93
5.92
5.19
5.16
5.15
5.15
5.01
5.01
4.99
4.97
3.87
3.62
3.61
^{1}H NMR, $CDCl_3$ 400 MHz
CN
O
HO
43a
1.00
1.01
0.98
1.01
0.94
1.01
3.04
2.00
f1 (ppm)

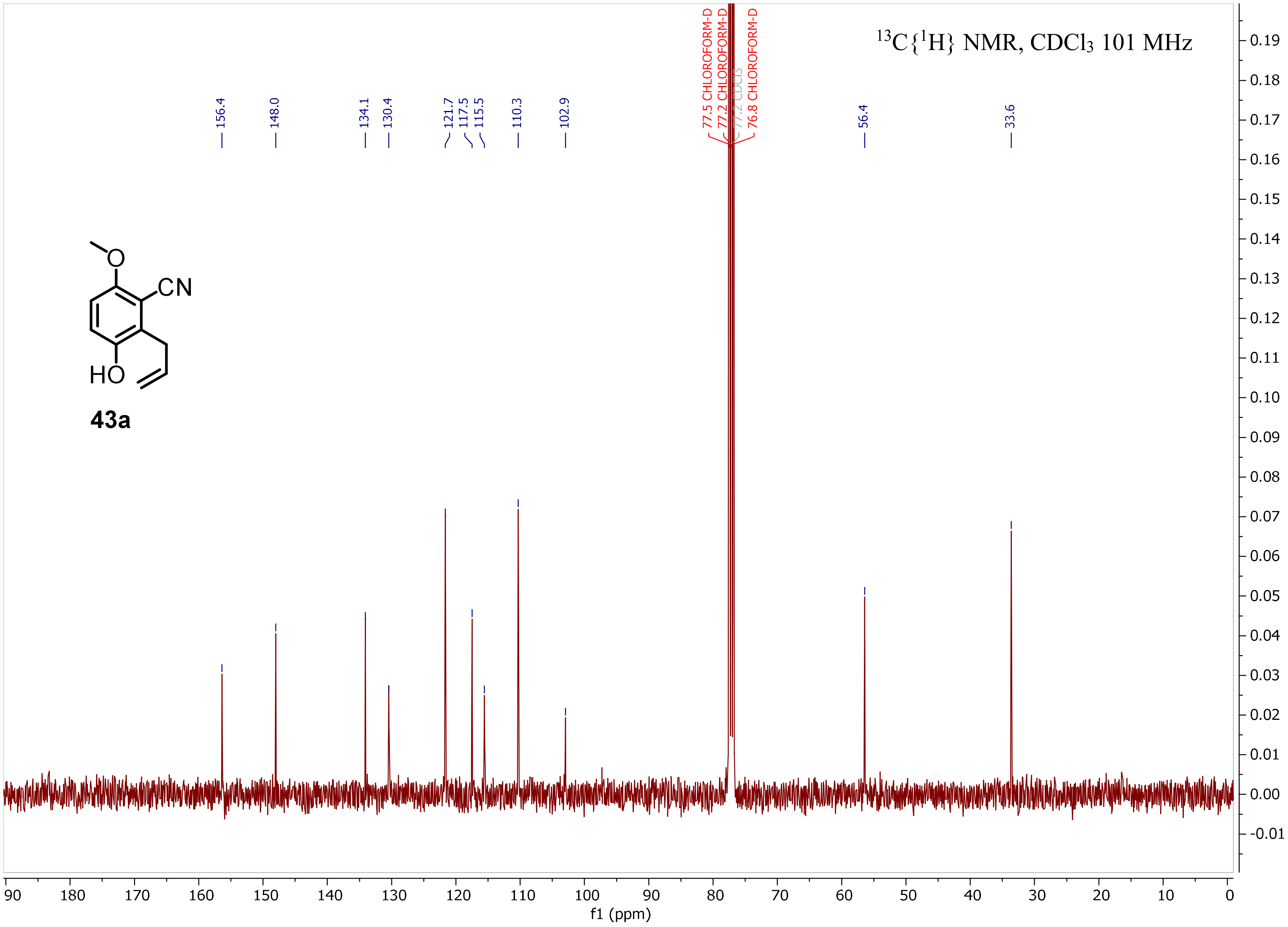

$^{13}C\{^{1}H\}$ NMR, $CDCl_3$ 101 MHz
156.4
148.0
134.1
130.4
121.7
117.5
115.5
110.3
102.9
77.5 CHLOROFORM-D
77.2 CHLOROFORM-D
77.2 CDCl3
76.8 CHLOROFORM-D
56.4
33.6
f1 (ppm)
O
CN
HO

43a

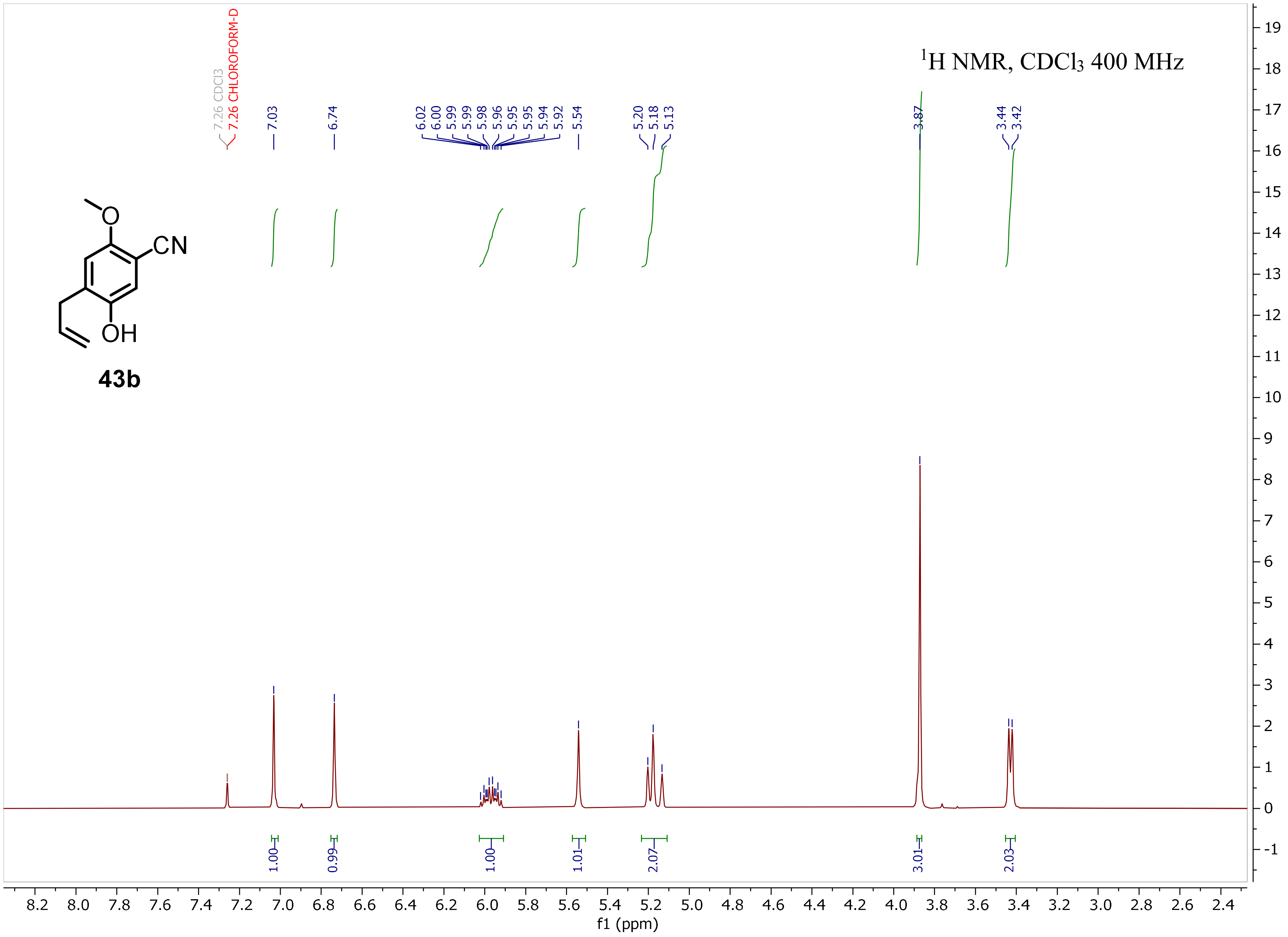

1H NMR, CDCl3 400 MHz
7.26 CDCl3
7.26 CHLOROFORM-D
7.03
6.74
6.02
6.00
5.99
5.99
5.98
5.96
5.95
5.95
5.94
5.92
5.54
5.20
5.18
5.13
3.87
3.44
3.42
CN
O
OH
43b
1.00
0.99
1.00
1.01
2.07
3.01
2.03
f1 (ppm)

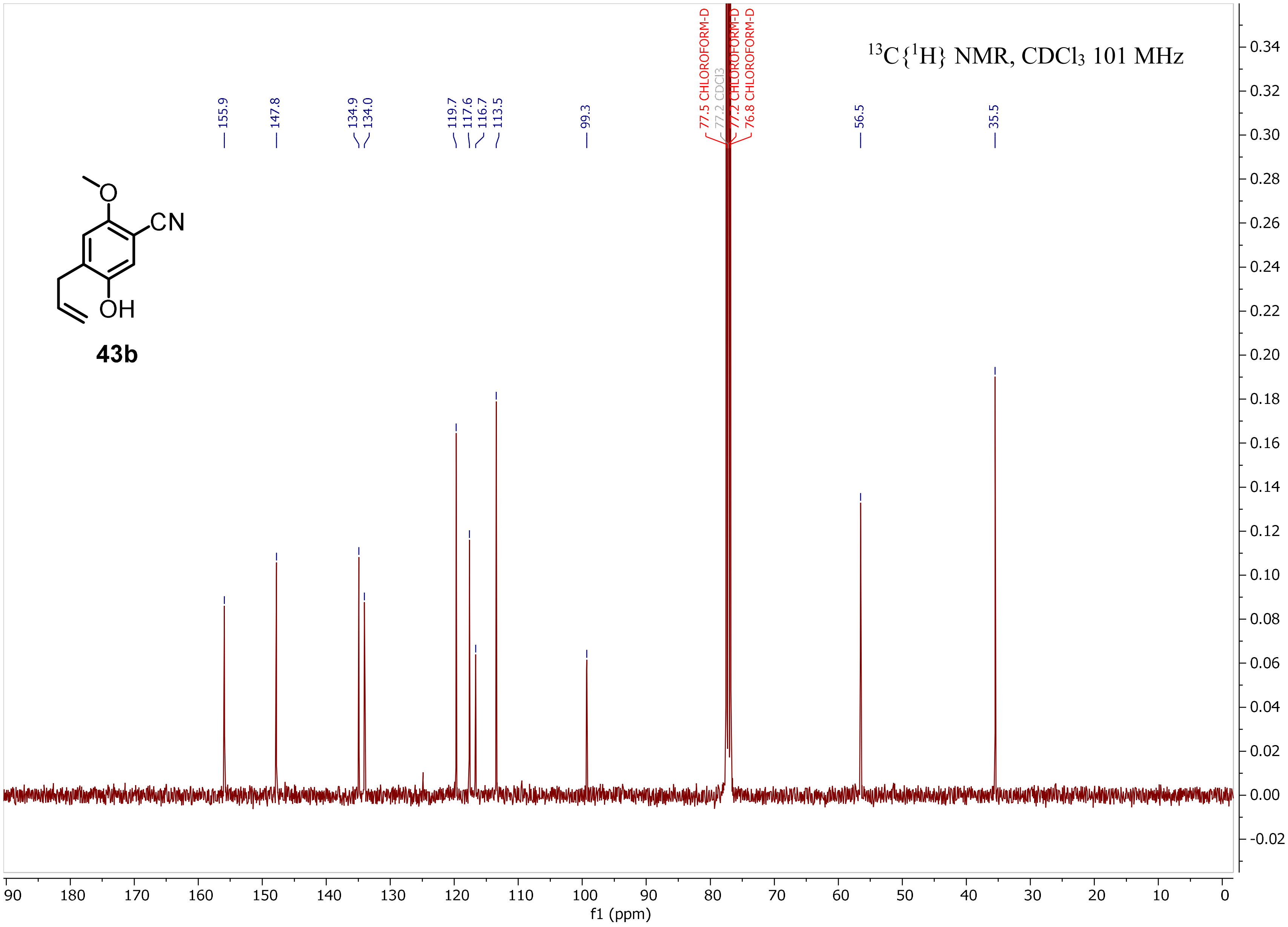

^{13}C{^{1}H} NMR, CDCl$_3$ 101 MHz
155.9
147.8
134.9
134.0
119.7
117.6
116.7
113.5
99.3
77.5 CHLOROFORM-D
77.2 CDCl3
77.2 CHLOROFORM-D
76.8 CHLOROFORM-D
56.5
35.5
CN
O
OH
43b
f1 (ppm)

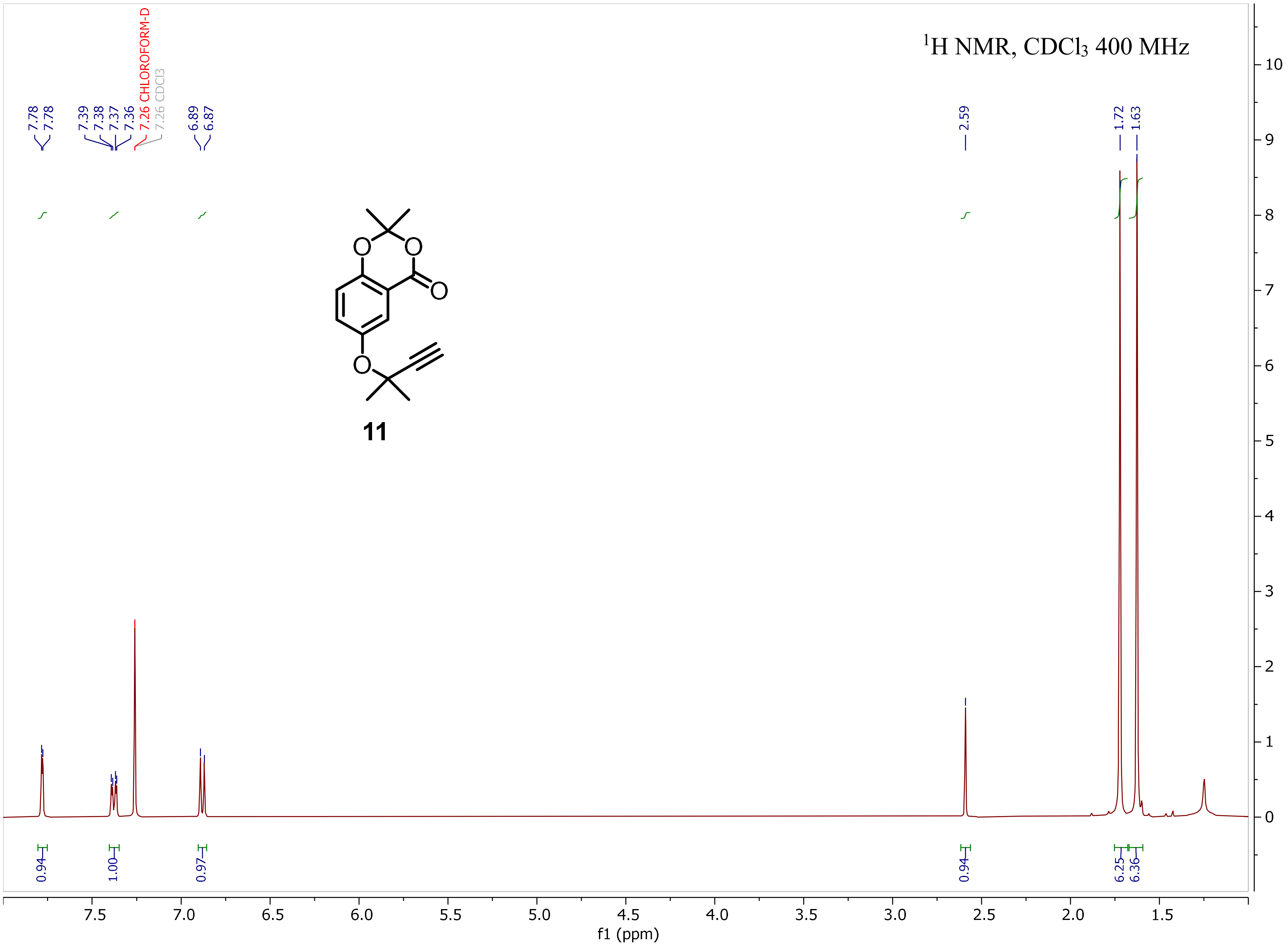

^{1}H NMR, CDCl3 400 MHz
7.78
7.78
7.39
7.38
7.37
7.36
7.26 CHLOROFORM-D
7.26 CDCl3
6.89
6.87
2.59
1.72
1.63
11
0.94
1.00
0.97
0.94
6.25
6.36
f1 (ppm)

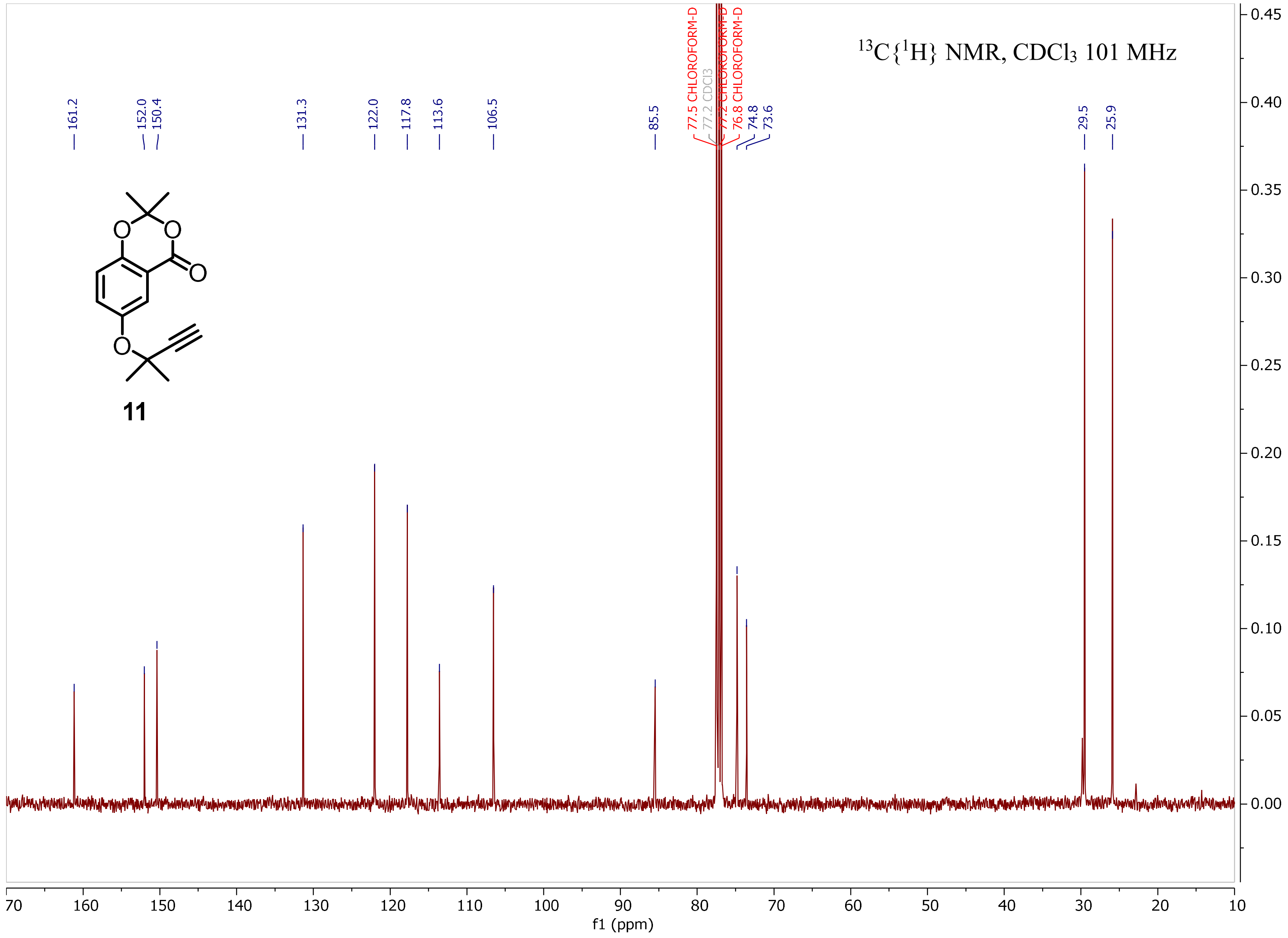

13C{1H} NMR, CDCl3 101 MHz
161.2
152.0
150.4
131.3
122.0
117.8
113.6
106.5
85.5
77.5 CHLOROFORM-D
77.2 CDCl3
77.2 CHLOROFORM-D
76.8 CHLOROFORM-D
74.8
73.6
29.5
25.9
11
f1 (ppm)

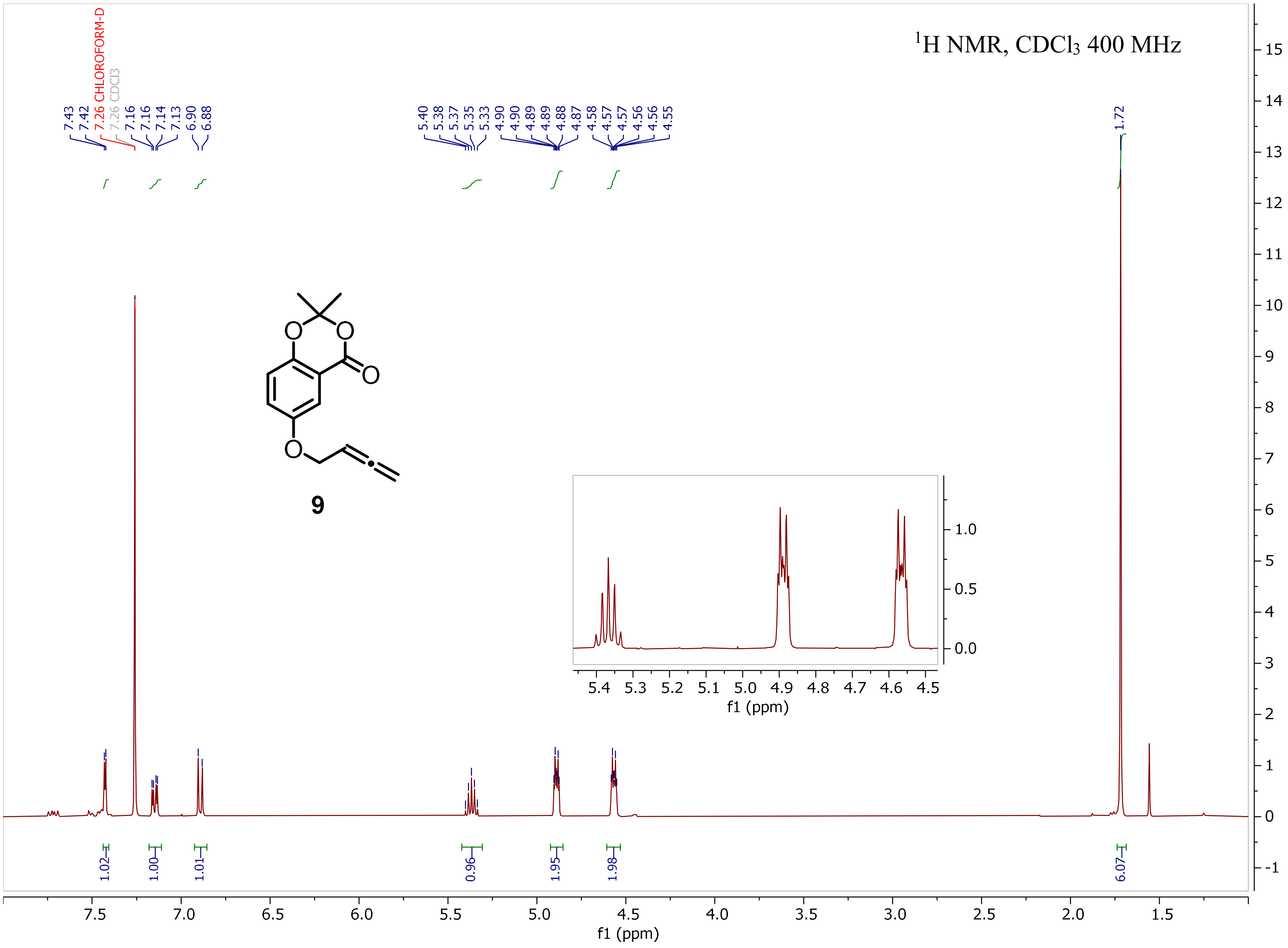

^{1}H NMR, CDCl$_3$ 400 MHz
9
f1 (ppm)

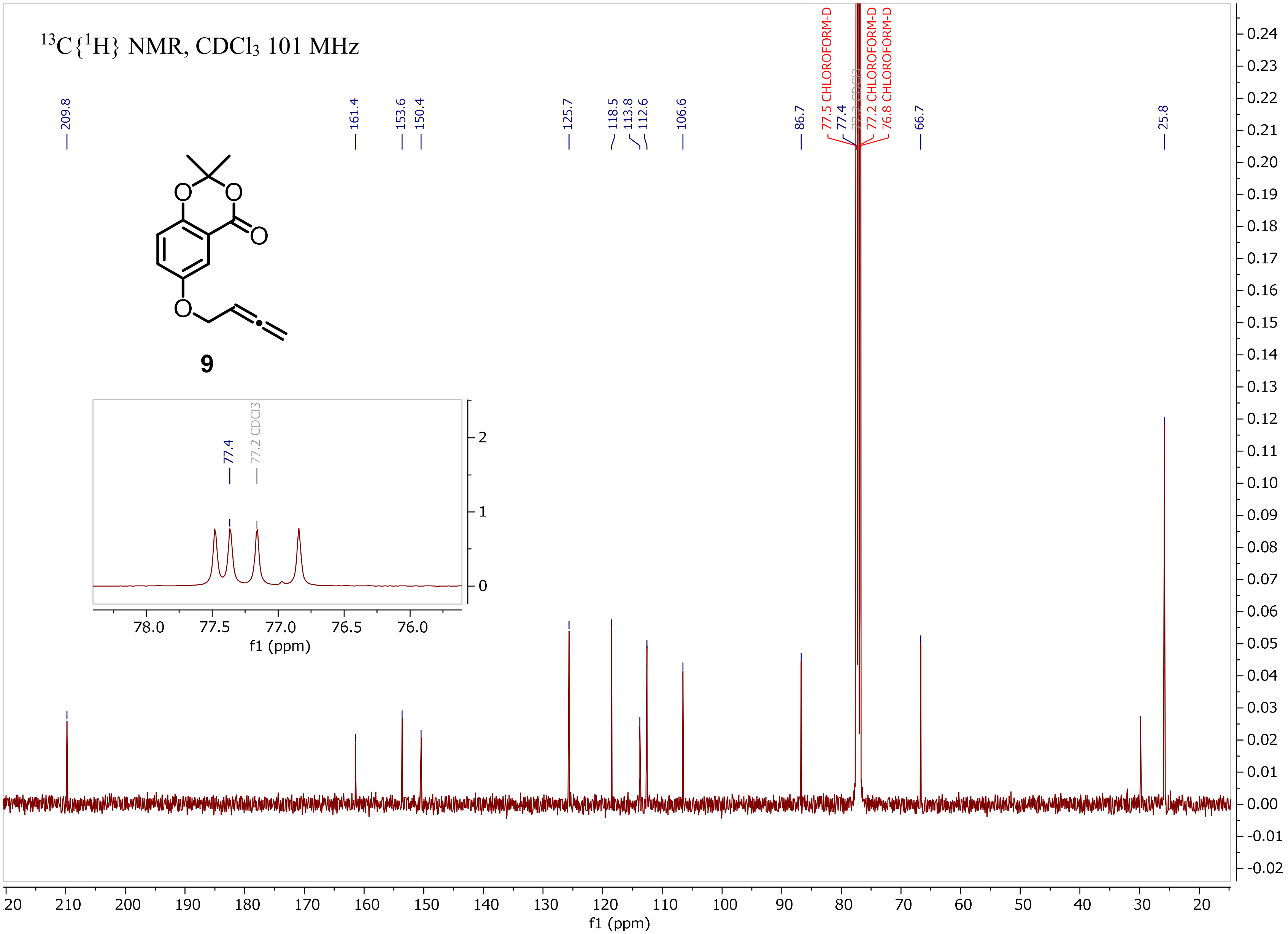

13C{1H} NMR, CDCl3 101 MHz
209.8
161.4
153.6
150.4
125.7
118.5
113.8
112.6
106.6
86.7
77.5 CHLOROFORM-D
77.4
77.2 CDCl3
77.2 CHLOROFORM-D
76.8 CHLOROFORM-D
66.7
25.8
9
f1 (ppm)

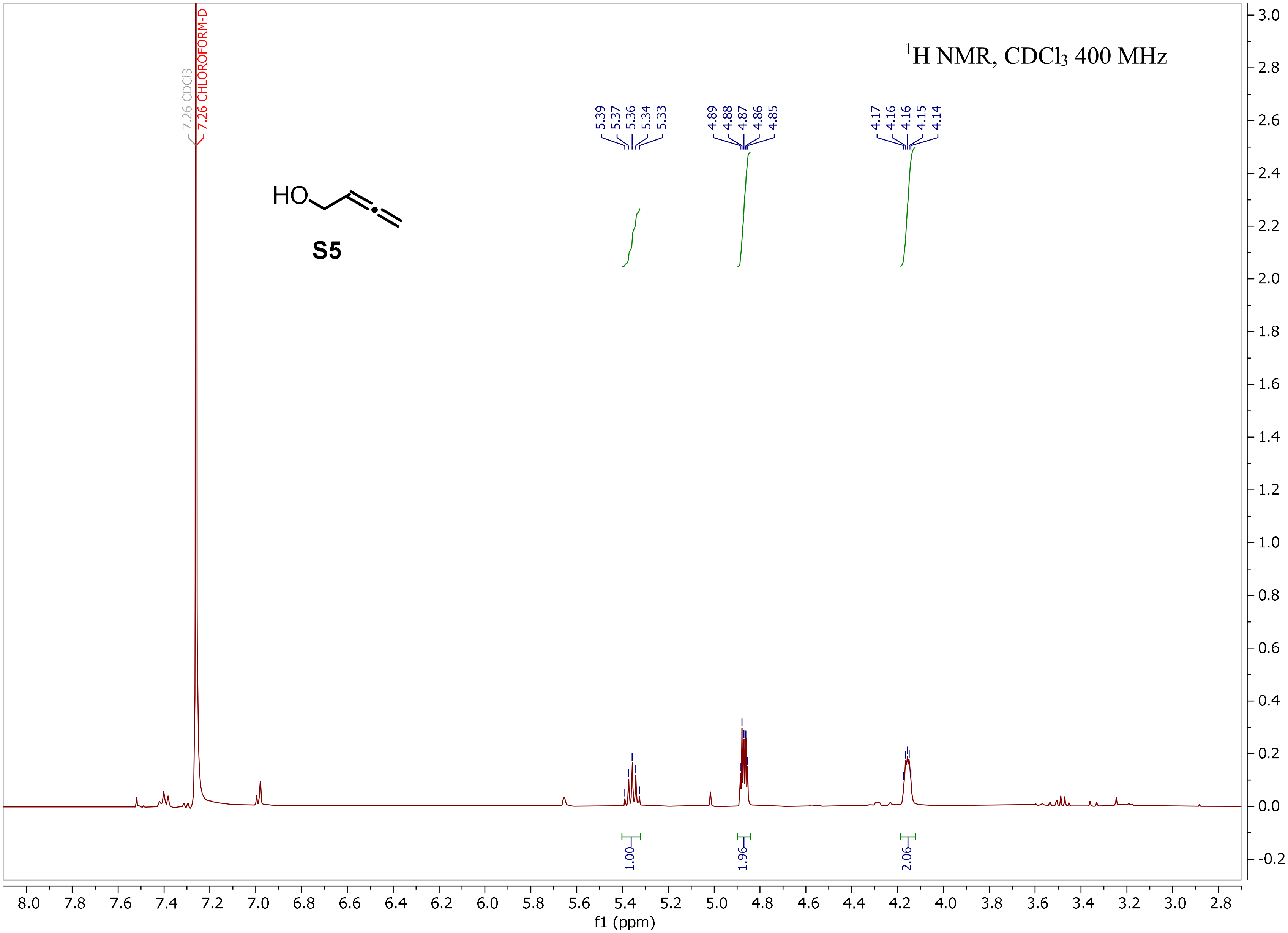

S5
¹H NMR, CDCl3 400 MHz
7.26 CDCl3
7.26 CHLOROFORM-D
5.39
5.37
5.36
5.34
5.33
4.89
4.88
4.87
4.86
4.85
4.17
4.16
4.16
4.15
4.14
1.00
1.96
2.06
f1 (ppm)

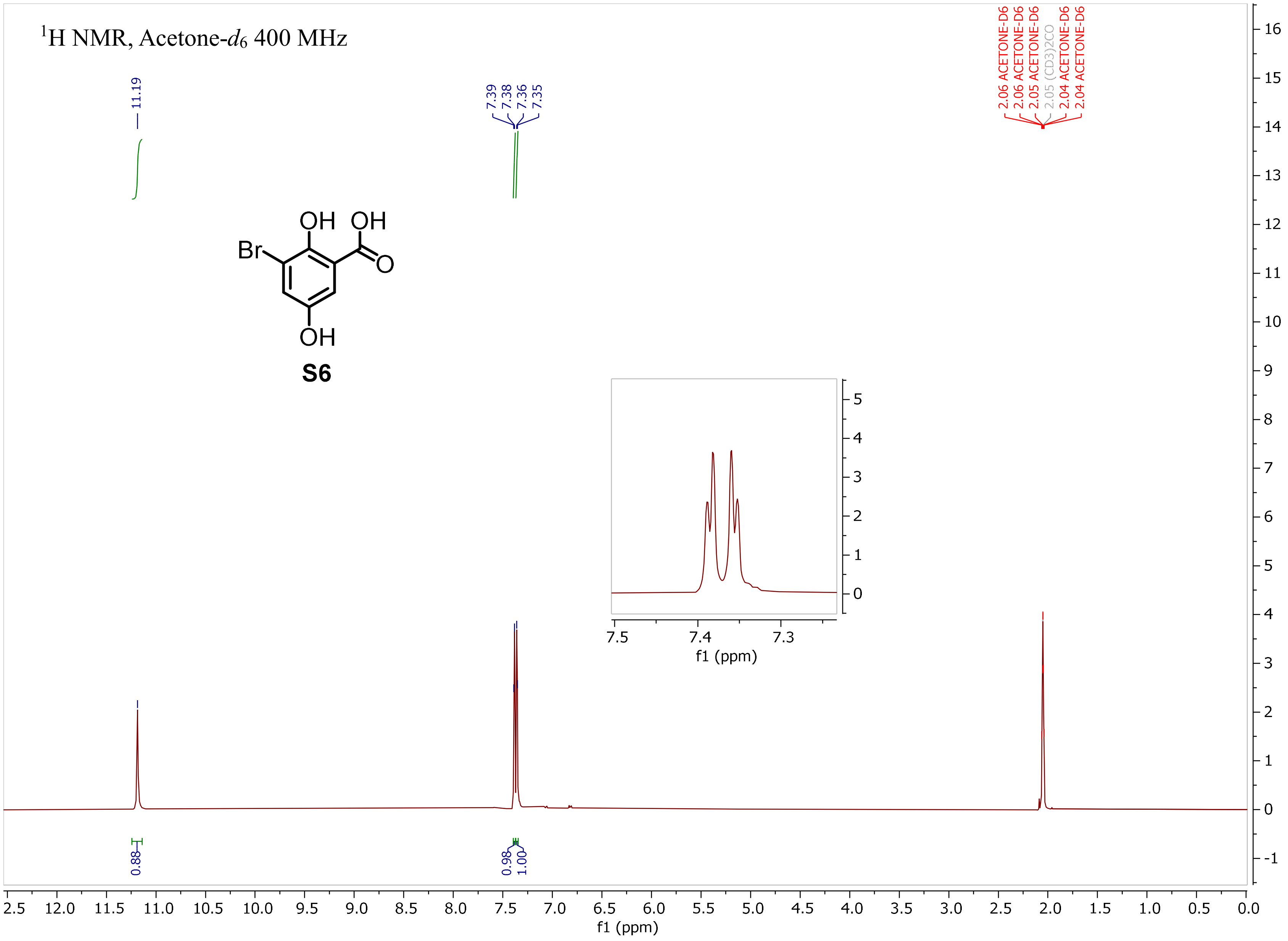

^{1}H NMR, Acetone-d_6 400 MHz
11.19
7.39
7.38
7.36
7.35
2.06 ACETONE-D6
2.06 ACETONE-D6
2.05 ACETONE-D6
2.05 (CD3)2CO
2.04 ACETONE-D6
2.04 ACETONE-D6
S6
0.88
0.98
1.00
f1 (ppm)

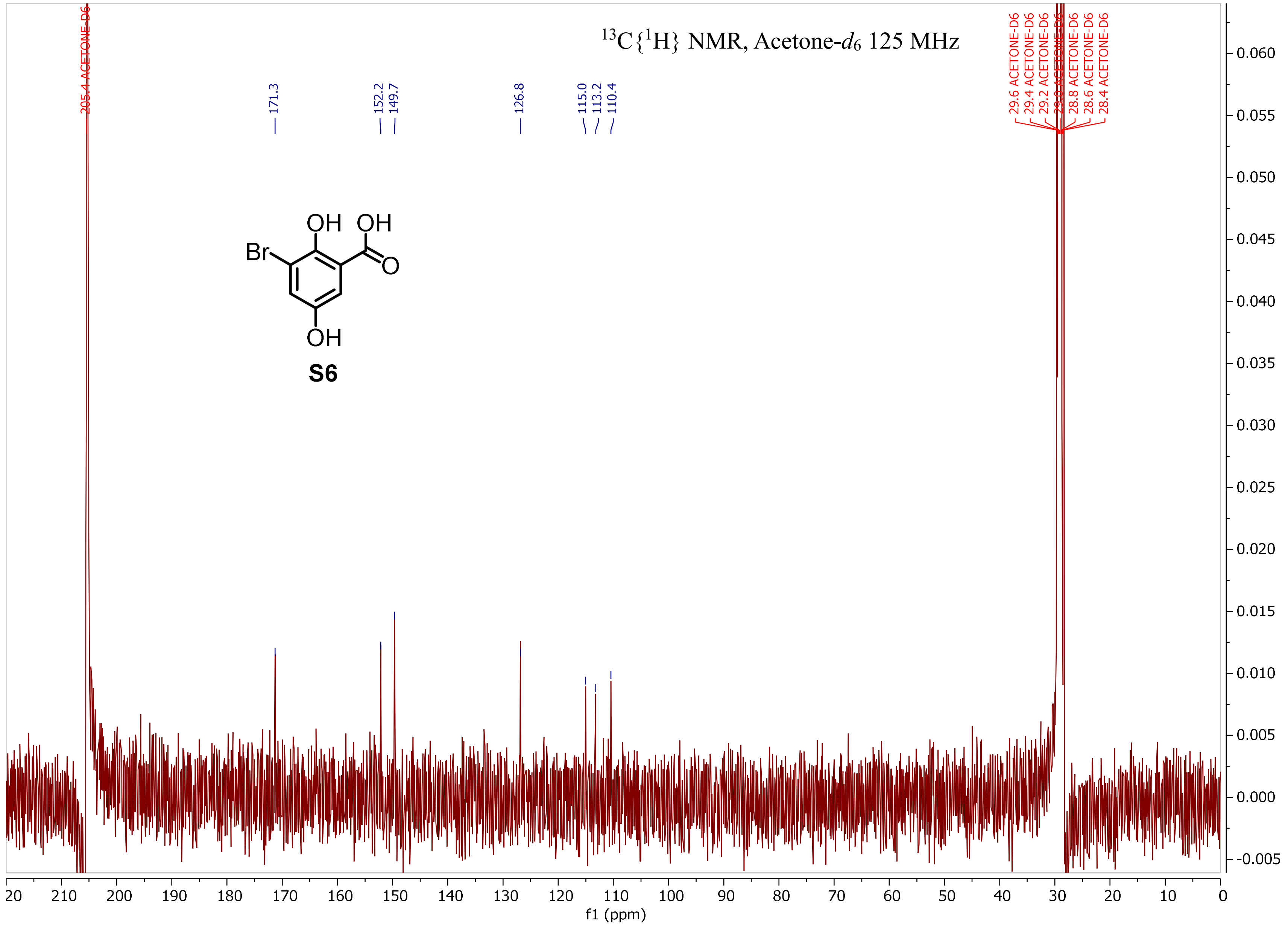

$^{13}C\{^{1}H\}$ NMR, Acetone-d_6 125 MHz
205.4 ACETONE-D6
171.3
152.2
149.7
126.8
115.0
113.2
110.4
29.6 ACETONE-D6
29.4 ACETONE-D6
29.2 ACETONE-D6
29.0 ACETONE-D6
28.8 ACETONE-D6
28.6 ACETONE-D6
28.4 ACETONE-D6
OH
OH
Br
O
OH
S6
f1 (ppm)

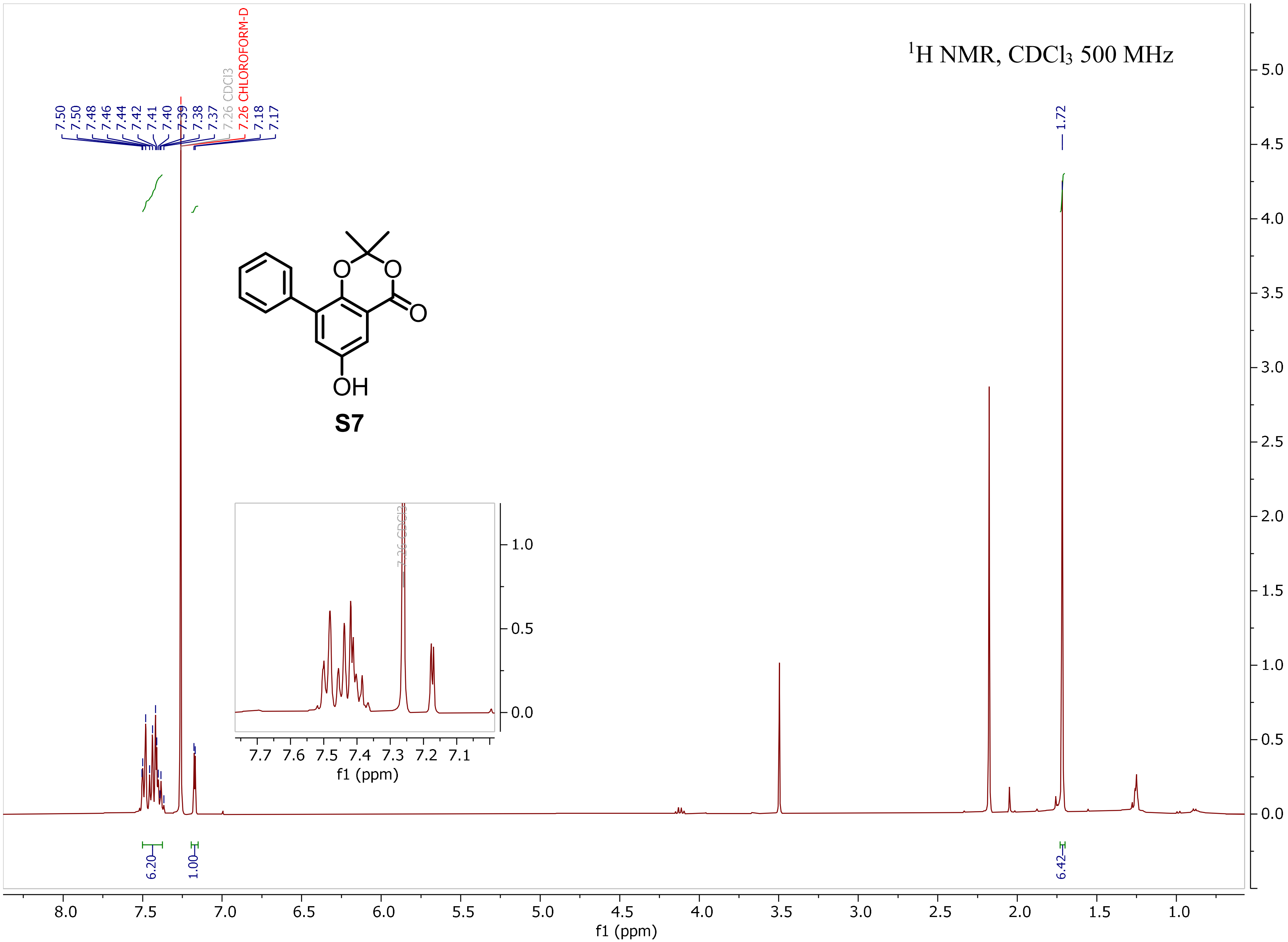

^{1}H NMR, CDCl3 500 MHz
7.50
7.50
7.48
7.46
7.44
7.42
7.41
7.40
7.39
7.38
7.37
7.26 CDCl3
7.26 CHLOROFORM-D
7.18
7.17
1.72
OH
S7
6.20
1.00
6.42
f1 (ppm)

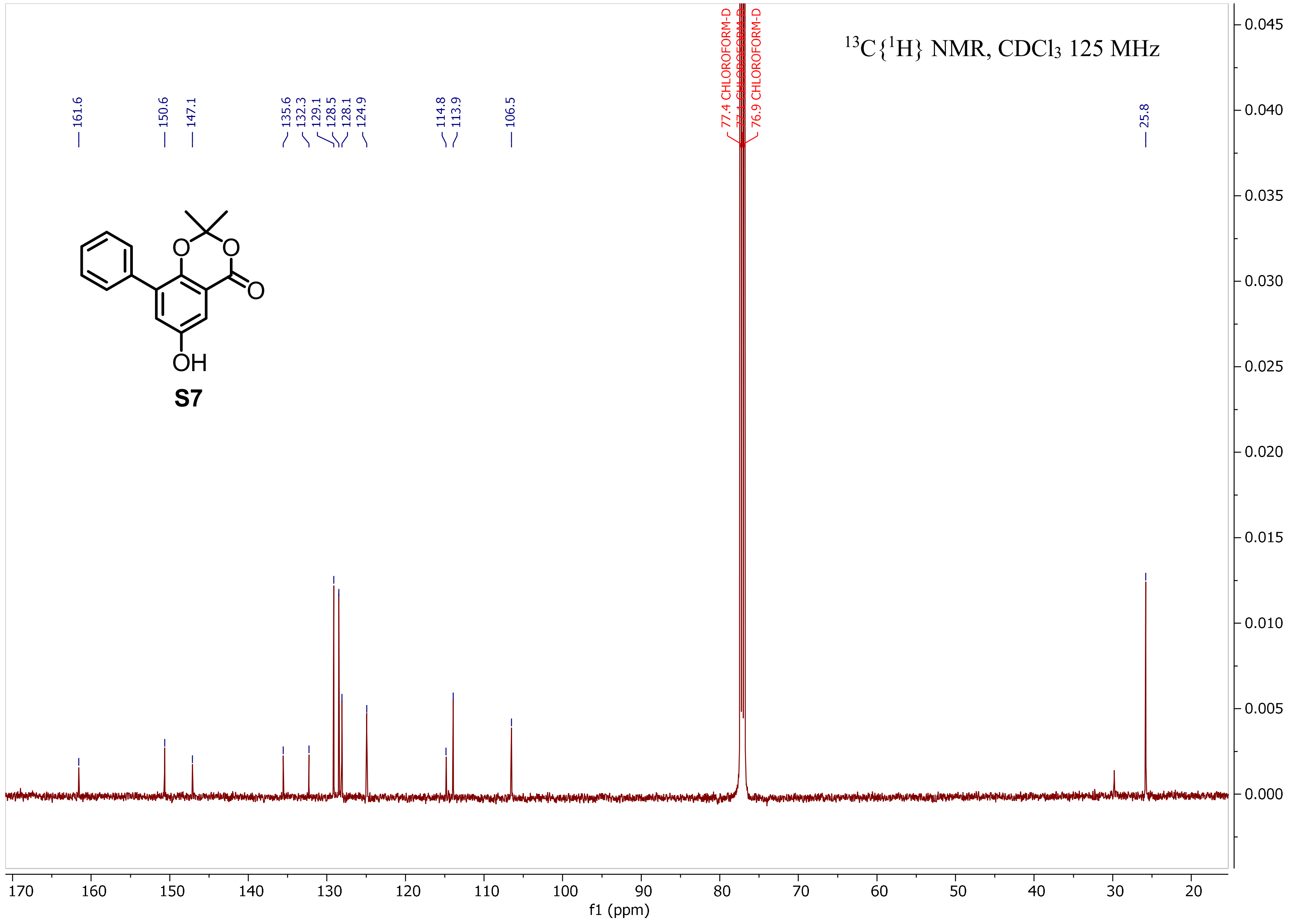

$^{13}C\{^1H\}$ NMR, $CDCl_3$ 125 MHz
161.6
150.6
147.1
135.6
132.3
129.1
128.5
128.1
124.9
114.8
113.9
106.5
77.4 CHLOROFORM-D
76.9 CHLOROFORM-D
25.8
OH
S7
f1 (ppm)

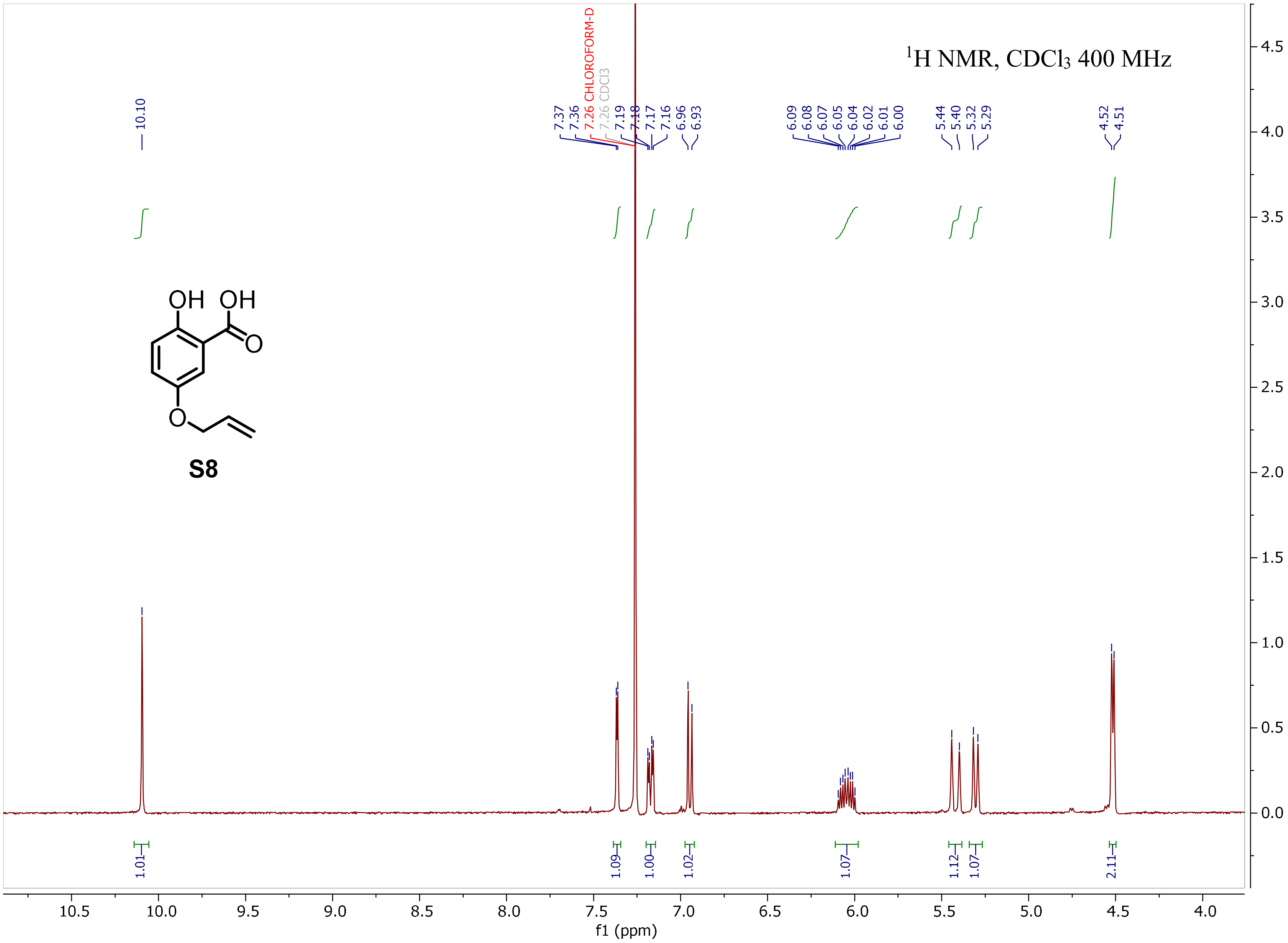

1H NMR, CDCl3 400 MHz
S8
OH
OH
O
O
f1 (ppm)

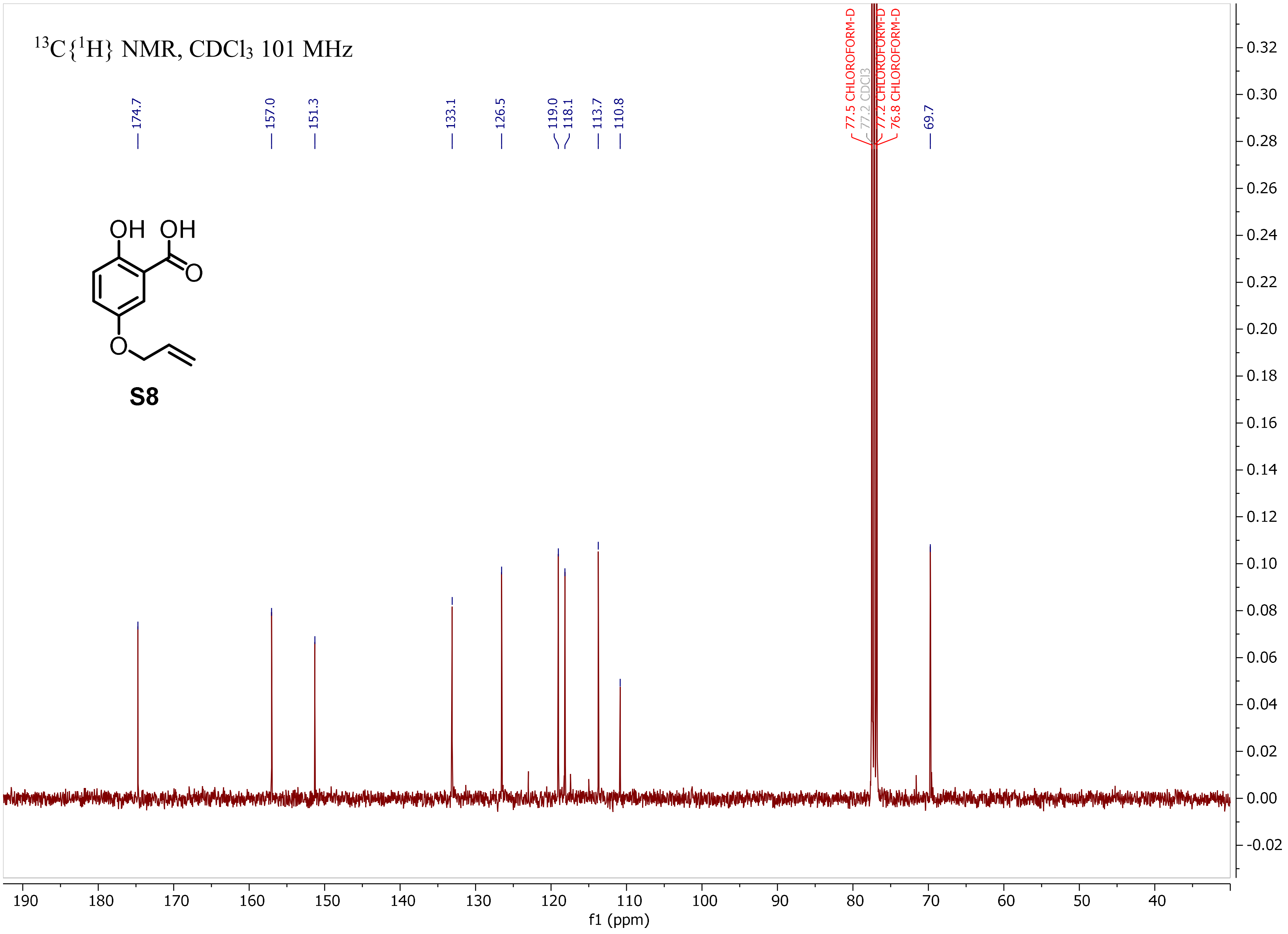

$^{13}C\{^1H\}$ NMR, $CDCl_3$ 101 MHz
174.7
157.0
151.3
133.1
126.5
119.0
118.1
113.7
110.8
77.5 CHLOROFORM-D
77.2 CDCl3
77.2 CHLOROFORM-D
76.8 CHLOROFORM-D
69.7
OH
OH
O
O
S8
190
180
170
160
150
140
130
120
110
100
90
80
70
60
50
40
f1 (ppm)
0.32
0.30
0.28
0.26
0.24
0.22
0.20
0.18
0.16
0.14
0.12
0.10
0.08
0.06
0.04
0.02
0.00
-0.02

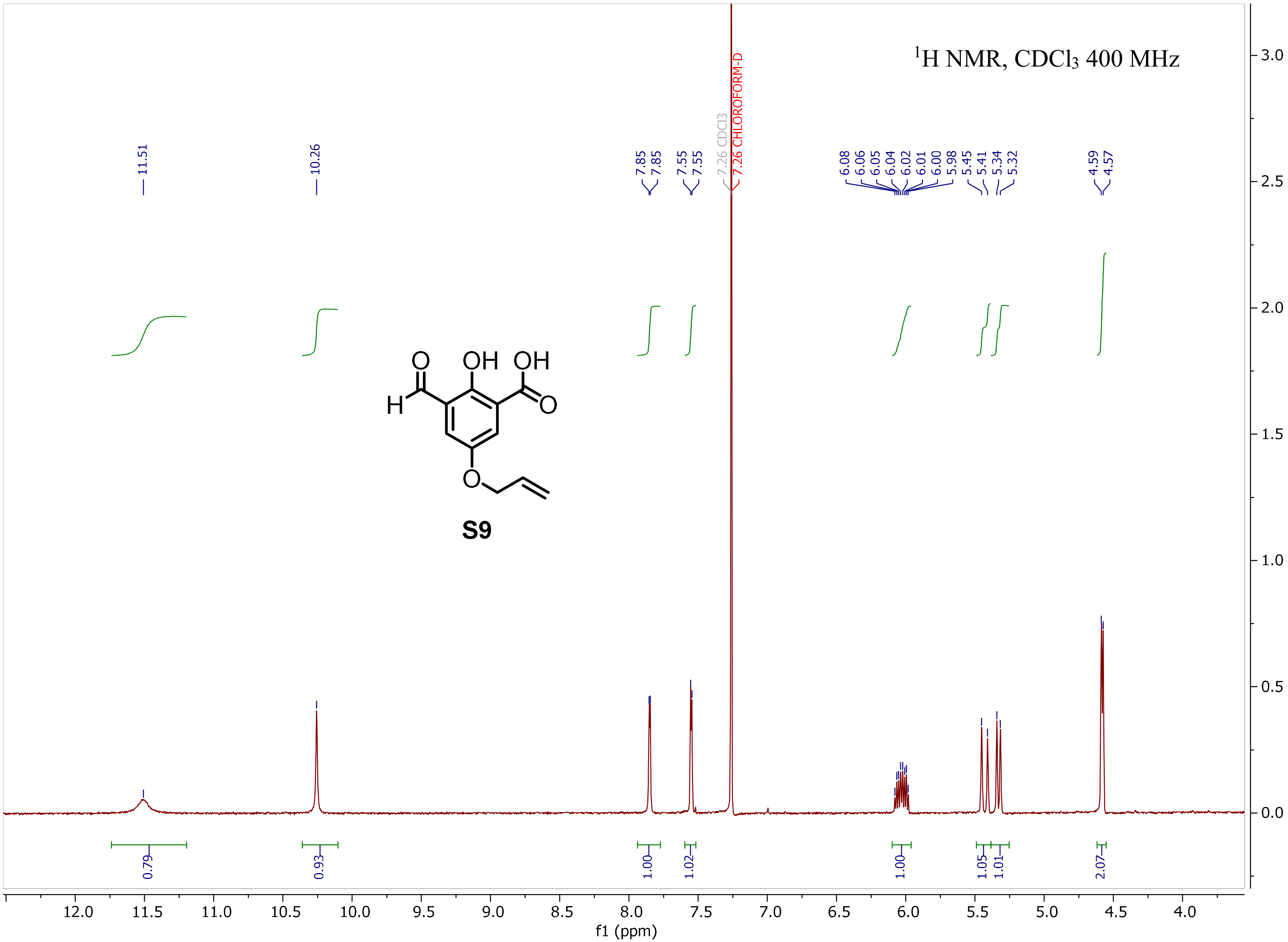

^{1}H NMR, CDCl$_3$ 400 MHz
11.51
10.26
7.85
7.85
7.55
7.55
7.26 CDCl3
7.26 CHLOROFORM-D
6.08
6.06
6.05
6.04
6.02
6.01
6.00
5.98
5.45
5.41
5.34
5.32
4.59
4.57
S9
0.79
0.93
1.00
1.02
1.00
1.05
1.01
2.07
12.0 11.5 11.0 10.5 10.0 9.5 9.0 8.5 8.0 7.5 7.0 6.5 6.0 5.5 5.0 4.5 4.0
f1 (ppm)
3.0 2.5 2.0 1.5 1.0 0.5 0.0

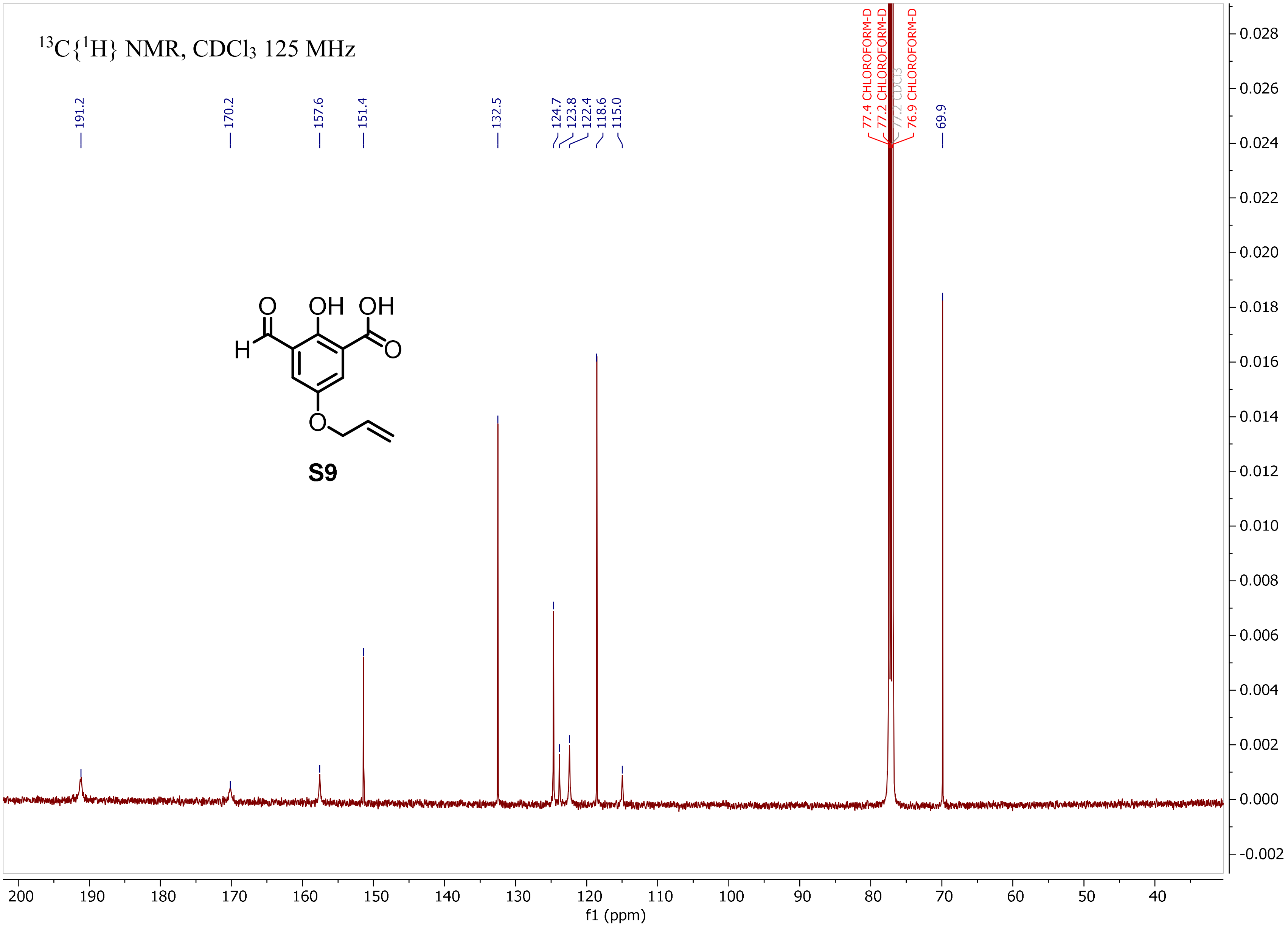

13C{1H} NMR, CDCl3 125 MHz
191.2
170.2
157.6
151.4
132.5
124.7
123.8
122.4
118.6
115.0
77.4 CHLOROFORM-D
77.2 CHLOROFORM-D
77.2 CDCl3
76.9 CHLOROFORM-D
69.9
O
OH
OH
H
O
O
S9
f1 (ppm)

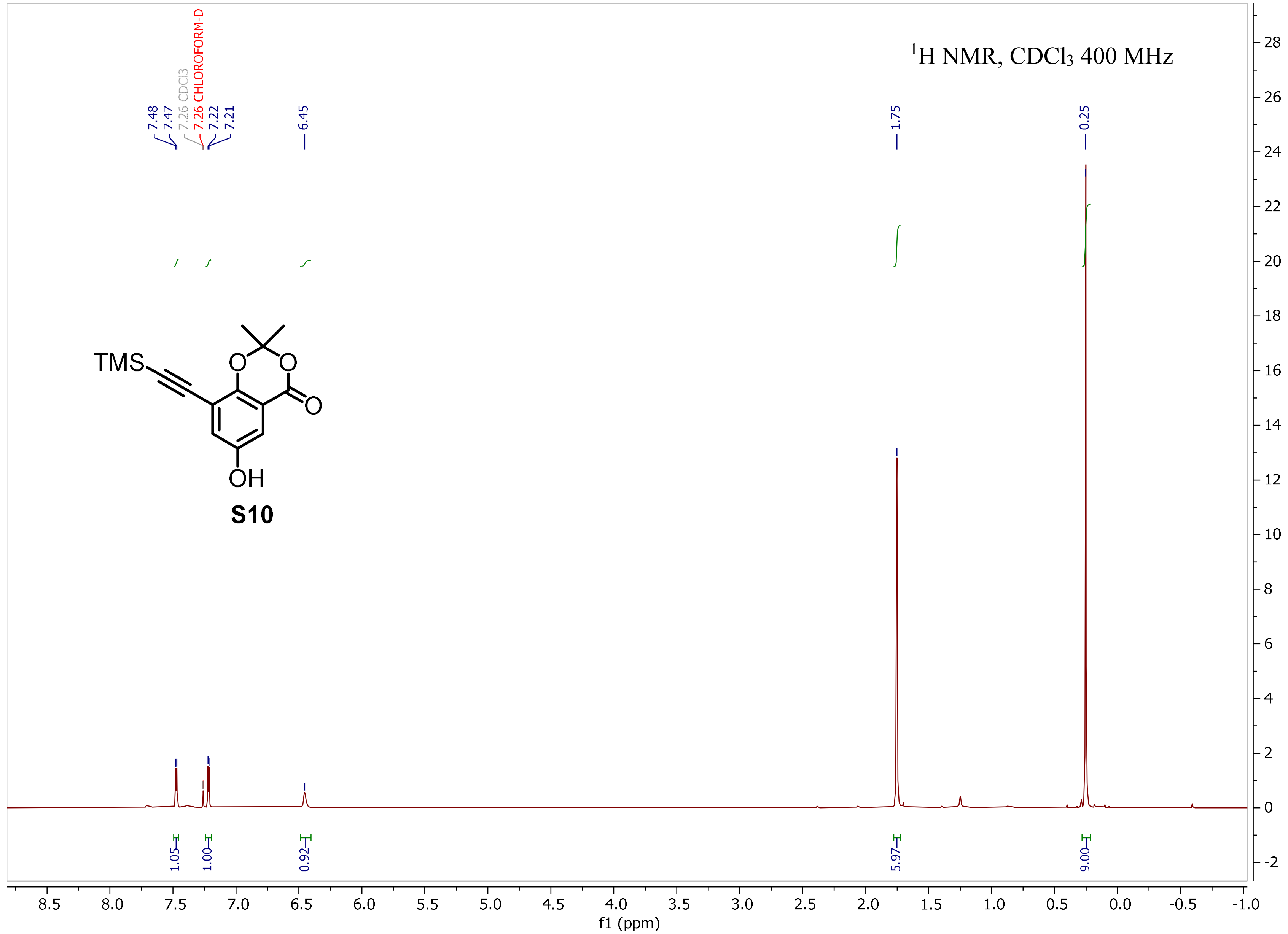

1H NMR, CDCl3 400 MHz
7.48
7.47
7.26 CDCl3
7.26 CHLOROFORM-D
7.22
7.21
6.45
1.75
0.25
TMS
O
O
O
OH
S10
1.05
1.00
0.92
5.97
9.00
f1 (ppm)

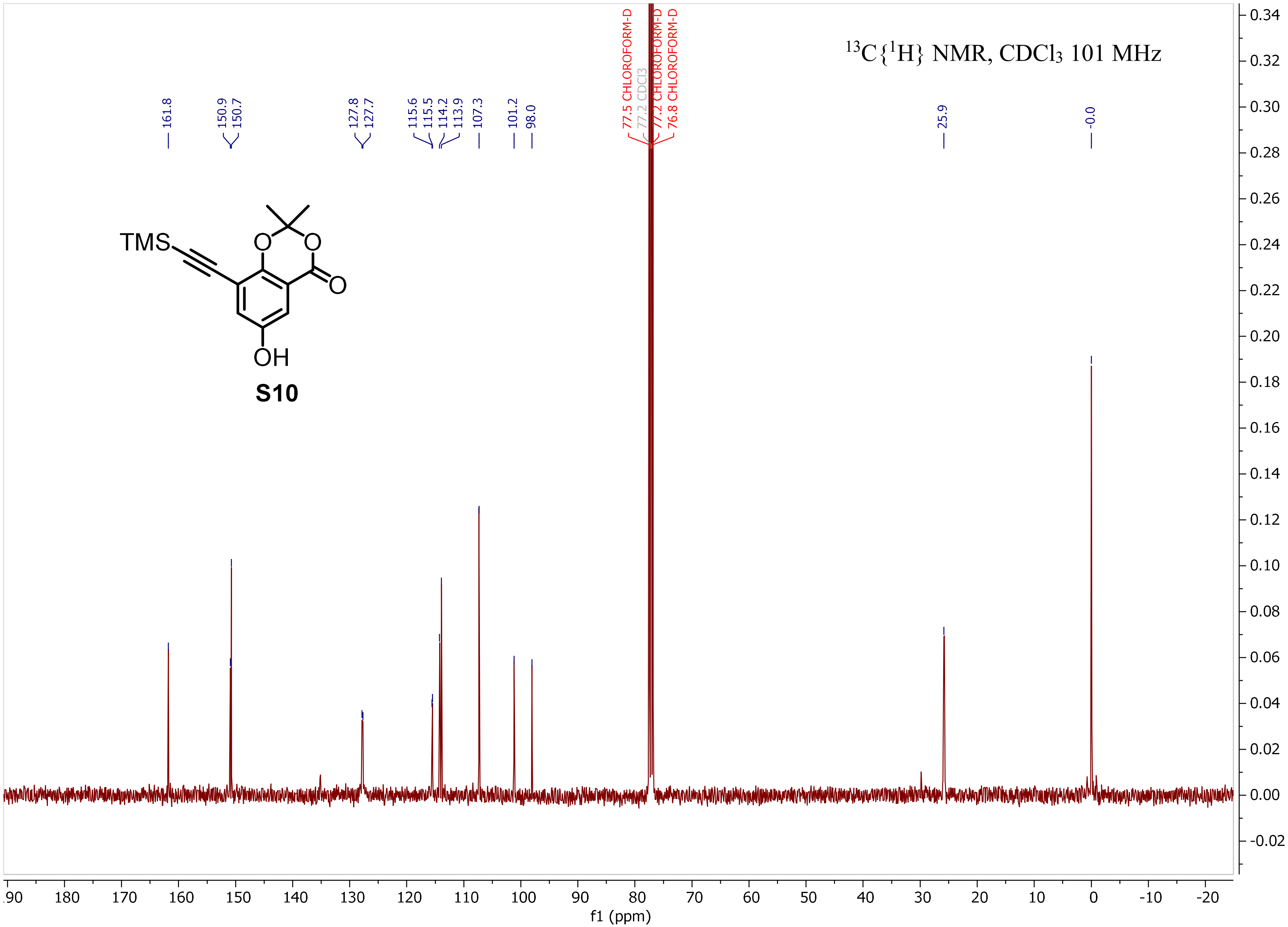

$^{13}C\{^1H\}$ NMR, $CDCl_3$ 101 MHz
161.8
150.9
150.7
127.8
127.7
115.6
115.5
114.2
113.9
107.3
101.2
98.0
77.5 CHLOROFORM-D
77.2 CDCl3
77.2 CHLOROFORM-D
76.8 CHLOROFORM-D
25.9
-0.0
TMS
O
O
O
OH
S10
f1 (ppm)

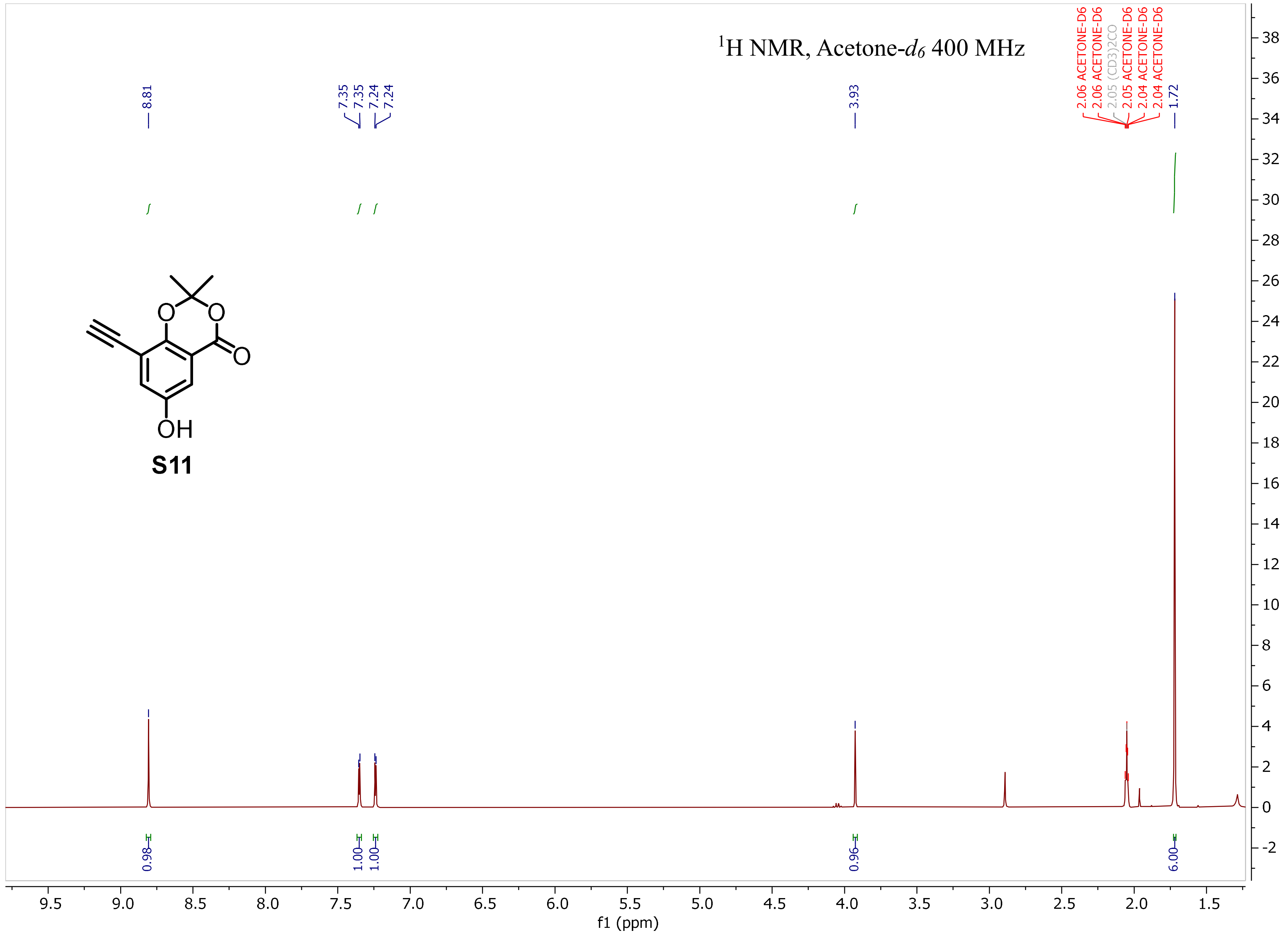

1H NMR, Acetone-d6 400 MHz
8.81
7.35
7.35
7.24
7.24
3.93
2.06 ACETONE-D6
2.06 ACETONE-D6
2.05 (CD3)2CO
2.05 ACETONE-D6
2.04 ACETONE-D6
2.04 ACETONE-D6
1.72
OH
O
O
O
S11
0.98
1.00
1.00
0.96
6.00
f1 (ppm)

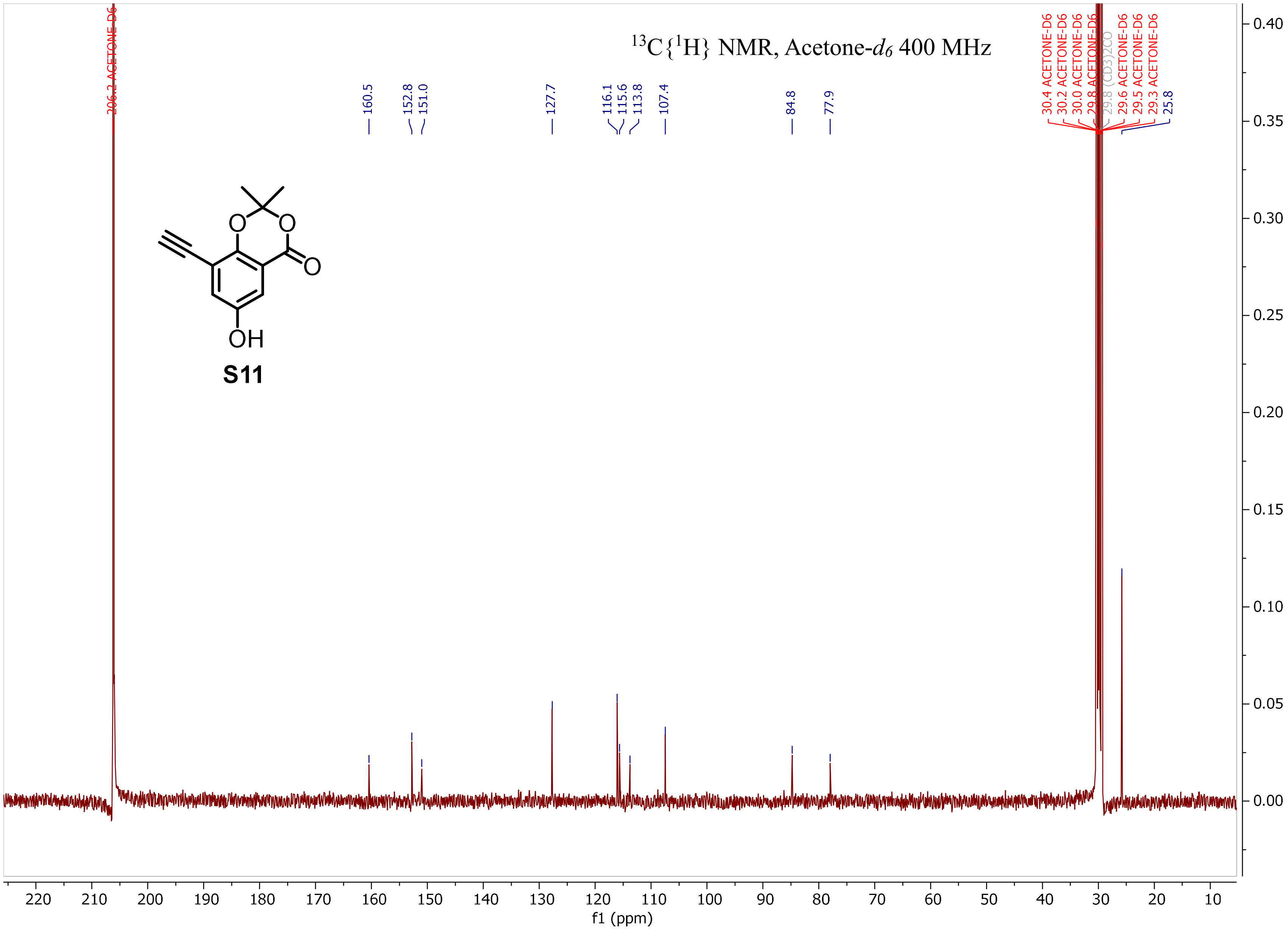

S11
$^{13}C\{^{1}H\}$ NMR, Acetone-d_6 400 MHz
206.2 ACETONE-D6
160.5
152.8
151.0
127.7
116.1
115.6
113.8
107.4
84.8
77.9
30.4 ACETONE-D6
30.2 ACETONE-D6
30.0 ACETONE-D6
29.8 ACETONE-D6
29.8 (CD3)2CO
29.6 ACETONE-D6
29.5 ACETONE-D6
29.3 ACETONE-D6
25.8
f1 (ppm)

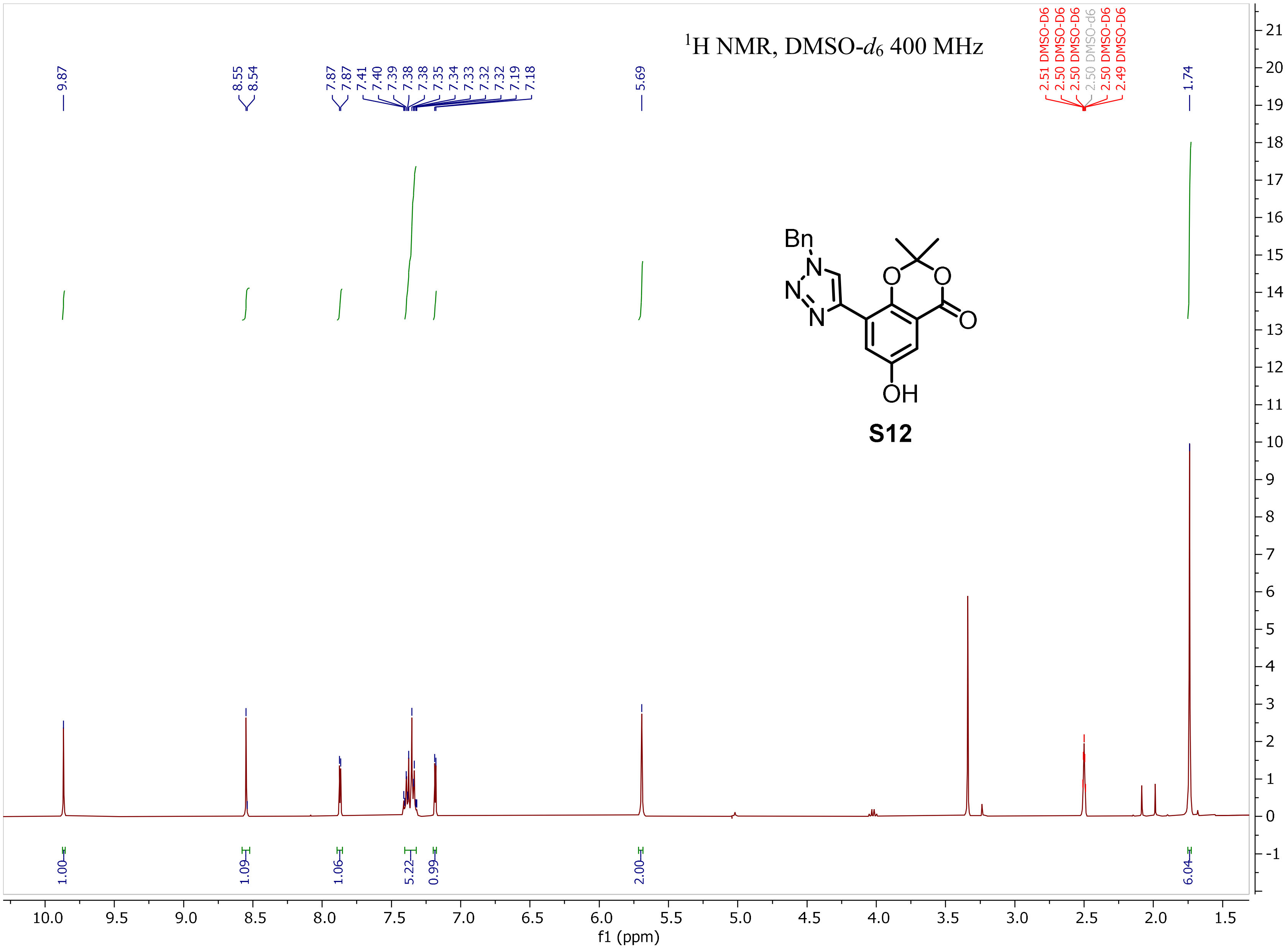

¹H NMR, DMSO-d_6 400 MHz
9.87
8.55
8.54
7.87
7.87
7.41
7.40
7.39
7.38
7.38
7.35
7.34
7.33
7.32
7.32
7.19
7.18
5.69
2.51 DMSO-D6
2.50 DMSO-D6
2.50 DMSO-D6
2.50 DMSO-d6
2.50 DMSO-D6
2.49 DMSO-D6
1.74
Bn
N
N
N
O
O
O
OH
S12
1.00
1.09
1.06
5.22
0.99
2.00
6.04
f1 (ppm)

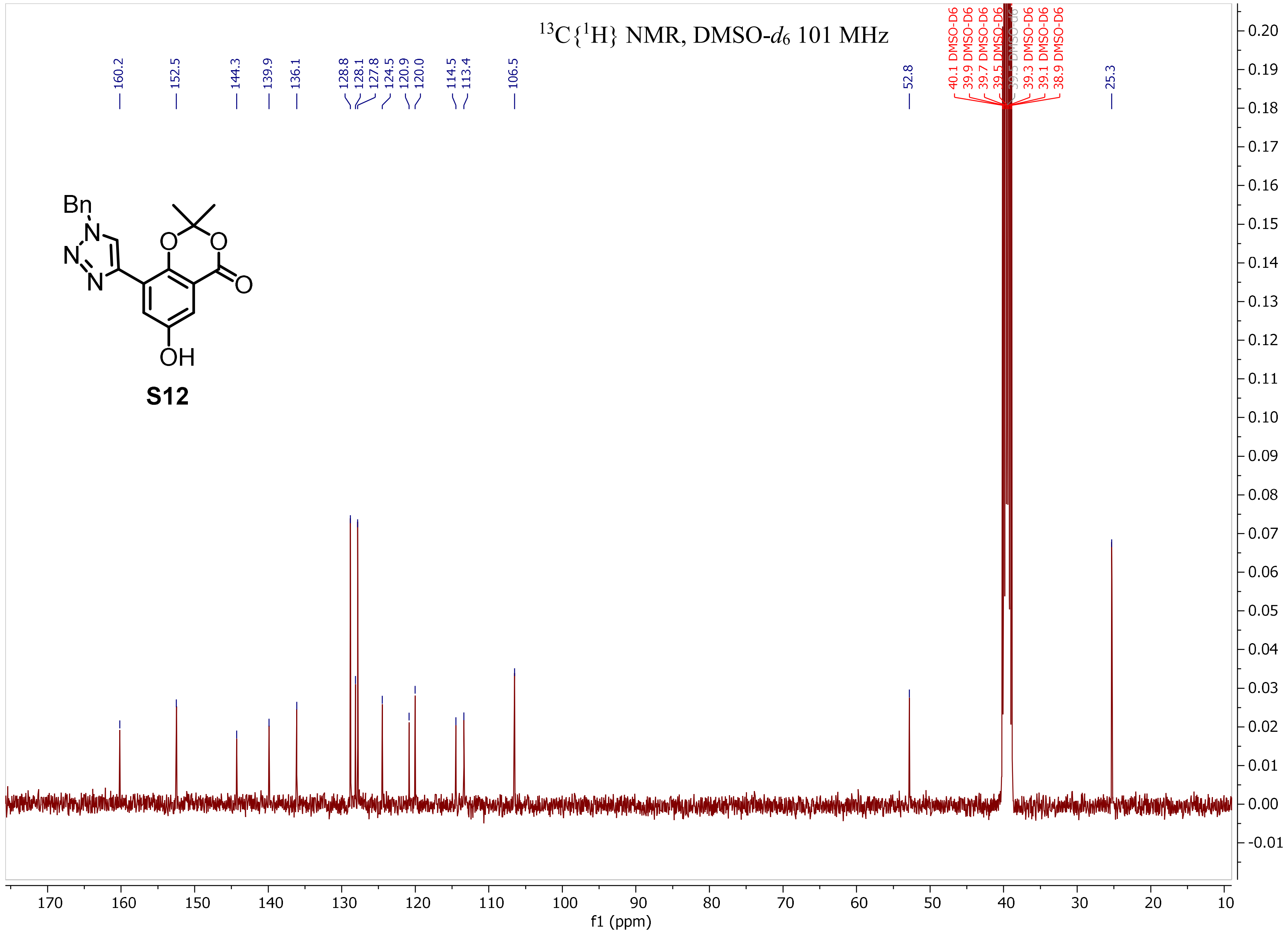
$^{13}C\{^1H\}$ NMR, DMSO-d_6 101 MHz
160.2
152.5
144.3
139.9
136.1
128.8
128.1
127.8
124.5
120.9
120.0
114.5
113.4
106.5
52.8
40.1 DMSO-D6
39.9 DMSO-D6
39.7 DMSO-D6
39.5 DMSO-D6
39.3 DMSO-D6
39.1 DMSO-D6
38.9 DMSO-D6
25.3
Bn
N
N
N
O
O
O
OH
S12
f1 (ppm)

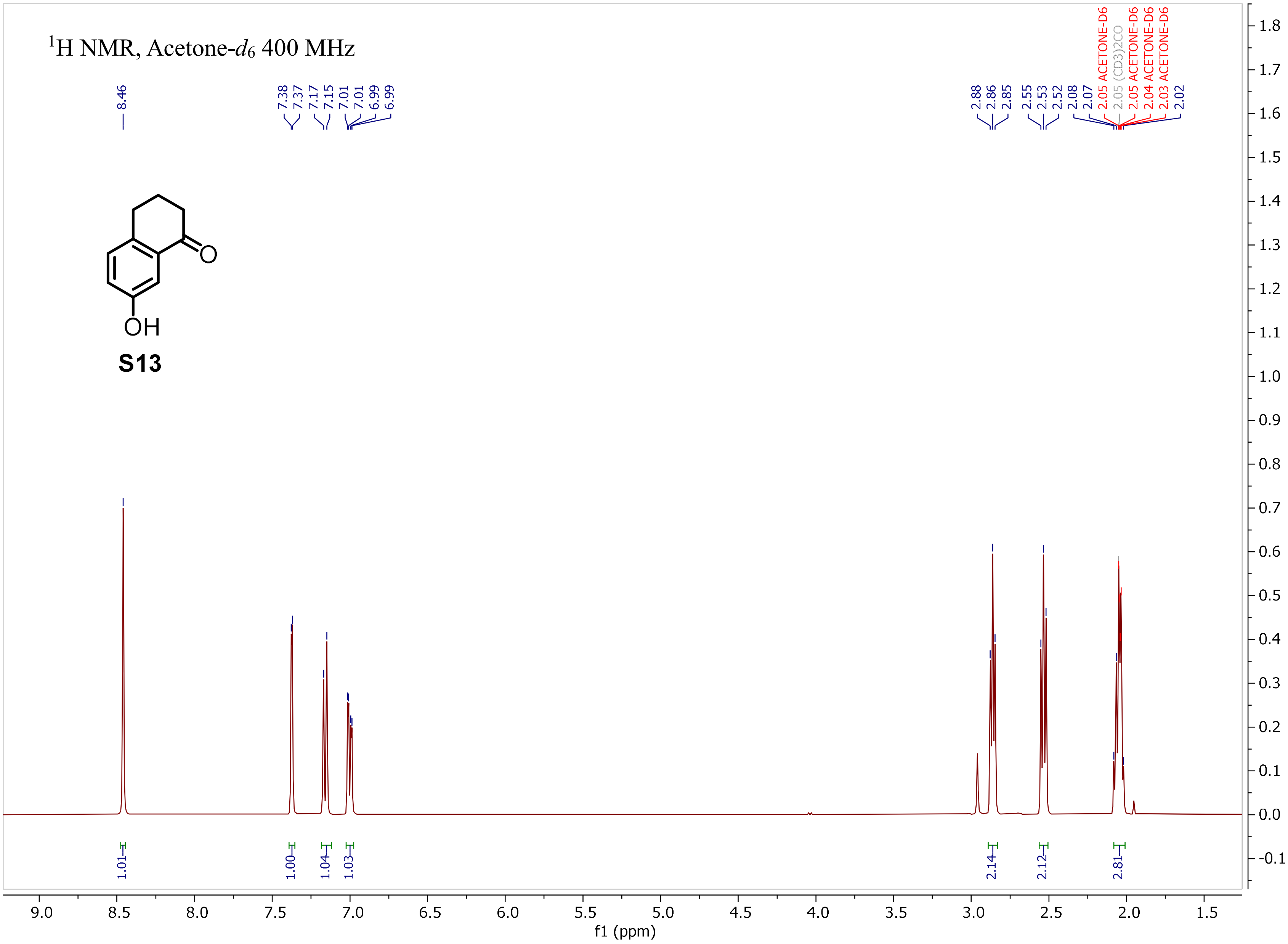

^{1}H NMR, Acetone-d_6 400 MHz
8.46
7.38
7.37
7.17
7.15
7.01
7.01
6.99
6.99
2.88
2.86
2.85
2.55
2.53
2.52
2.08
2.07
2.05 ACETONE-D6
2.05 (CD3)2CO
2.05 ACETONE-D6
2.04 ACETONE-D6
2.03 ACETONE-D6
2.02
OH
S13
1.01
1.00
1.04
1.03
2.14
2.12
2.81
f1 (ppm)

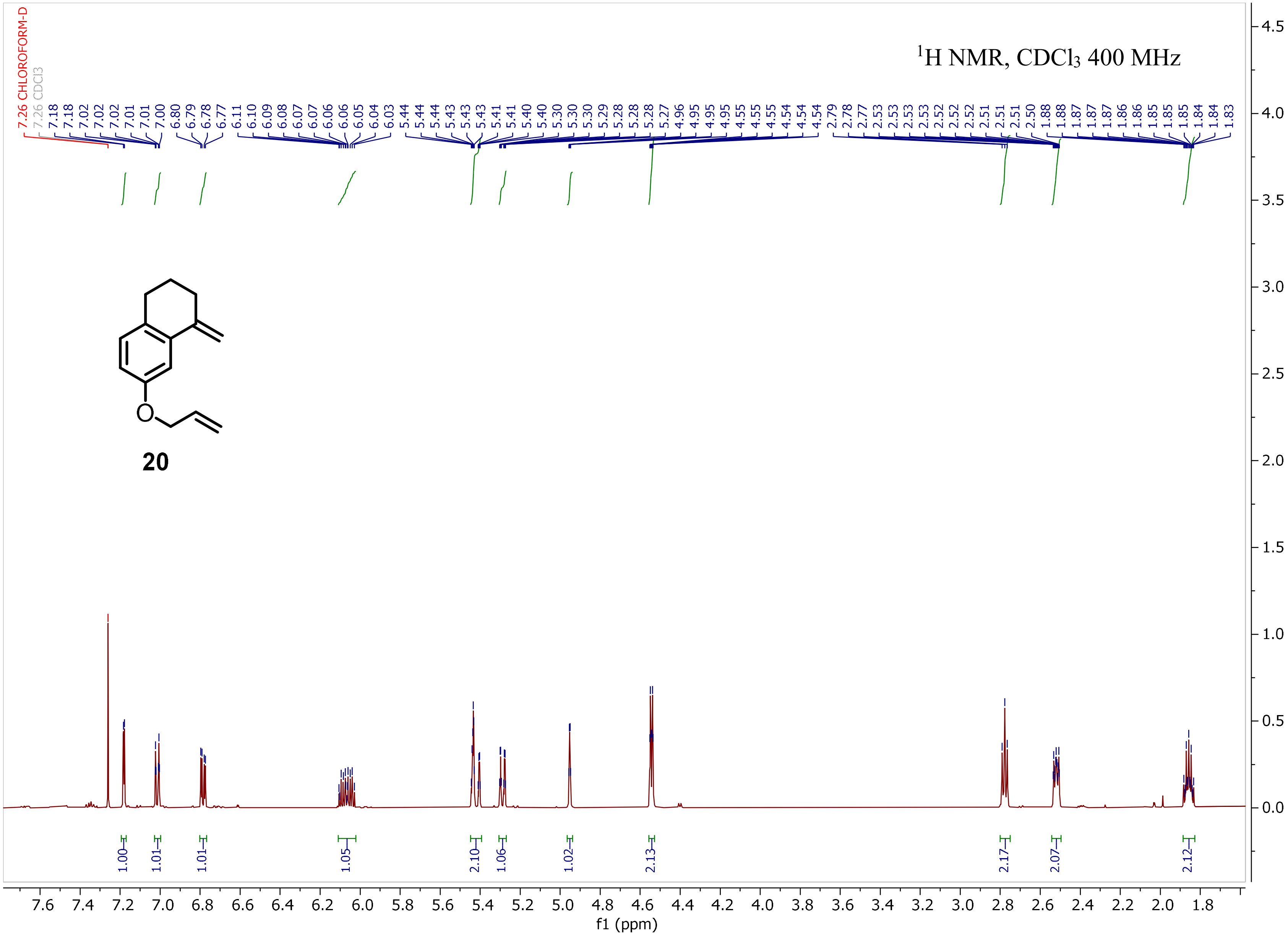
1H NMR, CDCl3 400 MHz
7.26 CHLOROFORM-D
7.26 CDCl3
7.18 7.18 7.02 7.02 7.02 7.01 7.01 7.00 6.80 6.79 6.78 6.77 6.11 6.10 6.09 6.08 6.07 6.06 6.06 6.05 6.04 6.03 5.44 5.44 5.43 5.43 5.43 5.41 5.40 5.40 5.30 5.30 5.29 5.28 5.28 5.28 5.27 4.96 4.95 4.95 4.95 4.55 4.55 4.55 4.54 4.54 4.54 2.79 2.78 2.77 2.53 2.53 2.53 2.53 2.52 2.52 2.52 2.51 2.51 2.51 2.50 1.88 1.88 1.87 1.87 1.87 1.86 1.86 1.85 1.85 1.85 1.84 1.84 1.83
20
1.00 1.01 1.01 1.05 2.10 1.06 1.02 2.13 2.17 2.07 2.12
f1 (ppm)

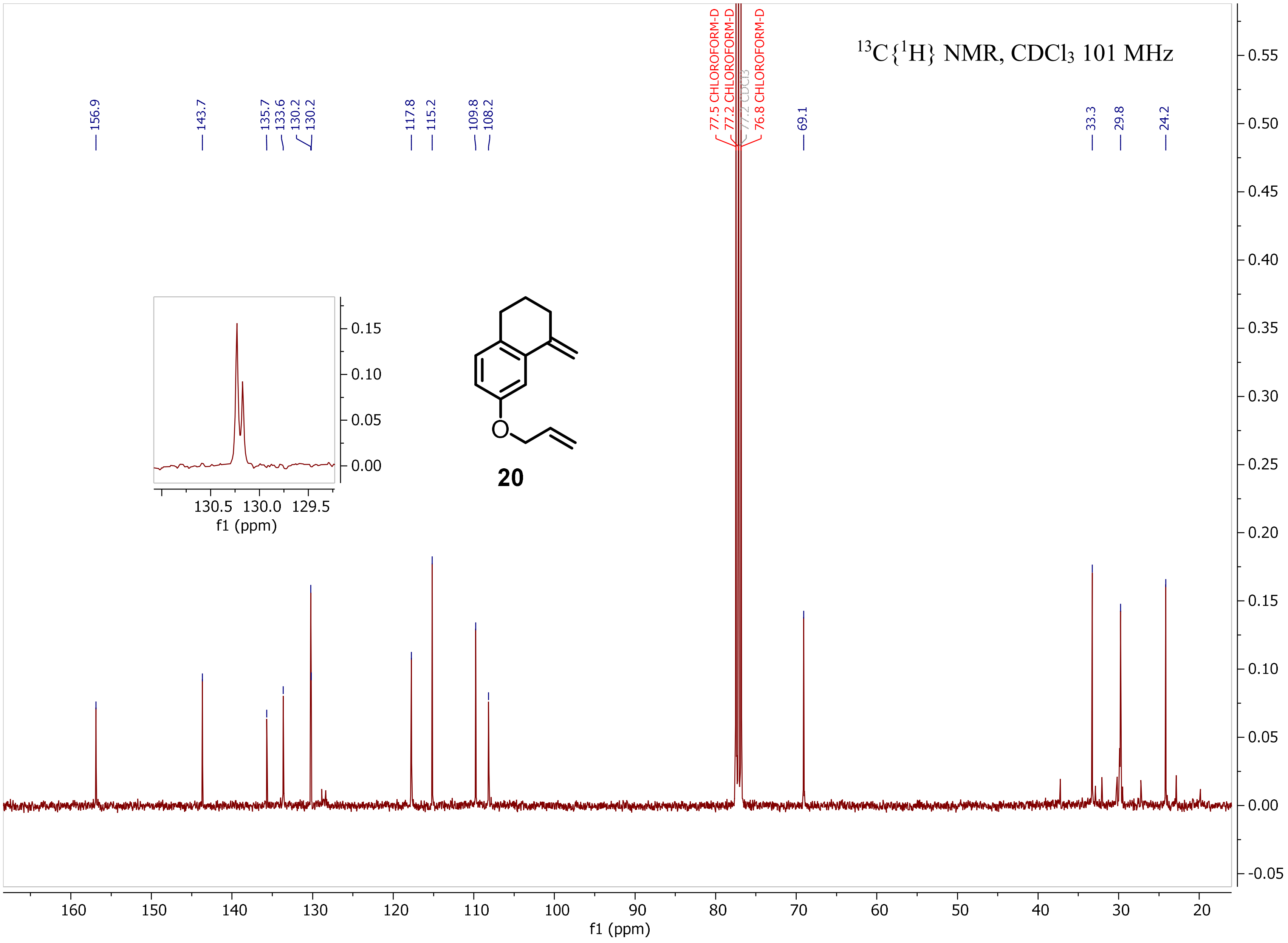

$^{13}C\{^1H\}$ NMR, $CDCl_3$ 101 MHz
156.9
143.7
135.7
133.6
130.2
130.2
117.8
115.2
109.8
108.2
77.5 CHLOROFORM-D
77.2 CHLOROFORM-D
77.2 CDCl3
76.8 CHLOROFORM-D
69.1
33.3
29.8
24.2
130.5 130.0 129.5
f1 (ppm)
20
f1 (ppm)

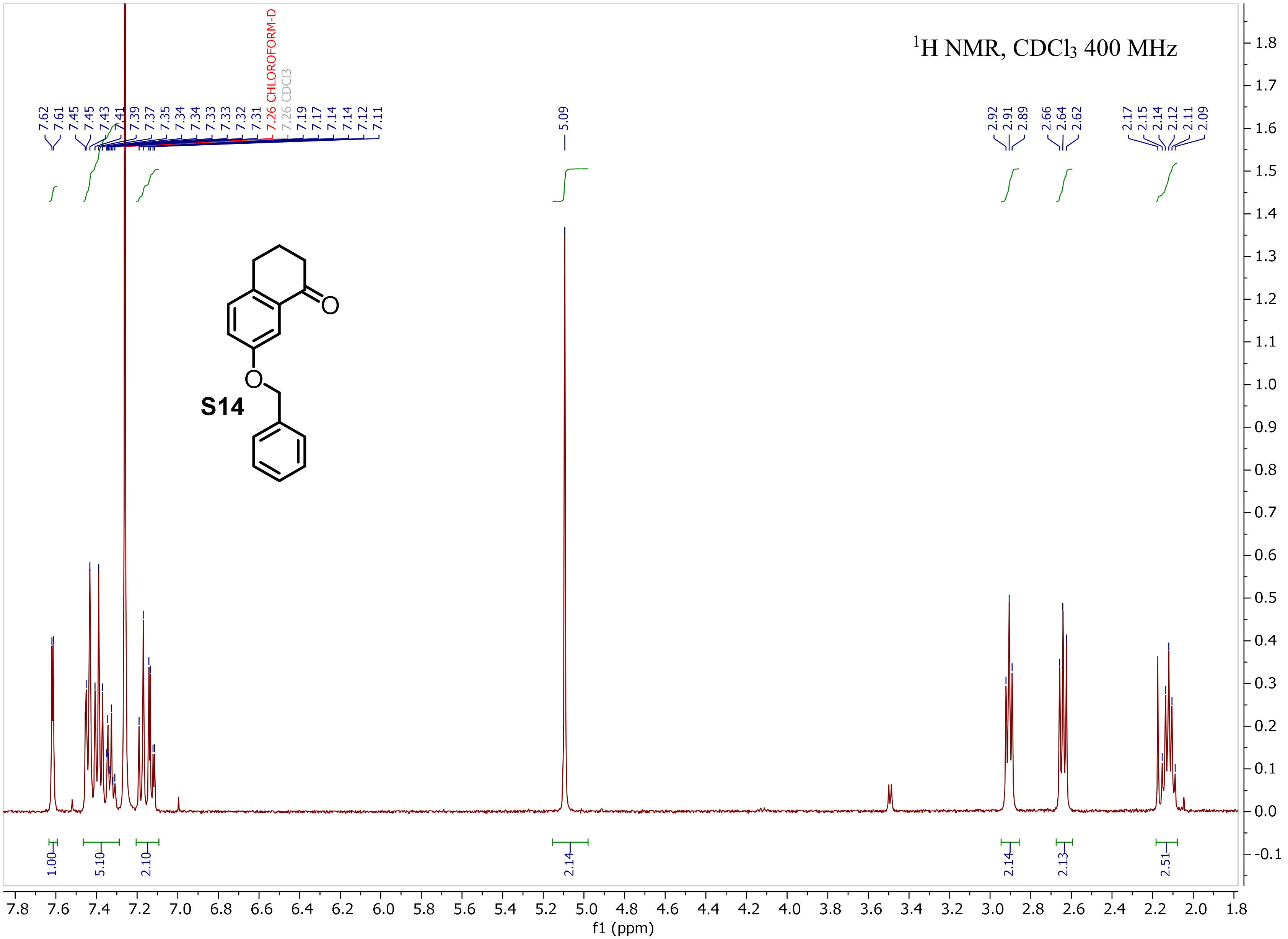

1H NMR, CDCl3 400 MHz
S14
7.62
7.61
7.45
7.45
7.43
7.41
7.39
7.37
7.35
7.34
7.34
7.33
7.33
7.32
7.31
7.26 CHLOROFORM-D
7.26 CDCl3
7.19
7.17
7.14
7.14
7.12
7.11
5.09
2.92
2.91
2.89
2.66
2.64
2.62
2.17
2.15
2.14
2.12
2.11
2.09
1.00
5.10
2.10
2.14
2.14
2.13
2.51
f1 (ppm)

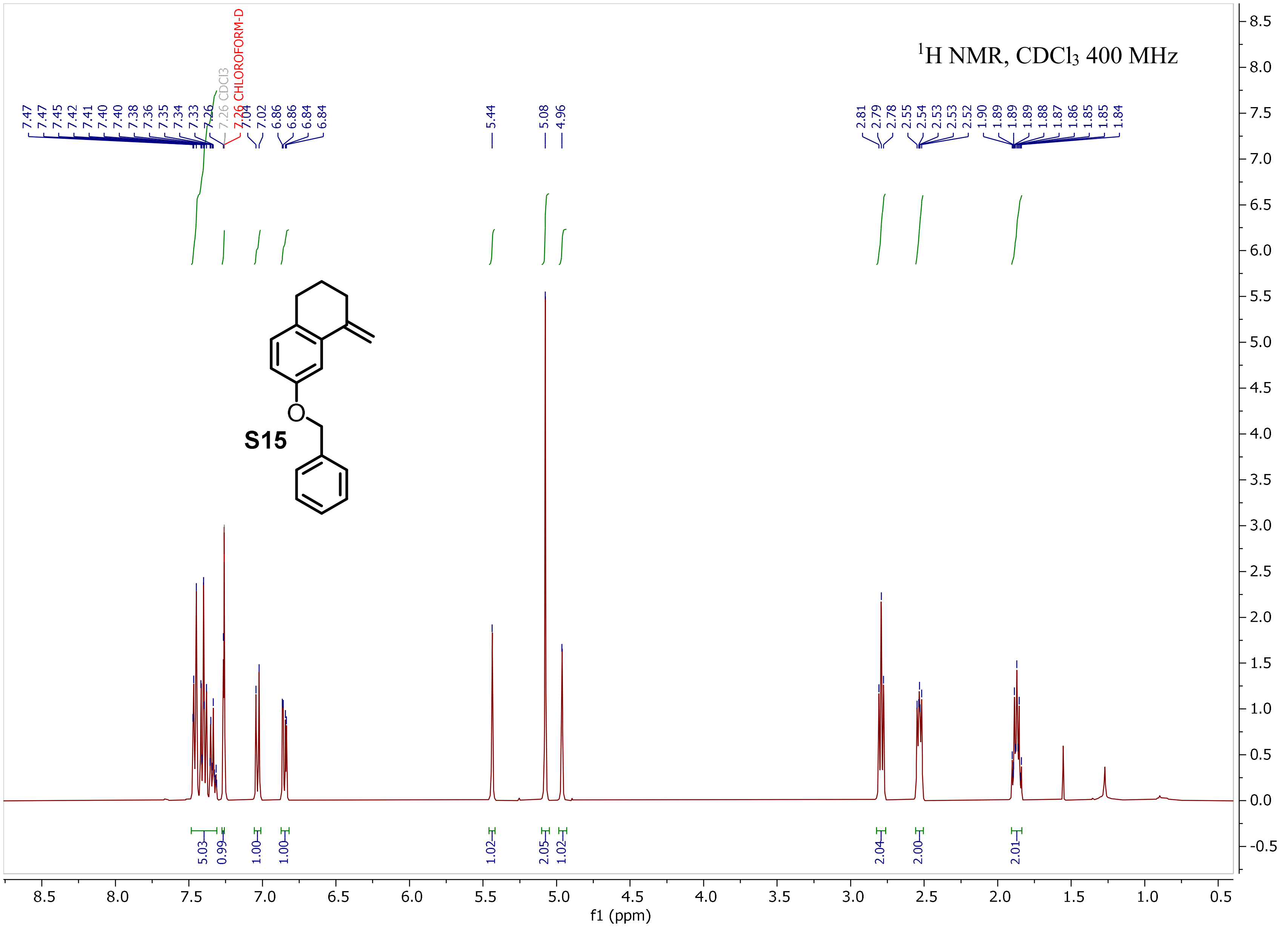

1H NMR, CDCl3 400 MHz
S15
7.47
7.47
7.45
7.42
7.41
7.40
7.40
7.38
7.36
7.35
7.34
7.33
7.26
7.26 CDCl3
7.26 CHLOROFORM-D
7.04
7.02
6.86
6.86
6.84
6.84
5.44
5.08
4.96
2.81
2.79
2.78
2.55
2.54
2.53
2.53
2.52
1.90
1.89
1.89
1.89
1.88
1.87
1.86
1.85
1.85
1.84
5.03
0.99
1.00
1.00
1.02
2.05
1.02
2.04
2.00
2.01
f1 (ppm)

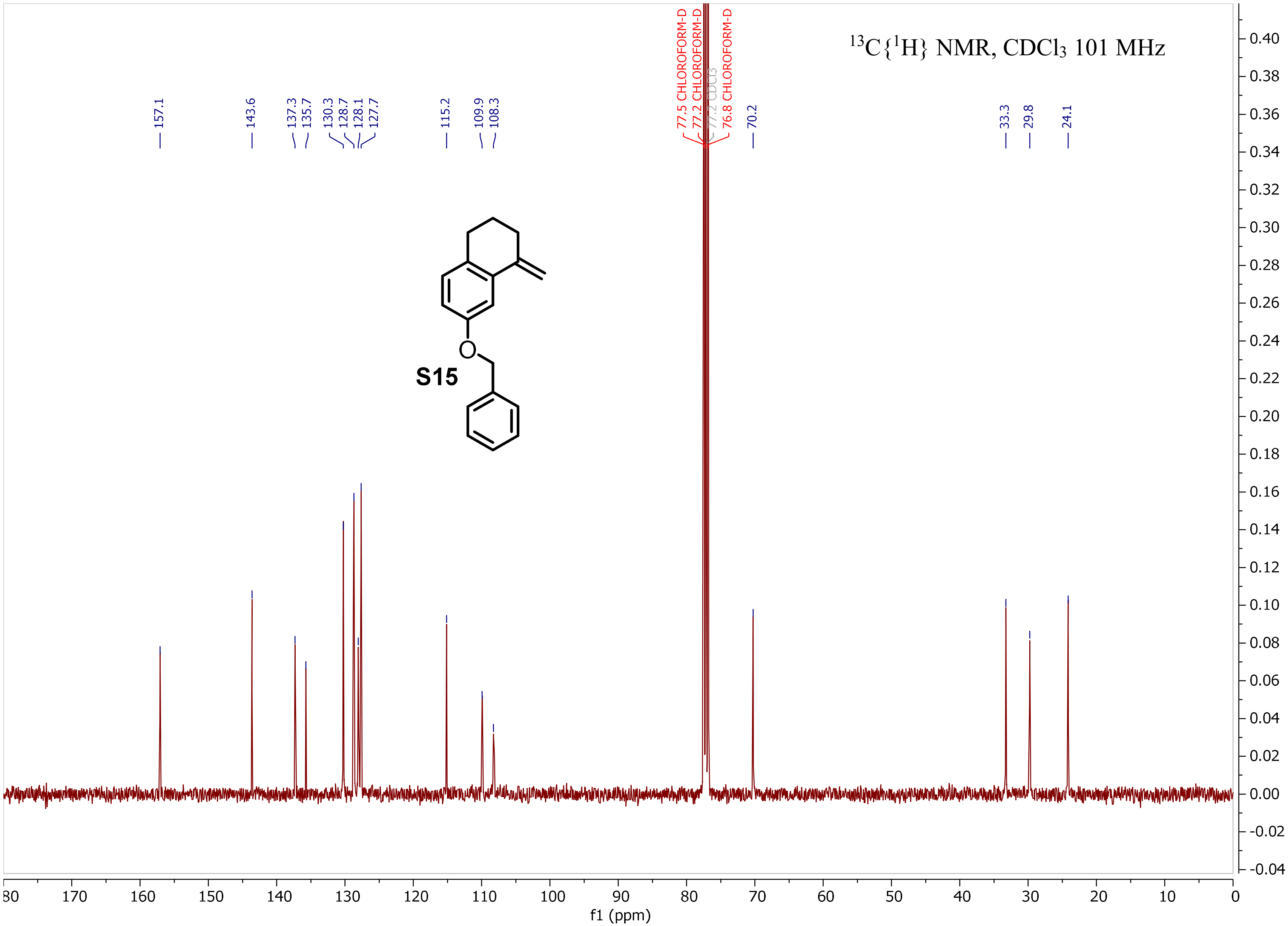
$^{13}C\{^1H\}$ NMR, $CDCl_3$ 101 MHz
157.1
143.6
137.3
135.7
130.3
128.7
128.1
127.7
115.2
109.9
108.3
77.5 CHLOROFORM-D
77.2 CHLOROFORM-D
77.2 CDCl3
76.8 CHLOROFORM-D
70.2
33.3
29.8
24.1
S15
f1 (ppm)

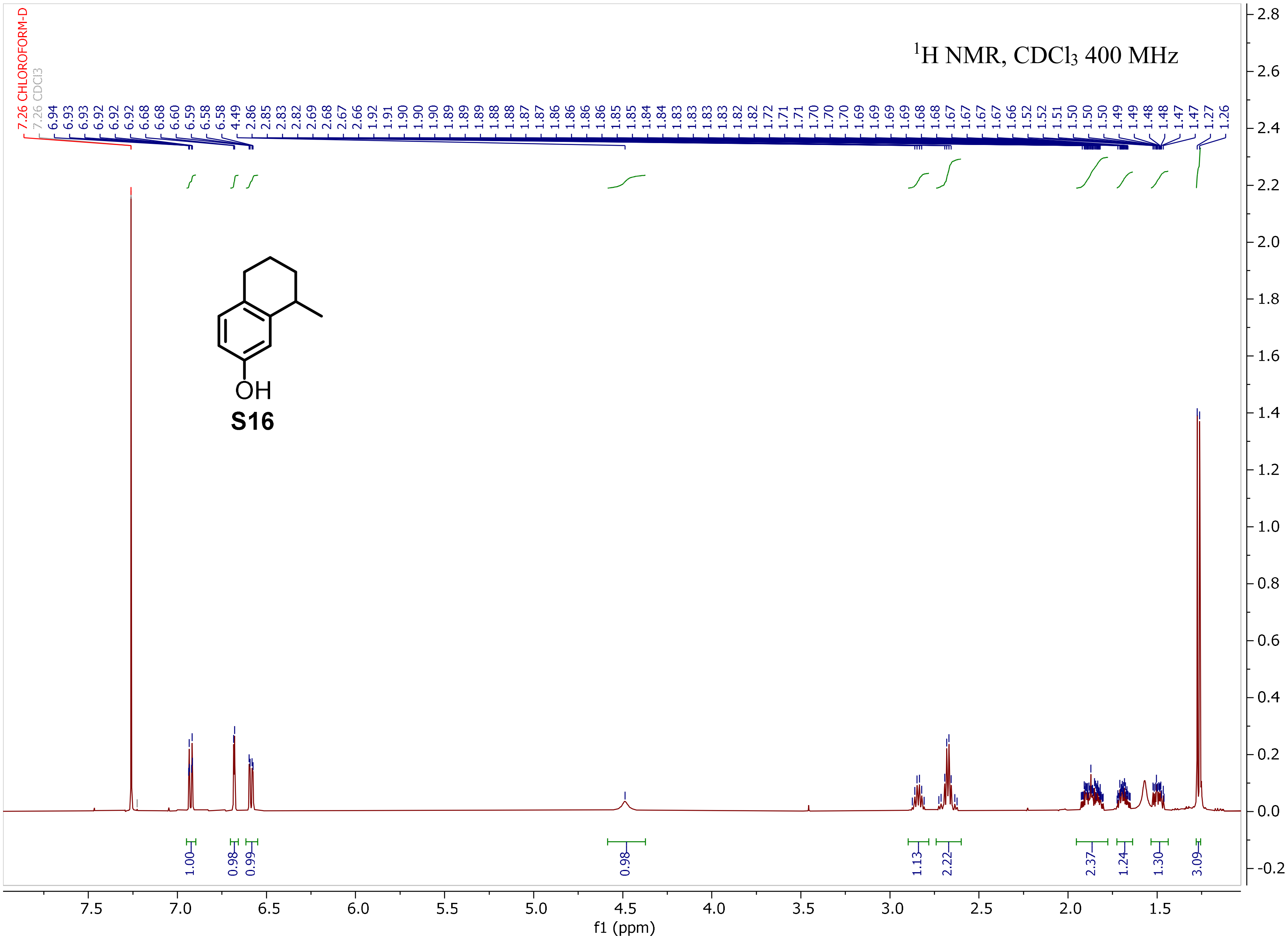

1H NMR, CDCl3 400 MHz
7.26 CHLOROFORM-D
7.26 CDCl3
6.94 6.93 6.93 6.92 6.92 6.92 6.68 6.68 6.60 6.59 6.58 6.58 4.49 2.86 2.85 2.83 2.82 2.69 2.68 2.67 2.66 1.92 1.91 1.90 1.90 1.90 1.89 1.89 1.89 1.88 1.88 1.87 1.87 1.86 1.86 1.86 1.86 1.85 1.85 1.84 1.84 1.83 1.83 1.83 1.83 1.82 1.82 1.72 1.71 1.71 1.70 1.70 1.70 1.69 1.69 1.69 1.69 1.68 1.68 1.67 1.67 1.67 1.67 1.66 1.52 1.52 1.51 1.50 1.50 1.50 1.49 1.49 1.48 1.48 1.47 1.47 1.27 1.26
OH
S16
1.00 0.98 0.99 0.98 1.13 2.22 2.37 1.24 1.30 3.09
f1 (ppm)

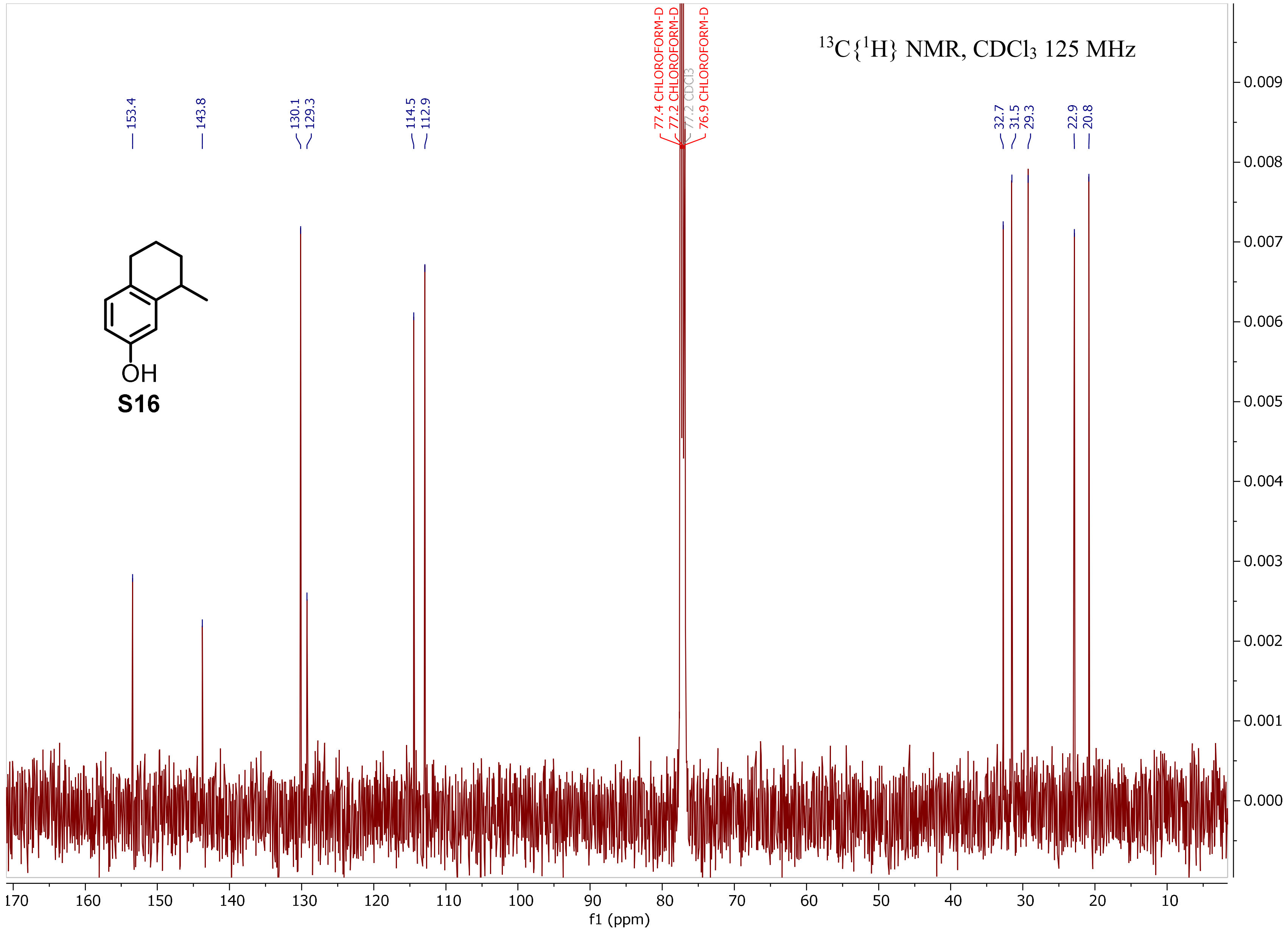

13C{1H} NMR, CDCl3 125 MHz
153.4
143.8
130.1
129.3
114.5
112.9
77.4 CHLOROFORM-D
77.2 CHLOROFORM-D
77.2 CDCl3
76.9 CHLOROFORM-D
32.7
31.5
29.3
22.9
20.8
OH
S16
f1 (ppm)

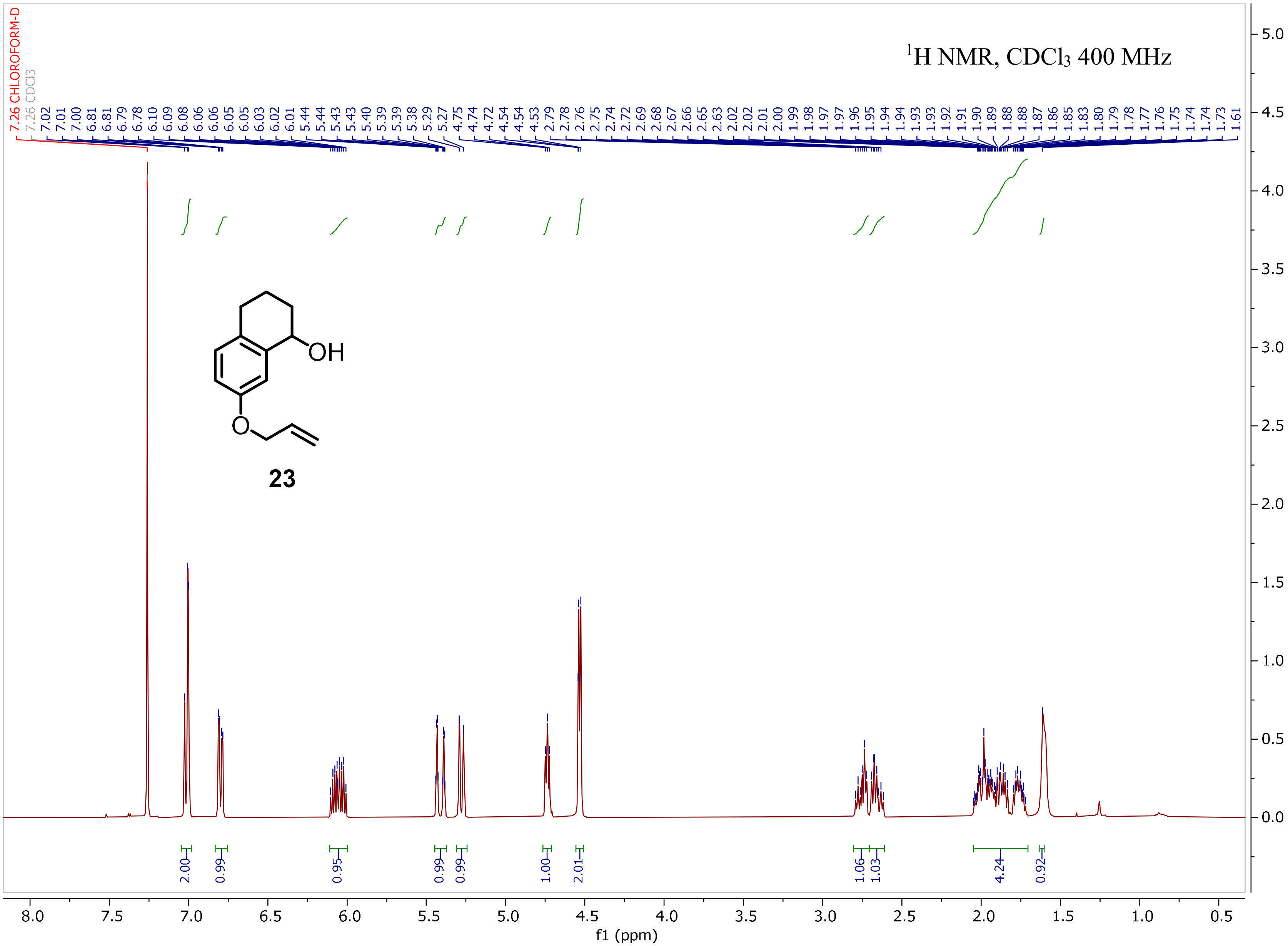

^{1}H NMR, CDCl$_3$ 400 MHz
OH
O
23
f1 (ppm)

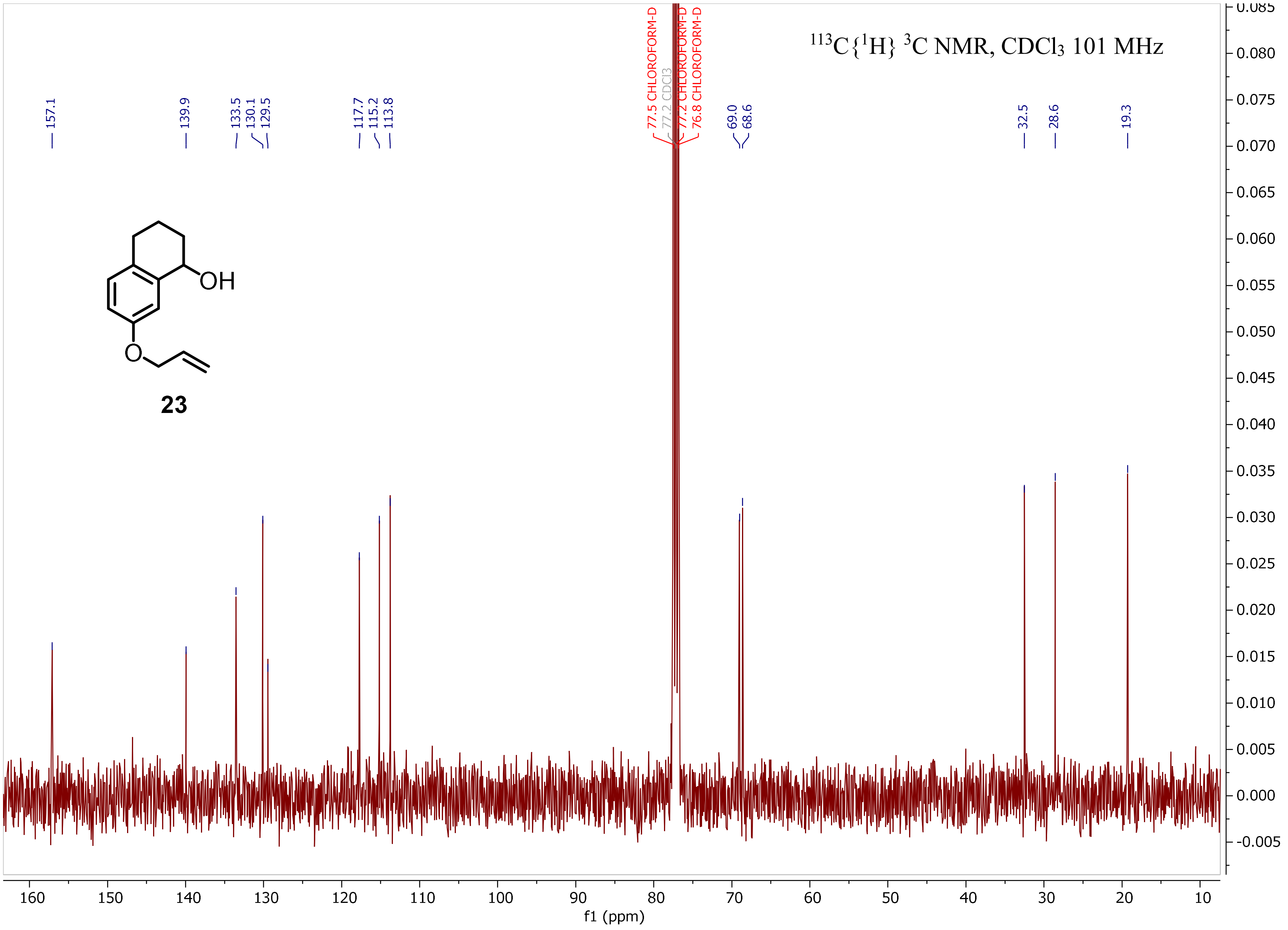
113C{1H} 3C NMR, CDCl3 101 MHz
157.1
139.9
133.5
130.1
129.5
117.7
115.2
113.8
77.5 CHLOROFORM-D
77.2 CDCl3
77.2 CHLOROFORM-D
76.8 CHLOROFORM-D
69.0
68.6
32.5
28.6
19.3
OH
O
23
f1 (ppm)

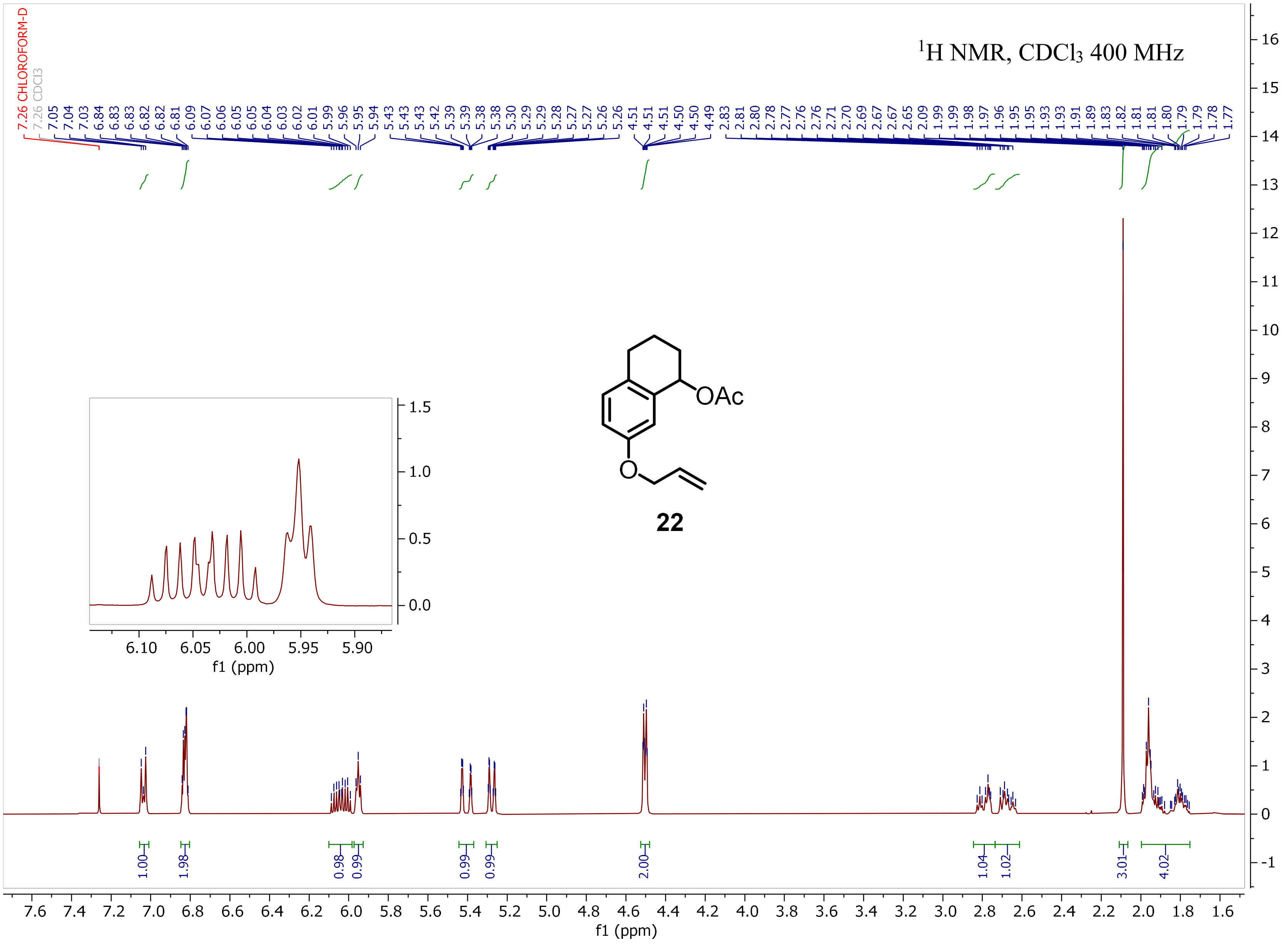

^{1}H NMR, CDCl$_3$ 400 MHz
7.26 CHLOROFORM-D
7.26 CDCl3
OAc
O
22
f1 (ppm)

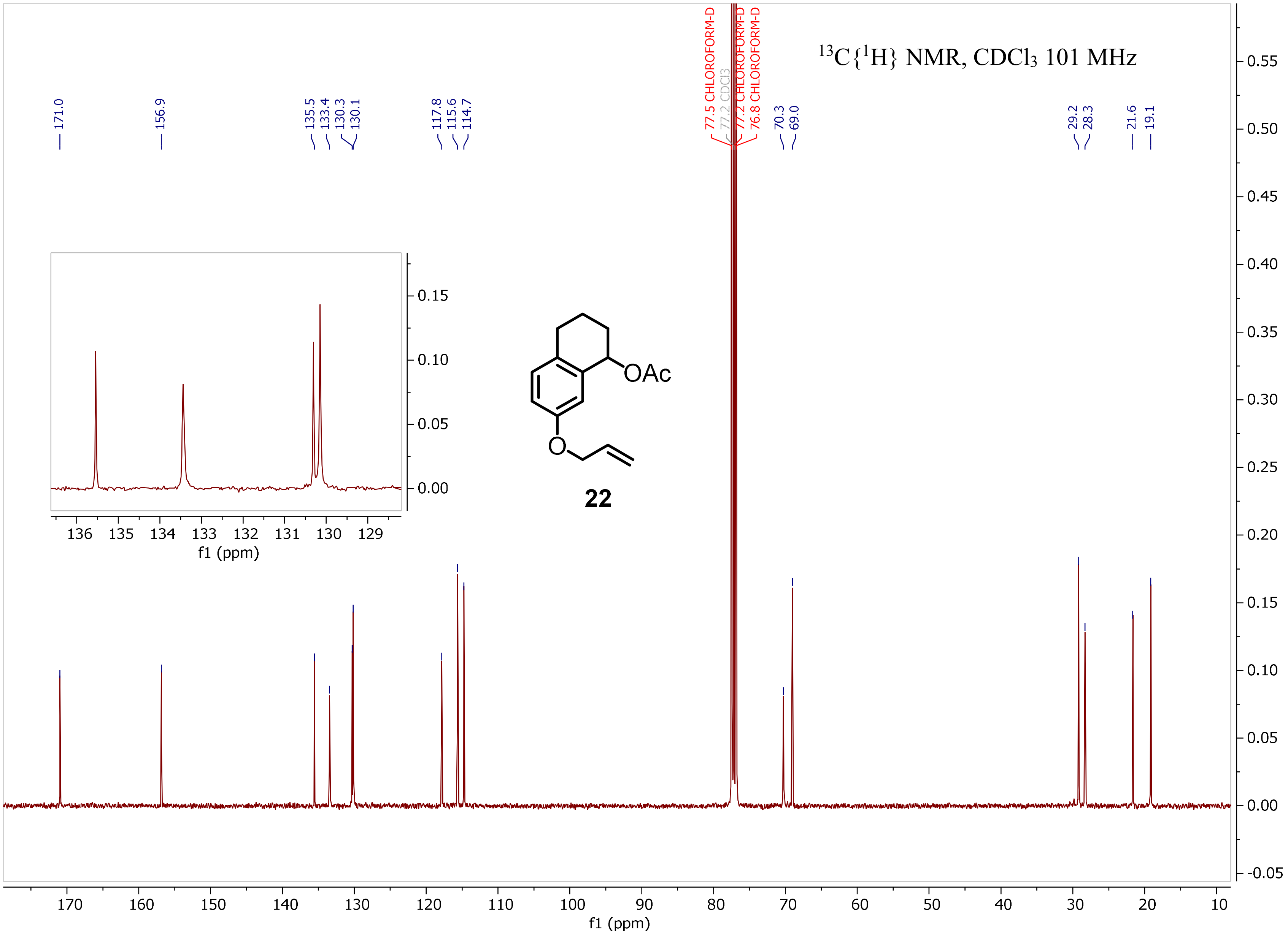

$^{13}C\{^{1}H\}$ NMR, $CDCl_3$ 101 MHz
171.0
156.9
135.5
133.4
130.3
130.1
117.8
115.6
114.7
77.5 CHLOROFORM-D
77.2 CDCl3
77.2 CHLOROFORM-D
76.8 CHLOROFORM-D
70.3
69.0
29.2
28.3
21.6
19.1
OAc
O
22
f1 (ppm)

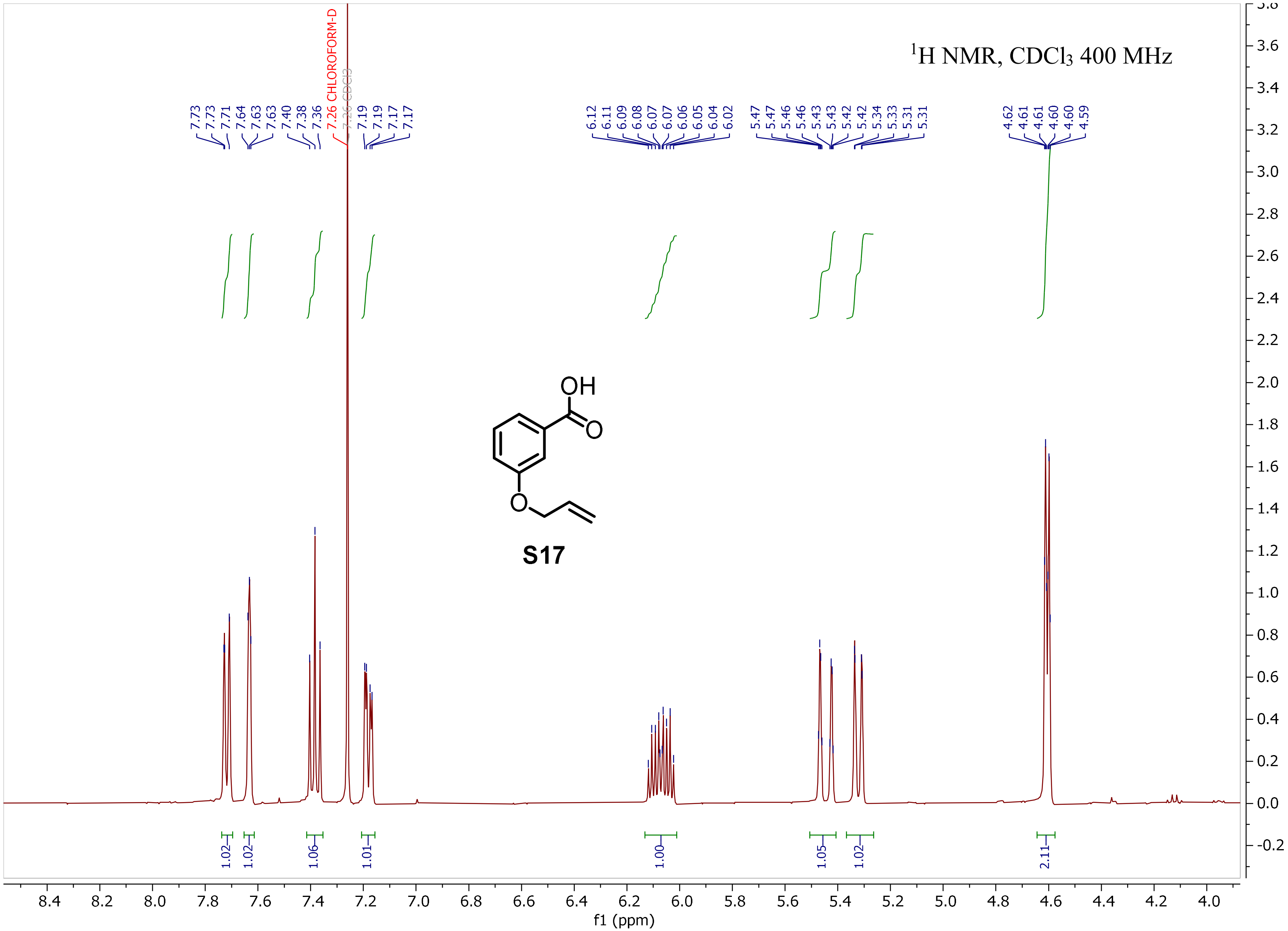

^{1}H NMR, CDCl$_3$ 400 MHz
S17
f1 (ppm)

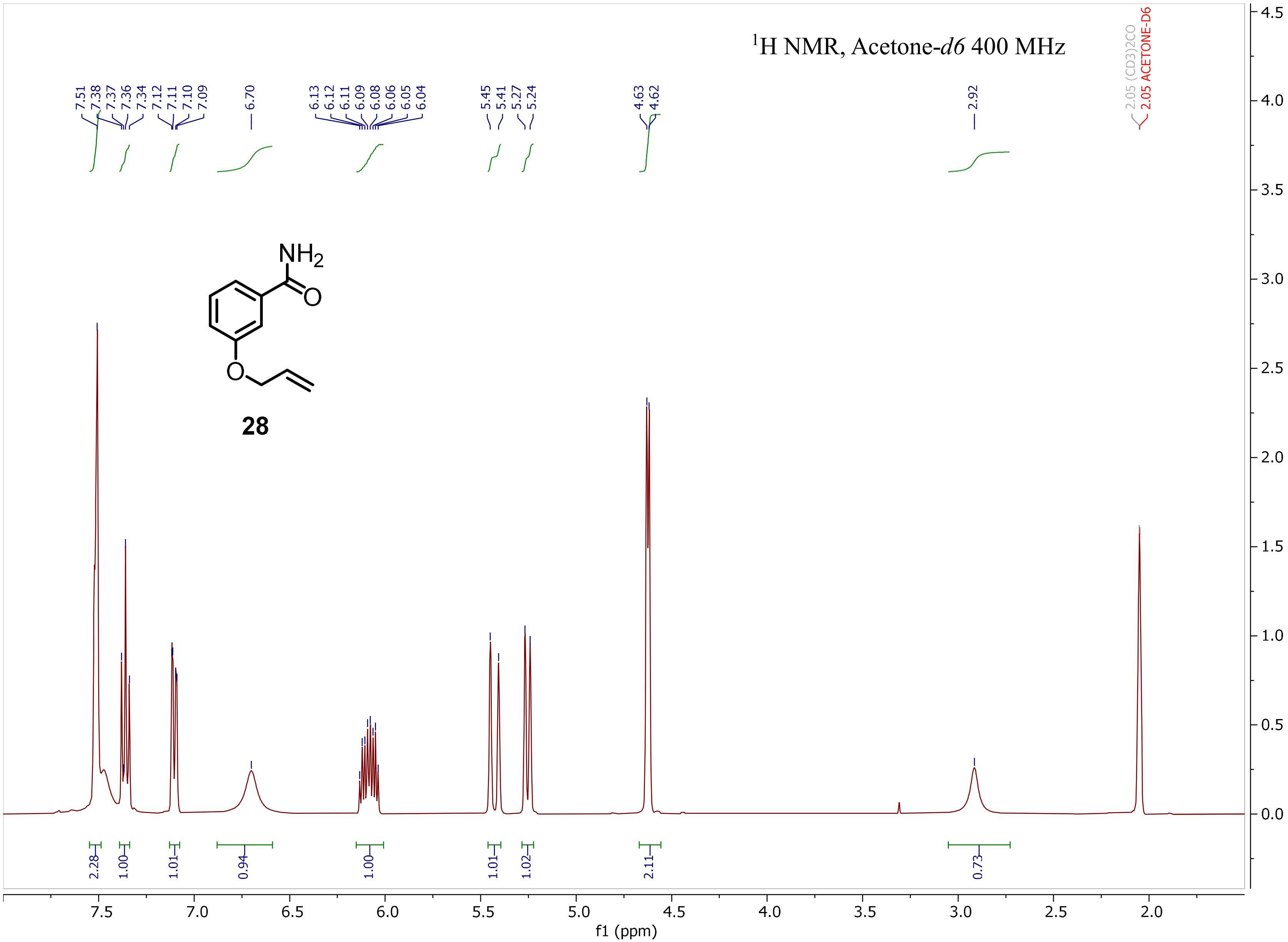

1H NMR, Acetone-d6 400 MHz
7.51
7.38
7.37
7.36
7.34
7.12
7.11
7.10
7.09
6.70
6.13
6.12
6.11
6.09
6.08
6.06
6.05
6.04
5.45
5.41
5.27
5.24
4.63
4.62
2.92
2.05 (CD3)2CO
2.05 ACETONE-D6
NH2
O
O
28
2.28
1.00
1.01
0.94
1.00
1.01
1.02
2.11
0.73
f1 (ppm)

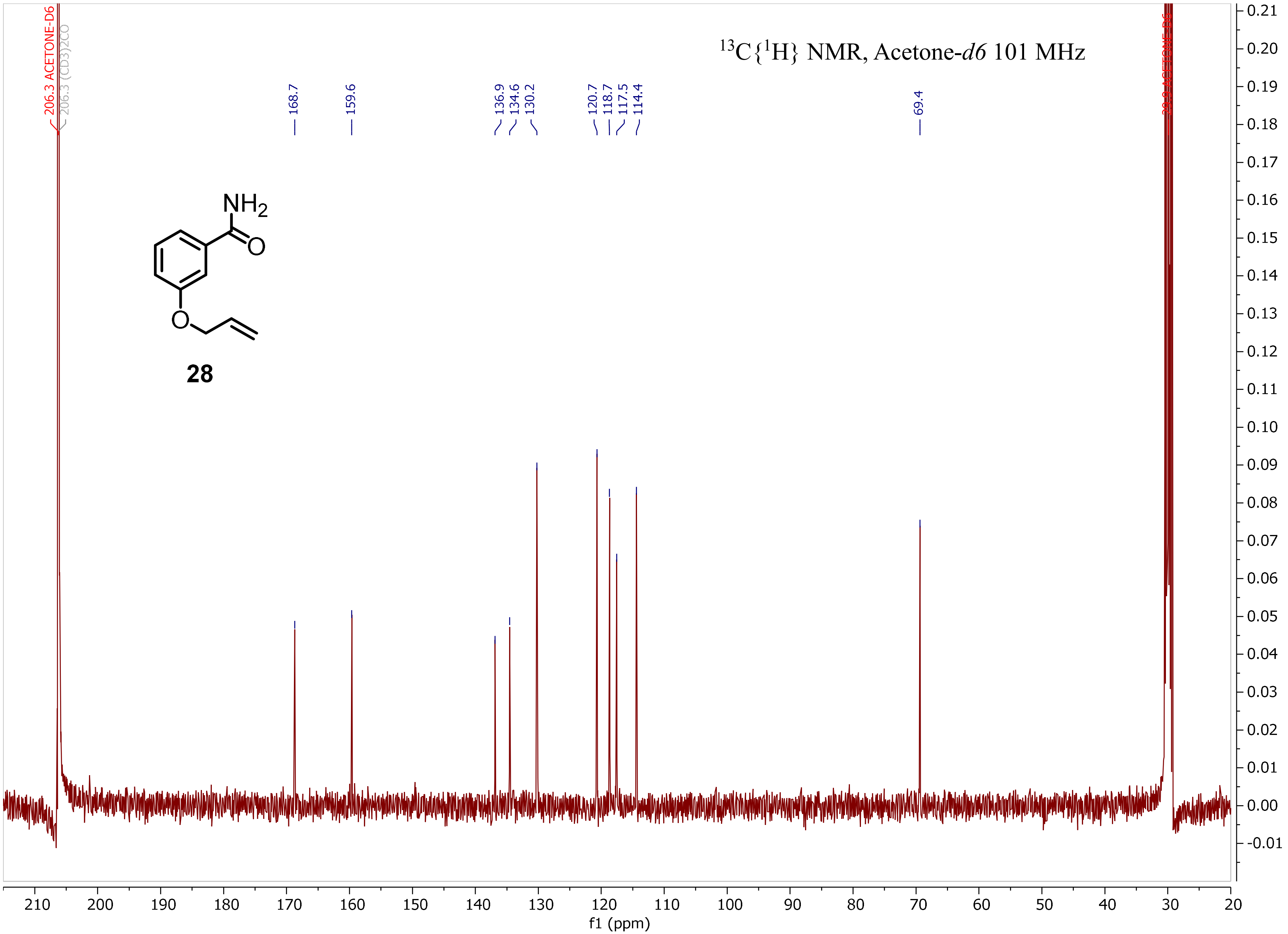

13C{1H} NMR, Acetone-d6 101 MHz
206.3 ACETONE-D6
206.3 (CD3)2CO
168.7
159.6
136.9
134.6
130.2
120.7
118.7
117.5
114.4
69.4
NH2
O
O
28
f1 (ppm)

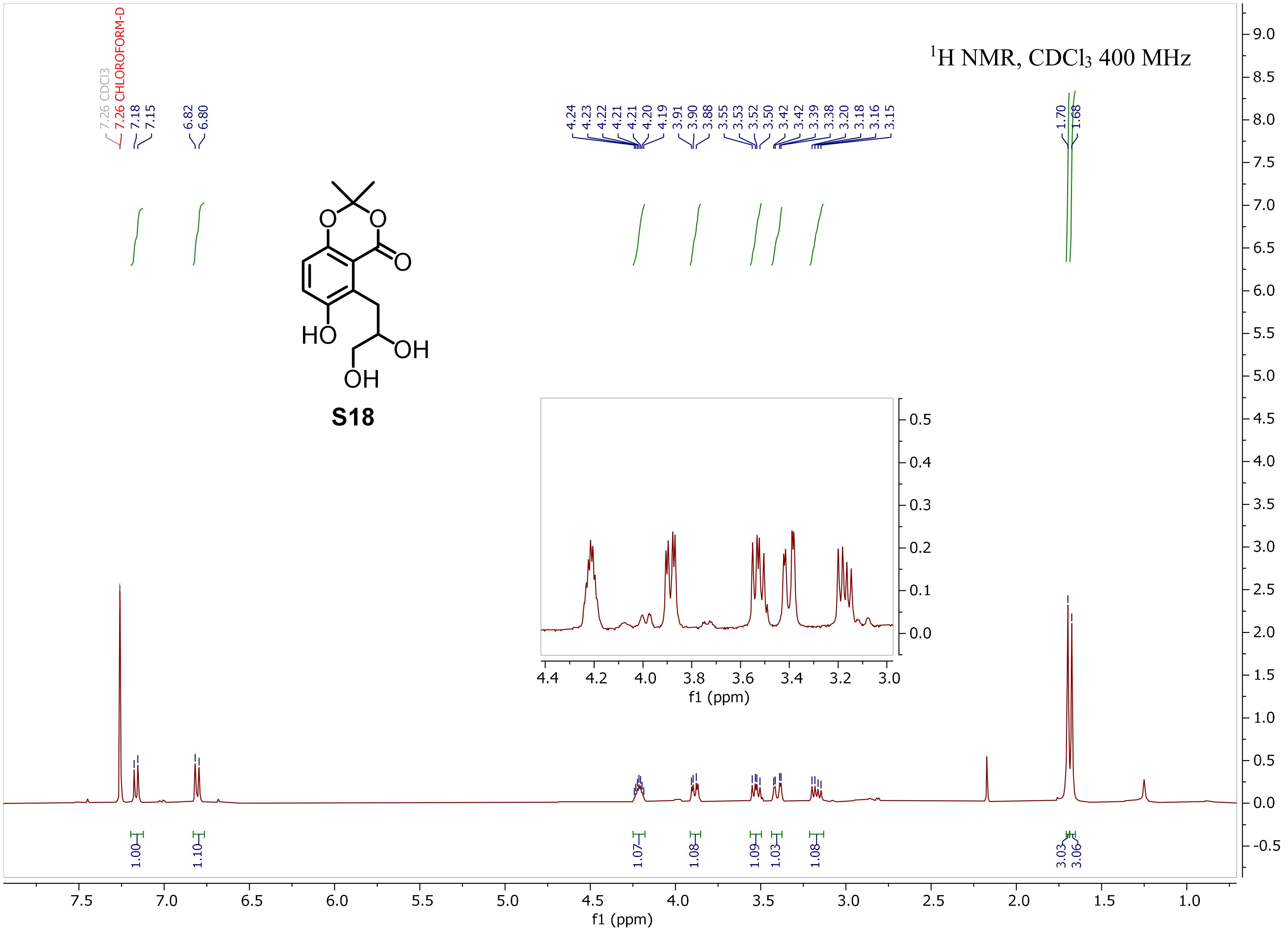

1H NMR, CDCl3 400 MHz
7.26 CDCl3
7.26 CHLOROFORM-D
7.18
7.15
6.82
6.80
4.24
4.23
4.22
4.21
4.21
4.20
4.19
3.91
3.90
3.88
3.55
3.53
3.52
3.50
3.42
3.42
3.39
3.38
3.20
3.18
3.16
3.15
1.70
1.68
HO
OH
OH
O
O
O
S18
1.00
1.10
1.07
1.08
1.09
1.03
1.08
3.03
3.06
f1 (ppm)

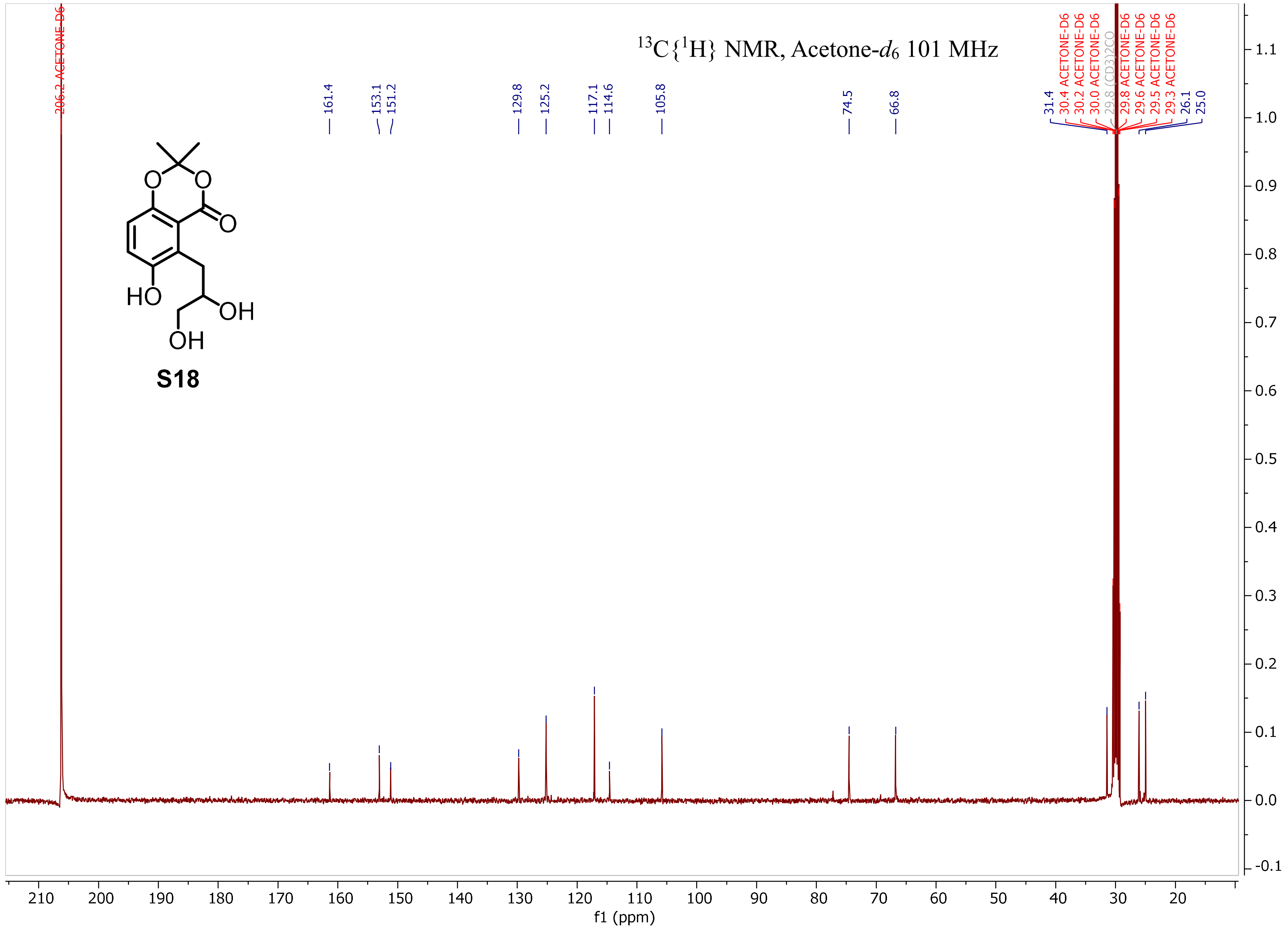

$^{13}C\{^1H\}$ NMR, Acetone-d_6 101 MHz
206.2 ACETONE-D6
161.4
153.1
151.2
129.8
125.2
117.1
114.6
105.8
74.5
66.8
31.4
30.4 ACETONE-D6
30.2 ACETONE-D6
30.0 ACETONE-D6
29.8 (CD3)2CO
29.8 ACETONE-D6
29.6 ACETONE-D6
29.5 ACETONE-D6
29.3 ACETONE-D6
26.1
25.0
HO
OH
OH
O
O
O
S18
f1 (ppm)

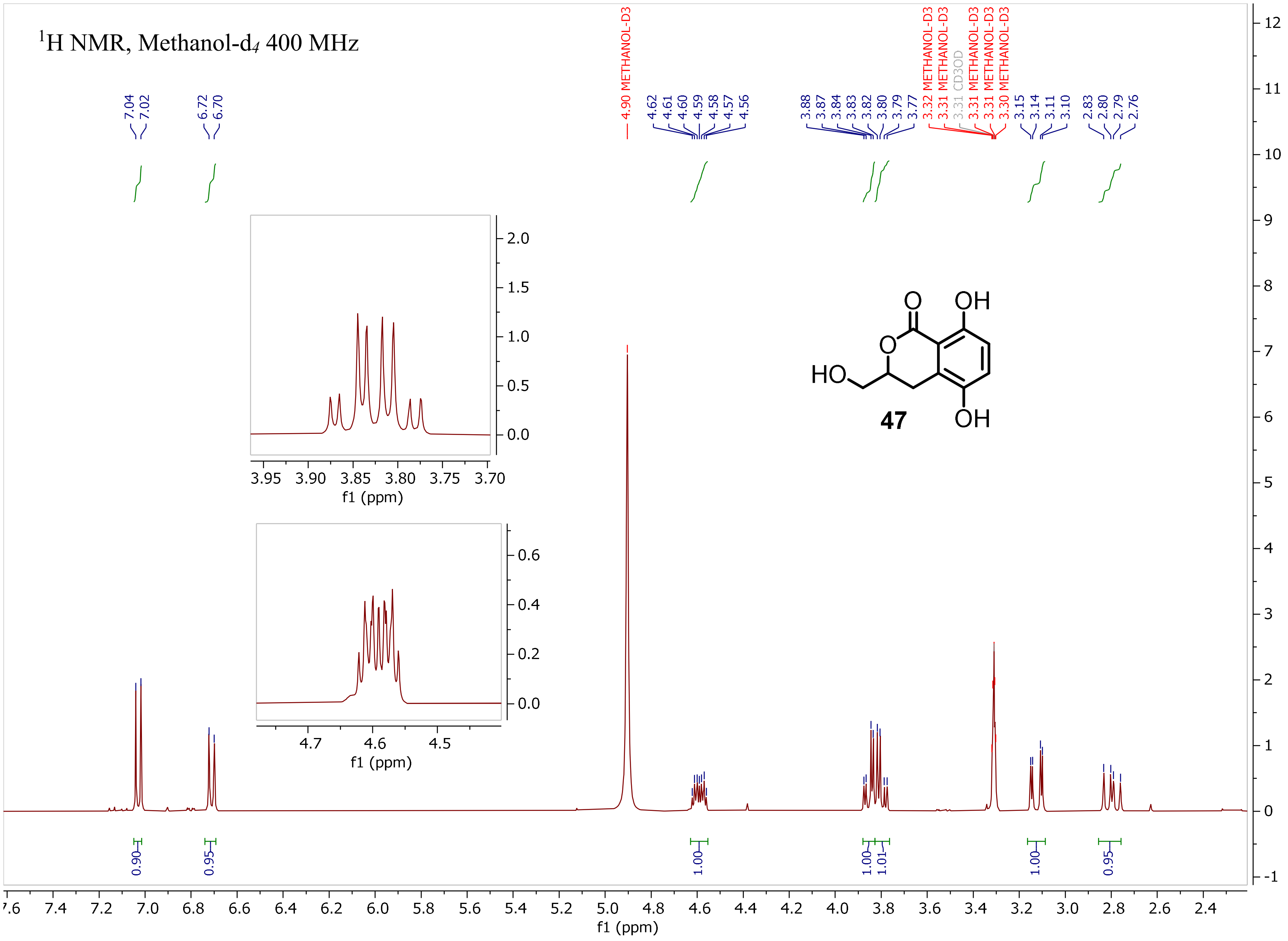

1H NMR, Methanol-d4 400 MHz
7.04
7.02
6.72
6.70
4.90 METHANOL-D3
4.62
4.61
4.60
4.59
4.58
4.57
4.56
3.88
3.87
3.84
3.83
3.82
3.80
3.79
3.77
3.32 METHANOL-D3
3.31 METHANOL-D3
3.31 CD3OD
3.31 METHANOL-D3
3.31 METHANOL-D3
3.30 METHANOL-D3
3.15
3.14
3.11
3.10
2.83
2.80
2.79
2.76
HO
O
O
OH
OH
47
0.90
0.95
1.00
1.00
1.01
1.00
0.95
f1 (ppm)

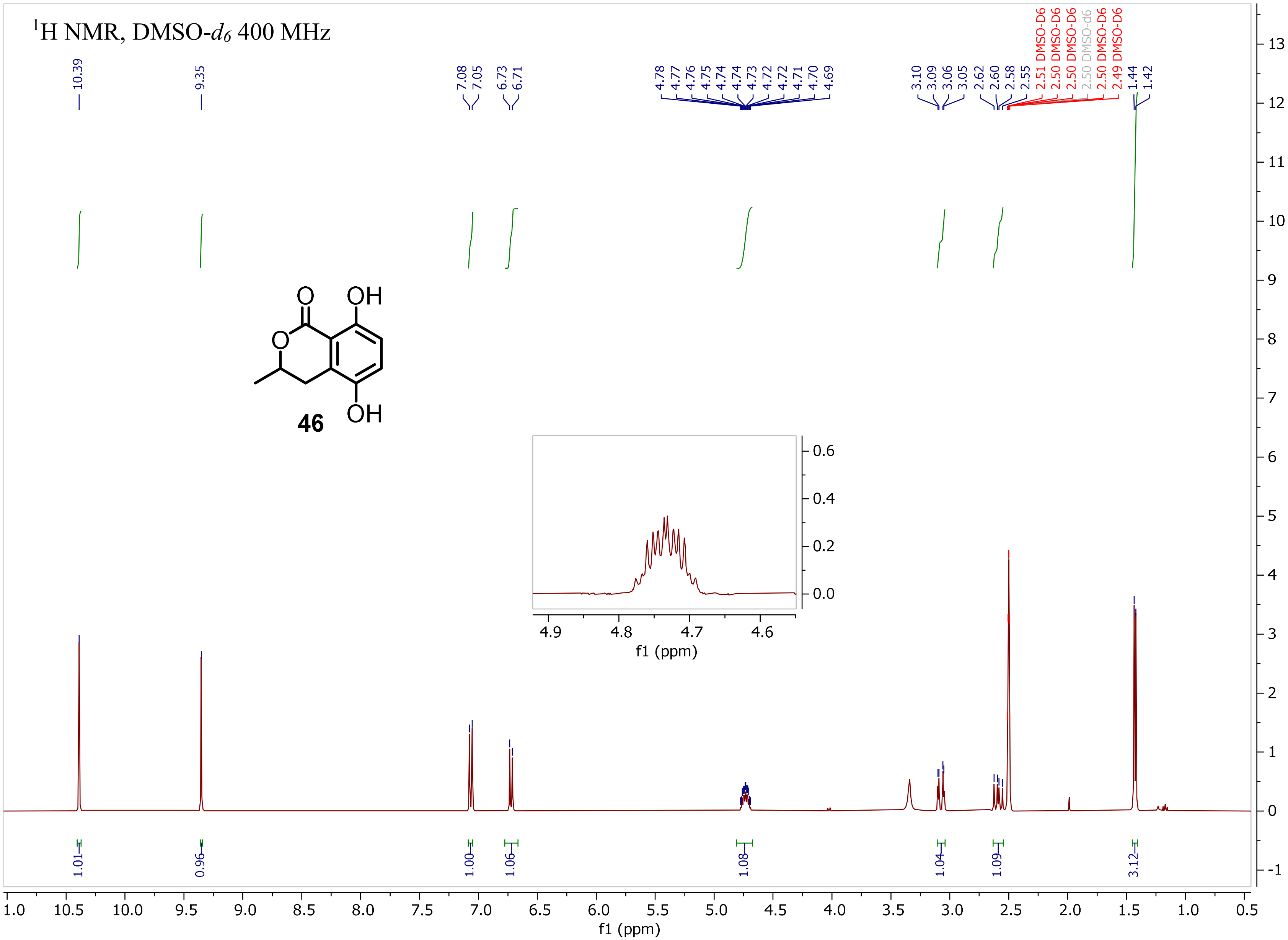

1H NMR, DMSO-d6 400 MHz
10.39
9.35
7.08
7.05
6.73
6.71
4.78
4.77
4.76
4.75
4.74
4.74
4.73
4.72
4.72
4.71
4.70
4.69
3.10
3.09
3.06
3.05
2.62
2.60
2.58
2.55
2.51 DMSO-D6
2.50 DMSO-D6
2.50 DMSO-D6
2.50 DMSO-d6
2.50 DMSO-D6
2.49 DMSO-D6
1.44
1.42
O
OH
O
OH
46
1.01
0.96
1.00
1.06
1.08
1.04
1.09
3.12
f1 (ppm)

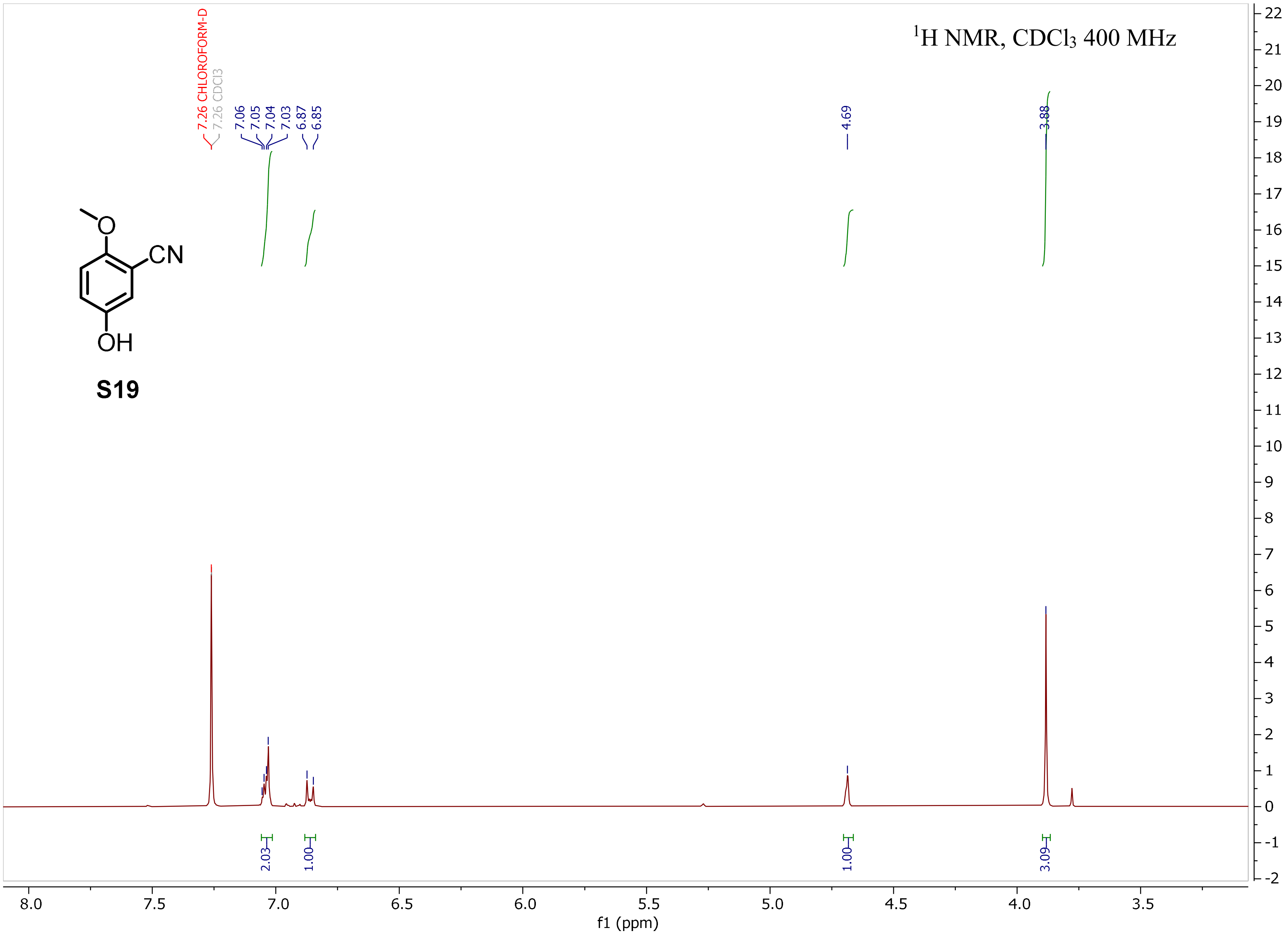

1H NMR, CDCl3 400 MHz
O
CN
OH
S19
7.26 CHLOROFORM-D
7.26 CDCl3
7.06
7.05
7.04
7.03
6.87
6.85
4.69
3.88
2.03
1.00
1.00
3.09
f1 (ppm)

Concentration Studies of Lactone 1 and Salicylamide 38

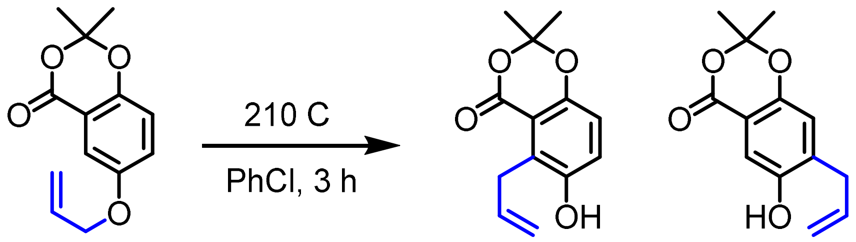

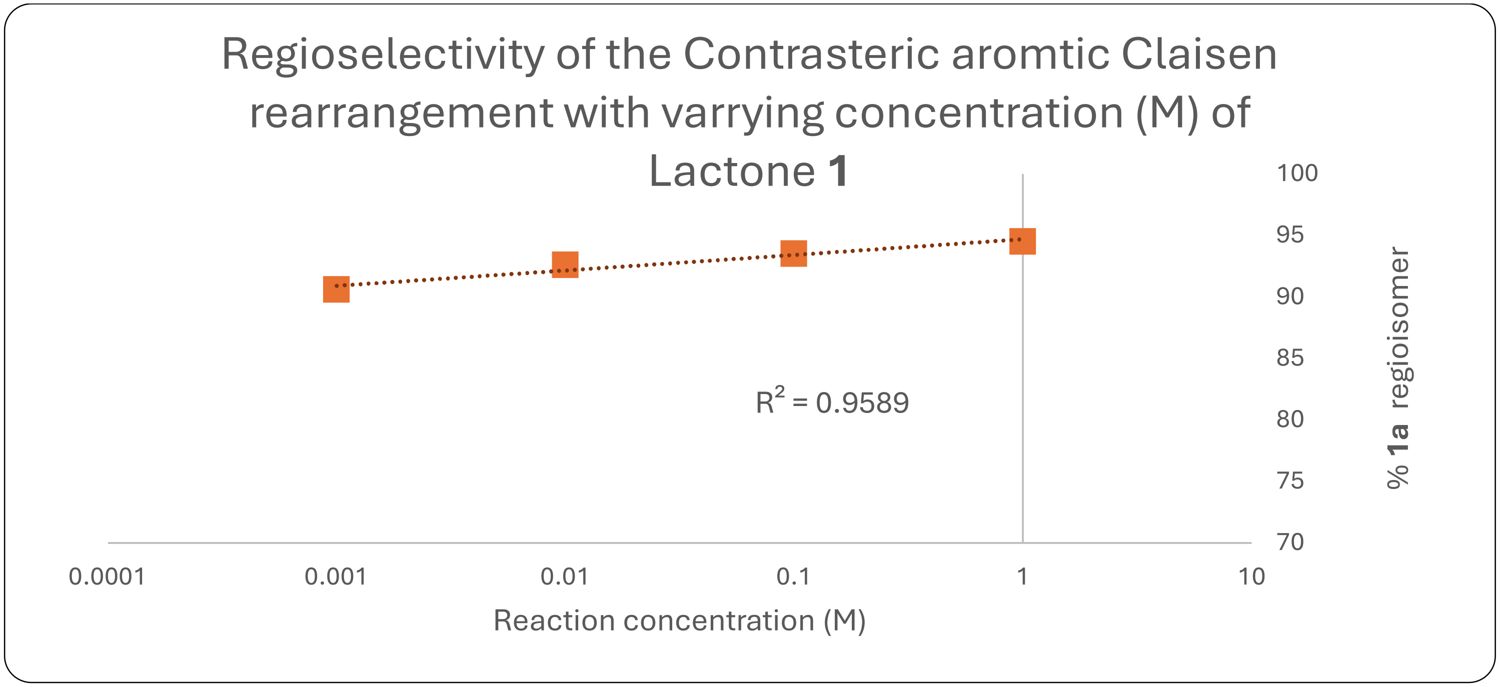

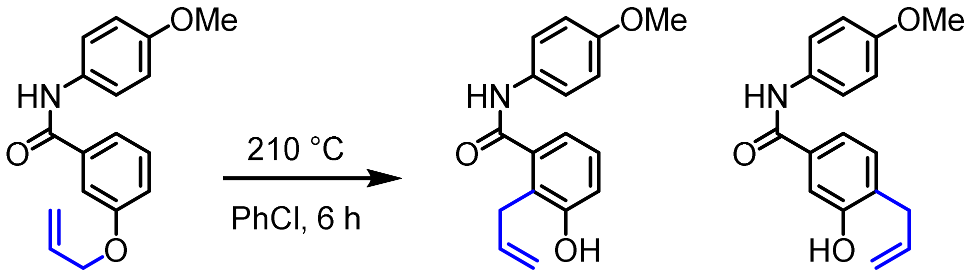
OMe
HN
O
O
210 °C
PhCl, 6 h
OMe
HN
O
OH
OMe
HN
O
HO

38 **38a** **38b**

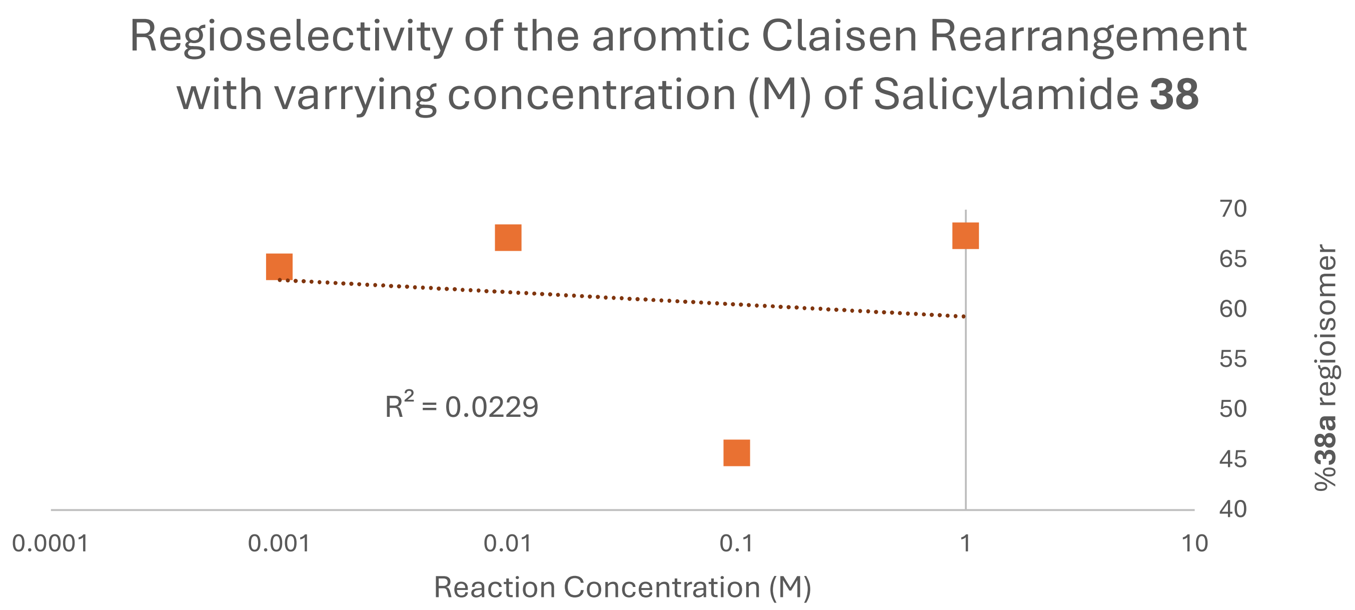
Regioselectivity of the aromtic Claisen Rearrangement
with varrying concentration (M) of Salicylamide 38
70
65
60
55
50
45
40
%38a regioisomer
R² = 0.0229
0.0001
0.001
0.01
0.1
1
10
Reaction Concentration (M)
